# MYB44-ENAP1/2 restricts HDT4 to regulate drought tolerance in Arabidopsis

**DOI:** 10.1371/journal.pgen.1010473

**Published:** 2022-11-22

**Authors:** Bo Zhao, Zhengyao Shao, Likai Wang, Fan Zhang, Daveraj Chakravarty, Wei Zong, Juan Dong, Liang Song, Hong Qiao

**Affiliations:** 1 Department of Molecular Biosciences, The University of Texas at Austin, Austin, Texas, United States of America; 2 Department of Plant Biology, Rutgers, The State University of New Jersey, New Brunswick, New Jersey, United States of America; 3 Department of Botany, University of British Columbia, Vancouver, British Columbia, Canada; 4 Institute for Cellular and Molecular Biology, The University of Texas at Austin, Austin, Texas, United States of America; Tsinghua University, CHINA

## Abstract

Histone acetylation has been shown to involve in stress responses. However, the detailed molecular mechanisms that how histone deacetylases and transcription factors function in drought stress response remain to be understood. In this research, we show that ENAP1 and ENAP2 are positive regulators of drought tolerance in plants, and the *enap1enap2* double mutant is more sensitive to drought stress. Both ENAP1 and ENAP2 interact with MYB44, a transcription factor that interacts with histone deacetylase HDT4. Genetics data show that *myb44* null mutation enhances the sensitivity of *enap1enap2* to drought stress. Whereas, HDT4 negatively regulates plant drought response, the *hdt4* mutant represses *enap1enap2myb44* drought sensitive phenotype. In the normal condition, ENAP1/2 and MYB44 counteract the HDT4 function for the regulation of H3K27ac. Upon drought stress, the accumulation of MYB44 and reduction of HDT4 leads to the enrichment of H3K27ac and the activation of target gene expression. Overall, this research provides a novel molecular mechanism by which ENAP1, ENAP2 and MYB44 form a complex to restrict the function of HDT4 in the normal condition; under drought condition, accumulated MYB44 and reduced HDT4 lead to the elevation of H3K27ac and the expression of drought responsive genes, as a result, plants are drought tolerant.

## Author summary

Water deficiency has become one of the greatest concerns limiting sustainable crop production. Uncovering the molecular mechanisms of drought stress response has been one of hot research topics worldwide. Histone modification and transcription factors have been shown to function in the drought stress response collectively. Yet, the detailed molecular mechanisms are still remained to study. In this research, we found a novel molecular mechanism that the histone binding protein ENAP1 and ENAP2 form a complex with MYB44 to restrict the function of histone deacetylase HDT4 from regulating H3K27 acetylation in the target genes in a normal condition; Whereas, under drought condition, MYB44 proteins are accumulated, and the HDT4 proteins are reduced, leading to elevations of H3K27ac and the expression of drought responsive genes, as a result, plants are drought tolerant. This research provides a new insight of how histone deacetylase, histone binding protein and transcription factor coordinate to regulate drought stress response in plants.

## Introduction

Water deficiency has become one of the greatest concerns limiting sustainable crop production worldwide. Plants are commonly exposed to the drought stress that severely hampers plants growth and reproduction [1]. As sessile organisms, plants have developed various strategies ranging from the cellular level to the organism level to minimize the damage from drought stress. When encountering decreased water availability, plants display short-term responses such as rolling and withering leaves, reducing aperture of stomatal pores and accumulating osmolytes, and long-term responses such as strengthening and prolonging root system, depositing more wax on leaves and even advancing flowering to escape drought [2,3].

At the cellular level, plants combat the drought stress by altering gene expression, and eventually abundance of proteins and metabolites that involved in stress tolerance [4,5]. These adaptive responses can be essentially attributed to the transcriptional regulations that facilitate the accumulation of stress responsive factors, which are mainly mediated by transcription factors. Several transcription factor families such as MYB, bHLH, AP2/ERF, bZIP, NAC, DREB and WRKY, have been shown to participate in modulating plant response to drought stress [6,7]. Among them, the MYB family is one of the largest transcription factor families and a few of them show specific roles in response to drought stress [8–14]. For example, MYB15, MYB37 and MYB96 are induced by drought stress, and they act as positive regulators in response to drought stress. Their overexpression enhances ABA-induced stomatal closure and therefore promotes plant drought tolerance [15–18]. In contrast, MYB20 and MYB60 function as negative regulators for drought responses, and their mutants reinforce ABA-induced stomatal closure, conferring plants the ability to resist drought stress [19–22]. MYB44, a member of MYB subfamily 22, has been well characterized in the regulation of different biotic and abiotic stresses [8,18,23–28]. The expression of *MYB44* is induced by phytohormones like Abscisic Acid (ABA), Salicylic Acid (SA) and Jasmonic Acid (JA), and abiotic stresses such as drought and salt [18,25]. Interestingly, the water loss rate is reduced in the plants that *MYB44* are overexpressed, as a result, the plants are more drought tolerant [18]. When Arabidopsis *MYB44* is heterologously expressed in soybean and rice, the drought tolerance is significantly improved [29,30], strongly indicating that MYB44 is a positive regulator in drought stress. Yet, the molecular mechanism that how MYB44 functions in drought stress is ambiguous.

Histone acetylation has been recognized as ubiquitous marks across species to regulate gene expression [31–36]. H3K9 acetylation (ac) and H3K27 acetylation (ac) have been found highly enriched in the gene body of some drought inducible genes under stress conditions, contributing to the activation of their expression [37,38]. HDA9 functions as a negative regulator under salt or drought stress by deacetylating H3K9ac on responsive genes [39]. HDA6 acts as a key factor to switch on/off essential drought responsive network, in which plants trigger a dynamic metabolic flux conversion from glycolysis into acetate synthesis to stimulate the Jasmonic Acid (JA) signal, conferring plants drought tolerance. The HDA6 binding level is dramatically decreased after drought treatment, and the binding of HDA6 is anti-correlated with H4ac enrichment [40], suggesting the importance of HDA6 in drought stress response. Nevertheless, HDA15 is recruited by MYB96 to a subset of *ROP* genes to remove acetyl groups from both H3 and H4, resulting in the activation of ABA signal to combat drought stress [41]. Plant specific HD2 family, including HDT3 and HDT4, are involved in drought response [42,43]. Interestingly, HDT4 has been shown to deacetylate H3K27ac and play a role in drought stress response [42–45], but it remains elusive that whether HDT4 is involved in the drought stress via the regulation of histone acetylation.

Chromatin regulator ENAP1 is recently reported to function in ethylene and ABA responses by modulating histone acetylation on the ethylene or the ABA responsive genes [46–48]. In this study, we demonstrate that ENAP1 and ENAP2 are positive regulators of plant drought tolerance. Our genetics results show that *ENAP1* gain-of-function plants are more drought tolerant than Col-0, whereas the *enap1enap2* mutants are more susceptible to drought stress. We then find that ENAP1 and ENAP2 physically interact with MYB44, and the drought sensitivity is enhanced in *enap1enap2myb44*. Furthermore, we identify a histone deacetylase, HDT4, interacting with MYB44. Interestingly, the drought sensitivity of *enap1enap2myb44* is partially restored in *enap1enap2myb44hdt4* quadruple mutant. Upon drought stress, the upregulation of *MYB44* and the downregulation of *HDT4* result in the enrichments of H3K27ac on drought responsive genes, and thus the activation of gene expression. Overall, this research provides a novel molecular mechanism by which ENAP1, ENAP2 and MYB44 form a complex and restrict the function of HDT4 to deacetylate H3K27ac under the normal condition; under the drought stress condition, the accumulation of MYB44 and the reduction of HDT4 lead to the elevation of H3K27ac and the expression of drought responsive genes, as a result, plants are drought tolerant.

## Materials and methods

### Plant materials and growth conditions

All Arabidopsis plants used in this study are in Col-0 background. *35S*::*ENAP1-YFP-HA*/Col-0 (*ENAP1ox*) and *35S*::*ENAP2-YFP-HA*/Col-0 (*ENAP2ox*) have been described before [46,48]. *ENAP1ox* #1 was crossed with *myb44-1* (SALK_039074) to generated *ENAP1ox/myb44-1*. *MYB44ox* were made by Agrobacterium floral dipping with the construct of *pUBQ10*::*Myc-MYB44* and homologous single insertion lines were used for experiment. Plants used for MYB44 protein expression were generated by Agrobacterium floral dipping of *pMYB44*::*MYB44-3xMyc* in the background of *ENAP1ox* or *ENAP2ox*. *enap1-1* and *enap1-2* deletion mutants were created by CRISPR/Cas9 [48]. *enap2* refers to SALKseq_0839981.2 and SALKseq_083981.3, representing two T-DNAs inserted to *ENAP2* first exon. Double mutant *enap1-1enap2* and triple mutant *enap1-1enap2myb44-1* were generated by crossing. The triple mutant was crossed with *hdt4-1* (SALKseq_127604.1) to generated quadruple mutant *enap1-1enap2myb44-1hdt4-1*. Seeds were surface sterilized with 50% bleach containing 0.05% TritonX-100 for 7 mins and washed four times with sterile ddH_2_O, and then were grown on ½ Murashige & Skoog (MS) medium with 0.8% Agar. Medium plates with seeds were kept in the dark at 4°C for three days before being placed in the long day light condition (16 h light / 8 h dark, 22°C).

### Drought tolerance assay

Drought assay was performed as previously described [49]. Briefly, 3-day-old seedlings grown on ½ MS medium in the long day condition were transplanted to the pots containing same amount of soil. Plants were grown in 12 h light / 12 h dark at 22°C and were well watered in the first week after transplanting. Starting from the second week, water was withheld until rewatering. Pots were rotated every day to avoid positional effect. Plants were imaged every week to monitor their response to the drought stress, and rewatered until achieving a relatively low soil water content. About 3–4 days after rewatering, plants that survived were recorded. For the water loss assay, the detached whole aerial part of 3-4-week plants grown under 12 h light / 12 h dark in the soil were air dried on laboratory bench and weighted at the first 0.5 h and every 1 hour thereafter. The relative water loss was calculated as the formula, 100*(W_F.W_−W_C.W_) / W_F.W_, where W_F.W_ is the initial fresh weight, and W_C.W_ is the current weight at the indicated time point.

### Dehydration treatment

Surface sterilized seeds were grown on the cellophane membrane (RPI) that was plated on top of ½ MS medium under the long day condition (16 h light / 8 h dark, 22°C). After 10-day growth, seedlings were transferred to the filter paper saturated with ½ liquid MS containing 25% PEG8000. Seedlings on filter paper saturated with ½ liquid MS served as the mock treatment group. Seedlings treated for the indicated time were collected, quickly rinsed by clean water, fast frozen in the liquid N2 and stored in -80°C for following use.

### Root elongation

Surface sterilized seeds were grown on ½ MS plates under the long day condition (16 h light / 8 h dark, 22°C) for 2 days. The 2-day seedlings were then transferred to the ½ MS medium containing 20 μM ABA or 300 mM Mannitol and vertically grown for 7 days. Elongated primary roots were measured with ImageJ [50].

### Stomatal closure in response to ABA

Stomatal closure in response to ABA was analyzed as described previously [51]. Briefly, 3-week rosette leaves (4^th^ or 5^th^ leaf) were detached and immersed in MES buffer (10 mM MES, pH 6.15, 50 mM CaCl_2_, 10 mM KCl) for 3 h under the illumination of 100 μmol m^−2^ s^−1^ light. The leaves were then washed with ddH_2_O and incubated in 1 μM ABA solution for additional 2 h. Epidermis peels were prepared from lower side leaves and imaged with Olympus DP80. The stomatal aperture before and after ABA treatment were measured with ImageJ [50].

### Plasmid construction

For the vectors in the yeast two-hybrid, the full length CDSs of those ABA responsive transcription factors and *ENAP1* were amplified and cloned to pDBLeu and pEXPAD502 respectively to generate the BD vectors and AD-ENAP1. For the pull-down assay, the CDSs of *ENAP1*/*2*, *MYB44* and *HDT4* were ligated to pVP13 (His tag removed), pGEX-KG and pET28a respectively to generate MBP-ENAP1/2, GST-MYB44 and His-HDT4. In the BiFC assay, *HDT4* were cloned to pDEST-^GW^VYCE, and *MYB44* was cloned to pDEST-^GW^VYNE by gateway cloning. For the *MYB44* transgenic plasmid, *MYB44* full length CDS plus its 3’UTR were fused with Myc tag and driven by MYB44 or *UBQ10* promoters in the backbone of pCambia1300. For *HDT4* transgenic plasmid, *HDT4* CDS fused with FLAG was ligated to pCHF3 vector. All primers used for cloning are listed in S4 Table.

### Western blots for planta proteins

10-day-old seedlings after the treatment were fast frozen and grounded in the liquid N2. Equal amount of grounded sample powder from different treatments or genotypes were dissolved with 2x loading buffer [50 mM Tris-HCl (pH 6.8), 2% SDS, 10% glycerol, 0.01% Bromophenol Blue, and freshly added 0.4% (v/v) β-mercaptoethanol]. Total proteins were separated by SDS-PAGE, transferred to a PVDF membrane, then probed with anti-HA antibody, anti-H3, anti-FLAG, or anti-Myc. RuBisCO proteins after staining or H3 were used as the loading control.

### Gene expression analysis

Total RNA was extracted using PureLink Plant RNA Reagent according to the manufacturer’s guidelines. The first cDNA strand was reverse transcribed with ProtoScript II Reverse Transcriptase kit. The cDNA templates were diluted and subjected for the qRT-PCR in LightCycler 480-II Real-Time System with the internal control *UBQ10*. Each sample was analyzed in triplicate. The primers used in qRT-PCR were listed in S4 Table.

### Yeast Two-hybrid

The yeast two-hybrid assay was performed as described previously [52]. Briefly, the CDSs of genes of interest were cloned to AD vectors (pEXPAD502) or BD vectors (pDBLeu), AD and BD vector pairs were then co-transformed to the yeast strain AH109. The transformants were sequentially diluted and grown on drop-out media. Yeast grown on SD/-Trp-Leu drop-out media served as the loading control. The growth of yeast on SD/-Trp-Leu-His drop-out media supplemented with 3’AT (Fisher Scientific) indicated the interaction between proteins of interest.

### BiFC assay

The BiFC assay followed the protocol described previously with minor revisions [53]. Briefly, Agrobacterium (GV3101) transferred with plasmids of interest and p19 was inoculated overnight at 28°C, collected and resuspended in the fresh infiltration buffer (10 mM MES/KOH pH 5.7, 10 mM MgCl_2_, 100 μM Acetosyringone). The different BiFC partner strains and p19 strain were mixed to yield a final OD_600_ of 0.4 for each strain. Then the mixture was gently shaken at 28°C for 3 h and injected to tobacco leaves. The fluorescence of leaf discs was observed under confocal microscopy (Zesis 710) after two days of infiltration.

### Pull-down assay

*E*. *coli* expressed destination vectors were broken down with lysis buffer (20 mM Tris-HCl pH 7.5, 150 mM NaCl, 20 mM KCl, 1.5 mM MgCl_2_, 10% Glycerol, 0.05% NP-40 and freshly added 1 mM PMSF and 1x protease inhibitor cocktail) followed by 30 mins sonication. The supernatant was incubated with resin at 4°C for 4 h with gentle rotation, and then the resin was collected by centrifuge. After washing three times, destination proteins were eluted and dialyzed with amicon filters. GST-, His- and MBP- tagged proteins were purified with Glutathione Sepharose resin, Ni-NTA agarose resin and Amylose resin respectively. Proteins to be tested were incubated overnight at 4°C, then washed four times and resuspended in 2x sampling buffer for following western blot analysis.

### Co- immunoprecipitation (Co-IP) assay

Co-IP assays were performed as described before with minor revisions [54]. For Co-IP of ENAP1/2 and MYB44, transgenic plants expressing both MYB44-3xMyc and ENAP1/2-YFP-HA were used. Total crude proteins were extracted from detached leaves of three-week plants untreated (Control) and air-dried 4 h (Dehydration). For Co-IP of HDT4 and MYB44, Agrobacteria with vectors expressing VYCE-HA-HDT4 (pDEST-^GW^VYCE) and MYB44-mCherry-FLAG (modified pEarlyGate101) was mixed with a final OD_600_ 0.4 and injected to 4-6-week tobacco leaves. After two days of infiltration, the injected leaf was scissored along the central vein. One half of the leaf was fast frozen in the liquid N_2_ as the control (D0) and the other half leaf was air dried for 4 h as dehydration treatment (D4). The crude total proteins were purified and incubated with the GFP-Trap or DYKDDDDK Fab-Trap at 4°C for 2 h, washed for four times and then resuspended with 2x sampling buffer for western blot analysis. Empty trap of IgG served as the control. MYB44-3xMyc and ENAP1/2-YFP-HA were probed with anti-Myc and anti-HA. MYB44-FLAG and HA-HDT4 were detected with anti-FLAG and anti-HA.

### ChIP-qPCR assay

ChIP assays followed the processes as previously described [46]. Briefly, 10-day seedlings grown in long-day light condition (16 h light / 8 h dark, 22°C) were treated with Mock (½ liquid MS) or Dehydration (½ liquid MS + 25% PEG8000), collected and fixed with 1% formaldehyde. The chromatin was isolated and subjected for the sonication to generate DNA fragments with a size range in 300–500 bp. Solubilized chromatin was then immunoprecipitated with Protein G Dynabeads conjugated with antibodies (anti-FLAG, anti-Myc, anti-GFP and anti-H3K27ac) overnight at 4°C. The immunoprecipitated DNA was purified and analyzed with qRT-PCR. All ChIP-qPCR primers were listed in S4 Table.

### RNA-seq library construction and analysis

RNA-seq library construction followed steps previously described [55]. 10-day-old seedlings grown in the long day condition were treated with ½ liquid MS containing 25% PEG8000 (Dehydration) or only ½ liquid MS (Mock) for 4 h. All samples were then subjected for RNA extraction with PureLink Plant RNA Reagent. 1 μg total RNA was used to prepare RNA-seq library with Ultra RNA Library Prep Kit. Indexed libraries were sequenced on the platform of HiSeq2000 (Illumina). RNA-seq raw data were evaluated with FastQC and low-quality reads were removed with Trim Galore (0.6.7). The trimmed reads were then mapped to the Arabidopsis genome (TAIR10) with botwie2 (2.4.2) with default parameters [56]. Mapped reads were counted by featureCounts (Subread 2.0.1) for each gene [57]. Differentially regulated genes were identified using DESeq2 (1.32.0) with a p-value < 0.01, q-value < 0.05 and | log2(fold change) | > 1 [58]. plotPCA (DESeq2) was used to perform the PCA analysis [58]. The targets genes of ABA responsive transcription factors under the mock condition (http://neomorph.salk.edu/dev/pages/shhuang/aba_web/pages/index.php) were overlapped with ENAP1/2 specifically regulated genes, and the overlapped gene numbers and gene expression fold changes were plotted with Cytoscape [59].

### ChIP-seq data analysis

ChIP-seq of ENAP1, MYB44 and histones were obtained from the Gene Expression Omnibus (GEO) database [47,60,61]. Raw sequencing data had quality control performed with FastQC. Low quality reads were removed with Trim Galore (0.6.7) and then mapped to the Arabidopsis genome with botwie2 (2.4.2) [56]. Aligned reads were subjected for PCR duplications removal with Sambamba [62] and peak calling performed with MACS2 [63]. Peaks associated genes were identified by using ChIPseeker [64]. The ChIP-seq signals along 1 kb upstream to 1 kb downstream of ENAP1/2 specifically regulated gene TSSs were calculated with bamCompare (deepTools 3.5.1) [65], and plotted with ggplot2 in R.

## Results

### ENAP1 positively regulates plant response to the drought stress

**Fig 1:**
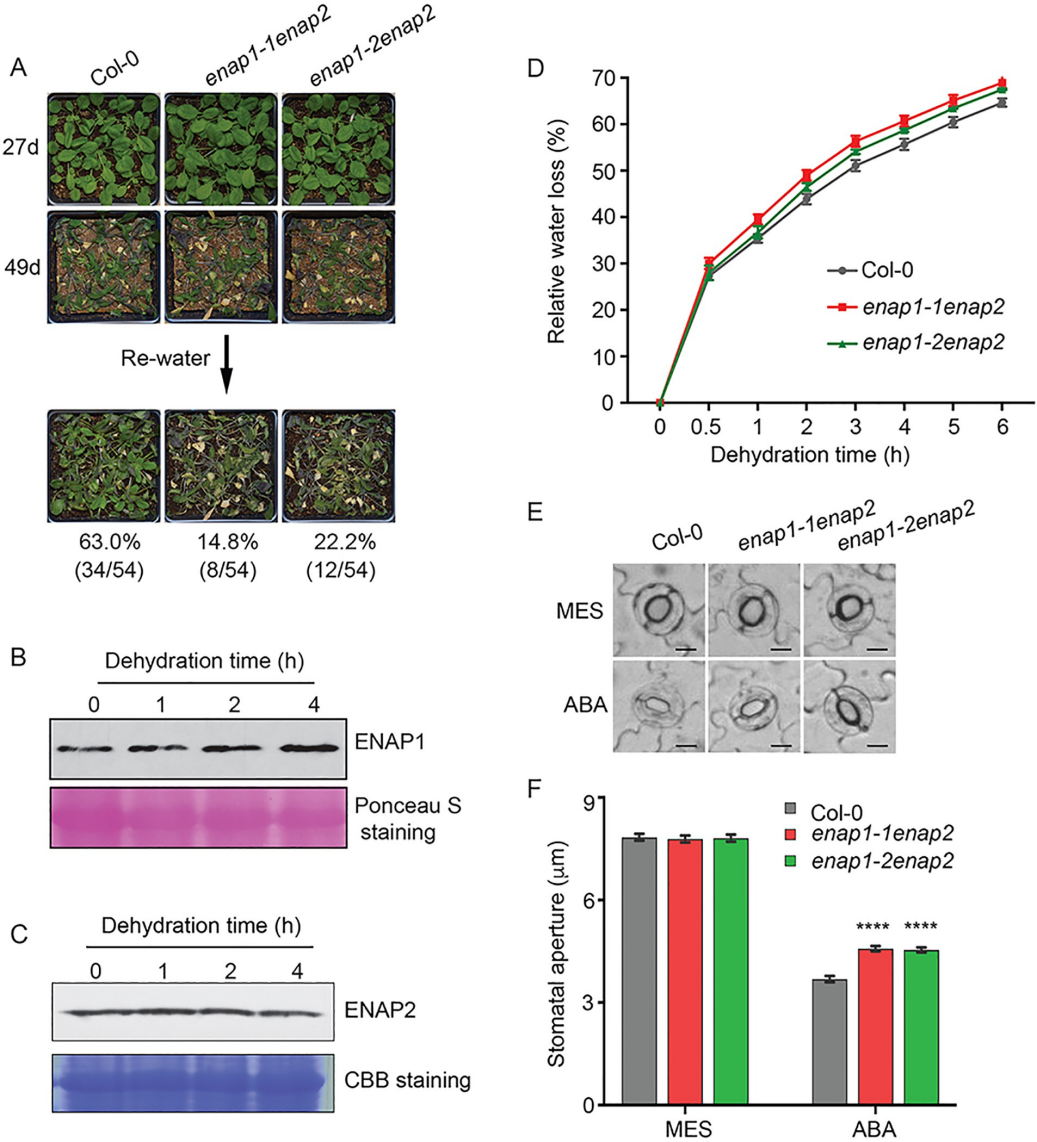
ENAP1 and ENAP2 positively regulates plant response to drought stress. (A) Drought phenotype of Col-0, *enap1-1enap2* and *enap1-2enap2*. Watering of 1-week-old plants was suspended until 49 days of growth and resumed. Survival rates (%) represented the percentage of surviving plants out of total plants in 6 independent replicates and are indicated under each plant line. (B—C) Western-blot to show the protein levels of ENAP1 (B) and ENAP2 (C) under dehydration conditions at 0, 1, 2 and 4 h. Total proteins were extracted from 10-day-old seedlings dehydrated for the indicated time. Ponceau S staining and Coomassie Brilliant Blue staining show the loading control. (D) Water loss assay of Col-0, *enap1-1enap2* and *enap1-2enap2*. Water loss was measured in detached rosette leaves from 3-week-old plants. Data are shown as mean ± SD of three replicates. (E—F) Analysis of ABA-induced stomatal closure in Col-0, *enap1-1enap2* and *enap1-2enap2*. Representative stomata (E) and the statistical results (F) are shown. 3-week-old leaves were soaked in the MES buffer under light to open the stomata, and then were transferred to 1 μM ABA solution for 2 h before being photographed. Stomata aperture before and after ABA treatment was measured with ImageJ. Data are shown as mean ± SE of three replicates (40 stomata per replicate). Stomatal aperture of *enap1-1enap2* and *enap1-2enap2* was compared to Col-0 with the unpaired and two- tailed t-test. **** *P* < 0.0001. Bars, 10 μm.

As a histone binding factor, ENAP1 is versatile enough to regulate multiple plant physiological activities [46,48,66]. Given that ENAP1 is involved in plant response to ABA, a well-established phytohormone that modulates seed germination and plant responses to environmental stresses such as drought stress, we aimed to investigate the role of ENAP1 in drought stress response. *ENAP2* (AT5G05550) was reported as the paralogous gene of *ENAP1*, and they share a high similarity in protein sequences (S1A Fig). We then obtained its T-DNA insertion lines, SALKseq_0839981.2 and SALKseq_083981.3 (S1B Fig), the expression of *ENAP2* was largely knocked down (S1C Fig). We first examined the drought phenotype of two *enap1* lines (*enap1-1* and *enap1-2*) and the *enap2* mutant. Those single mutants showed slight drought sensitive phenotypes (S1D Fig). We then generated double mutants *enap1-1enap2* and *enap1-2enap2*, and they showed an enhanced sensitivity to the drought stress even though the ENAP1 and ENAP2 protein levels were not significantly regulated by drought stress (Fig 1B and 1C). The water loss rate of *enap1enap2* mutants was higher than the wild type (Col-0) (Fig 1D). Moreover, the *ENAP1ox* plants were more tolerant to the drought stress compared to Col-0 (S1E Fig), suggesting a positive role of ENAP1 and ENAP2 in plant response to drought stress. Considering the critical role of ABA in the regulation of drought stress, we then evaluated the response of *enap1-1enap2* to ABA. No significant difference in root growth inhibition by ABA or by mannitol treatment was observed between *enap1-1enap2* and Col-0 (S1F and S1G Fig). However, the ABA-induced stomata closure in the *enap1-1enap2* and *enap1-2enap2* was compromised compared to that in Col-0 (Fig 1E and 1F), suggesting that ENAP1 and ENAP2 regulate drought stress potentially via water loss from leaves.

### ENAP1 and ENAP2 regulates drought responsive genes

**Fig 2:**
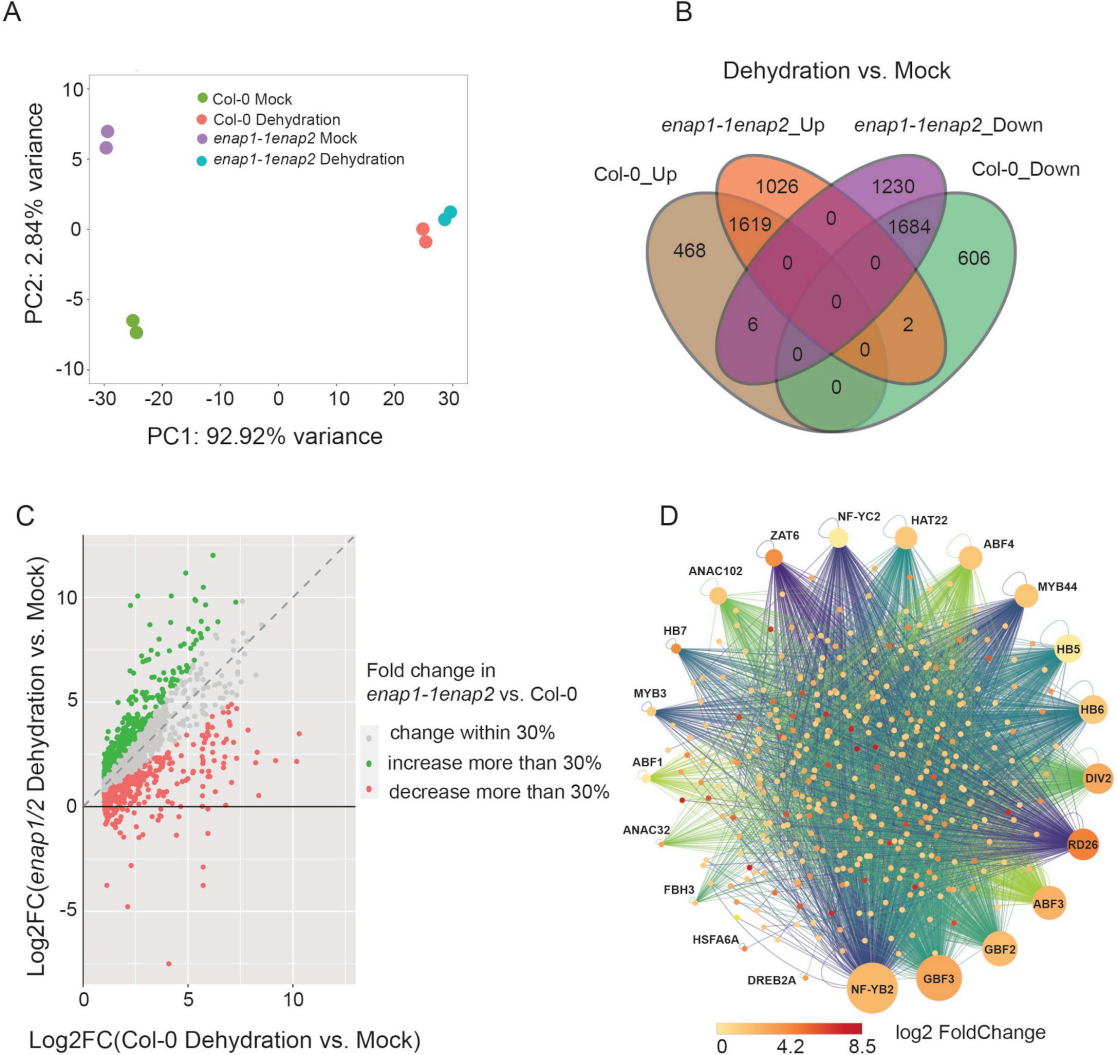
Transcriptome analysis in *enap1-1enap2* under the dehydration condition. (A) Principal component analysis of RNA-seq in Col-0 and *enap1-1enap2* under mock (½ MS) or dehydration (½ MS + 25% PEG8000) conditions. Color codes are shown. Each dot represents one sample. (B) Venn diagram to show the number of differentially regulated genes in Col-0 and *enap1-1enap2* under dehydration vs. mock treatment. (C) Dot plot to show the expression of dehydration induced genes in Col-0 and enap1-1enap2. The y-axis represents the expression levels of genes induced by dehydration in Col-0, and the x-axis represents the expression levels of these genes induced by dehydration in *enap1-1enap2*. 30% log2(FC) change of those genes in *enap1-1enap2* compared to Col-0 was used as the cutoff for classification. Color codes are shown. (D) Regulatory network of ABA responsive transcription factors. The big dots on the circle represent ABA responsive transcription factors, and the unidirectional lines show their target genes. The small dots inside the circle represent the genes that are less induced at least 30% by dehydration in *enap1-1enap2* than in Col-0. The dot color shows the log2(Fold Change) in Col-0 under dehydration vs. mock treatment. The graph is generated by Cytoscape.

To further evaluate the function of ENAP1 and ENAP2 in drought stress at the molecular level, we decided to examine the transcriptome of Col-0 and *enap1-1enap2* under the dehydration condition. To do so, we first tested the effect of dehydration stress caused by 25% PEG8000 (-0.5 MPa) [67]. Drought stress marker genes *RD29A*, *RD29B* and *RAB18* showed very strong induction upon the dehydration treatment and were completely restored after the recovery (S2A Fig), suggesting the dehydration treatment was sufficient to mimic drought stress. We then performed RNA-seq in 10-day-old seedlings treated with or without 25% PEG8000 solution for 4 hours. The sequencing reads exhibited high mapping rates and correlations (S1 Table and S2B Fig). Principal component analysis (PCA) showed a high correlation between two replications of each sample (Fig 2A), showing the reliability and reproductivity of those sequencing data. The sequencing results confirmed that *enap1-1* habitats a deletion mutation, and *ENAP2* gene expression was largely knocked down (S2C Fig). Further analysis revealed that 4385 and 5567 genes were altered by dehydration treatment in Col-0 and in the *enap1-1enap2* mutant respectively (Fig 2B, S2 and S3 Tables). GO analysis showed that dehydration induced genes were primarily involved in the response to ABA and abiotic stimuli including drought stress (S2D Fig). Moreover, a wide range of drought responsive transcription factors (TFs) were significantly induced by the dehydration treatment (S2E Fig). By comparing the drought induced genes in Col-0 and in *enap1-1enap2*, we found a large number of them were overlapped (Fig 2B). However, a significant number of genes that were less induced in *enap1-1enap2* than in Col-0 (S2C and S2F Fig). Given the positive role of ENAP1 and ENAP2 in the regulation of drought tolerance, we thus focused on these genes that were less induced by dehydration in the *enap1-1enap2* double mutant.

Our previous studies have shown that ENAP1 is a histone binding protein that functions together with transcription factors to regulate gene expression in different context [46,48]. In view of the involvement of ENAP1 in ABA-mediated drought tolerance (Fig 1), we hypothesize that ENAP1 co-functions with ABA responsive transcription factor(s) in response to the drought stress. To test this idea, using the published ChIP-seq data [60], we compared the binding targets of ABA responsive transcription factors with those less induced genes in the *enap1-1enap2* double mutant. The results showed that the binding targets of a series of ABA responsive transcription factors were enriched in the population of genes that were less induced in *enap1-1enap2* by dehydration (Fig 2D), suggesting a possibility that ENAP1 and ENAP2 co-function with these transcription factors to regulate gene expression under drought stress.

### ENAP1 and ENAP2 physically interacts with MYB44 to regulate drought response

**Fig 3:**
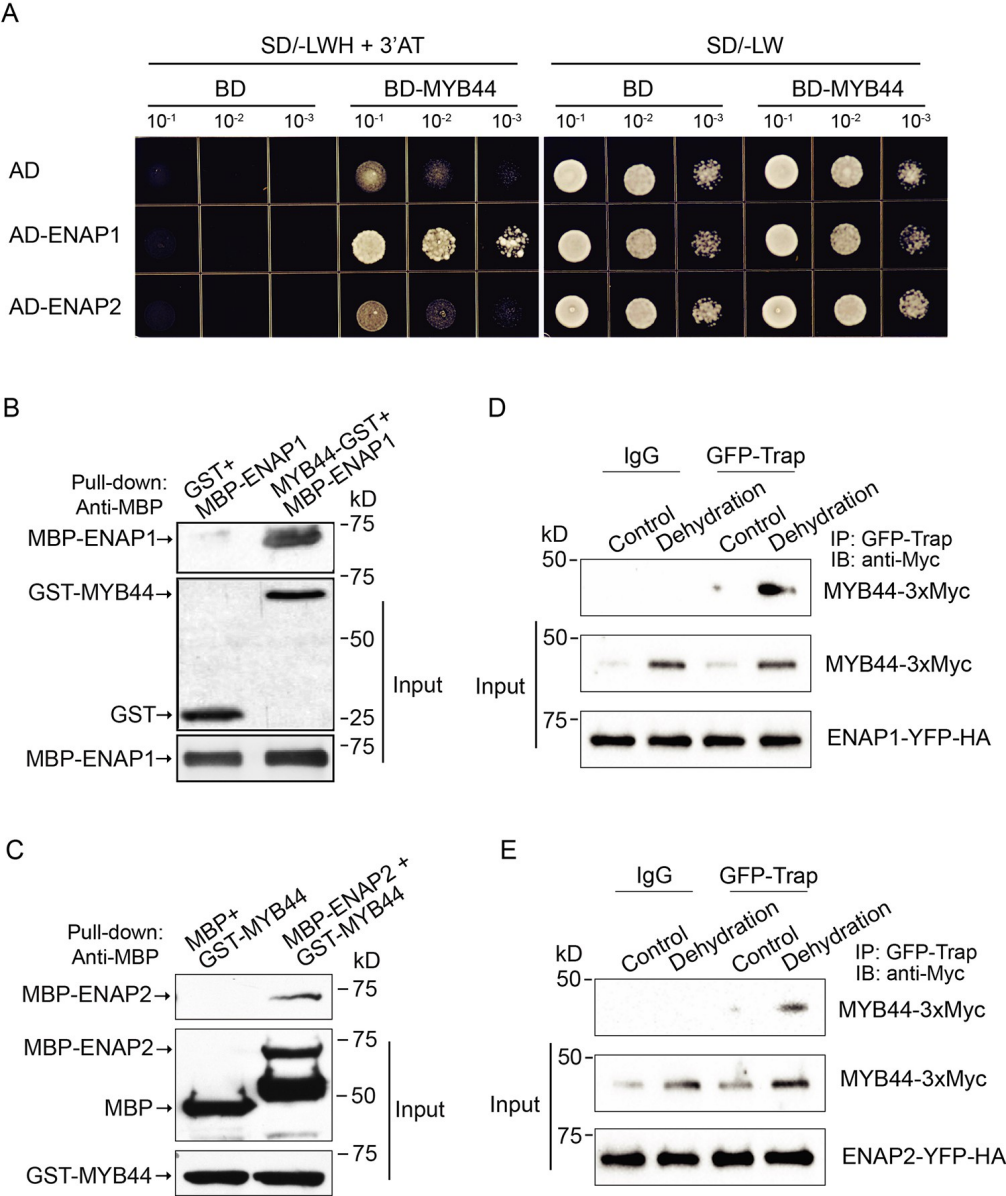
ENAP1 and ENAP2 interact with MYB44. (A) Yeast two-hybrid assay to show the interaction between ENAP1 and MYB44. The indicated constructs were co-transformed into yeast. Yeasts were grown on Leu (L), Trp (W) and His (H) drop-out medium (SD/-L-W-H) with 3’AT is to evaluate protein-protein interaction (left panel). Yeasts were grown on the Leu and Trp drop-out medium (SD/-L-W) served as the loading control (right panel). (B—C) Pull-down assay to show the interaction between ENAP1 and MYB44 (B), and the interaction between ENAP2 and MYB44 (C). GST was used as the control and GST-MYB44 was used as the bait protein. (D—E) Co-IP assay to show ENAP1 interacts with MYB44 (D), and ENAP2 interacts with MYB44 (E) *in vivo*. Detached leaves of three-week plants expressing MYB44-3xMyc and ENAP-YFP-HA were air-dried for 4 h (Dehydration) or untreated (Control) and subjected for total proteins extraction. Total proteins were immunoprecipitated with GFP trap or the empty trap (IgG). MYB44 was probed with anti-Myc and ENAP1 or ENAP2 was probed with anti-HA.

To further explore how ENAP1 plays its roles in the drought stress, we screened the interaction between ENAP1 and the ABA responsive transcription factors that had more than 100 overlapped binding targets by yeast two hybrid assay (Fig 2D). As a result, only MYB44 appeared to have a strong interaction with ENAP1 out of 14 transcription factors (Figs 3A and S3A). Interestingly, no obvious interaction between ENAP2 and MYB44 was detected (Fig 3A). But the following reciprocal *in vitro* pull-down assays showed that both ENAP1 and ENAP2 interacted with MYB44 (Figs 3C and S3B). Their interactions were further confirmed by *in vivo* co-immunoprecipitation assays (Fig 3D and 3E). MYB44 belongs to the R2R3-MYB protein subfamily that contains N-terminus DNA binding domain (R2R3) and C-terminus transcriptional regulation domain (S3D Fig). To further dissect the function of different MYB44 domains, we examined the interaction of truncated MYB44 with ENAP1 in the yeast. Interestingly, MYB44 N-terminus, but not C-terminus, showed a stronger interaction with ENAP1 than MYB44 full length protein (S3E Fig).

**Fig 4:**
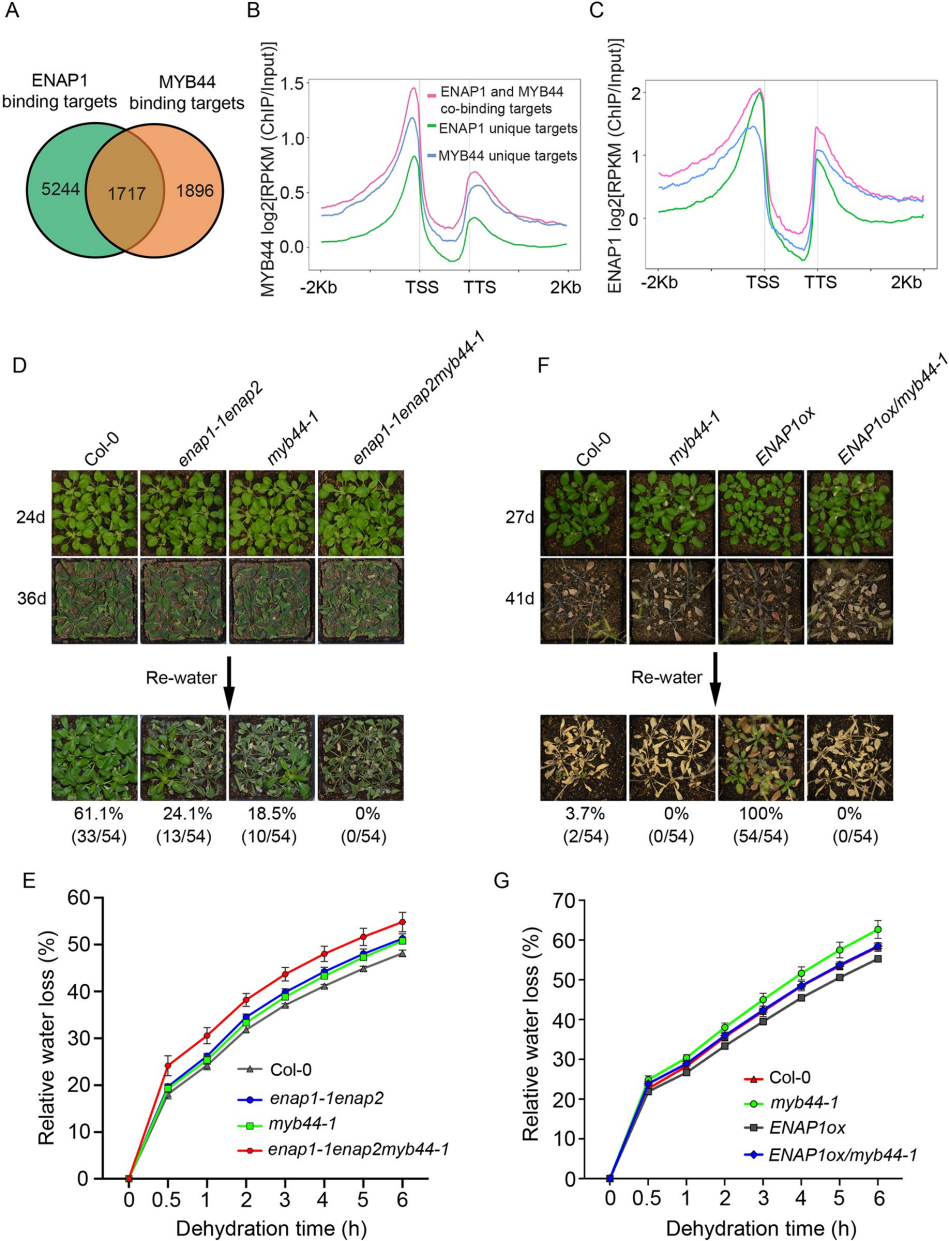
MYB44 functions synergistically with ENAP1 in response to the drought stress. (A) Venn diagram to compare the binding targets of MYB44 and ENAP1 identified from ChIP-seq. (B-C) Mean ChIP-seq signals of MYB44 (B) and ENAP1 (C). The ChIP-seq signals (Log2[RPKM(ChIP/Input)]) from 2 kb upstream of TSS to 2 kb downstream of TTS were presented. Target genes were classified as in (A), that is, MYB44 unique binding targets, ENAP1 unique binding targets and MYB44 and ENAP1 co-binding targets. (D—E) Drought phenotype of Col-0, *enap1-1enap2*, *myb44-1* and *enap1-1enap2myb44-1* (D) and Col-0, *ENAP1ox*, *myb44-1* and *ENAP1ox/myb44-1* (E). 1-week-old plants were exposed to the drought stress by withholding water for indicated days and rewatered thereafter. Survived plants were recorded 3 days after rewatering. Survival rates (%) were calculated from surviving plants out of total plants in 6 independent experiments and were indicated under each plant line. (F—G) Water loss in detached leaves of Col-0 and mutants. Rosette leaves from 3-week-old plants were used. Data represent mean ± SD in triplicate.

Next, we examined *MYB44* gene expression in response to drought. We found that the expression of *MYB44* was induced by dehydration treatment (S4A Fig). Drought response assay showed that the null *MYB44* mutant was more susceptible to drought stress than Col-0; the plants with overexpressed *MYB44* were more drought tolerant (S4B and S4C Fig), suggesting that MYB44 plays a positive role in the drought response. Given MYB44 is a transcription factor and interacts with ENAP1, we speculate that they cofunction to regulate gene expression in response to drought. To test this idea, we first compared the binding targets of MYB44 and ENAP1 using the published ChIP-seq datasets [47,60]. About 50% of MYB44 binding genes were also bound by ENAP1 (Fig 4A). They also shared a similar binding property that the binding peaks were enriched at the TSS region (Fig 4B and 4C). GO analysis showed that “response to water deprivation” and “response to abscisic acid” were overrepresented in their co-targets (S4D Fig). More interestingly, both MYB44 and ENAP1 preferred binding to their shared target genes over binding to the genes that were uniquely targeted by MYB44 or ENAP1 (Fig 4B and 4C). Specifically, the MYB44 binding signal was significantly higher on the targets that were bound by ENAP1 than those that were not bound by ENAP1 (Fig 4B), providing molecular evidence that ENAP1 enhances the binding activity of MYB44 to the target genes.

To further test whether ENAP1 and MYB44 co-function in drought stress, we generated the *enap1-1enap2myb44-1* triple mutant. The triple mutant was more susceptible to drought stress than either *enap1-1enap2* or *myb44-1* (Fig 4D). Consistently, *enap1-1enap2myb44-1* was prone to lose water faster than both *enap1-1enap2* and *myb44-1* (Fig 4E), demonstrating that ENAP1 and MYB44 function synergistically to improve plant drought tolerance. Transcription factors are essential for the regulation of gene expression, we therefore proposed that ENAP1 functions in drought stress possibly via interacting with MYB44. To test this hypothesis, we introduced *ENAP1ox* into *myb44-1* to generate *ENAP1ox/myb44-1*, and the ENAP1 protein levels in *ENAP1ox/myb44-1* were comparable to that in *ENAP1ox* (S4E Fig). However, the drought tolerance phenotype in *ENAP1ox* was completely repressed in *ENAP1ox/myb44-1* both in soil drought and water loss assays (Fig 4F and 4G). Altogether, these data demonstrated that ENAP1 and MYB44 co-function to regulate plant drought tolerance, in which MYB44 is required.

### HDT4 counteracts MYB44-ENAP1/2 to modulate drought response

**Fig 5:**
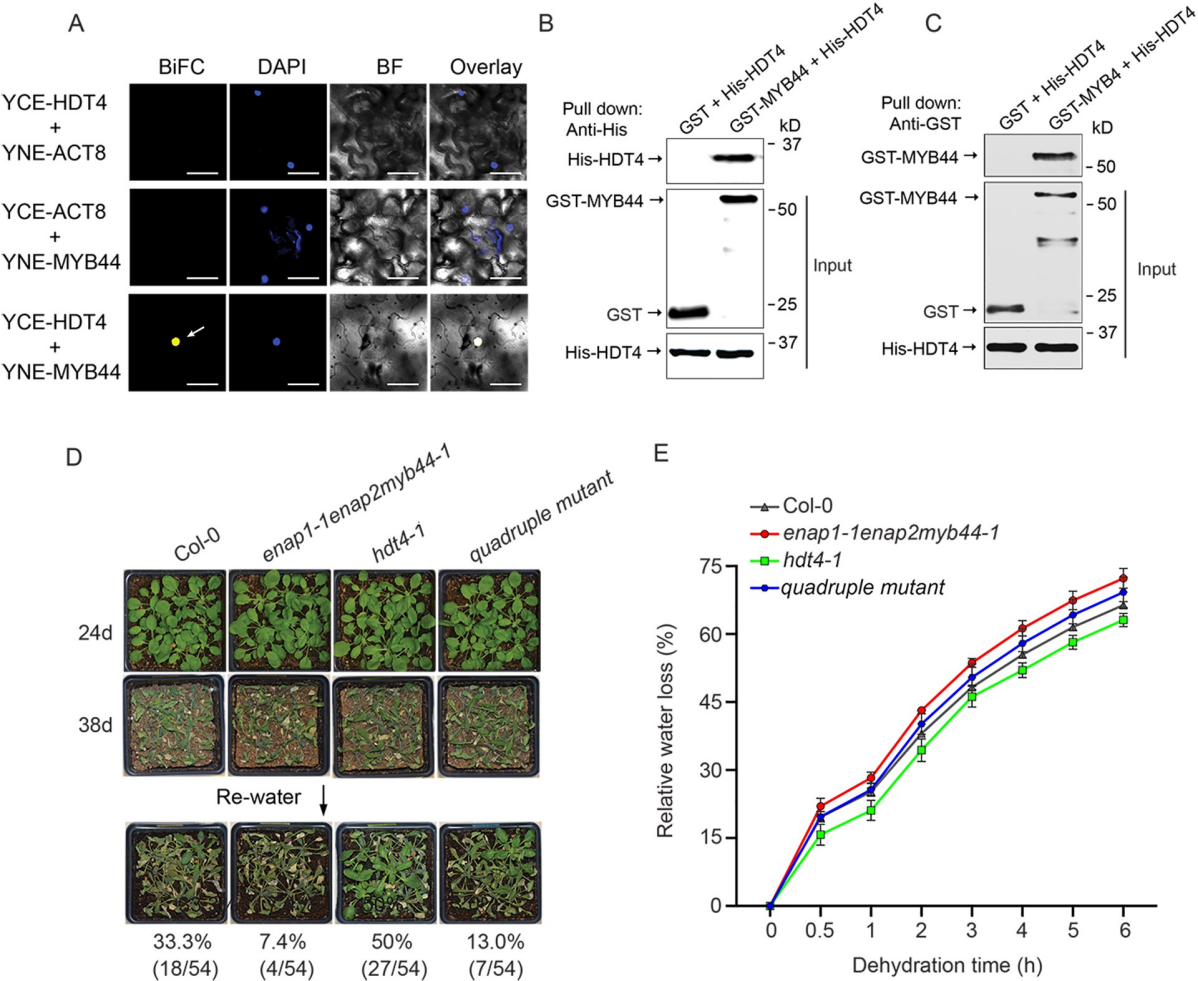
HDT4 interacts with MYB44 but negatively regulates drought response. (A) BiFC assay showing the interaction between MYB44 and HDT4. Agrobacteria containing indicated partner constructs was co-injected into tobacco leaves and the fluorescence was observed two days after infiltration. White arrow indicates the interactive complex. DAPI staining shows the nucleus. Bars, 50 μm. (B—C) Reciprocal pull-down to show MYB44 interacts with HDT4. Recombinant proteins purified from E. coli were used for the pull-down assay. GST-MYB44 (B) and His-HDT4 (C) acted as the bait protein respectively. (D) Drought phenotype of Col-0, *enap1-1enap2myb44-1*, *hdt4-1* and *enap1-1enap2myb44-1hdt4-1* (quadruple mutant). 1-week-old seedlings were subjected the drought stress by withholding water until 38 days and rewatered. Survival rates (%) represented the percentage of surviving plants out of total plants in 6 independent replicates and are indicated under each plant line. (E) Water loss of Col-0, *enap1-1enap2myb44-1*, *hdt4-1* and *enap1-1enap2myb44-1hdt4-1* (quadruple mutant). Water loss was examined in detached rosette leaves of 3-week-old plants. Data are shown as mean ± SD in triplicate.

ENAP1 was reported to enhance H3 acetylation, but whether ENAP1 is involved in the regulation of histone acetylation in the drought stress is unknown. To resolve this question, we first investigated the association of ENAP1 and MYB44 with HATs or HDACs. ENAP1 was previously found interacting with SRT1 and SRT2 [52]. However, no drought phenotype was observed in the *srt1-3srt2-1* or in the *srt1-3srt2-1* double mutant (S5A Fig). We then examined whether MYB44 interacts with any of HATs or HDACs that are known to involve in the drought stress [32]. A strong interaction between HDT4 and MYB44 was detected in the BiFC assay (Fig 5A). The reciprocal pull-down assays using GST-MYB44 and His-HDT4 as bait proteins further confirmed their interaction (Fig 5B and 5C). We then obtained *HDT4* T-DNA insertion line (*hdt4-1*) and explored its drought phenotype (S5B–S5D Fig) Fig We found that *hdt4-1* was more drought tolerant than Col-0 (Fig 5D), and *HDT4* gene expression was repressed by the dehydration treatment (S5E Fig), showing that HDT4 is a negative regulator in drought stress. To explore the genetic connections of HDT4, ENAP1/2 and MYB44, we generated *enap1-1enap2myb44-1hdt4-1* quadruple mutant. Interestingly, the drought tolerance phenotype of *hdt4-1* was repressed in *enap1-1enap2myb44-1hdt4-1* (Fig 5D). Water loss assay showed that the lower water loss rate of *hdt4-1* was restored in the quadruple mutant (Fig 5E). Altogether, these data suggest that HDT4 is a negative regulator of drought stress response and counteracts the function of MYB44-ENAP1/2 in response to drought stress.

### MYB44-ENAP1/2 restrains HDT4 from deacetylating H3K27ac

**Fig 6:**
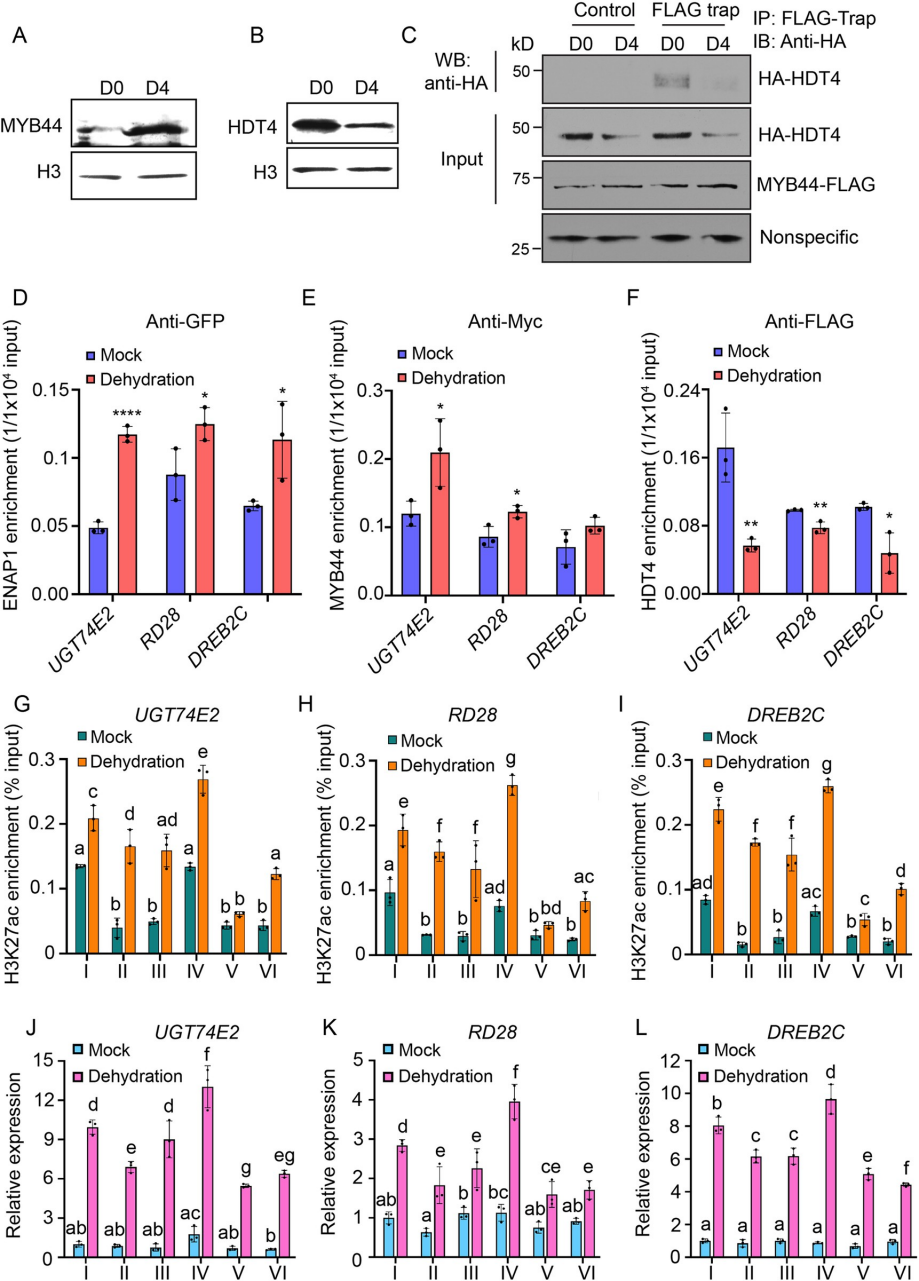
MYB44-ENAP1/2 counteracts HDT4 to regulate H3K27ac. (A—B) MYB44 (A) and HDT4 (B) protein level changes under dehydration condition. Total proteins from 10-day-old seedlings exposed to 25% PEG8000 for 0 h (D0) and 4 h (D4) were used for the western blot. H3 showed the loading. (C) Co-IP to show the interaction between MYB44 and HDT4. Agrobacteria with plasmids encoding HA-HDT4 and MYB44-FLAG was co-injected into 4-6-week tobacco leaves. Two days after the infiltration, the injected leaf was evenly scissored into two pieces. One piece was air dried for 4 h (D4) and the other piece was untreated (D0). Total proteins were immunoprecipitated with FLAG trap and the empty trap (control). Nonspecific bands indicated the loading. (D—F) ChIP-qPCR to examine the enrichment of ENAP1 (D), MYB44 (E) and HDT4 (F) on the promoter of target genes. Chromatin isolated from 10-day-old seedlings subjected for Mock and Dehydration treatments for 4 h was immunoprecipitated with anti-GFP, anti-Myc and anti-FLAG respectively. Data represent mean ± SD of three replicates. The binding of ENAP1, MYB44 and HDT4 under the Dehydration condition was compared to the Mock condition with unpaired and two- tailed t-test. *** *P* < 0.001, ** *P* < 0.01, * *P* < 0.05. (G—I) ChIP-qPCR to show the enrichment of H3K27ac on the promoter of *UGT74E2* (G), *RD28* (H) and *DREB2C* (I). Chromatin purified as in (D—F) was immunoprecipitated with anti-H3K27ac. (J—L) qPCR to show the relative expression of *UGT74E2* (J), *RD28* (K) and *DREB2C* (L) in the Col-0 and mutants. Total RNA was isolated from seedlings treated as in (D—F). Data represent mean ± SD of three replicates. Different letters represent significant differences with *P* < 0.05 in the one-way ANOVA test. I: Col-0, II: *enap1-1enap2*, III: *myb44-1*, IV: *hdt4-1*, V: *enap1-1enap2myb44-1*, VI: *enap1-1enap2myb44-1hdt4-1*.

To further explore how ENAP1, ENAP2, MYB44 and HDT4 function in drought stress, we first examined the protein levels of MYB44 and HDT4 in response to drought stress. MYB44 proteins were largely accumulated, whereas the HDT4 proteins were significantly decreased under drought stress (Fig 6A and 6B). We then checked the interaction between MYB44 and HDT4 under the drought stress condition. The interaction between them was decreased after the treatment because the reduction of HDT4 (Fig 6C). Additionally, we investigated the binding ability of ENAP1, MYB44 and HDT4 to the selected target genes that were co-bound by ENAP1 and MYB44 (S6A Fig). In line with their protein levels, the binding of ENAP1 and MYB44 was enhanced, but the binding of HDT4 was reduced by the dehydration treatment (Fig 6D–6F). Transcription factors play a critical role in collaboratively functioning with other chromatin factors by binding to DNA. We then examined ENAP1 binding activity with or without the presence of MYB44. We found that the ENAP1 binding was reduced in the absence of MYB44 (S6B–S6D Fig), suggesting that MYB44 is required for ENAP1 binding to targets.

Given the fact that ENAP1 is involved in the regulation of histone acetylation, we speculate that under the normal condition, MYB44-ENAP1/2 protein complex counteracts the function of HDT4 to maintain a basal level of histone acetylation and the expression of target genes. In the presence of drought stress, the upregulation of *MYB44* and the downregulation of *HDT4* lead to an elevation of histone acetylation and therefore the activation of target gene expression. To test this hypothesis, we explored the enrichment of various histone acetylation marks in those target genes that were specially regulated by ENAP1 and ENAP2 under drought stress by reanalyzing the published ChIP-seq data [61]. Remarkably, the H3K27ac was highly enriched over those target genes (S6E Fig). HDT4 was previously characterized as a H3K27ac deacetylase [45], and the enrichment of H3K27ac was presented on these drought induced genes [37], strongly indicating that HDT4 mediates the drought response by regulating H3K27ac. To explore this idea, we tested the levels of H3K27ac in the promoter of these selected target genes under normal and dehydration conditions. ChIP-qPCR assays showed that H3K27ac levels were significantly elevated by dehydration in Col-0, and such elevation was enhanced in *hdt4-1* but was compromised in both *enap1-1enap2* and *myb44-1* (Fig 6G–6I). Consistent with the hypersensitive drought phenotype, the H3K27ac enrichment was largely repressed under drought stress in *enap1-1enap2myb44-1* compared to Col-0, whereas the H3K27ac enrichment was partially restored in the *enap1-1enap2myb44-1hdt4-1* quadruple mutant (Fig 6G–6I). Furthermore, the expression of these target genes was positively correlated with the change of H3K27ac levels in different genetic backgrounds with or without drought stress (Fig 6J–6L). All together, these data demonstrate that HDT4 regulates H3K27ac in response to drought stress, and HDT4 is repressed during the drought stress when MYB44-ENAP1/2 promotes H3K27ac and gene expression to confer plants drought tolerance.

## Discussion

**Fig 7:**
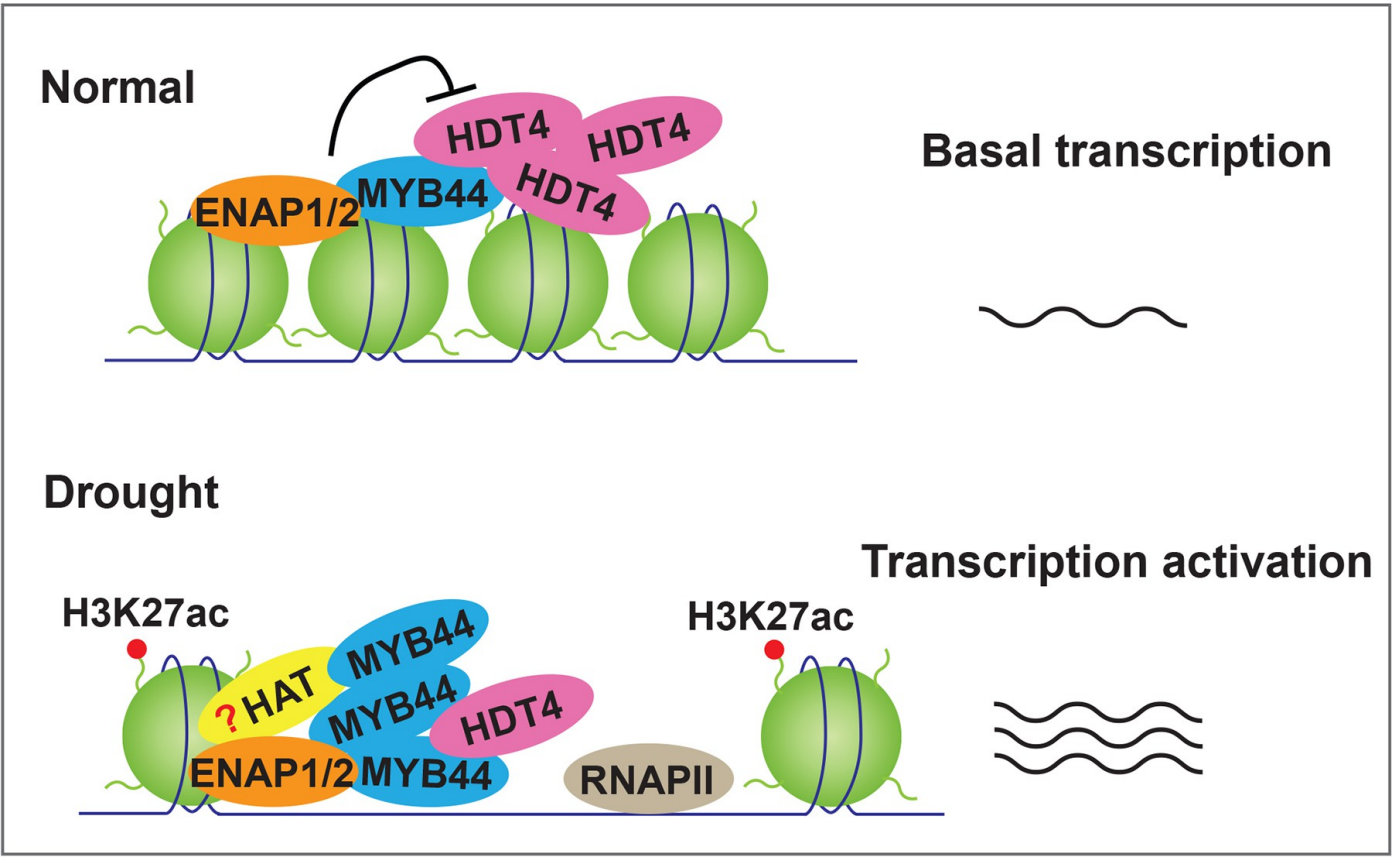
A possible model to show MYB44-ENAP1/2 restricts HDT4 to regulate drought response. Under the normal condition, ENAP1, ENAP2, MYB44 and HDT4 form a complex, and MYB44-ENAP1/2 restricts the function of HDT4 from deacetylating H3K27ac, leading to a basal level expression of target genes (upper panel); under drought stress condition, the up- regulation of *MYB44* and the down- regulation of *HDT4* lead to elevation of histone acetylation and activation of target genes, resulting in enhanced drought tolerance in plants (lower panel).

Drought stress caused by limited water availability is a major constrain of plant growth, development and reproductivity. Histone acetylation has been reported to orchestrate the transcriptional regulation to enable plants to combat drought stress [32,68]. A number of studies have focused on histone acetylation in the drought stress, however, the detailed molecular mechanisms how acetyltransferase or histone deacetylase collaborate with transcription factors to regulate drought stress remain to be understood. In this study, we show a novel molecular mechanism that the transcription factor functions together with the chromatin factor to counteract histone deacetylase to enhance drought tolerance. MYB44-ENAP1/2 acts as a counterweight of HDT4 to maintain a basal level of gene expression in the normal condition, in contrast to the condition of drought stress, when the balance is broken due to the repression of *HDT4* and the elevation of *MYB44*. As a result, the histone acetylation is elevated and the expression of their target genes is activated to for drought tolerance (Fig 7).

ABA plays a pivotal role in multiple plant physiological processes that are associated with cellular dehydration such as seed maturation and drought response [69]. Under water deficit conditions, ABA is accumulated and ABA signal is activated to induce stomatal closure and alter gene expression, thus conferring plants ability to withstand drought stress in the short term [70]. In this study, we found that *enap1enap2* showed a higher rate of detached leaves water loss, most of which is through transpiration evaporation (Fig 1D). ABA-induced stomata closure was compromised in *enap1enap2* mutants compared to Col-0 (Fig 1E and 1F), leading to a higher water loss through transpiration. Based on these results, we proposed that ENAP1 is involved in ABA-mediated plant drought tolerance. ENAP1 was also described as a positive regulator in response to ABA by associating with ABI5 during seed germination, and reacting to ethylene by interacting with EIN2 and EIN3 [46,48], indicating ENAP1 acts an important factor integrating the signals of ABA and ethylene. In support, the crosstalk of ABA and ethylene has been found converged in the regulation of stomatal movement [71]. ABA at a low concentration is proposed to promote primary root elongation and repress lateral root formation, while a higher concentration will inhibit root growth [72]. However, *enap1-1enap2* root growth was as sensitive as Col-0 in response to ABA treatment (S1F and S1G Fig), which implies that ENAP1 regulates drought stress through modulating stomatal dynamics, but not through roots. It will be a future interest to identify ENAP1 and ENAP2 targets that directly regulate stomata dynamics in drought stress.

MYB44 was shown to negatively regulate drought stress response, and *MYB44ox* exhibited a higher sensitivity to stress and a faster water loss rate than Col-0 [73]. However, in our study, we found that the plants harboring overexpressed *MYB44* showed enhanced drought tolerance, while *myb44-1* was less resistant to drought stress compared to Col-0 (S4C Fig), which is consistent with the previous report [18]. Others also found that overexpression of Arabidopsis *MYB44* in rice and in soybean led to an enhanced drought tolerance [29, 30], further suggesting the positive role of MYB44 in drought stress. MYB44 was previously proposed to activate transcription [26]. The transcriptional activation of MYB44 C-terminus was confirmed in the yeast [74]. Moreover, MYB44 directly activates *WRKY70* for the defense of a necrotrophic pathogen [25]. The ortholog protein of MYB44 from *Solanum melongena* (SmMYB44) was found to directly activate *SmSPDS* to protect eggplant from *R*. *solanacearum* [75]. However, some studies in Arabidopsis and other species characterized MYB44 as a transcriptional repressor [18,73,76,77]. Specifically, MYB44 repressed the expression of ABA induced genes under stress conditions [18,73]. One possible reason is that the transcriptional activity of MYB44 is determined by the contexts *in vivo*. Further *in vivo* studies in different stimulus or environment cues will provide more insights into the transcriptional activity of MYB44.

Histone marks are maintained by histone modifying enzymes that covalently add or remove those modifications. Histone acetylation, dynamically controlled by histone acetyltransferases (HATs) and histone deacetylases (HDACs), is generally associated with transcriptional activation due to the permissive chromatin resulting from the charge neutralization of histones [35,68]. Of those histone modifiers, HDA6, HDA9 and HDA15 have been well characterized for their negative roles in the drought stress generally through removing the acetyl groups from H3 or H4 on the target genes [39–41,78–80]. HDT4 was reported as a deacetylase of H3K27ac [45]. In our study, we found HDT4 is involved in drought stress through the regulation of H3K27ac. However, we noticed that the increased H3K27ac enrichments in *hdt4-1* were only detected under drought stress condition rather than the normal condition. This result suggests a possibility that only when MYB44 is accumulated and HDT4 is reduced under drought condition, HAT(s) are able to be recruited to the target genes to deposit H3K27ac. Whereas, in the normal condition the MYB44-ENAP1/2 might obstacle or expel HDT4 from binding the targets and therefore restrains the function of HDT4 to maintain the basal level of H3K27ac. As a result, no significant difference of H3K27ac levels was detected in the *hdt4-1* mutant compared to Col-0 in the normal condition (Fig 6G–6I). Therefore, further investigation of HAT(s) involved in MYB44-ENAP1/2 mediated drought response will be of our interest in the future.

HDT4 belongs to plant specific HD2 family of HDAC proteins, and plants overexpressing *HDT4* displayed improved drought tolerance [43]. However, our study showed that *hdt4-1* plants were more tolerant to drought stress than Col-0 (Fig 5D). Moreover, the gene expression of *HDT4* was repressed by ABA [44], and the levels of *HDT4* transcripts and proteins were decreased under dehydration treatment (Figs 6B and S5E), which support the conclusion that HDT4 negatively regulates plant drought tolerance. These results from the loss-of-function and the gain-of-function of *HDT4* suggest that the expression of *HDT4* is delicately regulated to maintain its normal function in plants. Either the deficiency or over accumulation of *HDT4* transcripts will leads plants to be more drought stress tolerant. However, the underlying molecular mechanisms that govern the drought stress response could be different. It will be a very interesting biological question to address how histone modifiers are transcriptionally fine tunned to function at a right timing and a right place.

## Supporting information

S1 Fig ENAP1 and ENAP2 positively regulate drought response. (A) Phylogenetic analysis of SANT domain containing proteins in Arabidopsis. ENAP1: AT3G11100; ENAP2: AT5G05550. (B) Schematic diagram of the T-DNA insertions in *ENAP2* gene. Black triangles represent the T-DNA, and red arrows show the insertion direction. Black filled boxes indicate the exons, and the open box indicates intron. The red arrows indicate the primer pair (F + R) used for RT-PCR in (C). (C) RT-PCR to show *ENAP2* gene expression. Total RNA was harvested from 10-day-old seedlings and subjected for RT-PCR. *UBQ10* served as an internal control. (D—E) Drought phenotype of the *enap1* and *enap2* single mutants (D) and the *ENAP1ox* plants (E). Plants were stopped from watering until indicated days of growth and rewatered afterwards. Survived plants were recorded, and the survival rates were indicated under each genotype. Totally 54 plants in 6 independent replicates were tested. (F—G) Plant root elongation under ABA and Mannitol treatment. 2-day-old seedlings were transferred to ½ MS medium plates containing 20 μM ABA or 300 mM Mannitol and vertically grown for 7 days. Then the seedlings were photographed (F) and the elongated primary roots were measured with ImageJ (G).
(JPG)

S2 Fig ENAP1 and ENAP2 regulate drought responsive genes. (A) The expression of drought marker genes in response to dehydration treatment. Total RNA was extracted from 10-day-old seedlings treated with ½ MS containing 25% PEG8000 for 0, 1, 2 and 4h (D0, D1, D2 and D4) and recovered thereafter in ½ MS for 2 and 4h (R2 and R4), and then subjected for the qRT-PCR. *UBQ10* was used as the internal control, and the relative expression was calculated by normalizing to D0. Data represent mean ± SD in three replicates. (B) Pearson correlation between each sequencing sample. The overall Pearson correlation derived from reads coverage that was calculated from consecutive equal bins (10 kb) along the genome. A higher correlation indicates a higher similarity between each sample pair. (C) The genome browser to show the reads coverage of *ENAP1* and *ENAP2* transcripts in Col-0 and in *enap1-1enap2*. Two biological replicates of each sample were shown. The open dashed box indicates the deletion in *enap1-1*. (D) GO analysis of all dehydration inducible genes in Col-0. (E) The distribution of dehydration induced transcription factors in each TF protein family. Totally 285 transcription factors were induced by dehydration treatment in Col-0. (F) Violin plot to show genes that were 30% less induced by dehydration in *enap1-1enap2* compared to Col-0.
(JPG)

S3 Fig MYB44 interacts with ENAP1. (A) Yeast two-hybrid to screen ABA responsive transcription factors. Yeast transformant from each AD and BD vector pair was sequentially diluted and placed on the three drop-out medium (left panel, L: Leu, W: Trp, H: His) to show the interaction, and on the two drop-out medium (right panel) to show the loading. (B—C) Pull-down assay to show the interaction between MYB44 and ENAP1 (B), or ENAP2 (C). The recombinant proteins of MBP-ENAP1, MBP-ENAP2 and GST-MYB44 purified from *E*. *coli* were used for the *in vitro* pull-down assay, and MBP and GST protein served as the control. MBP-ENAP1 and MBP-ENAP2 were used as the bait protein respectively. (D) Schematic diagram to show the MYB44 protein domains and truncated forms. R2 and R3 consist of the MYB domain in MYB44. (E) Yeast two-hybrid assay to show the interaction between ENAP1 and truncated MYB44. The N- and C- terminus of MYB44 diagramed in (D) were used. Yeast growth on the three drop-out medium (left panel) indicated the protein-protein interaction, and on the two drop-out medium (right panel) to show the loading.
(JPG)

S4 Fig MYB44 acts as a positive regulator in drought stress. (A—B) qRT-PCR showing the expression of *MYB44* in response to dehydration (A) and in *MYB44ox* plants (B). Total RNA was extracted from 10-day-old seedlings treated as in S2A Fig. Data represent the mean ± SD in triplicate. The expression data were compared to Col-0 D0 in (A) and Col-0 in (B) with the unpaired and two- tailed t-test. **** *P* < 0.0001, *** *P* < 0.001. (C) Drought phenotype of two *MYB44ox* lines. Plants of Col-0, *myb44-1* and *MYB44ox* were stopped from watering until 38^th^ day of growth and rewatered afterwards. Plants survival rates were recorded and indicated under each genotype. (D) GO analysis of ENAP1 and MYB44 co-target genes. (E) Western blot to show ENAP1 protein levels in *ENAP1ox* and *ENAP1ox/myb44-1*. Total proteins were harvested from 10-day-old seedlings. ENAP1 proteins were detected with anti-HA, and histone H3 served as a loading control.
(JPG)

S5 Fig *HDT4* gene expression is repressed by dehydration. (A) Drought phenotype of *srt1srt2* mutants. One of the representative repeats was imaged to show plants before and after rewatering. The survival plants out of 54 plants (6 independent replicates) were recorded. (B) Schematic diagram of the T-DNA insertion of HDT4. The filled boxes represent exon, and the open boxes represent intron. Primers used for (C) and (D) are also shown. (C—D) RT-PCR (C) and qRT-PCR (D) to show the expression of *HDT4* in *hdt4-1*.n10-day-old seedlings of Col-0 and *hdt4-1* were used for RNA extraction. Data in qRT-PCR are shown as mean ± SD. *HDT4* gene expression *hdt4-1* was compared to Col-0 with unpaired and two-tailed t-test. **** *P* < 0.0001. (E) RT-PCR to show the expression of *HDT4* in response to dehydration. Plants were sampled as in S4A Fig. *UBQ10* served as the loading control.
(JPG)

S6 Fig H3K27ac is highly enriched in ENAP1 and ENAP2 specially regulated genes. (A) The gene browser to show the binding of ENAP1 and MYB44. The red lines represent the location of primers used for ChIP-qPCR. (B—D) ChIP-qPCR to show ENAP1 enrichment on the target genes. Chromatin from 10-day-old seedlings of *ENAP1ox* and *ENAP1ox/myb44-1* with mock or dehydration treatment was immunoprecipitated with anti-GFP antibody. Data represent mean ± SD of three replicates. Different letters represent significant differences with P < 0.05 in the one-way ANOVA test. (E) Enrichment of various histone acetylation marks on ENAP1 and ENAP2 specially regulated genes. The ChIP-seq signals were calculated with bamCoverage (deepTools 3.5.1) and were plotted with ggplot2 in R along 1 kb upstream to 1 kb downstream of TSS.
(JPG)

S1 Table The mapping rates of each RNA-seq sample. (DOCX)

S2 Table Differentially regulated genes in Col-0 by dehydration. (DOCX)

S3 Table Differentially regulated genes in *enap1-1enap2* by dehydration. (DOCX)

S4 Table Primers used in this study. (DOCX)
