# Supplementary figures and images for: MYB44-ENAP1/2 restricts HDT4 to regulate drought tolerance in Arabidopsis
Source: PLoS Genet. 2022 Nov 22;18(11):e1010473. doi: 10.1371/journal.pgen.1010473 (PMC9681084; doi:10.1371/journal.pgen.1010473)

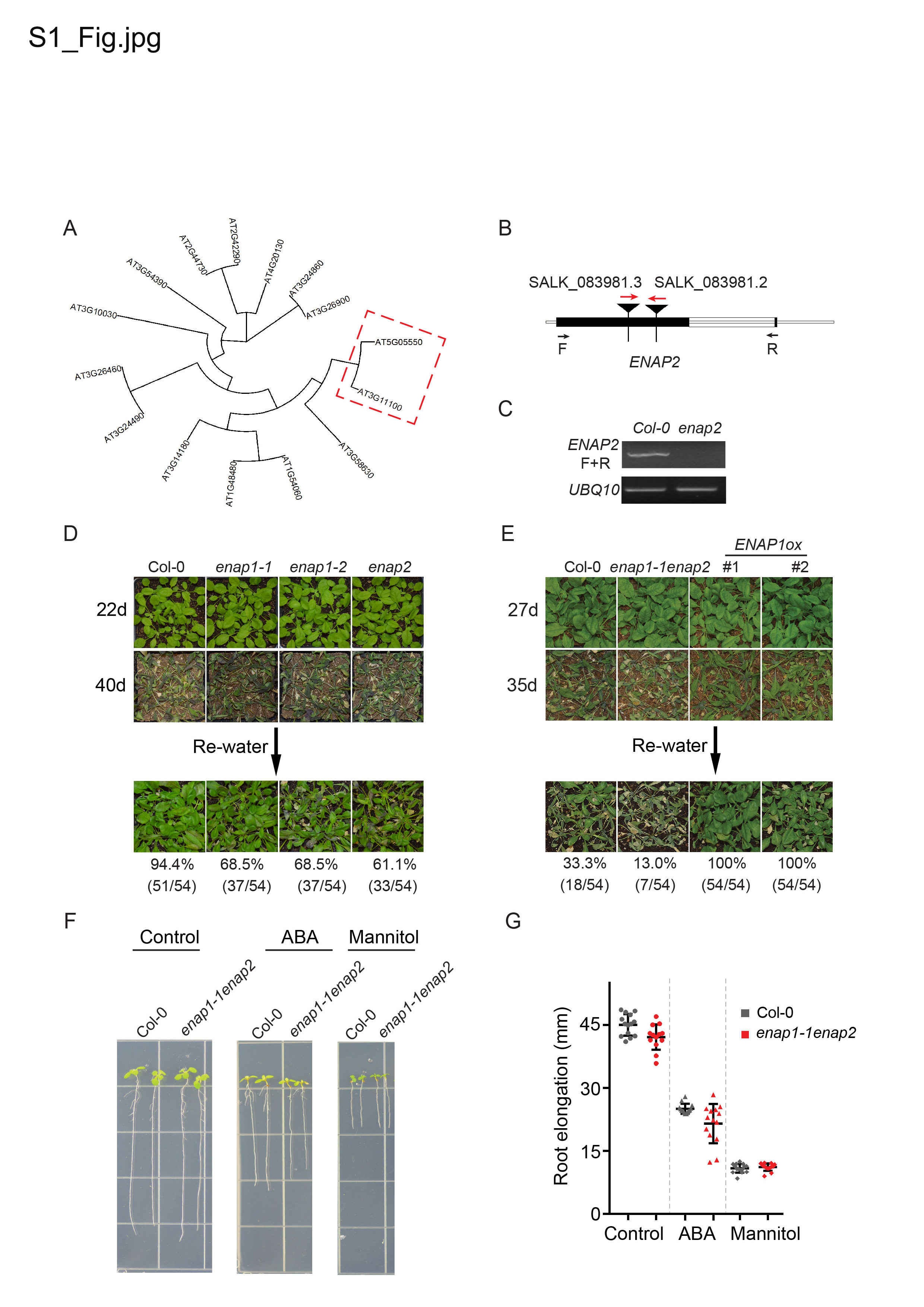

Supplement: S1 Fig — (A) Phylogenetic analysis of SANT domain containing proteins in Arabidopsis. ENAP1: AT3G11100; ENAP2: AT5G05550. (B) Schematic diagram of the T-DNA insertions in ENAP2 gene. Black triangles represent the T-DNA, and red arrows show the insertion direction. Black filled boxes indicate the exons, and the open box indicates intron. The red arrows indicate the primer pair (F + R) used for RT-PCR in (C). (C) RT-PCR to show ENAP2 gene expression. Total RNA was harvested from 10-day-old seedlings and subjected for RT-PCR. UBQ10 served as an internal control. (D—E) Drought phenotype of the enap1 and enap2 single mutants (D) and the ENAP1ox plants (E). Plants were stopped from watering until indicated days of growth and rewatered afterwards. Survived plants were recorded, and the survival rates were indicated under each genotype. Totally 54 plants in 6 independent replicates were tested. (F—G) Plant root elongation under ABA and Mannitol treatment. 2-day-old seedlings were transferred to ½ MS medium plates containing 20 μM ABA or 300 mM Mannitol and vertically grown for 7 days. Then the seedlings were photographed (F) and the elongated primary roots were measured with ImageJ (G). (JPG) [file pgen.1010473.s001.jpg]

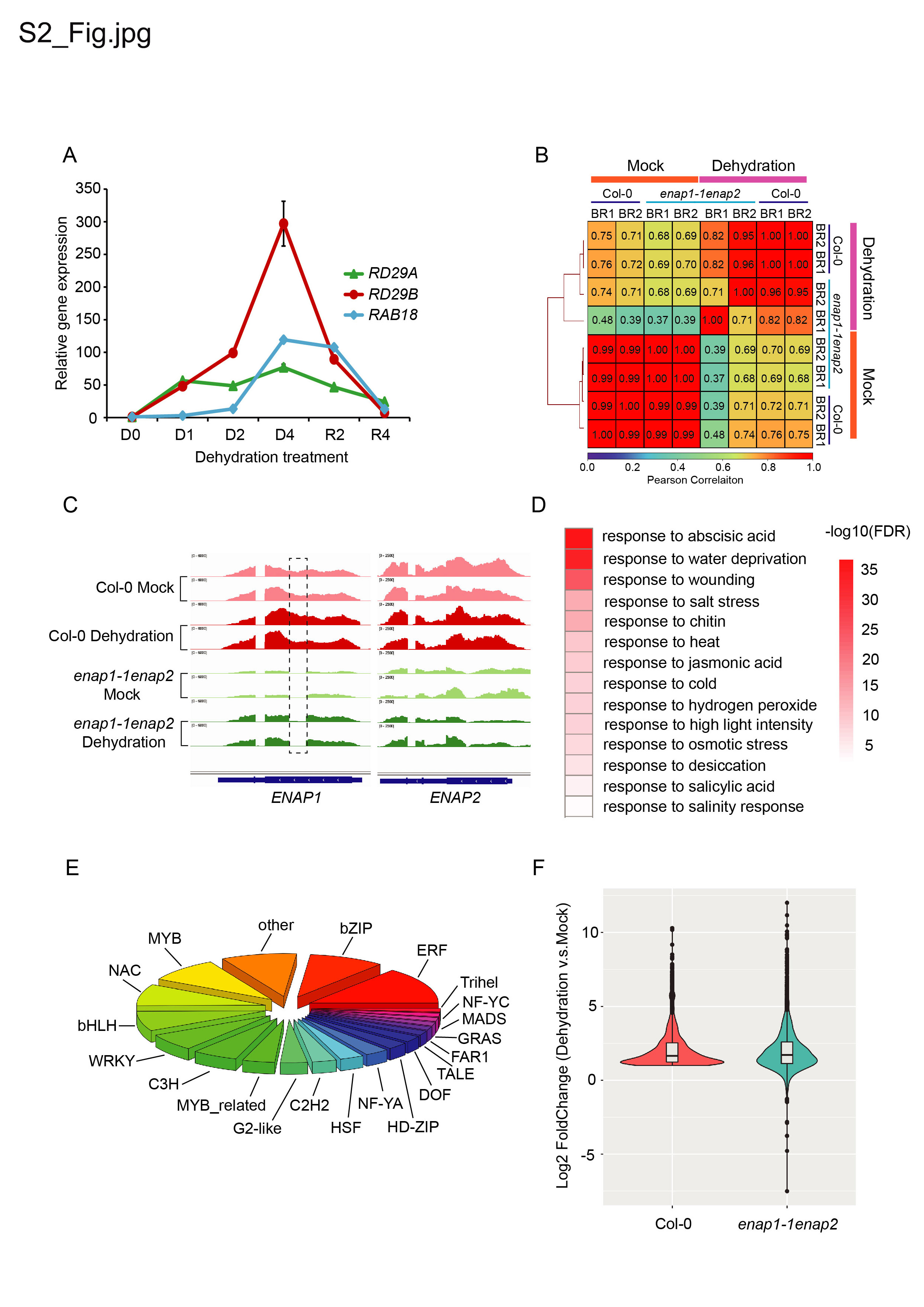

Supplement: S2 Fig — (A) The expression of drought marker genes in response to dehydration treatment. Total RNA was extracted from 10-day-old seedlings treated with ½ MS containing 25% PEG8000 for 0, 1, 2 and 4h (D0, D1, D2 and D4) and recovered thereafter in ½ MS for 2 and 4h (R2 and R4), and then subjected for the qRT-PCR. UBQ10 was used as the internal control, and the relative expression was calculated by normalizing to D0. Data represent mean ± SD in three replicates. (B) Pearson correlation between each sequencing sample. The overall Pearson correlation derived from reads coverage that was calculated from consecutive equal bins (10 kb) along the genome. A higher correlation indicates a higher similarity between each sample pair. (C) The genome browser to show the reads coverage of ENAP1 and ENAP2 transcripts in Col-0 and in enap1-1enap2. Two biological replicates of each sample were shown. The open dashed box indicates the deletion in enap1-1. (D) GO analysis of all dehydration inducible genes in Col-0. (E) The distribution of dehydration induced transcription factors in each TF protein family. Totally 285 transcription factors were induced by dehydration treatment in Col-0. (F) Violin plot to show genes that were 30% less induced by dehydration in enap1-1enap2 compared to Col-0. (JPG) [file pgen.1010473.s002.jpg]

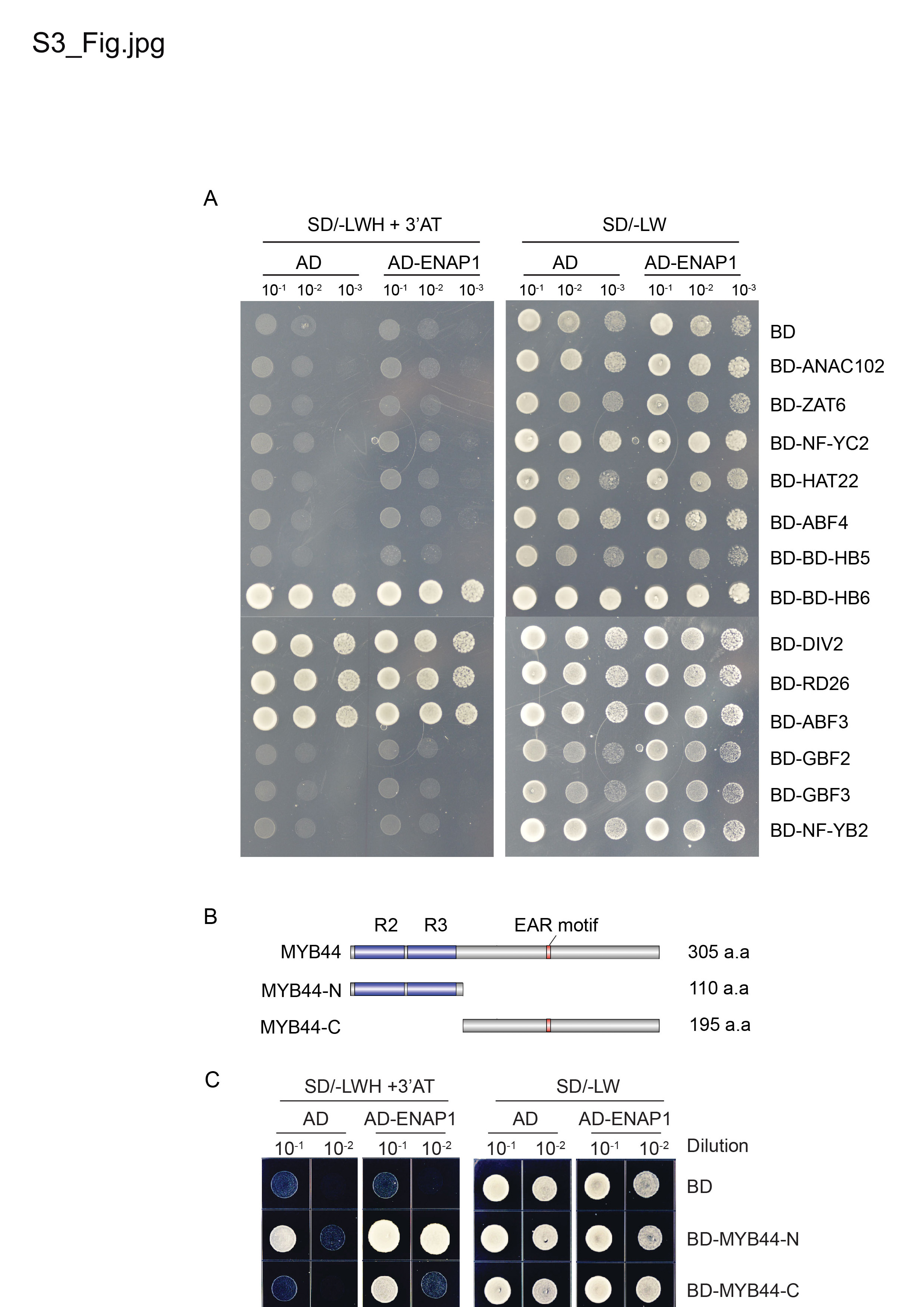

Supplement: S3 Fig — (A) Yeast two-hybrid to screen ABA responsive transcription factors. Yeast transformant from each AD and BD vector pair was sequentially diluted and placed on the three drop-out medium (left panel, L: Leu, W: Trp, H: His) to show the interaction, and on the two drop-out medium (right panel) to show the loading. (B—C) Pull-down assay to show the interaction between MYB44 and ENAP1 (B), or ENAP2 (C). The recombinant proteins of MBP-ENAP1, MBP-ENAP2 and GST-MYB44 purified from E. coli were used for the in vitro pull-down assay, and MBP and GST protein served as the control. MBP-ENAP1 and MBP-ENAP2 were used as the bait protein respectively. (D) Schematic diagram to show the MYB44 protein domains and truncated forms. R2 and R3 consist of the MYB domain in MYB44. (E) Yeast two-hybrid assay to show the interaction between ENAP1 and truncated MYB44. The N- and C- terminus of MYB44 diagramed in (D) were used. Yeast growth on the three drop-out medium (left panel) indicated the protein-protein interaction, and on the two drop-out medium (right panel) to show the loading. (JPG) [file pgen.1010473.s003.jpg]

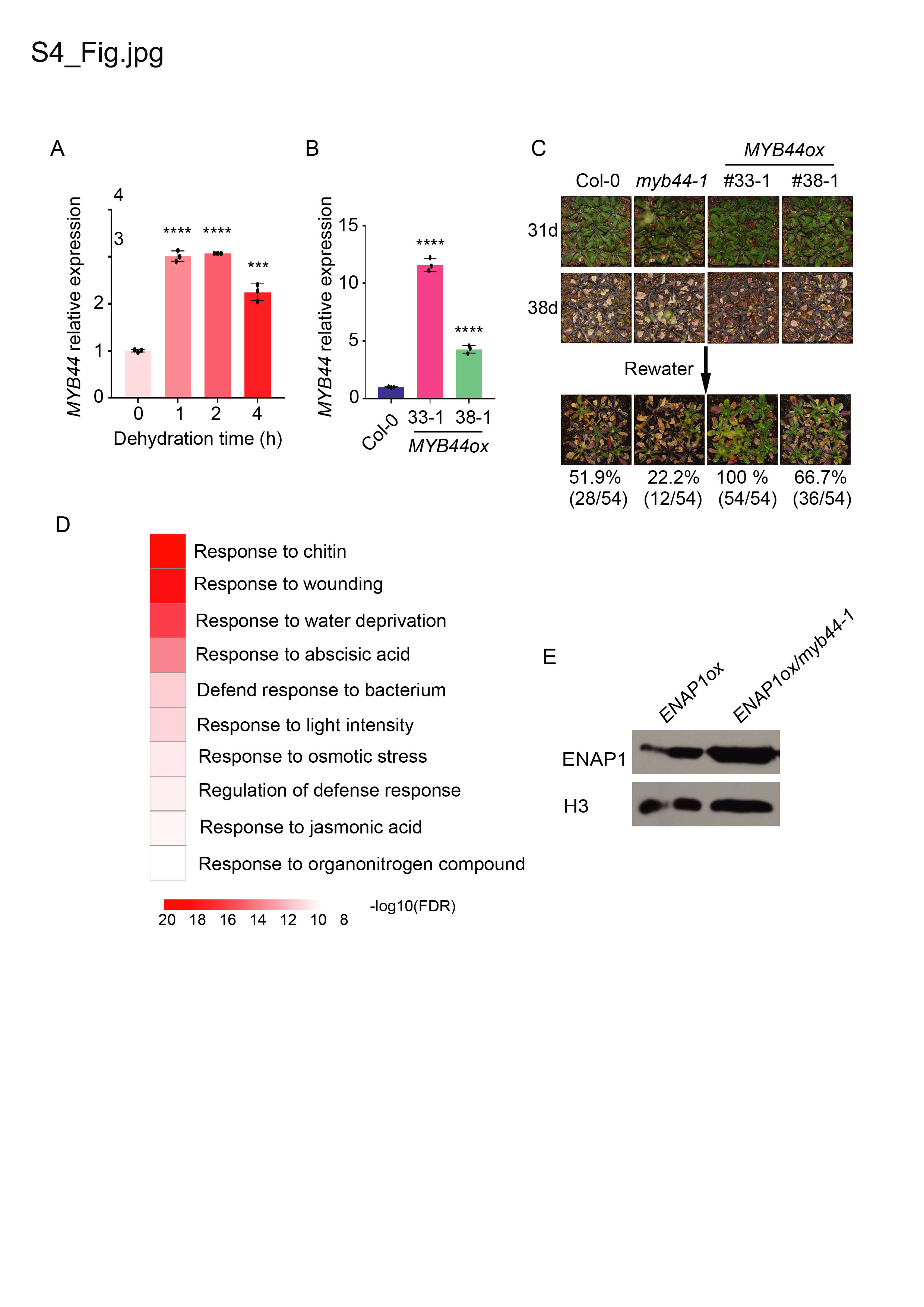

Supplement: S4 Fig — (A—B) qRT-PCR showing the expression of MYB44 in response to dehydration (A) and in MYB44ox plants (B). Total RNA was extracted from 10-day-old seedlings treated as in S2A Fig. Data represent the mean ± SD in triplicate. The expression data were compared to Col-0 D0 in (A) and Col-0 in (B) with the unpaired and two- tailed t-test. **** P < 0.0001, *** P < 0.001. (C) Drought phenotype of two MYB44ox lines. Plants of Col-0, myb44-1 and MYB44ox were stopped from watering until 38th day of growth and rewatered afterwards. Plants survival rates were recorded and indicated under each genotype. (D) GO analysis of ENAP1 and MYB44 co-target genes. (E) Western blot to show ENAP1 protein levels in ENAP1ox and ENAP1ox/myb44-1. Total proteins were harvested from 10-day-old seedlings. ENAP1 proteins were detected with anti-HA, and histone H3 served as a loading control. (JPG) [file pgen.1010473.s004.jpg]

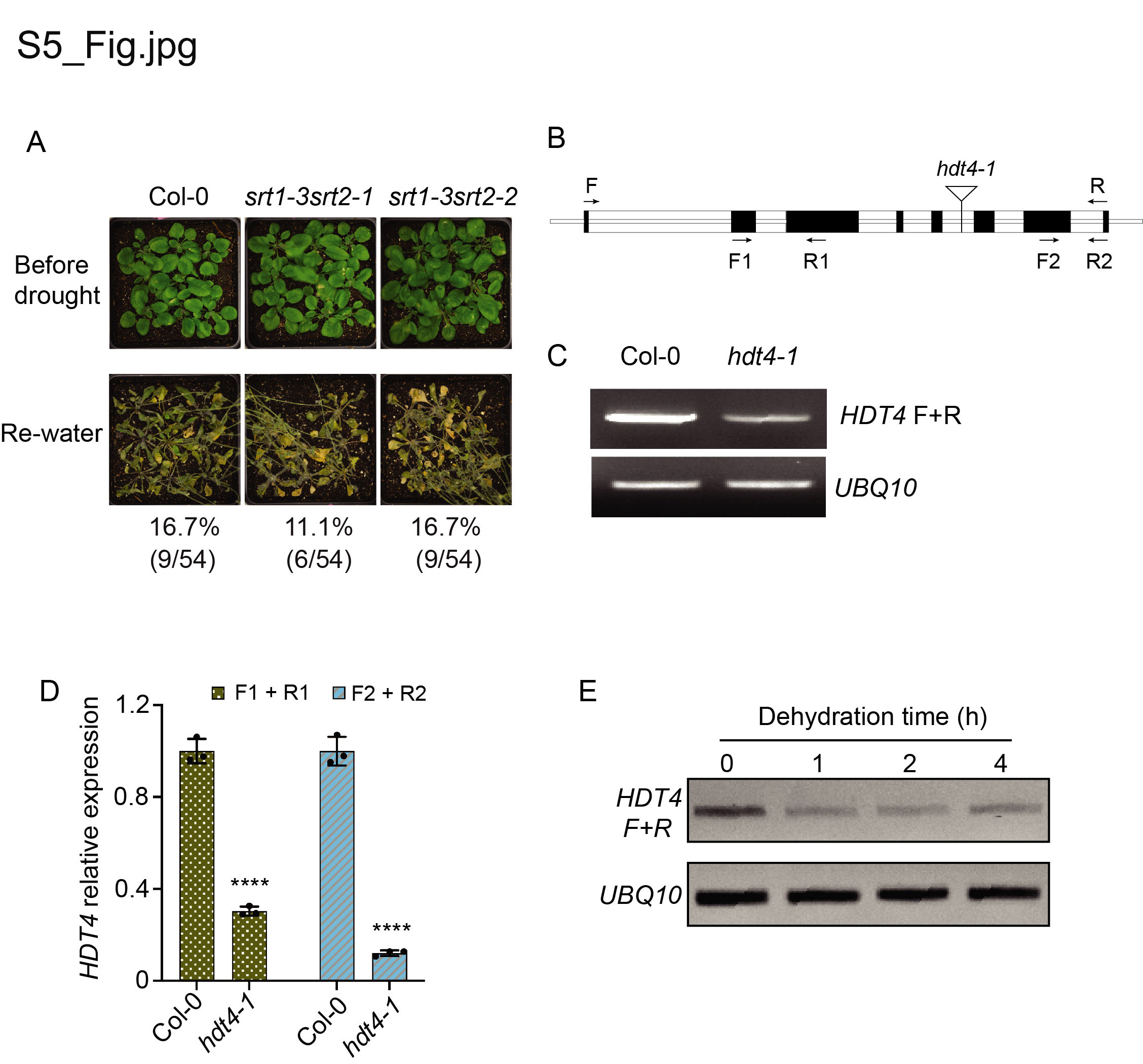

Supplement: S5 Fig — (A) Drought phenotype of srt1srt2 mutants. One of the representative repeats was imaged to show plants before and after rewatering. The survival plants out of 54 plants (6 independent replicates) were recorded. (B) Schematic diagram of the T-DNA insertion of HDT4. The filled boxes represent exon, and the open boxes represent intron. Primers used for (C) and (D) are also shown. (C—D) RT-PCR (C) and qRT-PCR (D) to show the expression of HDT4 in hdt4-1.n10-day-old seedlings of Col-0 and hdt4-1 were used for RNA extraction. Data in qRT-PCR are shown as mean ± SD. HDT4 gene expression hdt4-1 was compared to Col-0 with unpaired and two-tailed t-test. **** P < 0.0001. (E) RT-PCR to show the expression of HDT4 in response to dehydration. Plants were sampled as in S4A Fig. UBQ10 served as the loading control. (JPG) [file pgen.1010473.s005.jpg]

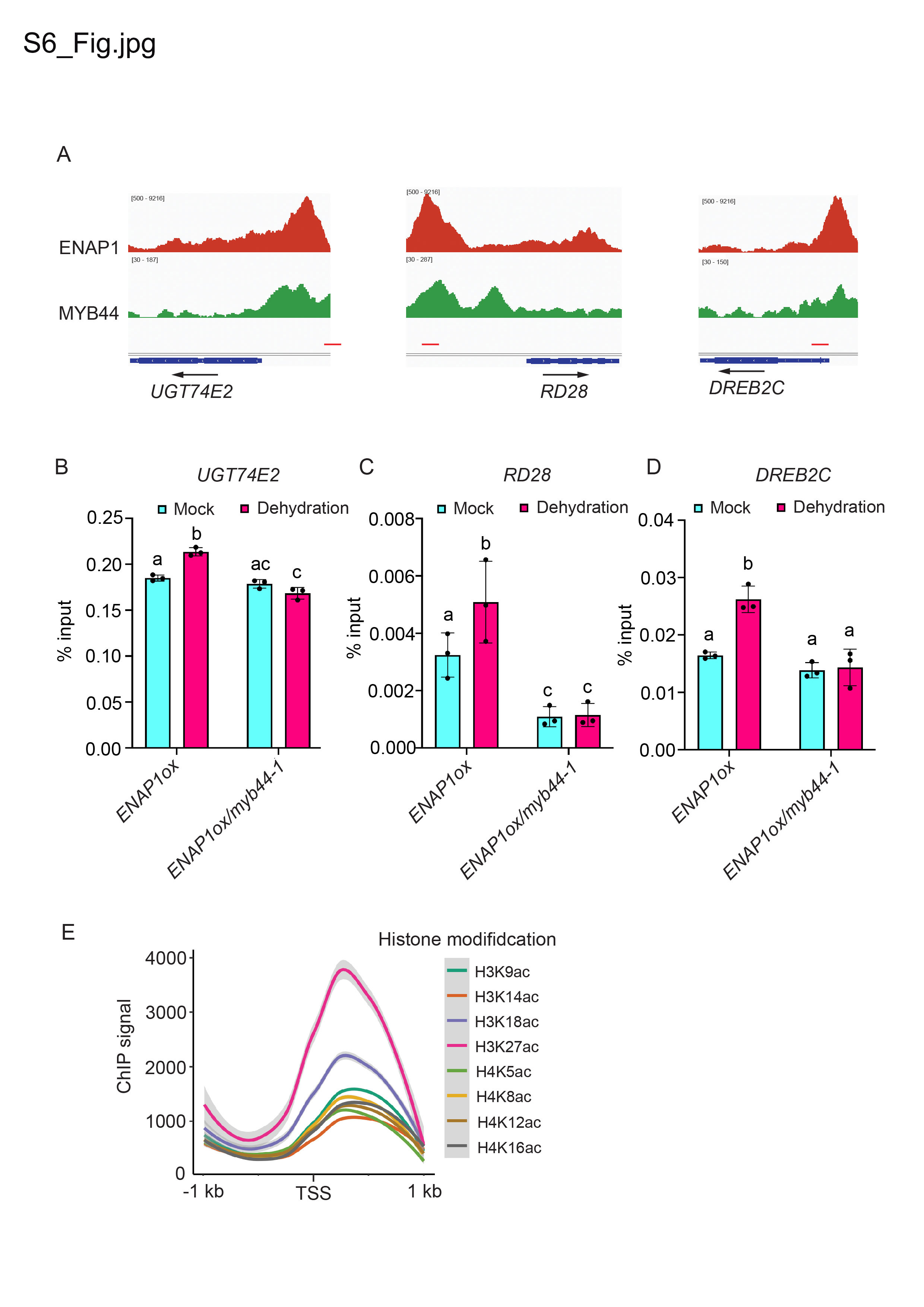

Supplement: S6 Fig — (A) The gene browser to show the binding of ENAP1 and MYB44. The red lines represent the location of primers used for ChIP-qPCR. (B—D) ChIP-qPCR to show ENAP1 enrichment on the target genes. Chromatin from 10-day-old seedlings of ENAP1ox and ENAP1ox/myb44-1 with mock or dehydration treatment was immunoprecipitated with anti-GFP antibody. Data represent mean ± SD of three replicates. Different letters represent significant differences with P < 0.05 in the one-way ANOVA test. (E) Enrichment of various histone acetylation marks on ENAP1 and ENAP2 specially regulated genes. The ChIP-seq signals were calculated with bamCoverage (deepTools 3.5.1) and were plotted with ggplot2 in R along 1 kb upstream to 1 kb downstream of TSS. (JPG) [file pgen.1010473.s006.jpg]
