# Supplementary material for: MYB44-ENAP1/2 restricts HDT4 to regulate drought tolerance in Arabidopsis
Source: PLoS Genet. 2022 Nov 22;18(11):e1010473. doi: 10.1371/journal.pgen.1010473 (PMC9681084; doi:10.1371/journal.pgen.1010473)
Supplement: S1 Table — (DOCX) [file pgen.1010473.s007.docx]

Table.S1. The mapping rates of each RNA-seq sample.

| Sample name | Average read length(bp) | Total reads | Uniquely mapped reads # | Uniquely mapped reads % |
| --- | --- | --- | --- | --- |
| Col-0 Mock BR1 | 284 | 19176303 | 18271748 | 95.28 |
| Col-0 Mock BR2 | 283 | 26569080 | 25333244 | 95.35 |
| *enap1-1enap2* Mock BR1 | 286 | 20230168 | 19408365 | 95.94 |
| *enap1-1enap2* Mock BR2 | 284 | 13362193 | 12878229 | 96.38 |
| Col-0 PEG BR1 | 284 | 13115960 | 12563915 | 95.79 |
| Col-0 PEG BR2 | 286 | 13693466 | 13057341 | 95.35 |
| *enap1-1enap2* PEG BR1 | 286 | 23601881 | 21661552 | 91.78 |
| *enap1-1enap2* PEG BR2 | 286 | 16151645 | 15465228 | 95.75 |
