# Supplementary material for: MYB44-ENAP1/2 restricts HDT4 to regulate drought tolerance in Arabidopsis
Source: PLoS Genet. 2022 Nov 22;18(11):e1010473. doi: 10.1371/journal.pgen.1010473 (PMC9681084; doi:10.1371/journal.pgen.1010473)
Supplement: S2 Table — (DOCX) [file pgen.1010473.s008.docx]

Table S2. Differentially regulated genes in Col-0 by dehydration.

| AGI | log2FoldChange | pvalue | padj |
| --- | --- | --- | --- |
| AT1G01010 | 1.205503649 | 1.5066E-14 | 1.7201E-13 |
| AT1G01030 | 2.107628506 | 3.828E-19 | 6.0345E-18 |
| AT1G01080 | -1.276143743 | 4.365E-21 | 7.7767E-20 |
| AT1G01120 | 1.539534532 | 1.5899E-44 | 7.1118E-43 |
| AT1G01190 | -3.776713242 | 1.2639E-35 | 4.2174E-34 |
| AT1G01200 | -2.641470066 | 3.3191E-09 | 2.3025E-08 |
| AT1G01240 | 2.890924434 | 1.0013E-68 | 8.7695E-67 |
| AT1G01300 | -1.343014624 | 3.3831E-29 | 8.8886E-28 |
| AT1G01360 | 1.114832604 | 1.7893E-08 | 1.1529E-07 |
| AT1G01370 | -1.36021709 | 4.3958E-05 | 0.00017482 |
| AT1G01390 | -1.285910258 | 0.00021502 | 0.00075104 |
| AT1G01430 | -1.179404654 | 2.3328E-14 | 2.619E-13 |
| AT1G01470 | 2.840924368 | 8.157E-110 | 1.434E-107 |
| AT1G01480 | 3.569416584 | 1.7258E-55 | 1.0548E-53 |
| AT1G01520 | 2.75557664 | 9.8809E-10 | 7.2928E-09 |
| AT1G01570 | 1.769250822 | 3.7622E-11 | 3.2146E-10 |
| AT1G01580 | 1.994927568 | 2.3657E-09 | 1.6656E-08 |
| AT1G01620 | 1.129822633 | 3.1136E-32 | 9.243E-31 |
| AT1G01650 | 1.373717938 | 3.4004E-32 | 1.0055E-30 |
| AT1G01720 | 2.624169452 | 3.227E-167 | 1.153E-164 |
| AT1G01725 | 1.415997413 | 0.00718569 | 0.01791759 |
| AT1G04047 | 2.467836833 | 0.00068583 | 0.0021638 |
| AT1G01750 | -2.224311976 | 0.00070895 | 0.00222968 |
| AT1G01830 | 1.062168981 | 1.0242E-11 | 9.1768E-11 |
| AT1G02080 | 1.017228063 | 1.8955E-13 | 1.9921E-12 |
| AT1G02190 | 1.885841497 | 0.001153 | 0.00346279 |
| AT1G02205 | 3.256181234 | 5.152E-216 | 3.02E-213 |
| AT1G02230 | 1.236061926 | 0.00065952 | 0.00208856 |
| AT1G02310 | 1.614818029 | 6.9884E-06 | 3.1644E-05 |
| AT1G02340 | 1.202106206 | 0.00015037 | 0.00054238 |
| AT1G02360 | -3.339553278 | 7.4891E-87 | 9.6172E-85 |
| AT1G02370 | -1.059113451 | 9.695E-07 | 4.94E-06 |
| AT1G02390 | 2.074503813 | 1.7414E-16 | 2.3062E-15 |
| AT1G02400 | 2.77982559 | 2.8031E-79 | 3.0657E-77 |
| AT1G02470 | 1.919883014 | 0.00032369 | 0.00109225 |
| AT1G02610 | 1.558649023 | 0.00054411 | 0.00175641 |
| AT1G02640 | -2.363968756 | 3.2414E-12 | 3.0516E-11 |
| AT1G02660 | 3.836138333 | 3.0091E-87 | 3.886E-85 |
| AT1G02670 | 1.345435128 | 0.00014546 | 0.00052554 |
| AT1G02700 | 1.459156051 | 3.9676E-05 | 0.00015913 |
| AT1G02730 | -1.032751746 | 5.5061E-15 | 6.538E-14 |
| AT1G02800 | -1.917108325 | 5.2236E-07 | 2.7595E-06 |
| AT1G02816 | 1.634679172 | 1.234E-27 | 2.9598E-26 |
| AT1G02820 | 3.438280803 | 4.0141E-63 | 3.0585E-61 |
| AT1G02860 | 1.081823295 | 4.2307E-07 | 2.2648E-06 |
| AT1G02900 | -1.967440636 | 3.9178E-23 | 7.8349E-22 |
| AT1G02920 | -1.809694052 | 2.6839E-50 | 1.4572E-48 |
| AT1G02980 | 2.831658517 | 0.00015931 | 0.00057149 |
| AT1G03055 | -1.444798715 | 0.00124602 | 0.00371918 |
| AT1G03070 | 2.788314922 | 1.4938E-21 | 2.7448E-20 |
| AT1G03080 | 1.588078687 | 5.9267E-57 | 3.7736E-55 |
| AT1G03090 | 1.418626102 | 1.4087E-21 | 2.5947E-20 |
| AT1G03190 | 1.21116569 | 4.2784E-13 | 4.3457E-12 |
| AT1G03290 | 1.474754757 | 2.8554E-29 | 7.5368E-28 |
| AT1G03440 | -1.105657309 | 2.4332E-07 | 1.3477E-06 |
| AT1G03520 | 1.294967151 | 2.9423E-09 | 2.0542E-08 |
| AT1G08825 | 2.049816466 | 3.5253E-10 | 2.7178E-09 |
| AT1G03610 | 1.074607343 | 1.3221E-10 | 1.0667E-09 |
| AT1G03700 | -2.404801555 | 1.5508E-05 | 6.6608E-05 |
| AT1G03750 | 1.191314093 | 9.8993E-07 | 5.0355E-06 |
| AT1G03770 | 1.027587365 | 2.1143E-06 | 1.0322E-05 |
| AT1G03840 | -1.10536492 | 0.0035879 | 0.00962924 |
| AT1G03870 | -3.454090052 | 7.3585E-12 | 6.6731E-11 |
| AT1G03920 | -1.088559517 | 0.00660991 | 0.01663246 |
| AT1G03990 | 1.306394792 | 0.00041029 | 0.00135527 |
| AT1G04040 | -1.108598615 | 5.2704E-08 | 3.193E-07 |
| AT1G04120 | 1.061203044 | 8.589E-25 | 1.8592E-23 |
| AT1G04180 | 2.550044626 | 1.0177E-18 | 1.5634E-17 |
| AT1G04220 | 2.779437251 | 3.6759E-93 | 5.1867E-91 |
| AT1G04247 | 4.047939196 | 6.6848E-57 | 4.2445E-55 |
| AT1G04300 | 1.192084831 | 2.0105E-22 | 3.8847E-21 |
| AT1G04310 | 1.982591512 | 4.5625E-30 | 1.2445E-28 |
| AT1G04520 | -1.973177075 | 6.431E-24 | 1.3474E-22 |
| AT1G04540 | -1.731061124 | 0.00748075 | 0.01855816 |
| AT1G04550 | -1.005850393 | 1.3417E-07 | 7.6919E-07 |
| AT1G04570 | 2.508357768 | 1.1876E-15 | 1.4834E-14 |
| AT1G04580 | 3.524551032 | 0.00026365 | 0.00090502 |
| AT1G04610 | -1.43604321 | 0.00131803 | 0.00391064 |
| AT1G04780 | 1.307429508 | 2.2914E-25 | 5.105E-24 |
| AT1G04830 | 1.753165423 | 7.1325E-33 | 2.1738E-31 |
| AT1G04980 | -1.022890964 | 1.0865E-10 | 8.8474E-10 |
| AT1G04990 | 1.27008435 | 2.8881E-23 | 5.8165E-22 |
| AT1G05000 | -1.614494354 | 2.215E-11 | 1.9325E-10 |
| AT1G05035 | -6.840343476 | 6.5488E-05 | 0.00025222 |
| AT1G05100 | 5.614862141 | 0 | 0 |
| AT1G05210 | -1.672570023 | 8.3532E-08 | 4.9299E-07 |
| AT1G05300 | -1.154815477 | 0.00098862 | 0.00302031 |
| AT1G05340 | 2.996196262 | 3.9633E-15 | 4.7706E-14 |
| AT1G05380 | 1.204429177 | 6.4968E-20 | 1.0699E-18 |
| AT1G05385 | -1.568769353 | 1.0161E-11 | 9.1188E-11 |
| AT1G05540 | -1.643327113 | 1.4524E-06 | 7.2437E-06 |
| AT1G05590 | -1.2166437 | 3.1916E-09 | 2.2195E-08 |
| AT1G05650 | -3.684321054 | 9.6866E-09 | 6.4086E-08 |
| AT1G05660 | -3.033079521 | 3.7054E-06 | 1.7474E-05 |
| AT1G05675 | 1.933241059 | 2.5286E-06 | 1.2183E-05 |
| AT1G05680 | 3.494586773 | 7.4E-199 | 4.027E-196 |
| AT1G05790 | 1.001245258 | 3.7848E-10 | 2.907E-09 |
| AT1G05880 | -9.338791461 | 7.3652E-10 | 5.518E-09 |
| AT1G05950 | -1.140677864 | 1.947E-05 | 8.2232E-05 |
| AT1G06120 | -4.453374485 | 0.00177237 | 0.00510172 |
| AT1G06430 | 1.447100704 | 2.5434E-29 | 6.7366E-28 |
| AT1G06540 | -1.75066602 | 0.0001142 | 0.00042192 |
| AT1G06550 | -1.203826205 | 1.0929E-27 | 2.6297E-26 |
| AT1G06570 | 2.381733792 | 4.521E-76 | 4.5524E-74 |
| AT1G06780 | 1.24382238 | 1.1813E-18 | 1.805E-17 |
| AT1G06790 | -1.389828265 | 2.1542E-07 | 1.203E-06 |
| AT1G06830 | -3.453326008 | 0.0004003 | 0.00132557 |
| AT1G06930 | -3.375415345 | 0.00245823 | 0.00687764 |
| AT1G06980 | -1.711098147 | 0.00099139 | 0.00302834 |
| AT1G07040 | 1.721874694 | 5.356E-35 | 1.7515E-33 |
| AT1G07050 | -1.89669042 | 8.048E-40 | 3.0866E-38 |
| AT1G07070 | -1.171359609 | 1.6231E-10 | 1.2977E-09 |
| AT1G07090 | -1.547155795 | 1.5187E-09 | 1.101E-08 |
| AT1G07150 | 2.437726203 | 7.6786E-38 | 2.7728E-36 |
| AT1G07175 | -6.054127248 | 0.00134812 | 0.00399007 |
| AT1G07270 | -2.027332183 | 0.00071202 | 0.0022381 |
| AT1G07370 | -1.594314298 | 9.802E-18 | 1.4003E-16 |
| AT1G07400 | 3.773290501 | 1.079E-101 | 1.7E-99 |
| AT1G07430 | 5.087189989 | 0 | 0 |
| AT1G07440 | -1.008678012 | 1.7449E-11 | 1.5352E-10 |
| AT1G07450 | -2.453768021 | 0.0039161 | 0.01041952 |
| AT1G07476 | 6.756976744 | 0.00061192 | 0.0019538 |
| AT1G07590 | 1.232402219 | 3.0611E-36 | 1.0459E-34 |
| AT1G07610 | 1.923830635 | 3.0799E-67 | 2.5693E-65 |
| AT1G07630 | 1.048156004 | 3.6854E-13 | 3.7642E-12 |
| AT1G07720 | 2.653453652 | 2.8267E-98 | 4.2508E-96 |
| AT1G07810 | 1.065638244 | 1.4623E-21 | 2.6913E-20 |
| AT1G07850 | 3.830314566 | 0.00525688 | 0.01357453 |
| AT1G07870 | 1.613746949 | 5.1738E-25 | 1.1317E-23 |
| AT1G07880 | -1.581994036 | 4.8292E-07 | 2.5623E-06 |
| AT1G07900 | 3.840425489 | 9.3121E-07 | 4.7566E-06 |
| AT1G07980 | 1.311483251 | 4.0196E-18 | 5.9354E-17 |
| AT1G07985 | 2.8474631 | 1.1831E-48 | 6.1183E-47 |
| AT1G08050 | 1.175235798 | 4.6047E-13 | 4.6614E-12 |
| AT1G08060 | 1.119036007 | 6.0684E-13 | 6.0758E-12 |
| AT1G08090 | -6.013547663 | 7.5263E-27 | 1.7681E-25 |
| AT1G08230 | 1.041738467 | 8.1139E-15 | 9.5258E-14 |
| AT1G08280 | -1.632387164 | 2.16E-12 | 2.065E-11 |
| AT1G08320 | -1.349703145 | 9.1945E-07 | 4.7003E-06 |
| AT1G08430 | -1.761941989 | 5.7653E-14 | 6.3176E-13 |
| AT1G08440 | 4.571451622 | 1.6001E-11 | 1.4105E-10 |
| AT1G08500 | 1.3339057 | 1.3753E-07 | 7.8672E-07 |
| AT1G08570 | 1.934385805 | 4.8684E-70 | 4.3469E-68 |
| AT1G08580 | -1.059819931 | 1.0348E-14 | 1.1977E-13 |
| AT1G08610 | -1.055231711 | 1.3463E-06 | 6.7462E-06 |
| AT1G08630 | 3.427645048 | 1.146E-46 | 5.5148E-45 |
| AT1G08650 | 1.698925654 | 4.1721E-24 | 8.8302E-23 |
| AT1G08670 | -3.767012868 | 0.00031033 | 0.00105073 |
| AT1G08800 | 1.22206647 | 8.3667E-15 | 9.8125E-14 |
| AT1G08810 | -1.647464261 | 7.1624E-09 | 4.7857E-08 |
| AT1G08830 | -1.117886866 | 8.5253E-21 | 1.4876E-19 |
| AT1G08890 | 1.88729123 | 1.0024E-50 | 5.5078E-49 |
| AT1G08920 | 1.334138877 | 5.4052E-23 | 1.0753E-21 |
| AT1G09070 | 1.56649583 | 6.546E-52 | 3.6764E-50 |
| AT1G09170 | -2.180957026 | 1.4782E-05 | 6.374E-05 |
| AT1G09180 | 1.732343024 | 0.0001412 | 0.00051147 |
| AT1G09200 | -1.186439606 | 5.2571E-21 | 9.2937E-20 |
| AT1G09390 | -1.600036965 | 4.6374E-05 | 0.00018371 |
| AT1G09460 | -1.225523059 | 0.00229489 | 0.00645859 |
| AT1G09490 | 1.786035694 | 4.0134E-45 | 1.8348E-43 |
| AT1G09500 | 5.35871019 | 1.0127E-16 | 1.3625E-15 |
| AT1G09520 | 1.142118641 | 2.4697E-13 | 2.5579E-12 |
| AT1G09530 | 2.685084621 | 3.772E-105 | 6.293E-103 |
| AT1G09750 | -2.123857002 | 5.4805E-76 | 5.4945E-74 |
| AT1G04723 | 7.858008851 | 4.2807E-06 | 1.9981E-05 |
| AT1G09810 | -1.108971527 | 3.4635E-06 | 1.6405E-05 |
| AT1G09950 | 4.26700625 | 1.512E-66 | 1.2256E-64 |
| AT1G09960 | 1.015697171 | 1.1594E-07 | 6.7145E-07 |
| AT1G10000 | -2.10465208 | 0.00371608 | 0.00994173 |
| AT1G10060 | 2.610429581 | 1.03E-104 | 1.693E-102 |
| AT1G10160 | 1.751862995 | 2.5174E-19 | 4.01E-18 |
| AT1G10170 | 3.017490245 | 7.823E-174 | 2.98E-171 |
| AT1G10280 | 1.560387972 | 9.5229E-13 | 9.3906E-12 |
| AT1G10330 | -1.302467309 | 0.0063169 | 0.01600263 |
| AT1G10340 | -1.96899102 | 0.0013003 | 0.00386354 |
| AT1G10370 | 1.004617363 | 1.5244E-19 | 2.4538E-18 |
| AT1G10380 | -1.207209762 | 1.6697E-07 | 9.4446E-07 |
| AT1G10450 | 1.072236833 | 2.0009E-16 | 2.6346E-15 |
| AT1G10460 | -3.024099161 | 7.0777E-05 | 0.00027104 |
| AT1G10470 | -1.259780105 | 3.693E-18 | 5.4744E-17 |
| AT1G10522 | -1.058551896 | 4.6663E-13 | 4.7175E-12 |
| AT1G10530 | 1.518921921 | 0.00162625 | 0.00472584 |
| AT1G10550 | -2.614834567 | 0.0081205 | 0.01996327 |
| AT1G10560 | 2.118633945 | 8.6081E-15 | 1.006E-13 |
| AT1G10580 | 1.201108493 | 2.3265E-23 | 4.7228E-22 |
| AT1G10585 | 4.240698103 | 4.6628E-17 | 6.3899E-16 |
| AT1G10640 | 1.184017289 | 3.8486E-18 | 5.6917E-17 |
| AT1G10657 | -1.039208094 | 0.00013677 | 0.00049688 |
| AT1G10740 | 1.640599867 | 1.2075E-30 | 3.3743E-29 |
| AT1G10750 | -1.109002388 | 0.0022946 | 0.00645856 |
| AT1G10770 | 1.880304805 | 0.00384127 | 0.01024571 |
| AT1G10780 | -1.046570554 | 8.8794E-05 | 0.00033459 |
| AT1G10960 | -1.119654755 | 1.2548E-19 | 2.0314E-18 |
| AT1G10990 | -3.029172257 | 3.1628E-10 | 2.4532E-09 |
| AT1G11080 | -1.449800877 | 1.0195E-10 | 8.3258E-10 |
| AT1G11100 | 1.075209228 | 3.4488E-05 | 0.0001396 |
| AT1G11130 | -1.071168247 | 3.7647E-11 | 3.2146E-10 |
| AT1G11160 | -1.265937064 | 0.00128525 | 0.0038233 |
| AT1G11170 | 2.149323536 | 7.002E-29 | 1.7983E-27 |
| AT1G11175 | 1.791316515 | 1.2338E-13 | 1.3191E-12 |
| AT1G11210 | 1.375720568 | 3.8861E-12 | 3.6272E-11 |
| AT1G11300 | -1.062309237 | 0.00032543 | 0.00109748 |
| AT1G11350 | -1.221635269 | 4.3647E-09 | 2.9888E-08 |
| AT1G11450 | -1.787464162 | 2.1185E-10 | 1.6756E-09 |
| AT1G11480 | 1.071657917 | 3.7702E-23 | 7.5529E-22 |
| AT1G11545 | -1.323287465 | 1.0209E-07 | 5.9532E-07 |
| AT1G11655 | -5.652792115 | 0.00491255 | 0.01278506 |
| AT1G11670 | -1.329459873 | 2.484E-15 | 3.0202E-14 |
| AT1G11700 | -1.443453975 | 2.0838E-10 | 1.6493E-09 |
| AT1G11740 | -2.56025095 | 0.00392195 | 0.01043387 |
| AT1G12030 | 2.075778856 | 0.00087999 | 0.00271823 |
| AT1G12040 | -3.850212181 | 4.5157E-15 | 5.4153E-14 |
| AT1G12064 | -6.343043874 | 0.00087904 | 0.00271567 |
| AT1G12110 | -1.020524819 | 5.7514E-21 | 1.0136E-19 |
| AT1G12420 | 1.54087969 | 2.5009E-19 | 3.9892E-18 |
| AT1G12480 | 1.152257781 | 0.00356302 | 0.00956921 |
| AT1G12500 | -1.621516997 | 5.1152E-24 | 1.0806E-22 |
| AT1G12560 | -5.024449612 | 5.9233E-28 | 1.4481E-26 |
| AT1G12740 | -2.197884875 | 1.4935E-14 | 1.7069E-13 |
| AT1G12900 | -1.255429415 | 2.9251E-39 | 1.0979E-37 |
| AT1G13100 | -1.599387778 | 1.3442E-07 | 7.7024E-07 |
| AT1G13110 | -1.956805074 | 3.7612E-44 | 1.6629E-42 |
| AT1G13250 | -1.342537718 | 1.6468E-13 | 1.7387E-12 |
| AT1G13370 | 1.999282941 | 0.0007729 | 0.0024112 |
| AT1G13420 | -1.61305154 | 1.2813E-06 | 6.4338E-06 |
| AT1G13480 | -2.448523219 | 1.7205E-06 | 8.4921E-06 |
| AT1G13500 | -2.624673434 | 0.00163005 | 0.00473474 |
| AT1G13510 | -5.582269127 | 2.7308E-11 | 2.36E-10 |
| AT1G13520 | -3.435665964 | 1.6383E-39 | 6.2207E-38 |
| AT1G13530 | -2.855122828 | 6.5595E-05 | 0.00025259 |
| AT1G13590 | -1.348909351 | 0.00545301 | 0.01402552 |
| AT1G13609 | 1.981222755 | 6.9435E-23 | 1.3682E-21 |
| AT1G13610 | -2.842920107 | 0.00959174 | 0.02313231 |
| AT1G13620 | -2.718770216 | 0.00312373 | 0.00850129 |
| AT1G13650 | -2.287622695 | 1.6531E-06 | 8.1861E-06 |
| AT1G13670 | -2.705880906 | 1.3485E-15 | 1.6816E-14 |
| AT1G13700 | 1.535212168 | 0.00181328 | 0.00520505 |
| AT1G13740 | 1.522364526 | 2.271E-15 | 2.7745E-14 |
| AT1G13830 | -2.629884871 | 1.2876E-08 | 8.4185E-08 |
| AT1G13960 | 1.161843187 | 4.5435E-17 | 6.2301E-16 |
| AT1G13970 | -1.055573112 | 0.00168309 | 0.00487172 |
| AT1G13990 | 2.607053529 | 5.5022E-72 | 5.1545E-70 |
| AT1G14080 | -2.238306542 | 2.5706E-09 | 1.8057E-08 |
| AT1G14120 | -1.704058956 | 5.7197E-23 | 1.1359E-21 |
| AT1G14160 | -3.377507093 | 1.1809E-06 | 5.9522E-06 |
| AT1G14180 | -1.547834843 | 1.3178E-08 | 8.611E-08 |
| AT1G14190 | -2.203577777 | 0.00017637 | 0.00062707 |
| AT1G14210 | -1.145202908 | 2.4465E-10 | 1.9184E-09 |
| AT1G14240 | -1.696586077 | 4.5188E-08 | 2.7672E-07 |
| AT1G14250 | -1.210546985 | 0.00125298 | 0.00373851 |
| AT1G14280 | -2.753771286 | 2.2653E-13 | 2.3591E-12 |
| AT1G14345 | -1.522482885 | 5.7273E-34 | 1.7983E-32 |
| AT1G14430 | -1.462304646 | 0.00011493 | 0.00042414 |
| AT1G14440 | -1.406023705 | 7.2363E-11 | 6.0192E-10 |
| AT1G14530 | 1.838865547 | 1.8539E-27 | 4.4051E-26 |
| AT1G14540 | -5.800065906 | 1.5542E-86 | 1.9736E-84 |
| AT1G14550 | -8.369244826 | 2.4409E-15 | 2.9709E-14 |
| AT1G14580 | -1.004450334 | 7.6518E-07 | 3.9607E-06 |
| AT1G14630 | -1.8709453 | 1.6842E-05 | 7.1835E-05 |
| AT1G14640 | 1.1913077 | 1.2399E-05 | 5.4106E-05 |
| AT1G14780 | -1.166130104 | 0.00047368 | 0.00154721 |
| AT1G14870 | -1.733775616 | 2.3129E-36 | 7.9382E-35 |
| AT1G14900 | -1.143295869 | 1.8812E-17 | 2.6445E-16 |
| AT1G14960 | -1.628499477 | 1.5568E-07 | 8.843E-07 |
| AT1G14980 | -1.038584283 | 7.6009E-18 | 1.0973E-16 |
| AT1G15010 | 3.652327728 | 2.7646E-28 | 6.8839E-27 |
| AT1G15085 | -3.164362872 | 0.0001708 | 0.00060954 |
| AT1G15125 | -2.197237306 | 1.9156E-11 | 1.6809E-10 |
| AT1G15150 | -1.872393585 | 0.00057154 | 0.00183563 |
| AT1G15230 | 1.018485348 | 1.7122E-13 | 1.8044E-12 |
| AT1G15250 | -1.327703608 | 2.6093E-11 | 2.261E-10 |
| AT1G15350 | 1.309159904 | 3.0673E-16 | 3.9904E-15 |
| AT1G15415 | 2.212044105 | 4.2688E-06 | 1.9934E-05 |
| AT1G15430 | 2.413786445 | 9.3419E-58 | 6.0837E-56 |
| AT1G15510 | -1.144775208 | 1.0784E-09 | 7.9185E-09 |
| AT1G15520 | 2.535763478 | 8.8104E-38 | 3.1765E-36 |
| AT1G15625 | -3.670384381 | 1.2842E-08 | 8.4058E-08 |
| AT1G15640 | -6.230981565 | 0.00071793 | 0.00225479 |
| AT1G15740 | 1.066448118 | 1.9244E-12 | 1.849E-11 |
| AT1G15760 | -1.909212407 | 9.5897E-06 | 4.2613E-05 |
| AT1G15800 | 1.8095313 | 3.2651E-20 | 5.4918E-19 |
| AT1G15870 | -1.152510551 | 0.00502917 | 0.01304695 |
| AT1G15960 | 1.084757337 | 5.3206E-07 | 2.8055E-06 |
| AT1G16030 | 3.452309069 | 7.112E-117 | 1.311E-114 |
| AT1G16070 | -1.555527437 | 6.5683E-05 | 0.00025289 |
| AT1G16120 | -2.376778824 | 0.00071172 | 0.00223745 |
| AT1G16225 | -5.566679995 | 0.00672523 | 0.01688733 |
| AT1G16250 | 1.095504272 | 3.3371E-08 | 2.0785E-07 |
| AT1G16300 | 1.301971804 | 2.4205E-18 | 3.6186E-17 |
| AT1G16350 | -1.005357858 | 9.1212E-13 | 9.0139E-12 |
| AT1G16370 | -1.564183267 | 0.00205167 | 0.00583225 |
| AT1G16390 | -5.806869399 | 5.8997E-17 | 8.0511E-16 |
| AT1G16400 | -3.641742794 | 1.6338E-48 | 8.3923E-47 |
| AT1G16410 | -3.002490992 | 9.488E-121 | 1.902E-118 |
| AT1G16440 | -3.309338504 | 0.00133325 | 0.00395116 |
| AT1G16500 | -1.188458647 | 1.3863E-05 | 6.006E-05 |
| AT1G16515 | 2.263571118 | 3.8475E-17 | 5.298E-16 |
| AT1G16540 | 1.189199914 | 3.5262E-12 | 3.3012E-11 |
| AT1G16630 | -1.037181263 | 1.5186E-05 | 6.5334E-05 |
| AT1G16840 | 1.486505461 | 2.8726E-31 | 8.2077E-30 |
| AT1G16850 | 3.69089445 | 3.318E-228 | 2.05E-225 |
| AT1G16860 | 1.08316254 | 3.2605E-20 | 5.4882E-19 |
| AT1G16880 | -1.007956596 | 2.64E-24 | 5.6031E-23 |
| AT1G17030 | -2.167665272 | 0.00442584 | 0.01164031 |
| AT1G17100 | -1.304707611 | 1.5737E-21 | 2.8847E-20 |
| AT1G17380 | 2.360081249 | 3.3522E-19 | 5.3101E-18 |
| AT1G17420 | 1.988352145 | 2.2316E-28 | 5.5871E-27 |
| AT1G17430 | -1.217079777 | 0.00062024 | 0.00197844 |
| AT1G17530 | 1.091241647 | 8.6149E-15 | 1.0062E-13 |
| AT1G17550 | 1.568063462 | 4.2396E-43 | 1.8046E-41 |
| AT1G17560 | -1.125315781 | 4.8481E-05 | 0.0001912 |
| AT1G17745 | 1.569104898 | 7.4762E-43 | 3.1414E-41 |
| AT1G17830 | 1.505845062 | 1.0104E-13 | 1.0894E-12 |
| AT1G17840 | 1.407367663 | 7.2242E-29 | 1.8512E-27 |
| AT1G17870 | 3.173401205 | 3.6497E-65 | 2.8867E-63 |
| AT1G17940 | 3.688730036 | 5.388E-167 | 1.895E-164 |
| AT1G18100 | 1.717557267 | 2.3589E-06 | 1.1419E-05 |
| AT1G18140 | -2.19608684 | 2.8294E-13 | 2.9159E-12 |
| AT1G18250 | -1.68664653 | 1.127E-16 | 1.5109E-15 |
| AT1G18360 | 1.366619172 | 2.6423E-21 | 4.7707E-20 |
| AT1G18460 | 1.665025684 | 1.6886E-25 | 3.7877E-24 |
| AT1G18590 | -2.664703902 | 7.2477E-54 | 4.2588E-52 |
| AT1G18650 | -1.473730734 | 7.4562E-12 | 6.7552E-11 |
| AT1G18810 | 1.233107011 | 5.4779E-09 | 3.7002E-08 |
| AT1G18830 | 4.391582856 | 2.3565E-23 | 4.7752E-22 |
| AT1G18860 | -2.186292108 | 4.9314E-05 | 0.00019402 |
| AT1G18870 | 1.912453447 | 1.014E-29 | 2.7236E-28 |
| AT1G18880 | -1.517878695 | 6.3845E-16 | 8.1212E-15 |
| AT1G18970 | -4.117784134 | 5.3811E-38 | 1.9524E-36 |
| AT1G19050 | -1.286401374 | 1.1358E-05 | 4.9938E-05 |
| AT1G19180 | 2.455526849 | 6.864E-100 | 1.046E-97 |
| AT1G19190 | -1.731927939 | 5.3584E-05 | 0.00020966 |
| AT1G19200 | 2.36872355 | 2.8425E-17 | 3.945E-16 |
| AT1G19210 | 3.574910719 | 0.00040847 | 0.00134983 |
| AT1G19310 | 1.086932777 | 1.4237E-10 | 1.145E-09 |
| AT1G19340 | -1.268954709 | 0.00460714 | 0.01206163 |
| AT1G19396 | 1.890763952 | 0.00060104 | 0.00192185 |
| AT1G19397 | 1.157717284 | 0.00523195 | 0.01352084 |
| AT1G19400 | 1.232677688 | 2.2599E-20 | 3.8264E-19 |
| AT1G19490 | 1.846436073 | 3.525E-13 | 3.6083E-12 |
| AT1G19565 | 3.083944092 | 1.1338E-07 | 6.5711E-07 |
| AT1G19640 | 2.137533165 | 2.2057E-09 | 1.561E-08 |
| AT1G19660 | 1.225257996 | 9.9294E-33 | 3.0102E-31 |
| AT1G19670 | -1.104397223 | 1.0991E-20 | 1.9046E-19 |
| AT1G19830 | -3.889840923 | 0.00840205 | 0.02057135 |
| AT1G19840 | 1.267266133 | 0.00021701 | 0.00075709 |
| AT1G19960 | -1.61951059 | 0.00026176 | 0.00089976 |
| AT1G19970 | 2.384290414 | 1.2783E-55 | 7.8336E-54 |
| AT1G20010 | -1.235165804 | 2.4384E-31 | 7.0109E-30 |
| AT1G20030 | 1.747307326 | 5.4765E-32 | 1.609E-30 |
| AT1G20100 | 1.10855877 | 7.9088E-23 | 1.5558E-21 |
| AT1G20140 | 1.93162861 | 2.5369E-43 | 1.0879E-41 |
| AT1G20310 | 2.904037774 | 1.4068E-06 | 7.0319E-06 |
| AT1G20390 | 1.038111433 | 5.8303E-09 | 3.9283E-08 |
| AT1G20440 | 2.993882874 | 2.152E-181 | 8.785E-179 |
| AT1G20450 | 3.575382969 | 9.536E-183 | 4.037E-180 |
| AT1G20460 | 1.150018009 | 7.4459E-06 | 3.3609E-05 |
| AT1G20670 | 1.145026634 | 8.794E-15 | 1.0256E-13 |
| AT1G20720 | -1.274133713 | 1.3868E-05 | 6.0072E-05 |
| AT1G20780 | 1.190952565 | 1.287E-12 | 1.2556E-11 |
| AT1G20840 | -1.049313843 | 1.1873E-13 | 1.27E-12 |
| AT1G20880 | 1.537951094 | 4.1097E-16 | 5.3073E-15 |
| AT1G20970 | 1.167910725 | 2.1889E-21 | 3.971E-20 |
| AT1G21000 | 2.387827038 | 2.0727E-72 | 1.9823E-70 |
| AT1G21010 | 1.08701369 | 7.5785E-08 | 4.503E-07 |
| AT1G21050 | -1.569469805 | 3.1467E-07 | 1.7134E-06 |
| AT1G21100 | -1.285820081 | 2.8559E-13 | 2.9419E-12 |
| AT1G21110 | -1.460016724 | 1.0226E-15 | 1.2837E-14 |
| AT1G21120 | -1.323041902 | 3.8542E-10 | 2.9583E-09 |
| AT1G21140 | -1.389132025 | 0.00174197 | 0.00502624 |
| AT1G21230 | -6.963763336 | 3.9136E-05 | 0.00015711 |
| AT1G21240 | -3.284737022 | 4.818E-05 | 0.00019011 |
| AT1G21270 | -3.02909317 | 2.2863E-18 | 3.4247E-17 |
| AT1G21310 | -1.190804107 | 6.1208E-17 | 8.3478E-16 |
| AT1G21400 | 3.115637995 | 2.11E-116 | 3.828E-114 |
| AT1G21410 | 2.497289222 | 1.1088E-66 | 9.0519E-65 |
| AT1G21550 | 1.177723365 | 3.572E-06 | 1.688E-05 |
| AT1G21670 | 1.094584338 | 3.0369E-14 | 3.378E-13 |
| AT1G21790 | 3.512775756 | 1.603E-143 | 4.212E-141 |
| AT1G21980 | 1.916363027 | 1.3213E-52 | 7.5132E-51 |
| AT1G22030 | -1.155552856 | 0.00016793 | 0.00060013 |
| AT1G22160 | 1.530428158 | 3.7612E-13 | 3.8398E-12 |
| AT1G22190 | 1.308863149 | 6.4888E-22 | 1.2217E-20 |
| AT1G22280 | 1.090436078 | 1.9395E-17 | 2.7215E-16 |
| AT1G22330 | -2.543415162 | 1.1015E-08 | 7.2432E-08 |
| AT1G22370 | 1.588608223 | 4.8876E-28 | 1.1974E-26 |
| AT1G22400 | 2.65339662 | 7.885E-124 | 1.638E-121 |
| AT1G22440 | -1.044190934 | 3.4497E-13 | 3.5345E-12 |
| AT1G22470 | 3.45676812 | 5.4097E-68 | 4.6487E-66 |
| AT1G22490 | 1.374749922 | 0.00224213 | 0.0063249 |
| AT1G22550 | -2.060415406 | 2.7658E-20 | 4.6692E-19 |
| AT1G22630 | -1.719715586 | 7.2283E-22 | 1.3543E-20 |
| AT1G22640 | 1.339993871 | 6.7176E-19 | 1.0446E-17 |
| AT1G22690 | -1.341641503 | 6.3948E-12 | 5.8422E-11 |
| AT1G22710 | 1.508791897 | 3.4199E-42 | 1.4136E-40 |
| AT1G22930 | 1.722014313 | 4.1078E-53 | 2.3651E-51 |
| AT1G22985 | 2.629369423 | 1.3181E-33 | 4.116E-32 |
| AT1G22990 | 2.464065977 | 3.9462E-05 | 0.00015833 |
| AT1G23030 | -1.087769552 | 6.2308E-09 | 4.1852E-08 |
| AT1G23040 | 1.114667973 | 7.2254E-12 | 6.5591E-11 |
| AT1G23080 | -1.063838626 | 7.3215E-18 | 1.0585E-16 |
| AT1G23120 | -1.52562763 | 0.00015448 | 0.00055581 |
| AT1G23190 | 1.030362108 | 2.1355E-24 | 4.5535E-23 |
| AT1G23200 | 2.258016753 | 1.6907E-19 | 2.7158E-18 |
| AT1G23205 | -1.396238267 | 6.4288E-14 | 7.0109E-13 |
| AT1G23340 | -1.803128782 | 2.5308E-07 | 1.3973E-06 |
| AT1G23440 | 1.040955417 | 1.012E-20 | 1.7577E-19 |
| AT1G23450 | 2.315053586 | 8.2627E-09 | 5.4984E-08 |
| AT1G23480 | -1.952347908 | 3.2737E-14 | 3.6361E-13 |
| AT1G23560 | -1.735454955 | 5.4344E-05 | 0.00021245 |
| AT1G23710 | 1.361209297 | 3.4152E-15 | 4.1216E-14 |
| AT1G23720 | -2.644489076 | 2.9019E-06 | 1.3909E-05 |
| AT1G23760 | -1.822297323 | 2.6284E-05 | 0.00010874 |
| AT1G23790 | -1.377473992 | 2.2937E-05 | 9.5778E-05 |
| AT1G23800 | 1.039152962 | 3.3143E-10 | 2.562E-09 |
| AT1G23850 | -1.528521452 | 0.00247362 | 0.00691224 |
| AT1G23870 | 1.114644719 | 9.8313E-15 | 1.1402E-13 |
| AT1G23880 | 1.089808543 | 3.6429E-05 | 0.00014689 |
| AT1G24020 | -1.778266504 | 4.9552E-55 | 2.9886E-53 |
| AT1G24070 | 1.212842443 | 4.222E-15 | 5.0686E-14 |
| AT1G24100 | -1.168454455 | 2.8803E-22 | 5.514E-21 |
| AT1G24130 | -1.228238479 | 0.00613142 | 0.01558282 |
| AT1G24148 | -1.565773452 | 3.3002E-05 | 0.00013413 |
| AT1G24150 | -1.09125286 | 2.306E-06 | 1.1179E-05 |
| AT1G24470 | 2.808218375 | 6.6089E-21 | 1.1576E-19 |
| AT1G24580 | 4.362188333 | 1.1763E-89 | 1.5542E-87 |
| AT1G24600 | 3.773267733 | 1.5357E-57 | 9.9158E-56 |
| AT1G25230 | -1.111472933 | 5.9041E-05 | 0.00022944 |
| AT1G25370 | 1.701933062 | 4.4659E-18 | 6.5774E-17 |
| AT1G25422 | 1.278334711 | 0.00018717 | 0.00066238 |
| AT1G25440 | -1.287685349 | 3.9458E-28 | 9.7402E-27 |
| AT1G25450 | 2.013317781 | 2.5803E-58 | 1.7096E-56 |
| AT1G25520 | 1.521159048 | 6.6452E-28 | 1.6176E-26 |
| AT1G25560 | 1.24935346 | 5.8854E-22 | 1.1115E-20 |
| AT1G26100 | -1.267740366 | 0.00070378 | 0.00221505 |
| AT1G26240 | -7.320302715 | 9.0247E-41 | 3.5323E-39 |
| AT1G26380 | -4.140770998 | 4.5782E-16 | 5.899E-15 |
| AT1G26390 | -6.453340396 | 2.514E-15 | 3.055E-14 |
| AT1G26410 | -6.650337011 | 2.2456E-17 | 3.1376E-16 |
| AT1G26420 | -2.645985304 | 1.6789E-15 | 2.0789E-14 |
| AT1G26450 | 1.132830109 | 7.5077E-15 | 8.846E-14 |
| AT1G26540 | -1.425377335 | 0.00510708 | 0.01323256 |
| AT1G26620 | 1.814080652 | 2.8111E-32 | 8.3558E-31 |
| AT1G26670 | 1.25511412 | 5.0594E-25 | 1.1077E-23 |
| AT1G26730 | 1.007233896 | 0.00143452 | 0.00422118 |
| AT1G26761 | -1.095620057 | 3.0371E-06 | 1.4517E-05 |
| AT1G26770 | 1.97949207 | 2.639E-54 | 1.5627E-52 |
| AT1G26800 | 1.001486898 | 0.00039204 | 0.001301 |
| AT1G26870 | -6.297374556 | 0.00057998 | 0.00186041 |
| AT1G26920 | 1.151129259 | 6.0961E-16 | 7.7802E-15 |
| AT1G26945 | -2.475622284 | 2.3138E-07 | 1.2877E-06 |
| AT1G26960 | -1.106513369 | 0.00198181 | 0.00564842 |
| AT1G26970 | -1.785142351 | 0.00273002 | 0.00754499 |
| AT1G27020 | -1.292529594 | 1.5199E-14 | 1.7328E-13 |
| AT1G27100 | -1.02441274 | 2.4203E-11 | 2.1044E-10 |
| AT1G27200 | 2.004390477 | 1.1932E-50 | 6.5395E-49 |
| AT1G27290 | 1.003450062 | 1.118E-13 | 1.1986E-12 |
| AT1G27730 | 2.740455245 | 3.1495E-75 | 3.1301E-73 |
| AT1G27740 | -8.330249063 | 7.2538E-08 | 4.3201E-07 |
| AT1G27760 | 1.089935297 | 2.7258E-25 | 6.0317E-24 |
| AT1G27910 | 1.071214183 | 7.9366E-12 | 7.1791E-11 |
| AT1G27950 | 1.016115352 | 3.4795E-16 | 4.5113E-15 |
| AT1G28010 | 1.536737294 | 3.1048E-17 | 4.296E-16 |
| AT1G28110 | -1.094671455 | 5.9361E-08 | 3.5736E-07 |
| AT1G28130 | -1.057172457 | 1.4764E-07 | 8.4202E-07 |
| AT1G28200 | 1.36207048 | 6.2046E-28 | 1.5136E-26 |
| AT1G28260 | 1.350549519 | 7.7186E-16 | 9.7584E-15 |
| AT1G28290 | -1.093796933 | 6.2002E-21 | 1.0877E-19 |
| AT1G28330 | 1.819396228 | 9.3417E-10 | 6.9105E-09 |
| AT1G28370 | 2.606942127 | 1.284E-10 | 1.0375E-09 |
| AT1G28400 | -2.355776541 | 6.2738E-80 | 7.0298E-78 |
| AT1G28470 | -1.599059716 | 1.1616E-05 | 5.0991E-05 |
| AT1G06163 | 8.234602739 | 5.9982E-07 | 3.1475E-06 |
| AT1G28520 | 1.609584684 | 1.7849E-35 | 5.9214E-34 |
| AT1G28570 | -1.285410643 | 2.0471E-07 | 1.1466E-06 |
| AT1G28660 | -2.359494726 | 1.1121E-19 | 1.808E-18 |
| AT1G28670 | -2.37448304 | 7.1195E-37 | 2.4998E-35 |
| AT1G28680 | -1.266401542 | 1.429E-05 | 6.1757E-05 |
| AT1G28710 | -1.062795408 | 5.2496E-07 | 2.7706E-06 |
| AT1G28980 | 6.298937233 | 0.00353596 | 0.00950546 |
| AT1G28960 | 1.669842126 | 9.9245E-36 | 3.3263E-34 |
| AT1G29025 | -1.433024919 | 0.0002486 | 0.0008571 |
| AT1G29050 | 1.053944271 | 9.5758E-10 | 7.0767E-09 |
| AT1G29195 | 2.342277534 | 1.9167E-24 | 4.0983E-23 |
| AT1G29230 | 1.732179842 | 0.00072325 | 0.00226932 |
| AT1G29240 | 1.245746145 | 9.6117E-09 | 6.3646E-08 |
| AT1G29280 | -1.464428551 | 2.1216E-14 | 2.3889E-13 |
| AT1G29330 | 1.739466484 | 3.9905E-37 | 1.412E-35 |
| AT1G29340 | 1.032783147 | 6.3502E-17 | 8.6452E-16 |
| AT1G29357 | 1.435534736 | 4.3992E-05 | 0.00017491 |
| AT1G29395 | 1.556560064 | 1.3171E-46 | 6.2852E-45 |
| AT1G29440 | -5.6572109 | 0.00486597 | 0.01268114 |
| AT1G29450 | -3.98457134 | 0.00586136 | 0.01496136 |
| AT1G29460 | -6.43520189 | 0.00039424 | 0.00130717 |
| AT1G29500 | 1.353256533 | 0.00191185 | 0.00546332 |
| AT1G29530 | -1.142287402 | 2.546E-10 | 1.9928E-09 |
| AT1G29600 | -1.802544567 | 0.00039125 | 0.00129875 |
| AT1G29640 | 2.227993545 | 1.4938E-30 | 4.1438E-29 |
| AT1G29660 | -3.398910075 | 6.3291E-75 | 6.2628E-73 |
| AT1G29730 | -1.466010069 | 0.0028464 | 0.00783326 |
| AT1G29980 | -1.025012637 | 1.7927E-10 | 1.4263E-09 |
| AT1G30080 | -1.470254574 | 2.2855E-05 | 9.5471E-05 |
| AT1G30190 | 2.25797105 | 0.0033323 | 0.0090131 |
| AT1G30220 | 2.375279745 | 1.474E-08 | 9.5853E-08 |
| AT1G30250 | 2.834278125 | 3.6329E-31 | 1.0341E-29 |
| AT1G30260 | 1.540721736 | 3.885E-19 | 6.1159E-18 |
| AT1G30320 | 1.418166065 | 7.6139E-28 | 1.8475E-26 |
| AT1G30370 | -3.247198126 | 3.6495E-07 | 1.9707E-06 |
| AT1G30410 | 1.057215929 | 1.4644E-19 | 2.3622E-18 |
| AT1G30420 | -1.394950311 | 1.1158E-11 | 9.963E-11 |
| AT1G30500 | 1.088310794 | 3.4171E-07 | 1.8535E-06 |
| AT1G30520 | -1.054139509 | 1.6319E-09 | 1.1775E-08 |
| AT1G30530 | -2.538191605 | 3.0793E-80 | 3.4845E-78 |
| AT1G30550 | -1.800411399 | 0.00065893 | 0.00208729 |
| AT1G30620 | 1.557590292 | 1.1796E-30 | 3.3004E-29 |
| AT1G30640 | 1.727676779 | 1.8547E-20 | 3.159E-19 |
| AT1G30650 | -1.013160937 | 0.00070422 | 0.00221601 |
| AT1G30720 | -1.228123261 | 7.9102E-06 | 3.5558E-05 |
| AT1G30750 | -2.089465578 | 1.7799E-09 | 1.2749E-08 |
| AT1G30840 | -5.028526677 | 7.9437E-26 | 1.7942E-24 |
| AT1G30850 | -4.195489543 | 0.00301908 | 0.00825183 |
| AT1G30860 | 1.342637295 | 3.6001E-06 | 1.6999E-05 |
| AT1G30870 | -4.438207676 | 3.4312E-39 | 1.2836E-37 |
| AT1G30900 | -1.338531372 | 1.1385E-06 | 5.7535E-06 |
| AT1G30990 | -3.884354262 | 1.0939E-05 | 4.8144E-05 |
| AT1G31050 | -1.574462273 | 7.872E-08 | 4.6592E-07 |
| AT1G31290 | 2.05953012 | 0.00039527 | 0.00131018 |
| AT1G31320 | -1.321945892 | 4.7546E-05 | 0.0001879 |
| AT1G31480 | 1.120652216 | 6.8189E-09 | 4.5615E-08 |
| AT1G31550 | -1.709142828 | 4.4401E-16 | 5.7276E-15 |
| AT1G31580 | -2.099928719 | 1.8816E-59 | 1.2762E-57 |
| AT1G31690 | -3.836171153 | 1.8129E-90 | 2.4666E-88 |
| AT1G31750 | 2.085599898 | 3.6998E-08 | 2.2919E-07 |
| AT1G31770 | -1.695374329 | 5.1842E-20 | 8.6182E-19 |
| AT1G31820 | 1.384942682 | 8.2003E-07 | 4.2283E-06 |
| AT1G31880 | 1.685880602 | 5.2554E-11 | 4.4426E-10 |
| AT1G31885 | -1.826253953 | 0.00099366 | 0.00303446 |
| AT1G31935 | -1.983621933 | 5.0416E-08 | 3.0625E-07 |
| AT1G06473 | 2.460780156 | 9.2037E-10 | 6.8171E-09 |
| AT1G32340 | 1.00837486 | 1.1256E-10 | 9.1464E-10 |
| AT1G32350 | -1.397431855 | 0.00298481 | 0.00816891 |
| AT1G32450 | 1.506221587 | 2.1552E-36 | 7.4193E-35 |
| AT1G32750 | 1.41722967 | 2.7802E-32 | 8.2748E-31 |
| AT1G32780 | -1.256259897 | 0.00714083 | 0.01782322 |
| AT1G32860 | 1.73946401 | 3.1569E-29 | 8.3134E-28 |
| AT1G32870 | 1.905967266 | 1.5467E-43 | 6.7085E-42 |
| AT1G32880 | 8.222506808 | 6.8982E-07 | 3.5877E-06 |
| AT1G32920 | 1.116020835 | 2.374E-12 | 2.2601E-11 |
| AT1G32928 | 1.157243783 | 0.00554284 | 0.01424215 |
| AT1G33030 | -2.310041336 | 0.00130178 | 0.00386745 |
| AT1G33110 | 1.543527009 | 3.6948E-36 | 1.2512E-34 |
| AT1G33170 | -1.657094729 | 1.3169E-19 | 2.1289E-18 |
| AT1G33240 | -1.057696395 | 2.1029E-09 | 1.4933E-08 |
| AT1G33260 | 1.364707595 | 2.5466E-10 | 1.9928E-09 |
| AT1G33340 | -2.051777598 | 0.00086106 | 0.00266543 |
| AT1G33440 | -2.581086095 | 1.4121E-05 | 6.1088E-05 |
| AT1G33480 | 1.567759103 | 2.7166E-19 | 4.3212E-18 |
| AT1G33700 | 1.199169547 | 1.4433E-07 | 8.2415E-07 |
| AT1G33750 | -1.595922115 | 4.5577E-09 | 3.1108E-08 |
| AT1G33800 | -1.295337749 | 1.9701E-09 | 1.4046E-08 |
| AT1G33811 | -2.903560135 | 9.9659E-56 | 6.1237E-54 |
| AT1G33930 | -1.402642274 | 0.00660222 | 0.01661741 |
| AT1G34010 | -1.074356389 | 1.2935E-06 | 6.4909E-06 |
| AT1G34040 | -2.121112536 | 3.6331E-08 | 2.2524E-07 |
| AT1G34065 | -1.129717379 | 6.0787E-05 | 0.00023546 |
| AT1G34220 | 1.150785692 | 6.4184E-14 | 7.0029E-13 |
| AT1G34245 | -1.373045979 | 8.8869E-05 | 0.00033482 |
| AT1G34260 | 1.64747368 | 1.6032E-23 | 3.2984E-22 |
| AT1G34315 | -1.160876032 | 0.00020929 | 0.00073316 |
| AT1G34510 | -6.522507233 | 0.00036721 | 0.00122501 |
| AT1G34570 | -1.010451214 | 1.6396E-07 | 9.2862E-07 |
| AT1G34630 | 1.825788973 | 4.2974E-24 | 9.0869E-23 |
| AT1G34750 | -1.246830746 | 2.7712E-07 | 1.5209E-06 |
| AT1G35140 | -2.769751192 | 0.00185985 | 0.00532805 |
| AT1G35250 | -2.686689344 | 4.9611E-20 | 8.2593E-19 |
| AT1G35260 | -2.37642065 | 7.9527E-20 | 1.304E-18 |
| AT1G35290 | -2.158789385 | 4.3184E-05 | 0.00017197 |
| AT1G35310 | -3.114894827 | 2.4036E-06 | 1.1618E-05 |
| AT1G35330 | -3.310419533 | 0.00098854 | 0.00302031 |
| AT1G06877 | 5.716105587 | 0.0013555 | 0.00400984 |
| AT1G35560 | -2.50889022 | 1.3456E-14 | 1.5433E-13 |
| AT1G36060 | -1.181353426 | 0.00038277 | 0.00127281 |
| AT1G36370 | 1.270946674 | 2.0681E-23 | 4.2057E-22 |
| AT1G36622 | -1.239462882 | 0.00041393 | 0.00136551 |
| AT1G06983 | -3.060027869 | 0.00028899 | 0.00098418 |
| AT1G36640 | -4.423170256 | 9.6839E-11 | 7.931E-10 |
| AT1G36940 | -2.102673982 | 0.00134812 | 0.00399007 |
| AT1G37130 | 1.194536092 | 5.2206E-21 | 9.2363E-20 |
| AT1G38131 | -1.680141663 | 0.00054584 | 0.00176151 |
| AT1G42470 | 1.11196193 | 7.6384E-14 | 8.2866E-13 |
| AT1G09475 | -8.076873791 | 2.3709E-07 | 1.3157E-06 |
| AT1G42980 | 1.877777483 | 0.00322853 | 0.00876249 |
| AT1G42990 | 1.104969753 | 6.2626E-20 | 1.0336E-18 |
| AT1G43160 | 5.408941086 | 0 | 0 |
| AT1G43800 | -1.713585573 | 0.00960785 | 0.02316627 |
| AT1G43890 | 1.040512024 | 5.7729E-21 | 1.0158E-19 |
| AT1G43910 | 1.665669296 | 9.2609E-20 | 1.512E-18 |
| AT1G44000 | -1.018595331 | 2.9486E-12 | 2.7874E-11 |
| AT1G44110 | -1.308407214 | 7.5402E-09 | 5.0308E-08 |
| AT1G44160 | -1.612566568 | 0.00307228 | 0.0083762 |
| AT1G44350 | 2.446435194 | 1.6022E-71 | 1.4767E-69 |
| AT1G44740 | -1.956789042 | 3.1087E-06 | 1.4822E-05 |
| AT1G44920 | -1.017491027 | 5.6684E-19 | 8.8503E-18 |
| AT1G44970 | -4.121112924 | 5.9745E-15 | 7.0906E-14 |
| AT1G45120 | 1.217281817 | 4.2152E-06 | 1.9704E-05 |
| AT1G45249 | 2.317553335 | 5.2044E-93 | 7.2983E-91 |
| AT1G46480 | -1.394030007 | 1.0439E-06 | 5.298E-06 |
| AT1G46554 | 1.985827745 | 3.7432E-24 | 7.9371E-23 |
| AT1G46768 | 2.293784961 | 1.1284E-23 | 2.3364E-22 |
| AT1G47128 | 1.114788673 | 1.049E-32 | 3.176E-31 |
| AT1G47270 | 1.181037734 | 2.589E-15 | 3.1411E-14 |
| AT1G47400 | 1.573900018 | 4.7518E-08 | 2.898E-07 |
| AT1G07367 | 2.467854013 | 0.00011743 | 0.00043245 |
| AT1G47480 | -1.959056986 | 7.4036E-16 | 9.3706E-15 |
| AT1G47510 | 3.666937108 | 1.3386E-47 | 6.6519E-46 |
| AT1G47580 | -1.067415983 | 2.3882E-07 | 1.3237E-06 |
| AT1G47890 | -3.980411794 | 6.3789E-08 | 3.819E-07 |
| AT1G47960 | 1.984406761 | 2.6409E-33 | 8.1576E-32 |
| AT1G48090 | 1.438064985 | 1.8754E-23 | 3.8308E-22 |
| AT1G48100 | 3.147858148 | 4.704E-67 | 3.8958E-65 |
| AT1G48300 | 1.017564139 | 8.9587E-23 | 1.7593E-21 |
| AT1G48370 | 1.696697183 | 4.8859E-28 | 1.1974E-26 |
| AT1G48405 | 4.582969066 | 7.0115E-08 | 4.1812E-07 |
| AT1G48460 | -1.333088908 | 7.0012E-12 | 6.3632E-11 |
| AT1G48480 | -1.373377686 | 1.5084E-21 | 2.7693E-20 |
| AT1G48510 | -1.787381814 | 0.0009747 | 0.00298497 |
| AT1G48570 | -1.139653688 | 4.3102E-10 | 3.295E-09 |
| AT1G48610 | -1.28494068 | 2.1508E-15 | 2.6361E-14 |
| AT1G48635 | 1.118239589 | 3.1479E-17 | 4.3529E-16 |
| AT1G48670 | -2.716390299 | 0.00929929 | 0.02250298 |
| AT1G48690 | -2.984251352 | 0.00252655 | 0.00704463 |
| AT1G48840 | 1.202381007 | 6.6339E-15 | 7.8366E-14 |
| AT1G48930 | -3.065187798 | 9.7287E-15 | 1.1288E-13 |
| AT1G49010 | -1.205822549 | 1.9584E-11 | 1.7151E-10 |
| AT1G49032 | 1.110171801 | 1.3644E-06 | 6.829E-06 |
| AT1G49230 | -3.485405908 | 7.8949E-13 | 7.8359E-12 |
| AT1G49310 | -2.309255249 | 8.1472E-14 | 8.8218E-13 |
| AT1G49320 | -2.396033727 | 9.5379E-08 | 5.5887E-07 |
| AT1G49390 | -1.973848351 | 0.00160057 | 0.00465707 |
| AT1G49450 | 3.932543258 | 5.653E-205 | 3.23E-202 |
| AT1G49500 | 1.634389751 | 3.0643E-38 | 1.1189E-36 |
| AT1G49510 | -1.367140721 | 1.6814E-16 | 2.2293E-15 |
| AT1G49570 | -4.387448282 | 0.00130985 | 0.00388789 |
| AT1G49700 | 1.116146645 | 0.00068959 | 0.00217477 |
| AT1G49750 | -2.371960181 | 5.6189E-78 | 5.9187E-76 |
| AT1G49860 | 1.94848003 | 8.612E-11 | 7.0912E-10 |
| AT1G49900 | 3.162447192 | 6.9723E-05 | 0.0002674 |
| AT1G49975 | -1.346112637 | 5.3437E-13 | 5.388E-12 |
| AT1G50055 | -1.466270509 | 1.0242E-05 | 4.5258E-05 |
| AT1G50060 | -2.933068356 | 1.3461E-09 | 9.8023E-09 |
| AT1G50110 | -1.48646169 | 2.6634E-08 | 1.6762E-07 |
| AT1G50260 | 2.749435013 | 1.737E-130 | 3.856E-128 |
| AT1G50732 | -1.212357385 | 2.2776E-07 | 1.2685E-06 |
| AT1G50900 | -1.138820543 | 2.5915E-10 | 2.0273E-09 |
| AT1G50930 | 2.419083148 | 9.7568E-05 | 0.00036507 |
| AT1G50960 | 3.638373052 | 0.00069522 | 0.00219071 |
| AT1G51060 | -1.008711182 | 4.7362E-21 | 8.4249E-20 |
| AT1G51080 | -1.602056859 | 2.3361E-10 | 1.8343E-09 |
| AT1G51090 | 1.747868342 | 2.2839E-39 | 8.6148E-38 |
| AT1G51140 | 1.653614352 | 2.3526E-54 | 1.4004E-52 |
| AT1G51150 | 7.095372658 | 0.00020246 | 0.00071165 |
| AT1G51270 | -1.976637432 | 8.8012E-10 | 6.5318E-09 |
| AT1G51420 | -1.90750809 | 9.9638E-24 | 2.0667E-22 |
| AT1G51470 | -1.788928385 | 5.0065E-06 | 2.3142E-05 |
| AT1G51500 | 1.8275083 | 3.3814E-73 | 3.275E-71 |
| AT1G51700 | 1.008968362 | 2.0868E-09 | 1.4832E-08 |
| AT1G51760 | 1.84051503 | 8.1019E-58 | 5.2912E-56 |
| AT1G51780 | 7.366824412 | 0.00019435 | 0.00068523 |
| AT1G51790 | -1.254414608 | 3.5925E-05 | 0.000145 |
| AT1G51805 | -1.101956541 | 2.0718E-12 | 1.9848E-11 |
| AT1G51830 | -2.480763915 | 9.8871E-30 | 2.6588E-28 |
| AT1G51840 | -3.068528198 | 6.765E-08 | 4.0396E-07 |
| AT1G51850 | -2.041764081 | 6.4008E-09 | 4.2944E-08 |
| AT1G51860 | -3.166171842 | 3.8722E-26 | 8.8777E-25 |
| AT1G51870 | -5.72330109 | 0.00398289 | 0.01058124 |
| AT1G51880 | -3.339057105 | 0.00333764 | 0.00902327 |
| AT1G51890 | -3.808777358 | 1.0139E-36 | 3.533E-35 |
| AT1G51920 | -5.512942253 | 0.008136 | 0.01999707 |
| AT1G51940 | -1.646892843 | 3.9485E-20 | 6.6073E-19 |
| AT1G52000 | -1.713495689 | 2.5481E-45 | 1.1766E-43 |
| AT1G52060 | -1.167860462 | 0.00519911 | 0.01344663 |
| AT1G52080 | 2.332475058 | 6.9588E-47 | 3.3902E-45 |
| AT1G52130 | -6.374706565 | 0.00041612 | 0.00137235 |
| AT1G52140 | -1.354859268 | 2.6828E-07 | 1.4755E-06 |
| AT1G52190 | -2.195926413 | 1.4351E-13 | 1.5229E-12 |
| AT1G52290 | -1.005905537 | 0.00599576 | 0.0152635 |
| AT1G52330 | -1.597186205 | 0.00387384 | 0.0103239 |
| AT1G52342 | -1.509205891 | 2.2532E-06 | 1.0942E-05 |
| AT1G52343 | -6.054127248 | 0.00134812 | 0.00399007 |
| AT1G52560 | 2.549145697 | 4.8796E-57 | 3.1243E-55 |
| AT1G52565 | 2.837772384 | 1.44E-34 | 4.6165E-33 |
| AT1G52660 | -7.247429815 | 1.1402E-05 | 5.0109E-05 |
| AT1G52690 | 5.327230992 | 6.4931E-12 | 5.9213E-11 |
| AT1G52700 | -1.643416554 | 0.00055551 | 0.00178998 |
| AT1G52720 | 1.060529338 | 2.3821E-16 | 3.1239E-15 |
| AT1G52750 | -1.156988371 | 0.00348482 | 0.00937571 |
| AT1G52827 | 2.828457028 | 2.4578E-86 | 3.1038E-84 |
| AT1G52855 | 3.792548729 | 1.6194E-40 | 6.306E-39 |
| AT1G52880 | 2.105389142 | 1.7749E-23 | 3.6387E-22 |
| AT1G52890 | 7.389765935 | 2.8441E-15 | 3.447E-14 |
| AT1G53090 | 1.03027051 | 5.676E-13 | 5.7054E-12 |
| AT1G53100 | 2.679353957 | 2.2883E-09 | 1.6159E-08 |
| AT1G53110 | 1.021816488 | 1.4019E-06 | 7.0087E-06 |
| AT1G53130 | -3.686511127 | 0.00085611 | 0.00265161 |
| AT1G53163 | -2.97313546 | 0.00266309 | 0.0073839 |
| AT1G53170 | 1.97864873 | 1.4197E-37 | 5.0863E-36 |
| AT1G53230 | -1.579813822 | 4.7582E-07 | 2.5275E-06 |
| AT1G53320 | 1.246746243 | 1.8999E-20 | 3.2288E-19 |
| AT1G53340 | -1.887017743 | 0.00697566 | 0.01744482 |
| AT1G53390 | -1.051505875 | 1.1251E-09 | 8.2508E-09 |
| AT1G53470 | 3.416390013 | 5.051E-160 | 1.604E-157 |
| AT1G53490 | 1.060119381 | 0.00025185 | 0.00086763 |
| AT1G53540 | 3.263248104 | 1.1478E-20 | 1.986E-19 |
| AT1G53560 | 1.804043377 | 5.6417E-34 | 1.7738E-32 |
| AT1G53570 | 1.103410271 | 4.1723E-18 | 6.1489E-17 |
| AT1G53580 | 1.557169793 | 5.7154E-46 | 2.6716E-44 |
| AT1G53625 | -3.301172031 | 1.3E-28 | 3.2944E-27 |
| AT1G53800 | -1.079048422 | 5.0817E-12 | 4.6876E-11 |
| AT1G53830 | -2.515949344 | 2.7518E-10 | 2.1475E-09 |
| AT1G53950 | -6.751983991 | 9.3199E-05 | 0.00034987 |
| AT1G53990 | -4.595033411 | 0.00729422 | 0.01815255 |
| AT1G54000 | -1.982954991 | 3.1E-60 | 2.1473E-58 |
| AT1G54010 | -1.138374913 | 0.00125787 | 0.00375113 |
| AT1G54100 | 2.754604991 | 1.352E-185 | 6.06E-183 |
| AT1G54120 | 1.548959853 | 1.0067E-09 | 7.4203E-09 |
| AT1G54130 | 1.711320794 | 8.0003E-35 | 2.6013E-33 |
| AT1G54160 | 3.052169749 | 1.2846E-32 | 3.8635E-31 |
| AT1G54170 | 1.089967908 | 2.8239E-14 | 3.1457E-13 |
| AT1G54210 | 1.314331911 | 4.9474E-09 | 3.3607E-08 |
| AT1G54215 | -5.632346788 | 0.00533963 | 0.01376178 |
| AT1G54290 | 1.592386174 | 3.5775E-28 | 8.8501E-27 |
| AT1G54385 | -1.38597766 | 1.287E-08 | 8.417E-08 |
| AT1G54560 | 2.704335259 | 0.00079045 | 0.00246429 |
| AT1G54570 | 1.109332979 | 9.1505E-22 | 1.7074E-20 |
| AT1G54575 | 1.800474833 | 6.9263E-47 | 3.3829E-45 |
| AT1G54690 | -1.175264656 | 2.2272E-06 | 1.0834E-05 |
| AT1G54710 | 1.736771018 | 4.7153E-42 | 1.9385E-40 |
| AT1G54820 | -1.679352065 | 1.1801E-16 | 1.5802E-15 |
| AT1G54830 | 1.371217697 | 1.4804E-16 | 1.9686E-15 |
| AT1G54860 | -5.487936547 | 0.0086588 | 0.02112304 |
| AT1G54890 | -5.327239623 | 4.1345E-14 | 4.5678E-13 |
| AT1G54940 | -6.930707223 | 4.4563E-05 | 0.00017709 |
| AT1G54970 | -6.306735138 | 3.2278E-07 | 1.7558E-06 |
| AT1G55020 | -1.773371982 | 1.9466E-50 | 1.062E-48 |
| AT1G55110 | 1.044441482 | 8.8224E-15 | 1.0284E-13 |
| AT1G55205 | -1.820205375 | 1.8692E-06 | 9.1805E-06 |
| AT1G55240 | -2.758813837 | 0.0002152 | 0.00075156 |
| AT1G55280 | 1.559421879 | 1.5772E-09 | 1.1412E-08 |
| AT1G55320 | -1.063178968 | 2.172E-10 | 1.7149E-09 |
| AT1G55370 | -1.232425021 | 2.91E-10 | 2.2656E-09 |
| AT1G55730 | 1.381950423 | 1.4296E-16 | 1.9043E-15 |
| AT1G55760 | 1.591475389 | 8.5631E-11 | 7.0586E-10 |
| AT1G55775 | -4.13353695 | 0.00331078 | 0.00896789 |
| AT1G55810 | -1.115269171 | 8.2599E-11 | 6.8235E-10 |
| AT1G55820 | 1.255169594 | 1.2005E-12 | 1.1747E-11 |
| AT1G55860 | 1.275333027 | 1.6521E-21 | 3.0187E-20 |
| AT1G55915 | 1.01110779 | 0.00072238 | 0.00226752 |
| AT1G55920 | 1.161612779 | 7.4072E-19 | 1.1463E-17 |
| AT1G55940 | -2.373018013 | 0.00877829 | 0.02138031 |
| AT1G56220 | 1.292007742 | 2.8417E-34 | 9.0216E-33 |
| AT1G56340 | -1.007998954 | 1.9791E-16 | 2.6089E-15 |
| AT1G56430 | -1.238388335 | 3.5943E-06 | 1.6975E-05 |
| AT1G56550 | -1.447242078 | 1.1339E-08 | 7.448E-08 |
| AT1G56600 | 5.602671736 | 6.5288E-49 | 3.415E-47 |
| AT1G56630 | -2.519302809 | 7.7386E-08 | 4.5898E-07 |
| AT1G56680 | -2.47578776 | 0.00151052 | 0.00442309 |
| AT1G57560 | -2.063819314 | 1.2941E-05 | 5.6279E-05 |
| AT1G57570 | -4.207343601 | 0.00269962 | 0.00747068 |
| AT1G57590 | 1.312523561 | 3.1836E-12 | 2.9996E-11 |
| AT1G57630 | -1.572255925 | 0.00178285 | 0.00512609 |
| AT1G57750 | 2.850000106 | 0.00982181 | 0.02361739 |
| AT1G57990 | -1.494153424 | 4.0377E-12 | 3.7646E-11 |
| AT1G58170 | -1.286883564 | 0.00043788 | 0.00143788 |
| AT1G58180 | 1.145696779 | 1.1382E-15 | 1.4232E-14 |
| AT1G58200 | 1.550622354 | 1.8749E-43 | 8.1016E-42 |
| AT1G58270 | 1.841884282 | 1.2536E-86 | 1.6008E-84 |
| AT1G58340 | 2.812290786 | 5.4838E-84 | 6.6675E-82 |
| AT1G58360 | 2.148132993 | 2.1891E-85 | 2.7194E-83 |
| AT1G58370 | -1.791486061 | 4.8108E-09 | 3.2748E-08 |
| AT1G58420 | 1.035376925 | 0.00606013 | 0.01541894 |
| AT1G58520 | 1.04940603 | 1.7637E-10 | 1.4047E-09 |
| AT1G58590 | 1.443890254 | 4.5416E-13 | 4.6036E-12 |
| AT1G59640 | 1.439671614 | 2.775E-06 | 1.3328E-05 |
| AT1G59940 | -1.92551664 | 1.5622E-05 | 6.7061E-05 |
| AT1G08283 | -2.603339537 | 0.0045955 | 0.01203527 |
| AT1G60000 | -1.743870842 | 1.2392E-34 | 3.9895E-33 |
| AT1G60010 | -1.135791985 | 9.6777E-14 | 1.0454E-12 |
| AT1G60060 | -2.279219706 | 7.8006E-06 | 3.5134E-05 |
| AT1G60140 | 1.935612389 | 3.5672E-47 | 1.7497E-45 |
| AT1G60190 | 5.867405644 | 0 | 0 |
| AT1G60390 | -2.601344591 | 4.604E-26 | 1.0492E-24 |
| AT1G60440 | 1.595409234 | 4.2586E-30 | 1.1672E-28 |
| AT1G60470 | 1.810122301 | 6.2905E-25 | 1.3707E-23 |
| AT1G60630 | -1.762767536 | 2.0955E-06 | 1.0232E-05 |
| AT1G60660 | -1.526499834 | 1.7575E-10 | 1.4007E-09 |
| AT1G60720 | 8.289911249 | 5.0767E-07 | 2.6856E-06 |
| AT1G60740 | -2.284582484 | 8.3613E-08 | 4.9335E-07 |
| AT1G60750 | 3.019169692 | 1.2765E-63 | 9.791E-62 |
| AT1G60787 | -5.6927871 | 0.00434892 | 0.0114525 |
| AT1G60950 | -1.547310974 | 5.5529E-41 | 2.1884E-39 |
| AT1G61050 | -2.331792943 | 0.00031418 | 0.00106219 |
| AT1G61080 | -3.847105726 | 0.00901743 | 0.02189278 |
| AT1G61120 | 6.157033468 | 9.0763E-07 | 4.6444E-06 |
| AT1G61140 | 1.093779918 | 6.8183E-14 | 7.4215E-13 |
| AT1G61170 | -1.085783088 | 0.00020312 | 0.00071363 |
| AT1G61255 | 3.370593638 | 4.3026E-48 | 2.1711E-46 |
| AT1G61260 | 1.381360311 | 5.071E-11 | 4.2931E-10 |
| AT1G61340 | 3.015557253 | 8.5234E-59 | 5.7134E-57 |
| AT1G61370 | 1.065866657 | 4.573E-05 | 0.00018141 |
| AT1G61560 | -2.19455671 | 8.3795E-13 | 8.3061E-12 |
| AT1G61590 | -1.232192768 | 0.00179411 | 0.00515393 |
| AT1G61600 | -1.38813093 | 2.7621E-05 | 0.0001137 |
| AT1G61667 | -2.227955995 | 3.665E-08 | 2.2715E-07 |
| AT1G61690 | 1.414387866 | 8.2056E-30 | 2.2118E-28 |
| AT1G61740 | 1.356532702 | 6.6808E-32 | 1.9553E-30 |
| AT1G61800 | 4.031481359 | 3.4575E-83 | 4.1378E-81 |
| AT1G61810 | -2.874510689 | 1.4488E-05 | 6.2519E-05 |
| AT1G61820 | -1.260855575 | 1.1799E-07 | 6.823E-07 |
| AT1G61890 | 2.62862748 | 2.762E-160 | 8.893E-158 |
| AT1G61930 | -2.131887527 | 1.6739E-06 | 8.2709E-06 |
| AT1G62000 | 5.945873524 | 0.00966118 | 0.02327522 |
| AT1G62130 | 1.0508725 | 1.196E-07 | 6.9089E-07 |
| AT1G62180 | 1.370313481 | 1.1973E-22 | 2.3332E-21 |
| AT1G62300 | 1.131504569 | 8.0008E-19 | 1.2348E-17 |
| AT1G62305 | 1.804996656 | 1.8735E-20 | 3.1864E-19 |
| AT1G62310 | 1.066958475 | 7.7922E-10 | 5.8226E-09 |
| AT1G62320 | -2.686482511 | 3.0298E-06 | 1.4486E-05 |
| AT1G62333 | -3.236494931 | 0.00823295 | 0.02020493 |
| AT1G62440 | -1.197421426 | 0.00689936 | 0.01728091 |
| AT1G62540 | 1.179941042 | 1.7736E-14 | 2.017E-13 |
| AT1G62570 | 3.308410237 | 4.252E-289 | 4.628E-286 |
| AT1G62620 | 2.638878023 | 2.4936E-06 | 1.2025E-05 |
| AT1G62710 | 2.143106277 | 3.1339E-25 | 6.9079E-24 |
| AT1G62740 | 1.029920953 | 1.2773E-19 | 2.0663E-18 |
| AT1G62750 | -1.006080575 | 1.3944E-20 | 2.3928E-19 |
| AT1G62770 | -1.866226185 | 2.9269E-06 | 1.402E-05 |
| AT1G62800 | -1.173786901 | 1.8703E-08 | 1.2029E-07 |
| AT1G62870 | -1.716854788 | 8.6686E-08 | 5.1004E-07 |
| AT1G62975 | -1.841544931 | 3.4778E-19 | 5.4975E-18 |
| AT1G62980 | -3.59687644 | 1.6366E-17 | 2.3092E-16 |
| AT1G63010 | 1.920442202 | 6.7669E-75 | 6.6672E-73 |
| AT1G63245 | -3.728576251 | 5.2219E-11 | 4.4159E-10 |
| AT1G63410 | -4.445252377 | 0.00103999 | 0.00315741 |
| AT1G63420 | 1.421212686 | 1.8529E-23 | 3.7916E-22 |
| AT1G63440 | 2.398889142 | 6.227E-66 | 4.9943E-64 |
| AT1G63600 | -6.601827631 | 0.000213 | 0.00074513 |
| AT1G63650 | -1.430352975 | 6.4672E-07 | 3.375E-06 |
| AT1G63720 | 1.801100827 | 3.0635E-18 | 4.559E-17 |
| AT1G63860 | 1.279244842 | 3.068E-07 | 1.6745E-06 |
| AT1G64110 | 1.356019537 | 8.8551E-09 | 5.8823E-08 |
| AT1G64160 | -4.442418926 | 0.0012295 | 0.00367179 |
| AT1G64170 | -1.700877608 | 7.9739E-11 | 6.6039E-10 |
| AT1G64340 | -2.762375143 | 6.4489E-09 | 4.3241E-08 |
| AT1G64380 | 1.821099921 | 1.2429E-27 | 2.9779E-26 |
| AT1G64390 | -1.196305399 | 1.2068E-29 | 3.2262E-28 |
| AT1G64405 | -2.110359595 | 0.00371573 | 0.00994173 |
| AT1G64480 | -5.928338175 | 0.00202841 | 0.00577403 |
| AT1G64500 | -1.136405819 | 6.2518E-06 | 2.8438E-05 |
| AT1G64563 | 2.410788641 | 7.5525E-06 | 3.4071E-05 |
| AT1G64640 | -1.728768088 | 1.2098E-12 | 1.1832E-11 |
| AT1G64660 | 2.20841349 | 1.4857E-42 | 6.1857E-41 |
| AT1G64740 | 1.140086725 | 4.6479E-10 | 3.5473E-09 |
| AT1G64780 | -1.937732694 | 1.5986E-21 | 2.9256E-20 |
| AT1G64810 | 1.063671088 | 2.567E-11 | 2.2277E-10 |
| AT1G64890 | 1.006693825 | 4.62E-07 | 2.4599E-06 |
| AT1G64920 | -1.691741416 | 0.00588551 | 0.01501295 |
| AT1G65060 | -2.711097347 | 6.249E-117 | 1.161E-114 |
| AT1G65190 | -1.733893236 | 9.0272E-28 | 2.1835E-26 |
| AT1G65310 | -2.384642548 | 0.00343372 | 0.00925676 |
| AT1G08757 | -4.482096512 | 0.00176823 | 0.0050916 |
| AT1G65481 | -6.231359284 | 2.2085E-11 | 1.9281E-10 |
| AT1G08763 | -5.668966979 | 1.4659E-06 | 7.305E-06 |
| AT1G65485 | -7.105774003 | 2.2208E-05 | 9.2955E-05 |
| AT1G65490 | -1.471359645 | 0.0073645 | 0.01830752 |
| AT1G65560 | -1.422893616 | 9.5997E-15 | 1.1144E-13 |
| AT1G65610 | -2.571735064 | 6.6394E-16 | 8.4407E-15 |
| AT1G65620 | -2.19673738 | 4.2599E-07 | 2.2788E-06 |
| AT1G65660 | 1.23927049 | 6.6652E-29 | 1.7176E-27 |
| AT1G65680 | -2.923436817 | 0.0033642 | 0.00908757 |
| AT1G65710 | -1.845494662 | 5.2112E-08 | 3.1579E-07 |
| AT1G65800 | -1.856930647 | 4.5786E-19 | 7.1831E-18 |
| AT1G65845 | -1.160149503 | 3.7647E-06 | 1.7721E-05 |
| AT1G65850 | -4.106970576 | 0.00434469 | 0.01144398 |
| AT1G65860 | -2.579965961 | 1.7354E-44 | 7.7475E-43 |
| AT1G65985 | -1.907477718 | 1.8242E-08 | 1.1746E-07 |
| AT1G66050 | 1.211512148 | 0.00053619 | 0.00173306 |
| AT1G66060 | 2.975055359 | 0.00052201 | 0.00169154 |
| AT1G66140 | -1.092105833 | 0.00011725 | 0.00043185 |
| AT1G66190 | -1.514475602 | 1.2159E-07 | 7.0168E-07 |
| AT1G66200 | -1.439656178 | 9.4243E-37 | 3.2889E-35 |
| AT1G66230 | 1.079068802 | 0.0049161 | 0.01279055 |
| AT1G66250 | -1.083508264 | 1.2136E-06 | 6.1061E-06 |
| AT1G66370 | 2.893955306 | 0.00249297 | 0.00696035 |
| AT1G66390 | 2.949996669 | 2.3534E-43 | 1.0131E-41 |
| AT1G66440 | -6.038683536 | 0.00140041 | 0.00412932 |
| AT1G66470 | -1.23831636 | 0.00056765 | 0.00182419 |
| AT1G66500 | 2.780703345 | 3.9144E-36 | 1.3217E-34 |
| AT1G66520 | -1.183528702 | 0.00043553 | 0.00143098 |
| AT1G66550 | 6.046496375 | 4.534E-06 | 2.1086E-05 |
| AT1G66700 | -2.438464517 | 3.242E-19 | 5.1391E-18 |
| AT1G66760 | 3.325758646 | 3.308E-143 | 8.592E-141 |
| AT1G66780 | 2.220023357 | 0.00171775 | 0.00496011 |
| AT1G08847 | -1.321344579 | 3.7365E-06 | 1.7599E-05 |
| AT1G66830 | 2.672210379 | 7.1138E-18 | 1.0305E-16 |
| AT1G66930 | -4.683386496 | 0.00031229 | 0.00105675 |
| AT1G66940 | -2.397349053 | 2.9403E-17 | 4.0709E-16 |
| AT1G67000 | -3.489803823 | 0.00011021 | 0.00040848 |
| AT1G67050 | -1.463663995 | 1.8006E-06 | 8.8648E-06 |
| AT1G67148 | -2.43148195 | 1.4326E-05 | 6.1901E-05 |
| AT1G67180 | -1.236475417 | 0.00125553 | 0.00374463 |
| AT1G67270 | -6.136094991 | 0.00012497 | 0.00045794 |
| AT1G67300 | 1.704565396 | 3.4921E-45 | 1.6029E-43 |
| AT1G67360 | 1.650248036 | 5.1302E-49 | 2.6896E-47 |
| AT1G67365 | 2.714944594 | 9.0979E-18 | 1.3038E-16 |
| AT1G67370 | 1.914257398 | 3.8511E-10 | 2.957E-09 |
| AT1G67470 | -1.365611127 | 0.00027869 | 0.00095165 |
| AT1G67480 | -1.025137997 | 1.2136E-17 | 1.723E-16 |
| AT1G67520 | -1.413875034 | 0.00065163 | 0.00206702 |
| AT1G67630 | -1.113092033 | 2.1724E-06 | 1.0581E-05 |
| AT1G67730 | 1.013617165 | 4.2569E-23 | 8.4981E-22 |
| AT1G67750 | -2.157545311 | 2.5863E-27 | 6.1135E-26 |
| AT1G67800 | -1.043284023 | 1.8115E-07 | 1.0199E-06 |
| AT1G08957 | -5.862455114 | 0.00259788 | 0.0072215 |
| AT1G67810 | -2.269749932 | 3.0498E-22 | 5.8288E-21 |
| AT1G67910 | -2.512771899 | 1.8658E-09 | 1.3336E-08 |
| AT1G67920 | 2.248929636 | 3.1261E-20 | 5.2697E-19 |
| AT1G67980 | -3.11865819 | 4.7393E-08 | 2.8912E-07 |
| AT1G68020 | 1.518096943 | 3.3656E-29 | 8.8527E-28 |
| AT1G68130 | -1.163093661 | 3.3333E-08 | 2.0767E-07 |
| AT1G68150 | -2.486767246 | 1.3477E-08 | 8.7913E-08 |
| AT1G68238 | -1.103891604 | 6.513E-07 | 3.3974E-06 |
| AT1G68340 | 1.377781287 | 4.4528E-08 | 2.7295E-07 |
| AT1G68390 | -1.869859384 | 3.7677E-06 | 1.7732E-05 |
| AT1G68430 | -1.027664487 | 0.00751432 | 0.01862729 |
| AT1G68440 | 1.778628212 | 8.5626E-56 | 5.2756E-54 |
| AT1G68450 | -1.848216198 | 1.7452E-08 | 1.1266E-07 |
| AT1G68500 | 3.688219033 | 1.651E-236 | 1.217E-233 |
| AT1G68520 | -1.439870884 | 2.1784E-35 | 7.2061E-34 |
| AT1G68530 | 1.66335599 | 1.0035E-49 | 5.3467E-48 |
| AT1G68560 | -1.118669338 | 1.8634E-23 | 3.8099E-22 |
| AT1G68570 | 2.12749927 | 3.3552E-41 | 1.3315E-39 |
| AT1G68580 | 1.001801102 | 7.1817E-15 | 8.4705E-14 |
| AT1G68610 | 2.933642667 | 0.00020524 | 0.00072033 |
| AT1G68620 | 1.437102739 | 1.4022E-19 | 2.2652E-18 |
| AT1G68650 | -1.260437171 | 5.3519E-09 | 3.6172E-08 |
| AT1G68780 | -1.550655106 | 1.1193E-11 | 9.9899E-11 |
| AT1G68840 | -1.348419561 | 1.4347E-09 | 1.0428E-08 |
| AT1G68880 | 1.573582857 | 2.763E-18 | 4.1225E-17 |
| AT1G69040 | -1.254601778 | 9.0165E-16 | 1.1349E-14 |
| AT1G69220 | 1.07363499 | 5.4641E-16 | 6.9932E-15 |
| AT1G69240 | -3.527508131 | 5.0665E-06 | 2.3396E-05 |
| AT1G69250 | 1.012452875 | 2.9452E-16 | 3.8403E-15 |
| AT1G69252 | 1.955843206 | 8.5791E-63 | 6.4507E-61 |
| AT1G69260 | 4.714199014 | 0 | 0 |
| AT1G69270 | 1.526054343 | 0.00011444 | 0.00042261 |
| AT1G69295 | 1.917037736 | 1.1506E-67 | 9.8139E-66 |
| AT1G69360 | 2.053123831 | 2.4246E-35 | 8.0087E-34 |
| AT1G69400 | 1.323670474 | 0.00014409 | 0.00052099 |
| AT1G69430 | 2.142015279 | 0.00018846 | 0.00066642 |
| AT1G69470 | 6.246101285 | 0.00611872 | 0.01555574 |
| AT1G69480 | 3.231601751 | 9.7197E-61 | 6.8705E-59 |
| AT1G69490 | 2.81905307 | 1.6246E-19 | 2.6115E-18 |
| AT1G69600 | 1.587647842 | 1.5462E-09 | 1.1199E-08 |
| AT1G69610 | 2.269633188 | 3.783E-48 | 1.9131E-46 |
| AT1G69780 | -1.503610344 | 9.368E-12 | 8.4337E-11 |
| AT1G69810 | -1.282450638 | 5.5031E-06 | 2.5284E-05 |
| AT1G69870 | 1.524899463 | 8.8179E-33 | 2.6768E-31 |
| AT1G69880 | 1.542668137 | 0.00038159 | 0.00126963 |
| AT1G69910 | -1.09883094 | 1.0856E-07 | 6.306E-07 |
| AT1G69920 | -5.052711338 | 3.1716E-19 | 5.0345E-18 |
| AT1G69930 | 2.660026206 | 1.2727E-05 | 5.5379E-05 |
| AT1G70210 | -1.882836351 | 8.1346E-19 | 1.2538E-17 |
| AT1G70250 | -1.489764347 | 6.4319E-05 | 0.00024818 |
| AT1G70270 | -5.661775248 | 0.0051847 | 0.01341544 |
| AT1G70290 | 1.584359072 | 6.3607E-18 | 9.2371E-17 |
| AT1G70320 | 1.134314722 | 2.8657E-21 | 5.1578E-20 |
| AT1G70370 | -1.501841368 | 3.0611E-46 | 1.4427E-44 |
| AT1G70420 | 1.776583348 | 2.1803E-31 | 6.2846E-30 |
| AT1G70440 | 2.857563536 | 1.0567E-11 | 9.4609E-11 |
| AT1G70460 | -2.223762704 | 4.13E-07 | 2.2129E-06 |
| AT1G70518 | 1.039577329 | 0.0002771 | 0.00094664 |
| AT1G70550 | -1.076744932 | 2.2292E-07 | 1.2431E-06 |
| AT1G70640 | 2.697224737 | 9.3885E-29 | 2.3924E-27 |
| AT1G70690 | -1.945943983 | 6.9055E-11 | 5.7545E-10 |
| AT1G70700 | 1.097665122 | 1.4652E-24 | 3.1418E-23 |
| AT1G70890 | -1.602026858 | 1.9204E-33 | 5.9642E-32 |
| AT1G70900 | 1.023822437 | 3.9776E-10 | 3.049E-09 |
| AT1G70990 | -8.265878806 | 1.1887E-07 | 6.8683E-07 |
| AT1G71000 | 3.201349565 | 2.4189E-55 | 1.4705E-53 |
| AT1G71240 | 1.070421086 | 1.2772E-13 | 1.3636E-12 |
| AT1G71330 | 2.668270209 | 3.5452E-20 | 5.9497E-19 |
| AT1G71340 | 1.412219226 | 7.2028E-22 | 1.3506E-20 |
| AT1G71350 | 1.167453692 | 3.7558E-15 | 4.5232E-14 |
| AT1G71360 | 1.821585894 | 1.8529E-30 | 5.1277E-29 |
| AT1G71400 | -1.599006894 | 3.9514E-07 | 2.1232E-06 |
| AT1G71490 | 1.135542778 | 0.00916747 | 0.0222075 |
| AT1G71520 | 4.18043844 | 4.1678E-72 | 3.9205E-70 |
| AT1G71697 | 1.387589754 | 7.7437E-23 | 1.5246E-21 |
| AT1G71740 | -1.749005098 | 0.00011665 | 0.00042991 |
| AT1G71890 | 2.577091262 | 5.7815E-13 | 5.7987E-12 |
| AT1G71910 | 2.542320668 | 1.3192E-13 | 1.4058E-12 |
| AT1G71950 | 1.214011794 | 1.1035E-17 | 1.5726E-16 |
| AT1G09287 | 2.580628233 | 0.00227419 | 0.00640742 |
| AT1G71960 | 1.332453211 | 1.8523E-16 | 2.4473E-15 |
| AT1G72120 | 2.601610075 | 6.3022E-44 | 2.7703E-42 |
| AT1G72140 | -1.991745873 | 4.6734E-16 | 6.0048E-15 |
| AT1G72230 | -1.747643703 | 3.9838E-13 | 4.0616E-12 |
| AT1G72275 | -1.330655154 | 2.0974E-05 | 8.8079E-05 |
| AT1G72360 | 1.262653958 | 0.00221985 | 0.00626745 |
| AT1G72416 | 1.270580723 | 4.0619E-09 | 2.7932E-08 |
| AT1G72430 | -2.668993707 | 2.2413E-21 | 4.0596E-20 |
| AT1G72520 | 1.296515215 | 1.0029E-06 | 5.0988E-06 |
| AT1G72680 | 1.59894066 | 1.7785E-38 | 6.5255E-37 |
| AT1G72690 | 1.052090505 | 0.00050859 | 0.00165133 |
| AT1G72760 | 3.307157694 | 3.5518E-07 | 1.9221E-06 |
| AT1G72770 | 3.413921276 | 2.212E-238 | 1.685E-235 |
| AT1G72800 | 1.814800083 | 1.1076E-07 | 6.4239E-07 |
| AT1G72890 | -1.169137832 | 0.0060373 | 0.01536584 |
| AT1G72910 | -2.678169712 | 1.9354E-05 | 8.1773E-05 |
| AT1G72930 | -1.696794928 | 1.4828E-23 | 3.0562E-22 |
| AT1G73010 | 1.324043553 | 0.00107283 | 0.00324504 |
| AT1G73066 | 2.201350495 | 0.00351546 | 0.00945259 |
| AT1G73080 | 1.033496737 | 1.4761E-12 | 1.4322E-11 |
| AT1G73160 | -3.004328969 | 0.00047823 | 0.0015603 |
| AT1G73210 | 1.878845202 | 2.3757E-13 | 2.465E-12 |
| AT1G73260 | -1.273923209 | 2.4541E-15 | 2.9854E-14 |
| AT1G73280 | -2.293867101 | 1.0511E-08 | 6.9298E-08 |
| AT1G73325 | -4.541566626 | 1.785E-13 | 1.8794E-12 |
| AT1G73330 | -1.445962892 | 2.8853E-35 | 9.5168E-34 |
| AT1G73390 | 3.212964829 | 1.585E-187 | 7.393E-185 |
| AT1G73480 | 4.813478276 | 0 | 0 |
| AT1G73500 | 1.910476044 | 6.234E-46 | 2.9081E-44 |
| AT1G73510 | 6.902563344 | 0.00033577 | 0.00112903 |
| AT1G73580 | -4.34483332 | 6.2127E-06 | 2.8283E-05 |
| AT1G73600 | -2.146730523 | 5.3626E-67 | 4.4252E-65 |
| AT1G73620 | -2.709118764 | 1.3415E-28 | 3.3957E-27 |
| AT1G73630 | -1.551231038 | 3.0524E-06 | 1.4581E-05 |
| AT1G73640 | -2.167766783 | 8.9384E-07 | 4.583E-06 |
| AT1G73680 | 2.126814834 | 5.8427E-62 | 4.2533E-60 |
| AT1G73750 | 1.959084181 | 9.6222E-28 | 2.3225E-26 |
| AT1G73830 | -2.249892406 | 0.00017136 | 0.00061135 |
| AT1G73880 | 3.293448114 | 6.4E-167 | 2.217E-164 |
| AT1G73920 | 2.730822025 | 1.309E-149 | 3.786E-147 |
| AT1G74010 | -1.222495805 | 0.00023882 | 0.00082635 |
| AT1G74070 | -2.037160901 | 1.2535E-46 | 6.0192E-45 |
| AT1G74080 | -2.02952845 | 2.1566E-06 | 1.0509E-05 |
| AT1G74090 | -2.634546704 | 1.0748E-57 | 6.9797E-56 |
| AT1G74250 | 1.110442432 | 5.204E-18 | 7.6302E-17 |
| AT1G74310 | 1.96916271 | 4.6146E-49 | 2.4304E-47 |
| AT1G74440 | -1.646453897 | 0.00762174 | 0.01886698 |
| AT1G09467 | 1.07497575 | 0.00022113 | 0.00076986 |
| AT1G74458 | -1.522239895 | 2.5513E-05 | 0.00010588 |
| AT1G74460 | -2.105492192 | 7.5025E-29 | 1.9204E-27 |
| AT1G74500 | -2.512371438 | 1.101E-16 | 1.4769E-15 |
| AT1G74670 | -2.31171804 | 4.1626E-11 | 3.5437E-10 |
| AT1G74750 | 1.338222436 | 4.4049E-14 | 4.8548E-13 |
| AT1G74770 | 1.444746053 | 8.115E-13 | 8.0474E-12 |
| AT1G74790 | 1.649395377 | 4.7475E-51 | 2.621E-49 |
| AT1G74830 | 2.035002367 | 0.00055773 | 0.00179609 |
| AT1G74840 | 1.587562699 | 3.1513E-36 | 1.0751E-34 |
| AT1G74950 | 1.281017509 | 1.8254E-24 | 3.9069E-23 |
| AT1G75030 | 1.470487167 | 1.1126E-06 | 5.6264E-06 |
| AT1G75040 | -1.303078544 | 8.6417E-11 | 7.1132E-10 |
| AT1G75090 | -1.117698375 | 0.00770693 | 0.019049 |
| AT1G75170 | 1.382813945 | 2.874E-26 | 6.6356E-25 |
| AT1G09537 | 2.472723018 | 0.00062771 | 0.00199919 |
| AT1G75370 | 1.998485391 | 8.9665E-72 | 8.3316E-70 |
| AT1G75380 | 1.084756095 | 1.8121E-25 | 4.053E-24 |
| AT1G75400 | 1.098309944 | 3.4436E-18 | 5.1113E-17 |
| AT1G75490 | 1.700864633 | 0.00711125 | 0.01775714 |
| AT1G75500 | -2.599314453 | 9.6099E-95 | 1.3729E-92 |
| AT1G75520 | -2.396188127 | 5.4996E-07 | 2.8932E-06 |
| AT1G75600 | 6.482701564 | 2.8496E-45 | 1.3132E-43 |
| AT1G75860 | 1.647812712 | 1.3694E-34 | 4.4026E-33 |
| AT1G76070 | 1.525215739 | 2.6713E-14 | 2.9815E-13 |
| AT1G76080 | -1.046773673 | 1.384E-15 | 1.7246E-14 |
| AT1G76090 | -1.789707024 | 7.8717E-29 | 2.0127E-27 |
| AT1G76110 | -1.413433013 | 8.1999E-08 | 4.8432E-07 |
| AT1G76130 | 1.189059489 | 4.4898E-11 | 3.8195E-10 |
| AT1G76180 | 2.534885846 | 5.851E-162 | 1.911E-159 |
| AT1G76380 | 1.242487066 | 7.9551E-15 | 9.349E-14 |
| AT1G76430 | -5.54491011 | 0.00690796 | 0.01730055 |
| AT1G76490 | 1.153420111 | 1.3715E-34 | 4.4031E-33 |
| AT1G76500 | -1.726678467 | 1.065E-05 | 4.6967E-05 |
| AT1G76530 | -2.243820331 | 0.00024739 | 0.00085343 |
| AT1G76580 | 1.348774538 | 3.7085E-23 | 7.4424E-22 |
| AT1G76590 | 2.969470176 | 9.8707E-96 | 1.428E-93 |
| AT1G76600 | 1.87245988 | 1.7585E-34 | 5.6219E-33 |
| AT1G76650 | 2.346909767 | 4.5583E-16 | 5.8766E-15 |
| AT1G76930 | -1.12738135 | 7.6589E-27 | 1.7974E-25 |
| AT1G76952 | -4.150192016 | 3.1116E-07 | 1.6963E-06 |
| AT1G76980 | 1.118986613 | 1.3663E-09 | 9.9368E-09 |
| AT1G77000 | 1.519127452 | 6.9331E-20 | 1.1409E-18 |
| AT1G77110 | -1.38651936 | 2.3183E-05 | 9.6773E-05 |
| AT1G77120 | 5.001909727 | 0 | 0 |
| AT1G77180 | 1.037363714 | 2.8695E-25 | 6.3372E-24 |
| AT1G77200 | 1.15341219 | 0.00044094 | 0.00144731 |
| AT1G77330 | -2.899346669 | 9.4271E-41 | 3.6835E-39 |
| AT1G77400 | -1.356449341 | 3.3344E-06 | 1.5833E-05 |
| AT1G77450 | 3.054617608 | 2.04E-193 | 1.014E-190 |
| AT1G77460 | -1.055551044 | 1.548E-07 | 8.8021E-07 |
| AT1G77520 | -3.68635583 | 4.7833E-52 | 2.7063E-50 |
| AT1G77530 | -3.682534209 | 4.3595E-20 | 7.279E-19 |
| AT1G77570 | 1.070889997 | 0.00974676 | 0.02345503 |
| AT1G77630 | -1.655627279 | 1.0748E-14 | 1.2427E-13 |
| AT1G77680 | 1.982570467 | 3.9654E-72 | 3.7611E-70 |
| AT1G77690 | -2.298491167 | 4.0236E-28 | 9.9215E-27 |
| AT1G77760 | 1.848059267 | 5.4296E-50 | 2.9202E-48 |
| AT1G77860 | -5.876076587 | 0.00242425 | 0.00679255 |
| AT1G77990 | -1.325166121 | 1.6102E-07 | 9.1305E-07 |
| AT1G78020 | -1.5201767 | 9.2551E-39 | 3.4067E-37 |
| AT1G78070 | 2.596127507 | 1.12E-151 | 3.281E-149 |
| AT1G78090 | -2.872747089 | 5.3734E-18 | 7.8633E-17 |
| AT1G78120 | -1.120888785 | 7.5163E-06 | 3.3921E-05 |
| AT1G78160 | 6.930040805 | 0.00064098 | 0.0020369 |
| AT1G78170 | 1.242977446 | 5.2011E-19 | 8.1374E-18 |
| AT1G09787 | -3.176755881 | 0.00227404 | 0.00640742 |
| AT1G78210 | 1.511064275 | 2.7531E-28 | 6.8625E-27 |
| AT1G78260 | -1.198595747 | 4.8828E-05 | 0.00019237 |
| AT1G78290 | -1.333380951 | 0.0003326 | 0.00111903 |
| AT1G78340 | -1.142346743 | 1.341E-13 | 1.4284E-12 |
| AT1G78370 | -1.581974603 | 8.0783E-35 | 2.6155E-33 |
| AT1G78430 | -1.196326385 | 4.3926E-06 | 2.0462E-05 |
| AT1G78440 | -2.785868308 | 8.7282E-05 | 0.00032928 |
| AT1G78450 | -1.378115839 | 0.00137543 | 0.00406301 |
| AT1G78460 | -1.518944994 | 2.3203E-10 | 1.8251E-09 |
| AT1G78530 | -1.32560685 | 0.00428435 | 0.01130459 |
| AT1G78610 | 1.83724736 | 7.5982E-63 | 5.732E-61 |
| AT1G78780 | -1.437964188 | 0.00774904 | 0.01914066 |
| AT1G78860 | -1.199831467 | 0.00018358 | 0.00065078 |
| AT1G78895 | 1.058971018 | 0.00019614 | 0.00069115 |
| AT1G78960 | 1.037802023 | 3.8265E-08 | 2.3663E-07 |
| AT1G78990 | -1.468554921 | 1.2575E-05 | 5.4792E-05 |
| AT1G78995 | -1.221209208 | 1.9032E-14 | 2.1525E-13 |
| AT1G79060 | -1.261140571 | 1.4017E-05 | 6.0671E-05 |
| AT1G79180 | -1.525315716 | 0.00470816 | 0.01230073 |
| AT1G79270 | 2.191910652 | 8.172E-109 | 1.426E-106 |
| AT1G79320 | -4.023133011 | 0.00011543 | 0.00042581 |
| AT1G79360 | 1.651698172 | 3.4884E-06 | 1.6512E-05 |
| AT1G09863 | 2.032046994 | 7.1993E-05 | 0.00027537 |
| AT1G79520 | 2.602309464 | 1.3859E-88 | 1.7999E-86 |
| AT1G79570 | 1.069831906 | 7.4016E-20 | 1.2172E-18 |
| AT1G79610 | 1.302502638 | 3.4918E-20 | 5.8645E-19 |
| AT1G79670 | -1.183182377 | 1.0222E-07 | 5.9591E-07 |
| AT1G79680 | -1.580596009 | 0.00138138 | 0.00407847 |
| AT1G79700 | 1.786530845 | 1.4927E-18 | 2.2641E-17 |
| AT1G79710 | 1.297677049 | 2.4143E-13 | 2.5016E-12 |
| AT1G79760 | -2.141831524 | 0.00020708 | 0.0007262 |
| AT1G79770 | -2.649400631 | 0.00181214 | 0.00520244 |
| AT1G79860 | -4.154199491 | 0.00346731 | 0.00933628 |
| AT1G79900 | 3.830339688 | 8.0687E-35 | 2.6155E-33 |
| AT1G80050 | -1.348467612 | 6.4388E-08 | 3.8518E-07 |
| AT1G80110 | 3.422301887 | 3.183E-95 | 4.5759E-93 |
| AT1G80120 | 1.891763069 | 1.0787E-18 | 1.6548E-17 |
| AT1G80130 | 2.301409162 | 1.1381E-14 | 1.3119E-13 |
| AT1G80160 | 3.111461212 | 5.5359E-28 | 1.3548E-26 |
| AT1G80170 | -1.503013147 | 1.5504E-06 | 7.7006E-06 |
| AT1G80240 | -2.726544681 | 7.1787E-18 | 1.0392E-16 |
| AT1G80270 | -1.365852762 | 2.2934E-22 | 4.4089E-21 |
| AT1G80280 | -1.111311304 | 6.6253E-12 | 6.0359E-11 |
| AT1G80340 | -2.91202611 | 7.9127E-32 | 2.307E-30 |
| AT1G80440 | 1.210375122 | 1.9877E-07 | 1.1147E-06 |
| AT1G80520 | -1.514020832 | 0.00081286 | 0.00252896 |
| AT1G80660 | 3.184139763 | 0.00266287 | 0.0073839 |
| AT1G80720 | -1.119731306 | 3.1493E-05 | 0.00012839 |
| AT1G80840 | 3.639194623 | 1.3486E-65 | 1.0741E-63 |
| AT1G80850 | -1.005237193 | 0.00089604 | 0.00276332 |
| AT1G80920 | 1.911171766 | 2.167E-62 | 1.6082E-60 |
| AT1G80960 | 1.218785402 | 0.0003754 | 0.00125013 |
| AT1G80970 | 1.475864194 | 6.1562E-07 | 3.2238E-06 |
| AT2G01008 | 4.043726464 | 4.9146E-50 | 2.6501E-48 |
| AT2G01010 | 3.277509898 | 7.0887E-40 | 2.7278E-38 |
| AT2G01020 | 4.462052394 | 1.064E-100 | 1.6656E-98 |
| AT2G03875 | 1.668983666 | 3.1395E-05 | 0.00012806 |
| AT2G01290 | -1.145087258 | 1.8857E-09 | 1.347E-08 |
| AT2G01505 | -1.400266536 | 7.7654E-05 | 0.00029579 |
| AT2G01580 | -2.415638954 | 4.0246E-05 | 0.00016123 |
| AT2G01660 | -2.2343144 | 2.4627E-14 | 2.7594E-13 |
| AT2G01760 | -1.180570803 | 7.0968E-06 | 3.2078E-05 |
| AT2G01850 | 1.288402792 | 8.1692E-32 | 2.3788E-30 |
| AT2G01880 | -1.121203697 | 0.00295792 | 0.00810794 |
| AT2G01900 | -2.03937305 | 0.00203446 | 0.00578907 |
| AT2G01913 | -1.710115173 | 3.4954E-05 | 0.00014141 |
| AT2G01950 | -1.996865482 | 2.1793E-22 | 4.1967E-21 |
| AT2G01990 | -3.211541089 | 4.6169E-20 | 7.6975E-19 |
| AT2G02020 | -1.80904824 | 2.6598E-08 | 1.6744E-07 |
| AT2G02061 | 1.846672983 | 0.00041949 | 0.00138224 |
| AT2G02070 | -1.040987198 | 2.3048E-15 | 2.8143E-14 |
| AT2G02130 | -1.273657687 | 9.6427E-31 | 2.7144E-29 |
| AT2G02300 | -5.72330109 | 0.00398289 | 0.01058124 |
| AT2G02680 | -3.004693789 | 6.4863E-05 | 0.00025002 |
| AT2G02700 | 7.314248758 | 6.2985E-05 | 0.00024336 |
| AT2G02710 | 3.240071667 | 7.799E-146 | 2.122E-143 |
| AT2G02780 | -1.415502922 | 1.7804E-09 | 1.2749E-08 |
| AT2G02820 | -1.108511874 | 2.0793E-06 | 1.016E-05 |
| AT2G02950 | -1.877434965 | 1.7732E-15 | 2.1885E-14 |
| AT2G02990 | 2.349110632 | 1.1265E-52 | 6.4215E-51 |
| AT2G03020 | 1.884651338 | 0.0025426 | 0.00708162 |
| AT2G03200 | -2.322815247 | 3.4077E-15 | 4.1148E-14 |
| AT2G03360 | -5.801529198 | 6.1415E-07 | 3.2168E-06 |
| AT2G03505 | -2.963532526 | 3.7395E-09 | 2.5831E-08 |
| AT2G03720 | -3.589824194 | 5.1194E-06 | 2.3635E-05 |
| AT2G03750 | -2.300095175 | 6.231E-22 | 1.1742E-20 |
| AT2G03760 | 2.727390139 | 5.1737E-69 | 4.566E-67 |
| AT2G04050 | 2.342552563 | 9.3418E-13 | 9.22E-12 |
| AT2G04070 | -4.670741222 | 5.5367E-07 | 2.9114E-06 |
| AT2G04725 | 3.405732641 | 0.00608692 | 0.01548179 |
| AT2G04170 | -1.147869344 | 1.1545E-10 | 9.3746E-10 |
| AT2G04240 | 1.174539933 | 2.7205E-14 | 3.0334E-13 |
| AT2G04350 | 2.102219293 | 1.006E-102 | 1.596E-100 |
| AT2G04460 | -4.593101775 | 0.00066258 | 0.00209679 |
| AT2G04500 | -3.93435985 | 0.00816115 | 0.02005025 |
| AT2G04570 | 2.035969662 | 5.6733E-61 | 4.0525E-59 |
| AT2G04805 | -1.749223403 | 0.00632425 | 0.01601948 |
| AT2G04680 | -3.070769568 | 8.0252E-05 | 0.00030472 |
| AT2G04780 | -1.344731837 | 1.3131E-22 | 2.5545E-21 |
| AT2G04790 | -1.150545497 | 0.00046854 | 0.00153128 |
| AT2G05430 | -5.500500589 | 0.00764395 | 0.01891172 |
| AT2G05510 | -1.203872632 | 1.8949E-15 | 2.3312E-14 |
| AT2G05518 | 1.499094408 | 0.00235643 | 0.00661548 |
| AT2G05520 | 1.153838128 | 9.5582E-05 | 0.00035829 |
| AT2G05540 | 2.402017995 | 1.4143E-71 | 1.3089E-69 |
| AT2G05632 | -1.03086582 | 0.00109292 | 0.00330055 |
| AT2G05920 | -1.159162995 | 2.6745E-29 | 7.0675E-28 |
| AT2G05995 | -4.30296126 | 0.00155798 | 0.00454732 |
| AT2G06002 | 2.687274091 | 0.00340774 | 0.00919213 |
| AT2G06200 | -1.990775911 | 0.00860008 | 0.02099997 |
| AT2G06850 | -1.300746956 | 3.1304E-16 | 4.068E-15 |
| AT2G07671 | 1.125860408 | 1.5862E-06 | 7.8667E-06 |
| AT2G07680 | -1.498304588 | 3.3215E-11 | 2.8489E-10 |
| AT2G08986 | -3.373721073 | 0.00012054 | 0.00044318 |
| AT2G10931 | -1.07543585 | 0.00266756 | 0.00739451 |
| AT2G10940 | -3.316894562 | 8.455E-193 | 4.112E-190 |
| AT2G11310 | 3.509241783 | 0.00035506 | 0.00118951 |
| AT2G11320 | 5.945873524 | 0.00966118 | 0.02327522 |
| AT2G11891 | 3.106423667 | 1.8753E-05 | 7.9335E-05 |
| AT2G12461 | -1.600527379 | 0.0085679 | 0.02093479 |
| AT2G12462 | -1.499783557 | 4.3789E-10 | 3.3465E-09 |
| AT2G13640 | 6.152977761 | 0.00648977 | 0.01637704 |
| AT2G13820 | -1.882866782 | 6.4891E-21 | 1.1375E-19 |
| AT2G14460 | -1.311227762 | 1.3689E-07 | 7.8345E-07 |
| AT2G14510 | -1.691912888 | 0.00141179 | 0.00415859 |
| AT2G14520 | 1.034567736 | 7.5282E-07 | 3.9029E-06 |
| AT2G14660 | -1.23010545 | 3.9487E-06 | 1.8522E-05 |
| AT2G14820 | -1.110522478 | 0.00527646 | 0.01361739 |
| AT2G14878 | 1.54137149 | 2.8696E-21 | 5.1607E-20 |
| AT2G14880 | -1.182040169 | 3.6576E-19 | 5.7699E-18 |
| AT2G15050 | -1.701956159 | 1.006E-28 | 2.5578E-27 |
| AT2G15090 | -1.523656066 | 9.7656E-20 | 1.5922E-18 |
| AT2G15220 | -2.533821863 | 5.3749E-30 | 1.4609E-28 |
| AT2G15370 | -3.122628106 | 5.0433E-06 | 2.3293E-05 |
| AT2G15390 | -2.787565407 | 1.3845E-15 | 1.7246E-14 |
| AT2G15440 | -1.147882001 | 0.00124557 | 0.00371834 |
| AT2G15760 | -2.388449073 | 5.785E-08 | 3.4881E-07 |
| AT2G15780 | 6.991765337 | 0.00052394 | 0.00169683 |
| AT2G15830 | 1.372754146 | 2.3275E-09 | 1.641E-08 |
| AT2G15880 | 2.398098198 | 3.3409E-26 | 7.6904E-25 |
| AT2G15960 | 3.197158462 | 2.446E-177 | 9.475E-175 |
| AT2G15970 | 2.008030622 | 6.5584E-79 | 7.0713E-77 |
| AT2G15990 | 7.071395251 | 0.00016223 | 0.00058113 |
| AT2G16005 | -1.302910736 | 9.0513E-08 | 5.3186E-07 |
| AT2G16380 | -1.425892811 | 7.4009E-09 | 4.9407E-08 |
| AT2G16385 | -2.153148224 | 0.0005549 | 0.00178874 |
| AT2G16390 | 1.044631989 | 8.47E-07 | 4.3576E-06 |
| AT2G16430 | -1.031861282 | 9.7043E-11 | 7.9449E-10 |
| AT2G16586 | 10.29891756 | 6.184E-12 | 5.6587E-11 |
| AT2G16630 | 2.287255277 | 7.6389E-47 | 3.7072E-45 |
| AT2G16700 | 1.633254633 | 6.1443E-34 | 1.9266E-32 |
| AT2G16710 | 1.14497115 | 2.3849E-15 | 2.9059E-14 |
| AT2G16750 | -1.043587647 | 2.8322E-05 | 0.00011639 |
| AT2G16760 | -2.277325335 | 2.3425E-13 | 2.4339E-12 |
| AT2G16850 | -1.044217704 | 3.519E-11 | 3.0127E-10 |
| AT2G16980 | -3.126884027 | 4.4245E-08 | 2.7136E-07 |
| AT2G17050 | -2.065876524 | 0.00332331 | 0.00899198 |
| AT2G17060 | -2.547562724 | 0.00035897 | 0.00119977 |
| AT2G17230 | -2.860129291 | 2.8831E-33 | 8.8936E-32 |
| AT2G17450 | 1.485691534 | 2.6081E-25 | 5.7768E-24 |
| AT2G17620 | -1.686636469 | 0.00165247 | 0.00479282 |
| AT2G17630 | -1.342221368 | 2.5263E-15 | 3.0667E-14 |
| AT2G17680 | 5.930536937 | 3.8533E-06 | 1.8112E-05 |
| AT2G17700 | 1.371039726 | 8.8061E-25 | 1.9026E-23 |
| AT2G17730 | 1.427065414 | 1.9112E-16 | 2.5223E-15 |
| AT2G17740 | -6.159598185 | 0.00091653 | 0.00282251 |
| AT2G17787 | 1.09661938 | 1.3612E-07 | 7.7944E-07 |
| AT2G17840 | 2.03902983 | 1.364E-73 | 1.3324E-71 |
| AT2G18050 | 2.153797771 | 4.7534E-29 | 1.2347E-27 |
| AT2G18090 | 1.15675599 | 3.3922E-21 | 6.0863E-20 |
| AT2G18100 | 1.371682292 | 0.00050459 | 0.0016402 |
| AT2G18120 | -1.961819793 | 0.00105502 | 0.00319792 |
| AT2G18150 | -1.831785033 | 0.00012806 | 0.00046835 |
| AT2G18160 | -1.303956568 | 7.6531E-26 | 1.7303E-24 |
| AT2G18170 | 1.812327927 | 8.4337E-50 | 4.5041E-48 |
| AT2G18193 | 2.571133176 | 3.5652E-33 | 1.0953E-31 |
| AT2G18210 | -1.321509845 | 0.00117521 | 0.00352534 |
| AT2G18230 | 1.038843569 | 7.2624E-16 | 9.2019E-15 |
| AT2G18240 | 1.114227938 | 6.0984E-08 | 3.6635E-07 |
| AT2G18300 | -2.903039907 | 6.2672E-24 | 1.3155E-22 |
| AT2G18328 | -3.576348174 | 3.258E-17 | 4.5025E-16 |
| AT2G18340 | 7.596021739 | 4.3974E-22 | 8.3554E-21 |
| AT2G18470 | -1.489045873 | 0.00791509 | 0.01950865 |
| AT2G18550 | 2.959245708 | 5.0142E-16 | 6.4355E-15 |
| AT2G18560 | -1.006271772 | 0.00017168 | 0.00061231 |
| AT2G18570 | -1.460681533 | 8.228E-11 | 6.7995E-10 |
| AT2G18650 | -1.771992006 | 0.00035384 | 0.0011856 |
| AT2G18690 | -1.072147477 | 5.7442E-13 | 5.7664E-12 |
| AT2G18700 | 3.261308891 | 3.351E-139 | 8.511E-137 |
| AT2G18800 | -3.056455259 | 6.8145E-06 | 3.0882E-05 |
| AT2G18910 | -1.293222088 | 4.5972E-11 | 3.9064E-10 |
| AT2G18915 | 1.258284976 | 4.7888E-17 | 6.5547E-16 |
| AT2G18980 | -3.142072676 | 2.0822E-43 | 8.9802E-42 |
| AT2G19060 | -2.593535895 | 0.00049755 | 0.001618 |
| AT2G19110 | -1.034632302 | 3.4463E-11 | 2.9515E-10 |
| AT2G19130 | -1.132162397 | 3.2469E-06 | 1.5449E-05 |
| AT2G19350 | 1.122863001 | 4.817E-05 | 0.0001901 |
| AT2G19450 | 1.040831903 | 3.7777E-16 | 4.8841E-15 |
| AT2G19590 | -1.441287956 | 1.108E-06 | 5.6071E-06 |
| AT2G19650 | -1.47985476 | 0.00033194 | 0.00111714 |
| AT2G19670 | -1.610247378 | 1.3092E-11 | 1.1622E-10 |
| AT2G19800 | -1.569505385 | 1.3074E-12 | 1.2739E-11 |
| AT2G19810 | 2.614526899 | 2.8832E-73 | 2.8045E-71 |
| AT2G19870 | -1.006361745 | 2.7999E-12 | 2.6534E-11 |
| AT2G19970 | -2.71668317 | 8.3356E-07 | 4.2942E-06 |
| AT2G20030 | -2.631063489 | 0.00105907 | 0.00320936 |
| AT2G20515 | -1.627298101 | 6.0116E-08 | 3.6152E-07 |
| AT2G20560 | 2.210166242 | 1.9268E-53 | 1.1207E-51 |
| AT2G20562 | -3.733245801 | 7.3029E-07 | 3.7912E-06 |
| AT2G20570 | -1.22188332 | 9.1335E-28 | 2.2069E-26 |
| AT2G20590 | -1.266053026 | 0.00016494 | 0.00059018 |
| AT2G20610 | -1.262831206 | 4.2381E-28 | 1.0439E-26 |
| AT2G20720 | 3.964488434 | 0.00617462 | 0.01568564 |
| AT2G20750 | -1.567070283 | 1.4711E-06 | 7.3277E-06 |
| AT2G20835 | 2.430968643 | 1.4834E-12 | 1.4385E-11 |
| AT2G20880 | 4.376156567 | 1.6765E-15 | 2.077E-14 |
| AT2G20950 | -1.033353365 | 2.6445E-09 | 1.8548E-08 |
| AT2G20980 | -1.17818357 | 0.00012082 | 0.00044409 |
| AT2G21045 | -1.403692356 | 1.1364E-18 | 1.7399E-17 |
| AT2G21050 | -1.496066467 | 1.8047E-14 | 2.0483E-13 |
| AT2G21080 | -1.584729763 | 2.43E-06 | 1.1731E-05 |
| AT2G21100 | -1.464486563 | 5.1649E-10 | 3.9274E-09 |
| AT2G21130 | 1.951348378 | 1.5831E-58 | 1.055E-56 |
| AT2G21180 | 1.773872812 | 1.963E-14 | 2.2158E-13 |
| AT2G21330 | -1.579573232 | 5.5948E-40 | 2.1603E-38 |
| AT2G21550 | -1.461713315 | 0.00157391 | 0.00458883 |
| AT2G21590 | 1.306913118 | 2.6489E-09 | 1.8573E-08 |
| AT2G21620 | 1.772057106 | 4.1553E-71 | 3.7993E-69 |
| AT2G21650 | -2.395619049 | 5.2126E-05 | 0.00020437 |
| AT2G21820 | 4.254144694 | 1.4116E-40 | 5.5063E-39 |
| AT2G21840 | -1.693805464 | 1.3938E-06 | 6.9715E-06 |
| AT2G22010 | 1.328547091 | 3.5808E-29 | 9.3865E-28 |
| AT2G22122 | -1.975508365 | 2.6508E-05 | 0.00010963 |
| AT2G22190 | 1.278895326 | 9.2751E-21 | 1.6147E-19 |
| AT2G22200 | 2.009336103 | 5.4398E-30 | 1.475E-28 |
| AT2G22240 | 2.512450943 | 9.7776E-85 | 1.2016E-82 |
| AT2G22250 | -1.149826366 | 8.4231E-16 | 1.0614E-14 |
| AT2G22290 | -5.599843579 | 0.0057288 | 0.01465738 |
| AT2G22330 | -1.232349776 | 4.1964E-23 | 8.3848E-22 |
| AT2G00740 | 2.884017123 | 0.00379399 | 0.01012883 |
| AT2G22430 | 1.408924204 | 1.2149E-35 | 4.06E-34 |
| AT2G22470 | 3.725493836 | 1.001E-181 | 4.159E-179 |
| AT2G22500 | 1.604421271 | 4.0666E-42 | 1.6779E-40 |
| AT2G22590 | -1.632537222 | 8.9966E-47 | 4.3477E-45 |
| AT2G22620 | -1.741131162 | 1.3452E-05 | 5.8369E-05 |
| AT2G22760 | 2.671953526 | 0.00366283 | 0.0098188 |
| AT2G22770 | -1.431490621 | 6.3574E-20 | 1.0477E-18 |
| AT2G22870 | -1.266641401 | 1.0063E-09 | 7.4203E-09 |
| AT2G22890 | -1.457894778 | 2.6582E-05 | 0.0001099 |
| AT2G22920 | -2.047222083 | 6.2896E-10 | 4.7479E-09 |
| AT2G22930 | -1.867340071 | 0.00011549 | 0.00042592 |
| AT2G23010 | -1.53884929 | 6.1261E-17 | 8.3501E-16 |
| AT2G23040 | -1.03467772 | 0.00103482 | 0.00314379 |
| AT2G23050 | -1.951195388 | 3.6621E-11 | 3.1316E-10 |
| AT2G23060 | -3.223311407 | 0.00065707 | 0.00208226 |
| AT2G23120 | 1.946984574 | 1.5244E-67 | 1.2954E-65 |
| AT2G23150 | 1.573097167 | 1.0706E-26 | 2.4971E-25 |
| AT2G23170 | 2.14043127 | 1.9933E-50 | 1.0848E-48 |
| AT2G23260 | -6.69731902 | 0.00013837 | 0.00050211 |
| AT2G23270 | -6.550463924 | 1.5317E-20 | 2.6225E-19 |
| AT2G23320 | 1.352922781 | 2.7702E-26 | 6.4091E-25 |
| AT2G23330 | 1.213541878 | 1.2006E-05 | 5.2592E-05 |
| AT2G23410 | -2.5606175 | 0.00215153 | 0.00609337 |
| AT2G23450 | 1.072354651 | 6.5067E-15 | 7.6942E-14 |
| AT2G23540 | -1.401017208 | 2.0747E-14 | 2.3396E-13 |
| AT2G23560 | -2.220809947 | 3.9417E-11 | 3.3606E-10 |
| AT2G23570 | -5.557137867 | 0.00660247 | 0.01661741 |
| AT2G23600 | -2.694580886 | 5.8812E-65 | 4.6197E-63 |
| AT2G23620 | -2.785762349 | 1.0264E-05 | 4.5346E-05 |
| AT2G23630 | -4.047037838 | 1.0682E-11 | 9.5567E-11 |
| AT2G23680 | -1.962636337 | 8.7891E-11 | 7.2318E-10 |
| AT2G23690 | -2.841807255 | 1.7389E-05 | 7.3962E-05 |
| AT2G23700 | -1.009690632 | 0.00013608 | 0.00049459 |
| AT2G23770 | -1.614623585 | 2.6759E-07 | 1.4721E-06 |
| AT2G23790 | 1.489853193 | 2.5112E-25 | 5.5728E-24 |
| AT2G23810 | 1.133078048 | 1.0884E-19 | 1.7707E-18 |
| AT2G23830 | -2.994463257 | 0.0050967 | 0.01320867 |
| AT2G23840 | 1.258706507 | 9.1736E-15 | 1.0666E-13 |
| AT2G24010 | -3.240648712 | 0.00668529 | 0.01680179 |
| AT2G24100 | 1.260730889 | 1.9553E-17 | 2.7419E-16 |
| AT2G24280 | -1.016306729 | 8.0782E-16 | 1.0202E-14 |
| AT2G24500 | 1.223906588 | 2.7167E-23 | 5.4858E-22 |
| AT2G24550 | 1.369585716 | 1.3498E-18 | 2.0556E-17 |
| AT2G24580 | -1.248988916 | 1.3467E-11 | 1.195E-10 |
| AT2G24645 | -1.501766511 | 0.0002344 | 0.00081218 |
| AT2G24850 | 4.245960213 | 1.7902E-57 | 1.1527E-55 |
| AT2G24980 | -3.633405093 | 1.5451E-19 | 2.4855E-18 |
| AT2G25000 | -1.844917788 | 1.6699E-06 | 8.2548E-06 |
| AT2G25160 | -1.599200579 | 6.4537E-12 | 5.8889E-11 |
| AT2G25220 | -1.442986971 | 0.00072711 | 0.00228051 |
| AT2G25260 | -2.352458641 | 6.7398E-13 | 6.7216E-12 |
| AT2G25297 | -3.091618318 | 1.223E-07 | 7.0521E-07 |
| AT2G25460 | 1.989414044 | 1.1361E-21 | 2.1061E-20 |
| AT2G25470 | 2.429836757 | 0.00051706 | 0.00167813 |
| AT2G25480 | -1.141491863 | 3.1404E-11 | 2.6976E-10 |
| AT2G25620 | 1.568789204 | 1.2619E-46 | 6.0469E-45 |
| AT2G25625 | 3.465773005 | 1.204E-135 | 2.838E-133 |
| AT2G25780 | -6.116137189 | 0.00108865 | 0.00328811 |
| AT2G25810 | -2.374881687 | 1.593E-05 | 6.8214E-05 |
| AT2G25900 | 1.242387492 | 5.7292E-11 | 4.8146E-10 |
| AT2G26110 | -1.206321909 | 2.1679E-11 | 1.8943E-10 |
| AT2G26150 | 2.381827966 | 5.8549E-29 | 1.5139E-27 |
| AT2G26300 | 1.101215488 | 2.1942E-20 | 3.718E-19 |
| AT2G26360 | -1.593470483 | 0.00192551 | 0.00549959 |
| AT2G26420 | -2.422369221 | 5.731E-07 | 3.0101E-06 |
| AT2G26480 | -2.194247759 | 2.2547E-06 | 1.0947E-05 |
| AT2G26520 | -1.947377263 | 0.00877076 | 0.02136653 |
| AT2G26560 | -1.985540146 | 3.3189E-28 | 8.2371E-27 |
| AT2G26570 | 1.07426112 | 6.2966E-23 | 1.2472E-21 |
| AT2G26580 | -1.01943152 | 2.9727E-07 | 1.6248E-06 |
| AT2G26650 | -1.497327107 | 8.8E-18 | 1.2635E-16 |
| AT2G26690 | 1.241839895 | 1.23E-20 | 2.1155E-19 |
| AT2G26800 | 1.120950677 | 1.9832E-17 | 2.7794E-16 |
| AT2G26820 | -2.166901363 | 2.9823E-08 | 1.8677E-07 |
| AT2G26840 | -1.13659421 | 4.3864E-06 | 2.0437E-05 |
| AT2G26920 | 1.060453216 | 9.1424E-15 | 1.0635E-13 |
| AT2G26980 | 1.150958636 | 5.0468E-19 | 7.9014E-18 |
| AT2G27010 | -1.837319312 | 0.00011544 | 0.00042581 |
| AT2G27150 | 1.756993542 | 2.8564E-42 | 1.185E-40 |
| AT2G27200 | 1.398957916 | 3.244E-18 | 4.8213E-17 |
| AT2G27370 | -3.356160034 | 3.1836E-11 | 2.7337E-10 |
| AT2G27400 | -1.616239529 | 3.4257E-09 | 2.3736E-08 |
| AT2G27402 | -3.657326282 | 1.3442E-63 | 1.0276E-61 |
| AT2G27420 | -2.519899496 | 0.00020297 | 0.00071326 |
| AT2G27430 | 1.785633887 | 1.5712E-06 | 7.7992E-06 |
| AT2G27490 | 1.306074311 | 2.0997E-13 | 2.1945E-12 |
| AT2G27500 | 1.139225722 | 6.0256E-15 | 7.1475E-14 |
| AT2G27505 | 6.796591655 | 0.00126221 | 0.00376259 |
| AT2G27550 | 1.23384845 | 7.6155E-18 | 1.0983E-16 |
| AT2G27660 | -1.859810464 | 3.0283E-09 | 2.1123E-08 |
| AT2G27690 | 1.319665231 | 0.00198618 | 0.00565945 |
| AT2G27740 | -1.710113797 | 0.00019099 | 0.00067401 |
| AT2G27770 | -1.313466906 | 1.6086E-05 | 6.8842E-05 |
| AT2G27775 | -1.199609839 | 6.0963E-07 | 3.1975E-06 |
| AT2G27830 | 2.9681099 | 1.227E-119 | 2.396E-117 |
| AT2G27950 | 1.25015631 | 4.7466E-28 | 1.1654E-26 |
| AT2G27970 | -1.013881778 | 1.7796E-07 | 1.0031E-06 |
| AT2G28000 | -1.114077496 | 6.2769E-20 | 1.0352E-18 |
| AT2G28110 | -1.499625379 | 2.2331E-05 | 9.3435E-05 |
| AT2G28140 | -1.492594387 | 0.00178566 | 0.00513352 |
| AT2G28190 | -1.245270248 | 1.1368E-19 | 1.8455E-18 |
| AT2G28210 | -1.029386599 | 0.00230168 | 0.00647691 |
| AT2G28250 | -1.914501489 | 1.0161E-16 | 1.3663E-15 |
| AT2G28305 | 1.055140441 | 1.2327E-09 | 9.011E-09 |
| AT2G28320 | 1.471468809 | 6.7357E-25 | 1.4649E-23 |
| AT2G28400 | 4.058278294 | 6.14E-186 | 2.807E-183 |
| AT2G28410 | -1.054097343 | 2.8834E-07 | 1.5798E-06 |
| AT2G28460 | -3.109848605 | 0.0012923 | 0.00384329 |
| AT2G28605 | -1.042350877 | 6.9442E-10 | 5.2197E-09 |
| AT2G28660 | -1.539531037 | 7.1987E-10 | 5.3967E-09 |
| AT2G28700 | -3.585258177 | 0.00090586 | 0.00279096 |
| AT2G28760 | -1.133935792 | 7.761E-08 | 4.6018E-07 |
| AT2G28780 | 1.878334841 | 1.124E-24 | 2.4192E-23 |
| AT2G28840 | 1.073680036 | 8.387E-26 | 1.8925E-24 |
| AT2G28870 | -1.642049835 | 0.00022544 | 0.000784 |
| AT2G28910 | 1.055834587 | 6.9324E-22 | 1.3031E-20 |
| AT2G28950 | -2.466976079 | 4.8901E-79 | 5.2975E-77 |
| AT2G28960 | -2.646973618 | 5.1289E-15 | 6.1092E-14 |
| AT2G28970 | -2.235808729 | 0.00070923 | 0.00223022 |
| AT2G28990 | -2.683587198 | 6.6159E-06 | 3.0029E-05 |
| AT2G29090 | 1.374108519 | 2.359E-05 | 9.8325E-05 |
| AT2G29120 | 2.155174206 | 2.1583E-21 | 3.9216E-20 |
| AT2G29150 | -5.169418438 | 3.1242E-05 | 0.0001275 |
| AT2G29180 | -1.098092666 | 1.0708E-08 | 7.0558E-08 |
| AT2G29260 | -1.035581435 | 0.00746663 | 0.01852717 |
| AT2G29290 | -1.76136025 | 1.6193E-20 | 2.7663E-19 |
| AT2G29310 | -1.158281267 | 1.23E-09 | 8.9941E-09 |
| AT2G29320 | -1.527935577 | 3.5656E-08 | 2.2129E-07 |
| AT2G29340 | -1.141231177 | 9.5553E-11 | 7.8369E-10 |
| AT2G29370 | 2.133445932 | 0.00013958 | 0.00050613 |
| AT2G29380 | 5.126923967 | 8.6914E-06 | 3.8871E-05 |
| AT2G29440 | 1.018590611 | 1.082E-06 | 5.484E-06 |
| AT2G29450 | 1.364184618 | 4.2233E-30 | 1.1589E-28 |
| AT2G29460 | 1.353863597 | 1.0755E-22 | 2.103E-21 |
| AT2G29620 | -2.176055805 | 0.00290067 | 0.00797014 |
| AT2G29660 | -1.129449009 | 6.3337E-07 | 3.3099E-06 |
| AT2G29670 | 1.190987204 | 1.6742E-21 | 3.0566E-20 |
| AT2G29710 | -1.701801542 | 0.00070287 | 0.00221298 |
| AT2G29750 | -2.000177466 | 3.581E-13 | 3.664E-12 |
| AT2G29995 | -1.082922962 | 6.1583E-05 | 0.00023846 |
| AT2G30010 | -2.535048154 | 4.2648E-30 | 1.1675E-28 |
| AT2G30040 | 1.420826567 | 2.3679E-14 | 2.6563E-13 |
| AT2G30140 | 1.349385814 | 2.6683E-27 | 6.3008E-26 |
| AT2G30210 | -3.034156323 | 3.5091E-46 | 1.6504E-44 |
| AT2G30250 | 1.348564786 | 1.111E-13 | 1.1918E-12 |
| AT2G30400 | 2.486973531 | 3.7866E-14 | 4.1874E-13 |
| AT2G30480 | 1.110273086 | 3.1199E-06 | 1.4866E-05 |
| AT2G30550 | 2.400131281 | 1.8876E-90 | 2.553E-88 |
| AT2G30580 | 1.069678209 | 2.763E-08 | 1.7365E-07 |
| AT2G30660 | -7.70866214 | 1.5281E-06 | 7.5949E-06 |
| AT2G30670 | -2.770579981 | 0.00749072 | 0.01857484 |
| AT2G30750 | -6.653861183 | 1.905E-235 | 1.32E-232 |
| AT2G30770 | 2.406090683 | 0.00528705 | 0.0136391 |
| AT2G30830 | 3.851544749 | 0.0074976 | 0.01858989 |
| AT2G30840 | -2.469655028 | 2.2818E-11 | 1.99E-10 |
| AT2G30930 | -1.426361824 | 2.8084E-13 | 2.8969E-12 |
| AT2G31083 | -2.427485619 | 0.00308315 | 0.00839883 |
| AT2G31110 | -2.016389082 | 2.3822E-05 | 9.9241E-05 |
| AT2G31141 | 1.290360625 | 0.00717592 | 0.01789909 |
| AT2G31260 | 1.396844458 | 2.2071E-22 | 4.2466E-21 |
| AT2G31350 | 1.154244826 | 2.1695E-18 | 3.2561E-17 |
| AT2G31585 | 1.660373432 | 5.4824E-16 | 7.0127E-15 |
| AT2G31590 | 3.980281293 | 0.00114589 | 0.00344598 |
| AT2G31680 | 1.422433328 | 4.1494E-18 | 6.1192E-17 |
| AT2G31730 | -1.011597095 | 0.00235283 | 0.00660701 |
| AT2G31751 | 1.286482964 | 6.4751E-05 | 0.00024975 |
| AT2G31790 | -1.998843624 | 7.1752E-46 | 3.3335E-44 |
| AT2G31940 | 1.262403638 | 0.00166655 | 0.00482996 |
| AT2G31945 | 1.195652048 | 0.00161656 | 0.00469939 |
| AT2G31955 | 1.262375186 | 1.1559E-20 | 1.9971E-19 |
| AT2G32100 | -1.665980594 | 0.00010823 | 0.00040188 |
| AT2G32120 | 1.834619605 | 1.7124E-15 | 2.1158E-14 |
| AT2G32150 | 1.319834622 | 2.085E-13 | 2.1822E-12 |
| AT2G32179 | -2.951369031 | 0.006867 | 0.01720928 |
| AT2G32190 | 1.815041687 | 4.0734E-13 | 4.1475E-12 |
| AT2G32210 | 1.397710594 | 8.7521E-05 | 0.00033007 |
| AT2G32235 | 2.573412694 | 8.2419E-16 | 1.0403E-14 |
| AT2G32250 | 1.174564952 | 1.8412E-14 | 2.0865E-13 |
| AT2G32270 | -5.313600508 | 6.5465E-13 | 6.5374E-12 |
| AT2G32280 | -1.221615735 | 1.1454E-05 | 5.0319E-05 |
| AT2G32300 | -1.286222998 | 2.7936E-05 | 0.00011491 |
| AT2G32415 | 1.01261441 | 3.1124E-09 | 2.1667E-08 |
| AT2G32510 | 2.842759485 | 7.9818E-82 | 9.2614E-80 |
| AT2G32540 | -1.683230793 | 4.2245E-08 | 2.5979E-07 |
| AT2G32560 | -1.333779659 | 3.7012E-14 | 4.0969E-13 |
| AT2G32660 | -7.354392336 | 6.9597E-06 | 3.1521E-05 |
| AT2G32800 | 2.344944924 | 1.5069E-77 | 1.5657E-75 |
| AT2G32930 | 1.153271534 | 2.0287E-08 | 1.2978E-07 |
| AT2G32990 | -1.060190007 | 2.0203E-09 | 1.4382E-08 |
| AT2G33020 | -5.417868035 | 0.00965076 | 0.02326483 |
| AT2G33180 | -1.387400523 | 1.0854E-16 | 1.4577E-15 |
| AT2G33205 | -1.837923819 | 1.4161E-06 | 7.0752E-06 |
| AT2G33330 | -2.451253072 | 3.5533E-39 | 1.3271E-37 |
| AT2G33380 | 5.664734488 | 0 | 0 |
| AT2G33480 | -1.031327321 | 3.578E-09 | 2.4761E-08 |
| AT2G33530 | -1.202804626 | 1.6166E-13 | 1.7092E-12 |
| AT2G33590 | 2.458356755 | 1.043E-89 | 1.3861E-87 |
| AT2G33690 | 3.38244449 | 0.00322017 | 0.00874519 |
| AT2G33700 | 1.356372954 | 1.9244E-21 | 3.5051E-20 |
| AT2G33710 | -1.099829542 | 0.00023431 | 0.00081211 |
| AT2G33847 | -2.176676465 | 0.00069163 | 0.0021803 |
| AT2G34050 | -1.35706317 | 1.7252E-08 | 1.1152E-07 |
| AT2G34060 | -1.085833638 | 0.00264036 | 0.00732889 |
| AT2G34070 | 2.231816463 | 4.3282E-62 | 3.1709E-60 |
| AT2G34190 | -1.376703068 | 9.5099E-10 | 7.0326E-09 |
| AT2G34260 | -1.096550612 | 1.2858E-07 | 7.3957E-07 |
| AT2G34430 | 1.334893951 | 3.5653E-39 | 1.3295E-37 |
| AT2G34490 | -1.186391145 | 1.1878E-12 | 1.1643E-11 |
| AT2G34510 | -2.121517298 | 2.4219E-28 | 6.0437E-27 |
| AT2G34720 | 1.032980147 | 1.3537E-12 | 1.3172E-11 |
| AT2G34790 | 1.387397505 | 1.4333E-10 | 1.1524E-09 |
| AT2G34810 | 2.850507057 | 1.428E-13 | 1.516E-12 |
| AT2G34830 | -1.181019881 | 0.00035511 | 0.00118951 |
| AT2G34850 | 2.586241149 | 2.9971E-26 | 6.9129E-25 |
| AT2G34910 | -1.656054226 | 0.00622001 | 0.01578867 |
| AT2G34960 | -1.803910993 | 0.00172624 | 0.00498211 |
| AT2G35000 | -2.070613716 | 3.7796E-08 | 2.3388E-07 |
| AT2G35060 | 1.612481926 | 7.8751E-42 | 3.2087E-40 |
| AT2G35070 | 5.736152399 | 9.9316E-23 | 1.9436E-21 |
| AT2G35130 | -1.041572583 | 3.6859E-09 | 2.547E-08 |
| AT2G35150 | -2.374218188 | 9.5487E-07 | 4.872E-06 |
| AT2G35155 | -1.062013238 | 2.1547E-09 | 1.5275E-08 |
| AT2G35290 | 2.726523381 | 7.4247E-09 | 4.9552E-08 |
| AT2G35380 | -2.969720198 | 2.5146E-19 | 4.0083E-18 |
| AT2G35630 | 1.193069003 | 1.9865E-19 | 3.1819E-18 |
| AT2G35690 | 1.251833539 | 6.1405E-16 | 7.8326E-15 |
| AT2G35730 | 1.839766892 | 5.7861E-05 | 0.00022523 |
| AT2G00880 | 1.565705382 | 0.00045645 | 0.00149392 |
| AT2G08760 | 1.758131 | 0.00389115 | 0.01036279 |
| AT2G35760 | 1.325962603 | 5.1685E-12 | 4.7638E-11 |
| AT2G35770 | -2.074550758 | 1.3403E-10 | 1.0803E-09 |
| AT2G35860 | -1.613140739 | 3.9783E-05 | 0.00015954 |
| AT2G35930 | -1.49278664 | 6.0838E-10 | 4.5987E-09 |
| AT2G35940 | 1.433448506 | 2.8864E-37 | 1.0277E-35 |
| AT2G35950 | 3.103159525 | 2.8855E-18 | 4.2969E-17 |
| AT2G35980 | -3.339671249 | 4.4676E-26 | 1.0192E-24 |
| AT2G36090 | -1.254555456 | 0.00014121 | 0.00051147 |
| AT2G36100 | -1.988530058 | 9.8394E-15 | 1.1405E-13 |
| AT2G36120 | -1.247746218 | 7.0575E-28 | 1.7144E-26 |
| AT2G36220 | 1.359029864 | 0.00043255 | 0.00142241 |
| AT2G36270 | 2.812293023 | 1.8732E-62 | 1.3947E-60 |
| AT2G36295 | -1.859313911 | 9.2573E-05 | 0.00034775 |
| AT2G36430 | -1.509797562 | 9.6464E-12 | 8.6708E-11 |
| AT2G36570 | -1.362046126 | 2.8528E-17 | 3.9568E-16 |
| AT2G36590 | 2.488539003 | 1.5344E-67 | 1.299E-65 |
| AT2G36630 | 3.567719732 | 1.269E-148 | 3.626E-146 |
| AT2G36640 | 4.645077164 | 2.8519E-11 | 2.46E-10 |
| AT2G36750 | 2.439955969 | 7.2251E-45 | 3.2638E-43 |
| AT2G36770 | 3.185871359 | 2.119E-41 | 8.4532E-40 |
| AT2G36780 | 6.179745864 | 1.0161E-48 | 5.2786E-47 |
| AT2G36810 | 1.339131901 | 2.3073E-27 | 5.4711E-26 |
| AT2G36815 | 3.566867583 | 0.00712017 | 0.01777553 |
| AT2G36830 | 1.758742389 | 9.7687E-61 | 6.8705E-59 |
| AT2G36840 | 1.035453446 | 1.2254E-05 | 5.3538E-05 |
| AT2G36870 | -1.091321464 | 1.5023E-08 | 9.7585E-08 |
| AT2G36880 | -1.223478977 | 7.0517E-39 | 2.5998E-37 |
| AT2G36885 | -1.034140589 | 1.2915E-12 | 1.2589E-11 |
| AT2G36895 | 1.382021363 | 2.8008E-25 | 6.1916E-24 |
| AT2G36900 | 1.205898317 | 2.6314E-12 | 2.4989E-11 |
| AT2G36970 | -1.217435407 | 3.2344E-08 | 2.0167E-07 |
| AT2G37040 | -1.408995802 | 2.5115E-54 | 1.4911E-52 |
| AT2G37110 | 1.061754468 | 1.6616E-23 | 3.4155E-22 |
| AT2G37130 | -2.011739477 | 3.1481E-06 | 1.4998E-05 |
| AT2G37150 | 2.167040621 | 3.2937E-44 | 1.4591E-42 |
| AT2G37170 | 1.227331812 | 4.9887E-22 | 9.4553E-21 |
| AT2G37180 | 2.3533688 | 5.9161E-16 | 7.5547E-15 |
| AT2G37380 | -2.076061896 | 4.8995E-08 | 2.9801E-07 |
| AT2G37430 | -1.629748407 | 0.00037851 | 0.00125994 |
| AT2G37460 | -1.866230901 | 4.5775E-09 | 3.1234E-08 |
| AT2G37510 | -1.2784848 | 0.00032163 | 0.00108579 |
| AT2G37570 | 1.08290325 | 1.1205E-18 | 1.7178E-17 |
| AT2G37600 | -1.069078255 | 2.2881E-11 | 1.9947E-10 |
| AT2G37640 | -1.09627849 | 8.6649E-13 | 8.5815E-12 |
| AT2G37770 | 1.779059336 | 9.2575E-08 | 5.4314E-07 |
| AT2G37870 | 3.571846829 | 5.9886E-20 | 9.8979E-19 |
| AT2G37880 | 1.652445866 | 0.00172915 | 0.0049899 |
| AT2G37900 | 3.277718908 | 2.8339E-14 | 3.1553E-13 |
| AT2G38000 | 1.11489413 | 1.7684E-11 | 1.5553E-10 |
| AT2G38060 | -1.958942055 | 9.0055E-07 | 4.6113E-06 |
| AT2G38110 | -1.292687529 | 1.5099E-07 | 8.598E-07 |
| AT2G38120 | -2.375227563 | 5.0012E-63 | 3.7979E-61 |
| AT2G38210 | -2.342511315 | 1.58E-17 | 2.232E-16 |
| AT2G38230 | -1.830663587 | 5.3462E-41 | 2.1106E-39 |
| AT2G38240 | 3.290460966 | 5.4022E-83 | 6.3981E-81 |
| AT2G38250 | 2.28704207 | 1.4616E-15 | 1.8177E-14 |
| AT2G38310 | -2.514803638 | 3.971E-45 | 1.819E-43 |
| AT2G38320 | -1.234302084 | 0.00113123 | 0.00340637 |
| AT2G38400 | 2.353199812 | 3.8087E-73 | 3.6734E-71 |
| AT2G38410 | 1.305210376 | 5.8094E-19 | 9.0519E-18 |
| AT2G38460 | -1.067707438 | 1.5325E-05 | 6.5859E-05 |
| AT2G38490 | 1.76526471 | 0.00133779 | 0.00396258 |
| AT2G38600 | -4.905202854 | 0.00012175 | 0.00044714 |
| AT2G38640 | 1.741598067 | 2.8968E-11 | 2.4968E-10 |
| AT2G38720 | -1.043349739 | 0.00552803 | 0.0142073 |
| AT2G38820 | 2.08830169 | 3.8425E-29 | 1.0049E-27 |
| AT2G38860 | -1.910013702 | 2.5035E-60 | 1.7394E-58 |
| AT2G38870 | 1.249146975 | 1.0772E-29 | 2.8865E-28 |
| AT2G38940 | -2.96783517 | 1.1477E-22 | 2.2404E-21 |
| AT2G39010 | 1.145517298 | 1.4172E-29 | 3.7799E-28 |
| AT2G39030 | 4.6931876 | 1.8804E-23 | 3.8378E-22 |
| AT2G39040 | -5.197248873 | 5.3235E-05 | 0.00020835 |
| AT2G39050 | 2.145526322 | 6.7638E-70 | 6.0158E-68 |
| AT2G39180 | -1.517978573 | 1.868E-05 | 7.9056E-05 |
| AT2G39220 | -1.18735461 | 1.3397E-09 | 9.7659E-09 |
| AT2G39310 | -1.21573174 | 3.4764E-25 | 7.6408E-24 |
| AT2G39350 | 2.782795493 | 2.078E-125 | 4.358E-123 |
| AT2G39370 | -2.1762338 | 0.00010075 | 0.00037611 |
| AT2G39410 | -1.485861366 | 0.00295995 | 0.00811156 |
| AT2G39430 | -3.7961065 | 5.3665E-20 | 8.9082E-19 |
| AT2G39450 | 1.344565667 | 8.1203E-31 | 2.2887E-29 |
| AT2G39518 | -1.527819075 | 3.8341E-06 | 1.8026E-05 |
| AT2G39530 | -2.904702283 | 1.586E-17 | 2.2392E-16 |
| AT2G39700 | -1.753101647 | 4.6349E-27 | 1.0922E-25 |
| AT2G39800 | 3.543891669 | 1.106E-255 | 1.054E-252 |
| AT2G39880 | -1.364765881 | 0.00166107 | 0.0048153 |
| AT2G39980 | 2.382351648 | 4.0662E-66 | 3.2727E-64 |
| AT2G40000 | 2.573831658 | 6.7014E-11 | 5.5864E-10 |
| AT2G40020 | -1.004148086 | 3.7221E-06 | 1.7535E-05 |
| AT2G40113 | -3.28454448 | 1.487E-14 | 1.7003E-13 |
| AT2G40150 | -1.628088521 | 2.1732E-08 | 1.3841E-07 |
| AT2G40180 | 1.689679294 | 0.00015966 | 0.00057257 |
| AT2G40340 | 3.806602883 | 2.002E-146 | 5.649E-144 |
| AT2G40390 | -1.229035019 | 0.00174284 | 0.00502811 |
| AT2G40460 | -3.113764341 | 4.8543E-58 | 3.1885E-56 |
| AT2G40610 | -7.083037669 | 4.7645E-05 | 0.00018826 |
| AT2G40710 | 2.127782497 | 0.00174873 | 0.0050432 |
| AT2G40740 | -3.167237613 | 0.00967619 | 0.023304 |
| AT2G40810 | 1.145190265 | 5.8893E-06 | 2.6934E-05 |
| AT2G40850 | 1.013052049 | 0.00280457 | 0.00773211 |
| AT2G40880 | 1.079824328 | 9.9956E-13 | 9.844E-12 |
| AT2G40925 | 6.756976744 | 0.00061192 | 0.0019538 |
| AT2G41070 | 1.352910827 | 7.9381E-07 | 4.1032E-06 |
| AT2G41090 | -2.896754005 | 9.7495E-61 | 6.8705E-59 |
| AT2G41170 | 2.533891272 | 2.8792E-64 | 2.2385E-62 |
| AT2G41190 | 5.298204413 | 2.951E-300 | 3.372E-297 |
| AT2G41200 | 1.131588718 | 3.4511E-06 | 1.6353E-05 |
| AT2G41210 | 2.090411905 | 9.634E-17 | 1.2984E-15 |
| AT2G41230 | 3.506413853 | 9.5884E-05 | 0.00035924 |
| AT2G41240 | 3.520471177 | 7.6933E-12 | 6.9618E-11 |
| AT2G41260 | -2.921843827 | 0.0088077 | 0.02144509 |
| AT2G41280 | 3.706764064 | 1.8381E-05 | 7.7878E-05 |
| AT2G41312 | 1.460512006 | 3.9434E-12 | 3.6791E-11 |
| AT2G41380 | -1.2049653 | 4.3347E-14 | 4.7796E-13 |
| AT2G41410 | 1.50995126 | 1.8055E-51 | 9.993E-50 |
| AT2G41430 | 1.141925997 | 3.5772E-33 | 1.0975E-31 |
| AT2G41480 | -1.685548587 | 4.9314E-05 | 0.00019402 |
| AT2G41500 | 1.176813505 | 9.4677E-18 | 1.3551E-16 |
| AT2G41630 | 1.405766975 | 3.5472E-32 | 1.0476E-30 |
| AT2G41705 | 1.097583146 | 5.59E-12 | 5.1336E-11 |
| AT2G41730 | 1.586587091 | 1.618E-07 | 9.1729E-07 |
| AT2G41780 | -1.340911559 | 0.00020621 | 0.00072326 |
| AT2G41800 | -1.126051435 | 3.9863E-06 | 1.8691E-05 |
| AT2G41810 | -2.450606327 | 0.0059857 | 0.015243 |
| AT2G41820 | -1.226130546 | 6.1654E-16 | 7.8555E-15 |
| AT2G41870 | 3.14919691 | 8.496E-136 | 2.023E-133 |
| AT2G41880 | 1.273524797 | 1.2563E-11 | 1.1178E-10 |
| AT2G41900 | 1.227726698 | 7.14E-32 | 2.0844E-30 |
| AT2G41940 | -1.382991024 | 6.2266E-09 | 4.1836E-08 |
| AT2G41950 | -2.293702298 | 4.4972E-28 | 1.1053E-26 |
| AT2G41970 | -2.937862472 | 1.4517E-06 | 7.2422E-06 |
| AT2G41990 | -2.212311302 | 5.8589E-05 | 0.00022776 |
| AT2G42060 | -2.809641442 | 1.0653E-09 | 7.8271E-09 |
| AT2G42160 | 1.090761963 | 1.1766E-10 | 9.5473E-10 |
| AT2G42170 | -1.395200904 | 9.7907E-06 | 4.3447E-05 |
| AT2G42190 | -1.041531786 | 3.3559E-10 | 2.5933E-09 |
| AT2G42270 | 1.77917504 | 6.1348E-32 | 1.7978E-30 |
| AT2G42330 | 1.207811222 | 1.0186E-11 | 9.138E-11 |
| AT2G42350 | -2.484154371 | 4.3099E-12 | 4.0015E-11 |
| AT2G42380 | -2.84998042 | 3.0445E-10 | 2.3686E-09 |
| AT2G42485 | -1.168386923 | 0.00056457 | 0.00181582 |
| AT2G42530 | 1.391930757 | 3.0535E-06 | 1.4584E-05 |
| AT2G42540 | 1.964887375 | 2.1863E-10 | 1.7245E-09 |
| AT2G42560 | 4.172469541 | 4.0908E-09 | 2.8114E-08 |
| AT2G42610 | -1.373113787 | 4.8727E-05 | 0.00019204 |
| AT2G42660 | -3.401837997 | 7.4856E-05 | 0.0002858 |
| AT2G42690 | -1.66531496 | 1.9198E-29 | 5.1026E-28 |
| AT2G42750 | 1.382088817 | 5.6814E-30 | 1.5369E-28 |
| AT2G42790 | 2.076976383 | 2.197E-80 | 2.4985E-78 |
| AT2G42800 | -1.579099308 | 2.5978E-05 | 0.00010759 |
| AT2G42850 | -4.821453037 | 0.00018911 | 0.00066792 |
| AT2G42870 | -1.462639693 | 0.00122296 | 0.00365368 |
| AT2G43000 | -3.374245373 | 7.0922E-29 | 1.8195E-27 |
| AT2G43020 | 2.506791854 | 5.657E-119 | 1.078E-116 |
| AT2G43100 | -1.896414213 | 2.0792E-60 | 1.449E-58 |
| AT2G43150 | -1.277900596 | 1.8419E-28 | 4.6369E-27 |
| AT2G43240 | 1.571557422 | 4.0913E-26 | 9.3518E-25 |
| AT2G43320 | 1.011356892 | 2.3507E-08 | 1.4889E-07 |
| AT2G43330 | 1.388061517 | 1.8714E-20 | 3.1851E-19 |
| AT2G43360 | -1.446213867 | 4.1782E-30 | 1.1479E-28 |
| AT2G43390 | -2.580550557 | 0.00015366 | 0.00055306 |
| AT2G43465 | -1.081585287 | 0.0023723 | 0.00665839 |
| AT2G43500 | 1.006033586 | 4.7315E-11 | 4.0146E-10 |
| AT2G43510 | -1.132985697 | 4.7401E-10 | 3.6165E-09 |
| AT2G43530 | -1.226035919 | 3.7933E-21 | 6.774E-20 |
| AT2G43550 | -1.90139021 | 6.263E-46 | 2.9157E-44 |
| AT2G43590 | -1.165629237 | 3.987E-24 | 8.4462E-23 |
| AT2G43610 | -1.810935417 | 7.8627E-24 | 1.6428E-22 |
| AT2G43620 | 1.926507382 | 0.00335194 | 0.00905658 |
| AT2G43820 | 3.061211802 | 8.19E-132 | 1.835E-129 |
| AT2G43840 | -1.361080302 | 0.00593794 | 0.01513655 |
| AT2G43870 | -1.803280691 | 0.00232867 | 0.00654398 |
| AT2G43880 | -2.354025349 | 1.4138E-06 | 7.0655E-06 |
| AT2G43910 | -1.686543502 | 1.2434E-53 | 7.2688E-52 |
| AT2G44010 | -1.805670509 | 6.8922E-06 | 3.1221E-05 |
| AT2G44060 | 1.01566239 | 3.3968E-19 | 5.3734E-18 |
| AT2G44080 | 1.214783883 | 8.6897E-07 | 4.4646E-06 |
| AT2G44110 | -1.677505574 | 0.0034682 | 0.00933656 |
| AT2G44195 | 3.715955647 | 0.000518 | 0.00168044 |
| AT2G44210 | -1.13134978 | 1.3096E-14 | 1.5027E-13 |
| AT2G44230 | -1.294796348 | 2.5844E-11 | 2.2411E-10 |
| AT2G44370 | -1.919372007 | 8.5868E-10 | 6.3892E-09 |
| AT2G44380 | -1.977228501 | 2.328E-05 | 9.7141E-05 |
| AT2G44460 | 2.941780104 | 0.00358268 | 0.00961749 |
| AT2G44578 | -1.72637224 | 0.00086567 | 0.00267797 |
| AT2G44580 | -1.462573985 | 1.1034E-05 | 4.8539E-05 |
| AT2G44740 | -2.634267887 | 9.4258E-05 | 0.00035361 |
| AT2G44790 | -1.36268369 | 3.7141E-26 | 8.5237E-25 |
| AT2G44810 | 6.688079418 | 0.00078952 | 0.0024617 |
| AT2G44840 | 1.842337079 | 0.00609027 | 0.01548686 |
| AT2G44940 | -1.803071843 | 2.3957E-22 | 4.6018E-21 |
| AT2G44970 | 1.404670308 | 5.649E-32 | 1.6576E-30 |
| AT2G45180 | -3.494303825 | 1.411E-220 | 8.489E-218 |
| AT2G45210 | 1.195812442 | 1.1895E-06 | 5.9913E-06 |
| AT2G45470 | -1.505751278 | 1.0501E-44 | 4.7251E-43 |
| AT2G45490 | 1.437015135 | 2.9516E-06 | 1.4126E-05 |
| AT2G45570 | 5.319120292 | 2.12E-165 | 7.234E-163 |
| AT2G45580 | 3.466783385 | 0.00207897 | 0.00590617 |
| AT2G45600 | 1.17330578 | 2.279E-08 | 1.4479E-07 |
| AT2G45610 | 6.695870224 | 0.00074896 | 0.00234292 |
| AT2G45660 | 1.404408872 | 7.8895E-20 | 1.2946E-18 |
| AT2G45670 | 1.067238382 | 8.0642E-22 | 1.5084E-20 |
| AT2G45750 | -1.732716955 | 2.6027E-09 | 1.8277E-08 |
| AT2G45760 | -2.523669801 | 2.8951E-07 | 1.5855E-06 |
| AT2G45820 | 1.291293552 | 5.4734E-34 | 1.7233E-32 |
| AT2G45890 | -1.054572495 | 0.00467951 | 0.01223706 |
| AT2G45910 | 1.420515327 | 6.7957E-22 | 1.2785E-20 |
| AT2G45980 | 1.301694833 | 1.0978E-26 | 2.5579E-25 |
| AT2G46240 | 1.379680504 | 2.4412E-16 | 3.1996E-15 |
| AT2G46260 | 1.197154217 | 2.1145E-22 | 4.0788E-21 |
| AT2G46270 | 2.879659336 | 3.319E-136 | 7.987E-134 |
| AT2G46330 | -1.076922576 | 1.3275E-10 | 1.0703E-09 |
| AT2G46370 | 1.137366134 | 3.8482E-25 | 8.4499E-24 |
| AT2G46430 | -1.548376587 | 1.0209E-11 | 9.1512E-11 |
| AT2G46490 | 1.156406191 | 4.6499E-16 | 5.9813E-15 |
| AT2G46495 | -4.479750612 | 9.4762E-23 | 1.8577E-21 |
| AT2G46510 | 1.507704056 | 6.5051E-29 | 1.6783E-27 |
| AT2G46535 | -1.522201408 | 4.0039E-13 | 4.0803E-12 |
| AT2G46650 | -2.132896662 | 8.0807E-49 | 4.2075E-47 |
| AT2G46660 | -1.186784997 | 0.00016916 | 0.00060415 |
| AT2G46680 | 3.987741913 | 0 | 0 |
| AT2G46720 | 1.322037735 | 2.3304E-08 | 1.4768E-07 |
| AT2G46740 | -1.645452944 | 5.6232E-11 | 4.736E-10 |
| AT2G46750 | -2.296620421 | 2.9014E-39 | 1.0908E-37 |
| AT2G46780 | -1.06124232 | 0.0005499 | 0.00177361 |
| AT2G46790 | 1.788950595 | 0.00069464 | 0.00218916 |
| AT2G46860 | -5.419916064 | 7.0232E-06 | 3.177E-05 |
| AT2G46870 | -1.910466348 | 4.2551E-20 | 7.1099E-19 |
| AT2G46890 | -1.328962834 | 1.568E-08 | 1.0168E-07 |
| AT2G46970 | -1.854556107 | 0.00772008 | 0.01907735 |
| AT2G47180 | 2.081530789 | 4.9158E-50 | 2.6501E-48 |
| AT2G47190 | 4.009410411 | 5.6777E-08 | 3.4252E-07 |
| AT2G47360 | -3.233016221 | 1.2524E-12 | 1.2234E-11 |
| AT2G47370 | -1.967534459 | 4.5386E-09 | 3.0987E-08 |
| AT2G47410 | 1.275220875 | 5.8886E-22 | 1.1115E-20 |
| AT2G47440 | -1.143053865 | 2.0638E-10 | 1.6351E-09 |
| AT2G47485 | 2.254258622 | 4.3726E-50 | 2.3685E-48 |
| AT2G47520 | 2.475708626 | 1.8562E-08 | 1.1941E-07 |
| AT2G47530 | -4.321755597 | 0.00221382 | 0.00625506 |
| AT2G47540 | -7.685838012 | 6.2898E-13 | 6.292E-12 |
| AT2G47550 | -1.203741361 | 0.00773585 | 0.01911221 |
| AT2G47600 | 1.095274575 | 8.9285E-18 | 1.2804E-16 |
| AT2G47700 | 1.145750109 | 4.5394E-21 | 8.0811E-20 |
| AT2G47730 | 1.103732468 | 1.5365E-28 | 3.8808E-27 |
| AT2G47750 | -2.234262302 | 0.00075988 | 0.00237417 |
| AT2G47770 | 6.422042422 | 2.1757E-19 | 3.4753E-18 |
| AT2G47800 | 1.477264122 | 3.9942E-39 | 1.4846E-37 |
| AT2G47820 | 1.159309375 | 2.3866E-06 | 1.1543E-05 |
| AT2G47860 | -1.311489748 | 4.565E-08 | 2.7923E-07 |
| AT2G47910 | -1.519407218 | 1.1307E-27 | 2.7176E-26 |
| AT2G47930 | -2.25801317 | 0.00010829 | 0.00040198 |
| AT2G47950 | 2.258760478 | 1.2559E-06 | 6.3118E-06 |
| AT2G48080 | -1.681388686 | 2.7043E-05 | 0.00011154 |
| AT2G48090 | -1.177670374 | 0.00115158 | 0.00345987 |
| AT2G48130 | -1.197814932 | 1.2382E-11 | 1.1022E-10 |
| AT3G01060 | -1.307094322 | 1.974E-08 | 1.265E-07 |
| AT3G01070 | -2.236608939 | 0.00559338 | 0.01435427 |
| AT3G01100 | 1.061690254 | 3.9991E-14 | 4.4203E-13 |
| AT3G01175 | -2.371152239 | 0.00156689 | 0.00457012 |
| AT3G01190 | -2.156492352 | 9.4804E-24 | 1.9736E-22 |
| AT3G01220 | -1.852724681 | 9.7048E-08 | 5.6793E-07 |
| AT3G01260 | -1.723341 | 0.00307527 | 0.00838337 |
| AT3G01290 | -1.434370188 | 5.5497E-27 | 1.3064E-25 |
| AT3G01320 | 1.131991967 | 1.1355E-17 | 1.6132E-16 |
| AT3G01420 | -2.900123441 | 2.423E-126 | 5.275E-124 |
| AT3G01440 | -2.035334265 | 7.1785E-45 | 3.2492E-43 |
| AT3G01500 | -1.004035501 | 9.7595E-28 | 2.3532E-26 |
| AT3G01516 | -1.548895882 | 0.00425353 | 0.01123363 |
| AT3G01540 | 1.43609123 | 2.3342E-53 | 1.3542E-51 |
| AT3G01590 | 1.570504688 | 4.1795E-39 | 1.5484E-37 |
| AT3G01640 | 1.087433776 | 3.0996E-12 | 2.9241E-11 |
| AT3G01650 | 1.836201498 | 4.1772E-39 | 1.5484E-37 |
| AT3G01770 | 1.083177292 | 1.2904E-15 | 1.6109E-14 |
| AT3G01860 | -1.338738936 | 1.6745E-10 | 1.3379E-09 |
| AT3G02020 | -1.99779441 | 7.0614E-57 | 4.4712E-55 |
| AT3G02040 | -1.740557751 | 3.7841E-09 | 2.61E-08 |
| AT3G02070 | 1.012706973 | 4.3554E-08 | 2.6755E-07 |
| AT3G02120 | -1.185598239 | 7.9384E-08 | 4.6948E-07 |
| AT3G02140 | 2.978438751 | 2.071E-118 | 3.913E-116 |
| AT3G02150 | 2.073490006 | 3.3859E-36 | 1.1534E-34 |
| AT3G02160 | 1.035483958 | 0.00011349 | 0.00041948 |
| AT3G02240 | -1.687184155 | 0.00836898 | 0.02049916 |
| AT3G02260 | 1.490858028 | 8.2288E-40 | 3.1507E-38 |
| AT3G02410 | 2.158719262 | 0.00018531 | 0.0006565 |
| AT3G02480 | 5.835772818 | 1.7611E-10 | 1.4031E-09 |
| AT3G02515 | 3.04775561 | 0.00735167 | 0.01828557 |
| AT3G02680 | 1.014223098 | 0.00021916 | 0.00076366 |
| AT3G02800 | 1.167067096 | 1.2051E-05 | 5.2762E-05 |
| AT3G02875 | 2.412336669 | 7.6614E-60 | 5.259E-58 |
| AT3G02885 | -3.176116421 | 1.2384E-10 | 1.0027E-09 |
| AT3G02990 | 2.935777807 | 1.074E-70 | 9.7032E-69 |
| AT3G03020 | 1.065922323 | 3.0856E-05 | 0.00012604 |
| AT3G03060 | -1.309999636 | 1.8077E-14 | 2.0507E-13 |
| AT3G03130 | -1.118133005 | 4.5291E-08 | 2.7718E-07 |
| AT3G03170 | 2.199402582 | 8.296E-21 | 1.4487E-19 |
| AT3G03310 | 1.552238466 | 1.1769E-24 | 2.5307E-23 |
| AT3G03341 | 5.630505018 | 1.4074E-17 | 1.9932E-16 |
| AT3G03440 | 2.228102251 | 4.7019E-55 | 2.8433E-53 |
| AT3G03470 | 3.937508066 | 2.785E-141 | 7.153E-139 |
| AT3G03490 | 1.163001303 | 5.6021E-12 | 5.1427E-11 |
| AT3G03620 | 2.18683869 | 0.00831247 | 0.02037384 |
| AT3G03640 | 1.516320735 | 3.98E-35 | 1.3076E-33 |
| AT3G03650 | -2.045726574 | 0.00687513 | 0.017224 |
| AT3G03660 | -3.123111813 | 0.0048521 | 0.01264645 |
| AT3G03670 | -3.766833323 | 4.8042E-05 | 0.00018966 |
| AT3G03780 | -2.010304246 | 1.6699E-81 | 1.9181E-79 |
| AT3G03840 | -5.897281085 | 0.00251714 | 0.00702012 |
| AT3G03870 | 1.174239401 | 6.2067E-10 | 4.6869E-09 |
| AT3G03900 | 1.423828391 | 1.9489E-14 | 2.201E-13 |
| AT3G03910 | -3.225928437 | 0.00066168 | 0.00209455 |
| AT3G04000 | 2.195315968 | 2.2615E-24 | 4.8176E-23 |
| AT3G04010 | 3.14441185 | 2.309E-118 | 4.326E-116 |
| AT3G04040 | 1.42984687 | 3.2587E-06 | 1.5499E-05 |
| AT3G04140 | -1.711028527 | 2.3704E-13 | 2.4606E-12 |
| AT3G04240 | 2.071957425 | 2.7908E-90 | 3.7525E-88 |
| AT3G04290 | -1.876721371 | 9.8333E-47 | 4.742E-45 |
| AT3G04370 | -1.591704079 | 0.00455334 | 0.01193854 |
| AT3G04410 | 6.288584393 | 0.00330994 | 0.00896748 |
| AT3G01455 | 4.962551505 | 0.00090555 | 0.00279038 |
| AT3G04550 | -1.150278978 | 1.3072E-18 | 1.992E-17 |
| AT3G04570 | -1.587847442 | 3.4247E-12 | 3.2122E-11 |
| AT3G04620 | 3.985994241 | 3.319E-133 | 7.586E-131 |
| AT3G04640 | 1.389158499 | 4.0366E-09 | 2.7775E-08 |
| AT3G04770 | -1.07429052 | 1.2587E-08 | 8.2415E-08 |
| AT3G04790 | -1.04585733 | 5.0991E-15 | 6.077E-14 |
| AT3G04854 | 1.85312382 | 0.00025028 | 0.00086262 |
| AT3G05140 | -2.40545069 | 0.00044949 | 0.00147325 |
| AT3G05345 | 1.069441292 | 5.2048E-10 | 3.9552E-09 |
| AT3G05380 | 1.080134198 | 7.0871E-14 | 7.6994E-13 |
| AT3G05500 | 1.041367475 | 2.7798E-15 | 3.3708E-14 |
| AT3G05580 | 1.247964639 | 1.2107E-14 | 1.3935E-13 |
| AT3G05625 | -1.312115091 | 1.296E-13 | 1.3824E-12 |
| AT3G05630 | 3.669711288 | 2.9531E-72 | 2.8125E-70 |
| AT3G05640 | 3.850079687 | 1.592E-180 | 6.383E-178 |
| AT3G05650 | 2.093858934 | 2.2263E-15 | 2.7228E-14 |
| AT3G05660 | 1.921358816 | 1.8544E-15 | 2.2838E-14 |
| AT3G05670 | 1.026372389 | 2.8322E-16 | 3.6952E-15 |
| AT3G05740 | -1.140554465 | 0.00269069 | 0.00744869 |
| AT3G05790 | 1.604439584 | 0.00065863 | 0.00208691 |
| AT3G05820 | 2.007515134 | 1.2159E-06 | 6.1165E-06 |
| AT3G05880 | 1.407180778 | 4.4669E-45 | 2.0299E-43 |
| AT3G01795 | 1.883458754 | 5.4294E-14 | 5.9609E-13 |
| AT3G05920 | -2.797103347 | 2.7182E-10 | 2.1227E-09 |
| AT3G05936 | 1.485198573 | 3.5254E-06 | 1.6674E-05 |
| AT3G06070 | -1.066883478 | 0.00873652 | 0.02129219 |
| AT3G06140 | -1.028144136 | 0.00046057 | 0.00150632 |
| AT3G06145 | -3.451619751 | 3.7109E-12 | 3.465E-11 |
| AT3G06160 | -1.32259896 | 0.00106791 | 0.00323272 |
| AT3G06370 | -1.215159371 | 8.3507E-05 | 0.00031613 |
| AT3G06420 | 1.970833824 | 1.8036E-47 | 8.9427E-46 |
| AT3G06490 | 2.232045682 | 2.8936E-12 | 2.7377E-11 |
| AT3G06500 | 3.435554122 | 1.606E-281 | 1.668E-278 |
| AT3G06620 | 1.391413483 | 3.8875E-26 | 8.9039E-25 |
| AT3G06740 | -1.318521343 | 2.1428E-07 | 1.1973E-06 |
| AT3G06750 | -1.916330118 | 1.1516E-13 | 1.2341E-12 |
| AT3G06760 | 1.3126047 | 3.3665E-17 | 4.6497E-16 |
| AT3G06770 | -1.666461569 | 7.4346E-13 | 7.3983E-12 |
| AT3G06778 | -1.15142102 | 0.00126465 | 0.00376839 |
| AT3G06780 | 1.916667015 | 8.8823E-19 | 1.3681E-17 |
| AT3G06840 | -2.183426056 | 4.5534E-11 | 3.8707E-10 |
| AT3G06868 | -2.176142233 | 0.00024286 | 0.00083932 |
| AT3G06880 | -1.329766545 | 1.886E-18 | 2.8494E-17 |
| AT3G06990 | -3.371285713 | 0.00360502 | 0.00967291 |
| AT3G07000 | -3.392000755 | 0.00021746 | 0.00075853 |
| AT3G07010 | -1.599172649 | 1.4216E-12 | 1.381E-11 |
| AT3G07070 | -5.281469479 | 2.0186E-05 | 8.5053E-05 |
| AT3G07130 | -1.201506694 | 9.6269E-07 | 4.9086E-06 |
| AT3G07190 | -1.027357188 | 0.00012077 | 0.00044395 |
| AT3G07195 | -1.198267068 | 0.00168079 | 0.00486631 |
| AT3G07200 | -1.057190776 | 1.8378E-05 | 7.7878E-05 |
| AT3G07273 | 1.520342292 | 1.1627E-10 | 9.4377E-10 |
| AT3G07350 | 2.573247874 | 1.3406E-44 | 6.0085E-43 |
| AT3G07360 | 1.142823985 | 2.5077E-17 | 3.4952E-16 |
| AT3G07425 | -4.410414702 | 0.00120528 | 0.00360511 |
| AT3G07460 | -1.116649151 | 3.3773E-17 | 4.6617E-16 |
| AT3G07470 | -1.316253565 | 3.4898E-20 | 5.8645E-19 |
| AT3G07540 | -1.27781039 | 1.9026E-08 | 1.222E-07 |
| AT3G07700 | 1.676014735 | 9.3938E-38 | 3.3815E-36 |
| AT3G07720 | 1.031769351 | 8.595E-18 | 1.2364E-16 |
| AT3G07870 | 1.044654137 | 6.411E-09 | 4.2999E-08 |
| AT3G07900 | -3.188212757 | 4.5613E-05 | 0.00018098 |
| AT3G08040 | -1.959222524 | 5.0328E-15 | 6.0011E-14 |
| AT3G08490 | -6.400525701 | 0.00041502 | 0.0013689 |
| AT3G00260 | -2.238461774 | 0.00777878 | 0.01920581 |
| AT3G08505 | 1.154536515 | 1.9071E-09 | 1.3606E-08 |
| AT3G08600 | -1.370862852 | 6.5451E-07 | 3.4134E-06 |
| AT3G08720 | 1.433410562 | 5.6864E-19 | 8.8724E-18 |
| AT3G08750 | 5.945873524 | 0.00966118 | 0.02327522 |
| AT3G08860 | 2.610696893 | 4.7424E-31 | 1.3466E-29 |
| AT3G08970 | 1.079442752 | 1.3431E-06 | 6.7342E-06 |
| AT3G09010 | -1.155107238 | 0.00012699 | 0.00046472 |
| AT3G09020 | -1.295367018 | 0.00307645 | 0.00838458 |
| AT3G09035 | -1.394141654 | 2.5012E-10 | 1.9593E-09 |
| AT3G09220 | -3.549841701 | 1.351E-66 | 1.0989E-64 |
| AT3G09260 | -1.149586304 | 6.6185E-35 | 2.1581E-33 |
| AT3G09270 | -1.647240594 | 1.0261E-44 | 4.6259E-43 |
| AT3G09350 | 1.676176772 | 3.0459E-41 | 1.2109E-39 |
| AT3G09370 | 1.486799103 | 8.7853E-15 | 1.0251E-13 |
| AT3G09390 | 1.263910976 | 2.063E-30 | 5.6953E-29 |
| AT3G09405 | -3.00391869 | 4.8497E-21 | 8.6134E-20 |
| AT3G09450 | 4.784068903 | 2.3256E-09 | 1.6402E-08 |
| AT3G09480 | 1.297205185 | 1.203E-05 | 5.2679E-05 |
| AT3G09560 | 1.051822704 | 7.7406E-14 | 8.3935E-13 |
| AT3G09580 | -1.357278909 | 3.9655E-13 | 4.0448E-12 |
| AT3G09640 | 1.639094876 | 0.00029227 | 0.00099447 |
| AT3G09700 | -1.287099363 | 1.4409E-05 | 6.2203E-05 |
| AT3G09760 | 1.080732004 | 1.2996E-11 | 1.1546E-10 |
| AT3G09770 | 1.42245519 | 3.6581E-36 | 1.2406E-34 |
| AT3G09910 | 2.02189831 | 1.671E-11 | 1.4714E-10 |
| AT3G09920 | 1.749337181 | 2.3121E-47 | 1.1415E-45 |
| AT3G09925 | -2.932745604 | 2.3537E-11 | 2.0487E-10 |
| AT3G09950 | 2.223577658 | 1.5137E-05 | 6.5137E-05 |
| AT3G09960 | -3.17134695 | 4.2084E-05 | 0.00016788 |
| AT3G10020 | 1.913983872 | 2.7051E-47 | 1.3326E-45 |
| AT3G10040 | -1.225701036 | 0.00315314 | 0.00857418 |
| AT3G10080 | -1.493993282 | 1.0013E-09 | 7.3882E-09 |
| AT3G10110 | -1.080001684 | 0.00016067 | 0.00057601 |
| AT3G10185 | -3.07340799 | 4.5443E-05 | 0.00018035 |
| AT3G10190 | -1.02495076 | 0.00019779 | 0.0006963 |
| AT3G10250 | 1.0856536 | 3.1134E-10 | 2.419E-09 |
| AT3G10290 | -1.639093976 | 0.00029776 | 0.00101103 |
| AT3G10300 | 1.053938433 | 8.0578E-15 | 9.4648E-14 |
| AT3G10320 | -1.340832669 | 0.00073852 | 0.0023128 |
| AT3G10340 | 1.098891139 | 1.4153E-13 | 1.5033E-12 |
| AT3G10405 | -1.017481015 | 1.4539E-08 | 9.4598E-08 |
| AT3G10420 | 1.612631545 | 2.0903E-42 | 8.6871E-41 |
| AT3G10450 | 1.954857878 | 1.8456E-28 | 4.6411E-27 |
| AT3G10520 | -1.384001582 | 1.5981E-16 | 2.1226E-15 |
| AT3G10550 | 1.242613949 | 1.6092E-18 | 2.4359E-17 |
| AT3G10570 | -1.41304506 | 5.0607E-05 | 0.00019888 |
| AT3G10610 | -1.143777832 | 9.6117E-17 | 1.2962E-15 |
| AT3G10710 | -3.721650335 | 9.1358E-10 | 6.7691E-09 |
| AT3G10800 | 1.01300215 | 7.0836E-14 | 7.6994E-13 |
| AT3G10820 | 1.050484741 | 9.7699E-06 | 4.3363E-05 |
| AT3G10910 | 1.613040958 | 1.122E-17 | 1.5959E-16 |
| AT3G11020 | 1.352874633 | 0.00186171 | 0.00533203 |
| AT3G11090 | -1.030781923 | 6.9618E-05 | 0.00026704 |
| AT3G11110 | -1.259296599 | 0.00167403 | 0.00484857 |
| AT3G11210 | 1.217165217 | 1.115E-17 | 1.588E-16 |
| AT3G11260 | 1.138652612 | 0.00021438 | 0.00074916 |
| AT3G11340 | 1.520529981 | 3.8607E-21 | 6.889E-20 |
| AT3G11370 | -5.965523904 | 0.00291762 | 0.00801188 |
| AT3G11385 | -3.885230462 | 0.00819753 | 0.02012665 |
| AT3G11410 | 3.129598464 | 2.681E-235 | 1.803E-232 |
| AT3G11420 | 2.669027584 | 1.3251E-77 | 1.3831E-75 |
| AT3G11480 | 7.5308263 | 2.2993E-05 | 9.5997E-05 |
| AT3G11520 | -1.263148971 | 3.1395E-05 | 0.00012806 |
| AT3G11550 | -2.498396129 | 1.1262E-11 | 1.0048E-10 |
| AT3G11690 | 2.062437232 | 8.7638E-36 | 2.9416E-34 |
| AT3G11773 | 3.88313801 | 0.0039633 | 0.01053776 |
| AT3G12090 | -1.616121041 | 6.518E-11 | 5.4455E-10 |
| AT3G12110 | -1.049103789 | 7.3687E-07 | 3.8237E-06 |
| AT3G12170 | -1.986436432 | 1.22E-06 | 6.1355E-06 |
| AT3G12220 | -2.686645967 | 0.00987036 | 0.02371917 |
| AT3G12400 | 1.036038889 | 3.4282E-13 | 3.514E-12 |
| AT3G12540 | -3.225928437 | 0.00066168 | 0.00209455 |
| AT3G12580 | 1.989345909 | 3.0158E-92 | 4.1527E-90 |
| AT3G12870 | -1.102763218 | 0.00017157 | 0.00061201 |
| AT3G12930 | -1.212657027 | 9.0805E-16 | 1.1423E-14 |
| AT3G12960 | 3.275693864 | 6.2172E-06 | 2.8293E-05 |
| AT3G12977 | -2.319468472 | 6.3138E-05 | 0.00024383 |
| AT3G13000 | -1.153265393 | 1.2763E-10 | 1.0323E-09 |
| AT3G13020 | 1.036580816 | 0.00666517 | 0.01675861 |
| AT3G13080 | 1.652802711 | 1.1035E-43 | 4.8043E-42 |
| AT3G13130 | 1.981746574 | 3.0866E-06 | 1.4726E-05 |
| AT3G13275 | 1.146845593 | 3.7121E-06 | 1.7497E-05 |
| AT3G13277 | 1.902886115 | 0.00159062 | 0.00463281 |
| AT3G13330 | 1.18304437 | 5.7077E-24 | 1.2002E-22 |
| AT3G13404 | -3.080617814 | 0.00277099 | 0.00764966 |
| AT3G13420 | 1.281198524 | 4.9078E-05 | 0.00019322 |
| AT3G13432 | -7.240697935 | 1.1613E-05 | 5.0991E-05 |
| AT3G13433 | -2.934811196 | 0.00722829 | 0.01800417 |
| AT3G13470 | -1.472432439 | 2.004E-28 | 5.0283E-27 |
| AT3G13510 | -1.088410532 | 1.6453E-14 | 1.8729E-13 |
| AT3G13560 | -1.534568135 | 1.4287E-18 | 2.1699E-17 |
| AT3G13610 | -3.783902606 | 4.988E-135 | 1.163E-132 |
| AT3G13650 | -1.007624573 | 5.0859E-08 | 3.0861E-07 |
| AT3G13672 | 3.742638895 | 2.6353E-30 | 7.2664E-29 |
| AT3G13730 | -1.114132915 | 0.00010031 | 0.00037472 |
| AT3G13750 | -1.714121453 | 2.4835E-62 | 1.8312E-60 |
| AT3G13760 | -2.486273906 | 0.00497262 | 0.01291491 |
| AT3G13950 | -1.220738096 | 0.00416021 | 0.01101517 |
| AT3G14020 | -1.027073415 | 0.00150939 | 0.00442065 |
| AT3G14050 | 2.064162036 | 2.2661E-39 | 8.5618E-38 |
| AT3G14060 | 2.669278246 | 1.5373E-36 | 5.3322E-35 |
| AT3G14067 | 1.392285362 | 3.2318E-52 | 1.8331E-50 |
| AT3G14070 | 1.022702271 | 0.00013265 | 0.00048352 |
| AT3G14180 | 1.014198574 | 5.9156E-14 | 6.476E-13 |
| AT3G14210 | -1.563307297 | 7.1697E-37 | 2.5136E-35 |
| AT3G14225 | -3.721995439 | 0.00063957 | 0.00203272 |
| AT3G14240 | -1.387326072 | 1.8091E-28 | 4.5593E-27 |
| AT3G14310 | -1.472904582 | 1.2747E-46 | 6.0954E-45 |
| AT3G14360 | 1.631560654 | 5.714E-11 | 4.8036E-10 |
| AT3G14430 | 1.118762995 | 7.527E-13 | 7.487E-12 |
| AT3G14440 | 4.479921533 | 0 | 0 |
| AT3G14560 | 2.090195654 | 1.4251E-30 | 3.9582E-29 |
| AT3G14590 | 2.041221016 | 8.0575E-30 | 2.1745E-28 |
| AT3G14595 | 2.145399469 | 3.6255E-68 | 3.151E-66 |
| AT3G14620 | -1.520573746 | 4.8582E-22 | 9.2157E-21 |
| AT3G14670 | 3.194118464 | 0.00191527 | 0.00547174 |
| AT3G14680 | -1.017609035 | 2.9028E-06 | 1.391E-05 |
| AT3G14690 | 1.182355709 | 1.9405E-32 | 5.8132E-31 |
| AT3G14740 | -1.315504402 | 1.8236E-06 | 8.9683E-06 |
| AT3G14760 | -1.244223178 | 0.00059299 | 0.00189838 |
| AT3G14810 | 1.732803126 | 3.7354E-29 | 9.7804E-28 |
| AT3G14840 | -1.248570784 | 2.8893E-22 | 5.5267E-21 |
| AT3G15030 | -1.313213095 | 1.3303E-27 | 3.1841E-26 |
| AT3G15200 | 1.490149017 | 0.00012802 | 0.00046826 |
| AT3G15210 | 1.844375941 | 5.7785E-61 | 4.1148E-59 |
| AT3G00330 | 4.373463101 | 0.00598304 | 0.01523792 |
| AT3G15280 | -3.33590726 | 0.0094851 | 0.02291623 |
| AT3G15350 | 1.578888649 | 1.1582E-36 | 4.0296E-35 |
| AT3G15450 | 1.197748173 | 1.0824E-15 | 1.3564E-14 |
| AT3G15500 | 4.125954965 | 1.5461E-41 | 6.2219E-40 |
| AT3G15534 | 3.549144694 | 3.8645E-27 | 9.1161E-26 |
| AT3G15570 | -1.279385732 | 4.2824E-08 | 2.6321E-07 |
| AT3G15670 | 3.179799416 | 0.00331088 | 0.00896789 |
| AT3G15680 | -1.000752625 | 6.855E-10 | 5.1611E-09 |
| AT3G15700 | -6.137177509 | 0.0010548 | 0.00319768 |
| AT3G15720 | -1.415934164 | 0.0002432 | 0.00084025 |
| AT3G15760 | 1.40014615 | 3.7931E-06 | 1.7844E-05 |
| AT3G15780 | 1.223429136 | 1.0524E-06 | 5.3386E-06 |
| AT3G15850 | -1.199920317 | 3.3988E-17 | 4.6885E-16 |
| AT3G15880 | 1.052668479 | 1.0189E-20 | 1.7683E-19 |
| AT3G03165 | 1.697253962 | 0.0001752 | 0.0006234 |
| AT3G15950 | -1.216469132 | 3.9853E-31 | 1.133E-29 |
| AT3G16180 | -1.083416994 | 3.5469E-05 | 0.00014334 |
| AT3G16190 | 1.005072976 | 4.537E-13 | 4.601E-12 |
| AT3G16250 | -1.353456801 | 5.3512E-25 | 1.1694E-23 |
| AT3G16330 | 2.647711861 | 6.6724E-71 | 6.0764E-69 |
| AT3G16430 | -1.310120151 | 1.3304E-21 | 2.4524E-20 |
| AT3G16440 | -1.223788964 | 0.00181661 | 0.00521332 |
| AT3G16450 | -1.160144791 | 1.9368E-24 | 4.1376E-23 |
| AT3G16510 | 1.262982501 | 5.9248E-05 | 0.0002302 |
| AT3G16530 | -2.053001441 | 8.9679E-42 | 3.6475E-40 |
| AT3G16570 | 1.31178988 | 2.2263E-25 | 4.9648E-24 |
| AT3G16690 | -1.315631411 | 7.8688E-05 | 0.00029952 |
| AT3G16800 | 3.635474535 | 1.271E-156 | 3.873E-154 |
| AT3G16810 | -1.042230952 | 1.9524E-13 | 2.049E-12 |
| AT3G16990 | 1.746464651 | 6.9726E-19 | 1.082E-17 |
| AT3G17000 | 2.616403389 | 2.087E-108 | 3.615E-106 |
| AT3G17120 | -1.436482445 | 2.7648E-14 | 3.0813E-13 |
| AT3G17170 | -1.425784131 | 4.1066E-25 | 8.9998E-24 |
| AT3G17185 | -1.80749059 | 6.4747E-07 | 3.3782E-06 |
| AT3G00350 | 2.623588591 | 0.00052242 | 0.0016924 |
| AT3G17330 | -1.095871988 | 1.6344E-07 | 9.2612E-07 |
| AT3G17390 | -1.613341903 | 3.8374E-57 | 2.4639E-55 |
| AT3G17520 | 4.888989473 | 6.2675E-05 | 0.00024228 |
| AT3G17580 | -2.29756165 | 0.00477554 | 0.01246538 |
| AT3G17640 | -1.93533811 | 1.6365E-15 | 2.0286E-14 |
| AT3G17712 | 1.308073297 | 8.3584E-05 | 0.00031637 |
| AT3G17770 | 1.927705088 | 1.5605E-68 | 1.3615E-66 |
| AT3G17790 | 1.538765762 | 4.6728E-16 | 6.0048E-15 |
| AT3G17800 | 1.711714559 | 8.6313E-40 | 3.2937E-38 |
| AT3G17810 | 1.286782019 | 1.0305E-30 | 2.8974E-29 |
| AT3G17860 | 1.868624281 | 7.3623E-40 | 2.8283E-38 |
| AT3G18050 | -1.590280162 | 3.0922E-29 | 8.1523E-28 |
| AT3G18080 | -1.275766228 | 7.1052E-21 | 1.2436E-19 |
| AT3G18130 | -1.056543082 | 8.7676E-18 | 1.2597E-16 |
| AT3G18200 | -4.086751354 | 5.6345E-16 | 7.2033E-15 |
| AT3G18250 | -6.184416543 | 2.413E-14 | 2.7051E-13 |
| AT3G18280 | 1.310449157 | 2.7875E-28 | 6.9333E-27 |
| AT3G18290 | 1.523014422 | 7.1088E-42 | 2.9121E-40 |
| AT3G18320 | -1.882771393 | 1.19E-06 | 5.9925E-06 |
| AT3G18610 | 1.494570933 | 1.543E-10 | 1.2358E-09 |
| AT3G18620 | 1.044532343 | 3.0245E-07 | 1.652E-06 |
| AT3G18630 | -1.188917508 | 0.00455704 | 0.01194688 |
| AT3G18710 | -1.471958595 | 2.5525E-08 | 1.6113E-07 |
| AT3G18900 | -1.352903297 | 0.00013094 | 0.00047775 |
| AT3G18950 | 1.168985813 | 4.6028E-08 | 2.8116E-07 |
| AT3G19020 | -1.194037549 | 0.0001845 | 0.00065383 |
| AT3G19030 | 1.12525185 | 7.8552E-15 | 9.2411E-14 |
| AT3G19100 | 1.369391599 | 3.6762E-41 | 1.4563E-39 |
| AT3G19190 | 1.134746848 | 1.8394E-12 | 1.7711E-11 |
| AT3G19200 | 2.077919867 | 4.5115E-10 | 3.4455E-09 |
| AT3G19240 | 1.030825489 | 9.4213E-15 | 1.0948E-13 |
| AT3G19290 | 1.577142258 | 1.6007E-39 | 6.088E-38 |
| AT3G19370 | -1.382712215 | 6.1088E-26 | 1.3866E-24 |
| AT3G19400 | -1.57242273 | 1.037E-10 | 8.4596E-10 |
| AT3G19430 | -4.064237521 | 0.00012393 | 0.00045457 |
| AT3G19440 | -1.335180048 | 1.5417E-07 | 8.7704E-07 |
| AT3G19450 | -1.720162105 | 3.2901E-49 | 1.7489E-47 |
| AT3G19540 | -1.091397265 | 3.4176E-12 | 3.2069E-11 |
| AT3G19580 | 2.773894259 | 6.4012E-50 | 3.4267E-48 |
| AT3G19660 | -1.335017143 | 1.5777E-06 | 7.8261E-06 |
| AT3G19710 | -2.372522835 | 2.3168E-78 | 2.4517E-76 |
| AT3G19910 | 1.36945882 | 5.2988E-31 | 1.5027E-29 |
| AT3G19920 | 2.017958833 | 0.00039933 | 0.00132269 |
| AT3G20015 | -2.17414828 | 2.2382E-21 | 4.0572E-20 |
| AT3G20110 | -2.961080482 | 4.8246E-10 | 3.6773E-09 |
| AT3G20250 | 1.678818108 | 1.6299E-46 | 7.7456E-45 |
| AT3G20300 | 2.639821629 | 1.106E-57 | 7.1616E-56 |
| AT3G20370 | -2.141509144 | 3.3055E-49 | 1.7531E-47 |
| AT3G20475 | 1.193588835 | 0.00460004 | 0.01204579 |
| AT3G20640 | -1.342735106 | 4.8826E-06 | 2.2597E-05 |
| AT3G20660 | 1.195814607 | 3.3907E-08 | 2.1106E-07 |
| AT3G20820 | -1.083725394 | 7.3839E-19 | 1.1435E-17 |
| AT3G20910 | 1.120751414 | 4.5658E-07 | 2.4333E-06 |
| AT3G20960 | -1.634908175 | 6.0296E-06 | 2.7532E-05 |
| AT3G21090 | 1.557992077 | 2.1137E-15 | 2.592E-14 |
| AT3G21150 | 1.725351142 | 0.0004908 | 0.00159811 |
| AT3G21180 | -1.730854113 | 0.0012033 | 0.00360013 |
| AT3G21200 | -1.319264338 | 2.5989E-25 | 5.762E-24 |
| AT3G21270 | 1.060503537 | 5.8508E-09 | 3.9404E-08 |
| AT3G21300 | -1.175105164 | 1.7474E-12 | 1.6853E-11 |
| AT3G21430 | 1.16661863 | 8.3527E-16 | 1.0537E-14 |
| AT3G21510 | -1.175702168 | 1.3986E-12 | 1.3598E-11 |
| AT3G21520 | -2.087465906 | 0.00183411 | 0.00525893 |
| AT3G21530 | -1.148302227 | 2.4031E-05 | 0.00010005 |
| AT3G21560 | -1.306146413 | 3.2157E-32 | 9.5338E-31 |
| AT3G21660 | 5.079106771 | 6.0907E-09 | 4.0959E-08 |
| AT3G21700 | 1.035763509 | 1.6448E-09 | 1.1852E-08 |
| AT3G21710 | -1.356965913 | 5.6387E-10 | 4.2749E-09 |
| AT3G21720 | -5.649627796 | 0.00067907 | 0.00214365 |
| AT3G21770 | -1.559926397 | 2.3117E-16 | 3.0333E-15 |
| AT3G21865 | 1.053452965 | 1.5429E-20 | 2.6398E-19 |
| AT3G21890 | 1.660118138 | 7.2434E-05 | 0.00027701 |
| AT3G21950 | -1.749959094 | 9.0902E-06 | 4.0496E-05 |
| AT3G22060 | 1.313460521 | 2.3003E-10 | 1.81E-09 |
| AT3G22100 | 2.214098703 | 9.021E-05 | 0.00033954 |
| AT3G22200 | 1.42482952 | 4.561E-43 | 1.9378E-41 |
| AT3G22210 | -1.807909899 | 0.0016912 | 0.00489272 |
| AT3G22231 | -4.155926183 | 0.0035397 | 0.00951439 |
| AT3G22240 | -1.378807718 | 5.5389E-20 | 9.1811E-19 |
| AT3G22275 | 4.564465347 | 0.00296694 | 0.0081278 |
| AT3G22370 | 1.513971232 | 1.6615E-48 | 8.5152E-47 |
| AT3G22540 | -1.814765026 | 0.00598777 | 0.01524657 |
| AT3G22560 | 1.971942425 | 0.00053849 | 0.00174 |
| AT3G22740 | -1.116944905 | 1.6634E-07 | 9.4113E-07 |
| AT3G22800 | -3.489689576 | 1.43E-32 | 4.2898E-31 |
| AT3G22830 | 4.781909223 | 0 | 0 |
| AT3G22910 | 3.68145095 | 1.2494E-59 | 8.5249E-58 |
| AT3G22970 | -1.371624222 | 4.4318E-26 | 1.012E-24 |
| AT3G23000 | 1.434492625 | 5.6677E-43 | 2.3903E-41 |
| AT3G23030 | 1.028961062 | 2.6243E-17 | 3.6554E-16 |
| AT3G23110 | -1.468822829 | 0.00371082 | 0.00992999 |
| AT3G23175 | -2.65385881 | 5.6751E-12 | 5.2076E-11 |
| AT3G23180 | -1.597437029 | 2.7042E-05 | 0.00011154 |
| AT3G23190 | -2.113497052 | 3.7393E-11 | 3.1965E-10 |
| AT3G23230 | 4.083384141 | 2.0718E-05 | 8.7088E-05 |
| AT3G23290 | -1.575668837 | 2.4719E-07 | 1.3681E-06 |
| AT3G23370 | -1.863850806 | 7.256E-07 | 3.7678E-06 |
| AT3G23470 | -2.347814306 | 1.196E-16 | 1.6006E-15 |
| AT3G23510 | -1.623856871 | 1.2125E-07 | 6.9986E-07 |
| AT3G23530 | -1.259990924 | 5.3473E-31 | 1.5146E-29 |
| AT3G23550 | 1.129446162 | 0.00080362 | 0.00250228 |
| AT3G23605 | 2.133159959 | 1.2041E-32 | 3.6263E-31 |
| AT3G23730 | -1.144053084 | 1.1872E-09 | 8.692E-09 |
| AT3G23740 | -1.383335049 | 8.7378E-06 | 3.9048E-05 |
| AT3G23805 | -1.572852344 | 2.1921E-10 | 1.7278E-09 |
| AT3G23840 | 1.300571841 | 3.8267E-18 | 5.6651E-17 |
| AT3G23880 | -1.424161981 | 1.7491E-05 | 7.4382E-05 |
| AT3G23920 | 3.811350767 | 8.528E-301 | 1.026E-297 |
| AT3G23940 | -1.121636002 | 7.9408E-25 | 1.7221E-23 |
| AT3G24070 | 1.11449737 | 5.2248E-13 | 5.2705E-12 |
| AT3G24300 | -2.4778935 | 5.4522E-30 | 1.4766E-28 |
| AT3G24310 | 3.787465058 | 1.5057E-36 | 5.2305E-35 |
| AT3G24420 | 1.132920405 | 5.057E-23 | 1.0078E-21 |
| AT3G24450 | -1.466030347 | 0.00918312 | 0.02224071 |
| AT3G24500 | 1.653208654 | 2.7678E-18 | 4.1269E-17 |
| AT3G24510 | -6.145260302 | 0.00095977 | 0.00294278 |
| AT3G24650 | -4.963497648 | 0.00054257 | 0.00175197 |
| AT3G24740 | 1.069561003 | 4.3817E-15 | 5.2575E-14 |
| AT3G24770 | -1.119933823 | 1.6808E-08 | 1.0875E-07 |
| AT3G24840 | 1.288076539 | 1.5916E-15 | 1.9761E-14 |
| AT3G25010 | 1.518817504 | 0.00105963 | 0.00321064 |
| AT3G25130 | -2.691426472 | 4.0428E-12 | 3.7646E-11 |
| AT3G25190 | -2.05654152 | 6.8883E-33 | 2.105E-31 |
| AT3G25510 | -2.952335122 | 0.00560526 | 0.01437991 |
| AT3G25570 | 1.214787791 | 1.365E-18 | 2.0772E-17 |
| AT3G25573 | 6.607394937 | 1.2398E-21 | 2.289E-20 |
| AT3G25620 | 1.274743877 | 0.00017431 | 0.00062064 |
| AT3G25640 | -1.777109933 | 1.4093E-08 | 9.1828E-08 |
| AT3G25717 | -2.121589378 | 3.6509E-19 | 5.7632E-18 |
| AT3G25760 | 1.028202597 | 0.00073777 | 0.00231077 |
| AT3G25780 | 1.577014903 | 5.4537E-21 | 9.6188E-20 |
| AT3G25790 | -1.966560858 | 0.00055844 | 0.00179788 |
| AT3G25820 | -2.654759148 | 0.00347146 | 0.00934415 |
| AT3G25870 | 1.247932125 | 3.043E-09 | 2.1213E-08 |
| AT3G25890 | -1.086152149 | 2.2702E-09 | 1.6036E-08 |
| AT3G25930 | -1.154130416 | 1.9316E-06 | 9.4758E-06 |
| AT3G25940 | -1.290080872 | 1.445E-06 | 7.2118E-06 |
| AT3G26180 | -1.023741314 | 3.0354E-09 | 2.1167E-08 |
| AT3G26235 | -5.751004151 | 0.00404174 | 0.01072635 |
| AT3G26280 | 1.377049485 | 1.6636E-12 | 1.6072E-11 |
| AT3G26290 | 2.202316533 | 1.2594E-10 | 1.019E-09 |
| AT3G26320 | -3.314810026 | 5.1757E-05 | 0.00020301 |
| AT3G26330 | -1.617876986 | 5.4526E-08 | 3.2963E-07 |
| AT3G26440 | -1.080430516 | 8.7212E-13 | 8.6336E-12 |
| AT3G26460 | -1.095608208 | 8.1482E-10 | 6.0767E-09 |
| AT3G26470 | -2.912500177 | 6.5897E-38 | 2.3834E-36 |
| AT3G26490 | -1.158531633 | 0.00074437 | 0.00232919 |
| AT3G26500 | -1.284826361 | 0.00023712 | 0.00082086 |
| AT3G05055 | 3.675958292 | 0.00467689 | 0.01223161 |
| AT3G26539 | -1.597716889 | 0.0080751 | 0.01986232 |
| AT3G26570 | -1.321346034 | 3.9256E-30 | 1.0798E-28 |
| AT3G26700 | -1.389741047 | 1.6711E-08 | 1.0818E-07 |
| AT3G26760 | -1.653946806 | 9.108E-07 | 4.6585E-06 |
| AT3G26770 | -1.154135347 | 0.00012612 | 0.00046186 |
| AT3G26820 | -7.551859435 | 3.6064E-06 | 1.7025E-05 |
| AT3G26910 | 1.075902218 | 5.5944E-11 | 4.7134E-10 |
| AT3G26932 | -2.027698917 | 9.2539E-17 | 1.2487E-15 |
| AT3G26960 | -1.703244375 | 0.00056263 | 0.00181032 |
| AT3G27060 | -1.017788396 | 7.3347E-15 | 8.6466E-14 |
| AT3G27150 | -1.195696916 | 2.3368E-08 | 1.4805E-07 |
| AT3G27250 | 4.006358531 | 2.578E-119 | 4.994E-117 |
| AT3G27260 | 1.540450675 | 2.1973E-37 | 7.8478E-36 |
| AT3G27400 | -1.158741941 | 2.7664E-05 | 0.00011385 |
| AT3G05255 | -6.130778515 | 0.00101632 | 0.00309334 |
| AT3G27510 | -3.67428138 | 0.00155403 | 0.00453724 |
| AT3G27640 | -1.22425416 | 0.00725738 | 0.01806679 |
| AT3G27690 | -1.096222314 | 2.7449E-05 | 0.00011307 |
| AT3G27810 | 6.672165656 | 0.00106733 | 0.00323138 |
| AT3G27870 | 2.215293781 | 2.4396E-43 | 1.0482E-41 |
| AT3G27884 | -2.379851935 | 2.36E-07 | 1.3103E-06 |
| AT3G27930 | 1.154135509 | 4.9998E-14 | 5.4945E-13 |
| AT3G27950 | -5.500500589 | 0.00764395 | 0.01891172 |
| AT3G28007 | 4.878497278 | 6.755E-35 | 2.1995E-33 |
| AT3G28040 | -1.17056668 | 4.4141E-17 | 6.06E-16 |
| AT3G28080 | -2.115984328 | 5.179E-18 | 7.5983E-17 |
| AT3G28210 | 2.507606584 | 7.0069E-56 | 4.3523E-54 |
| AT3G28220 | -1.085261657 | 1.0989E-14 | 1.2692E-13 |
| AT3G28420 | -2.418081154 | 3.0825E-05 | 0.00012593 |
| AT3G28430 | 1.078366449 | 1.6007E-09 | 1.1568E-08 |
| AT3G28540 | -1.781616252 | 0.00356675 | 0.00957585 |
| AT3G28550 | -2.189990639 | 2.9102E-05 | 0.00011932 |
| AT3G28600 | 3.178283786 | 0.0085578 | 0.02091236 |
| AT3G28840 | -5.790519787 | 0.00325196 | 0.00881875 |
| AT3G28920 | -1.096712514 | 6.9454E-13 | 6.9236E-12 |
| AT3G28945 | 1.433912399 | 0.00721919 | 0.01798737 |
| AT3G28960 | -3.746353175 | 0.00060213 | 0.00192469 |
| AT3G29000 | 1.71494344 | 3.2272E-06 | 1.5362E-05 |
| AT3G29030 | -1.15721599 | 2.0679E-11 | 1.8082E-10 |
| AT3G29034 | -1.837845552 | 1.6105E-16 | 2.1378E-15 |
| AT3G29250 | -1.70234918 | 3.7174E-21 | 6.6489E-20 |
| AT3G29575 | 3.329852445 | 8.4E-236 | 6E-233 |
| AT3G29630 | -2.946898986 | 7.2139E-19 | 1.1179E-17 |
| AT3G29680 | -2.636450772 | 6.1591E-05 | 0.00023846 |
| AT3G29780 | -2.440212636 | 0.0044374 | 0.01166668 |
| AT3G30210 | 4.201322139 | 4.9507E-09 | 3.3619E-08 |
| AT3G30350 | -1.65552874 | 0.00811242 | 0.01994768 |
| AT3G30775 | -1.106236782 | 6.0562E-14 | 6.6204E-13 |
| AT3G41762 | 2.746949547 | 1.3614E-06 | 6.8153E-06 |
| AT3G41768 | 1.318832221 | 1.8223E-05 | 7.7282E-05 |
| AT3G06355 | 3.273083633 | 1.63E-125 | 3.45E-123 |
| AT3G06365 | 2.193271845 | 1.2573E-13 | 1.3436E-12 |
| AT3G42180 | -1.111487611 | 0.00874694 | 0.02131303 |
| AT3G42658 | -1.22791745 | 0.00453119 | 0.01188739 |
| AT3G43210 | 1.06084201 | 2.2824E-10 | 1.7965E-09 |
| AT3G43250 | 10.17740622 | 2.2091E-11 | 1.9281E-10 |
| AT3G43270 | 2.519936459 | 4.2404E-80 | 4.7747E-78 |
| AT3G06565 | 6.175733699 | 0.00469162 | 0.01226452 |
| AT3G43670 | -1.233927661 | 1.7892E-15 | 2.2047E-14 |
| AT3G43800 | -1.089863121 | 3.2596E-12 | 3.0662E-11 |
| AT3G43960 | -1.683712078 | 1.0008E-10 | 8.1844E-10 |
| AT3G44450 | -2.982192215 | 1.2694E-18 | 1.9383E-17 |
| AT3G44510 | -1.950637225 | 0.00306188 | 0.00835183 |
| AT3G44540 | -3.186645717 | 3.0371E-34 | 9.6286E-33 |
| AT3G44550 | -1.02734075 | 2.0125E-08 | 1.2882E-07 |
| AT3G44710 | -5.98978686 | 0.00203424 | 0.00578907 |
| AT3G44720 | -1.389652286 | 1.1433E-30 | 3.2065E-29 |
| AT3G44735 | 1.737616253 | 7.3508E-26 | 1.6636E-24 |
| AT3G44750 | -1.279250408 | 1.994E-18 | 3.0065E-17 |
| AT3G44860 | 4.355227617 | 1.1062E-30 | 3.1064E-29 |
| AT3G44880 | 2.003492994 | 2.5206E-61 | 1.8175E-59 |
| AT3G44940 | -1.575371126 | 8.9574E-08 | 5.2648E-07 |
| AT3G44970 | -1.187986141 | 1.7875E-15 | 2.2038E-14 |
| AT3G44990 | -5.282413178 | 9.635E-172 | 3.552E-169 |
| AT3G45050 | -1.075957869 | 6.0551E-10 | 4.5785E-09 |
| AT3G45070 | -1.260089439 | 5.9765E-05 | 0.00023201 |
| AT3G45160 | -1.467052573 | 3.0761E-11 | 2.6464E-10 |
| AT3G45230 | -1.37309613 | 2.1386E-07 | 1.1955E-06 |
| AT3G45330 | -3.153639002 | 0.00221714 | 0.00626213 |
| AT3G45410 | -2.58917279 | 3.1427E-10 | 2.4393E-09 |
| AT3G45430 | -1.265687991 | 0.0023275 | 0.00654149 |
| AT3G45650 | -2.154694195 | 6.8389E-12 | 6.2181E-11 |
| AT3G45680 | -2.21792849 | 8.7336E-22 | 1.631E-20 |
| AT3G45710 | -2.299015632 | 2.0518E-28 | 5.1425E-27 |
| AT3G45780 | -1.301163685 | 7.6178E-20 | 1.2509E-18 |
| AT3G45840 | -4.56523379 | 0.00057809 | 0.00185486 |
| AT3G45930 | -1.054211196 | 1.5623E-11 | 1.3793E-10 |
| AT3G45970 | 2.542934769 | 3.5299E-29 | 9.2637E-28 |
| AT3G46080 | 1.763554767 | 0.00024396 | 0.00084261 |
| AT3G46110 | 1.345751233 | 6.4768E-05 | 0.00024975 |
| AT3G46230 | 5.251527504 | 0.00017746 | 0.00063055 |
| AT3G46270 | -2.253442228 | 0.00140243 | 0.00413437 |
| AT3G46320 | -1.182796085 | 2.096E-13 | 2.1927E-12 |
| AT3G46370 | -2.481341519 | 0.00495356 | 0.01286978 |
| AT3G46450 | 1.706406558 | 7.8517E-42 | 3.2049E-40 |
| AT3G46490 | -1.832257627 | 3.1897E-12 | 3.0041E-11 |
| AT3G46540 | -1.153895222 | 1.9504E-12 | 1.8732E-11 |
| AT3G46640 | 1.028488897 | 6.7371E-19 | 1.0469E-17 |
| AT3G00810 | 2.116604531 | 0.00431119 | 0.01136752 |
| AT3G46690 | -3.319369833 | 5.2296E-12 | 4.8162E-11 |
| AT3G46700 | -2.267801374 | 5.5371E-29 | 1.435E-27 |
| AT3G46720 | -1.951274696 | 8.335E-05 | 0.00031564 |
| AT3G46760 | -5.441547825 | 0.00915529 | 0.02218271 |
| AT3G46900 | -1.98602395 | 1.6179E-08 | 1.0482E-07 |
| AT3G46940 | -1.591317496 | 7.0623E-18 | 1.0237E-16 |
| AT3G47040 | -2.909214787 | 9.3048E-05 | 0.00034942 |
| AT3G47050 | -3.972837874 | 0.00598871 | 0.01524727 |
| AT3G47060 | 1.032547059 | 2.1803E-12 | 2.0826E-11 |
| AT3G47070 | -1.214845657 | 5.6017E-24 | 1.1812E-22 |
| AT3G47080 | 1.153830707 | 4.7688E-20 | 7.9449E-19 |
| AT3G47295 | 1.575479636 | 7.5073E-05 | 0.00028658 |
| AT3G47340 | 1.125626136 | 7.9044E-06 | 3.5539E-05 |
| AT3G47430 | -1.718095453 | 1.7215E-19 | 2.7633E-18 |
| AT3G47480 | -1.32272027 | 0.00055535 | 0.00178994 |
| AT3G47500 | 1.181360463 | 5.7748E-14 | 6.3249E-13 |
| AT3G47510 | 1.260244428 | 3.9001E-09 | 2.6884E-08 |
| AT3G47580 | 2.725072 | 1.3206E-10 | 1.0659E-09 |
| AT3G47600 | 2.12472777 | 1.6441E-46 | 7.7967E-45 |
| AT3G47680 | 1.3158138 | 2.1773E-22 | 4.1964E-21 |
| AT3G47740 | -5.872286056 | 0.00246694 | 0.00689524 |
| AT3G47780 | -1.466814224 | 2.0282E-18 | 3.0521E-17 |
| AT3G47830 | -1.603557007 | 0.00317658 | 0.00863379 |
| AT3G47875 | 2.410866143 | 0.00096501 | 0.00295804 |
| AT3G47960 | 1.17584849 | 4.1642E-20 | 6.963E-19 |
| AT3G48020 | 1.869063919 | 1.7709E-10 | 1.4099E-09 |
| AT3G48080 | -2.248098212 | 1.2912E-05 | 5.6166E-05 |
| AT3G48090 | -1.205957532 | 8.0831E-11 | 6.6895E-10 |
| AT3G48200 | -1.522129528 | 1.4899E-33 | 4.6461E-32 |
| AT3G48240 | 2.709444981 | 2.492E-16 | 3.2643E-15 |
| AT3G48280 | -1.64445805 | 5.6944E-05 | 0.00022178 |
| AT3G48350 | 1.047732205 | 6.631E-15 | 7.8366E-14 |
| AT3G48360 | 1.024121997 | 4.052E-18 | 5.9794E-17 |
| AT3G48380 | 1.022195031 | 4.5775E-15 | 5.4839E-14 |
| AT3G48390 | 2.2218758 | 1.1093E-10 | 9.0233E-10 |
| AT3G48450 | -1.522964512 | 3.0405E-06 | 1.453E-05 |
| AT3G48460 | 1.843723675 | 8.8356E-43 | 3.699E-41 |
| AT3G48470 | 1.015361383 | 0.00097072 | 0.00297397 |
| AT3G48480 | -1.338403645 | 0.00279767 | 0.00771588 |
| AT3G48510 | 4.930490501 | 9.521E-195 | 4.946E-192 |
| AT3G48520 | 4.056261803 | 2.776E-173 | 1.04E-170 |
| AT3G48530 | 1.011511335 | 2.0608E-18 | 3.0991E-17 |
| AT3G48610 | -1.203706015 | 2.7128E-12 | 2.574E-11 |
| AT3G48920 | -2.755913808 | 8.4277E-14 | 9.1213E-13 |
| AT3G48970 | -1.899084968 | 5.4384E-05 | 0.00021253 |
| AT3G48990 | 1.879916179 | 7.3966E-49 | 3.8601E-47 |
| AT3G49120 | -1.123645693 | 4.1921E-17 | 5.7621E-16 |
| AT3G49220 | 2.442852715 | 6.9215E-97 | 1.0274E-94 |
| AT3G49260 | -1.694522926 | 1.3195E-30 | 3.6781E-29 |
| AT3G49330 | -1.43815483 | 0.00288054 | 0.00791767 |
| AT3G49530 | 1.008669661 | 6.0712E-14 | 6.6336E-13 |
| AT3G49540 | 4.4508381 | 3.0412E-08 | 1.9035E-07 |
| AT3G49570 | 1.591922274 | 8.5532E-21 | 1.4913E-19 |
| AT3G49620 | 2.852527016 | 6.044E-52 | 3.4028E-50 |
| AT3G49670 | -2.396566758 | 3.2217E-67 | 2.6778E-65 |
| AT3G49750 | -1.216023134 | 0.00111202 | 0.00335292 |
| AT3G49790 | 1.152658818 | 1.3107E-06 | 6.5758E-06 |
| AT3G49845 | -2.598394681 | 6.5867E-19 | 1.0249E-17 |
| AT3G49860 | -1.596707422 | 0.00017887 | 0.00063515 |
| AT3G49870 | 1.263349909 | 2.7642E-22 | 5.2963E-21 |
| AT3G49880 | 1.18583807 | 3.0948E-16 | 4.024E-15 |
| AT3G49930 | -1.724074207 | 0.00718319 | 0.0179133 |
| AT3G49940 | -1.388214409 | 1.777E-17 | 2.5011E-16 |
| AT3G49960 | -4.672498525 | 2.3679E-20 | 4.0034E-19 |
| AT3G50060 | 1.393333735 | 1.6086E-09 | 1.1618E-08 |
| AT3G50120 | -2.692322915 | 0.00303419 | 0.0082852 |
| AT3G50130 | -4.701802184 | 0.00032958 | 0.00110998 |
| AT3G50140 | -6.844790739 | 6.4769E-05 | 0.00024975 |
| AT3G50260 | 1.802969495 | 6.0247E-24 | 1.2657E-22 |
| AT3G50270 | -1.683287727 | 9.4715E-20 | 1.5453E-18 |
| AT3G50280 | -1.457610656 | 0.00730905 | 0.01818748 |
| AT3G50300 | -4.306995673 | 1.0977E-43 | 4.7886E-42 |
| AT3G50380 | 1.063285006 | 1.5082E-11 | 1.3331E-10 |
| AT3G08115 | 3.830314566 | 0.00525688 | 0.01357453 |
| AT3G50560 | -1.577426005 | 0.00202289 | 0.00575903 |
| AT3G50640 | -2.532015496 | 9.2573E-10 | 6.8546E-09 |
| AT3G50650 | 1.002982892 | 1.4826E-08 | 9.6386E-08 |
| AT3G50750 | -2.461760208 | 4.8596E-09 | 3.3069E-08 |
| AT3G50760 | 1.019357945 | 2.1448E-06 | 1.0458E-05 |
| AT3G50910 | 1.500701328 | 1.245E-41 | 5.0367E-40 |
| AT3G50970 | 3.945149918 | 0 | 0 |
| AT3G51000 | 1.068413923 | 2.2394E-14 | 2.5166E-13 |
| AT3G51080 | -1.44236412 | 4.7569E-07 | 2.5275E-06 |
| AT3G51090 | 1.170340926 | 5.8132E-08 | 3.5033E-07 |
| AT3G51130 | 1.505824475 | 3.606E-37 | 1.2799E-35 |
| AT3G51230 | -1.195108793 | 0.00130604 | 0.00387808 |
| AT3G51280 | -1.215333101 | 1.2106E-08 | 7.9335E-08 |
| AT3G51290 | -1.531638789 | 1.9746E-10 | 1.5666E-09 |
| AT3G51330 | -1.751459288 | 2.8358E-18 | 4.2256E-17 |
| AT3G51350 | -1.301405499 | 3.493E-05 | 0.00014134 |
| AT3G51360 | -2.680786771 | 0.00013259 | 0.00048337 |
| AT3G51410 | -6.061362898 | 0.00164445 | 0.00477077 |
| AT3G51470 | -1.447790637 | 2.5153E-06 | 1.2124E-05 |
| AT3G51540 | -2.22213504 | 1.2129E-05 | 5.3049E-05 |
| AT3G51660 | 1.107199716 | 6.473E-08 | 3.8713E-07 |
| AT3G51680 | -6.393815045 | 0.00038688 | 0.00128518 |
| AT3G51710 | -1.481209491 | 0.00233712 | 0.00656449 |
| AT3G51750 | 1.331138066 | 6.631E-08 | 3.9616E-07 |
| AT3G51830 | 1.001431206 | 5.4841E-12 | 5.0424E-11 |
| AT3G51860 | 1.861445894 | 4.2183E-42 | 1.7373E-40 |
| AT3G51890 | 1.575037519 | 6.5223E-17 | 8.869E-16 |
| AT3G51910 | 1.691686301 | 1.3399E-09 | 9.7659E-09 |
| AT3G51950 | -1.063901266 | 2.0657E-21 | 3.7594E-20 |
| AT3G52170 | -1.326581983 | 3.6639E-14 | 4.0596E-13 |
| AT3G52340 | 1.910990972 | 1.9174E-54 | 1.1473E-52 |
| AT3G52370 | -1.875973126 | 3.5446E-22 | 6.7689E-21 |
| AT3G52430 | -1.57156213 | 4.617E-09 | 3.1475E-08 |
| AT3G52450 | -1.329316817 | 5.2048E-07 | 2.7508E-06 |
| AT3G52500 | -1.094243417 | 5.8659E-18 | 8.5567E-17 |
| AT3G52520 | -1.522254768 | 0.00996925 | 0.02393668 |
| AT3G52630 | -1.903246866 | 6.7317E-10 | 5.07E-09 |
| AT3G52720 | -4.006815936 | 5.5045E-52 | 3.1067E-50 |
| AT3G52748 | -2.87845392 | 1.953E-15 | 2.4001E-14 |
| AT3G52790 | -1.773199712 | 1.3973E-05 | 6.0491E-05 |
| AT3G52800 | 1.238735272 | 9.1467E-25 | 1.9724E-23 |
| AT3G52870 | -1.174693245 | 8.991E-20 | 1.469E-18 |
| AT3G52900 | -1.32324412 | 3.5005E-08 | 2.1749E-07 |
| AT3G52910 | -1.083966267 | 2.716E-07 | 1.4923E-06 |
| AT3G53000 | 1.185203852 | 2.3263E-10 | 1.8286E-09 |
| AT3G53150 | -2.585814836 | 4.8807E-15 | 5.8288E-14 |
| AT3G53180 | 1.33740752 | 1.9097E-38 | 6.9955E-37 |
| AT3G53190 | -2.015682377 | 5.405E-44 | 2.3805E-42 |
| AT3G53230 | 1.597884307 | 2.1955E-36 | 7.5464E-35 |
| AT3G53232 | 3.468063678 | 1.9855E-10 | 1.5748E-09 |
| AT3G53235 | -2.46484807 | 0.00015591 | 0.00056042 |
| AT3G53260 | -1.608933995 | 2.7483E-61 | 1.9755E-59 |
| AT3G53420 | 2.076391752 | 3.209E-119 | 6.164E-117 |
| AT3G53450 | 1.429842369 | 0.00423185 | 0.01118284 |
| AT3G53530 | -1.133915652 | 0.00139376 | 0.00411132 |
| AT3G53540 | 1.21322758 | 6.2597E-17 | 8.527E-16 |
| AT3G53650 | 1.078997029 | 5.0103E-09 | 3.4004E-08 |
| AT3G53680 | -1.00867236 | 0.00032232 | 0.00108794 |
| AT3G53850 | -1.078609425 | 4.382E-06 | 2.0421E-05 |
| AT3G53930 | 1.027303247 | 7.1231E-10 | 5.3418E-09 |
| AT3G53960 | 2.248486626 | 1.8718E-48 | 9.5718E-47 |
| AT3G53990 | 1.059781135 | 1.114E-22 | 2.1763E-21 |
| AT3G54030 | 1.168492789 | 2.2249E-14 | 2.5016E-13 |
| AT3G54040 | -2.076853757 | 1.1959E-50 | 6.5395E-49 |
| AT3G54130 | 1.863949548 | 1.0924E-32 | 3.3031E-31 |
| AT3G54140 | 1.004778358 | 4.7612E-13 | 4.8113E-12 |
| AT3G54150 | -2.502402449 | 3.717E-16 | 4.811E-15 |
| AT3G54200 | 1.610139753 | 1.2952E-25 | 2.9112E-24 |
| AT3G08375 | 1.521728336 | 2.5325E-05 | 0.00010512 |
| AT3G54420 | -1.848217188 | 4.4024E-08 | 2.7022E-07 |
| AT3G54470 | -1.078513009 | 1.7375E-17 | 2.447E-16 |
| AT3G54580 | -2.19373401 | 1.9858E-06 | 9.7217E-06 |
| AT3G54590 | -4.024448459 | 3.2132E-40 | 1.2449E-38 |
| AT3G54600 | -2.766546584 | 1.2985E-92 | 1.7989E-90 |
| AT3G54620 | 1.142601427 | 1.7973E-23 | 3.6813E-22 |
| AT3G54640 | -1.402059321 | 2.9828E-33 | 9.1887E-32 |
| AT3G54680 | 1.572128723 | 2.5004E-31 | 7.1803E-30 |
| AT3G54770 | -1.489405663 | 1.7135E-05 | 7.302E-05 |
| AT3G54780 | -2.14938808 | 4.2893E-07 | 2.2935E-06 |
| AT3G54810 | -1.132876444 | 8.4426E-13 | 8.365E-12 |
| AT3G54820 | 1.315823174 | 5.1476E-21 | 9.1212E-20 |
| AT3G54830 | -1.286298967 | 4.5192E-08 | 2.7672E-07 |
| AT3G08435 | 5.727551042 | 5.1446E-22 | 9.7428E-21 |
| AT3G55070 | 1.160229001 | 1.6718E-16 | 2.2179E-15 |
| AT3G55090 | 1.832077857 | 7.1159E-16 | 9.0264E-15 |
| AT3G55120 | -1.626016874 | 1.3106E-51 | 7.2889E-50 |
| AT3G55130 | 1.044151683 | 1.4549E-16 | 1.9368E-15 |
| AT3G55150 | -2.733570439 | 2.2423E-09 | 1.5853E-08 |
| AT3G55230 | -2.030829024 | 5.9025E-18 | 8.5991E-17 |
| AT3G55240 | 1.570211429 | 0.00037328 | 0.00124363 |
| AT3G55290 | 1.094325905 | 1.5604E-07 | 8.8613E-07 |
| AT3G55430 | 1.742575558 | 3.4301E-54 | 2.0207E-52 |
| AT3G55510 | -1.169023892 | 4.363E-12 | 4.0491E-11 |
| AT3G55580 | 2.844437339 | 0.00018598 | 0.00065828 |
| AT3G55600 | 1.009854335 | 2.0208E-06 | 9.8805E-06 |
| AT3G55605 | -1.035339815 | 2.9274E-06 | 1.402E-05 |
| AT3G55610 | 1.654754488 | 7.1827E-63 | 5.4365E-61 |
| AT3G55630 | -1.44094184 | 1.7591E-29 | 4.681E-28 |
| AT3G55640 | 1.592360738 | 5.8777E-21 | 1.0335E-19 |
| AT3G55660 | -1.322976493 | 2.366E-09 | 1.6656E-08 |
| AT3G55710 | -1.902009632 | 2.5784E-07 | 1.4215E-06 |
| AT3G55790 | -6.219496809 | 0.00077792 | 0.00242654 |
| AT3G55840 | 3.946354291 | 6.9671E-60 | 4.7968E-58 |
| AT3G55880 | 3.099462145 | 1.7123E-96 | 2.5252E-94 |
| AT3G55900 | -3.8507152 | 0.00874574 | 0.02131238 |
| AT3G55940 | 3.273304951 | 1.5396E-61 | 1.1137E-59 |
| AT3G55970 | 3.899193567 | 4.466E-14 | 4.9197E-13 |
| AT3G56000 | -1.478865507 | 0.00075976 | 0.00237411 |
| AT3G56080 | 1.275417143 | 1.8018E-13 | 1.8962E-12 |
| AT3G56100 | -1.085435334 | 0.00015931 | 0.00057149 |
| AT3G56220 | -1.906284549 | 0.00150304 | 0.00440467 |
| AT3G56250 | 1.243846244 | 0.00025459 | 0.00087655 |
| AT3G56260 | 2.010110171 | 1.6128E-28 | 4.0689E-27 |
| AT3G56270 | 1.318515951 | 1.413E-13 | 1.5016E-12 |
| AT3G56360 | 1.108087991 | 6.4847E-25 | 1.4117E-23 |
| AT3G56400 | -1.656188542 | 1.4726E-08 | 9.579E-08 |
| AT3G56500 | -3.906090621 | 0.00794604 | 0.01957712 |
| AT3G56600 | 2.786368767 | 0.00155895 | 0.00454876 |
| AT3G56620 | 2.271119606 | 1.4307E-26 | 3.32E-25 |
| AT3G56790 | 3.670993046 | 3.7599E-09 | 2.5941E-08 |
| AT3G56810 | -1.404175412 | 4.0514E-09 | 2.7868E-08 |
| AT3G56825 | 1.396987487 | 0.00223597 | 0.00630907 |
| AT3G56880 | 1.932390004 | 2.3909E-56 | 1.4973E-54 |
| AT3G56970 | 3.248393648 | 8.0092E-07 | 4.1364E-06 |
| AT3G57010 | 1.231278576 | 2.4113E-27 | 5.7118E-26 |
| AT3G57130 | -4.378712567 | 1.0827E-05 | 4.7692E-05 |
| AT3G57230 | 1.384171399 | 6.4016E-24 | 1.3425E-22 |
| AT3G57260 | 2.871224823 | 0.00010059 | 0.00037559 |
| AT3G57410 | 1.209740132 | 8.1198E-29 | 2.0715E-27 |
| AT3G57460 | 2.537988526 | 1.0616E-05 | 4.6827E-05 |
| AT3G57520 | 1.843280547 | 2.1497E-67 | 1.8132E-65 |
| AT3G57540 | 1.841198915 | 3.6597E-30 | 1.0079E-28 |
| AT3G57680 | 2.390917178 | 1.0636E-17 | 1.5176E-16 |
| AT3G57760 | 2.075603442 | 3.8496E-18 | 5.6917E-17 |
| AT3G57830 | -1.162971161 | 4.0108E-08 | 2.4751E-07 |
| AT3G58100 | -1.461366938 | 8.4547E-06 | 3.7886E-05 |
| AT3G58120 | -2.278312524 | 8.0178E-17 | 1.0864E-15 |
| AT3G58750 | 1.185973502 | 6.4701E-23 | 1.2794E-21 |
| AT3G58850 | -2.849059308 | 7.7657E-08 | 4.6034E-07 |
| AT3G58990 | -4.354061534 | 4.589E-154 | 1.362E-151 |
| AT3G59010 | -2.181983725 | 4.1441E-14 | 4.5761E-13 |
| AT3G59030 | 7.170143122 | 0.00011338 | 0.00041928 |
| AT3G59050 | 1.322929593 | 6.3012E-29 | 1.6275E-27 |
| AT3G59140 | 1.298690608 | 1.3832E-26 | 3.2163E-25 |
| AT3G59250 | -1.937177748 | 9.9944E-05 | 0.00037359 |
| AT3G59340 | -2.691967781 | 1.6974E-08 | 1.0979E-07 |
| AT3G59350 | 1.400377475 | 5.3203E-30 | 1.4477E-28 |
| AT3G59370 | -1.277315715 | 0.00511612 | 0.01325147 |
| AT3G59480 | 2.224408292 | 1.223E-10 | 9.9096E-10 |
| AT3G59680 | -1.296495994 | 9.1355E-05 | 0.00034351 |
| AT3G59700 | -1.053774035 | 0.00080386 | 0.00250269 |
| AT3G59710 | -1.137728404 | 0.00539387 | 0.01388697 |
| AT3G59730 | -6.539135615 | 0.00027907 | 0.00095266 |
| AT3G59840 | -1.06200151 | 2.0868E-09 | 1.4832E-08 |
| AT3G60010 | 4.474511742 | 0.00608791 | 0.01548259 |
| AT3G60020 | 7.077367099 | 0.00015926 | 0.00057149 |
| AT3G60040 | 1.002285174 | 0.00450662 | 0.0118337 |
| AT3G60110 | 1.487649376 | 3.5292E-19 | 5.5749E-18 |
| AT3G60120 | -3.692820533 | 4.4035E-13 | 4.4696E-12 |
| AT3G60160 | 1.818146202 | 6.8897E-16 | 8.754E-15 |
| AT3G60220 | 1.680847131 | 1.0238E-13 | 1.1023E-12 |
| AT3G60270 | -5.804565756 | 6.7068E-07 | 3.4929E-06 |
| AT3G60280 | -5.302326178 | 1.2745E-05 | 5.5449E-05 |
| AT3G60300 | 1.589103749 | 8.8736E-41 | 3.4791E-39 |
| AT3G60330 | -2.196644796 | 1.2709E-16 | 1.6969E-15 |
| AT3G60415 | 1.785821413 | 1.1699E-27 | 2.8091E-26 |
| AT3G60520 | 1.20199594 | 6.4099E-08 | 3.8365E-07 |
| AT3G60590 | 1.012206295 | 1.6365E-05 | 6.9934E-05 |
| AT3G60620 | -1.130977975 | 9.6882E-12 | 8.7049E-11 |
| AT3G60870 | -3.660784507 | 0.00067467 | 0.00213094 |
| AT3G60900 | -1.030598069 | 3.9376E-06 | 1.8474E-05 |
| AT3G60930 | 1.809858604 | 6.1099E-15 | 7.2433E-14 |
| AT3G60966 | 3.027339795 | 3.4274E-05 | 0.00013886 |
| AT3G09545 | 4.897761595 | 0.00119984 | 0.00359121 |
| AT3G60970 | 8.36908784 | 2.9551E-07 | 1.6159E-06 |
| AT3G61060 | 3.324945005 | 3.3081E-90 | 4.422E-88 |
| AT3G61100 | -1.143205737 | 1.9257E-05 | 8.1408E-05 |
| AT3G61270 | -1.021366816 | 0.00065458 | 0.00207553 |
| AT3G61390 | -1.754651374 | 4.1894E-06 | 1.9591E-05 |
| AT3G61400 | -3.255999796 | 7.8891E-05 | 0.00030025 |
| AT3G61420 | 1.934995447 | 4.3737E-46 | 2.0487E-44 |
| AT3G61430 | 1.900815997 | 3.5623E-64 | 2.7602E-62 |
| AT3G61570 | 1.180875316 | 1.9008E-09 | 1.3569E-08 |
| AT3G61580 | 1.248581075 | 2.5708E-33 | 7.9518E-32 |
| AT3G61630 | 2.348843062 | 7.2739E-42 | 2.9744E-40 |
| AT3G61820 | -2.16152314 | 5.3495E-73 | 5.1378E-71 |
| AT3G61840 | -3.290554168 | 0.00366974 | 0.00983387 |
| AT3G61880 | -1.674622822 | 0.00045287 | 0.00148264 |
| AT3G61890 | 4.472036025 | 5.717E-308 | 7.259E-305 |
| AT3G61920 | -2.488445138 | 9.9616E-15 | 1.1541E-13 |
| AT3G61930 | -4.547501972 | 0.00066252 | 0.00209679 |
| AT3G61950 | -1.752073129 | 1.3205E-07 | 7.582E-07 |
| AT3G61960 | 1.021321727 | 2.5481E-14 | 2.851E-13 |
| AT3G62090 | 3.14151697 | 2.7214E-05 | 0.0001122 |
| AT3G62100 | 2.788300828 | 1.0932E-21 | 2.0283E-20 |
| AT3G62260 | 1.945073109 | 3.1892E-39 | 1.1951E-37 |
| AT3G62270 | -1.414000429 | 1.3848E-14 | 1.5858E-13 |
| AT3G62410 | -1.172025955 | 1.1393E-26 | 2.652E-25 |
| AT3G62455 | 1.743295095 | 4.0558E-11 | 3.454E-10 |
| AT3G62590 | 2.896218434 | 1.6141E-53 | 9.412E-52 |
| AT3G62660 | 1.560259783 | 3.7104E-39 | 1.3813E-37 |
| AT3G62680 | -4.215338146 | 4.7886E-26 | 1.0902E-24 |
| AT3G62730 | 2.303759539 | 3.0474E-07 | 1.6637E-06 |
| AT3G62770 | 1.086822025 | 1.1529E-12 | 1.1315E-11 |
| AT3G62920 | 1.169717772 | 0.0002728 | 0.00093319 |
| AT3G62930 | -1.200879531 | 0.0049298 | 0.0128168 |
| AT3G62990 | 2.45478358 | 1.1059E-07 | 6.4157E-07 |
| AT3G63050 | 1.243235566 | 0.00128523 | 0.0038233 |
| AT3G09885 | 7.695873934 | 9.0895E-06 | 4.0496E-05 |
| AT3G63060 | 5.332689485 | 6.644E-234 | 4.339E-231 |
| AT3G63070 | 1.221490457 | 3.7584E-21 | 6.717E-20 |
| AT3G63110 | -1.433668245 | 6.5237E-09 | 4.3717E-08 |
| AT3G63200 | -2.082358122 | 5.5102E-57 | 3.5182E-55 |
| AT3G63260 | 1.120997718 | 2.4935E-18 | 3.7252E-17 |
| AT3G63310 | 1.044941239 | 2.278E-16 | 2.9908E-15 |
| AT3G63445 | 1.262478487 | 1.8068E-07 | 1.0175E-06 |
| AT4G00020 | -1.068270168 | 7.0434E-06 | 3.1856E-05 |
| AT4G00050 | -1.305766861 | 8.7718E-15 | 1.024E-13 |
| AT4G00070 | -2.811087709 | 0.0014757 | 0.00433179 |
| AT4G00080 | -1.457670335 | 1.2871E-06 | 6.4617E-06 |
| AT4G00360 | 1.727732827 | 1.3624E-31 | 3.9469E-30 |
| AT4G00370 | -1.163476224 | 2.7245E-23 | 5.4967E-22 |
| AT4G00430 | 2.478847891 | 2.9069E-96 | 4.2322E-94 |
| AT4G00440 | 1.510926362 | 1.3145E-41 | 5.3086E-40 |
| AT4G00480 | -1.390875723 | 2.0794E-09 | 1.4794E-08 |
| AT4G00680 | -3.057858198 | 6.2262E-09 | 4.1836E-08 |
| AT4G00700 | -1.229525664 | 1.1593E-05 | 5.0913E-05 |
| AT4G00750 | 1.802673538 | 8.1784E-47 | 3.9606E-45 |
| AT4G00880 | -1.739056034 | 0.00322458 | 0.00875386 |
| AT4G00910 | 3.780232162 | 5.6859E-40 | 2.1917E-38 |
| AT4G00950 | -1.298709677 | 0.00014987 | 0.00054086 |
| AT4G01000 | 1.063204967 | 1.5878E-21 | 2.9081E-20 |
| AT4G01023 | 6.278238765 | 0.00334739 | 0.00904535 |
| AT4G01026 | 2.285761377 | 9.9095E-29 | 2.5224E-27 |
| AT4G01060 | 2.314621151 | 4.2951E-19 | 6.7429E-18 |
| AT4G01070 | 1.031492481 | 9.7221E-27 | 2.2723E-25 |
| AT4G01080 | -3.534823316 | 1.6561E-71 | 1.5203E-69 |
| AT4G01120 | 2.034466175 | 8.87E-52 | 4.9451E-50 |
| AT4G01270 | -1.175016664 | 0.00546621 | 0.01405633 |
| AT4G01350 | -1.920968668 | 3.3779E-07 | 1.8331E-06 |
| AT4G01360 | 2.740668885 | 8.0826E-09 | 5.3801E-08 |
| AT4G01540 | 1.479323516 | 7.7288E-13 | 7.6777E-12 |
| AT4G01550 | 1.419863046 | 2.3478E-15 | 2.8638E-14 |
| AT4G01580 | -1.204541133 | 0.00011342 | 0.0004193 |
| AT4G01630 | -2.246032434 | 4.4424E-07 | 2.3725E-06 |
| AT4G01700 | -1.193337678 | 9.9333E-07 | 5.0513E-06 |
| AT4G01740 | -1.740195444 | 0.00015715 | 0.00056446 |
| AT4G01750 | -1.32319697 | 8.1858E-05 | 0.00031035 |
| AT4G01770 | -2.611200823 | 0.00038238 | 0.00127188 |
| AT4G01870 | 2.207645153 | 1.87E-78 | 1.9974E-76 |
| AT4G01895 | 1.154557643 | 0.00274026 | 0.00757032 |
| AT4G01920 | -3.268985168 | 5.9917E-08 | 3.6042E-07 |
| AT4G01925 | -2.321346974 | 0.00397434 | 0.01056404 |
| AT4G01960 | 1.635433217 | 5.3453E-33 | 1.6356E-31 |
| AT4G01985 | 5.817269765 | 2.115E-120 | 4.204E-118 |
| AT4G02005 | 1.191848894 | 0.00033495 | 0.00112651 |
| AT4G02060 | -1.108458253 | 1.143E-11 | 1.0194E-10 |
| AT4G02270 | -3.228905274 | 4.7191E-12 | 4.3743E-11 |
| AT4G02280 | 2.610501291 | 6.8905E-84 | 8.3335E-82 |
| AT4G02290 | -1.086535104 | 1.4711E-10 | 1.1811E-09 |
| AT4G02360 | 2.574521379 | 1.1804E-44 | 5.3009E-43 |
| AT4G02380 | 2.77309365 | 4.203E-134 | 9.704E-132 |
| AT4G02410 | 2.338086671 | 2.1857E-46 | 1.0323E-44 |
| AT4G02800 | -1.299888408 | 3.0074E-10 | 2.3406E-09 |
| AT4G02850 | -3.635603873 | 9.7936E-19 | 1.5075E-17 |
| AT4G02880 | 1.071420475 | 6.4448E-11 | 5.3883E-10 |
| AT4G02890 | 1.626285565 | 1.5094E-60 | 1.0551E-58 |
| AT4G02990 | -1.299544301 | 2.5E-21 | 4.5246E-20 |
| AT4G03030 | 1.565629732 | 9.9516E-36 | 3.3305E-34 |
| AT4G03070 | -1.26233929 | 0.00010977 | 0.00040699 |
| AT4G03210 | -2.10514729 | 8.1054E-59 | 5.4492E-57 |
| AT4G03320 | 1.25050979 | 1.581E-12 | 1.5304E-11 |
| AT4G03330 | -2.597614441 | 0.00299519 | 0.00819241 |
| AT4G03340 | -1.332638941 | 0.00348898 | 0.00938578 |
| AT4G04595 | 1.067748949 | 0.00412065 | 0.01091549 |
| AT4G03420 | 1.046239779 | 2.2557E-12 | 2.151E-11 |
| AT4G03430 | 1.88299922 | 9.9962E-63 | 7.4916E-61 |
| AT4G03820 | 2.241927372 | 2.5458E-31 | 7.3014E-30 |
| AT4G04020 | 2.082943399 | 3.7933E-11 | 3.2378E-10 |
| AT4G04223 | 1.457679187 | 0.00056365 | 0.0018131 |
| AT4G04450 | -1.914660647 | 7.1646E-07 | 3.722E-06 |
| AT4G04540 | -4.000185464 | 0.00624693 | 0.01584293 |
| AT4G04570 | -2.002294186 | 2.404E-23 | 4.8672E-22 |
| AT4G04695 | -1.450241791 | 5.5181E-06 | 2.5333E-05 |
| AT4G04745 | -2.44361507 | 3.5323E-08 | 2.1929E-07 |
| AT4G04750 | -1.078250335 | 1.6449E-11 | 1.4489E-10 |
| AT4G04830 | -1.184198999 | 2.3999E-17 | 3.349E-16 |
| AT4G04840 | -1.824318387 | 1.4821E-07 | 8.4461E-07 |
| AT4G04940 | -1.106162849 | 7.4653E-12 | 6.7607E-11 |
| AT4G04990 | -5.152411395 | 5.4432E-29 | 1.4123E-27 |
| AT4G05010 | 1.461185783 | 1.2325E-11 | 1.0975E-10 |
| AT4G05020 | 1.108148021 | 1.801E-19 | 2.8889E-18 |
| AT4G05070 | 1.348734034 | 1.9801E-15 | 2.4321E-14 |
| AT4G05100 | 3.914888571 | 2.704E-145 | 7.186E-143 |
| AT4G05170 | -3.66759781 | 0.00095223 | 0.00292084 |
| AT4G05190 | -1.086335127 | 7.9256E-08 | 4.6885E-07 |
| AT4G05200 | -3.093987921 | 1.9916E-16 | 2.6238E-15 |
| AT4G05320 | 1.244963121 | 1.4688E-41 | 5.9214E-40 |
| AT4G05400 | -1.23593233 | 1.0474E-12 | 1.0302E-11 |
| AT4G06534 | 1.102053729 | 0.00017428 | 0.0006206 |
| AT4G06536 | 1.439381315 | 3.5692E-05 | 0.00014419 |
| AT4G06744 | -1.088213189 | 2.1263E-14 | 2.3931E-13 |
| AT4G06746 | 1.766278724 | 2.145E-14 | 2.4129E-13 |
| AT4G07960 | -1.908066993 | 2.4055E-05 | 0.00010014 |
| AT4G08150 | -2.334391599 | 1.7918E-16 | 2.3711E-15 |
| AT4G08170 | 1.186477858 | 5.1442E-15 | 6.1242E-14 |
| AT4G08290 | 1.177215791 | 3.4093E-08 | 2.1199E-07 |
| AT4G08300 | -1.309609324 | 2.085E-05 | 8.7591E-05 |
| AT4G08390 | -1.080196994 | 1.4022E-17 | 1.9883E-16 |
| AT4G08400 | -3.723961929 | 8.5916E-14 | 9.2942E-13 |
| AT4G08410 | -4.5677138 | 1.4366E-15 | 1.7876E-14 |
| AT4G08555 | 1.480199153 | 1.1406E-19 | 1.8504E-18 |
| AT4G08685 | -1.417505302 | 4.6347E-15 | 5.5495E-14 |
| AT4G08770 | -2.996444396 | 3.4389E-25 | 7.5729E-24 |
| AT4G08780 | -2.675620348 | 1.1906E-08 | 7.8067E-08 |
| AT4G08950 | -2.361314153 | 1.9105E-23 | 3.8956E-22 |
| AT4G09030 | 2.014725899 | 0.00107069 | 0.00324005 |
| AT4G09100 | -7.377488052 | 6.9995E-06 | 3.1682E-05 |
| AT4G09160 | -1.159793407 | 4.8577E-11 | 4.1186E-10 |
| AT4G09300 | -3.508411939 | 8.7427E-07 | 4.4908E-06 |
| AT4G09420 | -2.003021961 | 8.7052E-06 | 3.891E-05 |
| AT4G09500 | 1.319902866 | 6.4198E-17 | 8.7347E-16 |
| AT4G09550 | -1.065274221 | 9.5605E-06 | 4.2492E-05 |
| AT4G09650 | -1.204591 | 4.5602E-32 | 1.345E-30 |
| AT4G09760 | 1.989688411 | 1.0694E-48 | 5.543E-47 |
| AT4G09820 | 2.164360896 | 4.356E-22 | 8.2836E-21 |
| AT4G09990 | -2.066011558 | 1.1496E-07 | 6.6594E-07 |
| AT4G10120 | -1.379545483 | 2.355E-23 | 4.7752E-22 |
| AT4G10250 | 3.539658464 | 1.0163E-31 | 2.9517E-30 |
| AT4G10350 | -3.814406141 | 0.0004174 | 0.00137615 |
| AT4G10370 | -2.18901241 | 0.00265664 | 0.00736871 |
| AT4G10380 | -1.437552907 | 1.5201E-09 | 1.1016E-08 |
| AT4G10390 | -1.566652333 | 7.0765E-06 | 3.1993E-05 |
| AT4G10510 | -2.940939093 | 2.1699E-13 | 2.2628E-12 |
| AT4G10530 | -6.64179674 | 8.4616E-09 | 5.6274E-08 |
| AT4G10580 | 2.495404267 | 0.00543183 | 0.01397735 |
| AT4G10590 | 1.051571292 | 2.761E-12 | 2.6176E-11 |
| AT4G10720 | -2.783513452 | 7.3129E-05 | 0.00027953 |
| AT4G10955 | 1.14947618 | 1.3751E-11 | 1.2192E-10 |
| AT4G10960 | 3.58086425 | 9.072E-138 | 2.23E-135 |
| AT4G11050 | -2.251298592 | 5.8061E-06 | 2.6581E-05 |
| AT4G11140 | -1.06259138 | 0.00246149 | 0.00688339 |
| AT4G11170 | -6.211547696 | 0.00022853 | 0.00079366 |
| AT4G11190 | -2.258536848 | 3.2789E-18 | 4.87E-17 |
| AT4G11220 | 1.021416759 | 2.7605E-21 | 4.9724E-20 |
| AT4G11350 | 1.03884068 | 1.0599E-05 | 4.6762E-05 |
| AT4G11460 | -7.473092182 | 2.884E-12 | 2.7297E-11 |
| AT4G11480 | -3.51730338 | 1.1922E-05 | 5.2267E-05 |
| AT4G11530 | -1.721385796 | 0.00012847 | 0.00046976 |
| AT4G11560 | 1.024863087 | 1.4981E-17 | 2.1203E-16 |
| AT4G11570 | 2.046656295 | 4.6853E-77 | 4.846E-75 |
| AT4G11610 | -1.535091546 | 0.00345328 | 0.00930289 |
| AT4G11650 | -2.431200788 | 9.1567E-60 | 6.2666E-58 |
| AT4G11655 | -4.913208057 | 0.00011574 | 0.00042669 |
| AT4G11660 | 1.152650282 | 3.9627E-08 | 2.4461E-07 |
| AT4G11800 | 1.118357478 | 2.9349E-17 | 4.0658E-16 |
| AT4G11890 | 3.302264973 | 1.7601E-59 | 1.1974E-57 |
| AT4G11910 | 1.642373641 | 6.0945E-05 | 0.00023603 |
| AT4G11990 | 1.036377981 | 0.00125265 | 0.00373799 |
| AT4G12000 | 2.53712155 | 3.6861E-47 | 1.8042E-45 |
| AT4G12005 | 2.757061784 | 6.9581E-05 | 0.00026695 |
| AT4G12020 | 1.823696402 | 7.6748E-36 | 2.5875E-34 |
| AT4G12030 | -3.017274188 | 1.7343E-78 | 1.8611E-76 |
| AT4G12120 | 1.08662591 | 1.8127E-08 | 1.1675E-07 |
| AT4G12320 | -3.37858416 | 7.224E-126 | 1.543E-123 |
| AT4G12390 | -2.274086028 | 3.2026E-33 | 9.8526E-32 |
| AT4G12400 | 1.073981928 | 9.7855E-13 | 9.6412E-12 |
| AT4G12410 | 5.432939032 | 3.0614E-48 | 1.5516E-46 |
| AT4G12420 | -1.133459769 | 6.8765E-25 | 1.4941E-23 |
| AT4G12430 | 2.284410878 | 1.8829E-34 | 6.0028E-33 |
| AT4G12470 | -1.510636102 | 0.00356159 | 0.00956648 |
| AT4G12480 | -2.365804326 | 3.0537E-42 | 1.2645E-40 |
| AT4G12490 | -2.058172357 | 1.9895E-05 | 8.392E-05 |
| AT4G12500 | -1.726745573 | 0.00343087 | 0.00925015 |
| AT4G12510 | -4.018126721 | 4.8444E-06 | 2.2438E-05 |
| AT4G12520 | -3.971475725 | 5.3675E-18 | 7.8597E-17 |
| AT4G12545 | -2.9000911 | 0.00523689 | 0.01352902 |
| AT4G12580 | 3.155826497 | 2.0128E-25 | 4.4974E-24 |
| AT4G12730 | -2.206545301 | 1.2908E-62 | 9.6422E-61 |
| AT4G12735 | 2.28770495 | 0.00036616 | 0.00122186 |
| AT4G12830 | -1.872088988 | 1.7761E-36 | 6.1512E-35 |
| AT4G12900 | -1.7689432 | 2.1513E-10 | 1.6998E-09 |
| AT4G12917 | -2.163777007 | 1.2297E-09 | 8.9941E-09 |
| AT4G12980 | -2.177198042 | 1.5677E-23 | 3.2284E-22 |
| AT4G13100 | -1.044628281 | 2.4498E-11 | 2.1292E-10 |
| AT4G13110 | 2.136099718 | 6.8581E-29 | 1.7653E-27 |
| AT4G13235 | -3.140330629 | 0.0033335 | 0.00901528 |
| AT4G13280 | -2.85304795 | 2.37E-11 | 2.0621E-10 |
| AT4G13290 | -1.489090801 | 1.7017E-13 | 1.7941E-12 |
| AT4G13345 | 1.215852622 | 1.4669E-12 | 1.4238E-11 |
| AT4G13390 | -3.756154755 | 3.029E-16 | 3.9452E-15 |
| AT4G13395 | 2.616628296 | 1.2457E-05 | 5.4328E-05 |
| AT4G13410 | -2.621840226 | 1.4101E-30 | 3.9212E-29 |
| AT4G13440 | -6.53392947 | 0.00023218 | 0.00080546 |
| AT4G13495 | -1.036848132 | 1.1288E-17 | 1.6046E-16 |
| AT4G13500 | -1.127842284 | 3.5891E-10 | 2.7642E-09 |
| AT4G13570 | -1.576996132 | 0.00652994 | 0.01645541 |
| AT4G13572 | -1.415945225 | 0.00040551 | 0.00134104 |
| AT4G06120 | -3.037921749 | 0.0094801 | 0.022909 |
| AT4G13575 | -1.621102947 | 1.9244E-07 | 1.0811E-06 |
| AT4G13580 | -3.207494789 | 5.4345E-14 | 5.9636E-13 |
| AT4G13620 | -2.144294688 | 0.00104265 | 0.00316507 |
| AT4G13660 | -2.175601181 | 1.1857E-21 | 2.1927E-20 |
| AT4G13770 | -2.664924753 | 2.931E-108 | 5.037E-106 |
| AT4G13800 | 2.342247648 | 5.0045E-12 | 4.6238E-11 |
| AT4G13810 | -1.302270685 | 2.083E-06 | 1.0176E-05 |
| AT4G13830 | 1.116467957 | 2.0327E-15 | 2.4954E-14 |
| AT4G13890 | -4.285723377 | 0.00222757 | 0.00628848 |
| AT4G13930 | -1.36674269 | 2.1966E-44 | 9.7686E-43 |
| AT4G14040 | -1.755015071 | 9.9334E-42 | 4.0259E-40 |
| AT4G14096 | -1.040530507 | 4.4532E-05 | 0.000177 |
| AT4G14103 | -1.40500855 | 0.00246063 | 0.00688181 |
| AT4G14120 | -1.44967742 | 0.00468278 | 0.01224281 |
| AT4G14130 | -3.622837702 | 0.00272513 | 0.00753307 |
| AT4G14270 | 1.260872657 | 1.5425E-29 | 4.1093E-28 |
| AT4G14380 | -3.691545672 | 7.0124E-05 | 0.00026881 |
| AT4G14400 | -3.37686705 | 7.2349E-58 | 4.7386E-56 |
| AT4G14465 | -2.180648362 | 1.4894E-21 | 2.7388E-20 |
| AT4G14500 | 1.00787573 | 2.5181E-14 | 2.8187E-13 |
| AT4G14550 | -1.957152456 | 4.7931E-11 | 4.0653E-10 |
| AT4G14630 | -2.880809556 | 9.0347E-42 | 3.6681E-40 |
| AT4G14650 | -2.193951451 | 5.1599E-06 | 2.3794E-05 |
| AT4G14746 | 2.056492132 | 9.1623E-46 | 4.2481E-44 |
| AT4G14750 | -3.109252383 | 7.6261E-13 | 7.5823E-12 |
| AT4G14930 | 1.538603045 | 1.9184E-30 | 5.3024E-29 |
| AT4G14940 | -1.620325921 | 2.1339E-05 | 8.9483E-05 |
| AT4G15070 | -1.29937802 | 0.0056305 | 0.01442847 |
| AT4G15100 | 2.923388854 | 0.00070232 | 0.00221154 |
| AT4G15120 | 1.84150808 | 1.7406E-16 | 2.3062E-15 |
| AT4G15210 | 2.619736064 | 1.2389E-64 | 9.665E-63 |
| AT4G15320 | -3.149282939 | 8.6095E-08 | 5.0721E-07 |
| AT4G15380 | -2.374022992 | 0.00090325 | 0.00278403 |
| AT4G15390 | -1.408678958 | 4.3589E-09 | 2.9858E-08 |
| AT4G15393 | -1.538564243 | 0.00013738 | 0.00049886 |
| AT4G15420 | 1.254385095 | 2.297E-17 | 3.2074E-16 |
| AT4G15480 | -3.497328581 | 1.5708E-79 | 1.7345E-77 |
| AT4G15490 | 1.171510709 | 3.0335E-16 | 3.9487E-15 |
| AT4G15500 | -1.840758598 | 1.6391E-09 | 1.1816E-08 |
| AT4G15530 | 1.687400188 | 2.8124E-43 | 1.2039E-41 |
| AT4G15540 | -1.032879465 | 3.5757E-08 | 2.2186E-07 |
| AT4G15545 | -1.055242023 | 1.4098E-23 | 2.9084E-22 |
| AT4G15620 | -1.200916683 | 2.5416E-13 | 2.63E-12 |
| AT4G15640 | -1.007273413 | 2.6504E-09 | 1.8578E-08 |
| AT4G15690 | -1.271321115 | 0.00375442 | 0.01003257 |
| AT4G15810 | -1.000606344 | 3.6315E-07 | 1.9624E-06 |
| AT4G15830 | -1.293946981 | 1.414E-10 | 1.1376E-09 |
| AT4G15910 | 1.328683893 | 1.3417E-24 | 2.8796E-23 |
| AT4G15960 | -1.275635766 | 2.0417E-05 | 8.5964E-05 |
| AT4G15990 | 1.329051326 | 0.00091776 | 0.00282467 |
| AT4G16000 | 2.561538156 | 2.3683E-14 | 2.6563E-13 |
| AT4G16140 | -2.121029253 | 1.0864E-25 | 2.4466E-24 |
| AT4G16190 | 1.218325815 | 3.1773E-26 | 7.3213E-25 |
| AT4G16220 | -3.717563721 | 0.00072984 | 0.00228813 |
| AT4G16230 | -2.926363533 | 0.00027056 | 0.00092638 |
| AT4G16250 | 1.278331164 | 1.399E-20 | 2.399E-19 |
| AT4G16260 | -1.62157792 | 1.4098E-26 | 3.2748E-25 |
| AT4G16270 | -3.084395362 | 6.5983E-05 | 0.000254 |
| AT4G16350 | -2.453005458 | 5.1118E-05 | 0.0002007 |
| AT4G16390 | -1.052366377 | 1.2744E-13 | 1.3613E-12 |
| AT4G16545 | 6.82984123 | 0.00044353 | 0.00145433 |
| AT4G16563 | -1.527730071 | 2.9694E-06 | 1.4206E-05 |
| AT4G16590 | -3.34833302 | 0.00294461 | 0.00807727 |
| AT4G16680 | 2.494783594 | 1.2137E-21 | 2.2427E-20 |
| AT4G16690 | 1.225537948 | 5.2807E-10 | 4.0089E-09 |
| AT4G16760 | 2.184307328 | 4.532E-99 | 6.8602E-97 |
| AT4G16765 | 1.135896649 | 1.0737E-12 | 1.0551E-11 |
| AT4G16880 | -1.915779558 | 4.9893E-11 | 4.2286E-10 |
| AT4G16910 | 2.731815579 | 0.00052681 | 0.00170587 |
| AT4G16920 | -1.090809787 | 0.00271331 | 0.00750402 |
| AT4G17030 | 1.276626778 | 1.9531E-18 | 2.9488E-17 |
| AT4G17090 | -1.515580242 | 1.1381E-28 | 2.8906E-27 |
| AT4G17140 | 1.191954702 | 1.9242E-23 | 3.92E-22 |
| AT4G17230 | 2.176771364 | 1.9432E-82 | 2.2778E-80 |
| AT4G17245 | 2.733565476 | 8.5108E-56 | 5.2578E-54 |
| AT4G17340 | 1.407023123 | 5.6952E-38 | 2.0631E-36 |
| AT4G17410 | 1.268368048 | 5.5723E-18 | 8.1491E-17 |
| AT4G17460 | -1.89298757 | 5.733E-13 | 5.7577E-12 |
| AT4G17550 | 2.770041641 | 4.7003E-68 | 4.0543E-66 |
| AT4G17650 | 1.98584862 | 4.4606E-56 | 2.7782E-54 |
| AT4G17690 | 6.965368895 | 0.00026208 | 0.00090069 |
| AT4G17730 | 1.037707887 | 9.0351E-14 | 9.7647E-13 |
| AT4G17800 | -1.300952537 | 1.9497E-08 | 1.2505E-07 |
| AT4G17810 | -2.193021943 | 4.5044E-11 | 3.8304E-10 |
| AT4G17840 | 2.09309309 | 4.4942E-68 | 3.8912E-66 |
| AT4G17880 | -1.249718423 | 2.0472E-11 | 1.7909E-10 |
| AT4G17970 | 1.391971419 | 2.6282E-11 | 2.2756E-10 |
| AT4G18010 | 1.265432377 | 1.0418E-29 | 2.7949E-28 |
| AT4G18140 | 1.347270289 | 1.066E-14 | 1.2331E-13 |
| AT4G18170 | 3.660324901 | 2.4925E-67 | 2.0869E-65 |
| AT4G18197 | -1.618519611 | 0.00295546 | 0.00810246 |
| AT4G18205 | -1.427174325 | 9.1425E-05 | 0.00034371 |
| AT4G18210 | 2.558418974 | 5.401E-17 | 7.3882E-16 |
| AT4G18250 | -1.740493096 | 0.00369312 | 0.00988955 |
| AT4G18260 | -2.664475441 | 5.1987E-05 | 0.00020386 |
| AT4G18280 | 1.931754631 | 3.3438E-32 | 9.9005E-31 |
| AT4G18290 | -1.098893485 | 5.9877E-05 | 0.00023222 |
| AT4G18430 | -2.968376983 | 2.6487E-14 | 2.9577E-13 |
| AT4G18490 | 1.36264758 | 0.00505713 | 0.01311502 |
| AT4G18530 | 1.22945095 | 5.2473E-12 | 4.8286E-11 |
| AT4G18610 | -2.99199446 | 8.6503E-08 | 5.0921E-07 |
| AT4G18700 | 1.653873926 | 3.0014E-48 | 1.5246E-46 |
| AT4G18760 | -1.770115987 | 6.519E-13 | 6.5127E-12 |
| AT4G18940 | -2.644552467 | 0.00154035 | 0.00450105 |
| AT4G18980 | 5.07061781 | 1.2551E-14 | 1.4432E-13 |
| AT4G19230 | 1.213668079 | 6.7328E-12 | 6.1266E-11 |
| AT4G19380 | -2.951443766 | 2.5456E-16 | 3.3308E-15 |
| AT4G19390 | 1.478201893 | 1.7591E-20 | 3.0029E-19 |
| AT4G19460 | -1.97190314 | 2.0271E-05 | 8.5395E-05 |
| AT4G19530 | -1.548312304 | 5.2108E-21 | 9.226E-20 |
| AT4G19590 | -2.337401416 | 0.00148422 | 0.004354 |
| AT4G19690 | 1.523374296 | 1.4065E-08 | 9.1671E-08 |
| AT4G19810 | -1.102123136 | 1.5536E-10 | 1.2438E-09 |
| AT4G19970 | -3.025034471 | 1.4318E-11 | 1.2685E-10 |
| AT4G19975 | -2.370646826 | 9.6044E-08 | 5.6253E-07 |
| AT4G19980 | -3.977770692 | 0.00623337 | 0.01581732 |
| AT4G20000 | -2.531392923 | 2.2918E-09 | 1.6179E-08 |
| AT4G20050 | -2.075979755 | 0.00051733 | 0.00167875 |
| AT4G20070 | 1.739672405 | 8.7347E-28 | 2.1173E-26 |
| AT4G20110 | -1.161298324 | 4.4671E-09 | 3.0544E-08 |
| AT4G20140 | -1.27751882 | 1.03E-05 | 4.5495E-05 |
| AT4G20230 | -2.44940243 | 8.3172E-07 | 4.2857E-06 |
| AT4G20235 | -3.607393855 | 4.6066E-05 | 0.00018255 |
| AT4G20240 | -2.134134674 | 5.0679E-09 | 3.4365E-08 |
| AT4G20320 | 1.056736232 | 2.5713E-12 | 2.4439E-11 |
| AT4G20390 | -2.337258531 | 1.1501E-20 | 1.9885E-19 |
| AT4G20430 | -1.063841827 | 4.8488E-08 | 2.95E-07 |
| AT4G20730 | 1.974322211 | 1.1154E-11 | 9.963E-11 |
| AT4G20780 | -1.361263919 | 6.0286E-08 | 3.6245E-07 |
| AT4G20830 | 1.013462613 | 5.8083E-20 | 9.6138E-19 |
| AT4G20880 | 1.333651581 | 3.3992E-15 | 4.1089E-14 |
| AT4G20940 | -1.776834704 | 7.6773E-19 | 1.1873E-17 |
| AT4G20970 | 1.215740538 | 0.0005714 | 0.00183545 |
| AT4G21060 | 1.663704989 | 1.1834E-21 | 2.1902E-20 |
| AT4G21230 | -2.359345072 | 1.473E-05 | 6.355E-05 |
| AT4G21270 | -1.161372862 | 8.0501E-06 | 3.6137E-05 |
| AT4G21320 | 1.798049536 | 8.5538E-15 | 1.0011E-13 |
| AT4G21323 | 1.928817721 | 1.0195E-05 | 4.5057E-05 |
| AT4G21340 | -4.562235254 | 2.791E-06 | 1.3402E-05 |
| AT4G21390 | 1.793222672 | 1.1785E-13 | 1.2618E-12 |
| AT4G21440 | 4.207258212 | 2.8775E-54 | 1.6996E-52 |
| AT4G21445 | -1.392171881 | 1.0973E-11 | 9.8051E-11 |
| AT4G21480 | 1.016861339 | 0.00779677 | 0.01924572 |
| AT4G21534 | 1.099352851 | 1.0412E-10 | 8.4879E-10 |
| AT4G21560 | 1.071003362 | 2.5673E-12 | 2.441E-11 |
| AT4G21570 | 1.516524932 | 2.7298E-36 | 9.341E-35 |
| AT4G21580 | 1.199176434 | 1.1734E-28 | 2.9769E-27 |
| AT4G21600 | -1.57806257 | 6.6553E-10 | 5.014E-09 |
| AT4G21620 | 1.438792205 | 1.8012E-05 | 7.6457E-05 |
| AT4G21650 | 1.255055119 | 3.174E-23 | 6.3809E-22 |
| AT4G21680 | 3.395374514 | 4.063E-86 | 5.1028E-84 |
| AT4G21710 | 1.028344537 | 1.5006E-22 | 2.9068E-21 |
| AT4G21760 | -2.269829594 | 1.0746E-05 | 4.7375E-05 |
| AT4G21770 | -1.056592067 | 4.4426E-09 | 3.0386E-08 |
| AT4G21850 | -1.708037886 | 0.00568552 | 0.01455479 |
| AT4G21870 | -1.571754316 | 6.2407E-08 | 3.7416E-07 |
| AT4G21903 | -2.731582467 | 2.1738E-05 | 9.1071E-05 |
| AT4G21930 | 3.273527222 | 1.917E-11 | 1.6815E-10 |
| AT4G21940 | 1.043442898 | 0.0005223 | 0.00169223 |
| AT4G22010 | -1.235014194 | 1.6962E-23 | 3.4835E-22 |
| AT4G22030 | -7.628498745 | 2.5399E-06 | 1.2235E-05 |
| AT4G22070 | -2.672653339 | 6.0506E-23 | 1.1995E-21 |
| AT4G22080 | -3.746916606 | 3.9462E-05 | 0.00015833 |
| AT4G22220 | 1.044720282 | 5.3037E-21 | 9.3687E-20 |
| AT4G22240 | 2.00692754 | 5.144E-55 | 3.0943E-53 |
| AT4G22270 | 1.937408266 | 2.662E-17 | 3.7034E-16 |
| AT4G22530 | 1.088559108 | 2.4355E-15 | 2.9659E-14 |
| AT4G22560 | -2.512880259 | 5.0082E-06 | 2.3146E-05 |
| AT4G22590 | 1.342762433 | 2.4285E-25 | 5.3946E-24 |
| AT4G22610 | -2.106710913 | 2.3744E-07 | 1.317E-06 |
| AT4G22620 | 1.773568963 | 4.1784E-05 | 0.0001668 |
| AT4G22640 | -3.953742607 | 0.00680632 | 0.01707782 |
| AT4G22666 | -4.997007639 | 2.6632E-21 | 4.8047E-20 |
| AT4G22690 | -1.781839711 | 3.4535E-25 | 7.5977E-24 |
| AT4G22710 | -2.447411929 | 5.059E-11 | 4.2845E-10 |
| AT4G22790 | -1.42602125 | 0.0005601 | 0.00180269 |
| AT4G22810 | -1.522085032 | 1.9509E-06 | 9.5655E-06 |
| AT4G22820 | 1.497110052 | 2.7338E-32 | 8.1472E-31 |
| AT4G22900 | -1.951998074 | 0.00959093 | 0.02313231 |
| AT4G22920 | 1.639985205 | 5.4302E-31 | 1.5362E-29 |
| AT4G22950 | 1.197486572 | 0.001274 | 0.00379328 |
| AT4G22960 | 3.672730543 | 6.0664E-26 | 1.3784E-24 |
| AT4G22970 | 1.069207144 | 3.5304E-08 | 2.1923E-07 |
| AT4G23040 | 1.174844065 | 2.5731E-13 | 2.6613E-12 |
| AT4G23050 | 3.105021643 | 1.25E-192 | 5.951E-190 |
| AT4G23060 | 1.573323294 | 5.3659E-32 | 1.5786E-30 |
| AT4G23170 | -1.044412626 | 7.813E-05 | 0.00029745 |
| AT4G23220 | 1.273941261 | 7.6128E-06 | 3.4336E-05 |
| AT4G23280 | -3.058694224 | 0.00165799 | 0.00480699 |
| AT4G23300 | -1.663358939 | 1.4417E-12 | 1.4E-11 |
| AT4G23450 | 1.987196067 | 3.9038E-22 | 7.4423E-21 |
| AT4G23496 | -3.944343654 | 1.8869E-39 | 7.1527E-38 |
| AT4G23510 | -2.055655285 | 9.6181E-07 | 4.9052E-06 |
| AT4G23550 | -2.234087889 | 4.2138E-13 | 4.2885E-12 |
| AT4G23600 | 1.371524951 | 0.00100508 | 0.00306403 |
| AT4G23630 | 1.024489439 | 3.4251E-28 | 8.4913E-27 |
| AT4G23690 | -2.334802624 | 3.9706E-29 | 1.0361E-27 |
| AT4G23700 | -1.952701502 | 5.7067E-19 | 8.8979E-18 |
| AT4G23770 | -1.962941867 | 8.903E-05 | 0.00033537 |
| AT4G23800 | -1.0423982 | 1.7925E-16 | 2.3711E-15 |
| AT4G23820 | -1.259352798 | 5.1802E-16 | 6.6448E-15 |
| AT4G23840 | 1.291180039 | 3.4385E-09 | 2.3818E-08 |
| AT4G23870 | 2.69123159 | 3.4096E-37 | 1.2121E-35 |
| AT4G23880 | 2.894882628 | 2.912E-31 | 8.3099E-30 |
| AT4G23920 | 1.308294755 | 1.5629E-18 | 2.3674E-17 |
| AT4G24000 | 8.508381155 | 1.6917E-09 | 1.2168E-08 |
| AT4G24040 | 1.689319658 | 3.6916E-14 | 4.0884E-13 |
| AT4G24110 | 1.137638389 | 0.00169694 | 0.00490622 |
| AT4G24130 | 1.035423538 | 1.5482E-17 | 2.1885E-16 |
| AT4G24220 | 1.044027946 | 2.0197E-22 | 3.8993E-21 |
| AT4G24265 | -1.718129801 | 0.00058308 | 0.00186928 |
| AT4G24310 | -2.969434137 | 4.6715E-07 | 2.4853E-06 |
| AT4G24340 | -1.730972777 | 9.6535E-08 | 5.6507E-07 |
| AT4G24350 | -1.472020902 | 1.0234E-20 | 1.7748E-19 |
| AT4G24380 | 1.780805436 | 2.6463E-14 | 2.9565E-13 |
| AT4G24400 | 1.353642907 | 4.5172E-29 | 1.1747E-27 |
| AT4G24410 | 3.603484469 | 1.4731E-26 | 3.415E-25 |
| AT4G24413 | 2.949241763 | 4.0254E-45 | 1.8366E-43 |
| AT4G24420 | 3.801972388 | 2.0637E-10 | 1.6351E-09 |
| AT4G24480 | 1.110568852 | 3.569E-12 | 3.3393E-11 |
| AT4G24510 | 2.036715072 | 6.8785E-72 | 6.4175E-70 |
| AT4G24590 | 1.059668643 | 2.7878E-10 | 2.1741E-09 |
| AT4G24670 | -1.276398759 | 1.7687E-06 | 8.7208E-06 |
| AT4G24700 | -1.29674532 | 0.00489438 | 0.01274792 |
| AT4G24810 | -1.619240806 | 1.8104E-20 | 3.0858E-19 |
| AT4G24860 | 1.303504409 | 0.00647298 | 0.01633826 |
| AT4G24950 | 6.68019884 | 0.00088448 | 0.00273023 |
| AT4G24960 | 2.601413866 | 3.535E-126 | 7.624E-124 |
| AT4G25050 | -1.255467537 | 7.7196E-30 | 2.0857E-28 |
| AT4G25090 | -1.410502952 | 5.7981E-05 | 0.00022563 |
| AT4G25110 | -2.332024179 | 6.2629E-05 | 0.00024215 |
| AT4G25200 | 3.572370385 | 0.00132322 | 0.00392348 |
| AT4G25230 | 1.068341333 | 3.2264E-21 | 5.7933E-20 |
| AT4G25240 | -1.483909427 | 2.2653E-08 | 1.4399E-07 |
| AT4G25250 | -2.944791478 | 1.1038E-06 | 5.5867E-06 |
| AT4G25260 | -1.706010262 | 1.2984E-16 | 1.7316E-15 |
| AT4G25390 | 1.304439928 | 7.9152E-19 | 1.2233E-17 |
| AT4G25400 | -2.279338911 | 4.2811E-09 | 2.936E-08 |
| AT4G25433 | 3.224643847 | 1.177E-20 | 2.0289E-19 |
| AT4G25480 | 2.083135166 | 5.3177E-16 | 6.8173E-15 |
| AT4G25490 | 2.515046064 | 3.0635E-09 | 2.1336E-08 |
| AT4G25520 | 1.18326552 | 1.436E-18 | 2.1795E-17 |
| AT4G25560 | -5.454669035 | 0.00901103 | 0.02187956 |
| AT4G25650 | 1.219701804 | 1.3333E-23 | 2.7531E-22 |
| AT4G25670 | 2.402621502 | 1.6849E-85 | 2.1045E-83 |
| AT4G25690 | 1.819319697 | 2.2492E-48 | 1.145E-46 |
| AT4G25760 | -1.539887664 | 1.3243E-05 | 5.7515E-05 |
| AT4G25780 | -1.248318189 | 0.00130601 | 0.00387808 |
| AT4G25790 | -3.289735095 | 0.0068079 | 0.01707991 |
| AT4G25820 | -1.88723419 | 3.0241E-08 | 1.8933E-07 |
| AT4G25850 | 2.341918808 | 2.8335E-34 | 9.0081E-33 |
| AT4G25890 | -1.039628463 | 2.7899E-13 | 2.8791E-12 |
| AT4G25900 | -1.0256597 | 4.6348E-16 | 5.9686E-15 |
| AT4G25960 | 1.296881353 | 1.3875E-32 | 4.1675E-31 |
| AT4G26010 | -1.631201136 | 5.0255E-08 | 3.0535E-07 |
| AT4G26080 | 3.179800201 | 3.051E-255 | 2.79E-252 |
| AT4G26120 | -1.849889573 | 1.2413E-08 | 8.1307E-08 |
| AT4G26150 | -1.262413807 | 2.2688E-07 | 1.264E-06 |
| AT4G26180 | 1.104594953 | 6.1833E-06 | 2.816E-05 |
| AT4G26200 | 1.301222149 | 0.00168439 | 0.00487425 |
| AT4G26220 | -1.166331488 | 2.4103E-05 | 0.0001003 |
| AT4G26260 | -3.270092772 | 0.00651389 | 0.01641978 |
| AT4G26320 | -1.332289267 | 0.00105042 | 0.00318611 |
| AT4G26400 | 1.198459716 | 5.0193E-12 | 4.6338E-11 |
| AT4G26470 | -1.584197899 | 1.8314E-07 | 1.0307E-06 |
| AT4G26540 | -1.054192422 | 2.699E-06 | 1.298E-05 |
| AT4G26660 | -1.161294792 | 5.8531E-09 | 3.9408E-08 |
| AT4G26750 | 1.134949485 | 2.2563E-15 | 2.758E-14 |
| AT4G26780 | -1.648020548 | 5.9124E-20 | 9.779E-19 |
| AT4G27020 | 1.272915796 | 5.5109E-17 | 7.5295E-16 |
| AT4G27100 | 1.036229301 | 1.0507E-07 | 6.1172E-07 |
| AT4G27230 | -1.199790661 | 2.0835E-18 | 3.1311E-17 |
| AT4G27260 | 1.364128975 | 2.5176E-18 | 3.7588E-17 |
| AT4G27300 | -1.494267523 | 1.9994E-06 | 9.7799E-06 |
| AT4G27350 | 1.987213385 | 1.2706E-29 | 3.3928E-28 |
| AT4G27410 | 4.408968113 | 0 | 0 |
| AT4G27440 | -1.313570611 | 2.196E-39 | 8.3107E-38 |
| AT4G27520 | 1.721671754 | 3.4425E-40 | 1.3314E-38 |
| AT4G27530 | 3.833786027 | 7.9991E-20 | 1.3107E-18 |
| AT4G27595 | -1.00966742 | 1.7749E-08 | 1.1454E-07 |
| AT4G27652 | 1.33386493 | 2.7804E-08 | 1.746E-07 |
| AT4G27657 | 1.863637639 | 1.3319E-12 | 1.2972E-11 |
| AT4G27670 | 7.164473578 | 0.00012921 | 0.000472 |
| AT4G27680 | 1.020072158 | 2.5244E-15 | 3.066E-14 |
| AT4G27780 | 1.06895686 | 1.0412E-10 | 8.4879E-10 |
| AT4G27830 | 1.221071687 | 3.3607E-19 | 5.3199E-18 |
| AT4G27840 | 1.325457969 | 5.2386E-15 | 6.2302E-14 |
| AT4G27860 | -1.509064373 | 4.1569E-22 | 7.9117E-21 |
| AT4G27970 | -1.365119964 | 0.00767737 | 0.01898414 |
| AT4G28080 | -1.259780029 | 1.9887E-41 | 7.9471E-40 |
| AT4G28110 | 2.829961659 | 4.6647E-14 | 5.1336E-13 |
| AT4G28140 | 5.05487317 | 8.1354E-22 | 1.5205E-20 |
| AT4G28150 | 1.23987722 | 0.00023953 | 0.00082871 |
| AT4G28240 | 1.157072857 | 1.6111E-13 | 1.7042E-12 |
| AT4G28250 | -3.076435669 | 1.02E-116 | 1.865E-114 |
| AT4G28300 | 1.445401636 | 4.7339E-37 | 1.6724E-35 |
| AT4G28310 | -2.215664681 | 1.1596E-15 | 1.4492E-14 |
| AT4G28410 | -1.601334263 | 0.00080721 | 0.00251209 |
| AT4G28430 | -1.519130774 | 0.00031247 | 0.0010572 |
| AT4G28460 | -3.573424461 | 2.2978E-13 | 2.3907E-12 |
| AT4G28490 | 1.541506498 | 5.8883E-21 | 1.0339E-19 |
| AT4G28660 | -1.086883933 | 2.1845E-17 | 3.0558E-16 |
| AT4G28680 | -1.888957639 | 0.0008449 | 0.00261973 |
| AT4G28703 | 2.365517351 | 1.9155E-12 | 1.8412E-11 |
| AT4G28780 | -2.084343505 | 3.8101E-36 | 1.2883E-34 |
| AT4G28890 | -1.186730023 | 0.00578986 | 0.01480035 |
| AT4G28940 | -2.257754295 | 5.2046E-20 | 8.6459E-19 |
| AT4G28950 | -1.600839944 | 0.00023393 | 0.00081091 |
| AT4G29070 | 1.334678299 | 5.3273E-23 | 1.0607E-21 |
| AT4G29080 | -1.195961961 | 7.9122E-15 | 9.3033E-14 |
| AT4G29110 | 1.033847083 | 4.0593E-06 | 1.9021E-05 |
| AT4G29140 | -2.397160729 | 5.804E-27 | 1.3649E-25 |
| AT4G29190 | 2.446751044 | 1.902E-78 | 2.0222E-76 |
| AT4G29240 | -1.13712157 | 0.00011369 | 0.00042016 |
| AT4G29270 | -1.056155298 | 3.3465E-13 | 3.4318E-12 |
| AT4G29310 | -2.332145968 | 7.55E-16 | 9.5505E-15 |
| AT4G29380 | 1.314669181 | 1.5242E-17 | 2.156E-16 |
| AT4G29570 | 4.899884839 | 0.00115097 | 0.00345896 |
| AT4G29610 | -2.00300124 | 2.0064E-05 | 8.4584E-05 |
| AT4G29700 | -1.720229893 | 2.039E-36 | 7.0404E-35 |
| AT4G29720 | -1.507008766 | 9.7181E-18 | 1.3892E-16 |
| AT4G29780 | 1.275904233 | 9.4704E-08 | 5.5506E-07 |
| AT4G29800 | -1.269854046 | 0.0004121 | 0.00136005 |
| AT4G29820 | 1.194562598 | 4.5241E-09 | 3.0897E-08 |
| AT4G29950 | 1.909340877 | 7.6243E-61 | 5.4123E-59 |
| AT4G30170 | -2.058673467 | 7.9358E-06 | 3.5661E-05 |
| AT4G30230 | -2.385656379 | 2.6529E-06 | 1.2769E-05 |
| AT4G30250 | -1.514700152 | 0.00183411 | 0.00525893 |
| AT4G30330 | -1.093306727 | 2.2363E-07 | 1.2468E-06 |
| AT4G30350 | 1.070682392 | 1.4486E-19 | 2.3384E-18 |
| AT4G30420 | -2.803532541 | 0.00194699 | 0.00555539 |
| AT4G30460 | 1.749768647 | 1.2636E-47 | 6.3064E-46 |
| AT4G30470 | 1.500495594 | 1.5896E-51 | 8.8194E-50 |
| AT4G30490 | 2.277904077 | 1.7004E-76 | 1.7352E-74 |
| AT4G30600 | 1.272246757 | 3.6185E-36 | 1.229E-34 |
| AT4G30610 | -2.130434246 | 6.9729E-22 | 1.3097E-20 |
| AT4G30670 | -1.300528247 | 9.4065E-09 | 6.2341E-08 |
| AT4G30710 | 1.14897352 | 7.6241E-11 | 6.3302E-10 |
| AT4G30780 | 1.204646626 | 1.8949E-10 | 1.5045E-09 |
| AT4G30800 | -1.372161331 | 3.5866E-10 | 2.7632E-09 |
| AT4G30825 | -1.056172866 | 5.1549E-08 | 3.1263E-07 |
| AT4G30830 | 2.176120181 | 4.895E-09 | 3.328E-08 |
| AT4G30940 | 1.413554541 | 5.2911E-07 | 2.7906E-06 |
| AT4G30960 | 2.052179621 | 8.882E-108 | 1.515E-105 |
| AT4G30980 | -1.052137742 | 4.4242E-06 | 2.0596E-05 |
| AT4G31100 | -1.44935707 | 0.00013617 | 0.00049485 |
| AT4G31140 | 1.447331819 | 5.517E-19 | 8.6197E-18 |
| AT4G31290 | 1.69992864 | 5.1591E-62 | 3.7676E-60 |
| AT4G31320 | -1.937874847 | 3.9189E-09 | 2.6997E-08 |
| AT4G31354 | 3.00306273 | 0.00330088 | 0.00894506 |
| AT4G31360 | -1.433973666 | 9.3245E-10 | 6.8999E-09 |
| AT4G31470 | -3.465155714 | 1.842E-05 | 7.803E-05 |
| AT4G31500 | -1.22812571 | 6.673E-23 | 1.3172E-21 |
| AT4G31600 | -1.378867252 | 1.7752E-09 | 1.272E-08 |
| AT4G31670 | 1.117122994 | 1.8705E-10 | 1.4872E-09 |
| AT4G31730 | -1.189811717 | 8.1715E-10 | 6.0921E-09 |
| AT4G31750 | 1.497543842 | 2.4785E-29 | 6.5722E-28 |
| AT4G31760 | 2.54956007 | 9.8693E-06 | 4.3749E-05 |
| AT4G31780 | 1.232565824 | 5.4256E-35 | 1.7717E-33 |
| AT4G31800 | 2.293430666 | 4.213E-45 | 1.9183E-43 |
| AT4G31805 | -1.192884221 | 0.00037702 | 0.00125517 |
| AT4G31840 | -1.229725859 | 3.1247E-11 | 2.6852E-10 |
| AT4G31850 | -1.022013268 | 1.0659E-11 | 9.5393E-11 |
| AT4G31860 | 2.461217932 | 1.209E-122 | 2.49E-120 |
| AT4G31870 | 1.317362099 | 0.00496685 | 0.01290138 |
| AT4G31890 | -1.501033024 | 5.2314E-09 | 3.5389E-08 |
| AT4G31910 | -2.459139348 | 3.3631E-34 | 1.0647E-32 |
| AT4G31970 | -9.650677746 | 1.1728E-20 | 2.0247E-19 |
| AT4G32070 | 1.492244155 | 2.8638E-41 | 1.1404E-39 |
| AT4G32190 | 1.370001725 | 5.9151E-22 | 1.1156E-20 |
| AT4G32250 | 1.675745309 | 1.4496E-35 | 4.816E-34 |
| AT4G32295 | 1.154929082 | 9.6881E-07 | 4.9376E-06 |
| AT4G32300 | 1.332747913 | 9.461E-23 | 1.8563E-21 |
| AT4G32460 | -1.256626936 | 2.1634E-09 | 1.5329E-08 |
| AT4G32570 | -1.01175864 | 2.2473E-10 | 1.7695E-09 |
| AT4G32605 | -1.183905777 | 1.2812E-10 | 1.0359E-09 |
| AT4G32630 | 4.649883036 | 3.9034E-05 | 0.00015675 |
| AT4G32770 | 1.287084788 | 3.9912E-15 | 4.799E-14 |
| AT4G32780 | -2.998514202 | 4.3906E-08 | 2.6957E-07 |
| AT4G32810 | -3.261891697 | 0.00053451 | 0.00172836 |
| AT4G32860 | -2.346974374 | 8.9943E-06 | 4.0108E-05 |
| AT4G32890 | -2.032205525 | 6.7137E-09 | 4.4937E-08 |
| AT4G32920 | 1.517065918 | 6.905E-37 | 2.432E-35 |
| AT4G32940 | 1.421747434 | 6.8759E-42 | 2.8217E-40 |
| AT4G32950 | -1.404375693 | 0.00011795 | 0.00043428 |
| AT4G32980 | -1.232505826 | 2.1475E-13 | 2.2425E-12 |
| AT4G33000 | -1.005569976 | 9.2818E-06 | 4.1325E-05 |
| AT4G33020 | -4.963096184 | 8.7212E-05 | 0.00032912 |
| AT4G33040 | 1.346894509 | 1.6329E-11 | 1.4389E-10 |
| AT4G33080 | 1.213255286 | 1.4168E-27 | 3.3804E-26 |
| AT4G33150 | 2.277649597 | 2.1065E-45 | 9.7468E-44 |
| AT4G33240 | 1.308254231 | 3.3038E-28 | 8.2085E-27 |
| AT4G33465 | 6.7036254 | 0.00075345 | 0.00235534 |
| AT4G33467 | 6.310232561 | 3.666E-121 | 7.482E-119 |
| AT4G33540 | 2.153617152 | 3.3106E-53 | 1.9158E-51 |
| AT4G33700 | 1.143839 | 9.6319E-22 | 1.7944E-20 |
| AT4G33730 | -6.54319932 | 0.00022022 | 0.0007671 |
| AT4G33790 | -2.459331736 | 0.00035699 | 0.00119421 |
| AT4G33905 | 4.918821149 | 5.252E-239 | 4.14E-236 |
| AT4G33930 | 7.003611004 | 8.627E-107 | 1.461E-104 |
| AT4G33940 | 1.67093858 | 2.7778E-31 | 7.9486E-30 |
| AT4G33950 | 1.589269008 | 1.7368E-34 | 5.5603E-33 |
| AT4G34000 | 2.415994923 | 1.832E-110 | 3.246E-108 |
| AT4G34131 | 1.033350584 | 2.0683E-15 | 2.5377E-14 |
| AT4G34160 | -1.47641055 | 2.0989E-16 | 2.762E-15 |
| AT4G34210 | 6.633072981 | 3.8663E-06 | 1.8166E-05 |
| AT4G34220 | -1.235305759 | 1.1948E-14 | 1.3759E-13 |
| AT4G34230 | 1.880686287 | 2.8438E-81 | 3.2502E-79 |
| AT4G34290 | -1.264963712 | 3.4246E-21 | 6.1395E-20 |
| AT4G34300 | 3.631410474 | 0.00245973 | 0.00688098 |
| AT4G34310 | 1.082900107 | 1.3514E-12 | 1.3156E-11 |
| AT4G34340 | 1.107549116 | 8.318E-05 | 0.0003151 |
| AT4G34370 | 1.239622001 | 3.3768E-16 | 4.3832E-15 |
| AT4G34410 | 5.256537971 | 0.00754022 | 0.01868339 |
| AT4G34419 | -4.325251879 | 0.00162836 | 0.00473068 |
| AT4G34470 | 5.773972669 | 6.4092E-06 | 2.9132E-05 |
| AT4G34510 | -3.071915203 | 8.5463E-05 | 0.000323 |
| AT4G34650 | 2.282047361 | 1.5244E-46 | 7.2593E-45 |
| AT4G34710 | 2.259329364 | 7.484E-127 | 1.645E-124 |
| AT4G34760 | -1.608943566 | 6.4585E-06 | 2.9344E-05 |
| AT4G34860 | 1.810740769 | 1.0391E-39 | 3.9587E-38 |
| AT4G34890 | 1.306435523 | 1.1919E-25 | 2.6815E-24 |
| AT4G34950 | -1.350274993 | 0.00013424 | 0.00048844 |
| AT4G34980 | -1.240288926 | 2.5443E-21 | 4.6011E-20 |
| AT4G35030 | -1.184721069 | 1.2977E-05 | 5.6423E-05 |
| AT4G35060 | -1.672847804 | 2.6964E-14 | 3.008E-13 |
| AT4G35110 | 1.299888655 | 7.0453E-14 | 7.6613E-13 |
| AT4G35140 | 1.236601339 | 8.1782E-12 | 7.3946E-11 |
| AT4G35190 | 3.355583767 | 1.0781E-42 | 4.4969E-41 |
| AT4G35200 | -4.207343601 | 0.00269962 | 0.00747068 |
| AT4G35300 | 1.341207243 | 3.4784E-35 | 1.1457E-33 |
| AT4G35390 | -1.21900032 | 0.00098629 | 0.00301399 |
| AT4G35480 | 1.428597377 | 2.0141E-08 | 1.2889E-07 |
| AT4G35510 | 1.535856064 | 1.005E-23 | 2.0826E-22 |
| AT4G35550 | 1.073562788 | 6.5965E-10 | 4.9714E-09 |
| AT4G35560 | 2.011527108 | 5.8577E-41 | 2.3006E-39 |
| AT4G09215 | -2.275908253 | 0.00012265 | 0.00045022 |
| AT4G35690 | 3.57019354 | 1.8287E-12 | 1.7615E-11 |
| AT4G35720 | 2.156222614 | 2.1313E-31 | 6.1513E-30 |
| AT4G35783 | 2.138222003 | 0.00035814 | 0.00119737 |
| AT4G35790 | 1.794181664 | 3.5163E-76 | 3.5564E-74 |
| AT4G35810 | -1.474788441 | 0.00033766 | 0.00113469 |
| AT4G35860 | 1.314665964 | 6.4239E-23 | 1.2713E-21 |
| AT4G35940 | 1.138586285 | 1.0063E-09 | 7.4203E-09 |
| AT4G36010 | 2.142647653 | 4.3573E-44 | 1.9228E-42 |
| AT4G36040 | 1.76432873 | 4.3215E-38 | 1.5704E-36 |
| AT4G36090 | 1.271337343 | 1.4702E-16 | 1.9561E-15 |
| AT4G36120 | -1.097056577 | 0.00417636 | 0.01105153 |
| AT4G36210 | 1.319317803 | 1.7635E-34 | 5.6298E-33 |
| AT4G36380 | 1.084920215 | 5.6523E-10 | 4.2824E-09 |
| AT4G36430 | -1.855197693 | 2.1333E-19 | 3.4101E-18 |
| AT4G36450 | 2.973226968 | 4.9569E-19 | 7.766E-18 |
| AT4G36490 | 4.677183391 | 0.00192973 | 0.00551027 |
| AT4G36540 | -2.007491233 | 1.4605E-27 | 3.4812E-26 |
| AT4G36570 | -8.229268693 | 1.5834E-07 | 8.9855E-07 |
| AT4G36700 | 1.791843851 | 0.00538283 | 0.01386375 |
| AT4G36720 | 1.047474754 | 0.00019738 | 0.00069496 |
| AT4G36740 | 2.465928837 | 1.4117E-27 | 3.3719E-26 |
| AT4G36760 | 1.294369424 | 7.3342E-24 | 1.5352E-22 |
| AT4G36820 | 1.821484876 | 0.00010777 | 0.00040037 |
| AT4G36900 | 1.857588984 | 5.8425E-23 | 1.1593E-21 |
| AT4G36920 | -1.157762539 | 2.0805E-12 | 1.9923E-11 |
| AT4G36950 | 4.183868292 | 2.008E-18 | 3.0257E-17 |
| AT4G37010 | -1.255717836 | 6.6249E-07 | 3.4518E-06 |
| AT4G37022 | 1.69138105 | 0.00304374 | 0.0083083 |
| AT4G37060 | -4.219466381 | 0.0029965 | 0.00819503 |
| AT4G37070 | -2.298152043 | 2.1501E-20 | 3.6486E-19 |
| AT4G37080 | -1.335364911 | 3.919E-18 | 5.7906E-17 |
| AT4G37160 | -3.365518482 | 2.6041E-11 | 2.2573E-10 |
| AT4G37180 | 1.229135221 | 2.557E-21 | 4.6204E-20 |
| AT4G37220 | 4.210658282 | 3.4852E-26 | 8.0144E-25 |
| AT4G37260 | 1.189892346 | 4.0941E-22 | 7.7985E-21 |
| AT4G37290 | -5.913041844 | 3.9588E-29 | 1.0342E-27 |
| AT4G37340 | -2.589971925 | 9.4006E-05 | 0.00035278 |
| AT4G37370 | 2.824900862 | 1.1469E-77 | 1.2025E-75 |
| AT4G37380 | -1.015444343 | 0.00029111 | 0.00099078 |
| AT4G37410 | -1.307711315 | 2.5722E-14 | 2.8765E-13 |
| AT4G37420 | 2.284586707 | 0.00663015 | 0.01667974 |
| AT4G37520 | -2.747432482 | 3.0193E-83 | 3.6324E-81 |
| AT4G37530 | -2.567917216 | 4.3331E-30 | 1.1834E-28 |
| AT4G37650 | -1.23649098 | 9.8115E-11 | 8.0297E-10 |
| AT4G37680 | 1.011433036 | 5.4754E-15 | 6.505E-14 |
| AT4G37700 | -3.367983542 | 3.223E-05 | 0.0001313 |
| AT4G37750 | -1.343770753 | 8.2774E-18 | 1.1922E-16 |
| AT4G37760 | 1.293920285 | 5.4066E-25 | 1.1804E-23 |
| AT4G37790 | 1.525252952 | 1.7074E-27 | 4.0613E-26 |
| AT4G37800 | -1.761967106 | 7.0189E-26 | 1.5901E-24 |
| AT4G37925 | -1.376311447 | 1.9897E-23 | 4.0499E-22 |
| AT4G37990 | 5.664313159 | 2.248E-239 | 1.835E-236 |
| AT4G38060 | 1.195791962 | 1.2344E-14 | 1.42E-13 |
| AT4G38080 | -1.210322954 | 2.5072E-16 | 3.2823E-15 |
| AT4G38160 | -1.387646207 | 3.0502E-23 | 6.1375E-22 |
| AT4G38400 | 1.97622893 | 2.4928E-17 | 3.4766E-16 |
| AT4G38420 | -1.833907723 | 2.1007E-12 | 2.0108E-11 |
| AT4G38520 | -1.062930941 | 1.6033E-20 | 2.741E-19 |
| AT4G38580 | 1.284965458 | 9.3687E-24 | 1.9521E-22 |
| AT4G38620 | -1.352623032 | 1.3641E-22 | 2.6492E-21 |
| AT4G38660 | -2.296437273 | 6.837E-52 | 3.8304E-50 |
| AT4G38700 | -2.652276311 | 0.00116092 | 0.0034852 |
| AT4G38730 | 2.568441028 | 1.3677E-48 | 7.0569E-47 |
| AT4G38810 | 1.519212193 | 3.7179E-41 | 1.4703E-39 |
| AT4G38830 | -3.234172532 | 0.00511485 | 0.01324969 |
| AT4G38840 | -2.497231639 | 2.1809E-21 | 3.9595E-20 |
| AT4G38860 | -3.538185678 | 1.961E-08 | 1.2574E-07 |
| AT4G38940 | 1.260475321 | 1.9468E-08 | 1.2489E-07 |
| AT4G38950 | -1.298710318 | 4.6622E-19 | 7.3092E-18 |
| AT4G39070 | -2.900550698 | 9.6334E-24 | 2.0036E-22 |
| AT4G39090 | 1.354501938 | 1.4417E-48 | 7.4223E-47 |
| AT4G09805 | -1.459446407 | 6.0468E-06 | 2.7594E-05 |
| AT4G39140 | 1.160712952 | 7.7734E-15 | 9.1542E-14 |
| AT4G39210 | 1.945802014 | 3.2697E-61 | 2.3429E-59 |
| AT4G39250 | -5.599843579 | 0.0057288 | 0.01465738 |
| AT4G39300 | -1.014219919 | 4.2889E-07 | 2.2935E-06 |
| AT4G39330 | 1.476130386 | 1.0639E-27 | 2.5625E-26 |
| AT4G39340 | 3.249079834 | 4.1551E-29 | 1.083E-27 |
| AT4G39345 | 7.316802051 | 5.9408E-05 | 0.00023071 |
| AT4G39360 | 3.861183617 | 2.7941E-11 | 2.411E-10 |
| AT4G39670 | 1.785411585 | 1.6651E-17 | 2.348E-16 |
| AT4G39700 | 1.792347155 | 0.00064385 | 0.00204548 |
| AT4G39720 | -1.462042273 | 0.00151434 | 0.00443269 |
| AT4G39770 | -2.642310012 | 1.6389E-09 | 1.1816E-08 |
| AT4G39795 | -1.820894552 | 1.7077E-05 | 7.2783E-05 |
| AT4G39940 | -2.043873975 | 1.0386E-73 | 1.0189E-71 |
| AT4G39950 | -2.598932131 | 1.5567E-93 | 2.2102E-91 |
| AT4G40010 | 4.192971264 | 1.719E-155 | 5.17E-153 |
| AT4G40020 | -1.821456885 | 0.00033 | 0.00111108 |
| AT4G40070 | 1.542746332 | 2.245E-05 | 9.3866E-05 |
| AT4G40090 | -2.881542838 | 1.0869E-14 | 1.2561E-13 |
| AT5G01015 | -2.55319113 | 0.0066612 | 0.01675046 |
| AT5G01050 | -2.103347251 | 0.00120638 | 0.00360793 |
| AT5G01100 | 2.77429293 | 1.9821E-55 | 1.2082E-53 |
| AT5G01200 | 1.551959649 | 1.146E-10 | 9.3086E-10 |
| AT5G01240 | -1.016847176 | 1.3714E-11 | 1.2165E-10 |
| AT5G01250 | -1.648926791 | 1.8968E-05 | 8.0215E-05 |
| AT5G01260 | 1.086418247 | 5.0026E-14 | 5.495E-13 |
| AT5G01270 | 1.263988727 | 4.7987E-21 | 8.5295E-20 |
| AT5G01280 | -3.028621311 | 0.00250962 | 0.00700256 |
| AT5G01300 | 3.553574628 | 0.00086115 | 0.00266543 |
| AT5G01340 | 1.364423402 | 5.179E-20 | 8.6158E-19 |
| AT5G01360 | -2.000341013 | 3.0688E-05 | 0.00012539 |
| AT5G01380 | 1.970467308 | 8.5167E-07 | 4.3806E-06 |
| AT5G01490 | -1.958490299 | 9.1437E-05 | 0.00034371 |
| AT5G01520 | 4.170015428 | 2.204E-138 | 5.537E-136 |
| AT5G01620 | -1.059715216 | 1.0052E-05 | 4.4462E-05 |
| AT5G01790 | -1.668789161 | 5.197E-13 | 5.2447E-12 |
| AT5G01820 | 1.017585702 | 1.542E-15 | 1.9167E-14 |
| AT5G01880 | 2.027761654 | 4.4315E-20 | 7.3939E-19 |
| AT5G01900 | 6.539545382 | 0.0013527 | 0.00400208 |
| AT5G01910 | -1.488364481 | 6.7801E-06 | 3.0744E-05 |
| AT5G01990 | 1.630290517 | 5.0664E-21 | 8.9844E-20 |
| AT5G02020 | 4.308703414 | 2.7457E-09 | 1.9211E-08 |
| AT5G02090 | -1.918384452 | 1.7788E-06 | 8.7649E-06 |
| AT5G02190 | -1.023342325 | 0.00502202 | 0.01303312 |
| AT5G02270 | -1.153705592 | 4.405E-28 | 1.0838E-26 |
| AT5G02320 | 1.610797376 | 3.4591E-12 | 3.2418E-11 |
| AT5G02360 | -2.206134768 | 0.00491449 | 0.01278863 |
| AT5G02430 | 1.2415567 | 7.6655E-07 | 3.9669E-06 |
| AT5G02540 | -1.541854955 | 0.00058453 | 0.00187341 |
| AT5G02550 | -1.168946054 | 5.9714E-05 | 0.00023186 |
| AT5G02760 | -1.98858554 | 5.0478E-07 | 2.6709E-06 |
| AT5G02780 | -2.808228166 | 2.8583E-19 | 4.5434E-18 |
| AT5G02830 | -1.00139932 | 9.9443E-14 | 1.0727E-12 |
| AT5G02880 | 1.238309692 | 2.2211E-18 | 3.3291E-17 |
| AT5G03020 | 2.330216808 | 0.00770831 | 0.01905033 |
| AT5G03030 | 1.942676147 | 1.4773E-43 | 6.4198E-42 |
| AT5G03080 | 1.056970138 | 1.9319E-16 | 2.5481E-15 |
| AT5G03120 | -1.586133352 | 0.00208077 | 0.00591055 |
| AT5G03204 | 5.386115703 | 1.4858E-11 | 1.3143E-10 |
| AT5G03210 | 5.224415199 | 1.8721E-31 | 5.4099E-30 |
| AT5G03230 | 1.430773009 | 6.6158E-14 | 7.2081E-13 |
| AT5G03340 | 1.196105559 | 2.1641E-20 | 3.6697E-19 |
| AT5G03350 | -2.161737595 | 4.8585E-05 | 0.00019157 |
| AT5G03355 | -3.702496711 | 2.5299E-05 | 0.00010503 |
| AT5G03390 | -1.481091428 | 7.3152E-05 | 0.00027957 |
| AT5G03495 | 1.54068685 | 0.0011008 | 0.0033226 |
| AT5G03553 | -1.742069378 | 0.0004768 | 0.0015563 |
| AT5G03640 | -4.014730696 | 0.00011904 | 0.00043801 |
| AT5G03720 | 1.701229713 | 2.1883E-10 | 1.7254E-09 |
| AT5G03870 | -1.334148298 | 4.3757E-06 | 2.0396E-05 |
| AT5G03890 | 1.801233261 | 2.6486E-08 | 1.6683E-07 |
| AT5G03995 | -3.079543375 | 0.00023435 | 0.00081212 |
| AT5G04010 | 4.585804961 | 2.7465E-05 | 0.00011312 |
| AT5G04080 | 1.54197183 | 1.0058E-19 | 1.6386E-18 |
| AT5G04120 | -2.256700822 | 0.00201381 | 0.00573388 |
| AT5G04230 | -2.567035897 | 3.1355E-21 | 5.6345E-20 |
| AT5G04235 | 1.502188496 | 7.531E-12 | 6.8176E-11 |
| AT5G04250 | 3.628025988 | 5.273E-146 | 1.452E-143 |
| AT5G04340 | 3.899702169 | 2.189E-229 | 1.39E-226 |
| AT5G04370 | 1.874395591 | 4.2562E-13 | 4.3297E-12 |
| AT5G04380 | 6.188813654 | 1.9708E-28 | 4.9505E-27 |
| AT5G04410 | 1.186552631 | 4.2928E-30 | 1.1737E-28 |
| AT5G04460 | 1.045152719 | 1.3245E-10 | 1.0683E-09 |
| AT5G04470 | -1.800087871 | 1.3367E-05 | 5.8024E-05 |
| AT5G04720 | 1.241022142 | 8.7429E-17 | 1.1804E-15 |
| AT5G04760 | 2.794163329 | 2.9461E-44 | 1.3076E-42 |
| AT5G04850 | 1.014906773 | 1.8697E-13 | 1.9659E-12 |
| AT5G04950 | -2.332658785 | 1.9518E-24 | 4.1657E-23 |
| AT5G04960 | -4.169953179 | 9.7795E-22 | 1.8189E-20 |
| AT5G04970 | -2.662402385 | 8.5118E-09 | 5.6592E-08 |
| AT5G05100 | 1.473773297 | 4.6214E-32 | 1.3613E-30 |
| AT5G05190 | 1.041832228 | 2.5921E-05 | 0.0001074 |
| AT5G05220 | 7.273511271 | 1.783E-268 | 1.772E-265 |
| AT5G05250 | 1.575264951 | 8.6545E-21 | 1.5078E-19 |
| AT5G05260 | -5.672927654 | 0.00458947 | 0.01202085 |
| AT5G05270 | -1.55320189 | 7.763E-56 | 4.8088E-54 |
| AT5G05340 | 2.683142164 | 2.2074E-18 | 3.3108E-17 |
| AT5G05410 | 3.321988827 | 7.109E-183 | 3.066E-180 |
| AT5G05490 | 2.667195908 | 3.338E-05 | 0.0001355 |
| AT5G05500 | -4.671563581 | 2.2901E-06 | 1.1107E-05 |
| AT5G05580 | -1.074527297 | 1.0021E-16 | 1.349E-15 |
| AT5G05600 | 2.504457733 | 2.303E-116 | 4.145E-114 |
| AT5G05810 | -1.663052253 | 0.00072283 | 0.00226832 |
| AT5G05840 | 1.037400981 | 0.00127915 | 0.00380663 |
| AT5G05850 | 1.467403265 | 9.8036E-10 | 7.2404E-09 |
| AT5G05870 | 1.070450243 | 2.6849E-09 | 1.8814E-08 |
| AT5G05890 | -1.836687359 | 8.7971E-12 | 7.9386E-11 |
| AT5G05940 | -1.044191716 | 0.00099493 | 0.00303682 |
| AT5G05960 | -2.422447151 | 4.6426E-39 | 1.7172E-37 |
| AT5G06090 | 1.696061388 | 8.8035E-14 | 9.519E-13 |
| AT5G06150 | -1.034445289 | 4.5508E-07 | 2.4265E-06 |
| AT5G06200 | -3.715611172 | 8.6931E-10 | 6.4594E-09 |
| AT5G06250 | -2.0858663 | 0.00148866 | 0.00436591 |
| AT5G06270 | -1.010117612 | 1.2244E-05 | 5.3504E-05 |
| AT5G06370 | 1.166859014 | 3.918E-20 | 6.561E-19 |
| AT5G06430 | -1.022565036 | 0.00559546 | 0.01435799 |
| AT5G06530 | 2.947804827 | 4.8296E-79 | 5.2569E-77 |
| AT5G06630 | -3.772989241 | 5.2012E-12 | 4.792E-11 |
| AT5G06640 | -2.432338908 | 7.9513E-09 | 5.2958E-08 |
| AT5G06740 | -2.1105483 | 3.2048E-08 | 1.9993E-07 |
| AT5G06760 | 5.639454552 | 3.8834E-23 | 7.7728E-22 |
| AT5G06790 | -1.718230466 | 3.7111E-06 | 1.7497E-05 |
| AT5G06839 | 1.219516723 | 4.6691E-09 | 3.1821E-08 |
| AT5G06930 | -1.137899117 | 0.00146355 | 0.00429788 |
| AT5G06980 | 2.331241947 | 9.6104E-07 | 4.9024E-06 |
| AT5G07010 | 2.187003492 | 2.365E-38 | 8.6494E-37 |
| AT5G07030 | -1.154144 | 2.469E-24 | 5.2451E-23 |
| AT5G07070 | 1.030072529 | 0.00028914 | 0.00098453 |
| AT5G07110 | -1.439488182 | 0.00213746 | 0.00605728 |
| AT5G07130 | -1.763554418 | 4.2319E-12 | 3.9322E-11 |
| AT5G07190 | -3.906920717 | 4.9395E-12 | 4.5656E-11 |
| AT5G07290 | 1.000733035 | 3.3256E-15 | 4.0242E-14 |
| AT5G07322 | -1.391743187 | 9.0949E-07 | 4.6529E-06 |
| AT5G07330 | 3.131752059 | 1.567E-05 | 6.7229E-05 |
| AT5G07390 | -2.146704804 | 2.6515E-08 | 1.6696E-07 |
| AT5G07460 | -1.361903613 | 5.7384E-18 | 8.3814E-17 |
| AT5G07500 | 5.958898877 | 0.00897829 | 0.02180702 |
| AT5G07580 | -2.019108135 | 7.3709E-24 | 1.5415E-22 |
| AT5G07650 | -2.775830676 | 3.6176E-06 | 1.7075E-05 |
| AT5G07690 | -2.065284003 | 2.9143E-25 | 6.43E-24 |
| AT5G07700 | -1.826498046 | 0.00153123 | 0.00447752 |
| AT5G07740 | 1.677398692 | 2.0094E-44 | 8.9536E-43 |
| AT5G07780 | -4.682173683 | 3.8569E-07 | 2.0758E-06 |
| AT5G07880 | 3.106423667 | 1.8753E-05 | 7.9335E-05 |
| AT5G07900 | -1.150459955 | 6.6734E-05 | 0.00025672 |
| AT5G07920 | 2.013888178 | 6.9707E-47 | 3.3902E-45 |
| AT5G07990 | -1.676417345 | 5.7352E-41 | 2.2564E-39 |
| AT5G08020 | -1.7083338 | 3.6027E-12 | 3.3681E-11 |
| AT5G08030 | 7.797501978 | 5.5171E-06 | 2.5333E-05 |
| AT5G08330 | -2.156006248 | 3.7399E-23 | 7.4989E-22 |
| AT5G08370 | -1.447700974 | 3.3917E-16 | 4.3999E-15 |
| AT5G08640 | -2.555647336 | 1.165E-156 | 3.598E-154 |
| AT5G08790 | 1.201120929 | 2.4417E-32 | 7.2958E-31 |
| AT5G09430 | 2.478162636 | 8.3779E-07 | 4.3151E-06 |
| AT5G09440 | 1.536142871 | 3.8414E-37 | 1.3614E-35 |
| AT5G09460 | -1.361951654 | 3.6695E-26 | 8.4299E-25 |
| AT5G09470 | 4.118277353 | 4.5682E-08 | 2.7927E-07 |
| AT5G09570 | 1.375283523 | 0.00014237 | 0.00051524 |
| AT5G09610 | 5.94454825 | 1.0437E-15 | 1.3094E-14 |
| AT5G09620 | 1.918298126 | 9.5174E-85 | 1.1759E-82 |
| AT5G09820 | -1.40556349 | 3.0029E-12 | 2.8363E-11 |
| AT5G09980 | 1.100224939 | 0.00231087 | 0.00650097 |
| AT5G09995 | -1.37159854 | 4.8224E-08 | 2.9355E-07 |
| AT5G10120 | -5.630706359 | 0.00699116 | 0.01747661 |
| AT5G10130 | -2.086971094 | 8.58E-17 | 1.1598E-15 |
| AT5G10230 | -1.118361348 | 0.00013721 | 0.00049829 |
| AT5G10300 | 2.431859674 | 6.2038E-50 | 3.3288E-48 |
| AT5G10340 | 4.72250899 | 6.3752E-07 | 3.3309E-06 |
| AT5G10390 | -1.433972133 | 4.6502E-15 | 5.5652E-14 |
| AT5G10430 | -2.236525558 | 0.00138184 | 0.0040793 |
| AT5G10480 | 1.218744394 | 1.1606E-31 | 3.3667E-30 |
| AT5G10625 | 1.798384752 | 1.4563E-31 | 4.2137E-30 |
| AT5G10650 | 1.224926677 | 2.4212E-12 | 2.3041E-11 |
| AT5G10740 | 1.003732125 | 2.9184E-06 | 1.3982E-05 |
| AT5G10770 | -2.587118707 | 1.3692E-30 | 3.8121E-29 |
| AT5G10850 | -1.043733543 | 0.00745138 | 0.01849333 |
| AT5G10930 | 2.698059 | 3.6511E-83 | 4.3467E-81 |
| AT5G00365 | 3.207361222 | 2.6062E-09 | 1.8296E-08 |
| AT5G11110 | 3.029419609 | 1.027E-193 | 5.219E-191 |
| AT5G11160 | -1.790433607 | 1.1051E-07 | 6.4132E-07 |
| AT5G11390 | 1.149128184 | 4.2667E-13 | 4.3385E-12 |
| AT5G11420 | -2.026390281 | 1.1829E-32 | 3.567E-31 |
| AT5G11520 | 1.096804898 | 6.494E-26 | 1.4726E-24 |
| AT5G11540 | -1.258214053 | 0.0040918 | 0.01084534 |
| AT5G11590 | -2.526948769 | 4.8888E-09 | 3.3248E-08 |
| AT5G11610 | -1.971252199 | 4.5576E-06 | 2.1187E-05 |
| AT5G11630 | -1.055793457 | 1.2101E-05 | 5.2961E-05 |
| AT5G11650 | 1.436937006 | 1.3939E-22 | 2.7048E-21 |
| AT5G11700 | 1.240037495 | 5.8354E-26 | 1.3272E-24 |
| AT5G11740 | 1.144184659 | 5.0255E-34 | 1.5845E-32 |
| AT5G11750 | -1.32876259 | 1.7518E-09 | 1.2573E-08 |
| AT5G11920 | -1.547002637 | 0.0012746 | 0.00379459 |
| AT5G11930 | -2.606020618 | 0.00160893 | 0.0046796 |
| AT5G12010 | 1.764549888 | 1.151E-47 | 5.7569E-46 |
| AT5G12020 | 4.25625515 | 7.4907E-37 | 2.6221E-35 |
| AT5G12030 | 3.361112163 | 7.979E-34 | 2.4984E-32 |
| AT5G12110 | -1.602091375 | 4.1458E-10 | 3.1757E-09 |
| AT5G12270 | -4.248801057 | 1.0189E-13 | 1.0981E-12 |
| AT5G12280 | -1.575799988 | 8.2252E-05 | 0.00031169 |
| AT5G12340 | -2.088949054 | 8.6433E-10 | 6.428E-09 |
| AT5G12400 | 1.222692877 | 1.8585E-19 | 2.9791E-18 |
| AT5G12840 | 1.707147062 | 6.7401E-32 | 1.9701E-30 |
| AT5G12900 | -1.516513221 | 8.9579E-15 | 1.0436E-13 |
| AT5G12970 | -1.096763728 | 0.00111651 | 0.00336469 |
| AT5G13080 | 2.093228354 | 7.8858E-18 | 1.1365E-16 |
| AT5G13110 | 1.331876196 | 6.9851E-29 | 1.796E-27 |
| AT5G13150 | -1.654808112 | 0.00019957 | 0.00070212 |
| AT5G13170 | 5.235090467 | 8.585E-138 | 2.133E-135 |
| AT5G13180 | 1.09274944 | 3.2485E-20 | 5.472E-19 |
| AT5G13200 | 2.098897953 | 6.2827E-53 | 3.5902E-51 |
| AT5G13205 | 2.532478997 | 6.3694E-13 | 6.3688E-12 |
| AT5G13210 | 1.933206658 | 4.2795E-13 | 4.3457E-12 |
| AT5G13220 | 2.251688198 | 8.5719E-20 | 1.4036E-18 |
| AT5G13310 | 1.527551983 | 1.024E-21 | 1.9029E-20 |
| AT5G13320 | -1.252651685 | 0.0002017 | 0.00070918 |
| AT5G13330 | 2.440203129 | 4.7799E-59 | 3.223E-57 |
| AT5G13370 | 1.404107172 | 7.4509E-33 | 2.2678E-31 |
| AT5G13490 | 1.246315145 | 9.6955E-24 | 2.0147E-22 |
| AT5G13510 | -1.032454102 | 1.2468E-12 | 1.2184E-11 |
| AT5G13550 | 1.161294555 | 1.1678E-18 | 1.7867E-17 |
| AT5G13655 | 6.354291718 | 0.00295509 | 0.00810246 |
| AT5G13700 | 2.719215458 | 1.6333E-26 | 3.7825E-25 |
| AT5G13740 | 1.32074561 | 3.528E-28 | 8.7369E-27 |
| AT5G13750 | 1.749360871 | 3.136E-70 | 2.8111E-68 |
| AT5G13760 | 1.133403269 | 2.3307E-10 | 1.8308E-09 |
| AT5G13800 | 1.580075578 | 8.5907E-40 | 3.2837E-38 |
| AT5G13820 | 2.092609233 | 2.2143E-33 | 6.8584E-32 |
| AT5G13880 | 2.174427847 | 2.1511E-16 | 2.8291E-15 |
| AT5G13910 | -1.180778381 | 0.00491679 | 0.01279055 |
| AT5G13930 | -1.845483714 | 4.7883E-84 | 5.853E-82 |
| AT5G13970 | 1.338148824 | 1.173E-18 | 1.7935E-17 |
| AT5G14130 | -1.018933375 | 0.00811841 | 0.01996026 |
| AT5G14150 | -2.940396402 | 5.0273E-09 | 3.4109E-08 |
| AT5G14200 | -2.098665239 | 2.1868E-76 | 2.2216E-74 |
| AT5G14330 | -2.19919899 | 5.9727E-18 | 8.6958E-17 |
| AT5G14390 | 1.032603193 | 2.9615E-12 | 2.7985E-11 |
| AT5G14450 | -1.092669966 | 2.349E-06 | 1.1373E-05 |
| AT5G14500 | 1.153829823 | 5.084E-13 | 5.133E-12 |
| AT5G14640 | 1.053324192 | 5.2001E-22 | 9.8397E-21 |
| AT5G14650 | -1.312361846 | 0.00150678 | 0.0044145 |
| AT5G14700 | 1.189883856 | 1.058E-13 | 1.1381E-12 |
| AT5G14750 | -1.240279173 | 0.00211196 | 0.00599169 |
| AT5G14780 | 1.963682302 | 1.6613E-09 | 1.1964E-08 |
| AT5G14895 | 2.233388132 | 0.00620777 | 0.01576285 |
| AT5G14960 | 1.108257315 | 2.5714E-05 | 0.00010663 |
| AT5G15020 | 1.232675069 | 1.2639E-22 | 2.4609E-21 |
| AT5G15130 | -2.320017812 | 2.1567E-08 | 1.3747E-07 |
| AT5G15160 | 1.137579646 | 0.00012856 | 0.00046996 |
| AT5G15180 | -1.256040239 | 0.00154291 | 0.00450648 |
| AT5G15190 | 2.894569516 | 3.5148E-36 | 1.1956E-34 |
| AT5G15210 | -1.665613626 | 9.4046E-12 | 8.4634E-11 |
| AT5G15240 | 2.246049539 | 1.5463E-27 | 3.6818E-26 |
| AT5G15250 | 7.364193715 | 4.6757E-05 | 0.00018516 |
| AT5G15260 | 1.491462044 | 7.7854E-15 | 9.1637E-14 |
| AT5G15310 | -1.200505163 | 5.8446E-08 | 3.5213E-07 |
| AT5G15350 | -1.211958343 | 1.4144E-18 | 2.1496E-17 |
| AT5G15500 | 4.747330355 | 1.003E-111 | 1.792E-109 |
| AT5G15650 | 1.026143146 | 3.6522E-28 | 9.0251E-27 |
| AT5G15660 | 5.726561349 | 1.4644E-11 | 1.2969E-10 |
| AT5G15820 | 1.122309249 | 2.063E-05 | 8.6765E-05 |
| AT5G15840 | 1.183563798 | 0.00495986 | 0.01288471 |
| AT5G15860 | 1.455103825 | 6.2083E-20 | 1.0254E-18 |
| AT5G15870 | 1.228263972 | 6.1127E-15 | 7.2433E-14 |
| AT5G15890 | -2.658398838 | 4.9232E-05 | 0.00019377 |
| AT5G15960 | 2.971411823 | 1.076E-245 | 9.111E-243 |
| AT5G15970 | 1.128483083 | 1.7361E-33 | 5.3993E-32 |
| AT5G15980 | -1.171741925 | 8.4968E-12 | 7.6736E-11 |
| AT5G16000 | -1.253585631 | 1.5432E-12 | 1.496E-11 |
| AT5G16010 | 1.056424053 | 5.7542E-16 | 7.3521E-15 |
| AT5G16030 | -1.440412082 | 6.5884E-11 | 5.4982E-10 |
| AT5G02515 | -3.031715736 | 0.00149374 | 0.00437968 |
| AT5G16110 | 1.147528794 | 8.5592E-23 | 1.6823E-21 |
| AT5G16170 | -2.631167683 | 6.6532E-05 | 0.00025598 |
| AT5G16190 | -1.895681979 | 7.5464E-08 | 4.4862E-07 |
| AT5G16220 | 1.009235675 | 1.919E-13 | 2.0149E-12 |
| AT5G16360 | 1.069275985 | 0.00015822 | 0.00056813 |
| AT5G16450 | 1.494889962 | 7.3205E-18 | 1.0585E-16 |
| AT5G16530 | -1.82859047 | 2.7144E-07 | 1.4918E-06 |
| AT5G16570 | -1.999135071 | 1.8431E-07 | 1.0369E-06 |
| AT5G16590 | -1.302364672 | 7.8865E-13 | 7.831E-12 |
| AT5G16600 | 1.806236611 | 1.6312E-13 | 1.7238E-12 |
| AT5G16720 | -1.121272844 | 1.6855E-05 | 7.1878E-05 |
| AT5G16830 | 1.115498714 | 8.1309E-14 | 8.8084E-13 |
| AT5G16960 | 4.112195171 | 8.7855E-65 | 6.8774E-63 |
| AT5G17000 | 1.161333755 | 1.5432E-18 | 2.3392E-17 |
| AT5G17010 | -1.108206323 | 5.3773E-21 | 9.4915E-20 |
| AT5G17030 | -1.875495866 | 0.00285789 | 0.00786013 |
| AT5G17040 | -2.400171485 | 5.3147E-18 | 7.7875E-17 |
| AT5G17160 | -1.201404749 | 1.2278E-11 | 1.0937E-10 |
| AT5G17170 | -1.864405465 | 3.0682E-47 | 1.5082E-45 |
| AT5G17300 | 1.805761799 | 1.0015E-07 | 5.8487E-07 |
| AT5G17370 | 1.117146621 | 2.3281E-07 | 1.2947E-06 |
| AT5G17380 | 1.565157803 | 1.6052E-43 | 6.949E-42 |
| AT5G17450 | 1.683257168 | 1.0396E-18 | 1.596E-17 |
| AT5G17460 | 3.796716773 | 0 | 0 |
| AT5G17490 | 1.531980923 | 4.6129E-11 | 3.9168E-10 |
| AT5G17630 | -1.0781346 | 5.0485E-09 | 3.4243E-08 |
| AT5G17650 | 1.01163283 | 2.4775E-07 | 1.3709E-06 |
| AT5G17700 | -1.515407172 | 6.9021E-23 | 1.3612E-21 |
| AT5G17760 | 3.00028043 | 2.641E-183 | 1.161E-180 |
| AT5G17800 | -1.100975254 | 0.00995682 | 0.02390933 |
| AT5G17820 | -1.615777358 | 8.3981E-16 | 1.0588E-14 |
| AT5G17850 | 2.118796159 | 2.081E-35 | 6.8938E-34 |
| AT5G17860 | 2.773764986 | 1.761E-54 | 1.0565E-52 |
| AT5G18030 | -2.605279357 | 0.00159703 | 0.0046489 |
| AT5G18060 | -3.32683211 | 0.00492657 | 0.01281131 |
| AT5G18080 | -2.602162531 | 0.00714479 | 0.01782727 |
| AT5G18130 | 2.192898787 | 9.7286E-89 | 1.2707E-86 |
| AT5G18400 | 1.212730376 | 3.3981E-23 | 6.8254E-22 |
| AT5G18404 | -5.527841609 | 0.00762017 | 0.01886515 |
| AT5G18430 | -1.912955978 | 5.1026E-09 | 3.4569E-08 |
| AT5G18630 | 1.023189606 | 2.8904E-08 | 1.8126E-07 |
| AT5G18633 | 3.393882634 | 0.00011388 | 0.00042082 |
| AT5G18661 | -4.204505619 | 0.00226962 | 0.00639929 |
| AT5G18690 | -1.834556048 | 2.1536E-09 | 1.5273E-08 |
| AT5G18790 | -1.007900602 | 0.00010211 | 0.00038077 |
| AT5G18840 | -1.147007938 | 0.00683258 | 0.01713053 |
| AT5G18930 | -1.194855427 | 0.0089245 | 0.02168558 |
| AT5G19040 | -2.024855598 | 1.0604E-06 | 5.3779E-06 |
| AT5G19060 | -1.536667647 | 0.00132157 | 0.00391911 |
| AT5G19120 | -1.067576876 | 4.525E-08 | 2.77E-07 |
| AT5G19190 | -2.068626834 | 9.7152E-08 | 5.6839E-07 |
| AT5G19230 | -2.137078379 | 5.7625E-21 | 1.0148E-19 |
| AT5G19240 | -2.777172068 | 2.5163E-40 | 9.7818E-39 |
| AT5G19260 | -2.981390571 | 1.6781E-12 | 1.6206E-11 |
| AT5G19410 | -1.168163652 | 0.00023033 | 0.00079954 |
| AT5G19440 | 1.516679418 | 2.1206E-36 | 7.3112E-35 |
| AT5G19470 | 2.638572014 | 0.00136132 | 0.0040249 |
| AT5G19530 | -1.530876353 | 5.4499E-19 | 8.5207E-18 |
| AT5G19600 | -2.69250486 | 1.2715E-23 | 2.6279E-22 |
| AT5G19790 | -3.3704702 | 5.2672E-06 | 2.4284E-05 |
| AT5G19800 | -6.642616958 | 0.00014987 | 0.00054086 |
| AT5G19855 | 1.335395256 | 1.7159E-23 | 3.5208E-22 |
| AT5G19875 | 1.252106115 | 4.2671E-12 | 3.9634E-11 |
| AT5G19880 | -7.516091006 | 3.3291E-06 | 1.5814E-05 |
| AT5G20000 | 1.014837491 | 2.1042E-17 | 2.9453E-16 |
| AT5G20190 | 1.287707263 | 4.0156E-34 | 1.2695E-32 |
| AT5G20230 | 2.54329946 | 1.256E-56 | 7.9311E-55 |
| AT5G20250 | -1.435802105 | 3.4525E-21 | 6.1847E-20 |
| AT5G20270 | 1.623119555 | 6.1273E-43 | 2.5793E-41 |
| AT5G20360 | 1.110611834 | 6.3655E-15 | 7.5351E-14 |
| AT5G20370 | 7.314248758 | 6.2985E-05 | 0.00024336 |
| AT5G20380 | 1.39361568 | 1.0219E-24 | 2.2016E-23 |
| AT5G20520 | 1.16842642 | 2.8097E-23 | 5.6635E-22 |
| AT5G20630 | -1.626906105 | 4.7298E-43 | 2.0058E-41 |
| AT5G20740 | -1.811880428 | 1.2786E-12 | 1.2479E-11 |
| AT5G20790 | -2.800936357 | 9.5386E-07 | 4.8679E-06 |
| AT5G20830 | 3.153075149 | 9.248E-179 | 3.645E-176 |
| AT5G20840 | 1.168748063 | 1.1486E-19 | 1.8607E-18 |
| AT5G20860 | -2.31151596 | 6.481E-05 | 0.00024986 |
| AT5G20900 | 1.589868521 | 1.9565E-48 | 9.9824E-47 |
| AT5G20910 | 1.228739063 | 8.1218E-11 | 6.719E-10 |
| AT5G20935 | -1.160551252 | 1.4075E-05 | 6.0909E-05 |
| AT5G21140 | -1.121195213 | 0.0069959 | 0.0174863 |
| AT5G21940 | 1.12037413 | 5.5719E-20 | 9.2292E-19 |
| AT5G22000 | 1.071857589 | 2.4996E-22 | 4.7974E-21 |
| AT5G22020 | -1.256216695 | 1.8728E-22 | 3.6217E-21 |
| AT5G22120 | 1.53276692 | 2.3447E-28 | 5.8575E-27 |
| AT5G22220 | 1.508776346 | 2.1906E-13 | 2.2833E-12 |
| AT5G22270 | 1.104305062 | 2.9649E-07 | 1.621E-06 |
| AT5G22290 | 1.705143201 | 8.6284E-26 | 1.945E-24 |
| AT5G22410 | -9.121188725 | 1.3083E-09 | 9.5449E-09 |
| AT5G22450 | 1.229652067 | 1.7585E-21 | 3.208E-20 |
| AT5G22460 | 3.862457934 | 4.2273E-53 | 2.4278E-51 |
| AT5G22500 | 2.798218878 | 1.292E-79 | 1.4336E-77 |
| AT5G22545 | 3.619111598 | 0.00270304 | 0.00747762 |
| AT5G22550 | -2.38405299 | 0.0006212 | 0.00198093 |
| AT5G22555 | -7.065746685 | 1.6859E-15 | 2.0858E-14 |
| AT5G22560 | -5.522862132 | 0.00720924 | 0.01796847 |
| AT5G22580 | -1.58161007 | 6.4452E-13 | 6.4418E-12 |
| AT5G22860 | 1.520565714 | 5.3627E-16 | 6.8672E-15 |
| AT5G22890 | -2.730550923 | 1.7083E-15 | 2.1118E-14 |
| AT5G22930 | -3.38019992 | 5.6964E-13 | 5.7234E-12 |
| AT5G22940 | -2.500518017 | 7.2681E-21 | 1.2711E-19 |
| AT5G23000 | -1.78340302 | 0.00956975 | 0.02309878 |
| AT5G23010 | -1.215093294 | 1.0062E-18 | 1.5468E-17 |
| AT5G23030 | -6.858606367 | 5.989E-05 | 0.00023222 |
| AT5G23050 | 1.082410575 | 8.0049E-24 | 1.671E-22 |
| AT5G23060 | -1.732765546 | 8.573E-52 | 4.7913E-50 |
| AT5G23080 | 1.000677326 | 2.2906E-13 | 2.3843E-12 |
| AT5G23100 | -1.483051229 | 5.3306E-08 | 3.2252E-07 |
| AT5G23110 | 1.222518352 | 8.4576E-17 | 1.1439E-15 |
| AT5G23150 | 1.09013394 | 3.3424E-12 | 3.1415E-11 |
| AT5G23210 | -1.516152479 | 9.1649E-16 | 1.1523E-14 |
| AT5G23220 | 1.808197864 | 0.00446776 | 0.01173975 |
| AT5G23300 | -1.125208522 | 2.1791E-10 | 1.72E-09 |
| AT5G23380 | 1.035367272 | 2.6905E-05 | 0.00011111 |
| AT5G23420 | -1.648618011 | 2.7074E-21 | 4.8805E-20 |
| AT5G23690 | -1.069490587 | 1.7511E-06 | 8.6359E-06 |
| AT5G23730 | -1.106877322 | 1.7197E-06 | 8.49E-06 |
| AT5G23820 | -1.129949963 | 1.9002E-17 | 2.6697E-16 |
| AT5G23830 | -2.229929031 | 4.4221E-33 | 1.355E-31 |
| AT5G23840 | -1.912211865 | 7.6825E-05 | 0.00029277 |
| AT5G23850 | 1.748538058 | 9.4568E-22 | 1.7632E-20 |
| AT5G24030 | 1.670847132 | 5.7062E-31 | 1.6123E-29 |
| AT5G24070 | -4.04086261 | 0.00470072 | 0.01228691 |
| AT5G24080 | 4.990875482 | 2.609E-145 | 7.016E-143 |
| AT5G24100 | -3.481591669 | 2.0678E-07 | 1.1579E-06 |
| AT5G24105 | -1.317587326 | 0.00115126 | 0.00345939 |
| AT5G24110 | 1.770860321 | 0.00129632 | 0.00385273 |
| AT5G24120 | 1.858496886 | 1.034E-37 | 3.7164E-36 |
| AT5G24140 | -1.470882184 | 0.00292881 | 0.00803972 |
| AT5G24230 | -3.025982563 | 7.0132E-08 | 4.1812E-07 |
| AT5G24290 | -1.275265738 | 4.0754E-08 | 2.5116E-07 |
| AT5G24313 | -6.355412781 | 0.00047493 | 0.00155108 |
| AT5G24330 | -2.889930339 | 4.1107E-07 | 2.2031E-06 |
| AT5G24570 | 1.672527414 | 1.6136E-21 | 2.9507E-20 |
| AT5G24580 | -1.434048562 | 3.1999E-07 | 1.7415E-06 |
| AT5G24600 | 1.611232311 | 0.00093458 | 0.00287016 |
| AT5G24770 | 1.808413265 | 0.00013289 | 0.00048423 |
| AT5G24800 | 1.911504426 | 3.5022E-58 | 2.307E-56 |
| AT5G24860 | 3.404901548 | 1.6743E-06 | 8.2715E-06 |
| AT5G24880 | -1.997362888 | 0.00333398 | 0.0090155 |
| AT5G24930 | 1.23220895 | 1.9627E-37 | 7.0208E-36 |
| AT5G24940 | 1.343326732 | 1.2714E-05 | 5.5332E-05 |
| AT5G24990 | -1.147163213 | 1.185E-05 | 5.1969E-05 |
| AT5G25090 | -1.54117818 | 3.104E-12 | 2.927E-11 |
| AT5G25110 | 2.67573919 | 1.5471E-30 | 4.2866E-29 |
| AT5G25160 | -1.367627747 | 0.00991815 | 0.02383151 |
| AT5G25220 | 1.277521433 | 4.6981E-30 | 1.28E-28 |
| AT5G25230 | 1.76127511 | 0.006868 | 0.01720991 |
| AT5G25240 | 2.064928633 | 1.0202E-12 | 1.0043E-11 |
| AT5G25250 | -2.820455694 | 1.1626E-30 | 3.2568E-29 |
| AT5G25260 | -5.007575365 | 7.6405E-08 | 4.5375E-07 |
| AT5G25280 | 1.687071241 | 4.962E-60 | 3.4266E-58 |
| AT5G25350 | 1.194152644 | 2.0736E-23 | 4.2132E-22 |
| AT5G25460 | -2.632986478 | 3.635E-100 | 5.5766E-98 |
| AT5G25490 | -1.06354873 | 0.00021822 | 0.00076071 |
| AT5G25610 | 2.333016289 | 3.2719E-12 | 3.0765E-11 |
| AT5G25810 | -1.658343083 | 1.0222E-06 | 5.1922E-06 |
| AT5G25840 | -1.029724079 | 9.0135E-07 | 4.6143E-06 |
| AT5G25970 | -2.745751614 | 4.5558E-07 | 2.4286E-06 |
| AT5G26270 | -1.067936436 | 2.9309E-05 | 0.00012008 |
| AT5G26280 | -1.157724923 | 2.3978E-25 | 5.3316E-24 |
| AT5G26290 | -2.411712942 | 8.0717E-35 | 2.6155E-33 |
| AT5G26310 | -1.740608796 | 0.00015931 | 0.00057149 |
| AT5G26330 | -1.661502024 | 1.5548E-07 | 8.8339E-07 |
| AT5G26340 | 2.686800842 | 1.282E-162 | 4.248E-160 |
| AT5G26670 | -1.406581576 | 7.6665E-08 | 4.5517E-07 |
| AT5G26760 | 1.144224876 | 6.8817E-19 | 1.0686E-17 |
| AT5G26770 | 2.083444448 | 2.2063E-37 | 7.8675E-36 |
| AT5G26865 | 2.711381115 | 0.00806791 | 0.01984891 |
| AT5G26920 | -2.008409579 | 8.6708E-18 | 1.2465E-16 |
| AT5G27150 | 1.48773565 | 1.7024E-41 | 6.8268E-40 |
| AT5G27280 | 1.387314862 | 1.2837E-14 | 1.4738E-13 |
| AT5G27330 | -1.259575468 | 1.2095E-20 | 2.0834E-19 |
| AT5G27360 | -1.599505017 | 1.8649E-05 | 7.894E-05 |
| AT5G27520 | 1.918793155 | 2.0484E-34 | 6.5213E-33 |
| AT5G27610 | 2.561540541 | 6.0608E-48 | 3.0515E-46 |
| AT5G27760 | 1.606787732 | 1.1557E-53 | 6.7736E-52 |
| AT5G27930 | 1.18424014 | 9.943E-17 | 1.3393E-15 |
| AT5G28030 | -1.222510513 | 7.3952E-05 | 0.00028244 |
| AT5G28237 | 5.258472385 | 7.4179E-11 | 6.1635E-10 |
| AT5G28400 | 1.189003976 | 0.00606019 | 0.01541894 |
| AT5G28510 | 1.763226569 | 1.4979E-14 | 1.7111E-13 |
| AT5G28520 | 3.001463199 | 1.2391E-05 | 5.4084E-05 |
| AT5G28630 | -1.675161133 | 0.00283324 | 0.00780081 |
| AT5G28650 | -3.210440663 | 0.0052063 | 0.01346216 |
| AT5G28830 | 1.47954819 | 1.9248E-11 | 1.6877E-10 |
| AT5G29000 | 1.358096298 | 2.4741E-20 | 4.1799E-19 |
| AT5G33370 | -1.830258114 | 4.3211E-06 | 2.0153E-05 |
| AT5G35110 | 1.648839508 | 0.00019637 | 0.00069185 |
| AT5G35190 | -5.643044784 | 1.2193E-20 | 2.0987E-19 |
| AT5G35320 | 1.243329623 | 4.9083E-15 | 5.8587E-14 |
| AT5G35460 | 1.141582424 | 2.2799E-19 | 3.6392E-18 |
| AT5G35740 | -1.197641259 | 3.7447E-07 | 2.0178E-06 |
| AT5G35777 | -2.571046678 | 0.00036512 | 0.00121904 |
| AT5G35917 | 6.82984123 | 0.00044353 | 0.00145433 |
| AT5G35970 | 1.261576718 | 2.078E-27 | 4.9323E-26 |
| AT5G36120 | -1.110502386 | 3.5873E-09 | 2.4818E-08 |
| AT5G36140 | -3.776958485 | 1.6863E-15 | 2.0858E-14 |
| AT5G36150 | -3.728478882 | 1.8787E-09 | 1.3424E-08 |
| AT5G36180 | -2.121493628 | 1.9594E-12 | 1.8811E-11 |
| AT5G36907 | 1.381213394 | 0.00583082 | 0.01489339 |
| AT5G36925 | 2.27269421 | 3.2958E-09 | 2.2881E-08 |
| AT5G36970 | -4.120917357 | 4.7693E-09 | 3.2474E-08 |
| AT5G37300 | 3.941988778 | 3.7751E-62 | 2.7747E-60 |
| AT5G37540 | 2.562441475 | 1.4005E-38 | 5.1466E-37 |
| AT5G37740 | -1.254195398 | 1.9068E-14 | 2.1545E-13 |
| AT5G37750 | 1.760702871 | 0.00149988 | 0.00439655 |
| AT5G37840 | -4.842082016 | 0.00016409 | 0.00058743 |
| AT5G38000 | -2.197615564 | 1.873E-10 | 1.4881E-09 |
| AT5G38010 | -2.504751331 | 3.7558E-09 | 2.5921E-08 |
| AT5G38020 | -2.229418524 | 3.6826E-46 | 1.7285E-44 |
| AT5G38030 | -2.054604221 | 9.709E-22 | 1.8072E-20 |
| AT5G38100 | -3.410738859 | 0.0021871 | 0.00618723 |
| AT5G38200 | 1.937385949 | 1.4437E-35 | 4.8037E-34 |
| AT5G38240 | 2.927064871 | 2.3102E-06 | 1.1197E-05 |
| AT5G38320 | -2.868364365 | 0.00097278 | 0.00297987 |
| AT5G38420 | -1.974230022 | 1.4713E-90 | 2.0139E-88 |
| AT5G38430 | -2.88379917 | 2.212E-146 | 6.165E-144 |
| AT5G38540 | -3.494854447 | 6.1001E-07 | 3.1988E-06 |
| AT5G38550 | -1.757552367 | 0.00038541 | 0.00128065 |
| AT5G38610 | -1.006474534 | 0.00639123 | 0.01615869 |
| AT5G38900 | -3.737528137 | 9.4601E-43 | 3.9532E-41 |
| AT5G38910 | -5.24577811 | 6.6245E-39 | 2.4463E-37 |
| AT5G38930 | -1.610750813 | 0.00013178 | 0.00048071 |
| AT5G38940 | -1.423834165 | 7.6252E-14 | 8.2762E-13 |
| AT5G38970 | -6.277173342 | 0.00069825 | 0.00219935 |
| AT5G38980 | -1.427741186 | 1.0527E-26 | 2.4578E-25 |
| AT5G38990 | -1.043108233 | 1.7168E-09 | 1.2333E-08 |
| AT5G39000 | -5.768241167 | 0.00042598 | 0.00140223 |
| AT5G00560 | 1.778290831 | 3.9355E-11 | 3.3566E-10 |
| AT5G39050 | 2.560861107 | 2.712E-104 | 4.397E-102 |
| AT5G39090 | 1.383479523 | 0.00019825 | 0.0006978 |
| AT5G39210 | -1.844173666 | 2.4555E-27 | 5.8103E-26 |
| AT5G39520 | 3.826388057 | 4.2246E-17 | 5.8033E-16 |
| AT5G39580 | -3.846249427 | 1.4484E-10 | 1.1641E-09 |
| AT5G39590 | 1.267501404 | 1.244E-30 | 3.4719E-29 |
| AT5G39610 | 2.759009202 | 7.6195E-68 | 6.5231E-66 |
| AT5G39660 | 1.796126523 | 3.5825E-17 | 4.9361E-16 |
| AT5G39670 | -2.637338337 | 2.5455E-07 | 1.4047E-06 |
| AT5G39720 | 2.82148726 | 0.00070541 | 0.00221944 |
| AT5G39770 | -5.522862132 | 0.00720924 | 0.01796847 |
| AT5G39785 | 1.336107254 | 3.1944E-09 | 2.2208E-08 |
| AT5G39860 | -4.583808242 | 0.00069738 | 0.00219721 |
| AT5G40010 | -1.293011979 | 0.00268152 | 0.00742691 |
| AT5G40080 | -1.221580394 | 2.3348E-05 | 9.7372E-05 |
| AT5G40150 | -1.755513609 | 1.7013E-10 | 1.3574E-09 |
| AT5G40240 | -1.017320906 | 1.6951E-06 | 8.3722E-06 |
| AT5G40390 | 1.362493215 | 6.7253E-37 | 2.3723E-35 |
| AT5G40450 | 1.408147189 | 6.7336E-28 | 1.6374E-26 |
| AT5G40510 | -1.58803747 | 5.3847E-11 | 4.5486E-10 |
| AT5G40540 | 1.288357585 | 1.0367E-11 | 9.2855E-11 |
| AT5G40590 | -4.161020345 | 2.6697E-12 | 2.5342E-11 |
| AT5G40610 | -1.017838857 | 1.0487E-08 | 6.9164E-08 |
| AT5G40630 | -1.368511278 | 0.00891826 | 0.02167502 |
| AT5G40790 | 3.750659783 | 3.1164E-11 | 2.679E-10 |
| AT5G40800 | 3.433885456 | 1.3671E-27 | 3.2688E-26 |
| AT5G40830 | -1.278945742 | 1.4376E-13 | 1.5248E-12 |
| AT5G40860 | -2.297630495 | 0.00048778 | 0.00158873 |
| AT5G40880 | 1.859437673 | 1.1511E-23 | 2.3812E-22 |
| AT5G40990 | -9.004201127 | 2.4036E-09 | 1.6915E-08 |
| AT5G41050 | -1.345230515 | 5.9664E-08 | 3.5899E-07 |
| AT5G41350 | 1.01158139 | 2.4642E-09 | 1.732E-08 |
| AT5G41360 | 1.110542133 | 8.052E-14 | 8.727E-13 |
| AT5G41750 | -2.385420003 | 0.00058933 | 0.00188748 |
| AT5G41880 | -1.144428237 | 1.0496E-06 | 5.3257E-06 |
| AT5G41900 | 1.832609861 | 8.4527E-25 | 1.8314E-23 |
| AT5G42050 | 1.424800371 | 4.7585E-51 | 2.621E-49 |
| AT5G42100 | -1.133545505 | 2.7347E-17 | 3.8E-16 |
| AT5G42180 | -3.821705126 | 5.4039E-30 | 1.467E-28 |
| AT5G42380 | 1.140234044 | 0.00011069 | 0.00040989 |
| AT5G42500 | -2.368226198 | 6.1762E-09 | 4.1523E-08 |
| AT5G42570 | 1.961697813 | 3.0288E-66 | 2.4464E-64 |
| AT5G42580 | -2.302849368 | 1.38E-08 | 8.9971E-08 |
| AT5G42590 | -2.933413026 | 8.106E-19 | 1.2503E-17 |
| AT5G00580 | -7.368532898 | 1.3187E-05 | 5.7306E-05 |
| AT5G42655 | -4.591184019 | 0.00065874 | 0.00208697 |
| AT5G42680 | -1.267042885 | 0.00121089 | 0.00362049 |
| AT5G42700 | -1.164018797 | 0.00551945 | 0.01418684 |
| AT5G42780 | -1.578985983 | 4.5507E-06 | 2.1159E-05 |
| AT5G42830 | -3.157712742 | 1.357E-35 | 4.5216E-34 |
| AT5G42860 | -1.176651034 | 1.655E-07 | 9.3687E-07 |
| AT5G42900 | 1.657279655 | 3.378E-45 | 1.5536E-43 |
| AT5G43020 | -1.076918955 | 6.5871E-11 | 5.4982E-10 |
| AT5G43040 | -1.733860652 | 0.00217795 | 0.00616439 |
| AT5G43070 | -1.224032562 | 4.48E-07 | 2.3915E-06 |
| AT5G43150 | 1.94472764 | 3.1485E-43 | 1.3452E-41 |
| AT5G43180 | 2.363795595 | 9.2841E-44 | 4.0654E-42 |
| AT5G43230 | -4.378491629 | 0.00137011 | 0.00404886 |
| AT5G43250 | -2.431424616 | 1.0928E-07 | 6.3464E-07 |
| AT5G43260 | 1.680360142 | 2.824E-39 | 1.0635E-37 |
| AT5G43350 | -2.556969411 | 2.0065E-47 | 9.9273E-46 |
| AT5G43380 | 2.22824502 | 2.6804E-40 | 1.0402E-38 |
| AT5G43430 | 1.332114593 | 3.646E-18 | 5.4082E-17 |
| AT5G43450 | 2.513547104 | 3.2488E-59 | 2.1971E-57 |
| AT5G43570 | -2.713312041 | 1.1677E-05 | 5.1251E-05 |
| AT5G43580 | -3.515905797 | 4.115E-103 | 6.578E-101 |
| AT5G43620 | 2.453002826 | 7.1994E-16 | 9.1272E-15 |
| AT5G43700 | -1.456397813 | 1.1435E-19 | 1.8537E-18 |
| AT5G43840 | 4.287885685 | 1.011E-19 | 1.6459E-18 |
| AT5G43850 | 1.546438927 | 1.198E-34 | 3.8624E-33 |
| AT5G43890 | -4.139466755 | 0.00338539 | 0.00913723 |
| AT5G44005 | 1.593667698 | 2.0382E-13 | 2.1352E-12 |
| AT5G44020 | -2.771806616 | 1.833E-100 | 2.8304E-98 |
| AT5G44090 | 1.181004307 | 1.3491E-22 | 2.6222E-21 |
| AT5G44120 | -7.287440576 | 0.00121132 | 0.00362129 |
| AT5G44130 | -1.815396034 | 4.8019E-35 | 1.5725E-33 |
| AT5G44210 | 1.252543526 | 1.7667E-05 | 7.5077E-05 |
| AT5G44260 | 2.671280683 | 3.1168E-15 | 3.7735E-14 |
| AT5G44310 | 1.658981857 | 0.00523975 | 0.01353488 |
| AT5G44350 | -2.000155766 | 3.5881E-06 | 1.6949E-05 |
| AT5G44380 | -2.058584289 | 2.479E-32 | 7.3974E-31 |
| AT5G44390 | -2.926289285 | 1.5032E-09 | 1.0908E-08 |
| AT5G44400 | -1.887075893 | 8.0173E-37 | 2.8021E-35 |
| AT5G44460 | -2.419852779 | 0.00010159 | 0.00037913 |
| AT5G44480 | -2.126815451 | 2.128E-21 | 3.8697E-20 |
| AT5G44550 | -1.600706301 | 1.1376E-16 | 1.5243E-15 |
| AT5G00585 | -2.575050307 | 0.00683704 | 0.01713985 |
| AT5G44570 | 4.041676818 | 1.9069E-09 | 1.3606E-08 |
| AT5G44572 | -1.011343358 | 0.00526312 | 0.01358911 |
| AT5G44600 | -1.122370048 | 4.2555E-07 | 2.277E-06 |
| AT5G44610 | -1.617846596 | 4.1484E-15 | 4.9855E-14 |
| AT5G44635 | -1.492615158 | 5.8588E-12 | 5.3718E-11 |
| AT5G44670 | 1.682323283 | 6.4996E-23 | 1.2841E-21 |
| AT5G44680 | -1.71994401 | 1.174E-20 | 2.0253E-19 |
| AT5G44820 | -2.316145329 | 2.6393E-05 | 0.00010917 |
| AT5G44900 | -6.982295128 | 3.6316E-05 | 0.00014645 |
| AT5G44910 | -1.371178519 | 1.4916E-05 | 6.426E-05 |
| AT5G44990 | -5.694561543 | 1.3459E-06 | 6.7462E-06 |
| AT5G45000 | -4.048053759 | 8.9016E-07 | 4.5673E-06 |
| AT5G45070 | -1.052553924 | 9.7739E-05 | 0.00036559 |
| AT5G45090 | -2.955171868 | 0.00174424 | 0.00503151 |
| AT5G45115 | -2.096592134 | 0.0022815 | 0.00642644 |
| AT5G45130 | 1.025932998 | 1.8103E-20 | 3.0858E-19 |
| AT5G45200 | -2.053326728 | 0.00169246 | 0.00489577 |
| AT5G45210 | -2.798454 | 5.4604E-09 | 3.6895E-08 |
| AT5G45330 | 1.26816982 | 2.5178E-11 | 2.1858E-10 |
| AT5G45340 | 3.487487288 | 9.9018E-80 | 1.1041E-77 |
| AT5G45370 | 1.029412265 | 0.00021586 | 0.00075364 |
| AT5G45380 | -1.801744637 | 1.2512E-10 | 1.0128E-09 |
| AT5G45630 | 7.15717668 | 1.6777E-41 | 6.7396E-40 |
| AT5G45650 | -1.139666676 | 1.2637E-09 | 9.2316E-09 |
| AT5G45670 | -1.041544698 | 2.692E-11 | 2.3273E-10 |
| AT5G45700 | -1.724147467 | 1.0869E-11 | 9.7198E-11 |
| AT5G45850 | -2.372920389 | 3.2409E-05 | 0.00013196 |
| AT5G45900 | 1.137335174 | 8.1991E-11 | 6.7781E-10 |
| AT5G45930 | -1.08424532 | 2.8521E-12 | 2.7006E-11 |
| AT5G45950 | 2.385043675 | 2.4178E-67 | 2.0319E-65 |
| AT5G46050 | 1.681635031 | 1.0099E-07 | 5.8962E-07 |
| AT5G46250 | 1.158367506 | 2.145E-25 | 4.7882E-24 |
| AT5G46260 | -2.994107859 | 0.0001589 | 0.00057045 |
| AT5G46280 | -1.443082503 | 1.085E-16 | 1.4577E-15 |
| AT5G46410 | 1.655775787 | 4.321E-23 | 8.6186E-22 |
| AT5G46570 | -1.004966603 | 3.0507E-09 | 2.1253E-08 |
| AT5G46580 | -1.091234591 | 3.895E-20 | 6.5272E-19 |
| AT5G46690 | -1.607577371 | 2.7047E-17 | 3.7606E-16 |
| AT5G46790 | -1.357794022 | 1.3521E-14 | 1.55E-13 |
| AT5G46960 | -6.511074158 | 0.00035044 | 0.00117557 |
| AT5G47020 | 1.842156673 | 2.074E-58 | 1.3781E-56 |
| AT5G47040 | 1.51230855 | 4.3799E-55 | 2.6556E-53 |
| AT5G47070 | 1.086687555 | 5.2825E-08 | 3.1995E-07 |
| AT5G47180 | 1.021287689 | 1.3093E-11 | 1.1622E-10 |
| AT5G47200 | 1.044204826 | 5.1769E-24 | 1.0926E-22 |
| AT5G47240 | 1.292021379 | 1.3734E-18 | 2.0887E-17 |
| AT5G47330 | 2.8384925 | 4.0813E-12 | 3.7984E-11 |
| AT5G47380 | -1.070619117 | 5.3909E-05 | 0.00021082 |
| AT5G47430 | 1.169150652 | 9.5453E-23 | 1.8696E-21 |
| AT5G47500 | -1.467716856 | 2.8169E-26 | 6.5105E-25 |
| AT5G47550 | 2.265476872 | 2.3362E-62 | 1.7282E-60 |
| AT5G47560 | 2.8907855 | 1.26E-132 | 2.853E-130 |
| AT5G47590 | 1.374578241 | 0.00189 | 0.0054063 |
| AT5G47640 | 2.094445577 | 3.5454E-53 | 2.0465E-51 |
| AT5G47740 | -2.113157629 | 1.3596E-05 | 5.8961E-05 |
| AT5G47860 | 1.140609443 | 5.3565E-13 | 5.3986E-12 |
| AT5G47880 | 1.951891488 | 1.1552E-82 | 1.3611E-80 |
| AT5G47910 | -1.510706316 | 3.976E-25 | 8.722E-24 |
| AT5G47920 | 1.118826619 | 0.00224907 | 0.00634213 |
| AT5G47950 | -1.802537153 | 0.00175038 | 0.00504733 |
| AT5G47960 | -1.551234538 | 0.0033202 | 0.00898676 |
| AT5G47980 | -4.667961528 | 2.7784E-31 | 7.9486E-30 |
| AT5G47990 | -2.214008964 | 3.489E-58 | 2.305E-56 |
| AT5G48000 | -2.418635563 | 2.9405E-70 | 2.6462E-68 |
| AT5G48010 | -2.235596619 | 5.6256E-67 | 4.6256E-65 |
| AT5G48110 | -2.039414876 | 9.2252E-11 | 7.5744E-10 |
| AT5G48170 | -2.086060611 | 0.00143791 | 0.00422955 |
| AT5G48180 | 2.959500149 | 3.843E-196 | 2.043E-193 |
| AT5G48190 | -5.572628424 | 0.00626169 | 0.0158786 |
| AT5G48290 | -1.084249167 | 0.00063545 | 0.0020216 |
| AT5G48360 | -1.039804065 | 4.7832E-12 | 4.4283E-11 |
| AT5G48390 | 2.00826853 | 2.2447E-05 | 9.3866E-05 |
| AT5G48400 | -1.017854413 | 3.0377E-05 | 0.00012424 |
| AT5G48410 | -2.304890066 | 9.9091E-11 | 8.1068E-10 |
| AT5G48412 | 1.387742581 | 8.7633E-25 | 1.8951E-23 |
| AT5G48430 | -3.738142312 | 2.1505E-46 | 1.0177E-44 |
| AT5G48490 | -2.932596741 | 2.9885E-11 | 2.573E-10 |
| AT5G48530 | 1.597961033 | 2.9767E-11 | 2.5638E-10 |
| AT5G48545 | -1.16498371 | 1.2976E-08 | 8.4814E-08 |
| AT5G48570 | 1.373421245 | 2.1581E-13 | 2.2525E-12 |
| AT5G48610 | 1.22021244 | 1.2908E-13 | 1.3774E-12 |
| AT5G48650 | 1.127574749 | 0.00017348 | 0.00061795 |
| AT5G48655 | 1.206743536 | 9.9962E-18 | 1.4272E-16 |
| AT5G48657 | -1.851040588 | 3.0456E-10 | 2.3687E-09 |
| AT5G48800 | -1.462871476 | 6.4801E-06 | 2.9436E-05 |
| AT5G48830 | -1.154873702 | 1.0955E-11 | 9.7934E-11 |
| AT5G48850 | 2.556538943 | 4.2025E-34 | 1.3268E-32 |
| AT5G48880 | -2.24655328 | 8.657E-77 | 8.9136E-75 |
| AT5G49120 | 2.500490921 | 2.2183E-10 | 1.7479E-09 |
| AT5G49270 | -4.171219542 | 1.2777E-16 | 1.705E-15 |
| AT5G49300 | -1.564620573 | 6.4139E-07 | 3.3488E-06 |
| AT5G49360 | -2.081752817 | 1.7461E-15 | 2.1563E-14 |
| AT5G49450 | 1.315354165 | 1.2682E-09 | 9.2612E-09 |
| AT5G49480 | 1.097131479 | 1.0854E-17 | 1.5477E-16 |
| AT5G49520 | 1.564259966 | 2.4017E-13 | 2.4898E-12 |
| AT5G49630 | -3.883169173 | 3.9815E-35 | 1.3076E-33 |
| AT5G49730 | -1.472454166 | 6.3805E-05 | 0.00024624 |
| AT5G49740 | -2.050841411 | 1.8551E-09 | 1.3268E-08 |
| AT5G49760 | -1.286992656 | 7.0866E-22 | 1.3299E-20 |
| AT5G49770 | -2.684886483 | 1.7178E-07 | 9.7045E-07 |
| AT5G49780 | -2.729614487 | 2.082E-08 | 1.3293E-07 |
| AT5G49800 | -1.349296332 | 0.00171842 | 0.00496142 |
| AT5G49910 | -1.137917692 | 5.8438E-19 | 9.0993E-18 |
| AT5G49920 | 2.954459479 | 0.00910949 | 0.02208343 |
| AT5G49930 | 1.322123646 | 8.9304E-28 | 2.1624E-26 |
| AT5G50010 | -2.524716319 | 1.2658E-12 | 1.236E-11 |
| AT5G50100 | 1.761645763 | 1.9814E-29 | 5.2603E-28 |
| AT5G50130 | -1.605938228 | 2.1297E-09 | 1.5114E-08 |
| AT5G50170 | 1.319102851 | 5.2205E-15 | 6.2119E-14 |
| AT5G50210 | 1.501441996 | 3.2633E-31 | 9.3008E-30 |
| AT5G50240 | 1.33676795 | 3.0987E-10 | 2.4092E-09 |
| AT5G50335 | 1.874819549 | 4.4499E-06 | 2.0707E-05 |
| AT5G50350 | 1.19476017 | 8.8449E-16 | 1.1139E-14 |
| AT5G50360 | 6.38292103 | 1.1147E-29 | 2.9837E-28 |
| AT5G50361 | 4.860627222 | 0.00132451 | 0.00392629 |
| AT5G50420 | -1.10376038 | 2.2841E-08 | 1.4503E-07 |
| AT5G50720 | 1.784878996 | 1.2391E-16 | 1.6563E-15 |
| AT5G50740 | -1.339455793 | 1.1194E-17 | 1.5933E-16 |
| AT5G50780 | 1.363963309 | 4.5346E-18 | 6.67E-17 |
| AT5G50950 | 1.195351349 | 5.6333E-24 | 1.1868E-22 |
| AT5G50960 | 1.019180137 | 4.4787E-18 | 6.592E-17 |
| AT5G51070 | 2.586657411 | 1.301E-100 | 2.023E-98 |
| AT5G51170 | 1.146391563 | 3.4172E-05 | 0.00013847 |
| AT5G51260 | 1.327260918 | 7.0892E-10 | 5.3199E-09 |
| AT5G51310 | -2.115765051 | 0.00592882 | 0.015115 |
| AT5G51440 | 1.22026061 | 6.9654E-10 | 5.2322E-09 |
| AT5G51460 | -1.023931941 | 7.9639E-07 | 4.1139E-06 |
| AT5G51465 | -4.800633973 | 0.00018557 | 0.00065722 |
| AT5G51490 | -8.359763195 | 6.221E-08 | 3.7338E-07 |
| AT5G51500 | -1.305408562 | 0.0025424 | 0.00708162 |
| AT5G51520 | -5.731798239 | 0.00408045 | 0.01081779 |
| AT5G51540 | -1.20317543 | 1.5402E-07 | 8.7644E-07 |
| AT5G51550 | -1.722465888 | 9.765E-34 | 3.0535E-32 |
| AT5G51670 | -1.133473656 | 0.00062731 | 0.00199821 |
| AT5G51710 | 1.00044298 | 4.9275E-09 | 3.3482E-08 |
| AT5G51720 | -1.89619452 | 5.5651E-29 | 1.4406E-27 |
| AT5G51740 | 1.089083036 | 4.2503E-10 | 3.2514E-09 |
| AT5G51760 | 2.602815341 | 0.00035863 | 0.00119883 |
| AT5G51780 | -2.278491148 | 1.7017E-09 | 1.2232E-08 |
| AT5G51790 | -2.523531338 | 0.00017374 | 0.0006188 |
| AT5G51830 | 2.109560887 | 1.1256E-81 | 1.2994E-79 |
| AT5G51980 | 1.006311254 | 2.0958E-14 | 2.3611E-13 |
| AT5G51990 | 7.623738299 | 7.7744E-07 | 4.0205E-06 |
| AT5G52010 | -1.203817506 | 1.0749E-13 | 1.1546E-12 |
| AT5G52050 | 3.794552928 | 5.6983E-24 | 1.1994E-22 |
| AT5G52300 | 5.36643325 | 3.5597E-06 | 1.6829E-05 |
| AT5G52310 | 3.372392728 | 7.7605E-27 | 1.8194E-25 |
| AT5G52510 | 1.103990924 | 2.6145E-23 | 5.284E-22 |
| AT5G52550 | 1.090323805 | 2.2551E-16 | 2.9625E-15 |
| AT5G52570 | 3.615235127 | 1.2606E-92 | 1.7569E-90 |
| AT5G52580 | 1.369291472 | 8.2926E-27 | 1.9401E-25 |
| AT5G52660 | 1.83081542 | 2.2713E-31 | 6.5386E-30 |
| AT5G52670 | -8.187347221 | 1.4496E-07 | 8.2752E-07 |
| AT5G52780 | -2.612289741 | 3.8029E-49 | 2.0122E-47 |
| AT5G52810 | 1.433752956 | 5.0386E-21 | 8.9419E-20 |
| AT5G52930 | -2.14545208 | 0.00958244 | 0.02312695 |
| AT5G53050 | 1.004260959 | 5.1288E-10 | 3.9013E-09 |
| AT5G00655 | 9.172929017 | 3.9917E-09 | 2.7474E-08 |
| AT5G53120 | 2.535762446 | 6.085E-121 | 1.231E-118 |
| AT5G53130 | 1.161322703 | 8.7644E-20 | 1.4341E-18 |
| AT5G53250 | -2.468314906 | 4.1373E-08 | 2.547E-07 |
| AT5G53320 | -2.754916194 | 1.5059E-10 | 1.2078E-09 |
| AT5G53390 | 1.824426422 | 0.0031851 | 0.0086549 |
| AT5G53450 | 2.191643431 | 4.605E-105 | 7.628E-103 |
| AT5G53486 | -2.204603618 | 2.3077E-09 | 1.6281E-08 |
| AT5G53590 | 1.0583936 | 6.3323E-12 | 5.7921E-11 |
| AT5G53710 | 4.814211476 | 1.4106E-12 | 1.3709E-11 |
| AT5G53830 | 1.122006387 | 5.4317E-07 | 2.8601E-06 |
| AT5G53870 | 2.147510241 | 3.4036E-09 | 2.3597E-08 |
| AT5G53880 | -1.152851249 | 6.6229E-13 | 6.6079E-12 |
| AT5G53970 | 2.664181112 | 3.601E-104 | 5.797E-102 |
| AT5G53980 | -4.984871046 | 0.00026242 | 0.00090175 |
| AT5G53990 | -1.178967201 | 7.7683E-06 | 3.4995E-05 |
| AT5G54040 | -2.037600546 | 0.00042138 | 0.0013877 |
| AT5G54050 | -5.72330109 | 0.00398289 | 0.01058124 |
| AT5G54080 | 2.28919 | 2.0357E-56 | 1.2784E-54 |
| AT5G54145 | -1.03670445 | 6.5341E-05 | 0.00025169 |
| AT5G54165 | 5.722329337 | 1.302E-158 | 4.077E-156 |
| AT5G54170 | 1.283371461 | 6.3344E-16 | 8.0619E-15 |
| AT5G54230 | 2.554156517 | 2.5399E-19 | 4.0429E-18 |
| AT5G54240 | 2.90423771 | 1.1562E-32 | 3.4912E-31 |
| AT5G54300 | 3.218226825 | 6.3633E-66 | 5.0857E-64 |
| AT5G54370 | -1.731712075 | 6.5555E-10 | 4.9437E-09 |
| AT5G54380 | -1.78298452 | 1.0507E-21 | 1.9511E-20 |
| AT5G54585 | 2.27066069 | 3.6237E-20 | 6.0771E-19 |
| AT5G54630 | -1.579940248 | 1.4877E-13 | 1.5773E-12 |
| AT5G54690 | -1.077620563 | 0.00578957 | 0.01480035 |
| AT5G54730 | 1.98219926 | 4.1899E-43 | 1.7868E-41 |
| AT5G54840 | 1.298923131 | 6.908E-16 | 8.7724E-15 |
| AT5G54860 | 1.295665422 | 5.4908E-20 | 9.1081E-19 |
| AT5G54870 | 1.059118705 | 2.7922E-16 | 3.645E-15 |
| AT5G54940 | 1.349436053 | 7.8551E-36 | 2.6444E-34 |
| AT5G54970 | -1.44509852 | 5.8543E-11 | 4.9108E-10 |
| AT5G55110 | -2.259949217 | 6.3429E-05 | 0.00024483 |
| AT5G55180 | 1.765971904 | 1.4668E-60 | 1.0285E-58 |
| AT5G55400 | 1.924906298 | 1.0292E-43 | 4.4983E-42 |
| AT5G55460 | 1.520906857 | 0.0098354 | 0.02364509 |
| AT5G55470 | 1.315391459 | 2.0589E-05 | 8.6606E-05 |
| AT5G55530 | 1.072506113 | 3.2006E-19 | 5.077E-18 |
| AT5G55580 | -1.238752703 | 1.7787E-12 | 1.7141E-11 |
| AT5G55700 | 1.861852513 | 6.4076E-45 | 2.906E-43 |
| AT5G55720 | -7.226442181 | 1.4288E-05 | 6.1757E-05 |
| AT5G55730 | -1.335139048 | 1.4977E-22 | 2.9037E-21 |
| AT5G55830 | -1.681457121 | 0.00040144 | 0.00132889 |
| AT5G55896 | 1.057316086 | 4.5021E-17 | 6.1771E-16 |
| AT5G56040 | -1.285468336 | 1.8218E-21 | 3.3208E-20 |
| AT5G56080 | -3.063273822 | 1.6959E-33 | 5.2814E-32 |
| AT5G56150 | 1.079765267 | 6.691E-18 | 9.7107E-17 |
| AT5G56160 | 1.584773433 | 6.184E-07 | 3.2361E-06 |
| AT5G56190 | 1.277577869 | 2.5128E-23 | 5.083E-22 |
| AT5G56210 | 1.213013159 | 1.8907E-11 | 1.6603E-10 |
| AT5G56320 | -2.371776757 | 7.4074E-14 | 8.0436E-13 |
| AT5G56340 | 1.066360522 | 5.4702E-13 | 5.5083E-12 |
| AT5G56490 | -1.78810757 | 0.00031066 | 0.00105169 |
| AT5G56530 | -1.319455058 | 1.7985E-14 | 2.0422E-13 |
| AT5G56750 | 1.484014376 | 1.1579E-21 | 2.1448E-20 |
| AT5G56850 | -1.506013689 | 1.8846E-16 | 2.4887E-15 |
| AT5G56860 | -1.919458505 | 9.9152E-32 | 2.8835E-30 |
| AT5G56870 | -1.747765306 | 9.2792E-11 | 7.6132E-10 |
| AT5G56960 | -4.414573884 | 3.7403E-06 | 1.7613E-05 |
| AT5G56970 | -3.934475408 | 0.00722665 | 0.01800204 |
| AT5G57010 | -1.172457129 | 0.00021212 | 0.00074219 |
| AT5G57040 | 1.90565569 | 5.1945E-53 | 2.9758E-51 |
| AT5G57050 | 3.707147791 | 1.982E-249 | 1.742E-246 |
| AT5G57060 | 1.068208852 | 3.208E-08 | 2.0008E-07 |
| AT5G57170 | -1.018438615 | 2.1446E-09 | 1.5214E-08 |
| AT5G57180 | -1.119148648 | 6.5111E-14 | 7.0974E-13 |
| AT5G57220 | -4.238349543 | 1.205E-136 | 2.929E-134 |
| AT5G57350 | 1.150038857 | 7.9761E-27 | 1.868E-25 |
| AT5G57530 | -5.56437396 | 2.705E-06 | 1.3006E-05 |
| AT5G57540 | -5.763050585 | 8.4384E-07 | 4.3433E-06 |
| AT5G57560 | 1.871855081 | 8.8501E-25 | 1.9102E-23 |
| AT5G57610 | 2.016503009 | 2.2605E-54 | 1.3491E-52 |
| AT5G57620 | -1.361910934 | 0.00227495 | 0.00640878 |
| AT5G57625 | -5.894076903 | 9.1903E-18 | 1.3162E-16 |
| AT5G57660 | 1.516128715 | 1.5111E-21 | 2.772E-20 |
| AT5G57685 | -2.114762254 | 2.4057E-24 | 5.1152E-23 |
| AT5G57770 | -2.702367222 | 0.00028133 | 0.00095964 |
| AT5G57780 | -1.895092857 | 5.1025E-09 | 3.4569E-08 |
| AT5G57785 | -1.377417888 | 3.6605E-12 | 3.4207E-11 |
| AT5G57800 | 2.254423189 | 8.2943E-98 | 1.2392E-95 |
| AT5G57810 | 3.063679165 | 1.7218E-05 | 7.3291E-05 |
| AT5G57900 | 1.760272869 | 1.8057E-36 | 6.2443E-35 |
| AT5G57920 | -2.265684698 | 0.00150946 | 0.00442065 |
| AT5G58010 | -2.026648947 | 0.00053928 | 0.00174233 |
| AT5G58020 | 1.200206485 | 1.306E-21 | 2.4094E-20 |
| AT5G58040 | 1.476888964 | 7.5849E-43 | 3.1812E-41 |
| AT5G58070 | 1.489968832 | 3.5645E-38 | 1.2995E-36 |
| AT5G58160 | 2.029068121 | 5.1216E-43 | 2.168E-41 |
| AT5G58320 | 1.092197462 | 1.0989E-12 | 1.0794E-11 |
| AT5G58370 | -1.135882662 | 1.0302E-08 | 6.7999E-08 |
| AT5G58375 | -1.051873946 | 3.5228E-10 | 2.7167E-09 |
| AT5G58380 | 1.271373961 | 3.7878E-22 | 7.2272E-21 |
| AT5G58480 | -1.085305132 | 8.9402E-07 | 4.583E-06 |
| AT5G58500 | -3.332208163 | 6.9982E-06 | 3.1682E-05 |
| AT5G58620 | 1.533161001 | 8.7551E-33 | 2.6612E-31 |
| AT5G58650 | 1.232568748 | 7.8992E-06 | 3.5538E-05 |
| AT5G58660 | 1.222501174 | 0.00519504 | 0.01343911 |
| AT5G58700 | 1.323634171 | 3.8499E-19 | 6.0649E-18 |
| AT5G58750 | -2.384678242 | 4.3875E-05 | 0.00017454 |
| AT5G58940 | -1.242189508 | 0.00017515 | 0.0006233 |
| AT5G59020 | -1.798252267 | 1.2822E-10 | 1.0363E-09 |
| AT5G59030 | 1.264573828 | 2.1301E-19 | 3.4073E-18 |
| AT5G59080 | 1.597014537 | 3.2477E-08 | 2.0244E-07 |
| AT5G59220 | 5.402479162 | 0 | 0 |
| AT5G59230 | 4.951917074 | 0.00088062 | 0.0027198 |
| AT5G59310 | 5.585802049 | 0 | 0 |
| AT5G59320 | 4.328241646 | 1.035E-34 | 3.3464E-33 |
| AT5G59340 | 2.562499873 | 1.6189E-09 | 1.1684E-08 |
| AT5G59480 | 1.73102723 | 4.9934E-49 | 2.6239E-47 |
| AT5G59490 | 2.446235824 | 8.5774E-18 | 1.2347E-16 |
| AT5G59520 | -2.362730325 | 7.9606E-29 | 2.0331E-27 |
| AT5G59530 | -1.666080492 | 3.4566E-09 | 2.3936E-08 |
| AT5G59550 | 1.319492478 | 1.5243E-19 | 2.4538E-18 |
| AT5G59570 | 1.548485866 | 1.3429E-25 | 3.0153E-24 |
| AT5G59680 | -2.64530324 | 4.877E-10 | 3.7159E-09 |
| AT5G59720 | 1.31168606 | 9.7222E-05 | 0.00036395 |
| AT5G59760 | 2.138550354 | 0.00032841 | 0.00110671 |
| AT5G59770 | -1.062939298 | 2.046E-09 | 1.456E-08 |
| AT5G59820 | 1.247343166 | 1.488E-19 | 2.3987E-18 |
| AT5G59845 | 1.282183468 | 0.00246039 | 0.00688181 |
| AT5G59940 | -2.478324538 | 0.00127115 | 0.00378579 |
| AT5G59960 | 1.268555531 | 9.713E-24 | 2.0165E-22 |
| AT5G60360 | 1.547153762 | 6.516E-48 | 3.2735E-46 |
| AT5G60380 | 5.945873524 | 0.00966118 | 0.02327522 |
| AT5G60490 | -2.18147345 | 1.0726E-06 | 5.4374E-06 |
| AT5G60520 | -3.235524899 | 3.0774E-07 | 1.6788E-06 |
| AT5G60530 | -2.876414965 | 4.6378E-35 | 1.5209E-33 |
| AT5G60610 | 2.136349446 | 4.1753E-05 | 0.00016671 |
| AT5G60630 | 2.629599587 | 8.0216E-06 | 3.6023E-05 |
| AT5G60680 | 2.500290535 | 7.9542E-89 | 1.0449E-86 |
| AT5G60760 | 1.321579129 | 1.525E-07 | 8.6797E-07 |
| AT5G60790 | 1.760964889 | 4.1015E-72 | 3.8741E-70 |
| AT5G60800 | 1.079854537 | 2.196E-09 | 1.5545E-08 |
| AT5G60850 | 1.290157366 | 9.7585E-16 | 1.2263E-14 |
| AT5G60880 | -1.655924483 | 3.2282E-05 | 0.00013149 |
| AT5G61000 | -1.311190339 | 2.7525E-11 | 2.376E-10 |
| AT5G61130 | -1.390230837 | 3.1396E-18 | 4.6691E-17 |
| AT5G61250 | -1.184567151 | 0.00039262 | 0.00130237 |
| AT5G61310 | -1.117790813 | 2.5813E-08 | 1.6281E-07 |
| AT5G61340 | -1.069924223 | 3.4984E-07 | 1.895E-06 |
| AT5G61412 | -3.374157686 | 0.003768 | 0.01006415 |
| AT5G61420 | -2.444456615 | 1.9249E-65 | 1.5277E-63 |
| AT5G61440 | -2.473711268 | 2.1628E-18 | 3.2482E-17 |
| AT5G61450 | 1.036741946 | 7.0205E-10 | 5.2718E-09 |
| AT5G61455 | 1.891147257 | 0.00159287 | 0.00463818 |
| AT5G61530 | 1.431960041 | 1.94E-33 | 6.0168E-32 |
| AT5G61560 | 2.25366652 | 1.9782E-32 | 5.9187E-31 |
| AT5G61790 | -1.559511862 | 6.9961E-37 | 2.4603E-35 |
| AT5G61810 | 1.432778161 | 6.231E-28 | 1.5184E-26 |
| AT5G61820 | 2.648355116 | 1.7785E-96 | 2.606E-94 |
| AT5G61865 | 1.231940725 | 0.00035643 | 0.00119253 |
| AT5G61890 | 1.849146541 | 1.1499E-06 | 5.8071E-06 |
| AT5G62020 | 2.454262496 | 9.7396E-59 | 6.5096E-57 |
| AT5G62040 | 2.304525743 | 4.0465E-05 | 0.00016199 |
| AT5G62090 | 1.546164289 | 4.2719E-38 | 1.5549E-36 |
| AT5G62100 | 1.587640154 | 2.1895E-15 | 2.6806E-14 |
| AT5G62150 | -1.308445273 | 4.0508E-07 | 2.173E-06 |
| AT5G62210 | -2.459482701 | 1.9663E-79 | 2.1608E-77 |
| AT5G62220 | 1.1461826 | 7.3034E-21 | 1.2763E-19 |
| AT5G62280 | -1.624850617 | 8.3875E-05 | 0.00031737 |
| AT5G62310 | -1.83744297 | 0.00434249 | 0.01144068 |
| AT5G62330 | -5.533328828 | 0.00890713 | 0.02165028 |
| AT5G62340 | -1.513445841 | 0.00027546 | 0.0009416 |
| AT5G62360 | -2.102731769 | 1.3256E-24 | 2.8478E-23 |
| AT5G62420 | -1.870424087 | 0.0023196 | 0.00652251 |
| AT5G09065 | 1.779822716 | 0.00137626 | 0.00406493 |
| AT5G62430 | 1.97609513 | 8.0631E-24 | 1.6816E-22 |
| AT5G62470 | 3.10853719 | 7.0581E-82 | 8.2313E-80 |
| AT5G62520 | 4.932436899 | 6.583E-106 | 1.106E-103 |
| AT5G62540 | 2.164223154 | 5.0508E-65 | 3.9811E-63 |
| AT5G62630 | -1.342867465 | 4.8179E-12 | 4.4586E-11 |
| AT5G62730 | -5.487936547 | 0.0086588 | 0.02112304 |
| AT5G62800 | 4.651653694 | 0.00243903 | 0.00682975 |
| AT5G63087 | -4.834864661 | 0.00018735 | 0.00066291 |
| AT5G63130 | 3.034973624 | 6.3821E-44 | 2.8E-42 |
| AT5G63140 | -1.50358924 | 2.8256E-13 | 2.9133E-12 |
| AT5G63160 | 2.641976008 | 2.61E-104 | 4.261E-102 |
| AT5G63180 | -2.519982913 | 5.2925E-43 | 2.2362E-41 |
| AT5G63190 | 2.228882045 | 1.3064E-47 | 6.5059E-46 |
| AT5G63270 | -2.430874016 | 0.0037383 | 0.009994 |
| AT5G63320 | 1.450710147 | 4.0529E-21 | 7.2264E-20 |
| AT5G63350 | 4.110057489 | 3.9172E-07 | 2.1063E-06 |
| AT5G63370 | 1.534939876 | 1.8869E-41 | 7.5537E-40 |
| AT5G63410 | -1.224614605 | 1.1127E-10 | 9.048E-10 |
| AT5G63450 | 1.395039905 | 3.054E-15 | 3.6995E-14 |
| AT5G63590 | -1.280611669 | 7.4245E-08 | 4.4172E-07 |
| AT5G63595 | -1.748342724 | 0.00018112 | 0.00064275 |
| AT5G63600 | -1.017376975 | 1.9847E-18 | 2.9944E-17 |
| AT5G63660 | -3.075480298 | 2.0559E-12 | 1.9703E-11 |
| AT5G63780 | -1.618224533 | 4.479E-29 | 1.1661E-27 |
| AT5G63790 | 1.620426426 | 7.3783E-62 | 5.3541E-60 |
| AT5G63820 | 1.37329336 | 0.00953897 | 0.02303176 |
| AT5G63880 | 1.30271613 | 2.6571E-16 | 3.4707E-15 |
| AT5G64080 | 1.33787853 | 9.5331E-21 | 1.6583E-19 |
| AT5G64100 | -1.902621851 | 1.8377E-56 | 1.1572E-54 |
| AT5G64120 | -3.601107017 | 1.022E-169 | 3.707E-167 |
| AT5G64170 | 1.710673752 | 7.0239E-33 | 2.1436E-31 |
| AT5G64220 | 1.124740979 | 7.575E-18 | 1.0945E-16 |
| AT5G64230 | 3.165088502 | 1.2518E-76 | 1.2831E-74 |
| AT5G64250 | 1.938106148 | 3.5592E-69 | 3.1533E-67 |
| AT5G64260 | 1.850707734 | 3.5446E-56 | 2.2137E-54 |
| AT5G64310 | 2.428362107 | 7.664E-120 | 1.51E-117 |
| AT5G64430 | 2.513894945 | 1.5877E-75 | 1.5848E-73 |
| AT5G64620 | -1.43421999 | 1.4072E-17 | 1.9932E-16 |
| AT5G64750 | 5.229451458 | 2.888E-200 | 1.61E-197 |
| AT5G64770 | -1.296419175 | 1.1689E-14 | 1.3468E-13 |
| AT5G64840 | 1.347314637 | 8.7287E-36 | 2.9341E-34 |
| AT5G64890 | -3.29954547 | 0.00020573 | 0.00072181 |
| AT5G64905 | -2.736097144 | 9.7792E-14 | 1.0559E-12 |
| AT5G65090 | -3.346804755 | 4.5119E-05 | 0.00017911 |
| AT5G65110 | 1.828608229 | 7.011E-48 | 3.5144E-46 |
| AT5G65140 | 2.723984382 | 6.6944E-67 | 5.4846E-65 |
| AT5G65160 | -1.947255199 | 9.2542E-09 | 6.1367E-08 |
| AT5G65170 | -1.576018896 | 0.00031779 | 0.0010736 |
| AT5G65207 | 3.288907361 | 8.4292E-69 | 7.4105E-67 |
| AT5G65300 | 2.008711337 | 1.3481E-20 | 2.3151E-19 |
| AT5G65310 | -1.184412009 | 2.553E-29 | 6.7544E-28 |
| AT5G65410 | -1.932712518 | 2.3367E-28 | 5.8439E-27 |
| AT5G65470 | 1.178160771 | 1.7982E-25 | 4.0258E-24 |
| AT5G65510 | -1.591153115 | 0.00798462 | 0.01966305 |
| AT5G65530 | -1.739109734 | 2.3572E-07 | 1.3091E-06 |
| AT5G65600 | -2.30291961 | 8.9827E-09 | 5.9654E-08 |
| AT5G65640 | 1.237156507 | 2.2011E-17 | 3.0772E-16 |
| AT5G65683 | -1.763807049 | 1.3747E-28 | 3.4761E-27 |
| AT5G65690 | -2.516295343 | 0.00048113 | 0.00156839 |
| AT5G65700 | -1.206130443 | 1.7627E-25 | 3.9503E-24 |
| AT5G65730 | -4.299631699 | 1.2703E-37 | 4.5582E-36 |
| AT5G65790 | -1.333152797 | 1.1115E-06 | 5.6223E-06 |
| AT5G65800 | -4.226286407 | 0.0058697 | 0.01498031 |
| AT5G65925 | 1.316299456 | 4.2437E-09 | 2.913E-08 |
| AT5G65970 | -2.322439187 | 3.3548E-12 | 3.1506E-11 |
| AT5G65980 | -1.154796181 | 0.00868005 | 0.02116583 |
| AT5G65990 | 1.828706679 | 6.6947E-64 | 5.1698E-62 |
| AT5G66020 | 5.429285365 | 0.00017183 | 0.00061276 |
| AT5G66050 | 1.217452809 | 1.8607E-17 | 2.6174E-16 |
| AT5G66052 | 1.224131339 | 1.2761E-14 | 1.4658E-13 |
| AT5G66070 | 2.633760742 | 1.0202E-63 | 7.8518E-62 |
| AT5G66080 | 1.253367481 | 1.0088E-06 | 5.1274E-06 |
| AT5G66110 | 2.506854649 | 0.00011487 | 0.00042397 |
| AT5G66230 | -1.415716093 | 8.4851E-15 | 9.9361E-14 |
| AT5G66400 | 5.273186679 | 0 | 0 |
| AT5G66460 | 1.702298177 | 9.8303E-30 | 2.6467E-28 |
| AT5G66480 | 1.2136812 | 0.0001085 | 0.00040262 |
| AT5G66590 | -1.022829224 | 0.00309597 | 0.00843176 |
| AT5G66630 | -1.284305902 | 0.00472489 | 0.01234162 |
| AT5G66650 | 1.354973106 | 3.3964E-14 | 3.765E-13 |
| AT5G66690 | -1.184426089 | 1.4227E-16 | 1.8962E-15 |
| AT5G66700 | 1.775661873 | 6.956E-05 | 0.00026693 |
| AT5G66760 | 1.172427975 | 2.481E-36 | 8.5022E-35 |
| AT5G66770 | -1.350511981 | 8.9597E-13 | 8.8582E-12 |
| AT5G66780 | 5.023924839 | 1.279E-163 | 4.3E-161 |
| AT5G66890 | -5.487936547 | 0.0086588 | 0.02112304 |
| AT5G66920 | -1.167499994 | 1.0604E-13 | 1.1401E-12 |
| AT5G66940 | -1.859781951 | 0.00029643 | 0.00100712 |
| AT5G66985 | -2.509194899 | 2.2236E-08 | 1.4154E-07 |
| AT5G67030 | 2.098350438 | 8.7883E-71 | 7.9715E-69 |
| AT5G67080 | 3.45137794 | 7.6931E-17 | 1.043E-15 |
| AT5G67090 | 1.778496971 | 7.159E-09 | 4.7848E-08 |
| AT5G67150 | -1.528031502 | 1.092E-16 | 1.4658E-15 |
| AT5G67190 | 1.094604723 | 0.00012103 | 0.00044471 |
| AT5G67200 | -1.445677106 | 1.2973E-10 | 1.0478E-09 |
| AT5G67230 | 1.002482463 | 0.00097049 | 0.00297364 |
| AT5G67280 | -1.388573046 | 2.3925E-18 | 3.5791E-17 |
| AT5G67300 | 1.628888505 | 3.9877E-49 | 2.1051E-47 |
| AT5G67310 | 4.152119589 | 2.1895E-16 | 2.878E-15 |
| AT5G67350 | 1.131784908 | 2.4357E-08 | 1.5401E-07 |
| AT5G67390 | -1.30092787 | 0.00020852 | 0.00073083 |
| AT5G67400 | -3.424212173 | 2.0129E-19 | 3.2221E-18 |
| AT5G67480 | 1.524220063 | 6.3167E-31 | 1.7826E-29 |
| AT5G67510 | -1.140059405 | 2.8778E-19 | 4.5713E-18 |
| AT5G67520 | 1.54362182 | 0.00010879 | 0.00040361 |
| AT5G67600 | 1.506396974 | 1.1804E-34 | 3.8111E-33 |
| AT5G67620 | 1.255283624 | 2.7021E-05 | 0.00011151 |
