# Supplementary material for: MYB44-ENAP1/2 restricts HDT4 to regulate drought tolerance in Arabidopsis
Source: PLoS Genet. 2022 Nov 22;18(11):e1010473. doi: 10.1371/journal.pgen.1010473 (PMC9681084; doi:10.1371/journal.pgen.1010473)
Supplement: S3 Table — (DOCX) [file pgen.1010473.s009.docx]

Table S3. Differentially regulated genes in enap1-1enap2 by dehydration.

| AGI | log2FoldChange | pvalue | padj |
| --- | --- | --- | --- |
| AT1G01080 | -1.370476054 | 3.715E-46 | 6.1915E-45 |
| AT1G01120 | 1.385924453 | 6.4907E-14 | 3.5471E-13 |
| AT1G01180 | -1.073762797 | 7.5398E-06 | 2.2338E-05 |
| AT1G01190 | -2.63906715 | 1.9001E-24 | 1.6778E-23 |
| AT1G01200 | -2.558773009 | 1.5022E-07 | 5.2723E-07 |
| AT1G01240 | 3.746216225 | 1.745E-193 | 1.826E-191 |
| AT1G01250 | 1.912494144 | 1.1036E-16 | 6.9425E-16 |
| AT1G01300 | -1.610433587 | 4.346E-77 | 1.3064E-75 |
| AT1G01320 | -1.165412804 | 3.325E-85 | 1.1477E-83 |
| AT1G01360 | 1.126265922 | 7.7101E-11 | 3.4759E-10 |
| AT1G01380 | 1.495699626 | 0.0002353 | 0.0005831 |
| AT1G01390 | -1.198340608 | 0.00047322 | 0.00112271 |
| AT1G01430 | -1.266101647 | 1.8964E-26 | 1.7939E-25 |
| AT1G01453 | -4.883899959 | 0.00024096 | 0.00059622 |
| AT1G01470 | 3.696605386 | 0 | 0 |
| AT1G01480 | 3.227773485 | 1.8258E-52 | 3.5574E-51 |
| AT1G01520 | 3.695158247 | 5.6415E-15 | 3.2644E-14 |
| AT1G01570 | 2.326606207 | 4.4611E-18 | 3.0203E-17 |
| AT1G01650 | 1.332607454 | 1.384E-52 | 2.699E-51 |
| AT1G01700 | 1.081281076 | 0.00238119 | 0.00507892 |
| AT1G01710 | 1.038745961 | 4.7204E-24 | 4.1075E-23 |
| AT1G01720 | 2.668309185 | 0 | 0 |
| AT1G01830 | 1.454596701 | 1.4749E-33 | 1.7481E-32 |
| AT1G02150 | -1.185703596 | 1.6473E-56 | 3.4931E-55 |
| AT1G02205 | 3.800281578 | 0 | 0 |
| AT1G02220 | 1.379066855 | 9.6818E-38 | 1.3102E-36 |
| AT1G02310 | 3.298820216 | 1.1051E-26 | 1.0581E-25 |
| AT1G02340 | 1.703125664 | 3.8914E-08 | 1.4362E-07 |
| AT1G02360 | -2.164812619 | 1.586E-67 | 4.1166E-66 |
| AT1G02370 | -1.727803619 | 7.953E-19 | 5.585E-18 |
| AT1G02390 | 2.848491596 | 6.0222E-31 | 6.625E-30 |
| AT1G02400 | 2.417524437 | 3.821E-89 | 1.4176E-87 |
| AT1G02460 | -1.916279129 | 3.6113E-06 | 1.1045E-05 |
| AT1G02470 | 3.74054269 | 6.147E-12 | 2.9595E-11 |
| AT1G02610 | 2.047261717 | 8.8176E-07 | 2.8683E-06 |
| AT1G02640 | -2.307184908 | 1.6325E-19 | 1.1832E-18 |
| AT1G02660 | 4.691459075 | 4.246E-258 | 6.823E-256 |
| AT1G02670 | 1.815204763 | 1.5135E-12 | 7.5776E-12 |
| AT1G02700 | 2.843324061 | 1.1185E-31 | 1.2615E-30 |
| AT1G02750 | 1.111184894 | 1.4981E-09 | 6.2045E-09 |
| AT1G02800 | -1.619941961 | 1.5146E-05 | 4.3567E-05 |
| AT1G02810 | -2.460500791 | 7.0194E-18 | 4.7077E-17 |
| AT1G02816 | 1.966123441 | 2.7489E-56 | 5.7858E-55 |
| AT1G02820 | 3.742085464 | 2.8278E-85 | 9.7757E-84 |
| AT1G02900 | -1.479600019 | 2.8546E-18 | 1.9524E-17 |
| AT1G02920 | -1.799465775 | 2.953E-128 | 1.723E-126 |
| AT1G02980 | 1.906373289 | 0.0035259 | 0.00729678 |
| AT1G03070 | 3.669339804 | 3.5345E-50 | 6.5192E-49 |
| AT1G03080 | 1.161589545 | 6.8934E-58 | 1.5109E-56 |
| AT1G03090 | 1.272137998 | 2.3657E-30 | 2.5484E-29 |
| AT1G03110 | -1.025623401 | 2.6519E-13 | 1.3913E-12 |
| AT1G03120 | 2.769507208 | 9.7964E-07 | 3.1704E-06 |
| AT1G03180 | 1.008592327 | 5.5307E-05 | 0.00014909 |
| AT1G03190 | 1.00853435 | 4.6612E-14 | 2.567E-13 |
| AT1G03290 | 2.068555373 | 2.4976E-96 | 1.0213E-94 |
| AT1G03520 | 1.937102017 | 5.797E-26 | 5.3875E-25 |
| AT1G08825 | 1.295135559 | 1.9007E-05 | 5.4099E-05 |
| AT1G03610 | 1.773154822 | 4.4709E-39 | 6.2275E-38 |
| AT1G03750 | 1.593508603 | 1.3535E-14 | 7.6646E-14 |
| AT1G03770 | 1.037647295 | 3.4222E-10 | 1.4838E-09 |
| AT1G03790 | 5.957449814 | 0.00528094 | 0.01060837 |
| AT1G03840 | -2.018068921 | 4.798E-08 | 1.7566E-07 |
| AT1G03870 | -4.561954447 | 3.803E-161 | 3.088E-159 |
| AT1G03940 | 1.12968877 | 1.8929E-22 | 1.5441E-21 |
| AT1G03970 | 1.130011972 | 1.5298E-10 | 6.7682E-10 |
| AT1G03990 | 1.561616473 | 7.3856E-08 | 2.6654E-07 |
| AT1G04040 | -2.20965033 | 8.6991E-49 | 1.547E-47 |
| AT1G04100 | 1.124236886 | 0.00061305 | 0.00142903 |
| AT1G04180 | 2.315986261 | 6.0904E-16 | 3.6928E-15 |
| AT1G04220 | 4.073813983 | 2.965E-218 | 3.56E-216 |
| AT1G04247 | 4.340020335 | 5.2126E-78 | 1.5879E-76 |
| AT1G04310 | 2.018044151 | 8.8143E-41 | 1.2867E-39 |
| AT1G04330 | -2.225071405 | 0.00050248 | 0.00118631 |
| AT1G04430 | -1.289121571 | 8.9855E-64 | 2.1927E-62 |
| AT1G04490 | 1.39295763 | 0.0089107 | 0.01713065 |
| AT1G04520 | -2.399404739 | 2.5954E-45 | 4.2357E-44 |
| AT1G04540 | -2.281116794 | 9.537E-06 | 2.7987E-05 |
| AT1G04550 | -1.099357716 | 3.6216E-11 | 1.6646E-10 |
| AT1G04560 | 4.476138577 | 3.7842E-19 | 2.6973E-18 |
| AT1G04570 | 2.394478575 | 4.3092E-12 | 2.095E-11 |
| AT1G04600 | 1.4620846 | 0.00863342 | 0.01664387 |
| AT1G04610 | -1.430664396 | 0.0007738 | 0.00178027 |
| AT1G04780 | 1.039966288 | 7.9418E-36 | 1.0146E-34 |
| AT1G04800 | -1.459160447 | 2.7831E-24 | 2.4451E-23 |
| AT1G04830 | 2.267031086 | 8.6389E-88 | 3.1436E-86 |
| AT1G04980 | -1.326318658 | 3.5634E-22 | 2.8658E-21 |
| AT1G04990 | 1.45537392 | 2.3349E-54 | 4.7061E-53 |
| AT1G05000 | -1.678501865 | 3.1642E-13 | 1.6502E-12 |
| AT1G05010 | -1.017795582 | 1.9081E-48 | 3.3644E-47 |
| AT1G05035 | -2.438905769 | 0.00377612 | 0.0077765 |
| AT1G04317 | -2.681413236 | 0.00157888 | 0.00346616 |
| AT1G05100 | 6.572935896 | 0 | 0 |
| AT1G05170 | 1.189139964 | 2.91E-16 | 1.792E-15 |
| AT1G05210 | -1.672526373 | 5.3308E-08 | 1.9448E-07 |
| AT1G05260 | 1.121543799 | 8.8197E-30 | 9.3769E-29 |
| AT1G05300 | -1.769885752 | 2.1806E-08 | 8.24E-08 |
| AT1G05340 | 5.101296302 | 0 | 0 |
| AT1G05450 | 2.675109764 | 1.6614E-07 | 5.7997E-07 |
| AT1G05510 | 5.885401186 | 0.00371751 | 0.0076641 |
| AT1G05540 | -1.058366002 | 8.3548E-05 | 0.00021956 |
| AT1G05590 | -1.166309464 | 1.9635E-11 | 9.1688E-11 |
| AT1G05650 | -1.840073179 | 0.00032429 | 0.0007878 |
| AT1G05660 | -1.329097553 | 0.00367759 | 0.00758935 |
| AT1G05675 | -1.454935537 | 0.00098239 | 0.00222186 |
| AT1G05680 | 1.435263601 | 1.0584E-79 | 3.3079E-78 |
| AT1G05690 | 1.208124469 | 0.00044876 | 0.001068 |
| AT1G05700 | -2.538206635 | 5.7574E-14 | 3.1577E-13 |
| AT1G05880 | -2.869978558 | 0.00049091 | 0.00116117 |
| AT1G05890 | 1.144343384 | 7.1822E-45 | 1.1621E-43 |
| AT1G06080 | -1.506667583 | 1.3408E-06 | 4.2823E-06 |
| AT1G06110 | 1.154712896 | 2.0942E-26 | 1.9785E-25 |
| AT1G06120 | -3.750290894 | 0.00088802 | 0.0020251 |
| AT1G06160 | -2.36582857 | 1.0412E-06 | 3.3591E-06 |
| AT1G06210 | 1.095593116 | 3.2478E-28 | 3.2978E-27 |
| AT1G06330 | -6.065436781 | 0.00244265 | 0.0051969 |
| AT1G06380 | -1.463750425 | 1.3156E-13 | 7.0328E-13 |
| AT1G06430 | 1.825707224 | 8.3556E-97 | 3.4537E-95 |
| AT1G06570 | 2.146155175 | 5.318E-109 | 2.517E-107 |
| AT1G06700 | 1.012338535 | 4.1299E-36 | 5.3117E-35 |
| AT1G06710 | -1.145911445 | 2.0661E-07 | 7.1521E-07 |
| AT1G06780 | 1.418068305 | 3.7051E-39 | 5.1736E-38 |
| AT1G06790 | -1.60965274 | 6.1055E-13 | 3.1367E-12 |
| AT1G06830 | -5.394974299 | 9.8652E-18 | 6.5584E-17 |
| AT1G06923 | -6.354075023 | 0.00083568 | 0.00191301 |
| AT1G06930 | -3.396133609 | 0.00884963 | 0.01702328 |
| AT1G06950 | -1.229750508 | 1.5341E-83 | 5.0876E-82 |
| AT1G06980 | -1.470836566 | 0.00139599 | 0.00308484 |
| AT1G07040 | 2.32240648 | 2.02E-127 | 1.173E-125 |
| AT1G07050 | -1.946011253 | 2.6946E-64 | 6.6609E-63 |
| AT1G07070 | -1.134607345 | 3.1308E-15 | 1.833E-14 |
| AT1G07080 | 1.158830239 | 1.6985E-55 | 3.5198E-54 |
| AT1G07090 | -1.923398197 | 2.0637E-19 | 1.4891E-18 |
| AT1G07150 | 2.001084568 | 1.9579E-27 | 1.9255E-26 |
| AT1G07160 | 1.563751861 | 0.00574624 | 0.01145732 |
| AT1G07370 | -1.978300124 | 3.1069E-40 | 4.4837E-39 |
| AT1G07400 | 1.897406737 | 5.4218E-58 | 1.1906E-56 |
| AT1G07430 | 6.059693493 | 5.4656E-83 | 1.7995E-81 |
| AT1G07470 | 1.27581521 | 7.3982E-26 | 6.8644E-25 |
| AT1G07480 | 1.524461742 | 1.0558E-28 | 1.091E-27 |
| AT1G07540 | 5.927740173 | 0.00021501 | 0.00053561 |
| AT1G07560 | -1.642851464 | 0.00012302 | 0.0003177 |
| AT1G07590 | 1.54879232 | 4.771E-130 | 2.842E-128 |
| AT1G07610 | 1.402962203 | 3.7605E-85 | 1.2941E-83 |
| AT1G07720 | 2.750486713 | 1.622E-217 | 1.928E-215 |
| AT1G07870 | 1.713263577 | 2.5277E-31 | 2.8132E-30 |
| AT1G07900 | 6.396378359 | 1.318E-168 | 1.143E-166 |
| AT1G07980 | 1.2748854 | 3.1624E-25 | 2.8587E-24 |
| AT1G07985 | 5.408284999 | 8.685E-269 | 1.49E-266 |
| AT1G08040 | 1.037923815 | 9.1723E-23 | 7.5742E-22 |
| AT1G08090 | -6.54425794 | 1.446E-108 | 6.832E-107 |
| AT1G08180 | 1.01584376 | 6.1158E-05 | 0.00016386 |
| AT1G08220 | -1.386270058 | 8.4015E-13 | 4.2902E-12 |
| AT1G08230 | 1.122320587 | 1.1102E-28 | 1.1456E-27 |
| AT1G08280 | -1.960880304 | 1.1783E-25 | 1.0836E-24 |
| AT1G08410 | -1.010984089 | 2.3243E-15 | 1.3703E-14 |
| AT1G08430 | -1.092643386 | 6.2427E-07 | 2.0568E-06 |
| AT1G08440 | 3.956556061 | 1.8077E-10 | 7.967E-10 |
| AT1G08500 | 2.369002496 | 9.7777E-38 | 1.3224E-36 |
| AT1G08570 | 2.390251353 | 1.399E-182 | 1.341E-180 |
| AT1G08580 | -1.553670878 | 2.8417E-44 | 4.5371E-43 |
| AT1G08610 | -2.061521633 | 4.6469E-19 | 3.2968E-18 |
| AT1G08630 | 3.603566242 | 1.3706E-63 | 3.3197E-62 |
| AT1G08650 | 1.090561326 | 6.4455E-20 | 4.7301E-19 |
| AT1G08670 | -3.134513991 | 7.4434E-05 | 0.00019715 |
| AT1G08770 | 1.093983165 | 9.6417E-11 | 4.3245E-10 |
| AT1G08810 | -1.835804555 | 5.9104E-11 | 2.6852E-10 |
| AT1G08890 | 2.078015406 | 4.901E-92 | 1.8825E-90 |
| AT1G08920 | 2.258433787 | 5.602E-143 | 3.77E-141 |
| AT1G08930 | -1.460249316 | 2.6415E-56 | 5.57E-55 |
| AT1G08990 | -2.025732583 | 2.5386E-08 | 9.5234E-08 |
| AT1G09070 | 1.007647535 | 3.3907E-45 | 5.5141E-44 |
| AT1G09090 | -2.702372195 | 0.00031619 | 0.00076928 |
| AT1G09140 | 1.326585175 | 2.1943E-77 | 6.6225E-76 |
| AT1G09170 | -1.823259332 | 1.7094E-06 | 5.3936E-06 |
| AT1G09180 | 2.875713242 | 2.234E-11 | 1.0404E-10 |
| AT1G09190 | -1.300460064 | 2.5185E-06 | 7.8159E-06 |
| AT1G09240 | -1.329539452 | 7.441E-05 | 0.00019714 |
| AT1G09350 | 1.89736993 | 0.00034316 | 0.00083108 |
| AT1G09390 | -1.294833293 | 0.00040339 | 0.00096728 |
| AT1G09440 | -1.05467887 | 1.8278E-05 | 5.2079E-05 |
| AT1G09460 | -1.046795465 | 0.00016775 | 0.00042469 |
| AT1G09490 | 2.164814556 | 6.083E-125 | 3.402E-123 |
| AT1G09500 | 5.418579292 | 0 | 0 |
| AT1G09510 | 6.38015601 | 0.00077275 | 0.00177821 |
| AT1G09520 | 1.016928709 | 1.8215E-14 | 1.0259E-13 |
| AT1G09530 | 2.814376167 | 8.251E-136 | 5.102E-134 |
| AT1G09590 | -1.115560153 | 3.6669E-72 | 1.0215E-70 |
| AT1G09660 | -1.168956536 | 5.8953E-25 | 5.2872E-24 |
| AT1G09750 | -2.719554079 | 1.293E-242 | 1.855E-240 |
| AT1G09800 | -1.383496311 | 5.2482E-10 | 2.2512E-09 |
| AT1G09810 | -1.426859763 | 8.4324E-16 | 5.0737E-15 |
| AT1G09812 | -1.38055242 | 0.0091212 | 0.01749847 |
| AT1G09830 | -1.206640883 | 5.4135E-27 | 5.2315E-26 |
| AT1G09900 | -1.235154097 | 1.768E-22 | 1.4438E-21 |
| AT1G09920 | 1.090238454 | 2.0335E-24 | 1.7935E-23 |
| AT1G09950 | 3.784041091 | 1.4447E-39 | 2.0385E-38 |
| AT1G09960 | 1.205943117 | 1.3483E-15 | 8.0469E-15 |
| AT1G10020 | -2.216196502 | 1.1366E-27 | 1.131E-26 |
| AT1G10060 | 1.894347037 | 5.3777E-76 | 1.5893E-74 |
| AT1G10170 | 1.465794394 | 6.567E-102 | 2.865E-100 |
| AT1G10270 | -1.17294141 | 4.558E-21 | 3.4944E-20 |
| AT1G10280 | 1.256521142 | 3.8821E-10 | 1.6785E-09 |
| AT1G10385 | -5.594343472 | 0.00955208 | 0.01826214 |
| AT1G10400 | -2.096583031 | 0.00048739 | 0.00115343 |
| AT1G10460 | -2.514293463 | 0.0005932 | 0.00138503 |
| AT1G10470 | -1.326584886 | 2.4261E-44 | 3.8846E-43 |
| AT1G10480 | 1.018044156 | 0.00014304 | 0.00036612 |
| AT1G10490 | -1.267507917 | 3.1054E-31 | 3.4411E-30 |
| AT1G10522 | -1.57467795 | 1.275E-39 | 1.8035E-38 |
| AT1G10550 | -2.960444552 | 0.00536703 | 0.01076708 |
| AT1G10560 | 1.460396157 | 1.1101E-07 | 3.9373E-07 |
| AT1G10640 | 1.242257356 | 6.5836E-22 | 5.2411E-21 |
| AT1G10650 | 1.252460042 | 6.2282E-24 | 5.3949E-23 |
| AT1G10690 | 1.118950785 | 0.0001999 | 0.00050032 |
| AT1G10740 | 1.954050158 | 9.2987E-68 | 2.4192E-66 |
| AT1G10750 | -1.041326452 | 0.00114695 | 0.00256935 |
| AT1G10930 | -1.310617951 | 1.4533E-27 | 1.4386E-26 |
| AT1G10960 | -1.208068025 | 2.3212E-81 | 7.4698E-80 |
| AT1G10990 | -2.675915546 | 4.3164E-13 | 2.2362E-12 |
| AT1G11112 | -2.559973419 | 0.00014767 | 0.00037705 |
| AT1G11130 | -1.467309441 | 1.2043E-31 | 1.3529E-30 |
| AT1G11160 | -1.859492924 | 1.2575E-09 | 5.2403E-09 |
| AT1G11170 | 2.575695885 | 3.909E-64 | 9.6314E-63 |
| AT1G11175 | 2.365635781 | 6.8991E-31 | 7.5714E-30 |
| AT1G11185 | -4.201729112 | 0.0001208 | 0.00031224 |
| AT1G11210 | 2.177150208 | 1.5099E-58 | 3.3383E-57 |
| AT1G11340 | -2.434050328 | 4.8544E-08 | 1.7755E-07 |
| AT1G11350 | -1.880209306 | 1.6472E-31 | 1.8422E-30 |
| AT1G11400 | 1.192824015 | 4.8742E-22 | 3.9076E-21 |
| AT1G11430 | -1.031197558 | 3.1827E-42 | 4.8286E-41 |
| AT1G11450 | -1.68108193 | 1.1615E-09 | 4.8538E-09 |
| AT1G11460 | -2.50039137 | 0.00329304 | 0.00685093 |
| AT1G11480 | 1.031738449 | 4.5126E-34 | 5.4076E-33 |
| AT1G11540 | -3.05171373 | 4.1516E-06 | 1.2615E-05 |
| AT1G11545 | -1.825774816 | 9.7753E-23 | 8.0634E-22 |
| AT1G11655 | -6.331329312 | 0.00087772 | 0.00200261 |
| AT1G11670 | -2.888958755 | 1.7823E-79 | 5.5476E-78 |
| AT1G11700 | -1.316978971 | 7.0207E-13 | 3.5997E-12 |
| AT1G11740 | -3.88349516 | 0.00038024 | 0.00091514 |
| AT1G11785 | 2.192253291 | 0.00368469 | 0.00760195 |
| AT1G11820 | -1.339786392 | 1.9431E-29 | 2.0506E-28 |
| AT1G11850 | -1.369985287 | 1.0288E-17 | 6.8315E-17 |
| AT1G11940 | 1.298863107 | 1.4534E-12 | 7.2896E-12 |
| AT1G12030 | 1.340348139 | 0.00017169 | 0.00043391 |
| AT1G12040 | -7.288767036 | 2.8657E-21 | 2.2171E-20 |
| AT1G12110 | -1.760617877 | 2.2071E-85 | 7.6648E-84 |
| AT1G12380 | -1.073495646 | 8.9151E-07 | 2.8984E-06 |
| AT1G12420 | 2.219843915 | 6.8135E-57 | 1.4542E-55 |
| AT1G12460 | -1.161295091 | 2.0523E-10 | 9.029E-10 |
| AT1G12500 | -2.191958964 | 7.2702E-58 | 1.5919E-56 |
| AT1G12560 | -4.942760717 | 4.0365E-37 | 5.3513E-36 |
| AT1G12672 | 5.555630744 | 0.00973072 | 0.01857409 |
| AT1G12680 | 1.245813265 | 1.8298E-22 | 1.4932E-21 |
| AT1G12740 | -3.047092135 | 7.2212E-31 | 7.9211E-30 |
| AT1G12810 | 1.160767504 | 1.0436E-13 | 5.637E-13 |
| AT1G04973 | 1.795294422 | 0.0089892 | 0.01727137 |
| AT1G12845 | 2.006977172 | 8.2275E-51 | 1.5387E-49 |
| AT1G12880 | 1.306069848 | 8.0503E-09 | 3.1446E-08 |
| AT1G12900 | -1.351750789 | 4.029E-135 | 2.464E-133 |
| AT1G12950 | -1.164072208 | 4.2689E-06 | 1.2957E-05 |
| AT1G13020 | -1.108221752 | 1.8251E-25 | 1.6643E-24 |
| AT1G13100 | -1.145695758 | 5.3492E-06 | 1.6122E-05 |
| AT1G13110 | -2.111261832 | 1.274E-89 | 4.781E-88 |
| AT1G13195 | 1.163849133 | 2.6288E-14 | 1.469E-13 |
| AT1G13250 | -1.272110759 | 3.2941E-13 | 1.7163E-12 |
| AT1G13260 | -2.257864342 | 2.374E-33 | 2.8021E-32 |
| AT1G13340 | 1.181237806 | 5.5058E-05 | 0.00014851 |
| AT1G13370 | 2.923525118 | 4.7361E-08 | 1.7348E-07 |
| AT1G13420 | -2.204444037 | 7.9874E-16 | 4.8122E-15 |
| AT1G13430 | -4.842552556 | 0.00037938 | 0.00091337 |
| AT1G13448 | 2.161681372 | 0.00846371 | 0.01634984 |
| AT1G13450 | 1.116155383 | 7.0167E-15 | 4.0417E-14 |
| AT1G13480 | -2.344541637 | 8.9395E-08 | 3.2014E-07 |
| AT1G13510 | -4.634655775 | 6.3231E-16 | 3.8308E-15 |
| AT1G13530 | -1.929399412 | 0.00225076 | 0.00482327 |
| AT1G13540 | -7.417549019 | 9.0509E-06 | 2.6622E-05 |
| AT1G13550 | -4.65174909 | 0.00060472 | 0.00141077 |
| AT1G13590 | -1.824187623 | 8.6735E-05 | 0.00022752 |
| AT1G13608 | 5.780435418 | 0.00481111 | 0.00973485 |
| AT1G13609 | 3.487264837 | 0.00211571 | 0.00455569 |
| AT1G13670 | -2.785973818 | 5.3749E-18 | 3.624E-17 |
| AT1G13740 | 1.068558792 | 3.5283E-09 | 1.4158E-08 |
| AT1G13790 | -1.577366956 | 1.2743E-09 | 5.3084E-09 |
| AT1G13800 | -1.509494478 | 0.00025666 | 0.00063295 |
| AT1G13830 | -2.625740602 | 1.6328E-13 | 8.6841E-13 |
| AT1G13920 | 1.358577816 | 3.6067E-16 | 2.2139E-15 |
| AT1G13960 | 1.347989652 | 7.8205E-30 | 8.3185E-29 |
| AT1G13990 | 2.781982727 | 4.001E-235 | 5.434E-233 |
| AT1G14040 | -1.241332659 | 4.9786E-09 | 1.9752E-08 |
| AT1G14060 | -1.046422541 | 6.0205E-13 | 3.0945E-12 |
| AT1G14080 | -2.660049869 | 1.3148E-14 | 7.4532E-14 |
| AT1G14120 | -1.588257133 | 3.879E-23 | 3.2586E-22 |
| AT1G14130 | -1.22889308 | 5.6258E-12 | 2.7177E-11 |
| AT1G14160 | -3.611300902 | 1.0733E-08 | 4.1472E-08 |
| AT1G14180 | -1.551403843 | 1.4205E-09 | 5.8972E-09 |
| AT1G14185 | -3.140130458 | 7.886E-07 | 2.5763E-06 |
| AT1G14190 | -1.984880138 | 0.00011877 | 0.00030717 |
| AT1G14205 | -1.248986705 | 0.00156722 | 0.00344221 |
| AT1G14210 | -2.13711459 | 1.2764E-45 | 2.1057E-44 |
| AT1G14240 | -1.531843937 | 9.5896E-09 | 3.7248E-08 |
| AT1G14280 | -2.711231078 | 2.746E-15 | 1.6131E-14 |
| AT1G14345 | -1.389681209 | 1.1014E-44 | 1.7784E-43 |
| AT1G14410 | -1.458078918 | 1.0679E-30 | 1.1636E-29 |
| AT1G14430 | -1.84869078 | 2.5571E-07 | 8.7694E-07 |
| AT1G14440 | -1.322789846 | 5.4594E-12 | 2.6401E-11 |
| AT1G14520 | 1.176458049 | 3.7405E-12 | 1.8239E-11 |
| AT1G14530 | 2.085106715 | 2.0754E-35 | 2.6061E-34 |
| AT1G14540 | -4.111325778 | 5.2123E-67 | 1.3453E-65 |
| AT1G14550 | -7.761847642 | 3.413E-13 | 1.7763E-12 |
| AT1G14580 | -1.263468375 | 2.8691E-17 | 1.8623E-16 |
| AT1G14600 | -1.194430944 | 0.00183412 | 0.00397901 |
| AT1G14630 | -1.255498123 | 0.00014243 | 0.00036468 |
| AT1G14730 | 2.264372216 | 1.115E-84 | 3.8141E-83 |
| AT1G14810 | -1.229883215 | 9.9716E-51 | 1.8618E-49 |
| AT1G14920 | -1.005049603 | 1.8775E-17 | 1.2302E-16 |
| AT1G14960 | -2.387045578 | 1.0036E-24 | 8.9412E-24 |
| AT1G14980 | -1.156819698 | 1.3894E-47 | 2.3979E-46 |
| AT1G15040 | -2.418724437 | 7.3985E-09 | 2.8984E-08 |
| AT1G15060 | 1.310605785 | 4.4713E-29 | 4.6754E-28 |
| AT1G15085 | -2.967369365 | 0.00090775 | 0.00206535 |
| AT1G15125 | -4.432780538 | 5.9917E-10 | 2.561E-09 |
| AT1G15190 | 1.985064538 | 0.0043804 | 0.0089235 |
| AT1G15210 | -1.22836628 | 3.6147E-24 | 3.1598E-23 |
| AT1G15230 | 1.459051813 | 7.1116E-40 | 1.0135E-38 |
| AT1G15250 | -1.650258663 | 1.8193E-24 | 1.607E-23 |
| AT1G15290 | -1.696791973 | 2.2273E-70 | 6.0211E-69 |
| AT1G15310 | 1.777742523 | 1.7897E-35 | 2.256E-34 |
| AT1G15330 | 6.588341645 | 1.1817E-16 | 7.4236E-16 |
| AT1G15350 | 1.85821382 | 6.1752E-53 | 1.2115E-51 |
| AT1G15390 | -1.205376447 | 1.1949E-19 | 8.7077E-19 |
| AT1G15400 | 1.313393891 | 4.3058E-37 | 5.6985E-36 |
| AT1G15430 | 1.736358363 | 9.897E-42 | 1.4817E-40 |
| AT1G15440 | -1.000771661 | 3.7504E-29 | 3.9269E-28 |
| AT1G15510 | -2.134676696 | 8.9645E-38 | 1.216E-36 |
| AT1G15640 | -6.153537507 | 0.00162397 | 0.00355658 |
| AT1G15740 | 2.038156612 | 6.1295E-90 | 2.3116E-88 |
| AT1G15760 | -1.570104218 | 6.6316E-06 | 1.9768E-05 |
| AT1G15800 | 1.528559862 | 2.0589E-16 | 1.2783E-15 |
| AT1G15870 | -1.225870769 | 0.00065372 | 0.00151717 |
| AT1G15880 | 1.344872973 | 1.0548E-27 | 1.0519E-26 |
| AT1G15920 | 1.008076945 | 1.2676E-13 | 6.7861E-13 |
| AT1G15960 | 2.346047984 | 1.7887E-60 | 4.0689E-59 |
| AT1G16030 | 3.785068149 | 1.178E-163 | 9.74E-162 |
| AT1G16060 | -1.619131188 | 7.1472E-05 | 0.00018975 |
| AT1G16070 | -1.109004484 | 0.00062723 | 0.00146013 |
| AT1G16090 | 1.287964713 | 4.119E-07 | 1.3841E-06 |
| AT1G16240 | 1.179515499 | 2.8648E-34 | 3.4511E-33 |
| AT1G16280 | -1.311829722 | 5.7479E-18 | 3.8685E-17 |
| AT1G16300 | 1.277537316 | 9.2738E-29 | 9.6134E-28 |
| AT1G16350 | -1.506369579 | 1.1442E-74 | 3.3173E-73 |
| AT1G16370 | -2.918234519 | 6.1234E-09 | 2.4121E-08 |
| AT1G16390 | -5.493587636 | 2.9765E-35 | 3.713E-34 |
| AT1G16400 | -4.3392843 | 6.7533E-93 | 2.6339E-91 |
| AT1G16410 | -4.594373124 | 0 | 0 |
| AT1G16420 | -2.732825633 | 0.00344303 | 0.00713952 |
| AT1G16440 | -2.802575302 | 8.7401E-05 | 0.00022918 |
| AT1G16445 | -1.209585721 | 3.9992E-05 | 0.00010966 |
| AT1G16510 | 1.5845428 | 3.1781E-05 | 8.8053E-05 |
| AT1G16515 | 3.506928286 | 1.4327E-53 | 2.835E-52 |
| AT1G16630 | -1.183403924 | 2.7082E-10 | 1.1837E-09 |
| AT1G16640 | -1.515361195 | 0.00092882 | 0.00210865 |
| AT1G16730 | 1.141288911 | 0.00080335 | 0.00184361 |
| AT1G16820 | 1.374090497 | 0.00870762 | 0.0167765 |
| AT1G16830 | -1.750656067 | 1.2801E-07 | 4.5185E-07 |
| AT1G16840 | 1.809573794 | 3.3097E-81 | 1.0606E-79 |
| AT1G16850 | 5.007798889 | 0 | 0 |
| AT1G16860 | 1.102364725 | 5.5942E-34 | 6.6931E-33 |
| AT1G16905 | -3.88853593 | 0.00034165 | 0.00082786 |
| AT1G17020 | 1.120630571 | 2.0656E-07 | 7.1516E-07 |
| AT1G17060 | 1.608989545 | 0.00030488 | 0.00074356 |
| AT1G17180 | 1.739001858 | 2.152E-67 | 5.5731E-66 |
| AT1G17260 | 1.086918728 | 0.00242742 | 0.00516732 |
| AT1G17310 | 2.890893225 | 0.00758712 | 0.014793 |
| AT1G17340 | 1.223336549 | 4.7177E-23 | 3.9501E-22 |
| AT1G17380 | 1.776341664 | 1.3692E-10 | 6.08E-10 |
| AT1G17550 | 1.951002511 | 6.787E-144 | 4.623E-142 |
| AT1G17560 | -1.414228719 | 5.008E-09 | 1.9862E-08 |
| AT1G17744 | 5.645394782 | 0.00754967 | 0.01472251 |
| AT1G17745 | 1.984405132 | 1.223E-121 | 6.628E-120 |
| AT1G17780 | 1.205392667 | 1.0422E-10 | 4.6633E-10 |
| AT1G17830 | 1.428955179 | 2.1858E-12 | 1.0839E-11 |
| AT1G17840 | 1.136461309 | 1.0543E-41 | 1.5763E-40 |
| AT1G17850 | -1.496679386 | 1.6497E-19 | 1.1949E-18 |
| AT1G17870 | 4.393397925 | 7.526E-225 | 9.435E-223 |
| AT1G17940 | 4.345159437 | 0 | 0 |
| AT1G17960 | 1.404786901 | 0.00051336 | 0.00121088 |
| AT1G18090 | -1.254239857 | 7.2708E-07 | 2.3831E-06 |
| AT1G18100 | 2.416511012 | 1.6058E-22 | 1.3141E-21 |
| AT1G05383 | 4.107564629 | 0.00908645 | 0.01743913 |
| AT1G18140 | -2.518214353 | 2.6393E-17 | 1.7171E-16 |
| AT1G18200 | -1.153409248 | 0.00813177 | 0.01576467 |
| AT1G18250 | -2.182764502 | 1.3991E-48 | 2.4785E-47 |
| AT1G18320 | -1.595756761 | 0.00039026 | 0.00093807 |
| AT1G18360 | 1.41364721 | 2.4751E-32 | 2.8406E-31 |
| AT1G18400 | -1.426459343 | 3.7819E-06 | 1.155E-05 |
| AT1G18460 | 1.578576733 | 3.8991E-44 | 6.1994E-43 |
| AT1G18590 | -2.947557452 | 1.17E-105 | 5.307E-104 |
| AT1G18650 | -1.418413736 | 7.2219E-19 | 5.0856E-18 |
| AT1G18710 | 1.950847007 | 2.0159E-27 | 1.9817E-26 |
| AT1G18745 | -1.688975779 | 0.00974264 | 0.01859529 |
| AT1G18810 | 1.396838612 | 1.4728E-11 | 6.943E-11 |
| AT1G18830 | 3.487837156 | 1.2909E-20 | 9.717E-20 |
| AT1G18850 | -1.029327533 | 2.7489E-13 | 1.4398E-12 |
| AT1G18860 | -1.53994107 | 0.00062203 | 0.00144848 |
| AT1G18870 | 3.049068094 | 1.044E-243 | 1.547E-241 |
| AT1G18880 | -1.435012642 | 1.1827E-21 | 9.3015E-21 |
| AT1G18890 | 1.299571708 | 6.1731E-24 | 5.3554E-23 |
| AT1G18970 | -2.761116886 | 2.4862E-30 | 2.6758E-29 |
| AT1G19050 | -1.982702401 | 8.4017E-22 | 6.6491E-21 |
| AT1G19180 | 1.987912407 | 7.845E-87 | 2.8055E-85 |
| AT1G19190 | -1.527551081 | 3.2505E-05 | 8.9971E-05 |
| AT1G19200 | 3.237064722 | 2.5976E-37 | 3.47E-36 |
| AT1G19210 | 2.729654621 | 0.00275762 | 0.0058155 |
| AT1G19310 | 1.067561921 | 2.8961E-13 | 1.5141E-12 |
| AT1G19340 | -1.180185281 | 0.00187624 | 0.00406497 |
| AT1G19380 | -1.858709646 | 2.2276E-06 | 6.9546E-06 |
| AT1G19394 | 2.895874862 | 8.9828E-07 | 2.9187E-06 |
| AT1G19396 | 2.240131172 | 4.1926E-07 | 1.4082E-06 |
| AT1G19397 | 2.086110744 | 1.5283E-10 | 6.7631E-10 |
| AT1G19400 | 1.289117229 | 6.4388E-32 | 7.2799E-31 |
| AT1G19490 | 2.43437577 | 8.7083E-33 | 1.0142E-31 |
| AT1G19520 | -1.076741322 | 4.7456E-26 | 4.4249E-25 |
| AT1G19630 | 2.056852394 | 6.531E-11 | 2.9542E-10 |
| AT1G19640 | 1.705746995 | 0.00049425 | 0.00116834 |
| AT1G19660 | 2.233152467 | 6.208E-272 | 1.081E-269 |
| AT1G19670 | -1.735552034 | 1.0413E-71 | 2.8798E-70 |
| AT1G19830 | -4.494714042 | 0.00247049 | 0.00525221 |
| AT1G19900 | -6.317578197 | 4.8247E-08 | 1.7652E-07 |
| AT1G19920 | -1.156665926 | 2.9008E-43 | 4.5178E-42 |
| AT1G19960 | -2.65066642 | 1.0936E-15 | 6.5472E-15 |
| AT1G19970 | 2.787375801 | 5.1818E-90 | 1.9574E-88 |
| AT1G19980 | 1.243046025 | 2.7748E-08 | 1.0375E-07 |
| AT1G20010 | -1.371769709 | 3.7727E-64 | 9.3058E-63 |
| AT1G20030 | 1.975424925 | 1.49E-54 | 3.0166E-53 |
| AT1G20100 | 1.263055318 | 4.3749E-62 | 1.0355E-60 |
| AT1G20140 | 1.89720301 | 6.7555E-65 | 1.6882E-63 |
| AT1G20160 | 1.15571391 | 1.1821E-13 | 6.3463E-13 |
| AT1G20190 | -1.468542518 | 2.904E-13 | 1.5179E-12 |
| AT1G20300 | -1.15835073 | 3.0344E-11 | 1.4015E-10 |
| AT1G20330 | -1.226749822 | 3.6126E-50 | 6.6579E-49 |
| AT1G20440 | 3.710650309 | 0 | 0 |
| AT1G20450 | 4.232040083 | 0 | 0 |
| AT1G20460 | 1.275443996 | 1.406E-07 | 4.9498E-07 |
| AT1G20490 | 1.35416414 | 3.1224E-12 | 1.5324E-11 |
| AT1G20550 | 1.060753875 | 2.4887E-07 | 8.545E-07 |
| AT1G20670 | 1.1264712 | 6.1174E-19 | 4.3185E-18 |
| AT1G20720 | -1.05563383 | 1.0406E-05 | 3.0439E-05 |
| AT1G20840 | -1.597240573 | 1.0221E-45 | 1.6912E-44 |
| AT1G20870 | 1.613140821 | 7.7447E-09 | 3.0304E-08 |
| AT1G20880 | 1.987972764 | 6.2131E-40 | 8.8765E-39 |
| AT1G20950 | -1.047089917 | 5.622E-39 | 7.8072E-38 |
| AT1G21000 | 2.980315814 | 6.774E-225 | 8.538E-223 |
| AT1G21050 | -1.892650014 | 1.4958E-12 | 7.4927E-12 |
| AT1G21100 | -1.79288146 | 1.2698E-36 | 1.6574E-35 |
| AT1G21110 | -2.37507567 | 2.228E-65 | 5.5983E-64 |
| AT1G21120 | -2.824133066 | 1.7512E-44 | 2.8198E-43 |
| AT1G21140 | -1.427648107 | 7.0786E-05 | 0.00018802 |
| AT1G21230 | -5.727367411 | 0.00661264 | 0.01303898 |
| AT1G21270 | -3.993796341 | 5.4681E-57 | 1.1704E-55 |
| AT1G21310 | -1.471383446 | 3.746E-166 | 3.154E-164 |
| AT1G21400 | 3.852968927 | 0 | 0 |
| AT1G21410 | 2.922788142 | 6.719E-146 | 4.66E-144 |
| AT1G21460 | 2.665044238 | 5.716E-123 | 3.15E-121 |
| AT1G21480 | 1.266774093 | 2.5672E-17 | 1.6716E-16 |
| AT1G21529 | 6.822610183 | 0.00012216 | 0.0003155 |
| AT1G21530 | -5.945361875 | 0.00341985 | 0.00709404 |
| AT1G21550 | 1.829242296 | 9.8967E-13 | 5.029E-12 |
| AT1G21600 | -1.174071984 | 6.3297E-36 | 8.1133E-35 |
| AT1G21660 | 1.097167233 | 7.7842E-35 | 9.5384E-34 |
| AT1G21740 | -1.248986071 | 2.7725E-09 | 1.1226E-08 |
| AT1G21780 | 1.408025997 | 4.5329E-38 | 6.1819E-37 |
| AT1G21790 | 3.863789254 | 5.917E-274 | 1.055E-271 |
| AT1G21980 | 1.417679405 | 2.6865E-51 | 5.1207E-50 |
| AT1G22030 | -1.452343969 | 3.0189E-07 | 1.0281E-06 |
| AT1G22160 | 3.031407846 | 6.982E-105 | 3.13E-103 |
| AT1G22180 | 1.161101951 | 2.0552E-11 | 9.5871E-11 |
| AT1G22190 | 1.310630197 | 6.1274E-28 | 6.156E-27 |
| AT1G22220 | -1.046645271 | 0.00632171 | 0.01250964 |
| AT1G22230 | -1.025436666 | 0.00017183 | 0.0004342 |
| AT1G22280 | 1.080813316 | 9.495E-24 | 8.1689E-23 |
| AT1G22330 | -2.115888204 | 1.576E-12 | 7.8785E-12 |
| AT1G22370 | 2.987915019 | 1.032E-159 | 8.206E-158 |
| AT1G22380 | 1.625060125 | 3.6064E-05 | 9.9364E-05 |
| AT1G22470 | 3.989454126 | 2.5717E-73 | 7.2888E-72 |
| AT1G22490 | 1.733306464 | 5.7303E-05 | 0.00015411 |
| AT1G22500 | -1.333497799 | 0.00067764 | 0.00156933 |
| AT1G22530 | -2.019428175 | 4.1624E-84 | 1.4007E-82 |
| AT1G22550 | -2.376733949 | 9.2608E-38 | 1.2547E-36 |
| AT1G22600 | 2.158133828 | 0.00035726 | 0.00086338 |
| AT1G22630 | -1.500884654 | 3.5735E-26 | 3.3484E-25 |
| AT1G22640 | 2.183175044 | 1.4467E-61 | 3.3994E-60 |
| AT1G22650 | -1.2534157 | 5.9878E-07 | 1.9768E-06 |
| AT1G22690 | -3.517331454 | 8.1311E-95 | 3.2777E-93 |
| AT1G22710 | 1.668940946 | 8.532E-158 | 6.713E-156 |
| AT1G22750 | 1.017983818 | 9.6224E-18 | 6.4045E-17 |
| AT1G22890 | 1.496619818 | 1.9952E-13 | 1.0552E-12 |
| AT1G22900 | -1.089026976 | 0.00113021 | 0.0025331 |
| AT1G22930 | 1.953833147 | 1.995E-213 | 2.346E-211 |
| AT1G22985 | 3.075161477 | 4.4665E-55 | 9.1398E-54 |
| AT1G22990 | 3.937877791 | 3.9051E-30 | 4.1771E-29 |
| AT1G23030 | -1.214704549 | 2.2292E-16 | 1.3825E-15 |
| AT1G23040 | 1.464533409 | 2.2639E-30 | 2.44E-29 |
| AT1G23070 | 8.83614943 | 1.1086E-08 | 4.2806E-08 |
| AT1G23080 | -1.300330316 | 2.2641E-50 | 4.1896E-49 |
| AT1G23100 | -1.393969836 | 2.9344E-10 | 1.2802E-09 |
| AT1G23190 | 1.280927401 | 8.2229E-77 | 2.4654E-75 |
| AT1G23200 | 2.680971687 | 6.4628E-23 | 5.362E-22 |
| AT1G23205 | -2.009821167 | 1.1281E-31 | 1.2717E-30 |
| AT1G23280 | -1.201991445 | 1.8411E-31 | 2.0552E-30 |
| AT1G23330 | 1.048566261 | 6.2655E-22 | 4.9984E-21 |
| AT1G23340 | -1.365762627 | 7.4089E-06 | 2.1962E-05 |
| AT1G23440 | 1.145387527 | 1.3543E-45 | 2.231E-44 |
| AT1G23480 | -2.191223207 | 3.7209E-28 | 3.7648E-27 |
| AT1G23710 | 1.305903337 | 2.1598E-13 | 1.1391E-12 |
| AT1G23720 | -3.640788007 | 3.019E-224 | 3.744E-222 |
| AT1G23800 | 1.699450183 | 1.1586E-33 | 1.376E-32 |
| AT1G23850 | -2.007371329 | 0.00033794 | 0.00081913 |
| AT1G23960 | 1.048553719 | 1.2638E-22 | 1.0391E-21 |
| AT1G24020 | -1.875872501 | 2.167E-138 | 1.377E-136 |
| AT1G24070 | 2.757450622 | 6.333E-235 | 8.551E-233 |
| AT1G24090 | -1.341762749 | 9.3007E-07 | 3.0181E-06 |
| AT1G24100 | -1.654105727 | 8.4071E-74 | 2.4007E-72 |
| AT1G24147 | -2.748979908 | 0.0017788 | 0.00386885 |
| AT1G24280 | -1.347338869 | 4.8312E-24 | 4.2024E-23 |
| AT1G24330 | 1.277383876 | 1.1594E-08 | 4.4652E-08 |
| AT1G24440 | 1.141326259 | 9.6167E-13 | 4.8911E-12 |
| AT1G24470 | 3.7680105 | 4.1806E-32 | 4.7621E-31 |
| AT1G24570 | 3.408938918 | 6.896E-07 | 2.2642E-06 |
| AT1G24580 | 5.772982166 | 2.837E-152 | 2.129E-150 |
| AT1G24575 | 1.218561088 | 1.5856E-11 | 7.4544E-11 |
| AT1G24600 | 5.612929768 | 2.6211E-96 | 1.0698E-94 |
| AT1G24735 | 1.000834067 | 0.00197927 | 0.004274 |
| AT1G25230 | -1.048745442 | 4.5526E-08 | 1.6694E-07 |
| AT1G25277 | 1.158048409 | 0.00283988 | 0.0059713 |
| AT1G25360 | -1.291974742 | 7.553E-07 | 2.471E-06 |
| AT1G25370 | 1.778683175 | 9.3307E-28 | 9.3209E-27 |
| AT1G25422 | 1.100241201 | 0.00383866 | 0.00789106 |
| AT1G25440 | -1.300166657 | 3.3234E-57 | 7.1671E-56 |
| AT1G25450 | 1.464658072 | 7.4629E-45 | 1.2059E-43 |
| AT1G25510 | -1.140519078 | 2.1243E-10 | 9.3317E-10 |
| AT1G25520 | 1.575396768 | 6.3523E-36 | 8.1378E-35 |
| AT1G25560 | 1.269020025 | 4.9482E-35 | 6.1258E-34 |
| AT1G26100 | -1.125521335 | 0.0009893 | 0.0022364 |
| AT1G26208 | 1.486780077 | 0.00135887 | 0.00300776 |
| AT1G26240 | -11.27218515 | 1.2291E-14 | 6.9759E-14 |
| AT1G26250 | -3.719243829 | 1.6832E-05 | 4.8162E-05 |
| AT1G26380 | -4.329817223 | 8.104E-108 | 3.797E-106 |
| AT1G26390 | -3.303997953 | 0.00136507 | 0.00302033 |
| AT1G26410 | -4.665106605 | 3.261E-14 | 1.8107E-13 |
| AT1G26420 | -1.530836283 | 3.8358E-07 | 1.292E-06 |
| AT1G26450 | 1.579172042 | 4.6603E-39 | 6.4795E-38 |
| AT1G26540 | -2.607729222 | 2.4504E-10 | 1.0735E-09 |
| AT1G26620 | 1.441154393 | 2.6674E-25 | 2.416E-24 |
| AT1G26665 | 1.112298898 | 3.5803E-21 | 2.7542E-20 |
| AT1G26670 | 1.969413304 | 4.152E-108 | 1.953E-106 |
| AT1G26730 | 1.521088742 | 1.9811E-10 | 8.7229E-10 |
| AT1G26800 | 2.101675303 | 4.5453E-20 | 3.3551E-19 |
| AT1G26920 | 1.406076931 | 1.7429E-26 | 1.6542E-25 |
| AT1G26945 | -1.583184569 | 3.8595E-05 | 0.00010603 |
| AT1G27020 | -2.866300047 | 1.401E-72 | 3.9173E-71 |
| AT1G27050 | -1.011325106 | 5.4924E-09 | 2.1703E-08 |
| AT1G27140 | -1.739648916 | 4.7001E-05 | 0.00012788 |
| AT1G27150 | 1.498884994 | 6.0611E-48 | 1.054E-46 |
| AT1G27200 | 2.549859313 | 2.078E-118 | 1.092E-116 |
| AT1G27290 | 1.585883476 | 6.1055E-51 | 1.1484E-49 |
| AT1G27300 | 1.1505482 | 1.2411E-16 | 7.7856E-16 |
| AT1G27460 | -1.178805247 | 2.5742E-16 | 1.5886E-15 |
| AT1G27461 | 7.145506502 | 9.4808E-11 | 4.254E-10 |
| AT1G27620 | -1.751872583 | 0.00013314 | 0.00034268 |
| AT1G27730 | 2.735263259 | 1.7019E-95 | 6.897E-94 |
| AT1G27740 | -6.251901313 | 5.8061E-08 | 2.1124E-07 |
| AT1G27880 | -1.165318149 | 1.4214E-06 | 4.5211E-06 |
| AT1G27990 | 6.675818219 | 0.00024793 | 0.00061286 |
| AT1G28070 | 1.15466825 | 3.7785E-05 | 0.00010389 |
| AT1G28110 | -1.250495362 | 7.3324E-10 | 3.1142E-09 |
| AT1G28130 | -1.77697674 | 1.6871E-26 | 1.6026E-25 |
| AT1G28200 | 1.17641224 | 5.299E-36 | 6.8076E-35 |
| AT1G28260 | 2.052822969 | 7.1865E-45 | 1.1621E-43 |
| AT1G28330 | 2.910807724 | 5.448E-254 | 8.572E-252 |
| AT1G28350 | -1.142468091 | 1.2577E-29 | 1.3334E-28 |
| AT1G28370 | 2.196974637 | 7.0808E-09 | 2.7768E-08 |
| AT1G28390 | -1.032184903 | 1.2092E-05 | 3.5122E-05 |
| AT1G28395 | -1.294405379 | 2.5467E-18 | 1.7496E-17 |
| AT1G28400 | -1.93315162 | 3.716E-114 | 1.855E-112 |
| AT1G28440 | -1.093653477 | 3.7406E-36 | 4.8299E-35 |
| AT1G06163 | 6.289785829 | 0.00095261 | 0.00215923 |
| AT1G28520 | 1.444537656 | 6.5234E-57 | 1.3936E-55 |
| AT1G28530 | -1.335008523 | 2.7023E-12 | 1.3291E-11 |
| AT1G28660 | -3.035599041 | 1.9663E-42 | 3.0129E-41 |
| AT1G28670 | -2.31664672 | 5.5842E-55 | 1.1406E-53 |
| AT1G28710 | -1.357121996 | 1.9016E-15 | 1.1272E-14 |
| AT1G28765 | 2.111234039 | 0.00019253 | 0.00048293 |
| AT1G09045 | 2.06029584 | 0.00516606 | 0.01039408 |
| AT1G28960 | 2.399991412 | 5.429E-126 | 3.073E-124 |
| AT1G29025 | -2.690593565 | 3.3693E-13 | 1.7543E-12 |
| AT1G29050 | 1.741334346 | 4.6579E-49 | 8.3549E-48 |
| AT1G29195 | 2.108424515 | 1.872E-17 | 1.227E-16 |
| AT1G29230 | 1.450021734 | 0.00151194 | 0.0033272 |
| AT1G29240 | 1.678688159 | 2.5743E-18 | 1.7676E-17 |
| AT1G29250 | -1.083899497 | 3.5886E-27 | 3.496E-26 |
| AT1G29280 | -1.941795844 | 7.2317E-38 | 9.833E-37 |
| AT1G29320 | -1.464663178 | 1.1915E-34 | 1.4514E-33 |
| AT1G29330 | 1.955484508 | 1.5968E-59 | 3.5823E-58 |
| AT1G29357 | 1.799564524 | 1.4898E-08 | 5.6919E-08 |
| AT1G29395 | 2.465350055 | 0 | 0 |
| AT1G29400 | 1.107381622 | 1.3435E-53 | 2.6631E-52 |
| AT1G29430 | -8.386657962 | 8.1447E-08 | 2.9269E-07 |
| AT1G29440 | -4.979611765 | 0.00632768 | 0.01252037 |
| AT1G29460 | -6.548451239 | 0.00040765 | 0.00097648 |
| AT1G06283 | -1.670576795 | 0.00237644 | 0.00506927 |
| AT1G29500 | 1.600813714 | 4.9786E-06 | 1.5037E-05 |
| AT1G29530 | -1.116919047 | 1.6803E-12 | 8.3816E-12 |
| AT1G29600 | -1.886436537 | 2.468E-05 | 6.9329E-05 |
| AT1G29640 | 2.802266453 | 1.3393E-68 | 3.5409E-67 |
| AT1G29660 | -5.030706748 | 0 | 0 |
| AT1G29760 | 1.283073532 | 1.2836E-20 | 9.6652E-20 |
| AT1G29785 | -1.236933155 | 4.9537E-07 | 1.6509E-06 |
| AT1G29800 | 1.219163454 | 2.8025E-13 | 1.4666E-12 |
| AT1G29840 | -1.084126999 | 0.00014111 | 0.0003616 |
| AT1G29900 | -1.22336014 | 8.8172E-70 | 2.3612E-68 |
| AT1G29970 | 1.244485847 | 9.7643E-21 | 7.394E-20 |
| AT1G29980 | -1.584659601 | 4.341E-34 | 5.2101E-33 |
| AT1G30080 | -1.208544405 | 1.4216E-05 | 4.1006E-05 |
| AT1G30190 | 9.611885238 | 1.3848E-10 | 6.1458E-10 |
| AT1G30220 | 3.713476336 | 2.7656E-22 | 2.2376E-21 |
| AT1G30240 | -1.251705278 | 2.0049E-17 | 1.3133E-16 |
| AT1G30250 | 5.462370164 | 4.3943E-41 | 6.4685E-40 |
| AT1G30260 | 2.306389704 | 3.0447E-35 | 3.7919E-34 |
| AT1G30360 | 1.009502903 | 1.7623E-66 | 4.5229E-65 |
| AT1G30370 | -2.723497678 | 6.2636E-05 | 0.00016762 |
| AT1G30475 | 1.256698593 | 0.00419416 | 0.00857243 |
| AT1G30500 | 2.446045825 | 3.3649E-51 | 6.3872E-50 |
| AT1G30530 | -2.599636476 | 4.458E-181 | 4.203E-179 |
| AT1G30550 | -1.256854086 | 0.00435033 | 0.00886857 |
| AT1G30610 | -1.234995874 | 1.2811E-20 | 9.6499E-20 |
| AT1G30620 | 1.33714507 | 2.6446E-32 | 3.0261E-31 |
| AT1G30640 | 1.766185466 | 6.2016E-41 | 9.0994E-40 |
| AT1G30650 | -1.124403394 | 1.493E-06 | 4.735E-06 |
| AT1G30720 | -2.198344481 | 7.249E-26 | 6.7287E-25 |
| AT1G30730 | -1.713212643 | 3.7364E-12 | 1.8223E-11 |
| AT1G30750 | -3.197809918 | 5.0015E-19 | 3.5406E-18 |
| AT1G30760 | -1.873264727 | 6.9264E-07 | 2.2735E-06 |
| AT1G30840 | -5.000934169 | 1.8175E-45 | 2.9769E-44 |
| AT1G30850 | -3.478628638 | 0.0031262 | 0.0065348 |
| AT1G30860 | 1.112399052 | 1.1074E-05 | 3.2297E-05 |
| AT1G30870 | -5.180717796 | 5.1318E-69 | 1.3615E-67 |
| AT1G30900 | -1.260578703 | 6.2218E-08 | 2.2601E-07 |
| AT1G30960 | -1.508885743 | 6.2459E-14 | 3.4166E-13 |
| AT1G30990 | -2.397428588 | 0.00125989 | 0.00280473 |
| AT1G31010 | -1.053289551 | 4.809E-05 | 0.00013065 |
| AT1G31040 | 1.206262405 | 0.00172235 | 0.00375547 |
| AT1G31050 | -1.278834084 | 8.8336E-07 | 2.8731E-06 |
| AT1G31170 | 1.334481288 | 1.6168E-28 | 1.6579E-27 |
| AT1G31180 | -1.056176057 | 5.2438E-35 | 6.4672E-34 |
| AT1G31320 | -1.982327182 | 1.6469E-13 | 8.7549E-13 |
| AT1G31480 | 1.055452839 | 3.1963E-12 | 1.5666E-11 |
| AT1G31580 | -1.270243791 | 7.2852E-84 | 2.4372E-82 |
| AT1G31660 | -1.099817322 | 2.0622E-29 | 2.1702E-28 |
| AT1G31690 | -3.407927233 | 7.394E-150 | 5.339E-148 |
| AT1G31710 | -1.05218278 | 3.0433E-21 | 2.3538E-20 |
| AT1G31750 | 3.660710434 | 1.2407E-28 | 1.2792E-27 |
| AT1G31770 | -2.333535756 | 2.6274E-55 | 5.4006E-54 |
| AT1G31820 | 1.93751391 | 3.9462E-16 | 2.4191E-15 |
| AT1G31830 | 1.145358507 | 2.2111E-20 | 1.6497E-19 |
| AT1G31880 | 1.188564538 | 2.5161E-06 | 7.8095E-06 |
| AT1G31885 | -2.369711136 | 6.9969E-07 | 2.296E-06 |
| AT1G06473 | 3.814034535 | 7.1497E-23 | 5.9211E-22 |
| AT1G32350 | -1.263048888 | 0.00600461 | 0.01192975 |
| AT1G06547 | -2.883759314 | 0.00287104 | 0.00603071 |
| AT1G06553 | -2.153055258 | 0.00075562 | 0.00174126 |
| AT1G32400 | 1.100343516 | 2.9866E-47 | 5.1197E-46 |
| AT1G32410 | 1.036856036 | 2.916E-15 | 1.7108E-14 |
| AT1G32415 | -1.256812584 | 0.00021186 | 0.00052816 |
| AT1G32450 | 1.378310777 | 1.8044E-64 | 4.47E-63 |
| AT1G32540 | -1.372416658 | 6.8552E-10 | 2.9175E-09 |
| AT1G32560 | 4.371025244 | 3.8214E-22 | 3.07E-21 |
| AT1G32740 | 1.3455712 | 5.7654E-08 | 2.098E-07 |
| AT1G32780 | -2.082919466 | 6.7188E-06 | 2.0015E-05 |
| AT1G32870 | 1.668597602 | 1.6129E-37 | 2.1737E-36 |
| AT1G32960 | -5.013875371 | 1.6496E-07 | 5.7601E-07 |
| AT1G33030 | -2.091892375 | 0.00068014 | 0.00157495 |
| AT1G33055 | 3.852839976 | 1.0372E-06 | 3.3476E-06 |
| AT1G33110 | 1.177808174 | 1.3179E-50 | 2.4566E-49 |
| AT1G33170 | -1.941534972 | 1.488E-30 | 1.6128E-29 |
| AT1G33240 | -1.647501505 | 9.3339E-34 | 1.1132E-32 |
| AT1G33260 | 1.04159069 | 3.7765E-07 | 1.2733E-06 |
| AT1G33270 | 1.103193087 | 2.1218E-20 | 1.5841E-19 |
| AT1G33280 | -2.22877218 | 0.00260576 | 0.0055187 |
| AT1G33340 | -1.918850264 | 0.00028944 | 0.00070812 |
| AT1G33390 | -1.236569075 | 2.5462E-21 | 1.9739E-20 |
| AT1G33440 | -1.796243201 | 3.4012E-05 | 9.3938E-05 |
| AT1G33475 | 1.589116174 | 0.0006346 | 0.00147611 |
| AT1G33480 | 2.599530436 | 2.8825E-73 | 8.1396E-72 |
| AT1G33590 | -1.321324535 | 1.9008E-55 | 3.9284E-54 |
| AT1G33600 | -1.30068871 | 5.6639E-28 | 5.7004E-27 |
| AT1G33610 | -1.529550118 | 1.7289E-05 | 4.9383E-05 |
| AT1G33700 | 1.676988557 | 4.1446E-22 | 3.3274E-21 |
| AT1G33750 | -2.155768953 | 1.2014E-18 | 8.3622E-18 |
| AT1G33811 | -3.510079465 | 1.111E-106 | 5.1E-105 |
| AT1G33930 | -1.536038472 | 0.00284187 | 0.00597494 |
| AT1G34040 | -2.772932027 | 5.8984E-18 | 3.9675E-17 |
| AT1G34065 | -1.436343992 | 4.1738E-09 | 1.6698E-08 |
| AT1G34120 | 1.300795955 | 5.0307E-13 | 2.5945E-12 |
| AT1G34220 | 1.096767074 | 1.9287E-25 | 1.7567E-24 |
| AT1G34245 | -1.236088534 | 0.00015443 | 0.00039302 |
| AT1G34420 | -1.066306878 | 0.00071007 | 0.00164043 |
| AT1G34510 | -4.792694852 | 0.00030564 | 0.00074518 |
| AT1G34630 | 1.995244629 | 3.0741E-38 | 4.2025E-37 |
| AT1G35140 | -3.715111081 | 5.4705E-12 | 2.6444E-11 |
| AT1G35180 | 1.07476426 | 1.4114E-05 | 4.0732E-05 |
| AT1G35250 | -2.17052595 | 1.3675E-20 | 1.0273E-19 |
| AT1G35260 | -1.707758283 | 2.7514E-13 | 1.4408E-12 |
| AT1G35290 | -3.205335686 | 2.096E-08 | 7.9292E-08 |
| AT1G35310 | -2.482221511 | 2.3019E-06 | 7.1728E-06 |
| AT1G35516 | 1.124426545 | 4.9771E-27 | 4.8138E-26 |
| AT1G35560 | -2.42501656 | 1.0457E-18 | 7.3007E-18 |
| AT1G36060 | -1.192770852 | 2.9932E-06 | 9.2226E-06 |
| AT1G36280 | -1.163139385 | 7.7134E-17 | 4.89E-16 |
| AT1G36370 | 1.414843853 | 1.1184E-70 | 3.0414E-69 |
| AT1G36380 | 1.796138469 | 1.4748E-16 | 9.2035E-16 |
| AT1G36390 | -1.333699475 | 6.6503E-21 | 5.073E-20 |
| AT1G36622 | -1.571851156 | 0.00054092 | 0.00127089 |
| AT1G38131 | -2.130921605 | 2.5515E-09 | 1.0366E-08 |
| AT1G41880 | -1.012956268 | 1.9245E-55 | 3.9701E-54 |
| AT1G09475 | -4.909628483 | 1.0776E-10 | 4.8201E-10 |
| AT1G42990 | 1.425003597 | 1.4685E-49 | 2.6613E-48 |
| AT1G43160 | 3.928977675 | 2.0353E-37 | 2.73E-36 |
| AT1G43245 | 1.267836937 | 2.229E-07 | 7.6987E-07 |
| AT1G07167 | -3.847133145 | 0.00071225 | 0.00164496 |
| AT1G43770 | 1.229240451 | 8.8997E-07 | 2.8938E-06 |
| AT1G07203 | 2.641516295 | 0.00103713 | 0.00233688 |
| AT1G43890 | 1.379711703 | 1.6214E-66 | 4.1707E-65 |
| AT1G43910 | 1.378077886 | 9.9948E-15 | 5.701E-14 |
| AT1G44020 | -6.925997608 | 8.0516E-05 | 0.00021203 |
| AT1G44050 | -5.849119694 | 0.0046642 | 0.0094569 |
| AT1G44100 | -1.322689977 | 3.6962E-25 | 3.3306E-24 |
| AT1G44160 | -1.186223957 | 0.00557429 | 0.01113784 |
| AT1G44350 | 1.232376772 | 3.4029E-14 | 1.8872E-13 |
| AT1G44760 | 1.034266356 | 2.8627E-19 | 2.0475E-18 |
| AT1G44770 | 1.026531946 | 2.1547E-17 | 1.4082E-16 |
| AT1G44780 | -1.111772287 | 2.4349E-07 | 8.3667E-07 |
| AT1G44800 | 1.303369888 | 1.6489E-66 | 4.2366E-65 |
| AT1G44830 | -4.532358512 | 0.00100182 | 0.00226312 |
| AT1G44900 | -1.269469614 | 6.8958E-24 | 5.9665E-23 |
| AT1G44970 | -4.506072869 | 2.2252E-06 | 6.9481E-06 |
| AT1G45110 | -1.209360601 | 3.7907E-05 | 0.0001042 |
| AT1G45120 | 1.030670089 | 5.4037E-06 | 1.6278E-05 |
| AT1G45145 | 1.150099176 | 7.8032E-42 | 1.1721E-40 |
| AT1G45150 | 1.044439658 | 5.7258E-13 | 2.9443E-12 |
| AT1G45249 | 3.003761953 | 0 | 0 |
| AT1G45976 | 1.378336712 | 1.7357E-25 | 1.5854E-24 |
| AT1G46480 | -1.099481699 | 4.347E-05 | 0.00011877 |
| AT1G46554 | 3.185666795 | 1.4344E-84 | 4.8919E-83 |
| AT1G46768 | 2.962226997 | 6.3747E-39 | 8.8362E-38 |
| AT1G47128 | 1.340343129 | 1.801E-139 | 1.154E-137 |
| AT1G47270 | 1.636457298 | 2.0266E-42 | 3.1034E-41 |
| AT1G07373 | 2.217099802 | 0.00217809 | 0.00468248 |
| AT1G47480 | -2.209261342 | 2.2267E-24 | 1.9631E-23 |
| AT1G47500 | -1.001167561 | 2.9327E-28 | 2.9832E-27 |
| AT1G47510 | 3.898750765 | 2.1064E-69 | 5.6143E-68 |
| AT1G47560 | 1.074149556 | 4.7882E-05 | 0.00013012 |
| AT1G47565 | 1.806373121 | 4.9756E-15 | 2.8864E-14 |
| AT1G47670 | -1.175960053 | 1.7788E-17 | 1.1669E-16 |
| AT1G47840 | -1.083509823 | 9.1585E-07 | 2.9737E-06 |
| AT1G47890 | -2.485633417 | 0.000371 | 0.00089461 |
| AT1G47960 | 3.05432577 | 5.501E-126 | 3.107E-124 |
| AT1G47990 | 2.130078746 | 0.00138563 | 0.00306371 |
| AT1G48100 | 2.752783059 | 4.2901E-28 | 4.3311E-27 |
| AT1G48260 | -1.950085374 | 0.00015197 | 0.00038721 |
| AT1G48300 | 1.714217707 | 1.0531E-21 | 8.294E-21 |
| AT1G48370 | 1.496334615 | 7.6301E-30 | 8.1274E-29 |
| AT1G48405 | 3.308394135 | 3.3463E-05 | 9.2534E-05 |
| AT1G48460 | -1.657585601 | 1.826E-28 | 1.8683E-27 |
| AT1G48480 | -1.815436696 | 5.9912E-62 | 1.4151E-60 |
| AT1G48500 | -1.100338132 | 0.00407087 | 0.0083376 |
| AT1G48510 | -2.244829282 | 5.7282E-06 | 1.7193E-05 |
| AT1G48570 | -2.135306903 | 4.182E-57 | 9.0015E-56 |
| AT1G48610 | -1.255247708 | 2.851E-18 | 1.951E-17 |
| AT1G48630 | -1.295696374 | 9.5843E-68 | 2.4906E-66 |
| AT1G48670 | -3.797638805 | 0.00081357 | 0.00186594 |
| AT1G48690 | -4.335803105 | 1.0207E-06 | 3.2978E-06 |
| AT1G48770 | 1.004883057 | 1.1828E-05 | 3.4392E-05 |
| AT1G48800 | -2.995348834 | 0.00122586 | 0.00273538 |
| AT1G48840 | 1.384608788 | 4.2761E-27 | 4.1499E-26 |
| AT1G48930 | -4.294292837 | 2.2048E-28 | 2.2478E-27 |
| AT1G49030 | -6.895179635 | 9.371E-05 | 0.00024502 |
| AT1G49032 | 1.076439588 | 5.3852E-08 | 1.9637E-07 |
| AT1G49230 | -3.648775911 | 2.8192E-19 | 2.017E-18 |
| AT1G49240 | 1.004013411 | 1.9209E-66 | 4.919E-65 |
| AT1G49310 | -1.559332373 | 8.3641E-10 | 3.5386E-09 |
| AT1G49320 | -2.129233457 | 1.3308E-08 | 5.0998E-08 |
| AT1G49390 | -1.875627361 | 0.00712502 | 0.01396003 |
| AT1G49405 | 2.497573661 | 0.00053632 | 0.00126112 |
| AT1G49450 | 4.690131935 | 0 | 0 |
| AT1G49500 | 1.734184088 | 9.7575E-74 | 2.7828E-72 |
| AT1G49510 | -1.375650117 | 3.0216E-22 | 2.4387E-21 |
| AT1G49570 | -3.426824875 | 3.0109E-06 | 9.2732E-06 |
| AT1G49600 | -1.2092249 | 2.098E-59 | 4.6976E-58 |
| AT1G49650 | -1.219275555 | 2.4752E-19 | 1.777E-18 |
| AT1G49750 | -2.884544074 | 1.972E-243 | 2.903E-241 |
| AT1G49832 | 1.236438869 | 0.00849478 | 0.0164057 |
| AT1G49900 | 2.153234899 | 0.00405653 | 0.00831049 |
| AT1G49975 | -1.086585169 | 2.071E-11 | 9.6572E-11 |
| AT1G50020 | 1.310694703 | 2.6933E-34 | 3.2497E-33 |
| AT1G50040 | -2.737480758 | 1.8744E-07 | 6.5084E-07 |
| AT1G50055 | -1.214846582 | 8.1613E-06 | 2.4126E-05 |
| AT1G50060 | -2.662187655 | 5.3919E-15 | 3.124E-14 |
| AT1G50110 | -1.205084418 | 1.6585E-09 | 6.844E-09 |
| AT1G50260 | 2.792554377 | 4.512E-243 | 6.556E-241 |
| AT1G50290 | 1.040099106 | 0.00277158 | 0.00584098 |
| AT1G50560 | -1.101047811 | 0.0006694 | 0.00155104 |
| AT1G50575 | -1.152335942 | 9.6927E-13 | 4.9287E-12 |
| AT1G50580 | -1.524648576 | 0.0016638 | 0.00363753 |
| AT1G50630 | 1.420817209 | 1.3987E-22 | 1.1479E-21 |
| AT1G50740 | 1.38835575 | 1.0257E-14 | 5.8462E-14 |
| AT1G50840 | -1.038432084 | 1.547E-20 | 1.1595E-19 |
| AT1G50900 | -1.494732591 | 7.9512E-22 | 6.3013E-21 |
| AT1G50930 | 3.866370578 | 3.888E-14 | 2.1495E-13 |
| AT1G50940 | 1.086490427 | 9.596E-12 | 4.5727E-11 |
| AT1G51080 | -1.76405058 | 2.4816E-12 | 1.2242E-11 |
| AT1G51090 | 2.873337588 | 2.382E-172 | 2.131E-170 |
| AT1G51100 | -1.232079922 | 2.676E-16 | 1.6493E-15 |
| AT1G51130 | 1.148720588 | 1.0431E-08 | 4.0364E-08 |
| AT1G51140 | 2.896918299 | 8.12E-300 | 1.669E-297 |
| AT1G51190 | -1.517079262 | 0.00021724 | 0.00054076 |
| AT1G51270 | -1.985139846 | 2.7315E-10 | 1.1934E-09 |
| AT1G51380 | -1.201837816 | 3.328E-08 | 1.2371E-07 |
| AT1G07743 | 1.88059428 | 0.00568636 | 0.01134289 |
| AT1G51470 | -1.096219643 | 0.00027519 | 0.00067543 |
| AT1G51480 | -4.0703368 | 0.00603887 | 0.01199259 |
| AT1G51500 | 1.898217928 | 1.332E-143 | 9.016E-142 |
| AT1G51700 | 1.061004621 | 2.7528E-13 | 1.4412E-12 |
| AT1G51740 | 1.036001619 | 1.5443E-08 | 5.8921E-08 |
| AT1G51780 | 6.337137806 | 2.9872E-05 | 8.3096E-05 |
| AT1G51790 | -2.457686303 | 9.4446E-18 | 6.2898E-17 |
| AT1G51800 | -1.741555478 | 7.5696E-23 | 6.2598E-22 |
| AT1G51805 | -1.725306337 | 3.2562E-49 | 5.8592E-48 |
| AT1G51820 | -2.869513337 | 1.4405E-11 | 6.7963E-11 |
| AT1G51830 | -3.626538691 | 3.8057E-75 | 1.1118E-73 |
| AT1G51840 | -3.985203782 | 1.2517E-20 | 9.4376E-20 |
| AT1G51850 | -3.388688979 | 5.1589E-32 | 5.856E-31 |
| AT1G51860 | -2.911445719 | 2.4691E-26 | 2.327E-25 |
| AT1G51870 | -3.237581546 | 0.00335001 | 0.00695865 |
| AT1G51880 | -4.137143914 | 3.1817E-05 | 8.8131E-05 |
| AT1G51890 | -2.474302092 | 5.209E-22 | 4.1687E-21 |
| AT1G51940 | -2.095666811 | 2.2909E-40 | 3.3165E-39 |
| AT1G52030 | 1.541208509 | 2.2434E-07 | 7.7425E-07 |
| AT1G52050 | -1.740828704 | 2.0936E-06 | 6.5515E-06 |
| AT1G52060 | -1.540682697 | 0.00346703 | 0.00718602 |
| AT1G52080 | 2.334428226 | 2.8997E-71 | 7.9424E-70 |
| AT1G52190 | -2.914656192 | 5.1616E-56 | 1.0814E-54 |
| AT1G52200 | -1.894248818 | 1.119E-41 | 1.672E-40 |
| AT1G52290 | -2.463474128 | 1.9267E-15 | 1.1415E-14 |
| AT1G52347 | 1.157715076 | 3.0258E-07 | 1.0302E-06 |
| AT1G52560 | 4.170579695 | 2.292E-204 | 2.526E-202 |
| AT1G52565 | 4.182040627 | 1.6462E-77 | 4.9881E-76 |
| AT1G52660 | -4.086715216 | 2.6191E-06 | 8.1138E-06 |
| AT1G52680 | 7.95572551 | 6.5685E-07 | 2.1601E-06 |
| AT1G52690 | 9.895489649 | 0 | 0 |
| AT1G52700 | -1.685399137 | 1.119E-05 | 3.2618E-05 |
| AT1G52720 | 1.529459983 | 3.6516E-47 | 6.2409E-46 |
| AT1G52750 | -1.500695408 | 1.7033E-05 | 4.8711E-05 |
| AT1G52827 | 3.3365096 | 5.228E-115 | 2.662E-113 |
| AT1G52855 | 5.27607092 | 9.0785E-12 | 4.3306E-11 |
| AT1G52880 | 1.835524521 | 1.2761E-20 | 9.6157E-20 |
| AT1G52890 | 6.90899638 | 1.848E-281 | 3.484E-279 |
| AT1G53025 | 1.006497024 | 1.5699E-06 | 4.9698E-06 |
| AT1G53030 | 1.479612144 | 4.6503E-09 | 1.8514E-08 |
| AT1G53080 | 2.868228288 | 1.6905E-05 | 4.8365E-05 |
| AT1G53090 | 1.215774129 | 7.7512E-25 | 6.9272E-24 |
| AT1G53100 | 3.929124615 | 1.8135E-23 | 1.5439E-22 |
| AT1G53110 | 1.054121562 | 6.6253E-08 | 2.399E-07 |
| AT1G53130 | -4.581690631 | 2.5161E-06 | 7.8095E-06 |
| AT1G53170 | 2.47692592 | 2.8572E-60 | 6.4802E-59 |
| AT1G53190 | 1.09100001 | 7.2091E-17 | 4.5843E-16 |
| AT1G53240 | -1.092267356 | 1.0951E-77 | 3.3269E-76 |
| AT1G53320 | 1.431011321 | 2.6635E-59 | 5.9521E-58 |
| AT1G53340 | -4.274209585 | 2.206E-06 | 6.8911E-06 |
| AT1G53345 | -1.119719579 | 0.00046792 | 0.00111081 |
| AT1G53390 | -1.327185369 | 1.7478E-17 | 1.1472E-16 |
| AT1G53400 | 1.576487195 | 5.6295E-23 | 4.6877E-22 |
| AT1G53410 | 1.76706405 | 3.1167E-05 | 8.6426E-05 |
| AT1G53430 | -1.637176131 | 8.5785E-42 | 1.2877E-40 |
| AT1G53440 | -1.504940068 | 2.7473E-31 | 3.0503E-30 |
| AT1G53470 | 3.663102625 | 1.706E-200 | 1.853E-198 |
| AT1G53530 | -1.001490024 | 1.3461E-05 | 3.8932E-05 |
| AT1G53540 | 3.146902224 | 1.255E-24 | 1.1159E-23 |
| AT1G53560 | 2.364961589 | 2.9052E-76 | 8.6307E-75 |
| AT1G53570 | 1.399488117 | 5.2351E-60 | 1.1826E-58 |
| AT1G53580 | 2.182466284 | 1.586E-134 | 9.652E-133 |
| AT1G53625 | -1.832750529 | 3.1658E-08 | 1.1791E-07 |
| AT1G53635 | -2.295609293 | 0.00801198 | 0.01554433 |
| AT1G53645 | -1.360940401 | 1.0607E-40 | 1.5464E-39 |
| AT1G53670 | 1.130844102 | 9.9374E-41 | 1.4497E-39 |
| AT1G53800 | -1.489568183 | 1.064E-27 | 1.0606E-26 |
| AT1G07937 | 3.156484337 | 9.0656E-10 | 3.8226E-09 |
| AT1G53830 | -1.451720974 | 3.8906E-05 | 0.00010685 |
| AT1G53920 | -1.324355687 | 7.9237E-08 | 2.8511E-07 |
| AT1G53950 | -5.857732429 | 0.00036998 | 0.00089234 |
| AT1G53990 | -4.36868078 | 6.1143E-07 | 2.0163E-06 |
| AT1G54000 | -2.273088618 | 1.331E-113 | 6.615E-112 |
| AT1G54010 | -1.870282873 | 2.9637E-06 | 9.1427E-06 |
| AT1G54020 | 1.693743502 | 0.00081902 | 0.00187749 |
| AT1G54100 | 3.207226649 | 0 | 0 |
| AT1G54120 | 2.291927635 | 2.2956E-22 | 1.8646E-21 |
| AT1G54130 | 2.372681346 | 4.49E-171 | 3.986E-169 |
| AT1G54160 | 3.82726183 | 3.4814E-75 | 1.0184E-73 |
| AT1G54170 | 1.037329309 | 3.7099E-27 | 3.6126E-26 |
| AT1G54210 | 1.771823825 | 1.6245E-19 | 1.1782E-18 |
| AT1G54290 | 1.747014151 | 1.1682E-47 | 2.0192E-46 |
| AT1G54385 | -1.286634229 | 2.2408E-12 | 1.1105E-11 |
| AT1G54450 | -1.384337044 | 2.465E-05 | 6.9253E-05 |
| AT1G54540 | 1.454603075 | 1.3597E-11 | 6.4281E-11 |
| AT1G54570 | 1.144175949 | 6.6361E-35 | 8.1534E-34 |
| AT1G54575 | 2.418700665 | 3.645E-105 | 1.643E-103 |
| AT1G54690 | -1.436862704 | 1.7587E-13 | 9.334E-13 |
| AT1G54710 | 1.826233773 | 4.7204E-97 | 1.9546E-95 |
| AT1G54820 | -1.426745429 | 5.6783E-18 | 3.8229E-17 |
| AT1G54830 | 1.638520716 | 5.6291E-36 | 7.2235E-35 |
| AT1G54890 | -4.727938188 | 9.7919E-25 | 8.727E-24 |
| AT1G54940 | -7.096879187 | 3.9859E-05 | 0.00010931 |
| AT1G54970 | -7.07550162 | 2.7318E-06 | 8.4538E-06 |
| AT1G55020 | -1.672348029 | 1.6727E-73 | 4.7587E-72 |
| AT1G55040 | -1.321512427 | 6.3471E-10 | 2.7089E-09 |
| AT1G55110 | 1.170605466 | 9.2252E-26 | 8.5319E-25 |
| AT1G55205 | -1.651090772 | 2.316E-08 | 8.7284E-08 |
| AT1G55240 | -1.420683771 | 0.006063 | 0.01203423 |
| AT1G55280 | 1.853295718 | 4.5691E-17 | 2.9366E-16 |
| AT1G55370 | -1.132545222 | 9.5274E-13 | 4.8478E-12 |
| AT1G55380 | -2.453418055 | 0.00038713 | 0.00093084 |
| AT1G55420 | -2.545249147 | 7.5591E-05 | 0.00020006 |
| AT1G55450 | -1.330016061 | 1.7932E-21 | 1.3969E-20 |
| AT1G55490 | -1.171804932 | 3.68E-109 | 1.745E-107 |
| AT1G55510 | 1.373048478 | 2.3862E-16 | 1.4763E-15 |
| AT1G55530 | 1.57816912 | 9.5656E-55 | 1.9417E-53 |
| AT1G55580 | -2.307755735 | 0.00523264 | 0.01052247 |
| AT1G55675 | 1.002208288 | 2.0755E-08 | 7.8545E-08 |
| AT1G55680 | 1.121307219 | 1.7592E-55 | 3.6389E-54 |
| AT1G55730 | 1.524673313 | 1.1356E-33 | 1.3494E-32 |
| AT1G55760 | 2.321140553 | 1.2018E-35 | 1.5234E-34 |
| AT1G55810 | -1.152345376 | 2.2232E-15 | 1.3134E-14 |
| AT1G55850 | -1.238979069 | 1.2582E-65 | 3.1826E-64 |
| AT1G55890 | -1.022356485 | 9.7079E-15 | 5.5443E-14 |
| AT1G55990 | 1.242722983 | 1.3968E-18 | 9.7016E-18 |
| AT1G56020 | -1.034208793 | 2.3605E-05 | 6.6516E-05 |
| AT1G56050 | -1.537047959 | 2.0411E-46 | 3.4294E-45 |
| AT1G56080 | -1.035339425 | 0.00028071 | 0.0006878 |
| AT1G56110 | -1.191227506 | 3.64E-80 | 1.1471E-78 |
| AT1G56220 | 2.066666923 | 4.808E-160 | 3.863E-158 |
| AT1G56300 | 1.20903301 | 3.9155E-46 | 6.5161E-45 |
| AT1G56340 | -1.175364186 | 6.0081E-51 | 1.132E-49 |
| AT1G56430 | -1.929024476 | 0.00010604 | 0.00027604 |
| AT1G56540 | 1.661199174 | 1.0173E-05 | 2.9788E-05 |
| AT1G56600 | 6.365010765 | 0 | 0 |
| AT1G56630 | -2.128782592 | 1.108E-07 | 3.9304E-07 |
| AT1G56680 | -4.051890933 | 3.6648E-12 | 1.7886E-11 |
| AT1G56690 | -1.12360386 | 0.00100397 | 0.00226685 |
| AT1G57540 | 1.018375285 | 3.9502E-07 | 1.3297E-06 |
| AT1G57560 | -1.804569422 | 0.00030088 | 0.0007346 |
| AT1G57590 | 2.021721107 | 1.4474E-76 | 4.3169E-75 |
| AT1G57630 | -1.791987929 | 0.00159557 | 0.00350044 |
| AT1G57750 | 6.925061816 | 7.7492E-05 | 0.00020461 |
| AT1G57765 | 1.248587957 | 3.0149E-17 | 1.9548E-16 |
| AT1G57860 | -1.01119075 | 9.9717E-26 | 9.2036E-25 |
| AT1G57990 | -1.914311499 | 7.6905E-26 | 7.1327E-25 |
| AT1G58030 | 1.207791219 | 1.2437E-53 | 2.4697E-52 |
| AT1G58180 | 2.14399442 | 7.5195E-88 | 2.7406E-86 |
| AT1G58200 | 1.726313933 | 1.1115E-79 | 3.4691E-78 |
| AT1G58235 | 1.512175259 | 8.203E-27 | 7.8738E-26 |
| AT1G58270 | 2.233085624 | 3.447E-188 | 3.45E-186 |
| AT1G58340 | 2.749260779 | 1.4977E-85 | 5.217E-84 |
| AT1G58360 | 2.528517338 | 1.249E-248 | 1.874E-246 |
| AT1G58370 | -1.879631953 | 1.5647E-15 | 9.3089E-15 |
| AT1G58380 | -1.322803661 | 1.0815E-49 | 1.9678E-48 |
| AT1G58420 | 1.550264434 | 0.00309828 | 0.0064812 |
| AT1G58520 | 1.302589699 | 2.94E-28 | 2.9893E-27 |
| AT1G58590 | 2.308402488 | 1.0097E-45 | 1.6719E-44 |
| AT1G59640 | 1.114277295 | 3.5834E-05 | 9.8803E-05 |
| AT1G59660 | 1.096042419 | 6.2497E-11 | 2.8326E-10 |
| AT1G59720 | -1.177007159 | 0.00015117 | 0.00038529 |
| AT1G59780 | -1.836146598 | 0.00084254 | 0.00192755 |
| AT1G59870 | -1.395442771 | 4.3596E-72 | 1.2101E-70 |
| AT1G59940 | -2.942482244 | 6.072E-09 | 2.3923E-08 |
| AT1G59990 | -1.506153441 | 2.4301E-49 | 4.3865E-48 |
| AT1G60000 | -1.770480527 | 1.3194E-41 | 1.9649E-40 |
| AT1G60060 | -2.779030897 | 4.1831E-09 | 1.6729E-08 |
| AT1G60095 | -1.848122214 | 0.00034502 | 0.00083523 |
| AT1G60140 | 1.251271218 | 9.2901E-37 | 1.2182E-35 |
| AT1G60190 | 6.815358033 | 0 | 0 |
| AT1G60230 | -1.322583612 | 2.9375E-15 | 1.7225E-14 |
| AT1G60390 | -2.442346106 | 1.8643E-38 | 2.5639E-37 |
| AT1G60430 | 1.372219624 | 1.4489E-17 | 9.563E-17 |
| AT1G60440 | 1.507594715 | 1.7346E-42 | 2.6615E-41 |
| AT1G60450 | 5.251642643 | 2.8474E-05 | 7.9412E-05 |
| AT1G60470 | 2.782059414 | 1.615E-141 | 1.068E-139 |
| AT1G60610 | 1.206546634 | 1.3697E-13 | 7.3134E-13 |
| AT1G60640 | -1.244398397 | 2.3195E-23 | 1.9681E-22 |
| AT1G60660 | -1.356403357 | 2.3461E-12 | 1.1599E-11 |
| AT1G60750 | 5.459456778 | 2.6868E-27 | 2.6242E-26 |
| AT1G60770 | -1.330337855 | 2.0492E-36 | 2.6626E-35 |
| AT1G60950 | -1.595609072 | 4.4298E-13 | 2.2934E-12 |
| AT1G60960 | -1.153411823 | 7.1529E-07 | 2.3458E-06 |
| AT1G61050 | -2.975857911 | 2.4513E-09 | 9.9694E-09 |
| AT1G61255 | 3.091571248 | 2.4127E-63 | 5.8191E-62 |
| AT1G61260 | 1.463820812 | 8.0314E-16 | 4.8375E-15 |
| AT1G61340 | 3.373065891 | 8.5948E-77 | 2.5735E-75 |
| AT1G61390 | -1.720459316 | 0.00460977 | 0.00935483 |
| AT1G61480 | -4.126380092 | 0.00019001 | 0.00047692 |
| AT1G61560 | -2.134051284 | 7.4205E-13 | 3.7986E-12 |
| AT1G61570 | -1.251235372 | 5.4974E-26 | 5.1153E-25 |
| AT1G61580 | -1.103906523 | 1.3268E-24 | 1.1793E-23 |
| AT1G61590 | -1.238181749 | 0.0024819 | 0.00527352 |
| AT1G61600 | -1.287177757 | 5.3517E-05 | 0.00014447 |
| AT1G61640 | -1.452922231 | 5.1666E-16 | 3.146E-15 |
| AT1G61667 | -1.427214142 | 3.6894E-05 | 0.00010158 |
| AT1G61690 | 1.075089941 | 1.6803E-30 | 1.8187E-29 |
| AT1G61800 | 2.338671701 | 2.2093E-41 | 3.2669E-40 |
| AT1G61810 | -3.026115841 | 8.1488E-07 | 2.6568E-06 |
| AT1G61840 | -7.050646597 | 5.0301E-05 | 0.00013625 |
| AT1G61870 | -1.077831819 | 1.4085E-25 | 1.2901E-24 |
| AT1G61890 | 2.791558493 | 1.741E-241 | 2.437E-239 |
| AT1G61930 | -2.619684829 | 2.0856E-09 | 8.5384E-09 |
| AT1G62010 | -1.185371732 | 4.7821E-05 | 0.00012997 |
| AT1G62040 | 1.568704671 | 1.3556E-31 | 1.5206E-30 |
| AT1G62045 | 1.376906112 | 3.8788E-12 | 1.8902E-11 |
| AT1G62110 | -1.176281971 | 1.0705E-05 | 3.1275E-05 |
| AT1G62130 | 1.239286893 | 1.8571E-14 | 1.0454E-13 |
| AT1G62150 | -1.05957513 | 3.7584E-05 | 0.00010338 |
| AT1G62180 | 1.113335137 | 1.1929E-27 | 1.1854E-26 |
| AT1G62280 | -2.919239524 | 0.00688325 | 0.01352815 |
| AT1G62305 | 2.489681592 | 1.9029E-41 | 2.8192E-40 |
| AT1G08507 | 2.329709068 | 0.00037164 | 0.00089576 |
| AT1G62310 | 1.295716118 | 1.1182E-27 | 1.1136E-26 |
| AT1G62320 | -1.366843495 | 0.00477452 | 0.00966768 |
| AT1G62380 | -1.431852451 | 2.3886E-86 | 8.4363E-85 |
| AT1G62440 | -1.796406504 | 2.4512E-07 | 8.4213E-07 |
| AT1G62500 | -1.500360597 | 3.48E-16 | 2.1379E-15 |
| AT1G62510 | 1.938166262 | 2.3848E-06 | 7.4181E-06 |
| AT1G62540 | 2.207246965 | 6.386E-103 | 2.829E-101 |
| AT1G62570 | 4.245354337 | 0 | 0 |
| AT1G62590 | -1.275713574 | 0.00014116 | 0.00036167 |
| AT1G62600 | 1.373692558 | 1.7602E-24 | 1.5566E-23 |
| AT1G62610 | 1.407933733 | 2.3853E-10 | 1.0456E-09 |
| AT1G62620 | 2.843044085 | 4.5561E-10 | 1.9614E-09 |
| AT1G62710 | 3.390729208 | 1.6971E-97 | 7.0787E-96 |
| AT1G62750 | -1.50305792 | 2.255E-144 | 1.55E-142 |
| AT1G62770 | -1.528037606 | 1.0011E-05 | 2.9345E-05 |
| AT1G62800 | -1.264156925 | 2.42E-12 | 1.1951E-11 |
| AT1G62810 | 1.20653268 | 3.3499E-22 | 2.6988E-21 |
| AT1G62870 | -1.993317715 | 3.1897E-09 | 1.2828E-08 |
| AT1G62910 | -1.738115306 | 0.00046104 | 0.00109516 |
| AT1G62975 | -1.714359883 | 0.00024768 | 0.00061231 |
| AT1G62980 | -10.66985085 | 3.8262E-13 | 1.9863E-12 |
| AT1G63010 | 2.012296844 | 7.29E-156 | 5.638E-154 |
| AT1G63050 | -1.019720832 | 1.4857E-09 | 6.1554E-09 |
| AT1G63150 | -1.216070346 | 0.00106905 | 0.00240476 |
| AT1G63260 | -1.3175058 | 1.0678E-09 | 4.4742E-09 |
| AT1G63350 | -2.072445495 | 6.6296E-06 | 1.9765E-05 |
| AT1G63420 | 2.480978212 | 3.332E-147 | 2.332E-145 |
| AT1G63440 | 2.125731623 | 8.9609E-81 | 2.8357E-79 |
| AT1G63480 | -1.025095025 | 1.2084E-10 | 5.3837E-10 |
| AT1G63600 | -6.555492661 | 0.00036613 | 0.00088362 |
| AT1G63650 | -1.934104256 | 1.4142E-14 | 8.0009E-14 |
| AT1G63680 | -1.300256425 | 2.4031E-41 | 3.5511E-40 |
| AT1G63710 | -1.953395531 | 0.00707837 | 0.01387695 |
| AT1G63720 | 2.407012161 | 2.8594E-43 | 4.4562E-42 |
| AT1G63800 | 1.246223912 | 1.2639E-17 | 8.3682E-17 |
| AT1G63810 | -1.00024314 | 5.178E-22 | 4.1453E-21 |
| AT1G63840 | 1.163312892 | 6.7476E-21 | 5.1438E-20 |
| AT1G64065 | 1.871400276 | 2.4917E-07 | 8.5542E-07 |
| AT1G64110 | 3.061776021 | 8.969E-104 | 3.997E-102 |
| AT1G64160 | -6.009391557 | 0.00269377 | 0.00569137 |
| AT1G64170 | -2.474160934 | 5.9994E-28 | 6.03E-27 |
| AT1G64220 | -1.352010786 | 0.00064917 | 0.00150723 |
| AT1G64230 | 1.015493779 | 2.307E-42 | 3.5232E-41 |
| AT1G64380 | 2.096989612 | 3.3251E-42 | 5.0409E-41 |
| AT1G64390 | -1.815189462 | 1.248E-127 | 7.262E-126 |
| AT1G64405 | -2.295855825 | 6.4454E-05 | 0.00017212 |
| AT1G64450 | -1.434707868 | 0.00532543 | 0.01069115 |
| AT1G64580 | -1.275306036 | 1.934E-07 | 6.7081E-07 |
| AT1G64590 | -1.043502801 | 0.00059033 | 0.00137873 |
| AT1G64640 | -2.052625137 | 1.0113E-27 | 1.0089E-26 |
| AT1G64660 | 3.432220025 | 1.961E-275 | 3.524E-273 |
| AT1G64770 | -1.212090271 | 1.3476E-42 | 2.0705E-41 |
| AT1G64780 | -3.351245402 | 2.113E-120 | 1.129E-118 |
| AT1G64820 | 6.058143144 | 0.00233538 | 0.0049882 |
| AT1G64890 | 1.37890353 | 4.3179E-20 | 3.1893E-19 |
| AT1G64900 | -1.570933393 | 3.2941E-40 | 4.7479E-39 |
| AT1G64910 | -5.452213491 | 1.4557E-05 | 4.1925E-05 |
| AT1G64920 | -2.833567771 | 5.9434E-05 | 0.0001595 |
| AT1G64940 | -1.009784757 | 0.00038092 | 0.00091669 |
| AT1G64970 | 1.193933679 | 3.1226E-13 | 1.6292E-12 |
| AT1G65000 | 1.506513112 | 1.7895E-06 | 5.6358E-06 |
| AT1G65030 | -1.064028068 | 5.8135E-18 | 3.9115E-17 |
| AT1G65040 | 1.005397518 | 1.4585E-26 | 1.3877E-25 |
| AT1G65060 | -2.458220618 | 4.836E-174 | 4.379E-172 |
| AT1G65180 | -1.220554026 | 0.00634071 | 0.01254501 |
| AT1G65190 | -2.21535405 | 5.0256E-61 | 1.1606E-59 |
| AT1G65310 | -1.461956686 | 2.1858E-06 | 6.8306E-06 |
| AT1G65390 | -1.34379364 | 5.1443E-05 | 0.00013907 |
| AT1G65470 | -1.296732318 | 2.5376E-14 | 1.4198E-13 |
| AT1G65480 | 1.52475698 | 3.6406E-05 | 0.00010026 |
| AT1G65481 | -5.706545237 | 1.8964E-06 | 5.9565E-06 |
| AT1G65485 | -5.940641678 | 0.00389649 | 0.00800489 |
| AT1G65484 | -4.677448252 | 0.00087494 | 0.00199667 |
| AT1G65490 | -1.249986216 | 0.00867048 | 0.01670825 |
| AT1G65570 | -3.116171383 | 4.0631E-06 | 1.2362E-05 |
| AT1G65590 | -1.242712759 | 1.3175E-13 | 7.0417E-13 |
| AT1G65610 | -2.303732553 | 2.8496E-21 | 2.2072E-20 |
| AT1G65620 | -2.140538633 | 1.4337E-08 | 5.4813E-08 |
| AT1G65660 | 1.280393423 | 3.3324E-53 | 6.5545E-52 |
| AT1G65680 | -2.172640878 | 0.00329167 | 0.00684932 |
| AT1G65710 | -1.850843532 | 4.1544E-09 | 1.6629E-08 |
| AT1G65730 | -2.656421476 | 1.3896E-06 | 4.428E-06 |
| AT1G65800 | -1.32265828 | 1.0828E-17 | 7.1877E-17 |
| AT1G65845 | -2.832901611 | 1.7549E-35 | 2.2134E-34 |
| AT1G65860 | -4.170383953 | 3.098E-110 | 1.485E-108 |
| AT1G65870 | -6.299682423 | 0.00140021 | 0.00309325 |
| AT1G65985 | -2.028294241 | 1.3334E-13 | 7.1232E-13 |
| AT1G66050 | 1.375077123 | 2.0624E-05 | 5.8484E-05 |
| AT1G66140 | -1.257867416 | 4.6549E-09 | 1.8529E-08 |
| AT1G66160 | -1.008211117 | 0.0006454 | 0.00149877 |
| AT1G66190 | -1.875444368 | 2.1384E-09 | 8.7435E-09 |
| AT1G66200 | -2.098260093 | 8.057E-296 | 1.627E-293 |
| AT1G66230 | 1.753159432 | 9.1812E-08 | 3.2828E-07 |
| AT1G66250 | -1.076414433 | 1.4172E-09 | 5.8857E-09 |
| AT1G66280 | -1.019083031 | 5.8917E-21 | 4.5049E-20 |
| AT1G66370 | 3.477851688 | 0.00097066 | 0.00219643 |
| AT1G66390 | 4.753748012 | 5.483E-111 | 2.645E-109 |
| AT1G66400 | 1.058157238 | 0.00111881 | 0.00250927 |
| AT1G66500 | 2.351254602 | 4.1645E-40 | 5.9797E-39 |
| AT1G66520 | -1.745339278 | 2.2619E-13 | 1.1913E-12 |
| AT1G66540 | 1.028595869 | 9.1266E-08 | 3.2669E-07 |
| AT1G66570 | -4.78116713 | 8.8407E-10 | 3.7314E-09 |
| AT1G66760 | 3.276462784 | 2.583E-238 | 3.55E-236 |
| AT1G66800 | -1.415554691 | 5.6165E-06 | 1.689E-05 |
| AT1G66830 | 2.977673554 | 1.4815E-28 | 1.5226E-27 |
| AT1G66890 | 1.482075383 | 2.556E-16 | 1.5783E-15 |
| AT1G66930 | -4.958616098 | 2.2099E-07 | 7.635E-07 |
| AT1G66940 | -3.167603742 | 1.0528E-46 | 1.7767E-45 |
| AT1G66970 | -1.195886493 | 6.0868E-43 | 9.4217E-42 |
| AT1G67090 | -1.049941753 | 2.0367E-85 | 7.0838E-84 |
| AT1G67105 | -1.008596645 | 0.00021343 | 0.00053196 |
| AT1G67110 | -2.652542044 | 2.3288E-09 | 9.5019E-09 |
| AT1G67148 | -1.358639543 | 0.00787267 | 0.01529876 |
| AT1G67150 | -5.950689365 | 0.00320709 | 0.00668735 |
| AT1G67180 | -1.083882182 | 0.00117182 | 0.00262242 |
| AT1G67265 | 2.957330988 | 0.0016551 | 0.00362024 |
| AT1G67270 | -2.826882183 | 0.00686865 | 0.01350177 |
| AT1G67300 | 2.118428852 | 6.54E-127 | 3.731E-125 |
| AT1G67310 | 1.134145782 | 3.2407E-32 | 3.7026E-31 |
| AT1G67340 | 1.028257172 | 2.1222E-15 | 1.2547E-14 |
| AT1G67360 | 2.663549524 | 1.276E-241 | 1.809E-239 |
| AT1G67365 | 3.382425955 | 7.046E-08 | 2.5481E-07 |
| AT1G67370 | 2.021908769 | 3.9857E-13 | 2.0677E-12 |
| AT1G67470 | -1.568577222 | 9.5327E-07 | 3.0903E-06 |
| AT1G67480 | -1.029663476 | 3.7323E-27 | 3.6314E-26 |
| AT1G67630 | -1.10771835 | 6.3349E-09 | 2.4929E-08 |
| AT1G67650 | 1.581957176 | 2.1131E-09 | 8.6432E-09 |
| AT1G67730 | 1.200830135 | 2.2453E-43 | 3.5088E-42 |
| AT1G67750 | -2.1486156 | 2.0422E-37 | 2.7377E-36 |
| AT1G67810 | -1.967273355 | 1.8353E-14 | 1.0334E-13 |
| AT1G67830 | -1.076199872 | 6.1862E-12 | 2.9777E-11 |
| AT1G67850 | 1.14845612 | 2.0313E-15 | 1.2019E-14 |
| AT1G67880 | 1.371786977 | 6.2384E-18 | 4.1876E-17 |
| AT1G67920 | 3.220811878 | 4.612E-61 | 1.0683E-59 |
| AT1G67980 | -3.211049726 | 9.7043E-06 | 2.8467E-05 |
| AT1G68020 | 1.746200599 | 7.5596E-54 | 1.5077E-52 |
| AT1G68030 | 1.179938669 | 0.00920639 | 0.01764559 |
| AT1G68130 | -1.830077274 | 1.0228E-17 | 6.794E-17 |
| AT1G68140 | 1.163762398 | 6.6073E-35 | 8.1224E-34 |
| AT1G68150 | -2.139953715 | 6.8136E-09 | 2.6739E-08 |
| AT1G68190 | 1.290663058 | 1.4346E-09 | 5.9513E-09 |
| AT1G68238 | -2.769304027 | 6.8279E-36 | 8.7372E-35 |
| AT1G68250 | 6.562318178 | 1.79E-16 | 1.1137E-15 |
| AT1G68340 | 1.015214529 | 4.0235E-06 | 1.2252E-05 |
| AT1G68400 | -1.381431085 | 2.1168E-22 | 1.7212E-21 |
| AT1G68440 | 2.026813776 | 1.516E-133 | 9.173E-132 |
| AT1G68470 | 1.089671814 | 1.4392E-06 | 4.5742E-06 |
| AT1G68500 | 4.315326916 | 0 | 0 |
| AT1G68520 | -1.709252373 | 1.2991E-63 | 3.1499E-62 |
| AT1G68530 | 1.676358714 | 2.234E-144 | 1.54E-142 |
| AT1G68560 | -1.5679839 | 8.837E-134 | 5.362E-132 |
| AT1G68570 | 2.404711321 | 7.939E-151 | 5.824E-149 |
| AT1G68568 | 4.093222255 | 0.00822622 | 0.01593155 |
| AT1G68610 | 5.234535977 | 0.00192364 | 0.00416411 |
| AT1G68620 | 2.049038409 | 2.3127E-46 | 3.8771E-45 |
| AT1G68650 | -1.397211794 | 4.6962E-13 | 2.4258E-12 |
| AT1G68780 | -1.366805057 | 5.5131E-15 | 3.1926E-14 |
| AT1G68795 | 1.349729978 | 2.1913E-06 | 6.8471E-06 |
| AT1G68820 | 1.218352399 | 4.6708E-25 | 4.2006E-24 |
| AT1G68830 | 1.086949354 | 1.5276E-30 | 1.6542E-29 |
| AT1G68840 | -1.782591597 | 1.1816E-23 | 1.0128E-22 |
| AT1G68880 | 1.936821832 | 6.1948E-31 | 6.8083E-30 |
| AT1G69010 | 1.087991722 | 8.837E-19 | 6.1867E-18 |
| AT1G69040 | -2.019365836 | 2.5382E-43 | 3.9612E-42 |
| AT1G69160 | 1.090646908 | 2.0886E-09 | 8.5479E-09 |
| AT1G69170 | 1.053667764 | 1.2252E-05 | 3.556E-05 |
| AT1G69200 | -1.861583391 | 1.7912E-80 | 5.6603E-79 |
| AT1G69230 | 1.476405486 | 1.5975E-14 | 9.0199E-14 |
| AT1G69240 | -8.803959307 | 9.5926E-09 | 3.7253E-08 |
| AT1G69252 | 3.237521738 | 2.109E-273 | 3.702E-271 |
| AT1G69260 | 5.852755137 | 0 | 0 |
| AT1G69270 | 1.84519765 | 4.3371E-06 | 1.3159E-05 |
| AT1G69295 | 2.225724673 | 4.501E-177 | 4.141E-175 |
| AT1G69360 | 2.017564963 | 1.0072E-52 | 1.9691E-51 |
| AT1G69400 | 1.40288518 | 1.3171E-06 | 4.2087E-06 |
| AT1G69410 | 1.008044498 | 5.5505E-31 | 6.109E-30 |
| AT1G69430 | 2.853141916 | 1.012E-08 | 3.9197E-08 |
| AT1G69480 | 3.411341877 | 3.4527E-46 | 5.7627E-45 |
| AT1G69490 | 4.142444959 | 4.3947E-58 | 9.66E-57 |
| AT1G69540 | 1.971152931 | 0.00149509 | 0.00329267 |
| AT1G69600 | 2.1767282 | 2.1344E-17 | 1.3961E-16 |
| AT1G69610 | 2.510586122 | 3.2083E-57 | 6.9253E-56 |
| AT1G69780 | -1.719997696 | 3.058E-21 | 2.3643E-20 |
| AT1G69790 | 1.606346858 | 1.5077E-06 | 4.7797E-06 |
| AT1G69900 | -2.203675138 | 3.9961E-10 | 1.7248E-09 |
| AT1G69920 | -3.837806567 | 2.6834E-11 | 1.2439E-10 |
| AT1G69930 | -1.474795221 | 0.00137883 | 0.00305017 |
| AT1G70070 | -1.081706212 | 1.4271E-27 | 1.4132E-26 |
| AT1G70160 | 1.111418064 | 7.3585E-51 | 1.3795E-49 |
| AT1G70200 | -1.601354075 | 7.3192E-57 | 1.5593E-55 |
| AT1G70210 | -1.966301926 | 1.1427E-26 | 1.0927E-25 |
| AT1G70290 | 1.542310969 | 3.9397E-35 | 4.8932E-34 |
| AT1G70300 | 1.33942767 | 2.3604E-35 | 2.9574E-34 |
| AT1G70370 | -1.891077045 | 3.042E-137 | 1.907E-135 |
| AT1G70420 | 2.646579352 | 1.9984E-79 | 6.2034E-78 |
| AT1G70440 | 2.818573036 | 1.2398E-14 | 7.0332E-14 |
| AT1G70460 | -2.559024398 | 2.4326E-10 | 1.0659E-09 |
| AT1G70518 | 1.046248364 | 0.00032513 | 0.00078951 |
| AT1G70600 | -1.06395982 | 2.6984E-70 | 7.286E-69 |
| AT1G70640 | 3.273056323 | 1.4063E-45 | 2.3134E-44 |
| AT1G70690 | -1.217223701 | 6.5296E-05 | 0.00017431 |
| AT1G70860 | -3.554043435 | 0.00279432 | 0.00588365 |
| AT1G70880 | -2.309061284 | 1.1573E-09 | 4.8371E-09 |
| AT1G70890 | -1.825774829 | 1.6082E-95 | 6.5288E-94 |
| AT1G70900 | 1.691315708 | 8.7117E-59 | 1.9298E-57 |
| AT1G70985 | -2.007726453 | 0.00352977 | 0.00730351 |
| AT1G70990 | -3.284874013 | 2.2227E-08 | 8.3921E-08 |
| AT1G71000 | 3.079296886 | 3.954E-60 | 8.95E-59 |
| AT1G71015 | 1.231259721 | 0.00317398 | 0.00662376 |
| AT1G71050 | 1.018531581 | 3.8838E-06 | 1.1845E-05 |
| AT1G71130 | 1.206320301 | 5.6148E-10 | 2.4017E-09 |
| AT1G71180 | -1.131336398 | 2.8067E-10 | 1.2252E-09 |
| AT1G71230 | 1.228157699 | 6.4844E-10 | 2.7644E-09 |
| AT1G71240 | 1.374347727 | 2.0156E-32 | 2.3215E-31 |
| AT1G71330 | 1.676263805 | 3.0456E-08 | 1.1356E-07 |
| AT1G71340 | 2.100730971 | 4.5036E-47 | 7.6682E-46 |
| AT1G71350 | 1.07928287 | 6.1879E-19 | 4.3669E-18 |
| AT1G71360 | 1.637199653 | 7.2459E-40 | 1.032E-38 |
| AT1G71380 | -3.557319078 | 1.3057E-06 | 4.1742E-06 |
| AT1G71400 | -2.539179799 | 1.07E-24 | 9.5256E-24 |
| AT1G71520 | 3.210479229 | 4.6996E-40 | 6.7396E-39 |
| AT1G71697 | 1.155596662 | 6.5571E-22 | 5.2219E-21 |
| AT1G71740 | -3.39537935 | 9.1303E-10 | 3.8456E-09 |
| AT1G71830 | -1.045337612 | 2.6581E-08 | 9.9569E-08 |
| AT1G71850 | -1.176020917 | 4.8446E-13 | 2.4997E-12 |
| AT1G71880 | -2.519727522 | 7.8E-151 | 5.741E-149 |
| AT1G71910 | 3.322629468 | 5.8345E-29 | 6.0868E-28 |
| AT1G71950 | 1.719812667 | 1.0249E-49 | 1.8662E-48 |
| AT1G71960 | 1.628457298 | 1.2509E-36 | 1.6346E-35 |
| AT1G72100 | 5.096061582 | 7.5699E-17 | 4.803E-16 |
| AT1G72120 | 2.556785474 | 1.9417E-35 | 2.4423E-34 |
| AT1G72140 | -1.731767176 | 1.9748E-13 | 1.0452E-12 |
| AT1G72260 | 2.954267258 | 0.00124446 | 0.00277282 |
| AT1G72416 | 1.748469057 | 4.7583E-23 | 3.9826E-22 |
| AT1G72430 | -2.992528371 | 1.8687E-53 | 3.6882E-52 |
| AT1G72440 | -1.419186848 | 4.0399E-51 | 7.6366E-50 |
| AT1G72490 | 1.231476594 | 3.526E-07 | 1.1932E-06 |
| AT1G72510 | 1.003277185 | 1.8114E-30 | 1.9569E-29 |
| AT1G72540 | 2.445602711 | 1.746E-07 | 6.0774E-07 |
| AT1G72680 | 1.652135387 | 3.7006E-79 | 1.1456E-77 |
| AT1G72690 | 1.259250216 | 1.7522E-05 | 5.0029E-05 |
| AT1G72770 | 3.730215034 | 0 | 0 |
| AT1G72800 | 1.710333392 | 5.9207E-07 | 1.9558E-06 |
| AT1G72910 | -1.269265795 | 0.00090366 | 0.00205725 |
| AT1G72930 | -2.184319917 | 1.9682E-54 | 3.974E-53 |
| AT1G72970 | -1.434856972 | 9.5723E-58 | 2.092E-56 |
| AT1G73010 | 2.31749682 | 5.4329E-23 | 4.5273E-22 |
| AT1G73030 | 1.237597735 | 9.2712E-27 | 8.8879E-26 |
| AT1G73040 | 1.927022881 | 2.6954E-09 | 1.0929E-08 |
| AT1G73160 | -2.501441602 | 7.9473E-05 | 0.00020941 |
| AT1G73170 | 1.013896002 | 1.8253E-22 | 1.49E-21 |
| AT1G73210 | 1.827541548 | 8.61E-16 | 5.1792E-15 |
| AT1G73220 | 1.638554156 | 8.3034E-16 | 4.9987E-15 |
| AT1G73270 | -1.449271692 | 0.00047255 | 0.00112135 |
| AT1G73280 | -1.693382107 | 3.3533E-06 | 1.0288E-05 |
| AT1G73325 | -3.284374412 | 6.7189E-08 | 2.4318E-07 |
| AT1G73330 | -1.822919211 | 3.166E-114 | 1.584E-112 |
| AT1G73340 | -2.365835282 | 0.00053882 | 0.00126621 |
| AT1G73390 | 3.737478457 | 0 | 0 |
| AT1G73480 | 4.800249953 | 0 | 0 |
| AT1G73500 | 2.274843247 | 4.9255E-93 | 1.9276E-91 |
| AT1G73540 | 1.181721048 | 1.5124E-06 | 4.7926E-06 |
| AT1G73580 | -6.053475402 | 1.9136E-07 | 6.6413E-07 |
| AT1G73600 | -3.276624365 | 0 | 0 |
| AT1G73620 | -2.883010453 | 5.945E-46 | 9.872E-45 |
| AT1G73630 | -1.383001218 | 1.4024E-06 | 4.4634E-06 |
| AT1G73680 | 2.418340846 | 2.923E-137 | 1.837E-135 |
| AT1G73750 | 2.037728181 | 1.2962E-29 | 1.3737E-28 |
| AT1G73810 | 1.789975284 | 4.7548E-17 | 3.0525E-16 |
| AT1G73830 | -3.237847175 | 1.8475E-13 | 9.7985E-13 |
| AT1G73880 | 3.453359703 | 6.587E-274 | 1.165E-271 |
| AT1G73920 | 2.429452358 | 4.031E-232 | 5.286E-230 |
| AT1G73980 | 1.200160103 | 3.4346E-37 | 4.5746E-36 |
| AT1G74010 | -1.092815648 | 0.00146992 | 0.0032391 |
| AT1G74020 | 1.08037472 | 2.288E-25 | 2.0806E-24 |
| AT1G74070 | -2.30138663 | 2.084E-92 | 8.0727E-91 |
| AT1G74090 | -2.55226295 | 4.787E-110 | 2.285E-108 |
| AT1G74100 | -1.109449102 | 2.7582E-39 | 3.8655E-38 |
| AT1G74310 | 1.41950914 | 1.1302E-45 | 1.8672E-44 |
| AT1G74400 | -1.426549612 | 0.00931565 | 0.01783761 |
| AT1G74410 | 1.191688287 | 3.407E-08 | 1.265E-07 |
| AT1G74460 | -1.520342493 | 2.5154E-22 | 2.0381E-21 |
| AT1G74470 | -1.154110346 | 3.5565E-89 | 1.3216E-87 |
| AT1G74500 | -2.569476404 | 8.624E-19 | 6.0395E-18 |
| AT1G74660 | -1.16715839 | 0.00965384 | 0.01843967 |
| AT1G74670 | -1.166030062 | 4.3596E-08 | 1.601E-07 |
| AT1G74720 | -1.000451005 | 1.2795E-18 | 8.8949E-18 |
| AT1G74740 | 1.004431306 | 1.3429E-13 | 7.172E-13 |
| AT1G74770 | 1.036059407 | 3.2506E-12 | 1.5922E-11 |
| AT1G74790 | 1.270576411 | 4.0054E-58 | 8.8212E-57 |
| AT1G74810 | 1.451360981 | 1.3366E-10 | 5.9376E-10 |
| AT1G74840 | 1.135223024 | 6.9482E-40 | 9.9143E-39 |
| AT1G74850 | -1.128246928 | 6.6875E-36 | 8.5624E-35 |
| AT1G74860 | 1.263201667 | 1.5769E-11 | 7.4153E-11 |
| AT1G74890 | -1.706377845 | 0.0068476 | 0.01346504 |
| AT1G75030 | 2.470164154 | 6.5826E-15 | 3.7994E-14 |
| AT1G75040 | -1.553282353 | 7.8784E-21 | 5.9938E-20 |
| AT1G75120 | -1.014489673 | 1.5124E-06 | 4.7926E-06 |
| AT1G75150 | -1.07405198 | 2.7347E-06 | 8.4615E-06 |
| AT1G75170 | 1.694509393 | 7.5682E-50 | 1.387E-48 |
| AT1G09537 | 2.947228703 | 0.00015413 | 0.00039239 |
| AT1G75190 | -1.090112346 | 3.7631E-05 | 0.00010349 |
| AT1G75240 | -1.454832322 | 3.2267E-18 | 2.2016E-17 |
| AT1G75370 | 1.967318761 | 6.207E-141 | 4.058E-139 |
| AT1G75380 | 1.906317692 | 3.808E-208 | 4.322E-206 |
| AT1G75400 | 1.656545693 | 4.4936E-67 | 1.1611E-65 |
| AT1G75500 | -3.077296788 | 9.678E-296 | 1.937E-293 |
| AT1G75520 | -1.078057763 | 0.00606455 | 0.01203625 |
| AT1G75550 | -1.944248926 | 5.6298E-05 | 0.0001516 |
| AT1G75600 | 5.877053878 | 1.5296E-33 | 1.8111E-32 |
| AT1G75640 | -1.33785524 | 4.9538E-11 | 2.2623E-10 |
| AT1G75670 | -1.567469462 | 7.7904E-14 | 4.2331E-13 |
| AT1G75780 | -1.185603376 | 9.603E-10 | 4.038E-09 |
| AT1G75860 | 1.48090178 | 1.5139E-43 | 2.3739E-42 |
| AT1G75880 | 1.538197271 | 8.9544E-15 | 5.1255E-14 |
| AT1G75900 | 1.480859229 | 5.0911E-35 | 6.2959E-34 |
| AT1G75960 | -1.559700321 | 2.0582E-11 | 9.5995E-11 |
| AT1G76070 | 1.113087506 | 1.3648E-08 | 5.2275E-08 |
| AT1G76080 | -1.335958709 | 1.1686E-81 | 3.782E-80 |
| AT1G76090 | -2.202616385 | 6.3804E-64 | 1.5653E-62 |
| AT1G76110 | -1.150637187 | 7.4081E-08 | 2.6723E-07 |
| AT1G76130 | 1.015692463 | 1.9506E-11 | 9.1141E-11 |
| AT1G76180 | 3.055603233 | 0 | 0 |
| AT1G76185 | 1.115434719 | 8.5784E-05 | 0.0002252 |
| AT1G76290 | 6.84920412 | 0.00010134 | 0.00026429 |
| AT1G76380 | 1.300487111 | 6.2636E-20 | 4.6026E-19 |
| AT1G76390 | 1.479611539 | 9.4512E-10 | 3.9778E-09 |
| AT1G76490 | 1.149046846 | 5.8173E-84 | 1.9519E-82 |
| AT1G76500 | -1.673978131 | 5.4853E-07 | 1.818E-06 |
| AT1G76530 | -2.006734702 | 5.7829E-05 | 0.00015547 |
| AT1G76570 | 1.275995264 | 7.5985E-16 | 4.5816E-15 |
| AT1G76580 | 1.399915802 | 2.0637E-44 | 3.309E-43 |
| AT1G76590 | 3.296218585 | 2.784E-195 | 2.954E-193 |
| AT1G76610 | 2.044271808 | 0.00020145 | 0.00050398 |
| AT1G76640 | 3.536488817 | 0.00214351 | 0.00461249 |
| AT1G76650 | 1.715574472 | 2.6077E-08 | 9.7729E-08 |
| AT1G76670 | 1.050384676 | 3.0887E-46 | 5.1627E-45 |
| AT1G76800 | -1.622978604 | 4.1739E-05 | 0.00011421 |
| AT1G76930 | -1.316309038 | 6.613E-107 | 3.054E-105 |
| AT1G76952 | -2.438855932 | 9.7378E-07 | 3.1528E-06 |
| AT1G76960 | 1.574634662 | 1.385E-08 | 5.2992E-08 |
| AT1G76980 | 1.389917526 | 2.1021E-17 | 1.3759E-16 |
| AT1G76990 | -1.038566816 | 2.5905E-32 | 2.9701E-31 |
| AT1G09925 | 1.1360786 | 0.0001687 | 0.00042683 |
| AT1G77000 | 2.153492142 | 3.5019E-57 | 7.5447E-56 |
| AT1G77030 | -1.051204452 | 1.89E-27 | 1.8603E-26 |
| AT1G77110 | -1.007160622 | 0.00059961 | 0.00139956 |
| AT1G77120 | 5.173617926 | 0 | 0 |
| AT1G77290 | 1.210692715 | 3.6391E-08 | 1.347E-07 |
| AT1G77330 | -3.292594434 | 6.739E-125 | 3.759E-123 |
| AT1G77370 | 1.050802505 | 1.591E-19 | 1.1549E-18 |
| AT1G77380 | -1.513213555 | 3.7818E-09 | 1.5154E-08 |
| AT1G77400 | -1.25791062 | 2.0881E-09 | 8.5474E-09 |
| AT1G77450 | 3.26724671 | 0 | 0 |
| AT1G77460 | -1.911328302 | 6.0956E-32 | 6.8987E-31 |
| AT1G77520 | -2.848536011 | 2.3932E-53 | 4.7193E-52 |
| AT1G77530 | -4.374657931 | 3.6369E-07 | 1.2284E-06 |
| AT1G77630 | -1.93810487 | 1.1444E-29 | 1.2144E-28 |
| AT1G77680 | 1.494488736 | 1.5336E-69 | 4.0974E-68 |
| AT1G77690 | -2.763042008 | 1.6158E-78 | 4.9751E-77 |
| AT1G77730 | 2.302852367 | 0.00323605 | 0.00674342 |
| AT1G77750 | -1.068671282 | 7.7061E-14 | 4.1882E-13 |
| AT1G77770 | 1.261763772 | 8.5323E-22 | 6.7477E-21 |
| AT1G77855 | -1.887833051 | 0.00029452 | 0.00071983 |
| AT1G77870 | -6.404656766 | 0.00065838 | 0.00152675 |
| AT1G77890 | 1.116277076 | 4.5593E-09 | 1.8164E-08 |
| AT1G77930 | 1.467752235 | 1.5458E-49 | 2.7947E-48 |
| AT1G77990 | -1.306856456 | 4.3384E-09 | 1.7332E-08 |
| AT1G78020 | -1.38698297 | 2.0461E-71 | 5.6313E-70 |
| AT1G78070 | 3.100953453 | 0 | 0 |
| AT1G78090 | -2.744796445 | 3.3899E-26 | 3.1803E-25 |
| AT1G78120 | -2.200007282 | 9.6548E-24 | 8.2938E-23 |
| AT1G78170 | 1.091139971 | 1.7642E-17 | 1.1577E-16 |
| AT1G09787 | -2.463562828 | 0.00481385 | 0.00973868 |
| AT1G78210 | 1.460076208 | 1.0148E-34 | 1.2388E-33 |
| AT1G78290 | -1.074765124 | 0.00354299 | 0.00732883 |
| AT1G78320 | -1.526047476 | 0.00026397 | 0.00064985 |
| AT1G78370 | -2.188509601 | 2.34E-268 | 3.985E-266 |
| AT1G78390 | 3.364487591 | 4.3074E-06 | 1.3071E-05 |
| AT1G78420 | 1.034165328 | 2.8956E-24 | 2.542E-23 |
| AT1G78440 | -1.878188578 | 0.00195467 | 0.00422486 |
| AT1G78450 | -1.853328301 | 0.00030483 | 0.00074353 |
| AT1G78460 | -2.037985133 | 4.2594E-19 | 3.0275E-18 |
| AT1G78520 | -6.404656766 | 0.00065838 | 0.00152675 |
| AT1G78610 | 2.138969957 | 6.077E-150 | 4.402E-148 |
| AT1G78680 | 1.667682428 | 4.096E-141 | 2.685E-139 |
| AT1G78790 | 1.10553767 | 4.8314E-09 | 1.9208E-08 |
| AT1G78820 | -1.097574525 | 3.3366E-26 | 3.1328E-25 |
| AT1G78860 | -1.999277609 | 5.1133E-09 | 2.0261E-08 |
| AT1G78895 | 1.052255165 | 5.4478E-06 | 1.6398E-05 |
| AT1G78930 | -1.434588042 | 1.4657E-07 | 5.147E-07 |
| AT1G78960 | 1.358056022 | 2.5184E-16 | 1.5559E-15 |
| AT1G78990 | -2.371040752 | 1.0031E-12 | 5.0942E-12 |
| AT1G79060 | -2.027403001 | 1.6362E-12 | 8.1689E-12 |
| AT1G79150 | -1.154093757 | 1.3777E-28 | 1.4185E-27 |
| AT1G79250 | -6.618573149 | 0.00030095 | 0.00073463 |
| AT1G79270 | 2.584472201 | 0 | 0 |
| AT1G79310 | -3.161326599 | 7.2429E-05 | 0.00019218 |
| AT1G79320 | -2.80656462 | 0.00071091 | 0.00164203 |
| AT1G79490 | -1.233249963 | 3.4988E-09 | 1.4049E-08 |
| AT1G09863 | 1.591424593 | 0.00044099 | 0.00105083 |
| AT1G79520 | 3.274791425 | 1.193E-205 | 1.341E-203 |
| AT1G79560 | -1.04105929 | 2.3674E-37 | 3.1699E-36 |
| AT1G79580 | -1.930714185 | 0.00352979 | 0.00730351 |
| AT1G79610 | 1.686547452 | 3.0997E-47 | 5.3096E-46 |
| AT1G79670 | -1.176508569 | 1.3691E-09 | 5.6911E-09 |
| AT1G79680 | -2.062837869 | 0.00533738 | 0.01071232 |
| AT1G79700 | 2.356050783 | 1.4027E-28 | 1.4429E-27 |
| AT1G79760 | -2.010297167 | 6.9956E-05 | 0.0001859 |
| AT1G79770 | -5.156666665 | 1.3419E-08 | 5.1406E-08 |
| AT1G79860 | -7.327116441 | 1.371E-05 | 3.9635E-05 |
| AT1G79900 | 4.559083669 | 2.5061E-57 | 5.4302E-56 |
| AT1G79970 | 1.221033339 | 5.7501E-37 | 7.5747E-36 |
| AT1G80050 | -1.836915094 | 5.1941E-16 | 3.1619E-15 |
| AT1G80090 | 7.011517513 | 5.0942E-05 | 0.00013784 |
| AT1G80110 | 4.240594864 | 1.559E-182 | 1.489E-180 |
| AT1G80120 | 1.81485809 | 1.0148E-21 | 8.0003E-21 |
| AT1G80130 | 1.358978041 | 3.8379E-08 | 1.4179E-07 |
| AT1G80160 | 3.768047441 | 2.509E-61 | 5.8472E-60 |
| AT1G80170 | -1.272415057 | 6.7189E-07 | 2.2067E-06 |
| AT1G80210 | 1.338002041 | 1.4523E-22 | 1.1907E-21 |
| AT1G80240 | -3.261429203 | 3.7332E-37 | 4.9579E-36 |
| AT1G80270 | -1.606723418 | 3.5627E-46 | 5.9421E-45 |
| AT1G80280 | -1.47464118 | 6.7092E-33 | 7.83E-32 |
| AT1G80340 | -3.218819911 | 1.0018E-36 | 1.3121E-35 |
| AT1G80560 | -1.215145716 | 1.962E-29 | 2.0696E-28 |
| AT1G80610 | 1.306658959 | 4.4759E-16 | 2.7379E-15 |
| AT1G80660 | 3.455794526 | 0.00037549 | 0.00090458 |
| AT1G80720 | -1.250279172 | 6.8996E-08 | 2.496E-07 |
| AT1G80730 | -3.364627789 | 0.00126709 | 0.00282022 |
| AT1G80830 | -1.114259616 | 2.429E-22 | 1.9701E-21 |
| AT1G80920 | 2.699897083 | 1.134E-174 | 1.031E-172 |
| AT1G80960 | 2.006934927 | 1.0802E-09 | 4.5245E-09 |
| AT1G80970 | 1.494410845 | 4.7575E-06 | 1.4401E-05 |
| AT2G01008 | 2.484508941 | 5.9834E-07 | 1.9757E-06 |
| AT2G01010 | 2.656232442 | 1.8345E-19 | 1.3266E-18 |
| AT2G01020 | 2.962679951 | 1.7548E-24 | 1.5524E-23 |
| AT2G03865 | 3.993356269 | 0.00546967 | 0.01095054 |
| AT2G03875 | 3.698053801 | 0.00303357 | 0.00634991 |
| AT2G01210 | -1.519841768 | 5.0684E-07 | 1.6872E-06 |
| AT2G01290 | -1.62085254 | 4.8574E-24 | 4.2236E-23 |
| AT2G01300 | 2.30374367 | 0.00026073 | 0.00064228 |
| AT2G01490 | 1.491659871 | 1.3663E-88 | 5.0441E-87 |
| AT2G01505 | -1.100462556 | 0.00049183 | 0.0011631 |
| AT2G01530 | -1.165872708 | 2.5271E-24 | 2.2236E-23 |
| AT2G01540 | 1.041234011 | 1.5384E-10 | 6.805E-10 |
| AT2G01580 | -1.378899273 | 0.0077513 | 0.01508733 |
| AT2G01630 | -1.395218709 | 1.1352E-20 | 8.5766E-20 |
| AT2G01660 | -2.106698569 | 2.2968E-19 | 1.6516E-18 |
| AT2G01735 | 1.203775111 | 2.8267E-12 | 1.3891E-11 |
| AT2G01850 | 1.026987925 | 1.0437E-35 | 1.3274E-34 |
| AT2G01880 | -1.144963656 | 0.00105127 | 0.00236663 |
| AT2G01900 | -4.970102777 | 0.00795848 | 0.01544842 |
| AT2G01913 | -1.897543271 | 2.9757E-06 | 9.1737E-06 |
| AT2G01950 | -2.46537186 | 3.8317E-50 | 7.0504E-49 |
| AT2G01990 | -2.075149648 | 2.1757E-14 | 1.2188E-13 |
| AT2G02010 | -1.25901448 | 7.9202E-09 | 3.098E-08 |
| AT2G02020 | -2.364299733 | 3.5108E-17 | 2.2686E-16 |
| AT2G02061 | 1.435152652 | 0.00347191 | 0.00719548 |
| AT2G02070 | -1.478198996 | 6.7768E-47 | 1.1479E-45 |
| AT2G02610 | -3.801757244 | 3.1071E-05 | 8.6201E-05 |
| AT2G02680 | -3.680426417 | 3.244E-10 | 1.409E-09 |
| AT2G02690 | -6.837484359 | 0.00012128 | 0.00031331 |
| AT2G02710 | 2.757882353 | 1.058E-209 | 1.213E-207 |
| AT2G02740 | -1.84196522 | 1.8009E-35 | 2.2688E-34 |
| AT2G02780 | -1.683816617 | 1.1236E-17 | 7.4524E-17 |
| AT2G02820 | -1.04742588 | 5.9798E-07 | 1.9747E-06 |
| AT2G02950 | -2.471294439 | 5.8616E-41 | 8.6115E-40 |
| AT2G02990 | 1.421448264 | 0.00048633 | 0.00115128 |
| AT2G03090 | -1.414243371 | 1.4631E-55 | 3.0348E-54 |
| AT2G03200 | -1.297427153 | 7.5729E-05 | 0.00020037 |
| AT2G03350 | -1.020232598 | 1.6725E-09 | 6.9003E-09 |
| AT2G03360 | -4.79733187 | 1.4216E-06 | 4.5211E-06 |
| AT2G03370 | -4.528031136 | 0.001557 | 0.00342245 |
| AT2G03505 | -2.580680465 | 3.923E-08 | 1.4474E-07 |
| AT2G03620 | 1.337758426 | 1.6033E-11 | 7.5333E-11 |
| AT2G03720 | -5.921269107 | 4.585E-07 | 1.5332E-06 |
| AT2G03750 | -2.251781722 | 1.2572E-23 | 1.0767E-22 |
| AT2G03760 | 1.917910996 | 3.8506E-78 | 1.1777E-76 |
| AT2G03880 | -1.68098012 | 0.00057702 | 0.00134915 |
| AT2G04030 | -1.505371657 | 4.949E-152 | 3.69E-150 |
| AT2G04040 | -1.070963702 | 5.1228E-11 | 2.3376E-10 |
| AT2G04050 | 1.154046541 | 0.00013415 | 0.00034504 |
| AT2G04160 | 1.477159918 | 4.635E-72 | 1.285E-70 |
| AT2G04725 | 3.448015675 | 0.00032466 | 0.00078853 |
| AT2G04190 | 3.92265413 | 1.5182E-06 | 4.8097E-06 |
| AT2G04240 | 2.237624926 | 3.6664E-84 | 1.2356E-82 |
| AT2G04350 | 2.826938601 | 0 | 0 |
| AT2G04460 | -2.800611082 | 3.3256E-06 | 1.0205E-05 |
| AT2G04550 | 1.016654863 | 9.8766E-13 | 5.0199E-12 |
| AT2G04570 | 2.603349121 | 2.459E-224 | 3.066E-222 |
| AT2G04680 | -3.152513081 | 1.6089E-05 | 4.6151E-05 |
| AT2G04690 | 1.221348784 | 2.6378E-15 | 1.5507E-14 |
| AT2G04770 | -5.655734717 | 0.00790297 | 0.01534981 |
| AT2G04780 | -1.118041012 | 6.8965E-29 | 7.1718E-28 |
| AT2G04790 | -1.056859352 | 0.00025246 | 0.0006236 |
| AT2G05100 | 1.066642922 | 2.4817E-36 | 3.2171E-35 |
| AT2G05185 | 2.504463204 | 0.00020823 | 0.00051964 |
| AT2G05510 | -1.202969291 | 8.4622E-24 | 7.2995E-23 |
| AT2G05518 | 2.007222952 | 0.0001288 | 0.00033202 |
| AT2G05540 | 2.532711122 | 7.038E-259 | 1.139E-256 |
| AT2G05910 | 1.365930599 | 0.00096777 | 0.00219055 |
| AT2G05920 | -1.412459076 | 8.516E-101 | 3.673E-99 |
| AT2G05995 | -4.280516863 | 2.8676E-05 | 7.9955E-05 |
| AT2G06025 | 1.319980871 | 1.315E-39 | 1.8566E-38 |
| AT2G06200 | -2.520170174 | 0.00173329 | 0.00377751 |
| AT2G06850 | -1.528730875 | 4.3961E-73 | 1.2398E-71 |
| AT2G06925 | -1.216887665 | 2.2441E-19 | 1.6142E-18 |
| AT2G05075 | 1.573664119 | 1.5795E-05 | 4.5337E-05 |
| AT2G07671 | 1.479193399 | 1.4224E-10 | 6.3088E-10 |
| AT2G07687 | 3.602320999 | 0.00085362 | 0.00195094 |
| AT2G07727 | 1.610304303 | 0.00085325 | 0.00195029 |
| AT2G07680 | -1.724894776 | 1.8435E-21 | 1.4351E-20 |
| AT2G07690 | -1.218143276 | 8.2051E-21 | 6.2319E-20 |
| AT2G09990 | -1.003343914 | 1.1713E-35 | 1.4863E-34 |
| AT2G10940 | -4.585057302 | 0 | 0 |
| AT2G11150 | -6.95618025 | 7.2328E-05 | 0.00019193 |
| AT2G12462 | -1.490221031 | 7.8679E-12 | 3.7683E-11 |
| AT2G12646 | -1.402264201 | 4.7715E-06 | 1.4437E-05 |
| AT2G05895 | -1.40527053 | 0.00954945 | 0.01826016 |
| AT2G13550 | -1.187653392 | 0.00053347 | 0.00125494 |
| AT2G13820 | -2.110979001 | 3.4571E-34 | 4.1558E-33 |
| AT2G13960 | 2.488748061 | 2.0821E-06 | 6.5165E-06 |
| AT2G14100 | -1.810498228 | 0.00017739 | 0.00044727 |
| AT2G14260 | 1.306238928 | 7.0798E-50 | 1.2985E-48 |
| AT2G14460 | -1.862437814 | 1.2275E-15 | 7.3337E-15 |
| AT2G14520 | 1.395106296 | 1.2586E-16 | 7.8913E-16 |
| AT2G14660 | -1.835073878 | 1.6157E-14 | 9.1204E-14 |
| AT2G14750 | -1.087972824 | 5.436E-40 | 7.7809E-39 |
| AT2G14820 | -1.637031906 | 8.9348E-05 | 0.00023408 |
| AT2G14878 | 2.534353542 | 1.8741E-68 | 4.9433E-67 |
| AT2G14880 | -1.588883129 | 6.8752E-57 | 1.466E-55 |
| AT2G15020 | -1.29947043 | 0.00580463 | 0.01156264 |
| AT2G15042 | -2.279760645 | 0.00764767 | 0.01490341 |
| AT2G15050 | -2.177645822 | 1.0199E-63 | 2.4809E-62 |
| AT2G15080 | -2.144568515 | 5.6214E-08 | 2.0475E-07 |
| AT2G15090 | -2.265936471 | 2.8254E-90 | 1.0708E-88 |
| AT2G15220 | -1.481500015 | 1.5892E-14 | 8.975E-14 |
| AT2G15300 | -1.261689615 | 1.6218E-05 | 4.6498E-05 |
| AT2G15350 | -5.794958417 | 0.0060591 | 0.01202857 |
| AT2G15370 | -3.376453886 | 3.0017E-07 | 1.0226E-06 |
| AT2G15390 | -2.48461066 | 2.0678E-17 | 1.3542E-16 |
| AT2G15440 | -1.057962243 | 0.00155363 | 0.00341598 |
| AT2G15570 | 1.566057772 | 1.5109E-33 | 1.7899E-32 |
| AT2G15620 | -1.137285541 | 1.5651E-10 | 6.9177E-10 |
| AT2G15690 | -1.014436554 | 2.0562E-06 | 6.4408E-06 |
| AT2G15780 | 5.852989419 | 0.00494583 | 0.00998178 |
| AT2G15830 | 1.795619424 | 1.2442E-14 | 7.0562E-14 |
| AT2G15880 | 1.638089553 | 6.2483E-10 | 2.6682E-09 |
| AT2G15960 | 4.14680277 | 0 | 0 |
| AT2G15970 | 2.72665028 | 0 | 0 |
| AT2G16230 | -2.456890559 | 3.9412E-05 | 0.00010816 |
| AT2G16270 | -1.008337043 | 1.4027E-07 | 4.9389E-07 |
| AT2G16380 | -1.833427522 | 4.981E-18 | 3.3663E-17 |
| AT2G16385 | -1.434718865 | 0.00918687 | 0.01761112 |
| AT2G16390 | 1.012301563 | 2.1088E-09 | 8.6278E-09 |
| AT2G16430 | -1.194784559 | 8.3729E-26 | 7.7594E-25 |
| AT2G16630 | 2.30119343 | 2.2588E-97 | 9.4047E-96 |
| AT2G16660 | -1.420180909 | 4.0702E-37 | 5.3929E-36 |
| AT2G16700 | 1.562368495 | 3.1384E-42 | 4.7706E-41 |
| AT2G16710 | 1.306307989 | 1.5642E-26 | 1.4876E-25 |
| AT2G16720 | 1.62588552 | 7.0441E-23 | 5.8358E-22 |
| AT2G16760 | -1.518045774 | 5.5846E-08 | 2.0351E-07 |
| AT2G16770 | 1.009053405 | 2.8268E-07 | 9.6524E-07 |
| AT2G16890 | 1.255055989 | 8.0212E-35 | 9.8182E-34 |
| AT2G16970 | -1.725314557 | 0.00443096 | 0.00901524 |
| AT2G16980 | -2.727004221 | 6.3836E-07 | 2.1017E-06 |
| AT2G16990 | 1.964529601 | 1.3882E-32 | 1.6069E-31 |
| AT2G17033 | -1.036228175 | 5.2754E-18 | 3.559E-17 |
| AT2G17050 | -3.21507522 | 1.1341E-05 | 3.3041E-05 |
| AT2G17060 | -2.454033569 | 5.5251E-05 | 0.00014897 |
| AT2G17070 | -4.405323841 | 0.00214959 | 0.00462428 |
| AT2G17120 | 1.242295348 | 5.0494E-28 | 5.0909E-27 |
| AT2G17230 | -3.408393361 | 9.2225E-88 | 3.3506E-86 |
| AT2G17240 | -1.055646557 | 1.0166E-33 | 1.21E-32 |
| AT2G17250 | -1.046186626 | 8.722E-21 | 6.6157E-20 |
| AT2G17380 | 1.107605691 | 4.6547E-23 | 3.8987E-22 |
| AT2G17450 | 1.273487249 | 1.9378E-23 | 1.6485E-22 |
| AT2G17490 | 1.226239721 | 0.00377942 | 0.00778154 |
| AT2G17500 | 1.240588846 | 8.5451E-50 | 1.5622E-48 |
| AT2G17570 | 1.028232889 | 5.6199E-06 | 1.6898E-05 |
| AT2G17590 | -5.849119694 | 0.0046642 | 0.0094569 |
| AT2G17630 | -1.969462502 | 2.2308E-45 | 3.6512E-44 |
| AT2G17680 | 8.495458168 | 4.5575E-08 | 1.6709E-07 |
| AT2G17700 | 1.196770502 | 5.7203E-27 | 5.5163E-26 |
| AT2G17730 | 1.415807169 | 2.1751E-21 | 1.6903E-20 |
| AT2G17787 | 1.246729478 | 4.4599E-12 | 2.1673E-11 |
| AT2G17840 | 2.230820155 | 1.671E-266 | 2.784E-264 |
| AT2G17880 | 1.221976137 | 0.0004519 | 0.0010749 |
| AT2G18000 | 1.122597326 | 0.00156704 | 0.00344215 |
| AT2G18050 | 4.387219822 | 7.824E-189 | 7.899E-187 |
| AT2G18100 | 1.997537923 | 2.1204E-10 | 9.3162E-10 |
| AT2G18170 | 2.022450261 | 1.1751E-98 | 4.9741E-97 |
| AT2G18193 | 1.75748329 | 5.9401E-42 | 8.9458E-41 |
| AT2G18196 | -1.576838559 | 6.1932E-05 | 0.00016583 |
| AT2G18220 | -1.558568564 | 1.0271E-45 | 1.6981E-44 |
| AT2G18230 | 1.638698409 | 2.1296E-79 | 6.6018E-78 |
| AT2G18240 | 1.923738201 | 3.4482E-36 | 4.4575E-35 |
| AT2G18300 | -3.808473175 | 2.7954E-78 | 8.5726E-77 |
| AT2G18328 | -3.074661642 | 6.1042E-18 | 4.0999E-17 |
| AT2G18340 | 9.819015963 | 1.5862E-12 | 7.9262E-12 |
| AT2G18480 | 1.210146572 | 1.512E-09 | 6.2597E-09 |
| AT2G18520 | -1.094086974 | 5.9457E-07 | 1.9638E-06 |
| AT2G18540 | 9.265677329 | 1.0108E-09 | 4.2466E-09 |
| AT2G18550 | 5.236629962 | 9.9422E-31 | 1.0859E-29 |
| AT2G18560 | -1.150811986 | 1.8407E-06 | 5.7903E-06 |
| AT2G18570 | -1.213455665 | 2.05E-10 | 9.0206E-10 |
| AT2G18600 | 1.812420993 | 4.5808E-09 | 1.8246E-08 |
| AT2G18650 | -1.482007833 | 0.00368385 | 0.00760089 |
| AT2G18700 | 3.09981516 | 2.36E-234 | 3.149E-232 |
| AT2G18800 | -5.397858603 | 0.00101044 | 0.00228034 |
| AT2G18910 | -1.87706362 | 2.6472E-20 | 1.9725E-19 |
| AT2G18915 | 1.269810657 | 2.7825E-22 | 2.2504E-21 |
| AT2G18940 | -1.467700424 | 4.9874E-39 | 6.9301E-38 |
| AT2G18980 | -2.788745582 | 2.4449E-58 | 5.3948E-57 |
| AT2G19050 | -5.783505316 | 0.00541349 | 0.0108498 |
| AT2G19060 | -4.905734738 | 1.7286E-07 | 6.0224E-07 |
| AT2G19110 | -1.24658279 | 2.5703E-27 | 2.5137E-26 |
| AT2G19170 | -1.29258124 | 4.3428E-16 | 2.6579E-15 |
| AT2G19180 | 1.155403072 | 2.0118E-18 | 1.3895E-17 |
| AT2G19320 | 5.807852771 | 0.00528245 | 0.01060954 |
| AT2G19350 | 1.362128206 | 2.5657E-09 | 1.042E-08 |
| AT2G19385 | -1.326116576 | 2.3444E-16 | 1.4524E-15 |
| AT2G19410 | -5.900873087 | 0.00378334 | 0.00778716 |
| AT2G19490 | -1.274792081 | 2.537E-11 | 1.1772E-10 |
| AT2G19500 | -5.134473395 | 8.6658E-05 | 0.00022734 |
| AT2G19590 | -1.4412635 | 7.6698E-08 | 2.7628E-07 |
| AT2G19640 | -1.18230376 | 1.9257E-14 | 1.0824E-13 |
| AT2G19650 | -1.239445465 | 0.00027515 | 0.0006754 |
| AT2G19660 | -1.856531116 | 0.00344698 | 0.00714678 |
| AT2G19670 | -2.169648047 | 4.1706E-34 | 5.0083E-33 |
| AT2G19800 | -2.505397739 | 1.6045E-65 | 4.0496E-64 |
| AT2G19810 | 3.885844484 | 5.749E-308 | 1.237E-305 |
| AT2G19830 | 1.099583031 | 9.753E-46 | 1.616E-44 |
| AT2G19870 | -1.718973969 | 2.8817E-51 | 5.4883E-50 |
| AT2G19900 | 5.000009147 | 8.1454E-37 | 1.0687E-35 |
| AT2G19970 | -2.069392733 | 2.1089E-09 | 8.6278E-09 |
| AT2G20020 | -1.020448545 | 1.8887E-27 | 1.8599E-26 |
| AT2G20030 | -1.998277271 | 0.00906665 | 0.01740554 |
| AT2G20180 | -1.327834577 | 3.2234E-11 | 1.4855E-10 |
| AT2G20250 | 1.256064987 | 0.0017735 | 0.00385841 |
| AT2G20450 | -1.469354625 | 2.6173E-80 | 8.2596E-79 |
| AT2G20515 | -1.643274217 | 1.1518E-09 | 4.815E-09 |
| AT2G20520 | -5.131509007 | 9.4716E-05 | 0.00024746 |
| AT2G20560 | 2.872674798 | 4.644E-163 | 3.811E-161 |
| AT2G20562 | -2.132651128 | 2.2747E-05 | 6.4186E-05 |
| AT2G20570 | -1.591470539 | 2.2873E-84 | 7.7659E-83 |
| AT2G20610 | -1.681859059 | 9.0194E-97 | 3.7213E-95 |
| AT2G20690 | -1.410934121 | 1.4417E-41 | 2.1429E-40 |
| AT2G20720 | 4.533788245 | 0.00157048 | 0.0034487 |
| AT2G20740 | 1.22527332 | 2.2162E-22 | 1.8008E-21 |
| AT2G20750 | -1.673068404 | 1.3737E-10 | 6.0989E-10 |
| AT2G20770 | 1.932151666 | 9.4185E-18 | 6.2743E-17 |
| AT2G20800 | 1.992328086 | 1.412E-05 | 4.0744E-05 |
| AT2G20835 | 1.796482395 | 2.7717E-05 | 7.7471E-05 |
| AT2G20850 | -1.102561993 | 8.106E-12 | 3.8781E-11 |
| AT2G20880 | 3.559002549 | 1.3444E-45 | 2.2163E-44 |
| AT2G20950 | -1.586772749 | 7.0598E-28 | 7.0772E-27 |
| AT2G20980 | -1.775430813 | 3.1174E-11 | 1.4384E-10 |
| AT2G21050 | -1.211559311 | 4.0547E-17 | 2.6126E-16 |
| AT2G21080 | -1.8380629 | 2.2596E-09 | 9.2294E-09 |
| AT2G21090 | -1.08618067 | 1.0828E-08 | 4.1832E-08 |
| AT2G21130 | 1.849570789 | 2.133E-149 | 1.53E-147 |
| AT2G21180 | 2.0683 | 4.3804E-34 | 5.2546E-33 |
| AT2G21330 | -1.735508157 | 3.726E-133 | 2.249E-131 |
| AT2G21440 | -1.482014258 | 1.3588E-50 | 2.5267E-49 |
| AT2G21490 | 3.056163558 | 0.00076111 | 0.00175302 |
| AT2G21510 | 1.718788585 | 0.00023844 | 0.00059069 |
| AT2G21550 | -1.077363321 | 0.0069133 | 0.01358253 |
| AT2G21560 | 1.029077498 | 6.4431E-09 | 2.5342E-08 |
| AT2G21590 | 1.887210943 | 9.5633E-24 | 8.2214E-23 |
| AT2G21620 | 1.834113787 | 9.679E-189 | 9.728E-187 |
| AT2G21650 | -2.862395375 | 3.5979E-10 | 1.558E-09 |
| AT2G21780 | 3.844266012 | 0.00103675 | 0.00233625 |
| AT2G21790 | -1.438999038 | 3.704E-66 | 9.4214E-65 |
| AT2G21820 | 6.102558927 | 1.3302E-69 | 3.5581E-68 |
| AT2G21840 | -1.601015703 | 9.3147E-09 | 3.6205E-08 |
| AT2G21880 | 1.091401293 | 0.00105865 | 0.0023823 |
| AT2G21990 | 4.108493136 | 0.00776876 | 0.01511615 |
| AT2G22122 | -1.975459467 | 1.4954E-06 | 4.7421E-06 |
| AT2G22190 | 1.839474068 | 2.0446E-74 | 5.8974E-73 |
| AT2G22200 | 1.642495206 | 9.8706E-17 | 6.2316E-16 |
| AT2G22240 | 3.522155501 | 0 | 0 |
| AT2G22250 | -1.285734697 | 1.1972E-25 | 1.1006E-24 |
| AT2G22330 | -1.548535795 | 7.0334E-65 | 1.7538E-63 |
| AT2G22420 | 1.017514481 | 8.6524E-08 | 3.102E-07 |
| AT2G00740 | 3.543807426 | 0.00503741 | 0.01015405 |
| AT2G22430 | 1.618722306 | 2.173E-175 | 1.983E-173 |
| AT2G22470 | 4.191318599 | 0 | 0 |
| AT2G22500 | 1.859400064 | 3.2362E-78 | 9.911E-77 |
| AT2G22620 | -1.120743035 | 0.00180732 | 0.00392573 |
| AT2G22690 | 1.352010128 | 6.7217E-12 | 3.2294E-11 |
| AT2G22770 | -1.549903775 | 3.374E-28 | 3.4214E-27 |
| AT2G22790 | 1.218476216 | 0.00031768 | 0.00077273 |
| AT2G22810 | -4.090311614 | 0.00814888 | 0.01579517 |
| AT2G22870 | -2.246340867 | 2.3408E-34 | 2.8318E-33 |
| AT2G22890 | -1.298012935 | 0.00025628 | 0.00063209 |
| AT2G22920 | -1.646330621 | 6.5713E-12 | 3.1578E-11 |
| AT2G23050 | -2.05871287 | 9.6604E-15 | 5.5186E-14 |
| AT2G23100 | -1.555001804 | 0.00621584 | 0.01231508 |
| AT2G23110 | 4.934324671 | 2.691E-07 | 9.2036E-07 |
| AT2G23120 | 2.311294467 | 1.5412E-49 | 2.7885E-48 |
| AT2G23130 | -2.175521782 | 1.2087E-19 | 8.8053E-19 |
| AT2G23150 | 1.016067362 | 4.1326E-14 | 2.2814E-13 |
| AT2G23170 | 1.896994186 | 2.3669E-43 | 3.6964E-42 |
| AT2G23200 | -1.027591705 | 0.00068966 | 0.00159539 |
| AT2G23300 | -1.269549234 | 6.1281E-09 | 2.4136E-08 |
| AT2G23320 | 1.09662627 | 9.1716E-26 | 8.4857E-25 |
| AT2G23330 | 1.200148789 | 2.7448E-07 | 9.3836E-07 |
| AT2G23350 | -1.075031773 | 4.0648E-65 | 1.018E-63 |
| AT2G23410 | -3.470226056 | 5.0443E-05 | 0.00013662 |
| AT2G23530 | -1.066738924 | 0.00616036 | 0.01221321 |
| AT2G23540 | -1.150420349 | 2.6389E-16 | 1.6277E-15 |
| AT2G23560 | -2.684306778 | 5.0389E-16 | 3.0723E-15 |
| AT2G23600 | -3.064514237 | 2.56E-128 | 1.498E-126 |
| AT2G23620 | -2.3884986 | 2.9439E-06 | 9.0842E-06 |
| AT2G23630 | -4.283440491 | 2.7578E-20 | 2.0536E-19 |
| AT2G23680 | -2.377281956 | 6.9103E-17 | 4.3991E-16 |
| AT2G23690 | -3.268871214 | 1.1557E-10 | 5.1562E-10 |
| AT2G23770 | -1.752998707 | 3.6138E-12 | 1.7641E-11 |
| AT2G23790 | 2.003396634 | 1.0242E-92 | 3.9811E-91 |
| AT2G23810 | 1.542035384 | 1.4225E-55 | 2.9586E-54 |
| AT2G23840 | 1.77742148 | 5.79E-45 | 9.3758E-44 |
| AT2G23980 | 1.037296392 | 7.654E-32 | 8.6495E-31 |
| AT2G24010 | -3.135638645 | 0.00251987 | 0.00534972 |
| AT2G24040 | 1.35939102 | 6.4724E-12 | 3.1116E-11 |
| AT2G24060 | -1.032308977 | 7.2438E-27 | 6.9618E-26 |
| AT2G24070 | -2.703295503 | 0.00012529 | 0.00032327 |
| AT2G24120 | -1.556262426 | 1.7775E-61 | 4.1511E-60 |
| AT2G24170 | -1.319977778 | 1.0277E-12 | 5.214E-12 |
| AT2G24210 | 5.734276524 | 0.00682257 | 0.01342044 |
| AT2G24260 | 1.183568608 | 4.2568E-17 | 2.742E-16 |
| AT2G24420 | 1.097684346 | 2.5993E-45 | 4.2391E-44 |
| AT2G24430 | 1.159048858 | 2.8979E-06 | 8.9493E-06 |
| AT2G24580 | -1.458491022 | 2.0917E-18 | 1.4431E-17 |
| AT2G24600 | -1.434299353 | 5.3955E-06 | 1.6256E-05 |
| AT2G24645 | -1.249196601 | 0.0001763 | 0.00044485 |
| AT2G24720 | -3.788644732 | 1.591E-06 | 5.034E-06 |
| AT2G24850 | 3.736341163 | 2.3026E-11 | 1.0713E-10 |
| AT2G24860 | 1.112994776 | 5.8386E-19 | 4.1242E-18 |
| AT2G24980 | -5.766172547 | 6.6882E-61 | 1.5383E-59 |
| AT2G25150 | -2.112958616 | 0.00076929 | 0.00177045 |
| AT2G25160 | -1.913799271 | 6.233E-18 | 4.1852E-17 |
| AT2G25220 | -1.226115873 | 0.00061706 | 0.00143749 |
| AT2G25260 | -2.629999839 | 2.6092E-17 | 1.6985E-16 |
| AT2G25355 | -1.275675364 | 1.584E-09 | 6.5473E-09 |
| AT2G25460 | 2.483343687 | 6.5509E-30 | 6.9877E-29 |
| AT2G25480 | -1.078765095 | 4.6512E-13 | 2.4042E-12 |
| AT2G25590 | 1.225904961 | 7.0891E-15 | 4.0804E-14 |
| AT2G25620 | 1.480675749 | 4.7506E-61 | 1.0993E-59 |
| AT2G25625 | 4.766441647 | 0 | 0 |
| AT2G25680 | -1.688744607 | 4.3142E-05 | 0.00011788 |
| AT2G25690 | 1.055520834 | 4.7042E-08 | 1.7236E-07 |
| AT2G25735 | -1.910499988 | 2.4826E-06 | 7.7098E-06 |
| AT2G25780 | -6.961244023 | 8.096E-05 | 0.0002131 |
| AT2G25790 | -1.338332773 | 3.2114E-23 | 2.7047E-22 |
| AT2G25810 | -1.838244486 | 1.8529E-26 | 1.7557E-25 |
| AT2G25840 | -1.20696252 | 1.429E-28 | 1.4693E-27 |
| AT2G25900 | 2.096443343 | 6.6619E-44 | 1.0541E-42 |
| AT2G25964 | 1.396438003 | 4.6901E-11 | 2.1458E-10 |
| AT2G26040 | -1.736419634 | 3.8937E-05 | 0.00010692 |
| AT2G26150 | 1.933701148 | 3.7702E-27 | 3.6651E-26 |
| AT2G26210 | 1.216964241 | 8.3302E-21 | 6.3249E-20 |
| AT2G26290 | 1.09041289 | 1.0321E-08 | 3.9952E-08 |
| AT2G26355 | 2.103417928 | 3.3611E-36 | 4.3473E-35 |
| AT2G26360 | -1.31071364 | 0.00166336 | 0.00363691 |
| AT2G26370 | -3.273779586 | 2.549E-05 | 7.1499E-05 |
| AT2G26400 | 1.641849578 | 0.0005147 | 0.00121379 |
| AT2G26420 | -3.337881658 | 5.0081E-11 | 2.2867E-10 |
| AT2G26440 | -1.836265059 | 9.5454E-13 | 4.8559E-12 |
| AT2G26480 | -1.877128369 | 1.328E-05 | 3.8427E-05 |
| AT2G26520 | -3.541574474 | 0.00022386 | 0.00055649 |
| AT2G26560 | -2.316948225 | 1.7936E-37 | 2.4117E-36 |
| AT2G26570 | 1.494419955 | 1.9667E-81 | 6.3379E-80 |
| AT2G26600 | 1.344107215 | 3.7176E-31 | 4.1075E-30 |
| AT2G26640 | -1.220758266 | 7.6698E-18 | 5.1273E-17 |
| AT2G26650 | -2.044332244 | 8.0406E-56 | 1.6769E-54 |
| AT2G26690 | 1.660602057 | 1.2705E-51 | 2.4299E-50 |
| AT2G26695 | 1.129309599 | 2.6653E-07 | 9.1199E-07 |
| AT2G26730 | -1.141029113 | 1.3289E-36 | 1.7325E-35 |
| AT2G26800 | 1.179238173 | 1.5096E-28 | 1.5501E-27 |
| AT2G26820 | -1.682624277 | 5.55E-08 | 2.0231E-07 |
| AT2G27000 | -1.432675525 | 5.3727E-08 | 1.9595E-07 |
| AT2G27080 | -1.269721118 | 5.1852E-05 | 0.00014012 |
| AT2G27150 | 1.514962426 | 1.7473E-47 | 3.0065E-46 |
| AT2G27200 | 1.136924525 | 2.19E-16 | 1.3589E-15 |
| AT2G27300 | 2.217458485 | 0.00013908 | 0.0003567 |
| AT2G27370 | -4.527020577 | 5.0184E-18 | 3.3906E-17 |
| AT2G27380 | -4.482119629 | 0.00133415 | 0.00295793 |
| AT2G27400 | -1.428551496 | 2.5353E-11 | 1.1767E-10 |
| AT2G27402 | -5.277825059 | 1.637E-139 | 1.052E-137 |
| AT2G27420 | -3.109938206 | 1.94E-08 | 7.3515E-08 |
| AT2G27430 | 1.364451865 | 0.00043921 | 0.00104691 |
| AT2G27490 | 1.12163816 | 3.5355E-12 | 1.7273E-11 |
| AT2G27550 | 1.635679138 | 1.5306E-48 | 2.7093E-47 |
| AT2G27610 | -1.443474455 | 8.3745E-05 | 0.00022005 |
| AT2G27660 | -3.31928915 | 6.7876E-28 | 6.8103E-27 |
| AT2G27710 | -1.138047954 | 4.4185E-81 | 1.412E-79 |
| AT2G27770 | -1.053986484 | 0.00010804 | 0.00028094 |
| AT2G27775 | -1.466809246 | 1.9464E-13 | 1.0309E-12 |
| AT2G27790 | 1.109977821 | 0.00167396 | 0.00365765 |
| AT2G27810 | -1.252176873 | 1.1203E-26 | 1.0717E-25 |
| AT2G27830 | 3.323089581 | 9.342E-252 | 1.412E-249 |
| AT2G27840 | -1.160702646 | 5.0963E-35 | 6.2989E-34 |
| AT2G27950 | 1.165215336 | 1.779E-44 | 2.8624E-43 |
| AT2G28000 | -1.863923166 | 2.447E-257 | 3.904E-255 |
| AT2G28085 | -3.244122409 | 0.00356219 | 0.00736587 |
| AT2G28140 | -2.193268338 | 2.2581E-07 | 7.7861E-07 |
| AT2G28160 | -1.020086035 | 0.00107577 | 0.00241867 |
| AT2G28210 | -2.165218177 | 1.2103E-16 | 7.5965E-16 |
| AT2G28250 | -1.949494768 | 4.7115E-26 | 4.3948E-25 |
| AT2G28305 | 1.127496509 | 3.0543E-17 | 1.9792E-16 |
| AT2G28320 | 1.630680051 | 3.0694E-82 | 1.0033E-80 |
| AT2G28400 | 4.367335477 | 8.7164E-42 | 1.3075E-40 |
| AT2G28460 | -2.932004292 | 5.4245E-06 | 1.6334E-05 |
| AT2G28500 | 1.001422994 | 0.00286939 | 0.00602781 |
| AT2G28570 | 1.46173328 | 9.1296E-17 | 5.7701E-16 |
| AT2G28630 | -1.238292445 | 2.5964E-09 | 1.0537E-08 |
| AT2G28660 | -1.561551145 | 2.9197E-13 | 1.5258E-12 |
| AT2G28700 | -3.269494348 | 2.2616E-07 | 7.7947E-07 |
| AT2G28780 | 1.260615319 | 2.0698E-13 | 1.0936E-12 |
| AT2G28840 | 1.377075184 | 1.4131E-93 | 5.559E-92 |
| AT2G28910 | 1.089829544 | 5.0418E-69 | 1.3392E-67 |
| AT2G28950 | -2.576544106 | 1.093E-141 | 7.315E-140 |
| AT2G28960 | -4.594870231 | 2.1425E-32 | 2.4651E-31 |
| AT2G28970 | -3.524233197 | 3.9733E-09 | 1.5916E-08 |
| AT2G28990 | -2.761896816 | 2.7071E-10 | 1.1835E-09 |
| AT2G29150 | -6.162476299 | 0.00180867 | 0.00392828 |
| AT2G29180 | -1.196724718 | 8.7649E-15 | 5.0183E-14 |
| AT2G29260 | -1.11895906 | 0.00111486 | 0.00250091 |
| AT2G29300 | 2.460246335 | 4.4663E-19 | 3.1696E-18 |
| AT2G29320 | -1.733810369 | 2.0984E-12 | 1.0417E-11 |
| AT2G29330 | -1.55361089 | 0.00034095 | 0.00082625 |
| AT2G29340 | -1.063516304 | 1.0694E-11 | 5.08E-11 |
| AT2G29350 | 2.272799649 | 6.9381E-22 | 5.5176E-21 |
| AT2G29370 | 3.249142458 | 1.7747E-13 | 9.4145E-13 |
| AT2G29380 | 7.562147507 | 2.676E-127 | 1.546E-125 |
| AT2G29400 | 1.102847318 | 1.2848E-41 | 1.9147E-40 |
| AT2G29450 | 1.398238146 | 1.9186E-15 | 1.137E-14 |
| AT2G29460 | 1.631047087 | 5.389E-51 | 1.0162E-49 |
| AT2G29510 | -1.43613975 | 2.3825E-37 | 3.1883E-36 |
| AT2G29525 | 1.199018395 | 3.2868E-05 | 9.0954E-05 |
| AT2G29620 | -2.170454863 | 0.00116673 | 0.00261136 |
| AT2G29660 | -1.290945823 | 3.2111E-12 | 1.5735E-11 |
| AT2G29720 | -1.299869418 | 3.5575E-08 | 1.319E-07 |
| AT2G29750 | -2.455760046 | 7.511E-28 | 7.5195E-27 |
| AT2G29760 | -1.330364744 | 4.8435E-12 | 2.3468E-11 |
| AT2G29990 | 1.219674852 | 3.5263E-12 | 1.7232E-11 |
| AT2G30010 | -3.924930479 | 1.713E-99 | 7.3073E-98 |
| AT2G30020 | 1.011828808 | 9.1895E-14 | 4.9743E-13 |
| AT2G30210 | -2.968439362 | 1.749E-56 | 3.6983E-55 |
| AT2G30250 | 1.022986777 | 4.1298E-12 | 2.0099E-11 |
| AT2G30400 | 1.072048434 | 0.00054274 | 0.00127489 |
| AT2G30550 | 3.050841466 | 7.038E-224 | 8.68E-222 |
| AT2G30600 | -1.603148616 | 7.2035E-41 | 1.0556E-39 |
| AT2G30660 | -3.874777889 | 7.2662E-05 | 0.00019278 |
| AT2G30670 | -3.524605389 | 3.9433E-06 | 1.2017E-05 |
| AT2G30750 | -4.470507362 | 1.9739E-29 | 2.0802E-28 |
| AT2G30770 | 6.382183833 | 3.112E-08 | 1.1598E-07 |
| AT2G30790 | 1.605665163 | 0.00021372 | 0.00053263 |
| AT2G30830 | 4.216462534 | 4.6969E-11 | 2.1485E-10 |
| AT2G30840 | -2.153701838 | 3.5898E-11 | 1.6507E-10 |
| AT2G30890 | -2.591573082 | 1.5627E-09 | 6.4637E-09 |
| AT2G30990 | -1.448002557 | 1.4331E-20 | 1.0756E-19 |
| AT2G31020 | -1.037564721 | 1.0528E-05 | 3.0779E-05 |
| AT2G31060 | -1.058470825 | 1.7433E-35 | 2.2013E-34 |
| AT2G31081 | -6.482039875 | 0.00048751 | 0.00115348 |
| AT2G31110 | -2.666139748 | 5.6388E-12 | 2.7229E-11 |
| AT2G31130 | 1.168409677 | 2.7207E-10 | 1.189E-09 |
| AT2G31170 | -1.191691183 | 7.1302E-22 | 5.6645E-21 |
| AT2G31230 | -1.294536158 | 2.4758E-06 | 7.6907E-06 |
| AT2G31260 | 1.733945634 | 1.0707E-64 | 2.6581E-63 |
| AT2G31350 | 1.786189739 | 2.5885E-48 | 4.557E-47 |
| AT2G31570 | 1.299248014 | 6.9755E-70 | 1.8746E-68 |
| AT2G31585 | 2.381073166 | 1.1365E-29 | 1.2067E-28 |
| AT2G31680 | 1.049058777 | 5.1191E-14 | 2.8117E-13 |
| AT2G31725 | -1.544599008 | 1.1077E-12 | 5.604E-12 |
| AT2G31730 | -2.587639852 | 1.0402E-18 | 7.2642E-18 |
| AT2G31751 | 1.325923863 | 0.00017149 | 0.00043349 |
| AT2G31790 | -2.345178771 | 2.704E-112 | 1.321E-110 |
| AT2G31840 | -1.128285393 | 2.3026E-21 | 1.7876E-20 |
| AT2G31865 | 1.019630204 | 0.00089864 | 0.00204746 |
| AT2G31900 | -1.487827258 | 0.00424095 | 0.00866262 |
| AT2G31940 | 1.342868777 | 7.8662E-05 | 0.00020739 |
| AT2G31945 | 3.585354637 | 1.5359E-20 | 1.1516E-19 |
| AT2G31955 | 1.341130494 | 5.6239E-36 | 7.2208E-35 |
| AT2G31980 | 2.314119882 | 2.2613E-09 | 9.2348E-09 |
| AT2G32030 | -1.707511182 | 0.00891945 | 0.01714314 |
| AT2G32090 | 1.033098824 | 1.1946E-09 | 4.9848E-09 |
| AT2G32100 | -2.251715678 | 4.4383E-10 | 1.9117E-09 |
| AT2G32120 | 1.789004243 | 1.9187E-20 | 1.4344E-19 |
| AT2G32140 | 2.425056959 | 5.38E-07 | 1.7852E-06 |
| AT2G32150 | 1.832283453 | 4.8158E-47 | 8.1815E-46 |
| AT2G32179 | -3.003064663 | 0.00224922 | 0.00482042 |
| AT2G32190 | 1.718885695 | 2.2182E-11 | 1.0335E-10 |
| AT2G32210 | 1.664394324 | 5.4509E-07 | 1.8075E-06 |
| AT2G32220 | -1.312761786 | 5.7618E-17 | 3.6824E-16 |
| AT2G32230 | -1.095461662 | 1.4089E-37 | 1.901E-36 |
| AT2G32235 | 1.465819821 | 7.2822E-05 | 0.00019318 |
| AT2G32270 | -5.772394557 | 3.7721E-21 | 2.8997E-20 |
| AT2G32280 | -2.014478327 | 1.2956E-13 | 6.9296E-13 |
| AT2G32400 | -1.415804093 | 5.3161E-19 | 3.761E-18 |
| AT2G32510 | 3.248524185 | 3.387E-141 | 2.233E-139 |
| AT2G32540 | -1.371941564 | 2.3782E-08 | 8.9525E-08 |
| AT2G32620 | -3.748181347 | 0.00016031 | 0.00040712 |
| AT2G32650 | -1.293067319 | 2.1572E-05 | 6.1019E-05 |
| AT2G32660 | -6.097868155 | 2.3648E-07 | 8.1369E-07 |
| AT2G32720 | -1.01771009 | 2.2757E-18 | 1.5682E-17 |
| AT2G32800 | 2.849952059 | 3.704E-186 | 3.627E-184 |
| AT2G32870 | 1.166570209 | 8.7829E-13 | 4.483E-12 |
| AT2G32880 | 1.603096194 | 5.959E-12 | 2.872E-11 |
| AT2G32900 | 1.044051682 | 5.301E-17 | 3.3974E-16 |
| AT2G32930 | 1.448853347 | 2.225E-15 | 1.3142E-14 |
| AT2G32960 | -1.11640187 | 1.7361E-07 | 6.0446E-07 |
| AT2G32970 | 1.018403228 | 2.1555E-13 | 1.1371E-12 |
| AT2G32990 | -1.2580668 | 1.5488E-15 | 9.2195E-15 |
| AT2G33060 | 1.239759592 | 4.8937E-09 | 1.9435E-08 |
| AT2G33180 | -1.298534555 | 1.8213E-19 | 1.3175E-18 |
| AT2G33205 | -1.275406748 | 6.358E-05 | 0.00016994 |
| AT2G33210 | -1.108482197 | 3.0353E-44 | 4.8428E-43 |
| AT2G33310 | 1.190617609 | 2.3938E-26 | 2.2588E-25 |
| AT2G33330 | -3.063820799 | 3.1947E-92 | 1.2334E-90 |
| AT2G33380 | 7.311613195 | 0 | 0 |
| AT2G33530 | -1.007741746 | 1.178E-10 | 5.2524E-10 |
| AT2G33590 | 3.202637303 | 0 | 0 |
| AT2G33680 | -1.296754722 | 2.5974E-06 | 8.0507E-06 |
| AT2G33690 | 6.189800883 | 0.00212217 | 0.00456915 |
| AT2G33700 | 1.437540014 | 3.6886E-37 | 4.9044E-36 |
| AT2G33710 | -1.204998435 | 1.8279E-05 | 5.2079E-05 |
| AT2G33735 | -1.055398358 | 0.00355131 | 0.00734536 |
| AT2G33750 | -2.45661523 | 0.00525589 | 0.01056457 |
| AT2G33830 | 1.224154834 | 3.0091E-46 | 5.0335E-45 |
| AT2G33847 | -2.646369944 | 6.0746E-06 | 1.819E-05 |
| AT2G34050 | -1.164125753 | 4.0075E-09 | 1.605E-08 |
| AT2G34060 | -1.210064791 | 6.3481E-05 | 0.00016972 |
| AT2G34070 | 1.298316732 | 6.2078E-32 | 7.0222E-31 |
| AT2G34190 | -1.0451545 | 1.6369E-07 | 5.7167E-07 |
| AT2G34260 | -1.452695938 | 1.5812E-20 | 1.184E-19 |
| AT2G34310 | 1.331193194 | 1.8698E-50 | 3.4655E-49 |
| AT2G34357 | -1.193208306 | 4.2461E-49 | 7.6224E-48 |
| AT2G34430 | 1.841885596 | 3.7966E-14 | 2.0995E-13 |
| AT2G34490 | -1.537498155 | 3.4388E-25 | 3.1012E-24 |
| AT2G34510 | -2.524434559 | 2.3413E-63 | 5.6529E-62 |
| AT2G34640 | -1.538562366 | 1.2244E-63 | 2.9719E-62 |
| AT2G34720 | 1.851592092 | 4.7247E-49 | 8.4614E-48 |
| AT2G34790 | 1.109271426 | 9.5282E-09 | 3.7022E-08 |
| AT2G34810 | 3.157956507 | 3.2089E-30 | 3.4438E-29 |
| AT2G34850 | 4.424102246 | 1.0059E-86 | 3.5916E-85 |
| AT2G34910 | -2.006480617 | 0.00205796 | 0.00443594 |
| AT2G34960 | -1.408651267 | 0.00559706 | 0.01117844 |
| AT2G35000 | -2.289141271 | 3.1112E-13 | 1.6238E-12 |
| AT2G35040 | -1.8670975 | 3.127E-116 | 1.61E-114 |
| AT2G35060 | 2.018246803 | 1.278E-113 | 6.367E-112 |
| AT2G35070 | 5.643955221 | 2.5504E-37 | 3.4089E-36 |
| AT2G35150 | -2.33413117 | 1.8108E-09 | 7.4482E-09 |
| AT2G35200 | -6.522940093 | 0.00042188 | 0.00100814 |
| AT2G35270 | -1.697154862 | 0.00035673 | 0.0008622 |
| AT2G35290 | 1.399059779 | 0.00109672 | 0.00246312 |
| AT2G35300 | 7.221346831 | 5.622E-105 | 2.53E-103 |
| AT2G35380 | -1.891924099 | 3.6599E-14 | 2.0263E-13 |
| AT2G35612 | -6.014337406 | 0.00285446 | 0.0059992 |
| AT2G35680 | 1.272244929 | 1.6298E-51 | 3.1144E-50 |
| AT2G35690 | 1.259968758 | 5.1208E-22 | 4.1024E-21 |
| AT2G35730 | 3.257376879 | 5.9155E-18 | 3.9778E-17 |
| AT2G00880 | 1.847520801 | 3.5091E-06 | 1.074E-05 |
| AT2G08760 | 1.433168002 | 0.00196607 | 0.0042479 |
| AT2G35760 | 1.730272956 | 3.1054E-28 | 3.156E-27 |
| AT2G35770 | -1.060399355 | 0.00014201 | 0.00036372 |
| AT2G35790 | -1.055498608 | 2.6642E-19 | 1.9109E-18 |
| AT2G35840 | 1.164532293 | 1.3862E-47 | 2.3942E-46 |
| AT2G35860 | -1.089772877 | 0.0014247 | 0.00314523 |
| AT2G35890 | -4.601816657 | 0.00127264 | 0.00283174 |
| AT2G35910 | -2.453957325 | 5.5072E-06 | 1.657E-05 |
| AT2G35930 | -1.803213345 | 5.284E-15 | 3.0622E-14 |
| AT2G35940 | 1.434861099 | 1.726E-50 | 3.2017E-49 |
| AT2G35950 | 4.319288288 | 2.0105E-22 | 1.6371E-21 |
| AT2G36000 | -1.19247022 | 3.2483E-29 | 3.4059E-28 |
| AT2G36090 | -1.633005854 | 9.8576E-11 | 4.4187E-10 |
| AT2G36100 | -2.373403855 | 6.3448E-33 | 7.4086E-32 |
| AT2G36120 | -1.772740991 | 7.902E-102 | 3.441E-100 |
| AT2G36210 | -2.308509446 | 0.00038733 | 0.00093123 |
| AT2G36220 | 2.637833688 | 2.2739E-12 | 1.1259E-11 |
| AT2G36270 | 3.067493024 | 2.138E-118 | 1.121E-116 |
| AT2G36400 | -1.033200531 | 2.3209E-11 | 1.0794E-10 |
| AT2G36420 | 1.411288033 | 7.2368E-33 | 8.4371E-32 |
| AT2G36430 | -1.234519971 | 5.5363E-11 | 2.5213E-10 |
| AT2G36540 | 4.904431726 | 0.00030476 | 0.00074345 |
| AT2G36570 | -1.799449746 | 5.2445E-41 | 7.71E-40 |
| AT2G36590 | 1.685359335 | 1.1346E-34 | 1.3836E-33 |
| AT2G36620 | -1.063525535 | 1.0865E-57 | 2.37E-56 |
| AT2G36630 | 2.299971376 | 3.9859E-62 | 9.4534E-61 |
| AT2G36640 | 6.009721582 | 3.0054E-82 | 9.838E-81 |
| AT2G36690 | -3.361770392 | 4.195E-14 | 2.3153E-13 |
| AT2G36750 | 1.09944785 | 1.8818E-23 | 1.6015E-22 |
| AT2G36770 | 1.881592958 | 7.0604E-18 | 4.7337E-17 |
| AT2G36780 | 3.007780121 | 6.931E-152 | 5.151E-150 |
| AT2G36830 | 1.063796721 | 4.7873E-83 | 1.5784E-81 |
| AT2G36840 | 1.240673604 | 4.6334E-09 | 1.845E-08 |
| AT2G36870 | -1.553393098 | 1.2705E-32 | 1.4723E-31 |
| AT2G36880 | -1.554923715 | 4.869E-165 | 4.07E-163 |
| AT2G36895 | 2.114673492 | 4.1139E-90 | 1.5566E-88 |
| AT2G36900 | 1.726638212 | 4.7708E-37 | 6.3101E-36 |
| AT2G36970 | -1.412184206 | 4.3865E-15 | 2.5531E-14 |
| AT2G37000 | -2.610618028 | 0.00822946 | 0.01593647 |
| AT2G37030 | -6.110293117 | 0.00194965 | 0.00421482 |
| AT2G37040 | -1.114970065 | 5.5433E-70 | 1.495E-68 |
| AT2G37070 | 1.412379064 | 0.00120034 | 0.00268185 |
| AT2G37080 | -1.115615047 | 2.3572E-25 | 2.141E-24 |
| AT2G37100 | -1.368467458 | 6.5193E-06 | 1.9456E-05 |
| AT2G37110 | 1.023354887 | 3.773E-36 | 4.8663E-35 |
| AT2G37130 | -2.602724023 | 1.4409E-75 | 4.2474E-74 |
| AT2G37150 | 2.023399387 | 2.0701E-52 | 4.0265E-51 |
| AT2G37180 | 1.09675529 | 1.0773E-36 | 1.4093E-35 |
| AT2G37230 | -1.151405395 | 1.0038E-27 | 1.0022E-26 |
| AT2G37240 | 1.102138701 | 1.2046E-27 | 1.196E-26 |
| AT2G37280 | -1.091091684 | 0.00020644 | 0.00051557 |
| AT2G37310 | -1.153571944 | 9.5649E-07 | 3.1003E-06 |
| AT2G37380 | -2.73789285 | 1.2255E-14 | 6.9571E-14 |
| AT2G37440 | 1.534961105 | 0.00077626 | 0.0017854 |
| AT2G37460 | -2.067954921 | 2.5652E-14 | 1.4345E-13 |
| AT2G37480 | 1.128923905 | 5.6405E-44 | 8.9371E-43 |
| AT2G37510 | -1.603491214 | 2.0597E-06 | 6.449E-06 |
| AT2G37540 | -1.052348724 | 1.7346E-07 | 6.0404E-07 |
| AT2G37560 | -1.254512534 | 0.000112 | 0.00029059 |
| AT2G37570 | 1.099897401 | 1.1841E-35 | 1.5017E-34 |
| AT2G37580 | 1.158299078 | 9.2701E-08 | 3.3131E-07 |
| AT2G37600 | -1.43410727 | 1.065E-36 | 1.3941E-35 |
| AT2G37640 | -1.932727333 | 2.572E-66 | 6.5714E-65 |
| AT2G37660 | -1.127854027 | 8.1113E-84 | 2.7057E-82 |
| AT2G37690 | -1.00566944 | 3.494E-21 | 2.6896E-20 |
| AT2G37760 | 1.4797445 | 1.6492E-65 | 4.1578E-64 |
| AT2G37770 | 1.988743844 | 9.894E-115 | 5.016E-113 |
| AT2G37820 | -3.925995778 | 0.00971306 | 0.01854504 |
| AT2G37870 | 5.318575058 | 0 | 0 |
| AT2G37880 | 2.008754636 | 0.00014166 | 0.00036291 |
| AT2G37900 | 4.011853385 | 2.8356E-18 | 1.9411E-17 |
| AT2G38000 | 1.614042136 | 1.1498E-31 | 1.2955E-30 |
| AT2G38100 | -5.643499861 | 0.00970069 | 0.01852296 |
| AT2G38110 | -1.263876821 | 2.8245E-09 | 1.1422E-08 |
| AT2G38120 | -2.829529371 | 7.098E-145 | 4.908E-143 |
| AT2G38210 | -2.097934783 | 7.7989E-15 | 4.4776E-14 |
| AT2G38220 | -3.19430926 | 0.00979779 | 0.01869274 |
| AT2G38230 | -1.684235108 | 1.553E-128 | 9.106E-127 |
| AT2G38250 | 2.004436394 | 3.926E-14 | 2.1699E-13 |
| AT2G38310 | -3.934097209 | 3.545E-179 | 3.301E-177 |
| AT2G38320 | -1.56605945 | 4.0582E-06 | 1.2349E-05 |
| AT2G38340 | 2.35138035 | 0.00304228 | 0.00636696 |
| AT2G38400 | 2.080363682 | 2.4302E-77 | 7.3248E-76 |
| AT2G38410 | 1.067041788 | 1.1429E-20 | 8.6314E-20 |
| AT2G38465 | 1.653881639 | 8.9431E-21 | 6.7812E-20 |
| AT2G38490 | 1.881846553 | 0.00332848 | 0.00691665 |
| AT2G38530 | 1.669432157 | 1.042E-129 | 6.192E-128 |
| AT2G38600 | -7.05566804 | 4.6058E-05 | 0.00012546 |
| AT2G38640 | 1.247767145 | 7.5888E-08 | 2.7362E-07 |
| AT2G38720 | -1.119296987 | 0.0005588 | 0.00131048 |
| AT2G38740 | 1.773941102 | 3.3757E-54 | 6.7799E-53 |
| AT2G38820 | 2.490334671 | 1.4813E-62 | 3.5353E-61 |
| AT2G38860 | -1.865859696 | 2.8407E-95 | 1.1492E-93 |
| AT2G38905 | 7.027764305 | 4.7224E-05 | 0.00012845 |
| AT2G38920 | 2.806543178 | 3.8157E-07 | 1.2856E-06 |
| AT2G38940 | -3.717418647 | 5.7124E-49 | 1.0206E-47 |
| AT2G38970 | -1.156950972 | 5.508E-14 | 3.0238E-13 |
| AT2G39000 | 1.18513811 | 5.133E-22 | 4.1108E-21 |
| AT2G39030 | 6.429252175 | 3.5615E-48 | 6.246E-47 |
| AT2G39040 | -4.455058744 | 2.2346E-07 | 7.7146E-07 |
| AT2G39050 | 3.324182013 | 8.549E-253 | 1.3E-250 |
| AT2G39100 | 1.482119976 | 2.0113E-14 | 1.1292E-13 |
| AT2G39110 | 2.276777693 | 5.7789E-48 | 1.0057E-46 |
| AT2G39120 | -1.185283774 | 0.00358574 | 0.00741052 |
| AT2G39140 | -1.325787653 | 7.3966E-28 | 7.4083E-27 |
| AT2G39180 | -2.510764441 | 3.3339E-17 | 2.1573E-16 |
| AT2G39220 | -1.370465527 | 5.4916E-16 | 3.3385E-15 |
| AT2G39230 | -1.302007189 | 0.00074638 | 0.00172102 |
| AT2G39250 | 1.23869928 | 3.5322E-26 | 3.311E-25 |
| AT2G39310 | -1.132917344 | 4.5276E-08 | 1.6605E-07 |
| AT2G39350 | 2.756881793 | 8.531E-205 | 9.449E-203 |
| AT2G39370 | -1.662685607 | 0.00789222 | 0.01533414 |
| AT2G39390 | -1.050255192 | 7.8793E-43 | 1.2163E-41 |
| AT2G39410 | -2.931667259 | 7.7478E-08 | 2.79E-07 |
| AT2G39430 | -3.003104044 | 1.6923E-21 | 1.3214E-20 |
| AT2G39450 | 1.69165351 | 2.2685E-72 | 6.3351E-71 |
| AT2G39510 | 1.72509531 | 1.7254E-28 | 1.7685E-27 |
| AT2G39518 | -2.332829178 | 7.1302E-12 | 3.4199E-11 |
| AT2G39530 | -4.475386302 | 1.1122E-30 | 1.2112E-29 |
| AT2G39700 | -1.657655284 | 1.0363E-79 | 3.2433E-78 |
| AT2G39725 | 1.074936002 | 1.7571E-06 | 5.5359E-06 |
| AT2G39800 | 4.417768733 | 0 | 0 |
| AT2G39870 | -1.315040708 | 9.8875E-26 | 9.1296E-25 |
| AT2G39980 | 1.096144534 | 4.0909E-12 | 1.9914E-11 |
| AT2G40000 | 2.508332567 | 4.561E-116 | 2.344E-114 |
| AT2G40060 | 1.003539904 | 2.0377E-42 | 3.1182E-41 |
| AT2G40113 | -3.513947349 | 1.2109E-15 | 7.2361E-15 |
| AT2G40130 | 1.10330048 | 8.104E-10 | 3.4317E-09 |
| AT2G40150 | -1.49851073 | 5.6587E-09 | 2.2333E-08 |
| AT2G40170 | 5.936617234 | 1.3563E-50 | 2.5241E-49 |
| AT2G40180 | 1.292981965 | 0.00910036 | 0.01746289 |
| AT2G40240 | -1.28481418 | 0.00011296 | 0.00029288 |
| AT2G40330 | -2.765502423 | 1.2598E-18 | 8.7635E-18 |
| AT2G40340 | 2.130035963 | 2.1088E-75 | 6.1843E-74 |
| AT2G40400 | -1.156047576 | 8.4565E-23 | 6.9882E-22 |
| AT2G40420 | 1.471354319 | 7.1882E-14 | 3.9151E-13 |
| AT2G40460 | -3.248463544 | 8.2841E-83 | 2.7235E-81 |
| AT2G40470 | -1.302005993 | 0.0094214 | 0.01803041 |
| AT2G40480 | -1.585115499 | 1.8709E-25 | 1.7054E-24 |
| AT2G40490 | -1.160856234 | 9.163E-53 | 1.793E-51 |
| AT2G40550 | -1.19837417 | 1.9902E-13 | 1.0528E-12 |
| AT2G40590 | -1.148677603 | 7.4266E-43 | 1.148E-41 |
| AT2G40610 | -3.738028155 | 5.0149E-16 | 3.0585E-15 |
| AT2G40690 | -1.196531602 | 1.5879E-10 | 7.0145E-10 |
| AT2G40700 | -1.212534455 | 4.7637E-21 | 3.6497E-20 |
| AT2G40810 | 1.316558197 | 2.4109E-09 | 9.8158E-09 |
| AT2G40830 | 1.010252585 | 4.3182E-19 | 3.0683E-18 |
| AT2G40880 | 1.247836958 | 1.9722E-26 | 1.8648E-25 |
| AT2G40925 | 5.734276524 | 0.00682257 | 0.01342044 |
| AT2G40970 | 1.141094374 | 1.1753E-09 | 4.9076E-09 |
| AT2G41070 | 1.718131134 | 1.8718E-13 | 9.9206E-13 |
| AT2G41090 | -2.865608887 | 3.448E-112 | 1.681E-110 |
| AT2G41100 | -1.227304244 | 0.00227019 | 0.00486262 |
| AT2G41170 | 2.277892317 | 1.1168E-90 | 4.2467E-89 |
| AT2G41190 | 6.99741821 | 3.321E-277 | 6.061E-275 |
| AT2G41200 | 1.677216816 | 1.5908E-17 | 1.0472E-16 |
| AT2G41210 | 2.157412216 | 1.4591E-20 | 1.0948E-19 |
| AT2G41250 | 1.068148853 | 3.0619E-07 | 1.0419E-06 |
| AT2G41312 | 1.62586775 | 1.5629E-12 | 7.8151E-12 |
| AT2G41370 | -1.235509038 | 1.5266E-07 | 5.3522E-07 |
| AT2G41410 | 1.636567043 | 2.239E-127 | 1.297E-125 |
| AT2G41430 | 1.315889226 | 3.065E-120 | 1.63E-118 |
| AT2G41505 | 1.577831222 | 0.00845941 | 0.01634431 |
| AT2G41630 | 1.041224457 | 1.7099E-30 | 1.8499E-29 |
| AT2G41650 | -1.129760836 | 6.1935E-22 | 4.9444E-21 |
| AT2G41780 | -1.17725328 | 0.0001156 | 0.00029938 |
| AT2G41820 | -1.194296377 | 1.2856E-25 | 1.1804E-24 |
| AT2G41870 | 3.932340541 | 0 | 0 |
| AT2G41880 | 1.001640478 | 5.4636E-11 | 2.4892E-10 |
| AT2G41900 | 1.053731342 | 4.0016E-51 | 7.5706E-50 |
| AT2G41940 | -2.198066631 | 4.2474E-32 | 4.8358E-31 |
| AT2G41950 | -2.576594453 | 1.02E-41 | 1.5261E-40 |
| AT2G41970 | -3.020451558 | 5.1961E-09 | 2.0575E-08 |
| AT2G41990 | -2.047320005 | 1.4387E-06 | 4.573E-06 |
| AT2G42060 | -3.474593342 | 6.1037E-18 | 4.0999E-17 |
| AT2G42065 | 2.816763376 | 0.0009361 | 0.00212454 |
| AT2G42160 | 1.29364994 | 9.3326E-22 | 7.3679E-21 |
| AT2G42170 | -1.184785162 | 4.23E-06 | 1.2842E-05 |
| AT2G42220 | -1.248459425 | 4.75E-60 | 1.0741E-58 |
| AT2G42270 | 1.354258366 | 2.3378E-45 | 3.8209E-44 |
| AT2G42280 | -1.822806731 | 4.2071E-11 | 1.9287E-10 |
| AT2G42330 | 1.153390631 | 2.9677E-13 | 1.5501E-12 |
| AT2G42350 | -2.968409979 | 9.6081E-18 | 6.3968E-17 |
| AT2G42380 | -2.595196453 | 4.4846E-15 | 2.6075E-14 |
| AT2G42530 | 2.628947076 | 0 | 0 |
| AT2G42540 | 3.058068195 | 0 | 0 |
| AT2G42560 | 8.801180425 | 7.4056E-06 | 2.1955E-05 |
| AT2G42610 | -1.843425094 | 3.6408E-11 | 1.6731E-10 |
| AT2G42620 | 1.188374972 | 3.9104E-23 | 3.2837E-22 |
| AT2G42660 | -2.499543769 | 0.00027099 | 0.00066598 |
| AT2G42710 | -1.377689763 | 2.5189E-34 | 3.0424E-33 |
| AT2G42730 | -1.114060973 | 0.00708834 | 0.01389413 |
| AT2G42750 | 2.191953029 | 2.5289E-15 | 1.4886E-14 |
| AT2G42790 | 2.586897496 | 2.038E-265 | 3.37E-263 |
| AT2G42800 | -1.617824068 | 5.7897E-06 | 1.7361E-05 |
| AT2G42850 | -4.866305011 | 5.3446E-07 | 1.7739E-06 |
| AT2G42870 | -1.217158124 | 0.00361 | 0.00745526 |
| AT2G42890 | 1.431747158 | 1.31E-27 | 1.2996E-26 |
| AT2G42950 | 1.122555488 | 3.5066E-09 | 1.4078E-08 |
| AT2G42975 | 1.477780213 | 1.4795E-12 | 7.4171E-12 |
| AT2G43000 | -3.075196731 | 1.3991E-21 | 1.0966E-20 |
| AT2G43020 | 1.873653105 | 3.68E-110 | 1.76E-108 |
| AT2G43050 | -1.640705715 | 0.00243767 | 0.00518727 |
| AT2G43100 | -2.53566661 | 2.942E-167 | 2.523E-165 |
| AT2G43110 | -1.315145857 | 1.2006E-18 | 8.3591E-18 |
| AT2G43120 | 1.067137579 | 3.0958E-15 | 1.813E-14 |
| AT2G43150 | -1.198394397 | 5.3683E-87 | 1.9258E-85 |
| AT2G43200 | -1.236751306 | 1.8216E-06 | 5.7336E-06 |
| AT2G43210 | 1.001408891 | 3.7375E-14 | 2.0683E-13 |
| AT2G43240 | 1.91192718 | 2.6261E-61 | 6.1141E-60 |
| AT2G43290 | 1.030290304 | 1.1217E-17 | 7.4416E-17 |
| AT2G43320 | 1.302930616 | 4.9538E-16 | 3.0237E-15 |
| AT2G43330 | 1.744578131 | 8.1177E-40 | 1.154E-38 |
| AT2G43360 | -1.888445794 | 8.7586E-81 | 2.7755E-79 |
| AT2G43480 | -1.635452792 | 0.00590371 | 0.01174155 |
| AT2G43500 | 1.710424549 | 2.6603E-39 | 3.7329E-38 |
| AT2G43510 | -1.042238858 | 1.1703E-08 | 4.5057E-08 |
| AT2G43550 | -1.836012472 | 4.9229E-87 | 1.7688E-85 |
| AT2G43570 | 1.135200773 | 2.85E-21 | 2.2072E-20 |
| AT2G43600 | -6.455099836 | 0.00061865 | 0.00144091 |
| AT2G43610 | -1.388350963 | 2.2389E-27 | 2.1962E-26 |
| AT2G43620 | 1.706197135 | 2.4715E-05 | 6.9421E-05 |
| AT2G43820 | 2.196501327 | 4.756E-197 | 5.094E-195 |
| AT2G43870 | -1.853211266 | 0.0023412 | 0.0049983 |
| AT2G43880 | -1.428896173 | 0.00173105 | 0.00377298 |
| AT2G43910 | -1.546690912 | 1.054E-110 | 5.071E-109 |
| AT2G43930 | 1.512802685 | 0.00962576 | 0.01839221 |
| AT2G44010 | -1.967788233 | 2.3585E-08 | 8.8825E-08 |
| AT2G44060 | 1.175091409 | 6.6438E-86 | 2.3249E-84 |
| AT2G44080 | 1.690895791 | 6.0781E-15 | 3.5117E-14 |
| AT2G44110 | -2.238667203 | 6.131E-06 | 1.835E-05 |
| AT2G44210 | -1.486363549 | 6.9737E-44 | 1.1026E-42 |
| AT2G44280 | 1.523527975 | 1.1852E-16 | 7.4431E-16 |
| AT2G44300 | 1.004503716 | 2.7126E-14 | 1.514E-13 |
| AT2G44340 | -2.796124183 | 0.00331372 | 0.00688892 |
| AT2G44370 | -1.709764149 | 4.872E-09 | 1.9366E-08 |
| AT2G44380 | -3.556226838 | 5.8772E-25 | 5.2731E-24 |
| AT2G44410 | 1.339781224 | 2.8896E-35 | 3.6066E-34 |
| AT2G44460 | 3.038224543 | 9.8614E-75 | 2.8662E-73 |
| AT2G44510 | -1.658061284 | 8.4586E-14 | 4.5885E-13 |
| AT2G44580 | -1.131479984 | 0.00016433 | 0.00041674 |
| AT2G44600 | 1.011648622 | 3.2965E-06 | 1.0124E-05 |
| AT2G44640 | -1.718083372 | 5.189E-57 | 1.1117E-55 |
| AT2G44740 | -3.077613288 | 3.5751E-07 | 1.209E-06 |
| AT2G44830 | -1.193406883 | 1.6516E-17 | 1.086E-16 |
| AT2G44850 | 1.431611434 | 3.1217E-05 | 8.6533E-05 |
| AT2G44940 | -1.683375761 | 5.5242E-24 | 4.7979E-23 |
| AT2G44970 | 1.623874907 | 2.6672E-62 | 6.3456E-61 |
| AT2G45170 | 1.744689715 | 4.4082E-39 | 6.144E-38 |
| AT2G45180 | -4.740942301 | 0 | 0 |
| AT2G45380 | 1.400453881 | 5.6837E-45 | 9.2101E-44 |
| AT2G45430 | -1.631220492 | 5.3684E-19 | 3.7968E-18 |
| AT2G45470 | -1.934384518 | 2.225E-147 | 1.567E-145 |
| AT2G45490 | 1.583428841 | 2.4842E-08 | 9.3331E-08 |
| AT2G45500 | 1.573460354 | 1.6681E-25 | 1.5255E-24 |
| AT2G45570 | 4.313924079 | 1.762E-237 | 2.407E-235 |
| AT2G45600 | 1.908583672 | 2.3545E-29 | 2.4756E-28 |
| AT2G45660 | 2.204920966 | 2.2958E-73 | 6.5152E-72 |
| AT2G45670 | 1.284583196 | 8.394E-66 | 2.128E-64 |
| AT2G45750 | -1.249314794 | 5.9327E-09 | 2.3382E-08 |
| AT2G45820 | 1.692080082 | 1.674E-152 | 1.265E-150 |
| AT2G45980 | 1.503999767 | 7.5769E-62 | 1.784E-60 |
| AT2G45990 | 1.020776027 | 1.1915E-20 | 8.9958E-20 |
| AT2G46070 | 1.255800041 | 1.6197E-12 | 8.088E-12 |
| AT2G46100 | 1.570416846 | 2.6962E-55 | 5.537E-54 |
| AT2G46240 | 1.4772267 | 3.119E-31 | 3.4545E-30 |
| AT2G46260 | 1.423725006 | 1.2056E-57 | 2.6246E-56 |
| AT2G46270 | 3.887478242 | 0 | 0 |
| AT2G46300 | 1.732769467 | 0.00511982 | 0.01030839 |
| AT2G46400 | 1.079209671 | 0.00586258 | 0.01166992 |
| AT2G46430 | -1.049732487 | 3.1854E-07 | 1.0818E-06 |
| AT2G46440 | -2.54711615 | 4.3581E-09 | 1.7408E-08 |
| AT2G46490 | 1.386074184 | 2.4222E-34 | 2.9272E-33 |
| AT2G46495 | -2.507510264 | 1.8941E-08 | 7.1823E-08 |
| AT2G46510 | 1.285611322 | 3.9553E-31 | 4.3659E-30 |
| AT2G46535 | -1.254696922 | 6.343E-15 | 3.6638E-14 |
| AT2G46550 | 1.253714735 | 3.7608E-36 | 4.8532E-35 |
| AT2G46590 | 1.235819448 | 4.5659E-15 | 2.6542E-14 |
| AT2G46600 | 1.53178113 | 1.921E-64 | 4.7536E-63 |
| AT2G46620 | 1.05156275 | 9.1372E-14 | 4.9472E-13 |
| AT2G46650 | -2.136351189 | 6.0537E-86 | 2.1217E-84 |
| AT2G46680 | 5.049425591 | 0 | 0 |
| AT2G46735 | 1.242163673 | 4.4212E-08 | 1.6228E-07 |
| AT2G46740 | -1.230911539 | 3.1062E-09 | 1.2512E-08 |
| AT2G46750 | -2.385807913 | 1.0303E-56 | 2.1889E-55 |
| AT2G46780 | -1.483542649 | 9.0274E-10 | 3.8079E-09 |
| AT2G46790 | 3.102283599 | 8.7135E-08 | 3.123E-07 |
| AT2G46800 | 1.004218124 | 2.9979E-35 | 3.7377E-34 |
| AT2G46830 | 5.67298231 | 6.9257E-65 | 1.7289E-63 |
| AT2G46860 | -4.528382726 | 7.1956E-06 | 2.1369E-05 |
| AT2G46870 | -1.32964234 | 1.2814E-13 | 6.8551E-13 |
| AT2G46890 | -2.552735087 | 4.2604E-27 | 4.1364E-26 |
| AT2G46950 | 2.989072844 | 4.0095E-21 | 3.0801E-20 |
| AT2G47040 | 5.599865414 | 0.00876618 | 0.01687414 |
| AT2G47130 | -1.03769019 | 2.4614E-05 | 6.9179E-05 |
| AT2G47180 | 2.148504677 | 2.6988E-84 | 9.1358E-83 |
| AT2G47190 | 2.523348231 | 0.00101214 | 0.00228372 |
| AT2G47270 | 2.295837385 | 3.8424E-16 | 2.3567E-15 |
| AT2G47360 | -2.667507196 | 3.5778E-13 | 1.8586E-12 |
| AT2G47370 | -1.759389897 | 5.2037E-09 | 2.0602E-08 |
| AT2G47440 | -1.611671048 | 1.0904E-28 | 1.1262E-27 |
| AT2G47450 | -1.391453265 | 3.4578E-71 | 9.4596E-70 |
| AT2G47485 | 2.640819431 | 2.428E-102 | 1.065E-100 |
| AT2G47530 | -2.740759245 | 0.00051547 | 0.0012151 |
| AT2G47540 | -8.064795526 | 3.7417E-14 | 2.0696E-13 |
| AT2G47550 | -2.123610747 | 6.6311E-09 | 2.605E-08 |
| AT2G47600 | 1.422510295 | 2.818E-42 | 4.2921E-41 |
| AT2G47700 | 1.195991229 | 2.5394E-38 | 3.4799E-37 |
| AT2G47710 | 1.205697378 | 2.1579E-59 | 4.8269E-58 |
| AT2G47730 | 1.34261137 | 9.2673E-73 | 2.604E-71 |
| AT2G47750 | -1.89614273 | 8.8696E-05 | 0.00023248 |
| AT2G47770 | 8.833259018 | 0 | 0 |
| AT2G47780 | 5.844813499 | 5.977E-189 | 6.061E-187 |
| AT2G47810 | 2.386064984 | 3.4048E-06 | 1.0436E-05 |
| AT2G47860 | -1.126140859 | 8.4334E-09 | 3.2886E-08 |
| AT2G47870 | 2.749876062 | 6.9896E-05 | 0.00018576 |
| AT2G47890 | 1.19057043 | 7.9504E-50 | 1.4558E-48 |
| AT2G47910 | -1.602848643 | 7.1628E-47 | 1.2124E-45 |
| AT2G47930 | -1.549653129 | 0.0053962 | 0.01082181 |
| AT2G47950 | 2.42382113 | 9.5078E-08 | 3.3954E-07 |
| AT2G47990 | -1.07246258 | 2.4846E-15 | 1.4633E-14 |
| AT2G48080 | -2.199511378 | 9.8509E-12 | 4.6893E-11 |
| AT2G48120 | -1.25773779 | 1.9489E-20 | 1.4564E-19 |
| AT3G01070 | -1.912427183 | 0.00568415 | 0.01133947 |
| AT3G01100 | 1.683062235 | 1.8568E-66 | 4.76E-65 |
| AT3G01120 | -1.01114993 | 5.6462E-70 | 1.5209E-68 |
| AT3G01170 | 1.313785862 | 4.0952E-18 | 2.7767E-17 |
| AT3G01190 | -2.114495742 | 1.8161E-37 | 2.4403E-36 |
| AT3G01220 | -1.778426743 | 2.0768E-09 | 8.5086E-09 |
| AT3G01260 | -3.207725998 | 1.0968E-12 | 5.5514E-12 |
| AT3G01290 | -2.195121633 | 1.8704E-98 | 7.8593E-97 |
| AT3G01345 | -3.065030649 | 0.00025387 | 0.00062688 |
| AT3G01350 | 1.019079806 | 0.00728996 | 0.01425382 |
| AT3G01420 | -1.276660723 | 6.592E-110 | 3.14E-108 |
| AT3G01440 | -1.745250927 | 6.9099E-52 | 1.3327E-50 |
| AT3G01490 | 1.154796178 | 2.478E-25 | 2.248E-24 |
| AT3G01516 | -1.626700622 | 0.00074701 | 0.00172228 |
| AT3G01520 | 1.169588104 | 5.0747E-33 | 5.9438E-32 |
| AT3G01590 | 1.836207926 | 2.903E-89 | 1.0805E-87 |
| AT3G01600 | 1.93685055 | 0.00030197 | 0.00073695 |
| AT3G01650 | 1.95620523 | 4.5059E-53 | 8.8474E-52 |
| AT3G01770 | 1.246649701 | 3.4714E-30 | 3.722E-29 |
| AT3G01860 | -1.78876051 | 1.0483E-20 | 7.9327E-20 |
| AT3G01960 | 1.552593718 | 5.2796E-05 | 0.00014259 |
| AT3G02020 | -2.648187295 | 1.528E-143 | 1.031E-141 |
| AT3G02060 | -1.483495242 | 5.1804E-48 | 9.0363E-47 |
| AT3G02110 | -1.329458855 | 1.0513E-33 | 1.2506E-32 |
| AT3G02140 | 2.651473483 | 2.841E-100 | 1.2207E-98 |
| AT3G02150 | 1.971228308 | 2.4046E-45 | 3.9272E-44 |
| AT3G02240 | -2.456017844 | 1.0065E-06 | 3.2553E-06 |
| AT3G02410 | 2.993035931 | 9.3206E-10 | 3.9236E-09 |
| AT3G02480 | 9.641217069 | 0 | 0 |
| AT3G02490 | -1.204359295 | 3.211E-11 | 1.48E-10 |
| AT3G02550 | 1.490671698 | 1.2954E-12 | 6.5116E-12 |
| AT3G02650 | -1.173465724 | 2.1573E-22 | 1.7535E-21 |
| AT3G02800 | 1.469821551 | 8.0805E-06 | 2.3894E-05 |
| AT3G02875 | 1.838672608 | 1.0174E-38 | 1.4051E-37 |
| AT3G02885 | -3.362259878 | 1.2206E-31 | 1.3706E-30 |
| AT3G02940 | 1.883068678 | 4.2516E-05 | 0.00011621 |
| AT3G02990 | 2.93498632 | 1.9795E-84 | 6.731E-83 |
| AT3G03000 | -1.846230999 | 6.618E-08 | 2.3971E-07 |
| AT3G03060 | -1.75962684 | 1.9447E-39 | 2.7389E-38 |
| AT3G03130 | -1.425525649 | 3.693E-16 | 2.2657E-15 |
| AT3G03150 | 1.427015264 | 2.4988E-87 | 9.0068E-86 |
| AT3G03170 | 4.007525531 | 9.786E-79 | 3.0213E-77 |
| AT3G03230 | 4.229266434 | 0.00561643 | 0.01121615 |
| AT3G03270 | 1.683912529 | 9.6351E-79 | 2.9788E-77 |
| AT3G03310 | 2.331966591 | 1.8057E-77 | 5.4567E-76 |
| AT3G03341 | 8.856227977 | 0 | 0 |
| AT3G03440 | 2.214118559 | 4.9811E-63 | 1.1976E-61 |
| AT3G03470 | 4.964215476 | 9.643E-291 | 1.897E-288 |
| AT3G03490 | 1.284042472 | 2.9437E-23 | 2.4838E-22 |
| AT3G03630 | -1.411493525 | 2.6364E-26 | 2.4835E-25 |
| AT3G03640 | 1.674991479 | 2.7567E-68 | 7.2629E-67 |
| AT3G03780 | -2.531569223 | 0 | 0 |
| AT3G03790 | 1.023952176 | 1.4128E-30 | 1.5336E-29 |
| AT3G03800 | 5.643024464 | 0.00825557 | 0.01598432 |
| AT3G03820 | -3.109417927 | 0.00224598 | 0.00481438 |
| AT3G01335 | -1.170252605 | 0.00793466 | 0.01540348 |
| AT3G03870 | 2.609477897 | 1.0923E-83 | 3.6278E-82 |
| AT3G03900 | 1.817458833 | 1.0074E-30 | 1.0992E-29 |
| AT3G03910 | -2.219499245 | 0.00397224 | 0.00815255 |
| AT3G04000 | 1.793623935 | 1.2016E-27 | 1.1935E-26 |
| AT3G04010 | 2.146747206 | 2.4296E-75 | 7.116E-74 |
| AT3G04040 | 2.341002263 | 3.8497E-19 | 2.742E-18 |
| AT3G04050 | 2.072591758 | 0.00013808 | 0.00035447 |
| AT3G04060 | 1.193699497 | 1.6383E-11 | 7.693E-11 |
| AT3G04070 | 1.44059976 | 1.9303E-21 | 1.5021E-20 |
| AT3G04140 | -1.873011612 | 7.0449E-19 | 4.9641E-18 |
| AT3G04210 | -4.483534497 | 0.0003643 | 0.00087966 |
| AT3G04220 | -2.160480917 | 0.00120258 | 0.00268606 |
| AT3G04240 | 1.974720926 | 1.847E-211 | 2.139E-209 |
| AT3G04290 | -2.391894149 | 1.428E-135 | 8.807E-134 |
| AT3G04320 | -4.54185711 | 0.00702523 | 0.01378344 |
| AT3G04370 | -1.852478852 | 0.00096581 | 0.00218676 |
| AT3G04485 | 1.344491224 | 5.6192E-07 | 1.8608E-06 |
| AT3G04530 | -1.661430251 | 0.00194119 | 0.00419732 |
| AT3G04550 | -1.525741326 | 1.1143E-84 | 3.8141E-83 |
| AT3G04570 | -1.315165014 | 1.0983E-13 | 5.9156E-13 |
| AT3G04620 | 2.337396688 | 5.309E-48 | 9.2536E-47 |
| AT3G04640 | 1.001598928 | 2.5304E-05 | 7.099E-05 |
| AT3G04650 | -1.609845342 | 1.9449E-41 | 2.8797E-40 |
| AT3G04760 | -1.032238517 | 7.9676E-25 | 7.115E-24 |
| AT3G04770 | -1.742875107 | 2.6677E-23 | 2.2559E-22 |
| AT3G04790 | -1.314758746 | 5.7146E-68 | 1.497E-66 |
| AT3G04950 | -1.134626952 | 7.5976E-06 | 2.2501E-05 |
| AT3G05030 | 1.12701402 | 3.5841E-22 | 2.8814E-21 |
| AT3G05060 | -1.123892062 | 4.7106E-52 | 9.1159E-51 |
| AT3G05130 | -1.62269564 | 1.7421E-25 | 1.5906E-24 |
| AT3G05140 | -1.537220232 | 0.008288 | 0.0160403 |
| AT3G05150 | -2.821810566 | 3.7547E-05 | 0.00010329 |
| AT3G05155 | -2.7727809 | 0.00465869 | 0.0094474 |
| AT3G05160 | 1.002016416 | 1.7361E-16 | 1.0811E-15 |
| AT3G05170 | 2.199633554 | 0.00026392 | 0.00064979 |
| AT3G05200 | 1.041453626 | 1.1114E-15 | 6.6506E-15 |
| AT3G05260 | 2.247511884 | 0.00057232 | 0.00133957 |
| AT3G05345 | 2.18891923 | 3.4298E-51 | 6.5049E-50 |
| AT3G05410 | -1.125545875 | 6.6042E-15 | 3.8099E-14 |
| AT3G05415 | -1.420787937 | 0.00117334 | 0.00262537 |
| AT3G05500 | 1.116199909 | 2.5999E-31 | 2.8923E-30 |
| AT3G05580 | 1.369892756 | 2.8128E-22 | 2.2725E-21 |
| AT3G05625 | -1.559209936 | 5.5898E-27 | 5.3972E-26 |
| AT3G05630 | 3.387037532 | 1.3604E-82 | 4.4596E-81 |
| AT3G05640 | 4.569751549 | 0 | 0 |
| AT3G05650 | 1.439467324 | 1.5746E-08 | 6.0046E-08 |
| AT3G05660 | 1.779772503 | 1.4035E-16 | 8.7707E-16 |
| AT3G05700 | 1.147256777 | 4.3085E-12 | 2.095E-11 |
| AT3G05820 | 1.229381533 | 0.00618099 | 0.0122503 |
| AT3G05830 | 1.281080536 | 7.1183E-13 | 3.6473E-12 |
| AT3G05880 | 2.070849555 | 7.691E-300 | 1.595E-297 |
| AT3G01795 | 2.850214556 | 1.3344E-27 | 1.3231E-26 |
| AT3G05890 | 1.776205928 | 3.7831E-83 | 1.2509E-81 |
| AT3G05920 | -2.079655124 | 9.1261E-07 | 2.9636E-06 |
| AT3G05936 | 2.508481116 | 9.6632E-16 | 5.7989E-15 |
| AT3G06020 | -2.99507731 | 6.1914E-06 | 1.8519E-05 |
| AT3G06070 | -1.280258745 | 5.6337E-05 | 0.00015167 |
| AT3G06145 | -3.637021897 | 1.5489E-21 | 1.2115E-20 |
| AT3G06160 | -1.155651492 | 0.00012776 | 0.00032945 |
| AT3G06370 | -1.14167588 | 4.7514E-05 | 0.00012918 |
| AT3G06420 | 2.665114636 | 3.197E-138 | 2.021E-136 |
| AT3G06455 | 1.057793511 | 0.00022778 | 0.00056534 |
| AT3G06460 | -2.27601163 | 0.00017033 | 0.00043075 |
| AT3G06470 | -1.09384055 | 4.287E-08 | 1.5756E-07 |
| AT3G06490 | 1.342304812 | 2.5272E-05 | 7.0913E-05 |
| AT3G06500 | 2.874615785 | 1.069E-300 | 2.237E-298 |
| AT3G06620 | 1.014282219 | 7.9895E-25 | 7.1318E-24 |
| AT3G06680 | -1.264370524 | 1.3377E-30 | 1.4541E-29 |
| AT3G06730 | -1.077474231 | 2.9175E-20 | 2.1704E-19 |
| AT3G06740 | -1.076852116 | 3.1114E-07 | 1.0578E-06 |
| AT3G06750 | -1.861229551 | 3.2918E-16 | 2.0244E-15 |
| AT3G06760 | 1.316555542 | 3.9049E-25 | 3.5173E-24 |
| AT3G06770 | -2.157308612 | 3.9926E-26 | 3.735E-25 |
| AT3G06780 | 2.714864011 | 5.8095E-37 | 7.6485E-36 |
| AT3G06840 | -2.077143307 | 5.4428E-10 | 2.3321E-09 |
| AT3G06868 | -2.437816203 | 6.9076E-05 | 0.00018367 |
| AT3G06880 | -1.800132411 | 2.3596E-54 | 4.7516E-53 |
| AT3G06890 | -1.833336864 | 0.00013315 | 0.00034268 |
| AT3G06980 | -1.587632879 | 1.8586E-91 | 7.1151E-90 |
| AT3G06990 | -4.607632337 | 2.0075E-06 | 6.295E-06 |
| AT3G07000 | -3.70252287 | 1.6776E-05 | 4.8006E-05 |
| AT3G07010 | -1.541204959 | 2.7587E-19 | 1.9768E-18 |
| AT3G07050 | -1.285719724 | 1.148E-49 | 2.0872E-48 |
| AT3G07070 | -8.046835947 | 4.5844E-07 | 1.5332E-06 |
| AT3G07130 | -1.251679541 | 1.103E-08 | 4.2598E-08 |
| AT3G07195 | -1.616930925 | 3.9951E-06 | 1.217E-05 |
| AT3G07255 | 10.46851976 | 1.1487E-12 | 5.7997E-12 |
| AT3G07270 | -1.004383725 | 1.2901E-17 | 8.5394E-17 |
| AT3G07273 | 1.608325244 | 2.266E-10 | 9.9408E-10 |
| AT3G07290 | -1.340084256 | 7.232E-06 | 2.1471E-05 |
| AT3G07310 | 1.197865452 | 1.1839E-31 | 1.3313E-30 |
| AT3G07350 | 3.389132601 | 8.5755E-22 | 6.7796E-21 |
| AT3G07360 | 1.61981566 | 8.4602E-66 | 2.1424E-64 |
| AT3G07370 | 1.000526434 | 5.6114E-14 | 3.0798E-13 |
| AT3G07425 | -3.370395602 | 0.00644961 | 0.01274285 |
| AT3G07430 | -1.857943769 | 7.977E-113 | 3.923E-111 |
| AT3G07460 | -1.72324509 | 6.5511E-59 | 1.4554E-57 |
| AT3G07470 | -1.555575398 | 8.5413E-41 | 1.2476E-39 |
| AT3G07700 | 1.796529314 | 1.019E-106 | 4.685E-105 |
| AT3G07730 | 1.229966668 | 7.3452E-06 | 2.1782E-05 |
| AT3G07750 | -1.181608745 | 6.5632E-14 | 3.585E-13 |
| AT3G07770 | -1.209767277 | 2.8984E-61 | 6.7341E-60 |
| AT3G07870 | 1.537755058 | 1.4766E-27 | 1.4604E-26 |
| AT3G07900 | -4.784569547 | 2.0479E-09 | 8.3976E-09 |
| AT3G07990 | -1.169335837 | 1.4792E-06 | 4.694E-06 |
| AT3G08040 | -2.059074773 | 1.5795E-21 | 1.2346E-20 |
| AT3G08490 | -5.009247339 | 0.00017032 | 0.00043075 |
| AT3G08505 | 1.132460244 | 8.122E-11 | 3.6565E-10 |
| AT3G08600 | -1.697535793 | 6.442E-15 | 3.7201E-14 |
| AT3G08660 | -1.755413178 | 0.00961601 | 0.01837666 |
| AT3G08680 | -1.330926738 | 9.4614E-44 | 1.4929E-42 |
| AT3G08720 | 1.289070141 | 2.2072E-21 | 1.7141E-20 |
| AT3G08820 | -1.549493297 | 9.6097E-07 | 3.114E-06 |
| AT3G08860 | 4.335738993 | 3.471E-40 | 4.9996E-39 |
| AT3G08870 | 1.425671569 | 4.9684E-05 | 0.00013468 |
| AT3G08970 | 1.363212433 | 5.5877E-13 | 2.8746E-12 |
| AT3G08980 | -1.336204334 | 2.8643E-06 | 8.8494E-06 |
| AT3G09035 | -1.432465554 | 1.8859E-14 | 1.0611E-13 |
| AT3G09070 | -1.212208459 | 7.4806E-19 | 5.2597E-18 |
| AT3G09085 | 1.284266058 | 0.00010318 | 0.00026893 |
| AT3G09162 | -1.040988977 | 0.00534159 | 0.01071889 |
| AT3G09220 | -3.539936888 | 4.768E-144 | 3.257E-142 |
| AT3G09260 | -1.365643079 | 3.5749E-13 | 1.8575E-12 |
| AT3G09270 | -1.236780831 | 6.4477E-35 | 7.9348E-34 |
| AT3G09290 | -2.078116512 | 0.00768572 | 0.01496862 |
| AT3G09340 | -4.936711015 | 0.0001532 | 0.00039017 |
| AT3G09370 | 1.317859901 | 3.4267E-14 | 1.8995E-13 |
| AT3G09390 | 1.685765078 | 4.462E-191 | 4.628E-189 |
| AT3G09405 | -2.098301702 | 5.463E-11 | 2.4892E-10 |
| AT3G09450 | 3.887191693 | 1.5347E-05 | 4.4112E-05 |
| AT3G09480 | 1.32227986 | 3.5112E-08 | 1.3024E-07 |
| AT3G09560 | 1.066749263 | 3.511E-24 | 3.0716E-23 |
| AT3G09580 | -1.533531583 | 9.0281E-21 | 6.8411E-20 |
| AT3G09640 | 3.651841236 | 1.3849E-23 | 1.1839E-22 |
| AT3G09700 | -1.092542817 | 1.0034E-05 | 2.9403E-05 |
| AT3G09760 | 1.048400867 | 2.6591E-14 | 1.4852E-13 |
| AT3G09770 | 1.856342999 | 1.88E-102 | 8.266E-101 |
| AT3G09780 | -1.414254294 | 6.1476E-05 | 0.00016463 |
| AT3G09910 | 3.773465675 | 4.2698E-50 | 7.8501E-49 |
| AT3G09920 | 1.045518184 | 3.3457E-25 | 3.022E-24 |
| AT3G09925 | -3.6399245 | 6.4222E-20 | 4.7161E-19 |
| AT3G09940 | -1.652196536 | 2.8301E-14 | 1.5784E-13 |
| AT3G09950 | 2.877980335 | 1.0416E-09 | 4.3684E-09 |
| AT3G09960 | -3.247503949 | 9.2127E-07 | 2.9909E-06 |
| AT3G10020 | 2.544211712 | 3.707E-211 | 4.272E-209 |
| AT3G10050 | -1.079647535 | 4.3091E-35 | 5.3432E-34 |
| AT3G10110 | -1.505432298 | 4.8759E-09 | 1.9374E-08 |
| AT3G10130 | 1.06825409 | 6.2434E-25 | 5.5973E-24 |
| AT3G10210 | 1.008145909 | 6.797E-10 | 2.8944E-09 |
| AT3G10250 | 1.648850122 | 2.857E-33 | 3.3653E-32 |
| AT3G10300 | 1.339499644 | 1.7938E-37 | 2.4117E-36 |
| AT3G10340 | 2.13129676 | 1.4578E-87 | 5.2795E-86 |
| AT3G10420 | 2.406442273 | 7.126E-165 | 5.934E-163 |
| AT3G10450 | 1.788108073 | 5.0088E-33 | 5.8696E-32 |
| AT3G10500 | 1.147384204 | 3.5648E-33 | 4.1925E-32 |
| AT3G10510 | 5.849210362 | 0.00407171 | 0.00833859 |
| AT3G10520 | -1.869597602 | 4.3167E-62 | 1.0227E-60 |
| AT3G10530 | -1.634107474 | 1.6897E-36 | 2.1992E-35 |
| AT3G10550 | 1.326848455 | 1.7352E-29 | 1.8337E-28 |
| AT3G10570 | -1.542330868 | 4.7064E-06 | 1.4255E-05 |
| AT3G10600 | -6.04592899 | 0.00260807 | 0.00552308 |
| AT3G10610 | -1.315210267 | 6.6788E-37 | 8.7728E-36 |
| AT3G10640 | 1.285447294 | 9.0902E-26 | 8.4138E-25 |
| AT3G10720 | -1.714167759 | 7.7517E-44 | 1.2248E-42 |
| AT3G10820 | 1.459783141 | 8.1397E-16 | 4.9014E-15 |
| AT3G10910 | 2.326482627 | 1.9964E-66 | 5.1066E-65 |
| AT3G10930 | 1.376059065 | 0.00569878 | 0.01136569 |
| AT3G10985 | 1.26765515 | 1.5471E-60 | 3.5229E-59 |
| AT3G11020 | 1.974914062 | 1.7091E-05 | 4.8848E-05 |
| AT3G11100 | 1.424047986 | 7.3762E-10 | 3.1322E-09 |
| AT3G11210 | 1.280441196 | 3.9607E-23 | 3.3235E-22 |
| AT3G11230 | 1.169890185 | 2.4001E-14 | 1.3435E-13 |
| AT3G11250 | -1.048979169 | 7.1568E-28 | 7.1712E-27 |
| AT3G11260 | 1.227519272 | 0.00087649 | 0.00200001 |
| AT3G11340 | 1.597092487 | 6.0052E-54 | 1.2008E-52 |
| AT3G11385 | -3.681598142 | 0.00121844 | 0.00272016 |
| AT3G11410 | 3.688166766 | 0 | 0 |
| AT3G11420 | 3.124061852 | 1.668E-253 | 2.589E-251 |
| AT3G11430 | 1.338932577 | 8.3982E-13 | 4.2895E-12 |
| AT3G11480 | 5.955849091 | 0.00348851 | 0.00722594 |
| AT3G11550 | -2.91325619 | 6.2964E-17 | 4.0151E-16 |
| AT3G11660 | 1.005921641 | 7.7833E-20 | 5.6918E-19 |
| AT3G11690 | 2.019110719 | 1.8428E-48 | 3.2517E-47 |
| AT3G11880 | 1.059114078 | 1.067E-16 | 6.7155E-16 |
| AT3G11964 | -1.012520553 | 9.5985E-39 | 1.3265E-37 |
| AT3G12090 | -1.17447697 | 1.3246E-08 | 5.0787E-08 |
| AT3G12110 | -1.296810693 | 3.0637E-14 | 1.7036E-13 |
| AT3G12120 | 1.013583456 | 4.9653E-82 | 1.6138E-80 |
| AT3G12170 | -1.497833966 | 5.3127E-05 | 0.00014345 |
| AT3G12220 | -3.594596223 | 0.00014873 | 0.00037946 |
| AT3G12270 | -1.223011692 | 2.9374E-29 | 3.0828E-28 |
| AT3G12300 | 1.019882719 | 2.637E-09 | 1.0698E-08 |
| AT3G12320 | 3.679604209 | 2.9606E-14 | 1.6483E-13 |
| AT3G12360 | 1.00741302 | 1.893E-26 | 1.7914E-25 |
| AT3G12520 | 1.02733428 | 2.2646E-13 | 1.1925E-12 |
| AT3G12540 | -8.083301984 | 3.7745E-07 | 1.2728E-06 |
| AT3G12580 | 2.157851938 | 1.137E-225 | 1.441E-223 |
| AT3G12710 | 1.039384202 | 7.2919E-10 | 3.0993E-09 |
| AT3G12720 | 1.267883175 | 0.00298281 | 0.00625123 |
| AT3G12750 | -1.062701637 | 0.00023221 | 0.00057575 |
| AT3G12870 | -1.028183574 | 9.2873E-06 | 2.7286E-05 |
| AT3G12900 | -5.891984753 | 5.9177E-07 | 1.9551E-06 |
| AT3G12930 | -1.676493355 | 2.6004E-44 | 4.1577E-43 |
| AT3G12955 | 1.533065602 | 3.589E-08 | 1.3296E-07 |
| AT3G12960 | 6.174841968 | 1.1591E-13 | 6.227E-13 |
| AT3G12977 | -2.397683004 | 0.00031451 | 0.00076544 |
| AT3G13000 | -1.142537317 | 5.1416E-12 | 2.4885E-11 |
| AT3G13020 | 1.028611443 | 0.00899281 | 0.01727686 |
| AT3G13040 | 1.375452948 | 8.0864E-52 | 1.5583E-50 |
| AT3G13065 | 1.756116502 | 4.1498E-06 | 1.2611E-05 |
| AT3G13070 | -1.270254067 | 6.4308E-40 | 9.1819E-39 |
| AT3G13110 | -1.176274155 | 1.7279E-29 | 1.8269E-28 |
| AT3G13120 | -1.0502481 | 3.7101E-60 | 8.406E-59 |
| AT3G13130 | 2.843474019 | 2.0989E-13 | 1.1085E-12 |
| AT3G13140 | 5.773904663 | 0.00548343 | 0.01097231 |
| AT3G13150 | -1.311892747 | 1.2351E-11 | 5.8489E-11 |
| AT3G13200 | 1.112367657 | 1.5163E-46 | 2.5532E-45 |
| AT3G13210 | 1.595134682 | 0.00849226 | 0.01640223 |
| AT3G13230 | -1.215000471 | 2.1457E-25 | 1.9528E-24 |
| AT3G13275 | 1.343134369 | 2.3136E-07 | 7.9691E-07 |
| AT3G13404 | -7.19776703 | 2.4588E-05 | 6.9115E-05 |
| AT3G13420 | 1.18762746 | 0.00011069 | 0.00028751 |
| AT3G13470 | -2.575177577 | 1.237E-302 | 2.614E-300 |
| AT3G13480 | 1.116964191 | 1.0057E-10 | 4.507E-10 |
| AT3G13510 | -1.35416448 | 1.369E-30 | 1.4867E-29 |
| AT3G13520 | 1.229247484 | 2.7749E-61 | 6.4537E-60 |
| AT3G13550 | 1.002713179 | 3.4705E-10 | 1.5036E-09 |
| AT3G13560 | -1.516651548 | 8.4679E-26 | 7.8411E-25 |
| AT3G13590 | 1.496956943 | 0.00134307 | 0.00297566 |
| AT3G13610 | -4.037909393 | 3.254E-184 | 3.146E-182 |
| AT3G13672 | 5.049021579 | 6.8525E-84 | 2.2959E-82 |
| AT3G13720 | 1.254797361 | 1.4367E-31 | 1.6092E-30 |
| AT3G13730 | -1.959651962 | 1.8159E-16 | 1.1292E-15 |
| AT3G13750 | -2.292231669 | 4.612E-199 | 4.964E-197 |
| AT3G13760 | -2.995620414 | 7.7503E-05 | 0.00020462 |
| AT3G13782 | -1.25810628 | 0.00565118 | 0.01128017 |
| AT3G13784 | 1.352739589 | 1.9527E-19 | 1.4112E-18 |
| AT3G13840 | -3.305547777 | 0.00275295 | 0.00580618 |
| AT3G13940 | -1.196625125 | 2.1077E-17 | 1.3791E-16 |
| AT3G13960 | -1.28114514 | 1.4412E-06 | 4.5786E-06 |
| AT3G14020 | -1.083360307 | 0.00032711 | 0.00079404 |
| AT3G14050 | 2.287504757 | 1.914E-113 | 9.451E-112 |
| AT3G14060 | 2.060294319 | 1.2481E-16 | 7.8276E-16 |
| AT3G14067 | 1.940183572 | 1.048E-279 | 1.928E-277 |
| AT3G14070 | 1.414190586 | 1.6829E-08 | 6.4091E-08 |
| AT3G14130 | 1.30895891 | 2.3669E-12 | 1.1696E-11 |
| AT3G14210 | -2.418147687 | 1.012E-299 | 2.061E-297 |
| AT3G14240 | -1.941389741 | 4.074E-115 | 2.079E-113 |
| AT3G14280 | 2.626703287 | 8.1901E-54 | 1.632E-52 |
| AT3G14310 | -2.282782392 | 1.449E-280 | 2.711E-278 |
| AT3G14330 | -1.13281939 | 8.2314E-15 | 4.7188E-14 |
| AT3G14360 | 2.335911809 | 1.7064E-32 | 1.9713E-31 |
| AT3G14390 | -1.242377409 | 1.8943E-43 | 2.9684E-42 |
| AT3G14430 | 1.655715237 | 3.3696E-34 | 4.0548E-33 |
| AT3G14440 | 6.037084621 | 0 | 0 |
| AT3G14540 | -1.928551822 | 4.0932E-06 | 1.2444E-05 |
| AT3G14560 | 2.713440972 | 1.7995E-88 | 6.6221E-87 |
| AT3G14580 | -1.551091109 | 2.4577E-05 | 6.9093E-05 |
| AT3G14590 | 2.693453419 | 1.6836E-86 | 5.9556E-85 |
| AT3G14595 | 2.84398013 | 2.435E-150 | 1.775E-148 |
| AT3G14620 | -1.599893466 | 2.3395E-52 | 4.5467E-51 |
| AT3G14690 | 1.079317458 | 1.9132E-55 | 3.9504E-54 |
| AT3G14740 | -1.850949515 | 2.618E-13 | 1.3741E-12 |
| AT3G14780 | 1.015317403 | 0.00285258 | 0.0059958 |
| AT3G14810 | 1.777375964 | 4.6276E-51 | 8.7403E-50 |
| AT3G14840 | -1.926971796 | 1.1795E-88 | 4.3615E-87 |
| AT3G14880 | 4.206874451 | 0.00014844 | 0.00037883 |
| AT3G14900 | -2.656280329 | 5.7921E-81 | 1.8457E-79 |
| AT3G14940 | -1.831671725 | 5.553E-121 | 2.988E-119 |
| AT3G15030 | -1.662045657 | 1.8575E-62 | 4.4239E-61 |
| AT3G15080 | -1.163582855 | 5.0927E-13 | 2.6247E-12 |
| AT3G15140 | -1.079653114 | 9.9524E-09 | 3.8585E-08 |
| AT3G15180 | 1.068351963 | 2.6261E-22 | 2.127E-21 |
| AT3G15210 | 1.897263924 | 1.6579E-98 | 6.992E-97 |
| AT3G00330 | 4.460334085 | 0.00227 | 0.00486262 |
| AT3G15240 | -1.116074506 | 8.4974E-09 | 3.3113E-08 |
| AT3G15280 | 6.575952669 | 0.00040288 | 0.00096637 |
| AT3G15290 | 1.105099792 | 2.2384E-19 | 1.6106E-18 |
| AT3G15350 | 2.22542101 | 2.16E-118 | 1.13E-116 |
| AT3G15358 | 1.280823817 | 0.00247486 | 0.00526053 |
| AT3G15370 | -3.705717352 | 0.00306353 | 0.00641085 |
| AT3G15450 | 1.151271325 | 1.1687E-26 | 1.1171E-25 |
| AT3G15500 | 3.236437494 | 3.0314E-35 | 3.7774E-34 |
| AT3G15530 | -1.027600617 | 1.7375E-11 | 8.1385E-11 |
| AT3G15534 | 4.443522403 | 3.1816E-30 | 3.4161E-29 |
| AT3G15570 | -1.379770412 | 3.1925E-10 | 1.3877E-09 |
| AT3G15580 | 1.348059649 | 4.2958E-33 | 5.0392E-32 |
| AT3G15650 | -1.107052244 | 2.9404E-05 | 8.1865E-05 |
| AT3G15670 | 8.689997948 | 1.988E-290 | 3.878E-288 |
| AT3G15680 | -1.43097779 | 1.479E-22 | 1.2121E-21 |
| AT3G15700 | -7.127241023 | 3.3812E-05 | 9.3431E-05 |
| AT3G15720 | -2.09950809 | 3.0911E-11 | 1.4268E-10 |
| AT3G15760 | 2.225512187 | 1.6028E-19 | 1.1631E-18 |
| AT3G15770 | 1.132465768 | 1.4302E-05 | 4.1237E-05 |
| AT3G15780 | 1.749642717 | 6.0076E-18 | 4.0386E-17 |
| AT3G15790 | 1.262889326 | 2.6113E-41 | 3.8538E-40 |
| AT3G15820 | -1.028796211 | 8.5111E-10 | 3.5981E-09 |
| AT3G15840 | 1.167278999 | 1.998E-87 | 7.2132E-86 |
| AT3G15850 | -1.653680221 | 4.7474E-81 | 1.5149E-79 |
| AT3G15880 | 1.038477267 | 2.2311E-48 | 3.931E-47 |
| AT3G03165 | 1.581437897 | 0.00328655 | 0.00684053 |
| AT3G15950 | -1.712857502 | 1.633E-26 | 1.5525E-25 |
| AT3G15990 | 1.474464463 | 3.3112E-16 | 2.0358E-15 |
| AT3G16000 | -1.243221982 | 5.1139E-49 | 9.1441E-48 |
| AT3G16120 | 1.650237081 | 3.3541E-05 | 9.2727E-05 |
| AT3G16180 | -1.893740151 | 7.2274E-23 | 5.9833E-22 |
| AT3G16190 | 1.11308912 | 2.1201E-23 | 1.8022E-22 |
| AT3G16240 | -1.370255856 | 1.4929E-61 | 3.5042E-60 |
| AT3G16330 | 2.563325109 | 4.7319E-86 | 1.6635E-84 |
| AT3G16360 | 1.720800712 | 2.7017E-18 | 1.8522E-17 |
| AT3G16410 | -1.085469578 | 2.7753E-09 | 1.1235E-08 |
| AT3G16430 | -1.316071986 | 2.8059E-34 | 3.3819E-33 |
| AT3G16450 | -1.271181337 | 2.5077E-42 | 3.8246E-41 |
| AT3G16460 | -1.55953659 | 2.9863E-97 | 1.2388E-95 |
| AT3G16490 | -1.195650761 | 0.00505709 | 0.01019191 |
| AT3G16510 | 1.440168481 | 1.0827E-06 | 3.4843E-06 |
| AT3G16530 | -3.099588428 | 1.689E-113 | 8.36E-112 |
| AT3G16570 | 1.354801106 | 1.6502E-44 | 2.6609E-43 |
| AT3G16660 | -1.325010221 | 1.761E-33 | 2.0818E-32 |
| AT3G16690 | -1.87043414 | 2.6252E-09 | 1.0652E-08 |
| AT3G16700 | 1.061927394 | 0.00026841 | 0.00066021 |
| AT3G16780 | -1.256182008 | 8.3607E-66 | 2.1219E-64 |
| AT3G16800 | 4.067223943 | 0 | 0 |
| AT3G16810 | -1.608381736 | 1.4376E-55 | 2.9845E-54 |
| AT3G16870 | -1.370103678 | 3.3235E-16 | 2.0422E-15 |
| AT3G16940 | 1.195695207 | 1.4269E-38 | 1.9671E-37 |
| AT3G16990 | 2.482997161 | 8.3166E-63 | 1.9911E-61 |
| AT3G17000 | 3.12746817 | 2.073E-294 | 4.113E-292 |
| AT3G17050 | -2.616270635 | 4.6811E-21 | 3.5877E-20 |
| AT3G17100 | 1.188570443 | 1.5538E-23 | 1.3248E-22 |
| AT3G17120 | -1.264597529 | 1.7708E-13 | 9.3958E-13 |
| AT3G17170 | -2.205648439 | 8.2643E-89 | 3.061E-87 |
| AT3G17185 | -1.497137353 | 8.8736E-06 | 2.6127E-05 |
| AT3G17330 | -1.114904649 | 1.5693E-09 | 6.4898E-09 |
| AT3G17350 | -1.130868732 | 1.3979E-06 | 4.4514E-06 |
| AT3G17390 | -1.935503444 | 8.652E-169 | 7.592E-167 |
| AT3G17510 | 1.242145255 | 1.1572E-35 | 1.4693E-34 |
| AT3G17520 | 11.16331138 | 1.311E-253 | 2.049E-251 |
| AT3G17640 | -1.858893921 | 1.6786E-17 | 1.1034E-16 |
| AT3G17660 | 1.046006042 | 0.0024081 | 0.00513056 |
| AT3G17770 | 1.809552339 | 3.2418E-92 | 1.2494E-90 |
| AT3G17790 | 2.449866301 | 1.204E-142 | 8.076E-141 |
| AT3G17800 | 2.072637099 | 5.8207E-93 | 2.274E-91 |
| AT3G17810 | 1.733304359 | 5.261E-126 | 2.986E-124 |
| AT3G17830 | -1.444304127 | 3.3024E-13 | 1.7203E-12 |
| AT3G17840 | -1.353072093 | 1.2367E-35 | 1.5658E-34 |
| AT3G17860 | 1.23567341 | 8.4675E-18 | 5.6506E-17 |
| AT3G17950 | 1.596328807 | 2.4885E-23 | 2.1083E-22 |
| AT3G18010 | -1.016595095 | 0.00061038 | 0.00142325 |
| AT3G18050 | -1.706097984 | 9.0472E-64 | 2.2054E-62 |
| AT3G18080 | -1.805228343 | 1.234E-141 | 8.208E-140 |
| AT3G18090 | -1.146090268 | 9.1789E-08 | 3.2825E-07 |
| AT3G18110 | -1.139032994 | 3.3645E-24 | 2.9456E-23 |
| AT3G18130 | -1.506998523 | 2.3453E-96 | 9.607E-95 |
| AT3G18200 | -4.454855735 | 3.2035E-21 | 2.4735E-20 |
| AT3G18230 | -1.03045177 | 1.7756E-14 | 1.0006E-13 |
| AT3G18250 | -3.80393749 | 3.1755E-09 | 1.2774E-08 |
| AT3G18280 | 1.717549105 | 6.131E-87 | 2.196E-85 |
| AT3G18290 | 1.505132922 | 1.2863E-05 | 3.7276E-05 |
| AT3G18350 | 1.118456987 | 4.7566E-10 | 2.0458E-09 |
| AT3G18510 | -1.126669035 | 0.00011173 | 0.00028997 |
| AT3G18580 | -1.041532012 | 6.618E-08 | 2.3971E-07 |
| AT3G18600 | -1.328478409 | 3.0997E-49 | 5.5818E-48 |
| AT3G18610 | 1.504055288 | 9.8457E-13 | 5.0053E-12 |
| AT3G18630 | -1.62531646 | 0.00011564 | 0.00029944 |
| AT3G18660 | 1.129450955 | 1.3374E-05 | 3.869E-05 |
| AT3G18680 | -1.297709815 | 1.1839E-60 | 2.7038E-59 |
| AT3G18710 | -1.755592971 | 1.0519E-11 | 5E-11 |
| AT3G18770 | 1.011997172 | 1.3225E-05 | 3.8273E-05 |
| AT3G18890 | -1.268208936 | 1.957E-52 | 3.8098E-51 |
| AT3G18900 | -1.343542073 | 1.2717E-05 | 3.6878E-05 |
| AT3G18950 | 1.396346206 | 3.5439E-11 | 1.6309E-10 |
| AT3G19020 | -1.432709458 | 3.3789E-08 | 1.255E-07 |
| AT3G19030 | 1.057860899 | 2.175E-39 | 3.0594E-38 |
| AT3G19100 | 1.942930516 | 1.77E-182 | 1.682E-180 |
| AT3G19200 | 2.559760954 | 2.8389E-14 | 1.5829E-13 |
| AT3G19240 | 1.088891191 | 9.8583E-22 | 7.7748E-21 |
| AT3G19260 | 1.019348839 | 3.963E-12 | 1.9308E-11 |
| AT3G19290 | 1.778034025 | 2.0956E-75 | 6.1535E-74 |
| AT3G19360 | -1.258188084 | 5.567E-10 | 2.3822E-09 |
| AT3G19370 | -1.529966049 | 1.1965E-49 | 2.1719E-48 |
| AT3G19400 | -1.223344124 | 9.0798E-10 | 3.8272E-09 |
| AT3G19430 | -5.311116714 | 2.2827E-13 | 1.2014E-12 |
| AT3G19440 | -2.159954982 | 1.2697E-22 | 1.0435E-21 |
| AT3G19450 | -2.012652893 | 4.181E-152 | 3.128E-150 |
| AT3G19540 | -1.060680386 | 1.037E-16 | 6.5359E-16 |
| AT3G19580 | 3.696138463 | 2.973E-135 | 1.823E-133 |
| AT3G19620 | 1.008834489 | 7.9532E-10 | 3.3704E-09 |
| AT3G19710 | -2.839838291 | 2.515E-216 | 2.973E-214 |
| AT3G19860 | 1.02203434 | 5.245E-20 | 3.864E-19 |
| AT3G19910 | 1.490164672 | 8.924E-61 | 2.0443E-59 |
| AT3G19930 | -1.344910239 | 8.1919E-28 | 8.1869E-27 |
| AT3G19950 | 1.028469445 | 1.7678E-18 | 1.2245E-17 |
| AT3G20015 | -2.764842313 | 1.8014E-57 | 3.9106E-56 |
| AT3G20060 | 1.078608853 | 6.7225E-35 | 8.2551E-34 |
| AT3G20110 | -4.855388536 | 5.1151E-28 | 5.1549E-27 |
| AT3G20240 | -1.450243221 | 3.6306E-19 | 2.5895E-18 |
| AT3G20250 | 2.289725412 | 1.303E-184 | 1.27E-182 |
| AT3G20300 | 2.729199921 | 3.222E-140 | 2.088E-138 |
| AT3G20370 | -2.247610865 | 3.497E-117 | 1.813E-115 |
| AT3G20380 | -1.843441351 | 8.5337E-07 | 2.7787E-06 |
| AT3G20395 | -3.435343388 | 0.00372803 | 0.00768371 |
| AT3G20440 | -2.25853303 | 1.7106E-55 | 3.5417E-54 |
| AT3G20460 | -2.053634764 | 0.00754557 | 0.01471701 |
| AT3G20540 | -1.527871389 | 7.9874E-38 | 1.0841E-36 |
| AT3G20557 | -6.253362302 | 0.00135689 | 0.00300397 |
| AT3G20590 | -5.326818696 | 3.0197E-05 | 8.394E-05 |
| AT3G20640 | -1.15287075 | 5.0335E-06 | 1.5199E-05 |
| AT3G20660 | 1.219856773 | 3.3824E-09 | 1.3592E-08 |
| AT3G20730 | -1.796544108 | 0.00187392 | 0.00406073 |
| AT3G20840 | -1.26413845 | 0.00055675 | 0.00130579 |
| AT3G20910 | 1.433028916 | 5.0092E-17 | 3.2131E-16 |
| AT3G20960 | -1.178701512 | 0.00019231 | 0.00048244 |
| AT3G21020 | -1.67042035 | 0.00884598 | 0.01701769 |
| AT3G21090 | 1.757461953 | 1.0956E-21 | 8.6258E-21 |
| AT3G21150 | 2.177007622 | 1.6294E-06 | 5.1506E-06 |
| AT3G21180 | -2.463461087 | 6.5516E-06 | 1.9548E-05 |
| AT3G21200 | -1.690467789 | 2.8733E-69 | 7.6495E-68 |
| AT3G21210 | 1.747262253 | 0.00272595 | 0.0057551 |
| AT3G21270 | 1.813307489 | 8.0225E-47 | 1.3559E-45 |
| AT3G21300 | -2.244955933 | 4.1589E-49 | 7.4717E-48 |
| AT3G21330 | 1.920509547 | 0.00332858 | 0.00691665 |
| AT3G21340 | -3.19211303 | 6.3519E-05 | 0.0001698 |
| AT3G21351 | -2.323985465 | 4.2571E-09 | 1.7019E-08 |
| AT3G21360 | 1.359463025 | 1.5182E-08 | 5.7934E-08 |
| AT3G21460 | 1.778400797 | 0.0056253 | 0.01123288 |
| AT3G21470 | -1.749197121 | 0.00083099 | 0.00190265 |
| AT3G21510 | -1.175982247 | 6.3396E-21 | 4.8376E-20 |
| AT3G21540 | -1.573125034 | 7.7292E-36 | 9.8795E-35 |
| AT3G21560 | -1.041989711 | 1.1928E-34 | 1.4523E-33 |
| AT3G21660 | 3.856271921 | 1.7914E-05 | 5.109E-05 |
| AT3G21680 | 1.367308626 | 4.7259E-07 | 1.578E-06 |
| AT3G21700 | 1.361865283 | 3.4813E-21 | 2.6807E-20 |
| AT3G21770 | -1.302876726 | 1.2435E-25 | 1.1427E-24 |
| AT3G21820 | -1.117199913 | 1.6232E-09 | 6.702E-09 |
| AT3G21865 | 1.262410468 | 3.8735E-51 | 7.3404E-50 |
| AT3G21890 | 1.52462018 | 0.00019046 | 0.000478 |
| AT3G21950 | -2.151248605 | 3.7919E-12 | 1.8482E-11 |
| AT3G22100 | 3.171208676 | 8.5369E-07 | 2.7794E-06 |
| AT3G22160 | -1.257479866 | 2.7269E-05 | 7.6284E-05 |
| AT3G22200 | 1.487216782 | 3.785E-141 | 2.489E-139 |
| AT3G22210 | -2.507761593 | 0.00011444 | 0.00029657 |
| AT3G22300 | -1.04820621 | 2.1321E-15 | 1.2602E-14 |
| AT3G22370 | 1.895423807 | 5.394E-138 | 3.4E-136 |
| AT3G22415 | 1.350767698 | 0.00148605 | 0.00327339 |
| AT3G22430 | 1.044296405 | 1.3941E-08 | 5.3334E-08 |
| AT3G22540 | -2.769275424 | 0.00133289 | 0.00295598 |
| AT3G22560 | 4.231575701 | 2.9567E-17 | 1.9175E-16 |
| AT3G22600 | 2.445548476 | 3.415E-167 | 2.919E-165 |
| AT3G22620 | 2.031973444 | 9.6947E-94 | 3.8335E-92 |
| AT3G22660 | -1.338169667 | 1.6149E-39 | 2.2773E-38 |
| AT3G22740 | -1.050745087 | 4.5423E-07 | 1.5202E-06 |
| AT3G22790 | -1.113184919 | 5.3927E-25 | 4.8422E-24 |
| AT3G22800 | -3.435407554 | 1.0861E-59 | 2.4439E-58 |
| AT3G22830 | 5.428475154 | 0 | 0 |
| AT3G22840 | 1.542606436 | 4.615E-119 | 2.443E-117 |
| AT3G22910 | 3.044363837 | 2.9879E-39 | 4.1834E-38 |
| AT3G22930 | 1.191433726 | 6.1289E-11 | 2.7812E-10 |
| AT3G22970 | -1.225631079 | 2.3693E-25 | 2.1511E-24 |
| AT3G23000 | 1.76643185 | 4.817E-127 | 2.762E-125 |
| AT3G23020 | -1.296045145 | 5.7357E-08 | 2.0882E-07 |
| AT3G23030 | 1.249517544 | 2.0389E-36 | 2.6507E-35 |
| AT3G23070 | -1.049095082 | 2.4378E-26 | 2.2993E-25 |
| AT3G23175 | -2.470069437 | 2.4613E-15 | 1.4503E-14 |
| AT3G23190 | -2.554079436 | 4.0971E-14 | 2.2623E-13 |
| AT3G23230 | -7.506357241 | 6.3318E-06 | 1.8922E-05 |
| AT3G23290 | -1.822603783 | 8.985E-12 | 4.287E-11 |
| AT3G23370 | -1.983341512 | 3.3125E-08 | 1.2315E-07 |
| AT3G23470 | -2.028280718 | 2.7038E-18 | 1.8531E-17 |
| AT3G23480 | 1.122701756 | 0.0007335 | 0.00169251 |
| AT3G23510 | -2.117657545 | 1.4286E-14 | 8.0778E-14 |
| AT3G23530 | -1.406413048 | 2.6962E-72 | 7.5205E-71 |
| AT3G23550 | -3.758221624 | 7.9376E-05 | 0.0002092 |
| AT3G23605 | 1.815726006 | 8.4926E-34 | 1.0134E-32 |
| AT3G23610 | 1.014450693 | 0.00061655 | 0.00143645 |
| AT3G23620 | -1.208486318 | 4.4264E-34 | 5.307E-33 |
| AT3G23730 | -1.572022084 | 1.3436E-22 | 1.1035E-21 |
| AT3G23740 | -2.11221956 | 1.396E-14 | 7.8995E-14 |
| AT3G23800 | -1.63215643 | 5.309E-05 | 0.00014337 |
| AT3G23805 | -1.206483593 | 3.4004E-10 | 1.4747E-09 |
| AT3G23810 | -1.065567686 | 2.5447E-84 | 8.627E-83 |
| AT3G23840 | 1.513352194 | 1.205E-32 | 1.3977E-31 |
| AT3G23910 | 1.352066782 | 3.518E-20 | 2.6069E-19 |
| AT3G23920 | 3.966850107 | 0 | 0 |
| AT3G23930 | -1.309800525 | 0.00640048 | 0.01264796 |
| AT3G23940 | -1.916088183 | 9.852E-135 | 6.01E-133 |
| AT3G24080 | -1.377243256 | 3.3011E-56 | 6.9416E-55 |
| AT3G24300 | -4.383525636 | 3.804E-101 | 1.653E-99 |
| AT3G24310 | 4.084036216 | 9.8188E-52 | 1.8889E-50 |
| AT3G24320 | -1.268166062 | 1.5706E-14 | 8.8724E-14 |
| AT3G24420 | 2.359132816 | 3.701E-121 | 1.996E-119 |
| AT3G24480 | -2.122649507 | 1.8336E-92 | 7.115E-91 |
| AT3G24500 | 2.655942913 | 1.4033E-74 | 4.0579E-73 |
| AT3G24670 | -1.194375141 | 1.0346E-09 | 4.3425E-09 |
| AT3G24740 | 1.421242445 | 5.2136E-45 | 8.4544E-44 |
| AT3G24750 | 2.505584784 | 1.5528E-11 | 7.3062E-11 |
| AT3G24770 | -1.12966299 | 7.4174E-14 | 4.0352E-13 |
| AT3G24840 | 1.264436346 | 2.4637E-22 | 1.9976E-21 |
| AT3G25130 | -2.829945619 | 1.185E-17 | 7.8505E-17 |
| AT3G25190 | -1.991406124 | 9.3979E-44 | 1.4839E-42 |
| AT3G25400 | 1.135319556 | 5.2992E-07 | 1.7593E-06 |
| AT3G25510 | -3.005013509 | 5.8271E-05 | 0.00015658 |
| AT3G25570 | 1.403526628 | 8.2007E-33 | 9.5561E-32 |
| AT3G25573 | 6.256011285 | 7.8905E-08 | 2.8405E-07 |
| AT3G25600 | -1.028314124 | 0.00031932 | 0.00077665 |
| AT3G25620 | 2.2164893 | 1.0742E-18 | 7.493E-18 |
| AT3G25640 | -1.143810262 | 4.675E-05 | 0.00012727 |
| AT3G25717 | -2.380799445 | 4.0265E-26 | 3.7651E-25 |
| AT3G25730 | -1.499199077 | 1.1376E-07 | 4.0309E-07 |
| AT3G25760 | 1.176416457 | 8.1302E-28 | 8.1288E-27 |
| AT3G25790 | -3.657256876 | 6.4587E-11 | 2.9227E-10 |
| AT3G25860 | -1.249990089 | 2.0212E-59 | 4.5301E-58 |
| AT3G25870 | 1.651380129 | 4.405E-16 | 2.6952E-15 |
| AT3G25890 | -1.648225803 | 4.3618E-31 | 4.81E-30 |
| AT3G25940 | -1.113091809 | 1.7221E-06 | 5.4331E-06 |
| AT3G25970 | -1.113821988 | 0.00736375 | 0.0143833 |
| AT3G25990 | 1.770536035 | 0.00090054 | 0.00205119 |
| AT3G26050 | -1.101631204 | 0.00108849 | 0.00244536 |
| AT3G26100 | 1.161802705 | 5.9826E-30 | 6.3874E-29 |
| AT3G26170 | 2.020796685 | 4.9972E-09 | 1.9822E-08 |
| AT3G26210 | -1.026353283 | 0.00017398 | 0.0004393 |
| AT3G26230 | 1.416841208 | 0.00927567 | 0.01777239 |
| AT3G26280 | 1.554734451 | 3.7178E-27 | 3.6188E-26 |
| AT3G26290 | 1.692955374 | 9.9935E-77 | 2.9884E-75 |
| AT3G26320 | -3.689767 | 0.00026016 | 0.00064117 |
| AT3G26330 | -2.270978435 | 4.4751E-20 | 3.3043E-19 |
| AT3G26395 | 1.274016266 | 6.9699E-11 | 3.1484E-10 |
| AT3G26470 | -2.141995799 | 1.0904E-15 | 6.5299E-15 |
| AT3G26480 | 1.342383989 | 0.00122086 | 0.00272501 |
| AT3G26490 | -1.265578809 | 0.00013395 | 0.00034456 |
| AT3G26500 | -1.689426624 | 6.4196E-07 | 2.1126E-06 |
| AT3G26510 | 1.345267031 | 5.7404E-07 | 1.8987E-06 |
| AT3G26520 | -1.577321589 | 2.248E-138 | 1.424E-136 |
| AT3G26570 | -1.619370016 | 4.9588E-82 | 1.6138E-80 |
| AT3G26590 | -1.028528099 | 7.6885E-09 | 3.0089E-08 |
| AT3G26610 | -1.828166041 | 0.0009245 | 0.0021001 |
| AT3G26612 | 1.405960694 | 5.7155E-08 | 2.0812E-07 |
| AT3G26700 | -1.968199327 | 2.1372E-20 | 1.5951E-19 |
| AT3G26740 | 1.237152486 | 2.501E-126 | 1.423E-124 |
| AT3G26770 | -1.294035612 | 3.5925E-07 | 1.2145E-06 |
| AT3G26820 | -3.307858698 | 0.00799031 | 0.01550623 |
| AT3G26840 | 1.19973775 | 2.8858E-11 | 1.3347E-10 |
| AT3G26890 | 1.236730125 | 1.3546E-40 | 1.9698E-39 |
| AT3G26932 | -1.475867173 | 2.8416E-10 | 1.2401E-09 |
| AT3G26960 | -1.337024543 | 0.00124081 | 0.00276575 |
| AT3G27025 | 3.373949477 | 6.8312E-10 | 2.9079E-09 |
| AT3G27170 | -1.592523853 | 4.037E-16 | 2.474E-15 |
| AT3G27180 | -1.258570591 | 2.3098E-22 | 1.8755E-21 |
| AT3G27210 | 1.640838978 | 2.4333E-41 | 3.5934E-40 |
| AT3G27220 | 1.378301712 | 2.4654E-13 | 1.2964E-12 |
| AT3G27250 | 5.072554632 | 6.033E-289 | 1.166E-286 |
| AT3G27260 | 1.213482059 | 1.4231E-54 | 2.8837E-53 |
| AT3G27270 | 1.43476518 | 1.3667E-10 | 6.07E-10 |
| AT3G27460 | 1.075099015 | 5.5923E-08 | 2.0376E-07 |
| AT3G27490 | -6.482039875 | 0.00048751 | 0.00115348 |
| AT3G27510 | -4.153378696 | 2.9104E-05 | 8.1079E-05 |
| AT3G27640 | -1.088604691 | 0.00134587 | 0.00298159 |
| AT3G27850 | -1.233134531 | 1.2286E-72 | 3.4437E-71 |
| AT3G05355 | -1.934522784 | 0.00321261 | 0.00669826 |
| AT3G27870 | 2.555709454 | 3.539E-107 | 1.645E-105 |
| AT3G27880 | 1.517699954 | 6.6677E-29 | 6.9403E-28 |
| AT3G27884 | -1.982023482 | 4.1442E-07 | 1.3923E-06 |
| AT3G27920 | -1.740420585 | 0.00197865 | 0.00427346 |
| AT3G28007 | 5.959670191 | 0 | 0 |
| AT3G28040 | -1.801087913 | 6.8131E-68 | 1.7766E-66 |
| AT3G28080 | -1.584359085 | 2.1161E-13 | 1.1166E-12 |
| AT3G28130 | -1.073924096 | 1.1412E-08 | 4.3998E-08 |
| AT3G28200 | -1.105699939 | 2.5564E-15 | 1.5044E-14 |
| AT3G28210 | 2.353382995 | 1.3388E-84 | 4.5729E-83 |
| AT3G28270 | 2.111548815 | 2.068E-242 | 2.949E-240 |
| AT3G28430 | 1.471383326 | 5.7194E-43 | 8.8591E-42 |
| AT3G28510 | 3.467939755 | 3.8197E-38 | 5.2124E-37 |
| AT3G28550 | -2.901060491 | 1.233E-122 | 6.764E-121 |
| AT3G28560 | -5.594343472 | 0.00955208 | 0.01826214 |
| AT3G28860 | -1.291224874 | 1.3404E-50 | 2.4966E-49 |
| AT3G28900 | -1.051259565 | 2.0059E-29 | 2.113E-28 |
| AT3G28920 | -1.520072555 | 1.6766E-41 | 2.4871E-40 |
| AT3G28930 | -1.10725942 | 7.9564E-10 | 3.3711E-09 |
| AT3G28945 | 1.962924255 | 0.00022501 | 0.00055882 |
| AT3G29000 | 3.28518057 | 5.9064E-15 | 3.4134E-14 |
| AT3G29030 | -1.744443569 | 2.6707E-27 | 2.6096E-26 |
| AT3G29034 | -2.560700018 | 2.7092E-31 | 3.0094E-30 |
| AT3G29035 | 1.120204087 | 4.8259E-08 | 1.7654E-07 |
| AT3G29240 | 1.120553115 | 1.8074E-35 | 2.2758E-34 |
| AT3G29250 | -1.570858853 | 7.1658E-35 | 8.79E-34 |
| AT3G29375 | -1.169916467 | 3.5954E-12 | 1.7555E-11 |
| AT3G29410 | -2.505643825 | 4.6824E-05 | 0.00012746 |
| AT3G29575 | 4.340668678 | 0 | 0 |
| AT3G29630 | -1.410213172 | 3.3586E-06 | 1.0301E-05 |
| AT3G29680 | -2.142983473 | 8.9524E-05 | 0.00023446 |
| AT3G29810 | -1.285267767 | 0.00063376 | 0.00147459 |
| AT3G30210 | 10.06706222 | 1.0436E-11 | 4.9634E-11 |
| AT3G30350 | -2.08502885 | 0.00156576 | 0.00344034 |
| AT3G30460 | 2.425695118 | 4.1836E-09 | 1.6729E-08 |
| AT3G30725 | -6.118547054 | 0.00241168 | 0.00513772 |
| AT3G30775 | -1.428723919 | 1.5093E-61 | 3.5392E-60 |
| AT3G32030 | -2.509208698 | 7.3901E-07 | 2.4205E-06 |
| AT3G32040 | -4.769442486 | 1.0207E-06 | 3.2978E-06 |
| AT3G41762 | 1.942030167 | 0.00891236 | 0.0171324 |
| AT3G41768 | 2.835884472 | 0.00241354 | 0.00514071 |
| AT3G06355 | 2.294667138 | 1.599E-123 | 8.858E-122 |
| AT3G06365 | 3.615846476 | 0.0006424 | 0.00149225 |
| AT3G42180 | -1.257234734 | 0.00018728 | 0.00047039 |
| AT3G42670 | -1.083054606 | 3.063E-09 | 1.2343E-08 |
| AT3G42800 | -1.615683497 | 0.00915404 | 0.01755668 |
| AT3G43205 | 5.668959219 | 0.0071544 | 0.01401278 |
| AT3G43210 | 1.073346973 | 2.5182E-13 | 1.3235E-12 |
| AT3G43220 | 1.190716716 | 3.6985E-32 | 4.2171E-31 |
| AT3G43230 | 1.051894784 | 1.0833E-25 | 9.974E-25 |
| AT3G43270 | 2.573093558 | 2.6703E-93 | 1.0486E-91 |
| AT3G43440 | 1.46962723 | 8.4727E-24 | 7.3059E-23 |
| AT3G43580 | -1.315481288 | 0.00235051 | 0.00501723 |
| AT3G43600 | -1.153309681 | 7.1405E-27 | 6.8655E-26 |
| AT3G43670 | -1.412572023 | 1.9222E-18 | 1.3294E-17 |
| AT3G43800 | -1.08739285 | 8.9767E-17 | 5.6766E-16 |
| AT3G43960 | -2.052493626 | 6.7673E-20 | 4.9615E-19 |
| AT3G44100 | 1.064752632 | 3.376E-32 | 3.8552E-31 |
| AT3G44120 | -2.156907483 | 0.0096062 | 0.01835944 |
| AT3G44150 | -1.152083282 | 8.2177E-12 | 3.9283E-11 |
| AT3G44290 | 1.708626146 | 0.0036711 | 0.00757664 |
| AT3G44310 | -1.076980462 | 1.6685E-56 | 3.5314E-55 |
| AT3G44450 | -3.62475535 | 1.4694E-33 | 1.7425E-32 |
| AT3G44510 | -1.910502606 | 0.00414423 | 0.00847568 |
| AT3G44540 | -1.783702113 | 1.2996E-16 | 8.1418E-16 |
| AT3G44590 | -1.180331086 | 4.4076E-31 | 4.8581E-30 |
| AT3G44720 | -1.121047191 | 1.6827E-25 | 1.5382E-24 |
| AT3G44735 | 2.585731591 | 8.993E-86 | 3.1422E-84 |
| AT3G44740 | -1.013676303 | 8.7221E-05 | 0.00022874 |
| AT3G44750 | -1.780352931 | 1.7012E-84 | 5.7933E-83 |
| AT3G44805 | -5.587344984 | 0.00962232 | 0.01838717 |
| AT3G44860 | 3.129829164 | 2.1096E-42 | 3.2239E-41 |
| AT3G44880 | 2.695318383 | 0 | 0 |
| AT3G44940 | -1.831758824 | 1.5464E-11 | 7.2808E-11 |
| AT3G44970 | -1.708834951 | 1.8057E-24 | 1.5957E-23 |
| AT3G44990 | -6.503036737 | 0 | 0 |
| AT3G45040 | 1.084746503 | 5.1878E-12 | 2.5104E-11 |
| AT3G45050 | -1.009708394 | 2.5037E-12 | 1.2346E-11 |
| AT3G45060 | -2.862481117 | 7.0643E-15 | 4.0671E-14 |
| AT3G45070 | -1.638478159 | 2.4938E-10 | 1.0917E-09 |
| AT3G45080 | -4.58418334 | 4.9499E-05 | 0.00013421 |
| AT3G45160 | -1.338543505 | 2.2703E-17 | 1.4829E-16 |
| AT3G45230 | -1.757062376 | 9.7968E-14 | 5.2993E-13 |
| AT3G45300 | 1.024108182 | 9.5752E-25 | 8.5372E-24 |
| AT3G45330 | -5.104447179 | 6.5057E-05 | 0.00017369 |
| AT3G45410 | -2.614293197 | 1.8104E-12 | 9.0147E-12 |
| AT3G45430 | -2.228129849 | 8.5888E-07 | 2.7959E-06 |
| AT3G45650 | -2.873286869 | 5.9127E-29 | 6.1628E-28 |
| AT3G45660 | -2.894174079 | 0.00040319 | 0.00096691 |
| AT3G45680 | -2.150109844 | 6.984E-30 | 7.4426E-29 |
| AT3G45700 | -4.330813308 | 2.4638E-05 | 6.9229E-05 |
| AT3G45710 | -3.378808709 | 1.0543E-71 | 2.9121E-70 |
| AT3G45780 | -1.128317079 | 2.443E-38 | 3.3518E-37 |
| AT3G45840 | -3.399012739 | 5.6872E-05 | 0.00015304 |
| AT3G45880 | -1.204179413 | 0.00061843 | 0.00144055 |
| AT3G45970 | 2.789710913 | 5.7409E-61 | 1.3244E-59 |
| AT3G46230 | 8.357404989 | 8.9198E-08 | 3.1949E-07 |
| AT3G46270 | -5.119470116 | 2.1935E-15 | 1.2962E-14 |
| AT3G46280 | -2.69503192 | 3.6496E-08 | 1.3507E-07 |
| AT3G46330 | -2.053237394 | 0.0062304 | 0.0123418 |
| AT3G46450 | 2.096561253 | 6.879E-115 | 3.495E-113 |
| AT3G46490 | -3.360021019 | 2.1221E-46 | 3.5627E-45 |
| AT3G46520 | 4.758241852 | 0.00057617 | 0.00134788 |
| AT3G46530 | -1.174966157 | 1.6366E-19 | 1.1858E-18 |
| AT3G46550 | -1.329703133 | 2.657E-18 | 1.8227E-17 |
| AT3G46690 | -3.154830218 | 1.211E-16 | 7.5989E-16 |
| AT3G46700 | -1.513046195 | 7.3733E-18 | 4.9378E-17 |
| AT3G46710 | -5.961350096 | 0.00334876 | 0.00695668 |
| AT3G46720 | -3.203457934 | 5.7579E-15 | 3.3284E-14 |
| AT3G46740 | -1.456420724 | 1.359E-99 | 5.805E-98 |
| AT3G46760 | -5.900873087 | 0.00378334 | 0.00778716 |
| AT3G46880 | -7.625396105 | 3.4273E-06 | 1.0501E-05 |
| AT3G46900 | -2.026840346 | 3.0102E-14 | 1.6747E-13 |
| AT3G46940 | -1.231211542 | 8.5126E-19 | 5.9651E-18 |
| AT3G47040 | -1.838006225 | 0.00434023 | 0.00885114 |
| AT3G47080 | 1.429912215 | 1.2668E-41 | 1.8904E-40 |
| AT3G47160 | 1.528590823 | 2.7256E-41 | 4.0199E-40 |
| AT3G47210 | -1.82158269 | 0.00133924 | 0.00296833 |
| AT3G47295 | 2.103299326 | 4.933E-07 | 1.6445E-06 |
| AT3G47380 | -2.54738818 | 3.1044E-16 | 1.9102E-15 |
| AT3G47430 | -1.38605881 | 7.4239E-19 | 5.223E-18 |
| AT3G47450 | -1.34554847 | 3.464E-39 | 4.8457E-38 |
| AT3G47500 | 1.983854013 | 1.8416E-42 | 2.8237E-41 |
| AT3G47510 | 2.168837079 | 3.5393E-30 | 3.7912E-29 |
| AT3G47580 | 2.75365975 | 3.1616E-09 | 1.2727E-08 |
| AT3G47590 | 1.170823168 | 3.1782E-18 | 2.1698E-17 |
| AT3G47600 | 2.495912332 | 1.9867E-89 | 7.4189E-88 |
| AT3G47610 | 1.018173956 | 2.2854E-15 | 1.3488E-14 |
| AT3G47640 | 1.710723341 | 1.7469E-22 | 1.4271E-21 |
| AT3G47680 | 1.495121372 | 2.8108E-35 | 3.5122E-34 |
| AT3G47740 | -5.655734717 | 0.00790297 | 0.01534981 |
| AT3G47770 | -4.329192198 | 0.00237509 | 0.00506685 |
| AT3G47780 | -1.344973758 | 1.0483E-28 | 1.0842E-27 |
| AT3G47830 | -1.662108307 | 0.00056376 | 0.00132074 |
| AT3G48020 | 3.470216482 | 6.6521E-51 | 1.2492E-49 |
| AT3G48080 | -3.141537664 | 5.6291E-12 | 2.7188E-11 |
| AT3G48110 | -1.052633633 | 1.7204E-35 | 2.1747E-34 |
| AT3G48115 | 1.780239948 | 3.2936E-31 | 3.6444E-30 |
| AT3G48195 | 1.041652528 | 2.5436E-12 | 1.2532E-11 |
| AT3G48200 | -1.419980447 | 2.4905E-51 | 4.755E-50 |
| AT3G48240 | 3.711109243 | 3.462E-33 | 4.0737E-32 |
| AT3G48250 | -1.035012339 | 3.6102E-07 | 1.2201E-06 |
| AT3G48280 | -1.113994628 | 0.00081256 | 0.001864 |
| AT3G48350 | 1.337503863 | 8.2626E-36 | 1.0538E-34 |
| AT3G48390 | 2.418751673 | 4.0749E-08 | 1.5012E-07 |
| AT3G48460 | 1.890041276 | 2.1303E-55 | 4.3867E-54 |
| AT3G48500 | -1.460489671 | 3.3776E-48 | 5.928E-47 |
| AT3G48510 | 6.74434245 | 5.0318E-76 | 1.491E-74 |
| AT3G48520 | 2.590574429 | 6.3377E-12 | 3.0487E-11 |
| AT3G48530 | 1.187084405 | 4.164E-45 | 6.7668E-44 |
| AT3G48610 | -2.016737476 | 4.679E-63 | 1.1261E-61 |
| AT3G48730 | -1.403973312 | 2.766E-102 | 1.211E-100 |
| AT3G48830 | -1.040303704 | 6.6879E-06 | 1.9926E-05 |
| AT3G48850 | -1.001690494 | 0.00039995 | 0.00095985 |
| AT3G48870 | -1.014902655 | 1.2262E-56 | 2.6025E-55 |
| AT3G48920 | -1.165669873 | 6.8909E-05 | 0.00018324 |
| AT3G48970 | -1.815772148 | 0.00022806 | 0.00056596 |
| AT3G48990 | 1.015388303 | 2.6763E-53 | 5.273E-52 |
| AT3G49080 | -1.322995112 | 9.3744E-29 | 9.7133E-28 |
| AT3G49130 | 1.634945601 | 7.1161E-05 | 0.00018899 |
| AT3G49180 | -1.056722336 | 1.5127E-08 | 5.7734E-08 |
| AT3G49190 | -2.123203228 | 0.00085947 | 0.00196296 |
| AT3G49210 | 1.200462035 | 2.1952E-16 | 1.3618E-15 |
| AT3G49220 | 2.498055078 | 0 | 0 |
| AT3G49260 | -1.877840861 | 4.8764E-45 | 7.9189E-44 |
| AT3G49410 | -1.602232573 | 2.7724E-08 | 1.0371E-07 |
| AT3G49530 | 1.138544536 | 3.4282E-26 | 3.2148E-25 |
| AT3G49570 | 1.224733837 | 5.5989E-24 | 4.8609E-23 |
| AT3G49580 | 1.029628014 | 5.4192E-17 | 3.4702E-16 |
| AT3G49590 | 1.491470959 | 2.7263E-35 | 3.4084E-34 |
| AT3G49630 | -7.220531401 | 2.22E-05 | 6.272E-05 |
| AT3G49660 | -1.522175867 | 8.7869E-10 | 3.7099E-09 |
| AT3G49670 | -2.921269189 | 2.752E-131 | 1.657E-129 |
| AT3G49680 | -1.073957829 | 1.1545E-49 | 2.0972E-48 |
| AT3G49720 | -1.148349543 | 7.9545E-36 | 1.0156E-34 |
| AT3G49730 | -1.03786314 | 3.217E-05 | 8.9077E-05 |
| AT3G49750 | -1.189289066 | 0.00056739 | 0.00132842 |
| AT3G49790 | 1.271221756 | 6.2878E-10 | 2.684E-09 |
| AT3G49800 | 1.205922889 | 4.8768E-26 | 4.5435E-25 |
| AT3G49860 | -1.301401241 | 0.00035071 | 0.00084836 |
| AT3G49870 | 1.2131101 | 5.071E-34 | 6.0735E-33 |
| AT3G49880 | 1.157314937 | 2.4642E-21 | 1.9111E-20 |
| AT3G49890 | -1.155320855 | 4.4795E-07 | 1.5006E-06 |
| AT3G49940 | -2.728690471 | 1.5744E-87 | 5.693E-86 |
| AT3G49960 | -5.714713319 | 2.4731E-08 | 9.2942E-08 |
| AT3G49970 | -6.515554808 | 0.00043931 | 0.00104705 |
| AT3G49990 | -1.191675354 | 2.91E-30 | 3.1274E-29 |
| AT3G50040 | -1.429658437 | 0.00199534 | 0.00430624 |
| AT3G50120 | -3.713405761 | 4.6298E-07 | 1.547E-06 |
| AT3G50130 | -7.769720702 | 1.7286E-06 | 5.4513E-06 |
| AT3G50260 | 1.453685027 | 4.1959E-16 | 2.5686E-15 |
| AT3G50270 | -2.315005599 | 7.5258E-35 | 9.2267E-34 |
| AT3G50300 | -3.776008707 | 1.1785E-54 | 2.3902E-53 |
| AT3G50390 | 2.608007306 | 0.00053251 | 0.00125295 |
| AT3G50400 | 1.825700274 | 2.293E-28 | 2.3366E-27 |
| AT3G50560 | -1.687341154 | 0.00135346 | 0.00299724 |
| AT3G50640 | -2.706208123 | 7.1338E-11 | 3.2205E-10 |
| AT3G50700 | -1.200504384 | 1.459E-11 | 6.8806E-11 |
| AT3G50740 | -1.567068415 | 5.2131E-34 | 6.2404E-33 |
| AT3G50750 | -2.434936997 | 1.2519E-13 | 6.705E-13 |
| AT3G50760 | 1.092589342 | 1.2649E-07 | 4.4662E-07 |
| AT3G50800 | 2.167894126 | 2.1248E-12 | 1.0546E-11 |
| AT3G50810 | 1.133057763 | 3.9449E-05 | 0.00010825 |
| AT3G08165 | -2.541137255 | 4.8081E-07 | 1.6036E-06 |
| AT3G50825 | -2.233994345 | 1.4023E-06 | 4.4634E-06 |
| AT3G50830 | 1.233412281 | 1.796E-23 | 1.5296E-22 |
| AT3G50870 | -1.249043103 | 0.00319185 | 0.00665739 |
| AT3G50910 | 2.016882749 | 3.858E-112 | 1.877E-110 |
| AT3G50970 | 5.120794936 | 0 | 0 |
| AT3G50980 | 3.423387528 | 6.4415E-08 | 2.3373E-07 |
| AT3G51000 | 1.551306308 | 1.9639E-44 | 3.151E-43 |
| AT3G51060 | -1.166942579 | 0.00663502 | 0.01307519 |
| AT3G51080 | -1.320940083 | 1.2993E-07 | 4.5832E-07 |
| AT3G51090 | 1.072179739 | 5.7602E-09 | 2.2714E-08 |
| AT3G51130 | 2.081271689 | 8.215E-123 | 4.517E-121 |
| AT3G51280 | -1.334451695 | 3.1116E-13 | 1.6238E-12 |
| AT3G51290 | -1.174936421 | 9.6091E-07 | 3.114E-06 |
| AT3G51320 | -1.487475678 | 3.5655E-11 | 1.6401E-10 |
| AT3G51330 | -1.849103514 | 2.3949E-28 | 2.4393E-27 |
| AT3G51340 | -1.234026132 | 0.00267906 | 0.00566238 |
| AT3G51350 | -1.529005083 | 2.2443E-07 | 7.7434E-07 |
| AT3G51360 | -3.074660787 | 5.4009E-05 | 0.00014576 |
| AT3G51370 | 1.130205164 | 2.0013E-55 | 4.1247E-54 |
| AT3G51400 | -2.355565786 | 0.000128 | 0.00033002 |
| AT3G51410 | -3.345010162 | 0.00688868 | 0.01353648 |
| AT3G51440 | 1.121554518 | 3.2524E-06 | 9.9929E-06 |
| AT3G51500 | 1.061673183 | 2.258E-11 | 1.051E-10 |
| AT3G51540 | -1.790934685 | 1.6633E-05 | 4.7658E-05 |
| AT3G51660 | 1.399195461 | 2.7524E-17 | 1.7881E-16 |
| AT3G51730 | 1.387429803 | 8.1527E-68 | 2.1234E-66 |
| AT3G51750 | 2.77970308 | 2.3519E-55 | 4.8387E-54 |
| AT3G51760 | 1.010305012 | 0.0004174 | 0.00099805 |
| AT3G51810 | 12.27467831 | 3.0444E-17 | 1.9734E-16 |
| AT3G51830 | 1.591877411 | 8.4147E-49 | 1.4976E-47 |
| AT3G51860 | 2.246010434 | 1.0209E-95 | 4.1521E-94 |
| AT3G51870 | -2.391468073 | 1.2715E-71 | 3.5078E-70 |
| AT3G51890 | 1.505407131 | 1.0427E-19 | 7.6053E-19 |
| AT3G51895 | 1.306439391 | 7.5012E-15 | 4.311E-14 |
| AT3G51910 | 1.282050315 | 1.142E-06 | 3.6651E-06 |
| AT3G51990 | 1.283510914 | 3.4297E-11 | 1.5796E-10 |
| AT3G52105 | 1.569550034 | 0.00829484 | 0.01604944 |
| AT3G52170 | -2.039381202 | 2.9493E-48 | 5.1803E-47 |
| AT3G52240 | 1.005279049 | 6.5132E-16 | 3.9428E-15 |
| AT3G52310 | 2.092573035 | 1.7259E-09 | 7.1094E-09 |
| AT3G52340 | 1.948328335 | 2.356E-111 | 1.141E-109 |
| AT3G52370 | -2.305419863 | 1.036E-48 | 1.8394E-47 |
| AT3G52380 | -1.997513662 | 6.79E-168 | 5.846E-166 |
| AT3G52430 | -1.460553752 | 2.7958E-13 | 1.4634E-12 |
| AT3G52450 | -1.858274674 | 1.1553E-17 | 7.6604E-17 |
| AT3G52500 | -1.432535065 | 4.8098E-48 | 8.4092E-47 |
| AT3G52520 | -1.494803009 | 0.00293283 | 0.00615202 |
| AT3G52630 | -1.721801778 | 1.3004E-10 | 5.7834E-10 |
| AT3G52720 | -3.657433603 | 5.1005E-78 | 1.5558E-76 |
| AT3G52748 | -2.149255112 | 2.0566E-10 | 9.0462E-10 |
| AT3G52800 | 1.944232671 | 1.79E-102 | 7.886E-101 |
| AT3G52870 | -1.302511032 | 1.2169E-42 | 1.8734E-41 |
| AT3G52900 | -1.602758954 | 1.6807E-12 | 8.382E-12 |
| AT3G52910 | -1.085046411 | 2.4022E-09 | 9.782E-09 |
| AT3G52920 | 1.174680088 | 2.4617E-30 | 2.6506E-29 |
| AT3G53000 | 1.465240507 | 4.9191E-24 | 4.274E-23 |
| AT3G53040 | 4.853528771 | 5.1885E-07 | 1.7262E-06 |
| AT3G53160 | 1.165861622 | 2.4243E-05 | 6.8177E-05 |
| AT3G53190 | -2.440052877 | 1.058E-103 | 4.706E-102 |
| AT3G53230 | 1.738361861 | 6.1911E-68 | 1.6181E-66 |
| AT3G53232 | 1.718988665 | 0.00283987 | 0.0059713 |
| AT3G53240 | -1.056654976 | 1.1092E-08 | 4.2823E-08 |
| AT3G53235 | -1.501939595 | 0.00436078 | 0.00888909 |
| AT3G53250 | -3.833489353 | 0.00112372 | 0.00251904 |
| AT3G53260 | -1.773473591 | 1.488E-125 | 8.36E-124 |
| AT3G53310 | -1.956448998 | 0.0008417 | 0.00192581 |
| AT3G53420 | 1.344967222 | 1.0021E-94 | 4.0255E-93 |
| AT3G53450 | 1.776643998 | 7.3268E-06 | 2.1736E-05 |
| AT3G53460 | -2.253218293 | 0 | 0 |
| AT3G53470 | 1.440308255 | 4.141E-24 | 3.6076E-23 |
| AT3G53540 | 1.314652638 | 1.757E-45 | 2.8799E-44 |
| AT3G53600 | 2.038909916 | 0.00381575 | 0.00784891 |
| AT3G53700 | -1.253001378 | 1.5768E-20 | 1.1811E-19 |
| AT3G53850 | -1.020332031 | 3.0743E-07 | 1.0457E-06 |
| AT3G00910 | 2.47814933 | 0.00721547 | 0.01412269 |
| AT3G53960 | 2.165614392 | 7.0035E-62 | 1.6507E-60 |
| AT3G53980 | 2.156539403 | 5.0759E-91 | 1.9338E-89 |
| AT3G53990 | 1.777364935 | 8.887E-152 | 6.583E-150 |
| AT3G54030 | 1.395555433 | 1.2342E-35 | 1.5636E-34 |
| AT3G54040 | -2.064093379 | 3.493E-102 | 1.527E-100 |
| AT3G54090 | -1.321939081 | 8.3952E-36 | 1.0701E-34 |
| AT3G54130 | 1.91782652 | 5.0945E-55 | 1.0415E-53 |
| AT3G54150 | -1.691275092 | 1.5587E-05 | 4.4773E-05 |
| AT3G54200 | 1.798875443 | 1.1348E-51 | 2.1764E-50 |
| AT3G08375 | 1.784226788 | 3.1474E-07 | 1.0697E-06 |
| AT3G54380 | 1.045359987 | 6.7414E-12 | 3.2382E-11 |
| AT3G54390 | 1.363459912 | 1.4112E-23 | 1.2059E-22 |
| AT3G54400 | -2.374921688 | 1.622E-156 | 1.263E-154 |
| AT3G54420 | -2.144743671 | 1.4412E-13 | 7.6881E-13 |
| AT3G54470 | -1.409400314 | 3.8336E-56 | 8.0467E-55 |
| AT3G54580 | -2.944945769 | 5.296E-146 | 3.684E-144 |
| AT3G54590 | -5.619708682 | 9.667E-113 | 4.743E-111 |
| AT3G54600 | -3.3830998 | 2.267E-190 | 2.341E-188 |
| AT3G54620 | 1.072359131 | 2.6164E-32 | 2.9967E-31 |
| AT3G54640 | -1.237348713 | 1.0858E-38 | 1.4986E-37 |
| AT3G54680 | 2.080009975 | 8.5726E-84 | 2.8554E-82 |
| AT3G54770 | -2.257351896 | 8.3141E-14 | 4.5122E-13 |
| AT3G54780 | -1.901980773 | 4.7057E-08 | 1.7239E-07 |
| AT3G54810 | -1.273836154 | 8.5337E-21 | 6.475E-20 |
| AT3G54820 | 1.504193331 | 3.3404E-38 | 4.5611E-37 |
| AT3G54830 | -2.303866423 | 1.6183E-37 | 2.1796E-36 |
| AT3G08435 | 10.4804939 | 1.1572E-12 | 5.8361E-12 |
| AT3G54850 | 1.119177338 | 3.808E-15 | 2.2209E-14 |
| AT3G55010 | -1.600492843 | 3.3888E-56 | 7.1197E-55 |
| AT3G55070 | 1.564736918 | 5.0223E-48 | 8.7741E-47 |
| AT3G55090 | 2.813829285 | 3.7693E-44 | 5.9973E-43 |
| AT3G55110 | -1.457192244 | 2.0598E-14 | 1.1556E-13 |
| AT3G55150 | -3.668720016 | 2.921E-15 | 1.7132E-14 |
| AT3G55200 | -1.069754131 | 1.2779E-07 | 4.5113E-07 |
| AT3G55230 | -2.567134838 | 1.3767E-46 | 2.3198E-45 |
| AT3G55240 | 1.997886125 | 2.1141E-05 | 5.9875E-05 |
| AT3G55290 | 1.553887828 | 3.6885E-16 | 2.2635E-15 |
| AT3G55340 | -1.341411366 | 1.9697E-28 | 2.0117E-27 |
| AT3G55430 | 1.621315845 | 2.901E-116 | 1.497E-114 |
| AT3G55500 | 2.246720945 | 7.763E-116 | 3.98E-114 |
| AT3G55510 | -1.903473549 | 6.6419E-47 | 1.1259E-45 |
| AT3G55610 | 1.916908573 | 1.137E-168 | 9.904E-167 |
| AT3G55640 | 1.601918118 | 2.5997E-32 | 2.9792E-31 |
| AT3G55646 | -4.532358512 | 0.00100182 | 0.00226312 |
| AT3G55820 | 1.245506195 | 0.00589781 | 0.01173187 |
| AT3G55840 | 4.04470422 | 5.7218E-80 | 1.7982E-78 |
| AT3G55880 | 3.532241826 | 4.725E-137 | 2.946E-135 |
| AT3G55940 | 4.383906589 | 4.531E-146 | 3.162E-144 |
| AT3G55970 | 2.347434247 | 1.2334E-19 | 8.9819E-19 |
| AT3G56000 | -1.050604681 | 0.00692758 | 0.01360707 |
| AT3G56070 | -1.405501662 | 4.0543E-56 | 8.5022E-55 |
| AT3G56080 | 1.877782397 | 9.1014E-40 | 1.293E-38 |
| AT3G56090 | -1.056037753 | 1.0637E-43 | 1.6761E-42 |
| AT3G56100 | -1.481600811 | 1.6179E-09 | 6.6823E-09 |
| AT3G56170 | 1.191576967 | 4.751E-20 | 3.5058E-19 |
| AT3G56220 | -1.680868884 | 0.00599668 | 0.01191502 |
| AT3G56230 | -2.442438675 | 0.0002685 | 0.00066036 |
| AT3G56240 | 1.077193422 | 8.7415E-49 | 1.5533E-47 |
| AT3G56250 | 1.715484882 | 2.4457E-09 | 9.9486E-09 |
| AT3G56260 | 1.787679115 | 7.8363E-29 | 8.1343E-28 |
| AT3G56270 | 1.15667264 | 7.2829E-14 | 3.9658E-13 |
| AT3G56320 | 1.162080953 | 2.4406E-09 | 9.9294E-09 |
| AT3G56330 | -1.414544264 | 1.0186E-12 | 5.1705E-12 |
| AT3G56360 | 1.322767271 | 3.5213E-05 | 9.7196E-05 |
| AT3G56370 | -1.32428023 | 1.4898E-49 | 2.6976E-48 |
| AT3G56480 | -1.094213199 | 1.2854E-16 | 8.0547E-16 |
| AT3G56590 | -1.229319727 | 5.0154E-25 | 4.5087E-24 |
| AT3G56600 | 7.199465039 | 2.5734E-05 | 7.2166E-05 |
| AT3G56620 | 2.202786836 | 2.5572E-27 | 2.5019E-26 |
| AT3G56790 | 3.560051145 | 1.2166E-07 | 4.2994E-07 |
| AT3G56810 | -1.594984102 | 1.2787E-13 | 6.8423E-13 |
| AT3G56880 | 2.322213678 | 3.849E-136 | 2.393E-134 |
| AT3G57010 | 2.159662468 | 1.842E-152 | 1.387E-150 |
| AT3G57090 | 1.312959118 | 7.0754E-40 | 1.009E-38 |
| AT3G57130 | -3.061722046 | 4.9438E-05 | 0.00013406 |
| AT3G57150 | -1.070464903 | 1.3887E-55 | 2.891E-54 |
| AT3G57157 | -2.090119009 | 8.4921E-07 | 2.7656E-06 |
| AT3G57180 | -1.332445986 | 4.6179E-24 | 4.0199E-23 |
| AT3G57230 | 1.453821945 | 1.4462E-34 | 1.7561E-33 |
| AT3G57260 | 3.286209072 | 3.3879E-07 | 1.148E-06 |
| AT3G57460 | 2.532643848 | 0.00099541 | 0.00224953 |
| AT3G57480 | 1.067255144 | 0.00016698 | 0.00042309 |
| AT3G57490 | -1.480244139 | 5.1285E-35 | 6.3353E-34 |
| AT3G57500 | -1.254162741 | 0.00166045 | 0.00363124 |
| AT3G57520 | 1.663018444 | 5.211E-200 | 5.635E-198 |
| AT3G57540 | 1.967454937 | 4.1636E-40 | 5.9797E-39 |
| AT3G57610 | -1.004820289 | 4.6287E-40 | 6.642E-39 |
| AT3G57680 | 3.446669921 | 7.4132E-66 | 1.8835E-64 |
| AT3G57760 | 1.13393512 | 1.0136E-06 | 3.2761E-06 |
| AT3G57830 | -1.362313133 | 6.3393E-14 | 3.4668E-13 |
| AT3G57940 | -1.651911237 | 3.4926E-41 | 5.1444E-40 |
| AT3G58100 | -1.375184509 | 1.2143E-07 | 4.2922E-07 |
| AT3G58120 | -2.60623036 | 9.9243E-34 | 1.1818E-32 |
| AT3G58150 | 2.094171384 | 0.00113627 | 0.00254618 |
| AT3G58350 | 1.126604949 | 0.00268665 | 0.00567685 |
| AT3G58450 | 5.182737938 | 6.8313E-05 | 0.00018183 |
| AT3G58520 | -1.065687646 | 5.5017E-05 | 0.00014841 |
| AT3G58610 | -1.177358352 | 4.192E-93 | 1.6434E-91 |
| AT3G58640 | 1.042944645 | 3.6865E-31 | 4.0751E-30 |
| AT3G58660 | -1.021410266 | 1.2734E-39 | 1.8024E-38 |
| AT3G58710 | 1.209397493 | 1.0994E-07 | 3.9018E-07 |
| AT3G58750 | 1.108611124 | 1.0576E-48 | 1.8763E-47 |
| AT3G58810 | 1.7293106 | 5.0895E-15 | 2.951E-14 |
| AT3G58850 | -2.352819287 | 3.9666E-07 | 1.335E-06 |
| AT3G58990 | -5.83738765 | 4.08E-287 | 7.823E-285 |
| AT3G59040 | -1.514922456 | 3.7974E-46 | 6.3242E-45 |
| AT3G59050 | 1.027668207 | 7.807E-22 | 6.1892E-21 |
| AT3G59120 | -3.157417486 | 0.00194774 | 0.0042111 |
| AT3G59210 | 1.013033438 | 1.077E-16 | 6.7766E-16 |
| AT3G59250 | -1.554141761 | 0.00010953 | 0.0002847 |
| AT3G59280 | 1.170986746 | 1.2202E-34 | 1.4848E-33 |
| AT3G59310 | -1.05426353 | 0.00056188 | 0.00131674 |
| AT3G59340 | -2.903254205 | 3.7256E-11 | 1.7114E-10 |
| AT3G59370 | -1.160564492 | 0.00675955 | 0.01330795 |
| AT3G59480 | 1.912209054 | 2.4895E-08 | 9.3515E-08 |
| AT3G59490 | 1.667875409 | 7.311E-19 | 5.1468E-18 |
| AT3G59530 | 1.011154234 | 0.00197919 | 0.004274 |
| AT3G59680 | -1.317837046 | 0.00013089 | 0.00033718 |
| AT3G59730 | -3.676321299 | 0.00299126 | 0.00626651 |
| AT3G59750 | -2.165079897 | 5.2267E-05 | 0.00014121 |
| AT3G59840 | -1.115570473 | 5.8806E-17 | 3.7561E-16 |
| AT3G59880 | 1.132949208 | 0.00323994 | 0.00675092 |
| AT3G59930 | 2.451468117 | 0.00091992 | 0.00209032 |
| AT3G59980 | -1.700987821 | 3.0007E-42 | 4.5673E-41 |
| AT3G60040 | 1.432285673 | 2.3825E-06 | 7.4118E-06 |
| AT3G60080 | 1.079402686 | 1.9276E-14 | 1.0833E-13 |
| AT3G60110 | 1.363847607 | 3.3365E-19 | 2.3819E-18 |
| AT3G60120 | -2.746226383 | 2.2641E-05 | 6.3901E-05 |
| AT3G60140 | 1.972902977 | 3.0966E-19 | 2.212E-18 |
| AT3G60160 | 1.09969543 | 9.4736E-10 | 3.9865E-09 |
| AT3G60180 | 1.251481501 | 1.0727E-07 | 3.814E-07 |
| AT3G60220 | 1.467094351 | 1.3086E-12 | 6.5765E-12 |
| AT3G60270 | -4.576114806 | 7.9076E-06 | 2.3398E-05 |
| AT3G60280 | -8.762765281 | 1.1951E-08 | 4.5976E-08 |
| AT3G60300 | 1.887807605 | 3.505E-92 | 1.3486E-90 |
| AT3G60320 | -1.639130055 | 2.4387E-46 | 4.0823E-45 |
| AT3G60330 | -2.677738898 | 3.5405E-23 | 2.9786E-22 |
| AT3G60415 | 2.344890313 | 6.4204E-54 | 1.2816E-52 |
| AT3G60440 | -1.261113073 | 3.274E-18 | 2.2292E-17 |
| AT3G60520 | 1.185358383 | 1.479E-09 | 6.1287E-09 |
| AT3G60580 | -2.096191684 | 0.00055664 | 0.00130579 |
| AT3G60620 | -1.228033154 | 1.5662E-17 | 1.0316E-16 |
| AT3G60640 | 1.033148812 | 1.9135E-07 | 6.6413E-07 |
| AT3G60670 | 1.608480898 | 0.00027303 | 0.00067027 |
| AT3G60690 | 1.060702713 | 1.1235E-13 | 6.0445E-13 |
| AT3G60720 | -1.049000488 | 0.00438768 | 0.00893593 |
| AT3G60930 | 1.871347898 | 2.6998E-12 | 1.3281E-11 |
| AT3G60960 | -1.232824794 | 1.7397E-09 | 7.1623E-09 |
| AT3G60966 | 3.016104489 | 0.00359672 | 0.00743121 |
| AT3G09545 | 4.768944277 | 0.00980536 | 0.01870561 |
| AT3G61040 | 5.599865414 | 0.00876618 | 0.01687414 |
| AT3G61060 | 3.532037766 | 2.216E-121 | 1.198E-119 |
| AT3G61100 | -1.533201017 | 5.7431E-15 | 3.3207E-14 |
| AT3G61170 | -1.155363903 | 0.00855722 | 0.01650951 |
| AT3G61270 | -1.603892836 | 3.1865E-11 | 1.4694E-10 |
| AT3G61400 | -3.392860822 | 4.5399E-10 | 1.9547E-09 |
| AT3G61420 | 1.888518846 | 2.1355E-50 | 3.9548E-49 |
| AT3G61430 | 1.129692641 | 1.3055E-34 | 1.5878E-33 |
| AT3G61450 | 2.585516633 | 1.4381E-07 | 5.0558E-07 |
| AT3G61460 | 1.329143795 | 1.3949E-34 | 1.6947E-33 |
| AT3G61570 | 1.245427641 | 3.8246E-17 | 2.4664E-16 |
| AT3G61630 | 2.371785952 | 8.9859E-50 | 1.6402E-48 |
| AT3G61710 | 1.001996117 | 2.7747E-15 | 1.6291E-14 |
| AT3G61780 | -1.629345052 | 8.416E-41 | 1.2301E-39 |
| AT3G61790 | 1.211050094 | 6.0262E-21 | 4.6015E-20 |
| AT3G61820 | -2.581806799 | 6.301E-118 | 3.29E-116 |
| AT3G61840 | -2.598337349 | 0.00290401 | 0.00609325 |
| AT3G61880 | -1.214373778 | 0.00360223 | 0.00744125 |
| AT3G61890 | 5.654069542 | 0 | 0 |
| AT3G61920 | -3.209081753 | 2.0323E-19 | 1.4674E-18 |
| AT3G61950 | -1.599743699 | 4.577E-08 | 1.6776E-07 |
| AT3G61960 | 1.415612633 | 1.6124E-40 | 2.3417E-39 |
| AT3G61980 | 1.442302873 | 6.7317E-15 | 3.8815E-14 |
| AT3G62090 | 3.050993011 | 8.3524E-07 | 2.7217E-06 |
| AT3G62100 | 2.478244141 | 2.5742E-15 | 1.5141E-14 |
| AT3G62110 | -1.076363493 | 7.8455E-20 | 5.7354E-19 |
| AT3G62150 | -1.790625038 | 5.6659E-22 | 4.5311E-21 |
| AT3G62190 | 1.260867855 | 6.2236E-22 | 4.9667E-21 |
| AT3G62260 | 2.212090535 | 3.3236E-68 | 8.7464E-67 |
| AT3G62270 | -1.880483034 | 1.7718E-47 | 3.0463E-46 |
| AT3G62280 | -2.577461259 | 1.1878E-05 | 3.4529E-05 |
| AT3G62380 | 5.996790364 | 0.0028649 | 0.00601947 |
| AT3G62460 | -1.079345154 | 4.3245E-09 | 1.7283E-08 |
| AT3G62530 | -1.299361659 | 1.3869E-88 | 5.1122E-87 |
| AT3G62590 | 3.377466479 | 2.192E-103 | 9.729E-102 |
| AT3G62650 | 1.000213297 | 1.1366E-29 | 1.2067E-28 |
| AT3G62660 | 1.970305288 | 5.009E-99 | 2.1281E-97 |
| AT3G62680 | -6.112694971 | 3.982E-49 | 7.1594E-48 |
| AT3G62730 | 2.090121866 | 1.1291E-08 | 4.3546E-08 |
| AT3G62740 | 1.05587504 | 3.1906E-18 | 2.1776E-17 |
| AT3G62770 | 1.145477495 | 1.8526E-27 | 1.8275E-26 |
| AT3G62800 | 1.162259839 | 6.9989E-14 | 3.8157E-13 |
| AT3G62920 | 1.411449498 | 4.645E-07 | 1.5517E-06 |
| AT3G62930 | -1.741397778 | 2.6792E-08 | 1.0033E-07 |
| AT3G62990 | 4.201487126 | 7.2661E-23 | 6.0132E-22 |
| AT3G63000 | 1.277439167 | 1.1747E-42 | 1.8097E-41 |
| AT3G63040 | 6.058143144 | 0.00233538 | 0.0049882 |
| AT3G63050 | 2.014451998 | 7.3646E-08 | 2.6583E-07 |
| AT3G63052 | 3.312760611 | 0.0055828 | 0.01115289 |
| AT3G09885 | 7.295719355 | 1.4508E-05 | 4.1794E-05 |
| AT3G63060 | 5.569563866 | 1.335E-195 | 1.423E-193 |
| AT3G63090 | -1.104840617 | 1.8641E-06 | 5.8608E-06 |
| AT3G63110 | -3.640913103 | 1.1616E-42 | 1.7908E-41 |
| AT3G63120 | 1.047550198 | 7.1622E-24 | 6.1922E-23 |
| AT3G63160 | -1.122969916 | 6.5188E-69 | 1.7275E-67 |
| AT3G63200 | -2.482384396 | 1.085E-107 | 5.073E-106 |
| AT3G63210 | 1.29046804 | 9.9081E-31 | 1.0827E-29 |
| AT3G63310 | 1.182425397 | 1.0609E-35 | 1.3477E-34 |
| AT3G63380 | -1.625556066 | 1.8088E-34 | 2.1941E-33 |
| AT3G63445 | 1.129193461 | 1.3517E-06 | 4.3139E-06 |
| AT4G00020 | -1.065992651 | 1.0996E-09 | 4.6044E-09 |
| AT4G00050 | -1.059846043 | 8.4345E-18 | 5.6302E-17 |
| AT4G00080 | -1.049080639 | 0.00010597 | 0.00027589 |
| AT4G00355 | 1.031725141 | 1.0416E-25 | 9.6017E-25 |
| AT4G00360 | 1.019046325 | 1.2811E-23 | 1.0968E-22 |
| AT4G00370 | -1.297855419 | 4.3652E-57 | 9.3871E-56 |
| AT4G00400 | -1.01954081 | 4.3322E-17 | 2.7883E-16 |
| AT4G00430 | 2.335846774 | 1.15E-208 | 1.312E-206 |
| AT4G00440 | 1.611262482 | 4.009E-76 | 1.1895E-74 |
| AT4G00480 | -1.437573869 | 1.0865E-13 | 5.8535E-13 |
| AT4G00620 | -1.456050141 | 3.114E-23 | 2.6237E-22 |
| AT4G00670 | -1.639328702 | 0.00475431 | 0.00962931 |
| AT4G00680 | -2.478090062 | 8.2808E-10 | 3.5046E-09 |
| AT4G03725 | 2.208328592 | 0.0090141 | 0.01731339 |
| AT4G00910 | 3.201094457 | 5.0253E-23 | 4.1953E-22 |
| AT4G00940 | 1.136744609 | 5.2018E-06 | 1.5691E-05 |
| AT4G00950 | -1.982891915 | 6.5532E-14 | 3.5804E-13 |
| AT4G00955 | -2.493932037 | 1.1605E-06 | 3.7225E-06 |
| AT4G00970 | -1.096320652 | 0.00026969 | 0.00066306 |
| AT4G01000 | 1.002890972 | 9.4534E-34 | 1.1269E-32 |
| AT4G01023 | 5.807852771 | 0.00528245 | 0.01060954 |
| AT4G01026 | 2.211291326 | 5.6623E-26 | 5.2667E-25 |
| AT4G03845 | 5.992934631 | 0.00258161 | 0.00547263 |
| AT4G01037 | -1.639182373 | 7.6168E-64 | 1.8626E-62 |
| AT4G01060 | 2.823333445 | 1.765E-32 | 2.038E-31 |
| AT4G01070 | 1.204663696 | 1.3824E-70 | 3.7505E-69 |
| AT4G01080 | -3.245870054 | 6.8238E-71 | 1.8623E-69 |
| AT4G01120 | 2.43058539 | 3.862E-101 | 1.675E-99 |
| AT4G01240 | -1.83354406 | 0.00089688 | 0.00204408 |
| AT4G01280 | 1.173808944 | 2.7284E-14 | 1.5224E-13 |
| AT4G01350 | -2.080445114 | 8.633E-10 | 3.6483E-09 |
| AT4G01360 | 3.25267505 | 6.2327E-12 | 2.9995E-11 |
| AT4G01430 | 1.39798503 | 6.5218E-07 | 2.145E-06 |
| AT4G01490 | -2.965698242 | 0.00124874 | 0.00278152 |
| AT4G01540 | 1.243719148 | 2.2564E-12 | 1.118E-11 |
| AT4G01550 | 1.797506077 | 4.1567E-34 | 4.9942E-33 |
| AT4G01580 | -1.124007799 | 7.2469E-06 | 2.151E-05 |
| AT4G01590 | -1.418926502 | 2.9124E-31 | 3.2304E-30 |
| AT4G01680 | -1.493059575 | 6.2882E-05 | 0.0001682 |
| AT4G01700 | -1.523611004 | 2.4354E-12 | 1.2022E-11 |
| AT4G01740 | -1.901624488 | 1.9806E-05 | 5.6277E-05 |
| AT4G01750 | -2.938534756 | 1.2909E-26 | 1.2303E-25 |
| AT4G01770 | -1.800894232 | 0.00219672 | 0.00472076 |
| AT4G01830 | -6.018587956 | 0.00323154 | 0.00673525 |
| AT4G01895 | 1.30087357 | 0.00168549 | 0.00368142 |
| AT4G01920 | -2.458284886 | 2.6494E-07 | 9.0684E-07 |
| AT4G01960 | 1.860936859 | 8.8707E-61 | 2.0341E-59 |
| AT4G01985 | 4.815338187 | 1.483E-118 | 7.816E-117 |
| AT4G02000 | 2.142443314 | 0.00118723 | 0.00265462 |
| AT4G02005 | 2.218120332 | 7.6161E-16 | 4.591E-15 |
| AT4G02060 | -1.258226136 | 5.6049E-27 | 5.4095E-26 |
| AT4G02270 | -6.16357545 | 2.8401E-26 | 2.6732E-25 |
| AT4G02280 | 3.83851825 | 0 | 0 |
| AT4G02290 | -1.508748606 | 1.7162E-31 | 1.9176E-30 |
| AT4G02360 | 3.942623115 | 1.301E-160 | 1.052E-158 |
| AT4G02380 | 2.18807109 | 0 | 0 |
| AT4G02410 | 1.656126944 | 1.2032E-51 | 2.3049E-50 |
| AT4G02510 | -1.003972576 | 2.9166E-65 | 7.3208E-64 |
| AT4G02690 | 5.812120019 | 0.0046319 | 0.00939724 |
| AT4G02740 | 1.020939277 | 1.1994E-13 | 6.4345E-13 |
| AT4G02810 | -1.97326011 | 0.00231409 | 0.00494691 |
| AT4G02850 | -3.158569013 | 5.2638E-26 | 4.9E-25 |
| AT4G02880 | 1.227377613 | 1.7505E-21 | 1.365E-20 |
| AT4G02890 | 1.5758911 | 2.43E-163 | 2.001E-161 |
| AT4G02990 | -1.828856204 | 9.955E-59 | 2.203E-57 |
| AT4G03030 | 1.775790231 | 1.0413E-73 | 2.9661E-72 |
| AT4G03100 | -1.147947173 | 3.8555E-11 | 1.7696E-10 |
| AT4G03200 | 1.060023035 | 9.8628E-36 | 1.2557E-34 |
| AT4G03210 | -2.135549376 | 4.55E-127 | 2.615E-125 |
| AT4G03292 | -1.708818762 | 0.00621687 | 0.01231606 |
| AT4G03320 | 1.626983276 | 5.9046E-29 | 6.1572E-28 |
| AT4G03400 | -1.319412395 | 4.8058E-21 | 3.6807E-20 |
| AT4G03420 | 1.536666018 | 5.9095E-40 | 8.4534E-39 |
| AT4G03540 | 2.210059479 | 1.1371E-21 | 8.9493E-21 |
| AT4G03820 | 2.327874493 | 1.8701E-75 | 5.4984E-74 |
| AT4G04020 | 1.861481904 | 1.0063E-28 | 1.0417E-27 |
| AT4G04450 | -2.719024676 | 2.5318E-13 | 1.3304E-12 |
| AT4G04540 | -6.14420069 | 0.0017939 | 0.00389843 |
| AT4G04570 | -3.53013201 | 3.58E-94 | 1.423E-92 |
| AT4G04620 | 1.355606679 | 2.8346E-23 | 2.3936E-22 |
| AT4G04700 | -1.146380725 | 2.4405E-05 | 6.8618E-05 |
| AT4G04745 | -3.634567834 | 2.5417E-17 | 1.6559E-16 |
| AT4G04840 | -2.689586944 | 2.234E-108 | 1.053E-106 |
| AT4G04940 | -1.868104597 | 6.8639E-55 | 1.3983E-53 |
| AT4G04990 | -2.613242638 | 5.7527E-10 | 2.4598E-09 |
| AT4G05010 | 1.777703528 | 1.9388E-15 | 1.1484E-14 |
| AT4G05020 | 1.550340986 | 1.624E-61 | 3.8042E-60 |
| AT4G05050 | 1.291942616 | 1.171E-129 | 6.94E-128 |
| AT4G05070 | 1.674305929 | 2.1775E-35 | 2.7313E-34 |
| AT4G05100 | 4.075903683 | 1.495E-166 | 1.264E-164 |
| AT4G05110 | 1.886585406 | 9.9018E-14 | 5.3548E-13 |
| AT4G05170 | -3.203218972 | 0.00132892 | 0.00294838 |
| AT4G05190 | -1.149491205 | 7.3915E-13 | 3.7847E-12 |
| AT4G05200 | -2.779522427 | 1.2978E-21 | 1.0182E-20 |
| AT4G05320 | 1.14804957 | 1.085E-114 | 5.474E-113 |
| AT4G05400 | -1.838762866 | 6.1966E-34 | 7.4061E-33 |
| AT4G05410 | -1.084696739 | 1.8672E-18 | 1.2922E-17 |
| AT4G06534 | 1.400852372 | 3.3648E-08 | 1.2501E-07 |
| AT4G06536 | 1.818625935 | 9.7984E-10 | 4.1171E-09 |
| AT4G06746 | -1.254495522 | 5.615E-06 | 1.6888E-05 |
| AT4G07400 | -1.137616207 | 2.2597E-05 | 6.3785E-05 |
| AT4G07960 | -1.741104549 | 2.4268E-06 | 7.5416E-06 |
| AT4G08150 | -1.738125899 | 3.7684E-15 | 2.1996E-14 |
| AT4G08170 | 1.055865105 | 4.5575E-17 | 2.9299E-16 |
| AT4G08290 | 2.288291521 | 3.2164E-53 | 6.3317E-52 |
| AT4G08400 | -5.17771161 | 3.4619E-23 | 2.9135E-22 |
| AT4G08410 | -6.016404916 | 3.4684E-30 | 3.7205E-29 |
| AT4G08570 | 1.594839119 | 4.1004E-35 | 5.0873E-34 |
| AT4G08620 | -3.001991103 | 7.6248E-05 | 0.00020165 |
| AT4G08770 | -3.206461381 | 4.6031E-41 | 6.7714E-40 |
| AT4G08780 | -3.294364715 | 3.4451E-14 | 1.9088E-13 |
| AT4G08910 | 1.766308631 | 3.9695E-05 | 0.00010889 |
| AT4G08950 | -2.380184516 | 2.336E-46 | 3.9133E-45 |
| AT4G09030 | 2.219394697 | 0.00011682 | 0.00030225 |
| AT4G09040 | -1.341330159 | 1.1334E-51 | 2.1764E-50 |
| AT4G09160 | -1.777955837 | 3.2515E-35 | 4.0472E-34 |
| AT4G09420 | -3.642918332 | 0.0001646 | 0.00041732 |
| AT4G09490 | 1.10879025 | 0.00107947 | 0.00242628 |
| AT4G09500 | 1.88482826 | 4.3209E-43 | 6.702E-42 |
| AT4G09595 | 5.925051856 | 0.00328836 | 0.00684306 |
| AT4G09600 | 7.429508919 | 5.555E-32 | 6.2963E-31 |
| AT4G09610 | 4.159368348 | 0.0001543 | 0.00039275 |
| AT4G09650 | -1.229608143 | 1.3329E-98 | 5.6319E-97 |
| AT4G09730 | -1.090254423 | 9.7041E-37 | 1.2717E-35 |
| AT4G09760 | 2.036934755 | 1.152E-99 | 4.9305E-98 |
| AT4G09820 | 2.581224451 | 8.0135E-70 | 2.151E-68 |
| AT4G09830 | 1.189377001 | 4.5129E-36 | 5.8009E-35 |
| AT4G09990 | -1.57136897 | 9.6873E-07 | 3.1373E-06 |
| AT4G10040 | 1.234299683 | 4.9112E-29 | 5.1284E-28 |
| AT4G10120 | -1.57768555 | 1.3498E-57 | 2.9359E-56 |
| AT4G10250 | 5.289027173 | 1.451E-105 | 6.568E-104 |
| AT4G10350 | -2.384936288 | 0.00968376 | 0.01849529 |
| AT4G10370 | -3.648520272 | 4.7824E-07 | 1.596E-06 |
| AT4G10380 | -1.892429079 | 1.6506E-25 | 1.51E-24 |
| AT4G10450 | -1.447986366 | 6.8731E-52 | 1.3267E-50 |
| AT4G10510 | -2.730015851 | 4.6039E-13 | 2.3808E-12 |
| AT4G10530 | -8.428371266 | 6.5628E-08 | 2.379E-07 |
| AT4G10560 | -3.156083766 | 0.00368661 | 0.00760521 |
| AT4G10720 | -3.038745537 | 0.00030263 | 0.00073839 |
| AT4G10843 | 2.121303977 | 0.00030781 | 0.00075007 |
| AT4G10955 | 1.076384838 | 2.4582E-12 | 1.2129E-11 |
| AT4G10960 | 3.982081925 | 2.987E-233 | 3.94E-231 |
| AT4G11050 | -2.465627726 | 7.352E-09 | 2.8807E-08 |
| AT4G11140 | -1.664549398 | 2.3862E-05 | 6.7182E-05 |
| AT4G11170 | -2.61394603 | 1.3764E-08 | 5.2682E-08 |
| AT4G11190 | -2.1659773 | 1.1853E-26 | 1.1325E-25 |
| AT4G11220 | 1.520473662 | 1.0825E-76 | 3.2329E-75 |
| AT4G11290 | -1.155113351 | 1.0343E-09 | 4.3419E-09 |
| AT4G11330 | 1.39972386 | 3.0591E-20 | 2.2713E-19 |
| AT4G11350 | 1.95542847 | 3.0648E-21 | 2.3688E-20 |
| AT4G11460 | -11.30458159 | 9.7707E-15 | 5.5788E-14 |
| AT4G11530 | -2.296865878 | 2.0331E-11 | 9.4883E-11 |
| AT4G11570 | 2.121196507 | 6.66E-187 | 6.607E-185 |
| AT4G11610 | -1.754784541 | 2.4241E-05 | 6.8177E-05 |
| AT4G11650 | -1.852696779 | 9.6734E-84 | 3.2173E-82 |
| AT4G11660 | 1.475025123 | 7.8813E-15 | 4.5226E-14 |
| AT4G11740 | 1.026025263 | 1.7141E-21 | 1.3378E-20 |
| AT4G11780 | -3.218244858 | 2.7858E-06 | 8.6161E-06 |
| AT4G11800 | 1.245928952 | 2.6356E-33 | 3.1061E-32 |
| AT4G11880 | 1.044839245 | 0.00052654 | 0.00123941 |
| AT4G11890 | 3.012909215 | 4.9052E-44 | 7.7936E-43 |
| AT4G11910 | 3.338403725 | 1.9971E-23 | 1.6983E-22 |
| AT4G11911 | 1.73326715 | 0.00382048 | 0.00785794 |
| AT4G11960 | 1.071484543 | 1.2467E-21 | 9.7851E-21 |
| AT4G12000 | 2.569523122 | 2.4411E-81 | 7.8447E-80 |
| AT4G12005 | 2.121157484 | 0.00712899 | 0.0139666 |
| AT4G12020 | 1.334637737 | 8.9517E-31 | 9.7911E-30 |
| AT4G12030 | -3.921936532 | 2.851E-164 | 2.366E-162 |
| AT4G12040 | 1.15980995 | 3.9143E-40 | 5.6275E-39 |
| AT4G12070 | 1.086487263 | 5.1044E-10 | 2.1924E-09 |
| AT4G12090 | -1.973417964 | 0.00218567 | 0.00469791 |
| AT4G12120 | 1.175146489 | 3.3698E-10 | 1.462E-09 |
| AT4G12240 | -1.284755997 | 2.1198E-05 | 6.0022E-05 |
| AT4G12270 | -7.805493018 | 1.4424E-06 | 4.5817E-06 |
| AT4G12310 | -1.550188101 | 5.1529E-47 | 8.7477E-46 |
| AT4G12320 | -4.440107752 | 4.768E-284 | 9.066E-282 |
| AT4G12390 | -2.469444905 | 8.4493E-50 | 1.5459E-48 |
| AT4G12400 | 1.109056526 | 5.7726E-23 | 4.8016E-22 |
| AT4G12410 | 6.087660784 | 9.1074E-64 | 2.2177E-62 |
| AT4G12420 | -1.446769846 | 1.2852E-79 | 4.0059E-78 |
| AT4G12430 | 3.401763917 | 4.631E-137 | 2.895E-135 |
| AT4G12470 | -2.63864103 | 1.8502E-89 | 6.9203E-88 |
| AT4G12480 | -2.546096252 | 2.551E-150 | 1.853E-148 |
| AT4G12490 | -1.557114537 | 0.00915033 | 0.01755141 |
| AT4G12500 | -2.642089897 | 2.8554E-09 | 1.1537E-08 |
| AT4G12510 | -4.482801799 | 1.6254E-07 | 5.6791E-07 |
| AT4G12520 | -3.664636904 | 5.9513E-05 | 0.00015969 |
| AT4G12560 | 1.113928032 | 6.4574E-30 | 6.8912E-29 |
| AT4G12570 | 1.202130405 | 1.3087E-20 | 9.8484E-20 |
| AT4G12580 | 5.211860108 | 8.8674E-93 | 3.4525E-91 |
| AT4G12600 | -1.259948499 | 3.7428E-50 | 6.8924E-49 |
| AT4G12680 | 2.069547761 | 2.3367E-25 | 2.1241E-24 |
| AT4G12690 | 1.105470772 | 0.00790759 | 0.01535748 |
| AT4G12730 | -2.714410058 | 2.271E-159 | 1.799E-157 |
| AT4G12735 | -2.818217648 | 1.6754E-05 | 4.7971E-05 |
| AT4G12830 | -1.826567342 | 7.912E-51 | 1.4809E-49 |
| AT4G12880 | -1.507795741 | 4.5994E-47 | 7.8255E-46 |
| AT4G12900 | -1.73880706 | 2.5151E-11 | 1.1678E-10 |
| AT4G12917 | -1.57924332 | 6.1757E-07 | 2.0359E-06 |
| AT4G12980 | -1.712848992 | 6.9189E-17 | 4.4034E-16 |
| AT4G13010 | 1.034107875 | 3.8842E-36 | 5.0013E-35 |
| AT4G13110 | 2.157995634 | 3.074E-31 | 3.408E-30 |
| AT4G13120 | -1.490483017 | 1.6824E-11 | 7.887E-11 |
| AT4G13130 | -4.307691198 | 2.6382E-07 | 9.0338E-07 |
| AT4G13170 | -1.340062261 | 7.7866E-48 | 1.35E-46 |
| AT4G13235 | -2.17486651 | 1.1278E-12 | 5.6979E-12 |
| AT4G13340 | -1.961322038 | 1.6947E-90 | 6.4337E-89 |
| AT4G13345 | 1.375301619 | 3.2782E-21 | 2.5269E-20 |
| AT4G13390 | -6.544239672 | 1.3644E-30 | 1.4824E-29 |
| AT4G13410 | -1.433067878 | 5.8839E-11 | 2.6742E-10 |
| AT4G13420 | -1.842907479 | 1.1231E-05 | 3.2727E-05 |
| AT4G13440 | -2.8171009 | 0.00401985 | 0.00824274 |
| AT4G13480 | 7.470412202 | 7.067E-06 | 2.1009E-05 |
| AT4G13495 | -1.17231109 | 2.927E-29 | 3.0747E-28 |
| AT4G13530 | 1.465651071 | 2.1276E-49 | 3.8436E-48 |
| AT4G13572 | -1.07624175 | 0.00419796 | 0.00857788 |
| AT4G06120 | -4.091135081 | 0.00023541 | 0.0005833 |
| AT4G13575 | -1.704201938 | 1.77E-08 | 6.7298E-08 |
| AT4G13580 | -3.669732658 | 1.0826E-13 | 5.8337E-13 |
| AT4G13620 | -4.526920721 | 4.3419E-08 | 1.595E-07 |
| AT4G13660 | -2.372379222 | 1.3697E-41 | 2.0372E-40 |
| AT4G13690 | -1.333428211 | 0.00428882 | 0.00875178 |
| AT4G13770 | -3.705832951 | 5.2604E-76 | 1.5567E-74 |
| AT4G13800 | 3.235502369 | 4.5132E-28 | 4.5543E-27 |
| AT4G13830 | 2.11834306 | 2.9908E-92 | 1.1566E-90 |
| AT4G13885 | 1.638419242 | 0.00267056 | 0.005646 |
| AT4G13890 | -3.037505292 | 0.00364698 | 0.00752959 |
| AT4G13930 | -1.626010469 | 5.648E-174 | 5.093E-172 |
| AT4G14020 | 1.593706421 | 3.3516E-28 | 3.4017E-27 |
| AT4G14040 | -2.615107635 | 3.092E-265 | 5.075E-263 |
| AT4G14050 | -1.061240008 | 7.2169E-05 | 0.00019156 |
| AT4G14090 | 1.022431164 | 6.538E-15 | 3.7746E-14 |
| AT4G14120 | -1.673508171 | 0.00012907 | 0.00033263 |
| AT4G14130 | -2.815214249 | 5.6122E-11 | 2.5538E-10 |
| AT4G14220 | 1.107248064 | 7.5323E-20 | 5.5118E-19 |
| AT4G14270 | 2.204246508 | 1.289E-141 | 8.55E-140 |
| AT4G14380 | -2.273675501 | 0.00075297 | 0.00173551 |
| AT4G14400 | -2.991972084 | 1.2465E-13 | 6.6776E-13 |
| AT4G14465 | -2.102279854 | 2.1119E-28 | 2.154E-27 |
| AT4G14480 | -1.330060452 | 5.6392E-06 | 1.6952E-05 |
| AT4G14500 | 1.081018205 | 1.1349E-24 | 1.0099E-23 |
| AT4G14510 | -1.355027626 | 9.9656E-24 | 8.5544E-23 |
| AT4G06195 | -2.672902268 | 0.00086576 | 0.00197651 |
| AT4G14550 | -1.120600143 | 1.185E-07 | 4.1912E-07 |
| AT4G14590 | -1.084795062 | 0.00225687 | 0.00483545 |
| AT4G14630 | -1.393659161 | 5.0565E-16 | 3.0814E-15 |
| AT4G14640 | -1.693857765 | 0.00928629 | 0.01779125 |
| AT4G14650 | -2.3800741 | 8.7394E-07 | 2.8433E-06 |
| AT4G14680 | -1.078820293 | 2.3762E-21 | 1.8441E-20 |
| AT4G14746 | 2.232940418 | 4.5949E-75 | 1.3406E-73 |
| AT4G14750 | -3.27720262 | 1.5708E-19 | 1.141E-18 |
| AT4G14930 | 1.507206773 | 2.8859E-49 | 5.201E-48 |
| AT4G14940 | -1.84445296 | 5.7684E-06 | 1.7304E-05 |
| AT4G15070 | -1.434841313 | 0.00055946 | 0.00131162 |
| AT4G15120 | 2.93260211 | 1.3055E-42 | 2.0072E-41 |
| AT4G15210 | 3.530150416 | 4.6754E-86 | 1.6462E-84 |
| AT4G15230 | -1.211377303 | 1.1728E-09 | 4.9001E-09 |
| AT4G15248 | 3.509343452 | 3.3783E-08 | 1.2549E-07 |
| AT4G15290 | -3.900769174 | 0.00044839 | 0.00106723 |
| AT4G15320 | -2.903963147 | 1.2889E-08 | 4.945E-08 |
| AT4G15340 | -2.182342167 | 2.7983E-06 | 8.6524E-06 |
| AT4G15350 | -2.607653822 | 0.00168969 | 0.00368918 |
| AT4G15380 | -2.067687721 | 0.00086274 | 0.00197 |
| AT4G15390 | -3.562595947 | 2.8378E-81 | 9.1066E-80 |
| AT4G15393 | -3.824362861 | 1.0872E-39 | 1.5417E-38 |
| AT4G15396 | -1.87457682 | 0.00200962 | 0.00433501 |
| AT4G15420 | 1.366922064 | 3.1603E-24 | 2.7701E-23 |
| AT4G15480 | -3.7655196 | 8.043E-167 | 6.847E-165 |
| AT4G15490 | 1.207112322 | 2.7963E-55 | 5.7322E-54 |
| AT4G15500 | -1.636725857 | 0.00541603 | 0.01085299 |
| AT4G15530 | 2.287764774 | 2.389E-150 | 1.747E-148 |
| AT4G15550 | -1.101518863 | 2.9582E-29 | 3.1032E-28 |
| AT4G15640 | -1.317483008 | 1.5839E-22 | 1.2976E-21 |
| AT4G15680 | -2.292673893 | 1.0551E-08 | 4.0809E-08 |
| AT4G15690 | -3.211436258 | 3.4305E-13 | 1.7846E-12 |
| AT4G15700 | -2.092381484 | 5.2905E-09 | 2.0942E-08 |
| AT4G15710 | -3.376450706 | 0.00473965 | 0.00960048 |
| AT4G15770 | -1.155188169 | 4.7481E-28 | 4.7892E-27 |
| AT4G15810 | -1.263731846 | 1.6593E-13 | 8.8144E-13 |
| AT4G15820 | -1.485486751 | 1.6971E-06 | 5.3563E-06 |
| AT4G15830 | -1.370218059 | 1.9374E-14 | 1.0885E-13 |
| AT4G15910 | 2.446116578 | 6.609E-221 | 8.107E-219 |
| AT4G15975 | -1.988688179 | 0.00230315 | 0.00492627 |
| AT4G15990 | 2.046137953 | 1.5537E-07 | 5.4437E-07 |
| AT4G16000 | 2.681791274 | 7.382E-14 | 4.0178E-13 |
| AT4G16008 | 1.428012218 | 7.0415E-06 | 2.0938E-05 |
| AT4G16110 | 1.131088251 | 2.5076E-17 | 1.6347E-16 |
| AT4G16140 | -2.618507799 | 2.3753E-44 | 3.8058E-43 |
| AT4G16141 | -1.14359385 | 1.3528E-20 | 1.0166E-19 |
| AT4G16160 | 7.285644462 | 2.1531E-05 | 6.0912E-05 |
| AT4G16190 | 1.531024165 | 3.614E-188 | 3.6E-186 |
| AT4G16220 | -7.490471143 | 6.5524E-06 | 1.9548E-05 |
| AT4G16230 | -3.128980972 | 0.00052577 | 0.00123771 |
| AT4G16260 | -1.154371088 | 6.2027E-31 | 6.8137E-30 |
| AT4G16270 | -1.758381175 | 0.00565195 | 0.01128017 |
| AT4G16350 | -1.700258567 | 0.00586795 | 0.01167857 |
| AT4G16390 | -1.69893046 | 4.0803E-58 | 8.9775E-57 |
| AT4G16442 | 1.170063113 | 3.503E-21 | 2.6956E-20 |
| AT4G16520 | 1.367619485 | 1.0057E-53 | 2.0005E-52 |
| AT4G16540 | 1.453670939 | 0.000159 | 0.00040408 |
| AT4G16545 | 3.224027357 | 0.00223481 | 0.00479585 |
| AT4G16563 | -3.091031238 | 1.5442E-27 | 1.5265E-26 |
| AT4G16590 | -1.72853426 | 0.00796098 | 0.01545195 |
| AT4G16620 | 1.482401945 | 0.0020588 | 0.00443733 |
| AT4G16690 | 1.694002139 | 2.5489E-16 | 1.5743E-15 |
| AT4G16700 | -1.462316544 | 5.5345E-07 | 1.8341E-06 |
| AT4G16740 | 6.486526285 | 0.00048291 | 0.00114379 |
| AT4G16745 | 1.410323592 | 0.00054415 | 0.00127783 |
| AT4G16750 | 1.197283298 | 1.7619E-07 | 6.1299E-07 |
| AT4G16760 | 2.129996295 | 8.064E-160 | 6.456E-158 |
| AT4G16765 | 1.491069368 | 4.2548E-33 | 4.9937E-32 |
| AT4G16835 | -1.272498724 | 6.6791E-06 | 1.9902E-05 |
| AT4G16845 | 1.001489449 | 1.8401E-17 | 1.2064E-16 |
| AT4G16860 | -1.29700799 | 9.6227E-22 | 7.5917E-21 |
| AT4G16880 | -2.278603389 | 3.1636E-18 | 2.1605E-17 |
| AT4G16990 | -1.175374738 | 1.3414E-25 | 1.2297E-24 |
| AT4G17030 | 2.367841205 | 7.565E-136 | 4.69E-134 |
| AT4G17090 | -2.207109607 | 2.142E-184 | 2.08E-182 |
| AT4G17220 | -1.120231158 | 0.00527485 | 0.01059708 |
| AT4G17230 | 1.61119173 | 3.7601E-80 | 1.1833E-78 |
| AT4G17245 | 2.255635128 | 5.0208E-49 | 8.9846E-48 |
| AT4G17250 | 1.080449511 | 8.7426E-10 | 3.6918E-09 |
| AT4G17280 | 1.930614325 | 1.9871E-32 | 2.2905E-31 |
| AT4G17460 | -2.335407259 | 6.6575E-27 | 6.4092E-26 |
| AT4G17500 | -1.220607249 | 9.8172E-15 | 5.6025E-14 |
| AT4G17520 | -1.15542199 | 2.5304E-77 | 7.6166E-76 |
| AT4G17530 | 1.15923233 | 5.438E-56 | 1.1383E-54 |
| AT4G17540 | -1.010167907 | 5.6227E-06 | 1.6905E-05 |
| AT4G17550 | 2.587339871 | 1.271E-128 | 7.472E-127 |
| AT4G17650 | 2.035481566 | 8.836E-106 | 4.016E-104 |
| AT4G17690 | 4.056663056 | 0.00903159 | 0.01734318 |
| AT4G17730 | 1.278032471 | 4.9547E-35 | 6.1305E-34 |
| AT4G17785 | 2.363536906 | 0.00136508 | 0.00302033 |
| AT4G17790 | 1.022617232 | 4.5409E-09 | 1.8097E-08 |
| AT4G17800 | -1.65357612 | 1.1587E-13 | 6.2263E-13 |
| AT4G17810 | -2.110550288 | 3.7705E-15 | 2.2002E-14 |
| AT4G17840 | 2.203340657 | 1.356E-205 | 1.516E-203 |
| AT4G17880 | -1.454667468 | 2.8671E-18 | 1.9603E-17 |
| AT4G17970 | 1.719960805 | 3.0038E-23 | 2.5327E-22 |
| AT4G18030 | -1.438003612 | 7.1051E-74 | 2.034E-72 |
| AT4G18140 | 1.553977554 | 1.0497E-30 | 1.1443E-29 |
| AT4G18170 | 2.328160931 | 1.1201E-20 | 8.4655E-20 |
| AT4G18197 | -2.723796349 | 3.2351E-12 | 1.585E-11 |
| AT4G18205 | -2.572928051 | 7.2295E-22 | 5.7393E-21 |
| AT4G18210 | 2.607879229 | 3.7723E-24 | 3.2926E-23 |
| AT4G18250 | -3.114399476 | 0.00166555 | 0.00364041 |
| AT4G18260 | -1.759283765 | 0.00274583 | 0.00579277 |
| AT4G18280 | 2.105398121 | 2.6638E-76 | 7.9241E-75 |
| AT4G18290 | -1.389437288 | 5.6409E-09 | 2.2267E-08 |
| AT4G18300 | 1.033327028 | 1.4479E-07 | 5.0885E-07 |
| AT4G18425 | 1.472769576 | 6.6239E-06 | 1.975E-05 |
| AT4G18430 | -2.733789425 | 1.712E-14 | 9.6542E-14 |
| AT4G18470 | -1.239553884 | 0.00731686 | 0.01430273 |
| AT4G18460 | -1.236447511 | 3.906E-06 | 1.1909E-05 |
| AT4G18510 | -2.869866434 | 2.0091E-05 | 5.7057E-05 |
| AT4G18530 | 1.009552486 | 5.1334E-09 | 2.0337E-08 |
| AT4G18550 | -1.684561539 | 0.00247838 | 0.00526652 |
| AT4G18610 | -1.687889692 | 0.00014679 | 0.00037492 |
| AT4G18670 | -1.010650481 | 3.93E-25 | 3.5385E-24 |
| AT4G18700 | 1.649816379 | 5.1683E-94 | 2.0508E-92 |
| AT4G18730 | -1.103076904 | 1.4827E-59 | 3.3297E-58 |
| AT4G18760 | -1.875978242 | 3.4362E-19 | 2.4523E-18 |
| AT4G18830 | 1.551780459 | 0.00587562 | 0.01169078 |
| AT4G18905 | -1.315289155 | 2.1458E-35 | 2.693E-34 |
| AT4G18940 | -3.64190607 | 1.0535E-07 | 3.7475E-07 |
| AT4G18980 | 6.404690797 | 0.00017734 | 0.00044718 |
| AT4G18990 | 2.771052 | 0.00162214 | 0.00355293 |
| AT4G19020 | -1.440206218 | 3.7728E-27 | 3.6661E-26 |
| AT4G19120 | -1.546178172 | 5.9664E-70 | 1.6053E-68 |
| AT4G19230 | 2.574813102 | 2.1862E-89 | 8.1503E-88 |
| AT4G19380 | -4.239968935 | 6.0777E-37 | 7.997E-36 |
| AT4G19390 | 2.234986276 | 4.667E-107 | 2.16E-105 |
| AT4G06880 | 1.904914971 | 5.2722E-07 | 1.7512E-06 |
| AT4G19460 | -1.606350513 | 3.0092E-05 | 8.3667E-05 |
| AT4G19530 | -1.527572138 | 4.1011E-39 | 5.7195E-38 |
| AT4G19590 | -1.995883684 | 0.00510159 | 0.01027615 |
| AT4G19640 | 1.182444739 | 1.6752E-45 | 2.7516E-44 |
| AT4G19680 | -2.50100523 | 0.00096484 | 0.0021852 |
| AT4G19720 | -1.365898723 | 0.00636492 | 0.01258424 |
| AT4G19810 | -1.166027544 | 1.7871E-16 | 1.1122E-15 |
| AT4G19860 | 1.597505552 | 5.1952E-86 | 1.8236E-84 |
| AT4G19880 | -1.005286919 | 2.1725E-38 | 2.9842E-37 |
| AT4G19970 | -2.291809721 | 0.00085026 | 0.00194364 |
| AT4G19980 | -4.200704644 | 0.0040341 | 0.00826899 |
| AT4G20000 | -1.657817386 | 0.00082218 | 0.00188418 |
| AT4G20070 | 1.567581094 | 1.3365E-49 | 2.4239E-48 |
| AT4G20140 | -1.279505783 | 8.5037E-08 | 3.0497E-07 |
| AT4G20160 | -5.783505316 | 0.00541349 | 0.0108498 |
| AT4G20190 | -4.155998047 | 0.00663371 | 0.01307519 |
| AT4G20230 | -3.086615719 | 6.8974E-13 | 3.5372E-12 |
| AT4G20235 | -2.009670796 | 0.00301114 | 0.00630642 |
| AT4G20240 | -1.701354 | 2.9768E-11 | 1.3754E-10 |
| AT4G20270 | -1.093620179 | 3.2082E-31 | 3.5515E-30 |
| AT4G20320 | 1.212651013 | 3.8783E-36 | 4.9965E-35 |
| AT4G20370 | 2.372065794 | 0.00290328 | 0.00609227 |
| AT4G20390 | -1.854444855 | 3.9991E-15 | 2.3312E-14 |
| AT4G20410 | 1.083173349 | 6.1319E-24 | 5.3216E-23 |
| AT4G20430 | -1.169928714 | 1.2592E-11 | 5.9595E-11 |
| AT4G20740 | -1.176674211 | 3.9782E-08 | 1.4671E-07 |
| AT4G20780 | -1.414106747 | 4.6606E-12 | 2.2615E-11 |
| AT4G20840 | -1.113015424 | 2.4339E-11 | 1.131E-10 |
| AT4G20860 | -1.292308398 | 5.9723E-49 | 1.0662E-47 |
| AT4G20880 | 1.46273418 | 1.1744E-31 | 1.3213E-30 |
| AT4G20930 | 1.110389686 | 1.2562E-10 | 5.5938E-10 |
| AT4G20940 | -2.066757315 | 6.1997E-38 | 8.4348E-37 |
| AT4G20960 | -1.082839941 | 3.9265E-18 | 2.6655E-17 |
| AT4G21020 | 7.47085928 | 4.0318E-32 | 4.5949E-31 |
| AT4G21060 | 1.463984677 | 8.652E-22 | 6.8377E-21 |
| AT4G21065 | -1.235083242 | 0.0003221 | 0.00078273 |
| AT4G21160 | 1.11944362 | 4.7942E-20 | 3.5365E-19 |
| AT4G21170 | -1.144632917 | 0.00054925 | 0.00128923 |
| AT4G21215 | 1.397466174 | 7.0163E-27 | 6.7518E-26 |
| AT4G21230 | -2.447853721 | 2.0051E-09 | 8.228E-09 |
| AT4G21320 | 3.054380706 | 2.1107E-74 | 6.0806E-73 |
| AT4G21323 | 3.628939851 | 5.5118E-17 | 3.5266E-16 |
| AT4G21340 | -3.058121937 | 0.00017347 | 0.0004381 |
| AT4G21410 | -1.412972575 | 2.0693E-19 | 1.4927E-18 |
| AT4G21440 | 5.076665303 | 8.6607E-82 | 2.8069E-80 |
| AT4G21480 | 1.380258876 | 0.00014831 | 0.00037859 |
| AT4G21534 | 1.324478439 | 1.6107E-18 | 1.1167E-17 |
| AT4G21540 | 1.052825063 | 6.9755E-19 | 4.9167E-18 |
| AT4G21550 | 1.05353941 | 0.00014649 | 0.00037423 |
| AT4G21560 | 1.25395773 | 1.7158E-22 | 1.4026E-21 |
| AT4G21570 | 1.937922277 | 6.748E-126 | 3.802E-124 |
| AT4G21580 | 1.346882108 | 1.4345E-70 | 3.887E-69 |
| AT4G21600 | -1.684181917 | 6.227E-14 | 3.4071E-13 |
| AT4G21620 | 1.023318173 | 2.9313E-29 | 3.0778E-28 |
| AT4G21650 | 1.374448178 | 8.5284E-39 | 1.1807E-37 |
| AT4G21680 | 1.465805171 | 3.5257E-12 | 1.7232E-11 |
| AT4G21705 | -1.696625029 | 2.8515E-13 | 1.4915E-12 |
| AT4G21760 | -2.231940476 | 1.2531E-06 | 4.0092E-06 |
| AT4G21770 | -1.140373415 | 1.398E-15 | 8.3411E-15 |
| AT4G21820 | -1.053439181 | 2.9996E-05 | 8.341E-05 |
| AT4G21830 | -1.62418875 | 3.3391E-05 | 9.2346E-05 |
| AT4G21850 | -2.468900211 | 4.6937E-29 | 4.9034E-28 |
| AT4G21870 | -1.845637204 | 7.4678E-16 | 4.5087E-15 |
| AT4G21880 | -1.261030714 | 6.7423E-10 | 2.8727E-09 |
| AT4G21890 | -1.192003043 | 0.00011275 | 0.0002924 |
| AT4G21903 | -2.737037124 | 9.359E-08 | 3.3433E-07 |
| AT4G21910 | 1.596741521 | 4.554E-139 | 2.911E-137 |
| AT4G21930 | 3.286325284 | 1.9853E-10 | 8.7393E-10 |
| AT4G21980 | 1.644597685 | 3.1023E-57 | 6.7092E-56 |
| AT4G22010 | -1.72435402 | 8.4685E-71 | 2.3084E-69 |
| AT4G22070 | -1.349659736 | 6.1559E-10 | 2.6292E-09 |
| AT4G22080 | -4.367560818 | 0.0082926 | 0.01604783 |
| AT4G22130 | -1.056470763 | 1.5067E-19 | 1.0952E-18 |
| AT4G22220 | 1.22546295 | 2.3673E-71 | 6.492E-70 |
| AT4G22230 | -1.419239638 | 0.00437273 | 0.00891026 |
| AT4G22240 | 2.165123092 | 2.67E-230 | 3.462E-228 |
| AT4G22270 | 2.409518739 | 4.0541E-35 | 5.0325E-34 |
| AT4G22560 | -1.246871978 | 0.00637305 | 0.01259813 |
| AT4G22590 | 1.497119401 | 1.0664E-51 | 2.0499E-50 |
| AT4G22610 | -3.921134409 | 1.3836E-23 | 1.1832E-22 |
| AT4G22666 | -5.580650765 | 7.6851E-20 | 5.6218E-19 |
| AT4G22690 | -2.735744166 | 9.917E-103 | 4.376E-101 |
| AT4G22710 | -4.498903676 | 5.825E-17 | 3.7217E-16 |
| AT4G22740 | 1.229195652 | 3.1768E-34 | 3.8249E-33 |
| AT4G22745 | 1.729562938 | 4.6571E-39 | 6.4791E-38 |
| AT4G22780 | 1.050998659 | 1.2287E-09 | 5.1241E-09 |
| AT4G22790 | -1.12017412 | 0.00571407 | 0.01139517 |
| AT4G22810 | -1.601958244 | 2.8623E-09 | 1.1559E-08 |
| AT4G22820 | 2.371896265 | 3.91E-182 | 3.702E-180 |
| AT4G22880 | 1.245594168 | 1.3637E-41 | 2.0296E-40 |
| AT4G22920 | 2.398865881 | 1.4643E-94 | 5.8408E-93 |
| AT4G22950 | 2.201063047 | 2.9089E-14 | 1.62E-13 |
| AT4G22960 | 3.804350467 | 3.3725E-34 | 4.0562E-33 |
| AT4G23040 | 1.010713992 | 5.2941E-13 | 2.726E-12 |
| AT4G23050 | 3.212582612 | 0 | 0 |
| AT4G23070 | -6.95067257 | 7.3178E-05 | 0.00019405 |
| AT4G23130 | -2.033428459 | 0.00516367 | 0.01039033 |
| AT4G23220 | 1.255989382 | 9.4848E-07 | 3.0753E-06 |
| AT4G23260 | -1.308012225 | 3.4235E-12 | 1.6741E-11 |
| AT4G23270 | -1.561919933 | 3.7685E-27 | 3.6651E-26 |
| AT4G23280 | -2.826161816 | 0.00203197 | 0.00438158 |
| AT4G23290 | -1.110034394 | 1.4146E-06 | 4.5002E-06 |
| AT4G23300 | -1.916849054 | 3.9113E-18 | 2.656E-17 |
| AT4G23340 | -3.44395698 | 0.00726697 | 0.01421495 |
| AT4G23400 | -1.238433312 | 7.268E-39 | 1.0068E-37 |
| AT4G23450 | 3.013442361 | 6.4873E-53 | 1.2716E-51 |
| AT4G23496 | -3.610609571 | 3.7283E-40 | 5.3635E-39 |
| AT4G23510 | -2.601878643 | 9.8163E-09 | 3.8077E-08 |
| AT4G23540 | -1.114740824 | 2.3861E-16 | 1.4763E-15 |
| AT4G23550 | -1.326544192 | 1.4066E-08 | 5.3803E-08 |
| AT4G23590 | 1.414352782 | 0.00703025 | 0.01379092 |
| AT4G23600 | 2.080330321 | 2.5022E-30 | 2.6916E-29 |
| AT4G23630 | 1.306693672 | 1.8386E-98 | 7.74E-97 |
| AT4G23690 | -1.585080278 | 2.2012E-21 | 1.71E-20 |
| AT4G23700 | -2.563420814 | 1.6304E-37 | 2.1946E-36 |
| AT4G23730 | 1.224598167 | 2.8082E-25 | 2.5405E-24 |
| AT4G23750 | -1.047427676 | 8.9689E-14 | 4.8595E-13 |
| AT4G23770 | -1.753524069 | 9.3113E-06 | 2.7345E-05 |
| AT4G23800 | -1.378376629 | 1.5717E-35 | 1.9889E-34 |
| AT4G23820 | -2.203725013 | 1.7004E-77 | 5.1455E-76 |
| AT4G23870 | 3.055507771 | 3.5032E-82 | 1.1418E-80 |
| AT4G23880 | 2.91963693 | 3.6579E-39 | 5.1138E-38 |
| AT4G23920 | 1.051924809 | 3.7204E-17 | 2.4006E-16 |
| AT4G24000 | 8.685036628 | 1.6921E-45 | 2.7774E-44 |
| AT4G24120 | -1.328716809 | 6.0662E-14 | 3.3247E-13 |
| AT4G24130 | 1.589882682 | 6.0036E-41 | 8.8146E-40 |
| AT4G24175 | -1.217446614 | 3.5084E-09 | 1.4083E-08 |
| AT4G24190 | -1.115257024 | 1.3346E-72 | 3.7364E-71 |
| AT4G24220 | 1.054603286 | 1.5824E-65 | 3.9982E-64 |
| AT4G24275 | -1.298906582 | 1.5096E-05 | 4.3433E-05 |
| AT4G24310 | -1.627107961 | 0.00160709 | 0.00352402 |
| AT4G24340 | -1.942096168 | 1.1302E-12 | 5.7087E-12 |
| AT4G24350 | -1.652991886 | 2.1093E-34 | 2.5572E-33 |
| AT4G24380 | 1.284383095 | 7.969E-08 | 2.866E-07 |
| AT4G24400 | 1.42498482 | 1.6222E-58 | 3.583E-57 |
| AT4G24410 | 3.91751894 | 4.1277E-37 | 5.4659E-36 |
| AT4G24413 | 4.04777759 | 1.486E-105 | 6.715E-104 |
| AT4G24430 | 1.562298148 | 0.00070234 | 0.00162324 |
| AT4G24450 | 1.185110622 | 8.5659E-20 | 6.2581E-19 |
| AT4G24480 | 1.533090427 | 1.0273E-31 | 1.1592E-30 |
| AT4G24510 | 1.630076806 | 4.9173E-61 | 1.1367E-59 |
| AT4G24660 | -1.086393235 | 1.6702E-11 | 7.8347E-11 |
| AT4G24670 | -1.459480576 | 1.6748E-11 | 7.8544E-11 |
| AT4G24810 | -1.181196068 | 6.2109E-11 | 2.8167E-10 |
| AT4G24830 | -1.184697406 | 1.8306E-54 | 3.7028E-53 |
| AT4G24960 | 3.016490481 | 0 | 0 |
| AT4G25050 | -2.082654156 | 8.964E-155 | 6.863E-153 |
| AT4G25070 | -1.58367706 | 6.3124E-07 | 2.0789E-06 |
| AT4G25080 | -1.058489137 | 4.5729E-57 | 9.8152E-56 |
| AT4G25090 | -2.414376847 | 3.1946E-20 | 2.3703E-19 |
| AT4G25110 | -3.931823871 | 1.9076E-11 | 8.9171E-11 |
| AT4G25160 | -1.397541477 | 0.00685958 | 0.01348744 |
| AT4G25220 | -3.156711142 | 0.00232704 | 0.00497318 |
| AT4G25240 | -1.544089565 | 3.4007E-15 | 1.988E-14 |
| AT4G25250 | -3.196370543 | 1.0223E-06 | 3.3015E-06 |
| AT4G25260 | -1.888855852 | 9.5057E-23 | 7.8467E-22 |
| AT4G25270 | -1.06346327 | 2.1345E-05 | 6.0409E-05 |
| AT4G25310 | -1.322529635 | 0.00328782 | 0.00684255 |
| AT4G25315 | -1.157491551 | 0.00202387 | 0.00436535 |
| AT4G25340 | -1.146455139 | 2.7016E-35 | 3.3794E-34 |
| AT4G25380 | 2.162684187 | 1.2002E-05 | 3.4881E-05 |
| AT4G25400 | -1.564310266 | 4.2422E-05 | 0.00011599 |
| AT4G25410 | -1.616475581 | 0.00262981 | 0.00556706 |
| AT4G25433 | 4.084968323 | 1.4077E-24 | 1.2492E-23 |
| AT4G25480 | 1.895564922 | 7.2799E-10 | 3.0948E-09 |
| AT4G03125 | 5.992934631 | 0.00258161 | 0.00547263 |
| AT4G25490 | 5.100994899 | 5.8144E-08 | 2.1151E-07 |
| AT4G25580 | 6.015525964 | 0.00025337 | 0.00062571 |
| AT4G25630 | -1.219219095 | 8.6052E-73 | 2.4209E-71 |
| AT4G25650 | 1.635669353 | 1.4015E-94 | 5.6001E-93 |
| AT4G25670 | 2.390427492 | 9.505E-202 | 1.038E-199 |
| AT4G25690 | 2.474046315 | 2.185E-129 | 1.291E-127 |
| AT4G25730 | -1.243713196 | 5.0874E-63 | 1.2218E-61 |
| AT4G25740 | -1.044795826 | 2.9031E-52 | 5.6372E-51 |
| AT4G25780 | -1.034920986 | 0.00241797 | 0.00514967 |
| AT4G25790 | -3.928195373 | 2.3198E-08 | 8.7412E-08 |
| AT4G25800 | 1.202387642 | 0.00604037 | 0.01199452 |
| AT4G25820 | -2.355465236 | 4.1383E-19 | 2.9433E-18 |
| AT4G25850 | 2.569606614 | 1.6172E-41 | 2.4022E-40 |
| AT4G25870 | -1.149116295 | 3.2538E-12 | 1.5935E-11 |
| AT4G25890 | -1.295678162 | 2.305E-31 | 2.568E-30 |
| AT4G26010 | -2.121237335 | 2.8893E-18 | 1.9749E-17 |
| AT4G26080 | 3.58696658 | 0 | 0 |
| AT4G26120 | -1.392152604 | 0.00018193 | 0.0004576 |
| AT4G26150 | -1.916669202 | 3.0502E-20 | 2.2654E-19 |
| AT4G26160 | 1.005265656 | 1.3212E-07 | 4.6597E-07 |
| AT4G26180 | 1.718661405 | 9.3166E-15 | 5.3289E-14 |
| AT4G26200 | 1.901630796 | 2.5381E-07 | 8.7069E-07 |
| AT4G26220 | -1.300930731 | 7.7615E-08 | 2.7945E-07 |
| AT4G26320 | -1.499549604 | 2.8203E-05 | 7.8722E-05 |
| AT4G26370 | -1.721691798 | 2.8722E-84 | 9.7084E-83 |
| AT4G26400 | 1.879745969 | 2.9432E-40 | 4.2528E-39 |
| AT4G26540 | -1.903394736 | 1.7461E-17 | 1.1464E-16 |
| AT4G26600 | -1.350608834 | 3.3024E-26 | 3.102E-25 |
| AT4G26660 | -1.031807134 | 3.4848E-09 | 1.3995E-08 |
| AT4G26670 | -1.153618355 | 1.2951E-39 | 1.8309E-38 |
| AT4G26750 | 1.130803506 | 1.558E-18 | 1.0817E-17 |
| AT4G26780 | -1.94539742 | 9.6507E-44 | 1.5217E-42 |
| AT4G27010 | -1.663799135 | 2.3424E-25 | 2.1284E-24 |
| AT4G27020 | 1.384289396 | 1.0371E-25 | 9.564E-25 |
| AT4G27100 | 1.044553732 | 2.3451E-09 | 9.5631E-09 |
| AT4G27260 | 1.209993732 | 1.5374E-19 | 1.1171E-18 |
| AT4G27300 | -2.697065278 | 5.7981E-28 | 5.8303E-27 |
| AT4G27340 | -1.221364057 | 4.2658E-17 | 2.7471E-16 |
| AT4G27350 | 1.764913839 | 2.3733E-26 | 2.2403E-25 |
| AT4G27400 | -2.373701463 | 0.00011089 | 0.0002879 |
| AT4G27410 | 5.424051863 | 0 | 0 |
| AT4G07815 | 1.555238817 | 0.00020465 | 0.00051138 |
| AT4G27440 | -1.632450103 | 6.407E-127 | 3.664E-125 |
| AT4G27450 | 1.803765835 | 1.2571E-08 | 4.8295E-08 |
| AT4G27460 | 2.132493944 | 0.00285667 | 0.00600331 |
| AT4G27520 | 1.756469994 | 2.738E-189 | 2.789E-187 |
| AT4G27530 | 4.218344638 | 2.0047E-19 | 1.4479E-18 |
| AT4G27580 | 6.81214024 | 0.0001209 | 0.00031245 |
| AT4G27652 | 1.474999058 | 9.082E-11 | 4.0823E-10 |
| AT4G27657 | 2.166121329 | 6.6755E-24 | 5.778E-23 |
| AT4G27670 | 7.473103072 | 2.7589E-07 | 9.4275E-07 |
| AT4G27720 | -1.182768911 | 2.2427E-27 | 2.198E-26 |
| AT4G27780 | 1.319471616 | 2.6211E-17 | 1.7058E-16 |
| AT4G27830 | 1.416618764 | 2.4674E-85 | 8.5426E-84 |
| AT4G27840 | 2.059804009 | 1.5625E-45 | 2.5685E-44 |
| AT4G27860 | -1.560653029 | 1.0148E-35 | 1.2913E-34 |
| AT4G27940 | 1.388985474 | 2.1133E-22 | 1.7189E-21 |
| AT4G27970 | -1.993508051 | 1.9102E-05 | 5.4351E-05 |
| AT4G28080 | -1.560088599 | 2.724E-122 | 1.49E-120 |
| AT4G28085 | 1.178621962 | 0.00789461 | 0.01533618 |
| AT4G28110 | 3.235501247 | 1.3713E-23 | 1.1731E-22 |
| AT4G28140 | 4.786135412 | 6.103E-105 | 2.741E-103 |
| AT4G28150 | 1.692322183 | 1.9249E-06 | 6.042E-06 |
| AT4G28210 | -1.01458189 | 4.0754E-20 | 3.0141E-19 |
| AT4G28240 | 2.328703999 | 2.196E-119 | 1.165E-117 |
| AT4G28250 | -3.512598199 | 2.062E-245 | 3.076E-243 |
| AT4G28300 | 1.329305594 | 1.8534E-53 | 3.6612E-52 |
| AT4G28310 | -1.79409973 | 2.122E-15 | 1.2547E-14 |
| AT4G28360 | -1.180411634 | 2.353E-22 | 1.9099E-21 |
| AT4G28410 | -1.420885251 | 0.00014259 | 0.00036505 |
| AT4G28420 | -6.360437859 | 0.00082995 | 0.00190065 |
| AT4G28490 | 1.698798876 | 8.0126E-36 | 1.0225E-34 |
| AT4G28650 | -1.97881769 | 0.00061235 | 0.00142754 |
| AT4G28660 | -1.102797029 | 8.6404E-51 | 1.6146E-49 |
| AT4G28680 | -2.113118811 | 9.2085E-06 | 2.7064E-05 |
| AT4G28700 | 3.6881156 | 2.6828E-07 | 9.177E-07 |
| AT4G28703 | 3.943237039 | 1.9756E-32 | 2.2788E-31 |
| AT4G28780 | -2.188900598 | 1.7583E-68 | 4.6433E-67 |
| AT4G28930 | -5.66853383 | 0.00851157 | 0.01643256 |
| AT4G28940 | -3.066387576 | 6.5933E-08 | 2.389E-07 |
| AT4G29050 | -1.691143227 | 0.00018186 | 0.00045749 |
| AT4G29060 | -1.369550523 | 1.338E-139 | 8.624E-138 |
| AT4G29070 | 2.074805244 | 1.6973E-81 | 5.4774E-80 |
| AT4G29080 | -1.320057175 | 3.5143E-24 | 3.0733E-23 |
| AT4G29110 | 1.122889678 | 5.1519E-08 | 1.8816E-07 |
| AT4G29140 | -3.366546839 | 4.7206E-57 | 1.0123E-55 |
| AT4G29160 | 1.502189239 | 1.0346E-93 | 4.0839E-92 |
| AT4G29180 | -2.562556315 | 1.8937E-06 | 5.9487E-06 |
| AT4G29190 | 3.198999036 | 3.381E-240 | 4.676E-238 |
| AT4G29240 | -1.656330165 | 4.9372E-09 | 1.9594E-08 |
| AT4G29310 | -3.27696876 | 4.9373E-32 | 5.6072E-31 |
| AT4G29380 | 1.439974382 | 7.1882E-47 | 1.2158E-45 |
| AT4G29430 | -1.004548948 | 1.6283E-08 | 6.2044E-08 |
| AT4G29610 | -2.592444546 | 8.8802E-07 | 2.8878E-06 |
| AT4G29700 | -1.342465514 | 7.5484E-41 | 1.1047E-39 |
| AT4G29720 | -1.614148478 | 1.6042E-28 | 1.6457E-27 |
| AT4G29740 | -1.941363173 | 1.5055E-08 | 5.7479E-08 |
| AT4G29750 | -1.012877848 | 2.1666E-21 | 1.6843E-20 |
| AT4G29820 | 1.86165845 | 1.2586E-26 | 1.2005E-25 |
| AT4G29920 | -1.260379959 | 1.7491E-08 | 6.6546E-08 |
| AT4G29930 | 1.204933411 | 3.4601E-10 | 1.4995E-09 |
| AT4G29950 | 1.765393402 | 1.4257E-86 | 5.0668E-85 |
| AT4G30020 | -1.21438133 | 7.3231E-42 | 1.1014E-40 |
| AT4G30170 | -2.303512732 | 3.9811E-59 | 8.879E-58 |
| AT4G30180 | 1.802875233 | 2.8184E-05 | 7.8698E-05 |
| AT4G30190 | -2.115789057 | 3.398E-257 | 5.384E-255 |
| AT4G30250 | -2.433199123 | 6.6373E-10 | 2.8285E-09 |
| AT4G30270 | -1.98465586 | 2.6113E-54 | 5.2539E-53 |
| AT4G30280 | -2.421444246 | 8.0153E-19 | 5.627E-18 |
| AT4G30290 | 1.349965622 | 2.8605E-21 | 2.2146E-20 |
| AT4G30320 | -2.276032775 | 0.0007591 | 0.00174892 |
| AT4G30330 | -1.138470058 | 4.8889E-09 | 1.9423E-08 |
| AT4G30350 | 1.112679402 | 4.4053E-26 | 4.1125E-25 |
| AT4G30420 | -3.337688296 | 1.4087E-05 | 4.0664E-05 |
| AT4G30440 | 1.308156742 | 7.0728E-74 | 2.0273E-72 |
| AT4G30460 | 2.625302673 | 9.947E-187 | 9.782E-185 |
| AT4G30470 | 2.063163195 | 1.159E-183 | 1.116E-181 |
| AT4G30490 | 2.474308197 | 2.106E-168 | 1.82E-166 |
| AT4G30600 | 1.087425108 | 1.9305E-57 | 4.187E-56 |
| AT4G30610 | -3.464279318 | 1.9117E-65 | 4.8144E-64 |
| AT4G30620 | -1.181173511 | 2.7115E-19 | 1.9442E-18 |
| AT4G30630 | 1.045008475 | 2.249E-09 | 9.1876E-09 |
| AT4G30710 | 1.336077859 | 1.6397E-22 | 1.3414E-21 |
| AT4G30720 | -1.018663924 | 1.6479E-24 | 1.4595E-23 |
| AT4G30800 | -1.491437704 | 1.1776E-17 | 7.8034E-17 |
| AT4G30825 | -1.368791127 | 2.1029E-19 | 1.516E-18 |
| AT4G30830 | 2.890307281 | 1.6507E-18 | 1.1441E-17 |
| AT4G30850 | 1.374807289 | 2.5146E-24 | 2.2134E-23 |
| AT4G30940 | 1.142972887 | 2.6785E-05 | 7.4985E-05 |
| AT4G30960 | 2.664246823 | 0 | 0 |
| AT4G30980 | -1.565525312 | 2.3538E-13 | 1.2386E-12 |
| AT4G30990 | -1.163568078 | 4.8421E-37 | 6.3934E-36 |
| AT4G31000 | -1.106490018 | 1.3259E-06 | 4.2364E-06 |
| AT4G31140 | 1.335395733 | 5.8757E-23 | 4.8838E-22 |
| AT4G31210 | -2.153449508 | 4.3967E-88 | 1.6076E-86 |
| AT4G31240 | 1.032910802 | 3.0974E-05 | 8.5964E-05 |
| AT4G31290 | 2.073299341 | 3.899E-202 | 4.277E-200 |
| AT4G31320 | -2.091224836 | 1.1282E-10 | 5.0374E-10 |
| AT4G31355 | 2.554176624 | 0.006958 | 0.01365858 |
| AT4G31360 | -2.045532903 | 1.1042E-22 | 9.092E-22 |
| AT4G31400 | 1.293329479 | 1.7689E-05 | 5.048E-05 |
| AT4G31420 | 1.314835101 | 2.6525E-39 | 3.7243E-38 |
| AT4G31500 | -1.914766031 | 1.266E-152 | 9.597E-151 |
| AT4G31510 | 1.418863187 | 2.2282E-13 | 1.1744E-12 |
| AT4G31550 | 1.177207243 | 2.682E-31 | 2.9807E-30 |
| AT4G31600 | -1.631959528 | 9.6262E-16 | 5.7813E-15 |
| AT4G31670 | 1.103925112 | 3.6586E-18 | 2.4866E-17 |
| AT4G31700 | -1.032981688 | 8.7981E-75 | 2.5604E-73 |
| AT4G31750 | 1.647464195 | 3.8657E-52 | 7.4936E-51 |
| AT4G31790 | -1.284245979 | 3.2189E-23 | 2.7101E-22 |
| AT4G31800 | 1.157920611 | 1.1755E-14 | 6.6799E-14 |
| AT4G31805 | -1.687821981 | 6.4652E-08 | 2.3455E-07 |
| AT4G31830 | 6.297132415 | 6.5668E-08 | 2.3801E-07 |
| AT4G31850 | -1.678178763 | 4.294E-48 | 7.5132E-47 |
| AT4G31860 | 3.081223671 | 0 | 0 |
| AT4G31870 | 1.392650889 | 3.0097E-06 | 9.2709E-06 |
| AT4G31890 | -2.084665214 | 3.1857E-21 | 2.4606E-20 |
| AT4G31910 | -2.253041247 | 3.9389E-47 | 6.7167E-46 |
| AT4G31940 | -2.417781008 | 1.7082E-07 | 5.9559E-07 |
| AT4G31970 | -7.825357022 | 7.4282E-25 | 6.6438E-24 |
| AT4G32070 | 1.030028372 | 5.4824E-33 | 6.4114E-32 |
| AT4G32150 | 1.086763141 | 7.3227E-43 | 1.1327E-41 |
| AT4G32190 | 1.032340813 | 2.6491E-16 | 1.6334E-15 |
| AT4G32250 | 2.089824178 | 8.362E-106 | 3.808E-104 |
| AT4G32295 | 1.488487003 | 1.0641E-12 | 5.3881E-12 |
| AT4G32400 | -1.036675032 | 2.1115E-20 | 1.5769E-19 |
| AT4G32440 | 1.352194044 | 1.5743E-20 | 1.1796E-19 |
| AT4G32490 | 6.385725928 | 0.00074986 | 0.00172869 |
| AT4G32570 | -1.025002883 | 8.6978E-17 | 5.5018E-16 |
| AT4G32650 | -1.131059895 | 1.333E-14 | 7.5509E-14 |
| AT4G32750 | 1.242105075 | 1.737E-08 | 6.6106E-08 |
| AT4G32770 | 1.815061938 | 1.3079E-70 | 3.5526E-69 |
| AT4G32780 | -4.382432768 | 7.889E-12 | 3.7774E-11 |
| AT4G32860 | -1.326791165 | 0.00066773 | 0.00154732 |
| AT4G32870 | 1.25984615 | 1.8465E-08 | 7.0089E-08 |
| AT4G32890 | -1.559115044 | 7.5612E-07 | 2.4733E-06 |
| AT4G32920 | 1.505012293 | 4.1642E-83 | 1.375E-81 |
| AT4G32940 | 1.922585354 | 1.623E-135 | 9.983E-134 |
| AT4G32950 | -1.249098872 | 0.00026749 | 0.00065801 |
| AT4G33020 | -2.825219231 | 0.00059737 | 0.00139447 |
| AT4G33040 | 2.353938347 | 6.0988E-51 | 1.1481E-49 |
| AT4G33070 | 1.907027875 | 1.0185E-60 | 2.3285E-59 |
| AT4G33150 | 3.159452337 | 0 | 0 |
| AT4G33240 | 1.05211029 | 5.8305E-35 | 7.183E-34 |
| AT4G33440 | 1.130291957 | 1.717E-14 | 9.6774E-14 |
| AT4G33467 | 8.197944343 | 1.9197E-79 | 5.9672E-78 |
| AT4G33540 | 2.35912159 | 6.952E-220 | 8.482E-218 |
| AT4G33550 | 2.616442084 | 1.0595E-66 | 2.7285E-65 |
| AT4G33700 | 1.490871411 | 2.2722E-78 | 6.9775E-77 |
| AT4G33730 | -7.867395232 | 1.1226E-06 | 3.6061E-06 |
| AT4G08835 | 5.013264196 | 0.00434204 | 0.00885405 |
| AT4G33790 | -1.964704198 | 0.00115293 | 0.00258197 |
| AT4G33880 | -3.929359102 | 8.6542E-06 | 2.5521E-05 |
| AT4G33905 | 6.481784891 | 0 | 0 |
| AT4G33910 | 1.298035135 | 1.1193E-27 | 1.1142E-26 |
| AT4G33920 | 1.297367041 | 4.3562E-35 | 5.3987E-34 |
| AT4G33930 | 3.888938782 | 1.0839E-68 | 2.869E-67 |
| AT4G33940 | 1.766694404 | 1.4692E-47 | 2.5318E-46 |
| AT4G33950 | 2.153759572 | 1.42E-113 | 7.044E-112 |
| AT4G33980 | 1.164045138 | 9.2137E-18 | 6.1414E-17 |
| AT4G34000 | 3.196634719 | 0 | 0 |
| AT4G34020 | -1.010036641 | 2.2291E-11 | 1.0384E-10 |
| AT4G34060 | 1.198602681 | 0.00110464 | 0.00247967 |
| AT4G34160 | -1.372985429 | 1.7145E-21 | 1.3378E-20 |
| AT4G34210 | 4.722974879 | 4.6927E-15 | 2.7251E-14 |
| AT4G34220 | -1.1070562 | 2.869E-13 | 1.5003E-12 |
| AT4G34230 | 2.238077771 | 1.08E-149 | 7.776E-148 |
| AT4G34280 | 1.06995751 | 1.0411E-13 | 5.6258E-13 |
| AT4G34290 | -1.836926806 | 8.023E-74 | 2.2939E-72 |
| AT4G34370 | 1.268124976 | 1.3381E-26 | 1.2742E-25 |
| AT4G34419 | -4.747246409 | 0.00038131 | 0.00091752 |
| AT4G34480 | 1.164664602 | 9.9691E-31 | 1.0883E-29 |
| AT4G34580 | -1.353616682 | 3.6073E-05 | 9.9365E-05 |
| AT4G34600 | 1.0520922 | 3.8886E-13 | 2.0178E-12 |
| AT4G34610 | 1.008251162 | 1.861E-26 | 1.7626E-25 |
| AT4G34630 | 1.328780589 | 1.7921E-44 | 2.8816E-43 |
| AT4G34650 | 3.449119696 | 2.104E-178 | 1.951E-176 |
| AT4G34710 | 2.540993844 | 0 | 0 |
| AT4G34740 | -1.716776486 | 3.6805E-39 | 5.1423E-38 |
| AT4G34760 | -1.783299659 | 6.0004E-10 | 2.5643E-09 |
| AT4G34770 | -6.95067257 | 7.3178E-05 | 0.00019405 |
| AT4G34790 | -7.037732006 | 5.5303E-05 | 0.00014909 |
| AT4G34800 | -2.175479396 | 0.00360701 | 0.00744976 |
| AT4G34810 | -3.038804963 | 0.00079575 | 0.00182729 |
| AT4G34830 | -1.624964113 | 9.6609E-95 | 3.8875E-93 |
| AT4G34860 | 1.973416976 | 3.3793E-66 | 8.6051E-65 |
| AT4G34881 | 1.346750724 | 2.3194E-09 | 9.4649E-09 |
| AT4G34890 | 1.275547392 | 2.2939E-63 | 5.5442E-62 |
| AT4G34930 | -1.491881924 | 0.00128147 | 0.00284888 |
| AT4G34950 | -3.052726095 | 7.8189E-61 | 1.7947E-59 |
| AT4G34980 | -1.269988406 | 3.3353E-53 | 6.5545E-52 |
| AT4G35030 | -1.049451605 | 1.761E-05 | 5.0261E-05 |
| AT4G35060 | -1.323057939 | 2.3461E-09 | 9.5654E-09 |
| AT4G35090 | -1.070213212 | 1.3074E-39 | 1.847E-38 |
| AT4G35140 | 1.246106634 | 2.539E-14 | 1.4202E-13 |
| AT4G35190 | 4.464717695 | 1.1502E-09 | 4.8098E-09 |
| AT4G35300 | 1.613803173 | 6.8448E-95 | 2.7641E-93 |
| AT4G35480 | 1.315128026 | 5.9063E-09 | 2.3282E-08 |
| AT4G35500 | 1.17786533 | 2.5745E-11 | 1.1941E-10 |
| AT4G35510 | 1.361432128 | 1.2143E-22 | 9.9913E-22 |
| AT4G35560 | 2.075287662 | 1.2256E-89 | 4.6069E-88 |
| AT4G35630 | -1.295655432 | 6.3805E-41 | 9.3559E-40 |
| AT4G35690 | 4.578347998 | 7.8592E-18 | 5.2523E-17 |
| AT4G35720 | 2.041484859 | 4.258E-24 | 3.7081E-23 |
| AT4G35770 | 1.351396093 | 3.5008E-08 | 1.299E-07 |
| AT4G35783 | 2.929754454 | 4.7845E-09 | 1.9028E-08 |
| AT4G35790 | 2.416662038 | 0 | 0 |
| AT4G35810 | -1.418451394 | 0.00034874 | 0.00084388 |
| AT4G35860 | 1.963774305 | 7.177E-85 | 2.4624E-83 |
| AT4G35940 | 1.408629069 | 1.5797E-19 | 1.1471E-18 |
| AT4G36010 | 1.44942463 | 9.05E-33 | 1.0524E-31 |
| AT4G36040 | 1.910179478 | 1.1368E-98 | 4.8212E-97 |
| AT4G36120 | -1.199812963 | 7.5759E-05 | 0.00020043 |
| AT4G36160 | 1.68495075 | 2.5069E-07 | 8.6036E-07 |
| AT4G36180 | -1.25850511 | 4.8019E-33 | 5.63E-32 |
| AT4G36210 | 1.699334544 | 1.599E-107 | 7.459E-106 |
| AT4G36220 | -1.002497868 | 3.6336E-35 | 4.5204E-34 |
| AT4G36450 | 3.328524712 | 1.3749E-12 | 6.9019E-12 |
| AT4G36540 | -2.080485555 | 9.6322E-42 | 1.443E-40 |
| AT4G36570 | -7.148725557 | 5.4209E-11 | 2.4707E-10 |
| AT4G36600 | 8.726485309 | 9.1331E-08 | 3.2677E-07 |
| AT4G36610 | 1.075791125 | 1.7769E-09 | 7.3126E-09 |
| AT4G36648 | 1.514754588 | 7.746E-101 | 3.347E-99 |
| AT4G36670 | -2.114291508 | 1.452E-25 | 1.3294E-24 |
| AT4G36680 | -1.125480099 | 4.1015E-18 | 2.7802E-17 |
| AT4G36700 | 3.321968961 | 1.5983E-12 | 7.9848E-12 |
| AT4G36720 | 1.423689027 | 1.6983E-09 | 6.9992E-09 |
| AT4G36740 | 2.848905074 | 4.5085E-50 | 8.2823E-49 |
| AT4G36760 | 1.23889108 | 1.4301E-55 | 2.9717E-54 |
| AT4G36780 | 1.030122955 | 1.5002E-23 | 1.2806E-22 |
| AT4G36791 | 4.330652598 | 0.00352398 | 0.00729345 |
| AT4G36900 | 2.083230085 | 1.0871E-32 | 1.2623E-31 |
| AT4G36920 | -1.41619284 | 2.456E-27 | 2.406E-26 |
| AT4G36950 | 3.29550914 | 2.6837E-05 | 7.5112E-05 |
| AT4G36980 | 1.094182779 | 3.3995E-54 | 6.8217E-53 |
| AT4G36990 | 1.111192206 | 9.2733E-61 | 2.1222E-59 |
| AT4G37022 | 1.543551122 | 0.00648666 | 0.01280605 |
| AT4G37040 | -1.06944354 | 2.1864E-27 | 2.1456E-26 |
| AT4G37070 | -1.318134367 | 1.522E-14 | 8.5998E-14 |
| AT4G37080 | -1.232321158 | 8.1144E-23 | 6.7079E-22 |
| AT4G37140 | 1.621863691 | 0.00078054 | 0.00179434 |
| AT4G37160 | -5.572327609 | 3.5897E-28 | 3.6369E-27 |
| AT4G37180 | 1.06652406 | 3.8427E-22 | 3.0861E-21 |
| AT4G37220 | 5.792292087 | 1.0241E-26 | 9.8138E-26 |
| AT4G37240 | -1.719332715 | 2.6614E-06 | 8.2404E-06 |
| AT4G37290 | -4.364298965 | 5.2194E-14 | 2.8661E-13 |
| AT4G37340 | -3.102038141 | 3.4615E-07 | 1.172E-06 |
| AT4G37370 | 1.609199464 | 1.3404E-48 | 2.3763E-47 |
| AT4G37380 | -1.113621203 | 7.6039E-05 | 0.00020112 |
| AT4G37410 | -1.121558563 | 1.2826E-15 | 7.6585E-15 |
| AT4G37420 | 5.218802495 | 2.4932E-08 | 9.3608E-08 |
| AT4G37450 | -1.142071573 | 1.5979E-22 | 1.3082E-21 |
| AT4G37470 | 1.167240765 | 1.3416E-46 | 2.2623E-45 |
| AT4G37520 | -2.882964302 | 1.674E-151 | 1.236E-149 |
| AT4G37530 | -1.852422755 | 1.7258E-25 | 1.5769E-24 |
| AT4G37540 | -1.012051502 | 3.3302E-22 | 2.684E-21 |
| AT4G37560 | 1.364227324 | 8.2722E-06 | 2.4432E-05 |
| AT4G37650 | -1.0538375 | 2.1677E-11 | 1.0102E-10 |
| AT4G37680 | 1.19574637 | 1.8508E-30 | 1.9985E-29 |
| AT4G37690 | 2.581730863 | 1.5089E-07 | 5.2948E-07 |
| AT4G37700 | -2.220247554 | 0.0048059 | 0.00972519 |
| AT4G37740 | -1.098153805 | 9.6463E-07 | 3.1249E-06 |
| AT4G37750 | -1.441760632 | 1.3353E-30 | 1.4522E-29 |
| AT4G37760 | 1.34824194 | 7.1469E-61 | 1.6421E-59 |
| AT4G37790 | 1.87980654 | 5.8978E-88 | 2.153E-86 |
| AT4G37800 | -3.058785417 | 4.247E-206 | 4.797E-204 |
| AT4G37810 | -1.188865535 | 0.00501492 | 0.01011227 |
| AT4G37920 | -1.143618971 | 3.2535E-18 | 2.2172E-17 |
| AT4G37950 | 5.769253124 | 0.00547047 | 0.01095054 |
| AT4G37990 | 1.682763871 | 0.0082082 | 0.01589934 |
| AT4G38060 | 2.20345612 | 5.0769E-91 | 1.9338E-89 |
| AT4G38062 | -1.040022354 | 3.0974E-06 | 9.532E-06 |
| AT4G38100 | -1.286655987 | 5.9243E-29 | 6.1721E-28 |
| AT4G38150 | -1.454517555 | 1.6398E-15 | 9.7456E-15 |
| AT4G38160 | -1.799298571 | 2.1531E-46 | 3.6122E-45 |
| AT4G38330 | 1.16691789 | 7.7586E-05 | 0.00020481 |
| AT4G38360 | 1.051286481 | 4.3685E-26 | 4.0799E-25 |
| AT4G38400 | 2.154791299 | 3.2696E-36 | 4.2313E-35 |
| AT4G38420 | -2.66784466 | 2.2664E-32 | 2.605E-31 |
| AT4G38520 | -1.035050778 | 6.0578E-31 | 6.6609E-30 |
| AT4G38620 | -1.102167157 | 1.0081E-22 | 8.3096E-22 |
| AT4G38660 | -2.430878336 | 2.3606E-91 | 9.0215E-90 |
| AT4G38710 | -1.018391888 | 6.6133E-48 | 1.1483E-46 |
| AT4G38730 | 2.816980929 | 1.1359E-94 | 4.5547E-93 |
| AT4G38770 | -1.349896216 | 6.745E-122 | 3.664E-120 |
| AT4G38830 | -3.171169183 | 0.00258005 | 0.00547035 |
| AT4G38840 | -2.779008759 | 1.9712E-39 | 2.7745E-38 |
| AT4G38860 | -5.809884324 | 0.00215962 | 0.00464498 |
| AT4G38905 | 6.331349897 | 0.00082625 | 0.00189274 |
| AT4G38932 | 1.305019278 | 7.4105E-21 | 5.6415E-20 |
| AT4G38940 | 1.084344141 | 3.6137E-07 | 1.2211E-06 |
| AT4G38950 | -1.063878588 | 1.3909E-21 | 1.0906E-20 |
| AT4G39010 | -1.56518061 | 0.00010003 | 0.00026103 |
| AT4G39070 | -2.745382615 | 2.2176E-23 | 1.883E-22 |
| AT4G39090 | 1.819964008 | 1.109E-240 | 1.542E-238 |
| AT4G39120 | -1.503642872 | 1.4011E-16 | 8.7581E-16 |
| AT4G39140 | 1.568337799 | 1.9411E-44 | 3.1167E-43 |
| AT4G39180 | 1.060015984 | 2.6386E-07 | 9.0338E-07 |
| AT4G39210 | 2.261272697 | 1.017E-179 | 9.511E-178 |
| AT4G39250 | -7.195441656 | 2.5266E-05 | 7.0907E-05 |
| AT4G39260 | -1.293967763 | 3.2181E-82 | 1.0504E-80 |
| AT4G39330 | 1.554594289 | 2.109E-114 | 1.062E-112 |
| AT4G39340 | 3.114781519 | 1.6912E-17 | 1.1114E-16 |
| AT4G39360 | 4.001805886 | 0.00025577 | 0.00063094 |
| AT4G39670 | 2.621441782 | 3.7772E-67 | 9.771E-66 |
| AT4G39675 | 2.339911056 | 0.00325984 | 0.00678866 |
| AT4G39700 | 2.297054543 | 3.7676E-08 | 1.3925E-07 |
| AT4G39720 | -1.381455776 | 0.00029863 | 0.00072935 |
| AT4G39730 | 1.430181201 | 2.911E-114 | 1.463E-112 |
| AT4G39770 | -1.704735688 | 1.0999E-05 | 3.2094E-05 |
| AT4G39790 | -1.184866519 | 0.00626111 | 0.01239833 |
| AT4G39890 | 1.078393917 | 0.00034231 | 0.00082919 |
| AT4G39925 | 1.638199926 | 0.00049262 | 0.00116484 |
| AT4G39940 | -2.598385836 | 1.839E-177 | 1.699E-175 |
| AT4G39950 | -2.535568632 | 6.951E-212 | 8.092E-210 |
| AT4G39960 | -1.412026084 | 1.7258E-61 | 4.0344E-60 |
| AT4G40010 | 5.42645487 | 6.75E-304 | 1.439E-301 |
| AT4G40070 | 1.738491946 | 8.6267E-09 | 3.36E-08 |
| AT4G40090 | -3.314043945 | 8.8492E-24 | 7.6247E-23 |
| AT5G01050 | -1.652928507 | 0.00153275 | 0.00337137 |
| AT5G01100 | 2.370659405 | 1.1493E-37 | 1.5535E-36 |
| AT5G01190 | -1.538295269 | 0.00030241 | 0.00073794 |
| AT5G01200 | 2.571524278 | 1.2726E-36 | 1.6601E-35 |
| AT5G01240 | -1.572663383 | 1.7273E-38 | 2.3783E-37 |
| AT5G01260 | 1.568842565 | 3.0784E-44 | 4.9082E-43 |
| AT5G01270 | 1.128635882 | 9.2256E-35 | 1.1274E-33 |
| AT5G01280 | -2.193524657 | 0.00432426 | 0.00882015 |
| AT5G01300 | 7.286542849 | 2.1638E-88 | 7.9245E-87 |
| AT5G01330 | -1.776987118 | 0.00265623 | 0.00561882 |
| AT5G01360 | -1.193487876 | 0.00253643 | 0.00538338 |
| AT5G01380 | 2.095024089 | 3.1355E-06 | 9.6466E-06 |
| AT5G01520 | 5.770819183 | 0 | 0 |
| AT5G01590 | -1.030944727 | 3.9742E-47 | 6.7719E-46 |
| AT5G01620 | -1.160487758 | 6.4215E-08 | 2.3304E-07 |
| AT5G01670 | 1.879512723 | 6.5501E-35 | 8.0564E-34 |
| AT5G01740 | -3.043119119 | 3.5073E-08 | 1.3012E-07 |
| AT5G01790 | -2.912698448 | 3.6816E-44 | 5.8618E-43 |
| AT5G01800 | 1.174951534 | 1.2546E-41 | 1.8734E-40 |
| AT5G01820 | 1.677036555 | 4.8604E-68 | 1.2747E-66 |
| AT5G01850 | 1.104886095 | 1.2611E-10 | 5.6132E-10 |
| AT5G01880 | 2.269574934 | 1.0927E-25 | 1.0057E-24 |
| AT5G01890 | -1.011632397 | 2.1161E-19 | 1.5245E-18 |
| AT5G01910 | -1.155368299 | 0.00027928 | 0.00068457 |
| AT5G01990 | 2.053206997 | 1.7832E-48 | 3.1515E-47 |
| AT5G02020 | 6.390227154 | 8.718E-267 | 1.463E-264 |
| AT5G02040 | 1.181396665 | 2.7614E-19 | 1.9782E-18 |
| AT5G02090 | -1.133848917 | 0.00030918 | 0.00075333 |
| AT5G02170 | 1.673075945 | 4.2865E-08 | 1.5756E-07 |
| AT5G02230 | 2.337241772 | 1.8286E-60 | 4.1554E-59 |
| AT5G02260 | -1.214000235 | 3.4725E-18 | 2.3622E-17 |
| AT5G02270 | -1.053738828 | 9.7481E-48 | 1.6875E-46 |
| AT5G02320 | 1.528779981 | 4.3315E-11 | 1.9845E-10 |
| AT5G02350 | -2.029755923 | 6.8486E-05 | 0.00018225 |
| AT5G02360 | -4.049043666 | 9.0758E-07 | 2.9481E-06 |
| AT5G02400 | -1.819200741 | 1.3976E-06 | 4.451E-06 |
| AT5G02430 | 1.2447661 | 1.8378E-08 | 6.977E-08 |
| AT5G02540 | -1.965809816 | 2.2439E-07 | 7.7431E-07 |
| AT5G02640 | 1.833136602 | 1.3738E-08 | 5.2591E-08 |
| AT5G02760 | -4.224832896 | 1.4674E-27 | 1.4519E-26 |
| AT5G02810 | 1.02073095 | 9.4992E-20 | 6.9311E-19 |
| AT5G02830 | -1.146955667 | 3.0191E-24 | 2.6484E-23 |
| AT5G02840 | 1.235254684 | 1.7442E-22 | 1.4254E-21 |
| AT5G02870 | -1.044412138 | 9.2048E-80 | 2.8888E-78 |
| AT5G02880 | 1.369978782 | 1.185E-57 | 2.5824E-56 |
| AT5G02890 | -1.979486905 | 1.1743E-43 | 1.8465E-42 |
| AT5G02970 | 1.207123971 | 1.162E-31 | 1.3086E-30 |
| AT5G03030 | 1.319757796 | 3.091E-33 | 3.639E-32 |
| AT5G03080 | 1.436026419 | 3.0695E-42 | 4.6689E-41 |
| AT5G03090 | -2.285415558 | 0.00971626 | 0.01854959 |
| AT5G03120 | -3.586683549 | 1.3633E-14 | 7.7185E-14 |
| AT5G03150 | -1.409287355 | 2.7833E-09 | 1.1264E-08 |
| AT5G03190 | 1.413574272 | 1.2313E-39 | 1.7439E-38 |
| AT5G03204 | 7.953160886 | 7.3914E-07 | 2.4206E-06 |
| AT5G03210 | 6.626531267 | 0 | 0 |
| AT5G03230 | 1.446744671 | 2.4382E-16 | 1.5076E-15 |
| AT5G03240 | 1.168695909 | 1.238E-116 | 6.405E-115 |
| AT5G03285 | 1.168407556 | 7.3879E-06 | 2.1905E-05 |
| AT5G03300 | -1.133562854 | 2.7115E-56 | 5.7124E-55 |
| AT5G03355 | -3.230212639 | 2.3793E-06 | 7.4039E-06 |
| AT5G03380 | -1.257206278 | 2.8847E-45 | 4.7012E-44 |
| AT5G03390 | -2.660271981 | 2.0864E-16 | 1.295E-15 |
| AT5G03495 | 2.386915943 | 4.3183E-11 | 1.9792E-10 |
| AT5G03640 | -1.690778505 | 0.00550987 | 0.01101781 |
| AT5G03670 | -1.849508164 | 8.9412E-06 | 2.6309E-05 |
| AT5G03700 | -1.005455734 | 9.352E-08 | 3.3413E-07 |
| AT5G03800 | -1.096563939 | 1.3223E-09 | 5.5034E-09 |
| AT5G03870 | -1.112896598 | 3.4371E-06 | 1.0528E-05 |
| AT5G03890 | 1.291259322 | 0.00052219 | 0.00122979 |
| AT5G03995 | -8.455443849 | 5.6858E-08 | 2.0707E-07 |
| AT5G04010 | 6.63251427 | 2.831E-09 | 1.1444E-08 |
| AT5G04080 | 2.263060664 | 6.8302E-64 | 1.6739E-62 |
| AT5G04120 | 2.513366595 | 0.00484284 | 0.00979386 |
| AT5G04150 | 3.149973381 | 0.00392734 | 0.00806682 |
| AT5G04230 | -2.777470834 | 5.9665E-32 | 6.7559E-31 |
| AT5G04250 | 3.87457534 | 1.251E-226 | 1.595E-224 |
| AT5G04310 | -1.764603105 | 1.0122E-06 | 3.2726E-06 |
| AT5G04340 | 3.911250887 | 0 | 0 |
| AT5G04370 | 2.85323837 | 2.6792E-39 | 3.7571E-38 |
| AT5G04380 | 12.00764899 | 1.4425E-16 | 9.0072E-16 |
| AT5G04410 | 1.152811414 | 2.0979E-54 | 4.2322E-53 |
| AT5G04470 | -1.330263107 | 0.00076531 | 0.00176181 |
| AT5G04490 | 1.205393458 | 6.2845E-16 | 3.8095E-15 |
| AT5G04530 | 1.3067136 | 7.8238E-38 | 1.0632E-36 |
| AT5G00980 | 1.035729791 | 0.0048543 | 0.00981528 |
| AT5G04760 | 2.607678566 | 9.5542E-50 | 1.7411E-48 |
| AT5G04800 | -1.086489121 | 1.1351E-51 | 2.1764E-50 |
| AT5G04850 | 1.314685301 | 6.3152E-28 | 6.3391E-27 |
| AT5G04895 | -1.535774944 | 4.6828E-19 | 3.3201E-18 |
| AT5G04950 | -3.427519123 | 1.091E-147 | 7.708E-146 |
| AT5G04960 | -7.69170194 | 2.3381E-23 | 1.9824E-22 |
| AT5G04970 | -2.955469143 | 0.00051429 | 0.00121295 |
| AT5G05100 | 1.472434964 | 3.2689E-51 | 6.2138E-50 |
| AT5G05110 | 1.418312188 | 2.5973E-24 | 2.2837E-23 |
| AT5G05140 | 1.22128719 | 1.7875E-29 | 1.8881E-28 |
| AT5G05180 | -1.076704384 | 7.4682E-07 | 2.4443E-06 |
| AT5G05220 | 9.768447216 | 5.786E-154 | 4.4E-152 |
| AT5G05250 | 1.848360584 | 0.00583229 | 0.01161267 |
| AT5G05290 | 6.218746406 | 0.00129874 | 0.00288473 |
| AT5G05320 | -2.421633012 | 0.00022499 | 0.00055882 |
| AT5G05340 | 2.551336885 | 1.2211E-13 | 6.5464E-13 |
| AT5G05390 | 1.426597506 | 1.6775E-05 | 4.8006E-05 |
| AT5G05400 | -2.237185456 | 6.1208E-11 | 2.778E-10 |
| AT5G05410 | 3.449384138 | 0 | 0 |
| AT5G05470 | -1.185904444 | 1.3944E-16 | 8.7238E-16 |
| AT5G05500 | -6.534123852 | 2.5291E-23 | 2.1411E-22 |
| AT5G05580 | -1.505834188 | 3.1226E-57 | 6.7467E-56 |
| AT5G05600 | 1.123407928 | 4.6897E-05 | 0.00012764 |
| AT5G05640 | 1.121876663 | 0.00064019 | 0.00148758 |
| AT5G05750 | 1.298671014 | 9.6966E-16 | 5.8175E-15 |
| AT5G05840 | 1.550293804 | 1.9536E-07 | 6.7742E-07 |
| AT5G05850 | 1.053312903 | 1.7796E-05 | 5.0778E-05 |
| AT5G05870 | 1.472182536 | 2.3162E-23 | 1.966E-22 |
| AT5G05890 | -2.284166263 | 2.7647E-19 | 1.9799E-18 |
| AT5G05930 | 1.445351268 | 1.4828E-28 | 1.5233E-27 |
| AT5G05960 | -1.300093599 | 1.824E-20 | 1.3645E-19 |
| AT5G05965 | 4.030988637 | 3.666E-06 | 1.1206E-05 |
| AT5G05987 | 1.156541821 | 8.7898E-12 | 4.1955E-11 |
| AT5G05990 | -1.470033645 | 1.6377E-16 | 1.0204E-15 |
| AT5G06080 | 2.542087177 | 0.00077401 | 0.00178059 |
| AT5G06090 | 2.947193158 | 1.8161E-46 | 3.0535E-45 |
| AT5G06190 | 1.437630385 | 0.00014264 | 0.00036514 |
| AT5G06200 | -4.581529211 | 1.127E-12 | 5.6954E-12 |
| AT5G06250 | -2.359252805 | 0.00012417 | 0.00032053 |
| AT5G06270 | -1.260713937 | 2.7035E-10 | 1.1821E-09 |
| AT5G06320 | 1.212088797 | 7.3718E-22 | 5.8503E-21 |
| AT5G06370 | 1.743806604 | 1.5529E-75 | 4.5719E-74 |
| AT5G06400 | -1.293506787 | 0.0028066 | 0.00590677 |
| AT5G06440 | 1.252295826 | 2.273E-36 | 2.95E-35 |
| AT5G06530 | 3.229847008 | 7.776E-163 | 6.359E-161 |
| AT5G06550 | -1.582650038 | 2.0974E-28 | 2.1402E-27 |
| AT5G06570 | -1.614356565 | 2.1606E-08 | 8.167E-08 |
| AT5G06630 | -6.046095181 | 3.2413E-27 | 3.1618E-26 |
| AT5G06640 | -6.879135328 | 6.2744E-27 | 6.0454E-26 |
| AT5G06720 | -2.740393946 | 8.8886E-05 | 0.00023294 |
| AT5G06740 | -2.942639657 | 6.1118E-14 | 3.3489E-13 |
| AT5G06760 | 8.072547046 | 0 | 0 |
| AT5G06790 | -1.560302735 | 8.6012E-06 | 2.5374E-05 |
| AT5G06811 | -1.254050953 | 2.279E-06 | 7.1056E-06 |
| AT5G06839 | 1.718677068 | 6.7297E-23 | 5.5814E-22 |
| AT5G06930 | -1.371165212 | 2.4131E-06 | 7.5019E-06 |
| AT5G06940 | -1.959197915 | 4.9078E-09 | 1.9488E-08 |
| AT5G06980 | 2.014503136 | 8.2867E-08 | 2.9756E-07 |
| AT5G07010 | 1.064351695 | 0.00177124 | 0.00385506 |
| AT5G07070 | 1.322910763 | 5.1929E-07 | 1.7274E-06 |
| AT5G07110 | -1.904416454 | 8.0699E-07 | 2.6326E-06 |
| AT5G07130 | -1.01173686 | 5.7133E-05 | 0.00015368 |
| AT5G07190 | -1.812454328 | 5.5701E-05 | 0.00015008 |
| AT5G07240 | -1.34314974 | 5.7433E-22 | 4.5898E-21 |
| AT5G07322 | -1.257990164 | 2.822E-06 | 8.7221E-06 |
| AT5G07330 | 7.033721495 | 7.7974E-81 | 2.4744E-79 |
| AT5G07340 | -1.129924146 | 5.6507E-33 | 6.6049E-32 |
| AT5G07460 | -1.915273898 | 2.7569E-55 | 5.6565E-54 |
| AT5G07570 | -4.409617364 | 0.00289565 | 0.0060785 |
| AT5G07580 | -2.290612819 | 3.7022E-43 | 5.7501E-42 |
| AT5G07650 | -2.223734296 | 8.6465E-05 | 0.00022688 |
| AT5G07690 | -3.262575409 | 5.8593E-68 | 1.5331E-66 |
| AT5G07700 | -1.417257287 | 0.00674214 | 0.01327482 |
| AT5G07740 | 1.171403521 | 1.7167E-50 | 3.1871E-49 |
| AT5G07780 | -3.312741732 | 1.9623E-05 | 5.5777E-05 |
| AT5G07900 | -2.210532129 | 8.2767E-17 | 5.2398E-16 |
| AT5G07920 | 2.111257983 | 8.8E-107 | 4.056E-105 |
| AT5G08020 | -1.800000827 | 1.6278E-19 | 1.1802E-18 |
| AT5G08030 | 7.902854196 | 1.0181E-06 | 3.2902E-06 |
| AT5G08150 | -3.661956016 | 0.00160551 | 0.00352089 |
| AT5G08180 | -1.002074119 | 6.6289E-37 | 8.7123E-36 |
| AT5G08280 | -1.274033404 | 6.1617E-82 | 1.9998E-80 |
| AT5G08330 | -2.560116004 | 3.1317E-50 | 5.7856E-49 |
| AT5G08600 | -1.258853079 | 1.3895E-07 | 4.8931E-07 |
| AT5G08610 | -1.843862497 | 4.855E-155 | 3.742E-153 |
| AT5G08640 | -1.914725347 | 1.684E-241 | 2.372E-239 |
| AT5G09225 | 1.14509391 | 3.0465E-14 | 1.6945E-13 |
| AT5G09240 | -1.26229008 | 3.0794E-05 | 8.5515E-05 |
| AT5G09430 | 1.784245708 | 0.00813789 | 0.0157752 |
| AT5G09440 | 1.153906931 | 2.8269E-27 | 2.7587E-26 |
| AT5G09460 | -1.345513204 | 1.1495E-39 | 1.629E-38 |
| AT5G09470 | 2.308853559 | 1.7331E-06 | 5.4633E-06 |
| AT5G09480 | -1.00345026 | 1.1811E-07 | 4.178E-07 |
| AT5G09510 | -1.015169748 | 5.6155E-44 | 8.9036E-43 |
| AT5G09520 | 1.233356683 | 1.1881E-11 | 5.6333E-11 |
| AT5G09570 | 1.035874333 | 0.00012879 | 0.00033202 |
| AT5G09610 | 5.704128121 | 2.3159E-11 | 1.0773E-10 |
| AT5G09620 | 1.814004733 | 2.528E-170 | 2.227E-168 |
| AT5G09820 | -1.22632427 | 4.8689E-14 | 2.6781E-13 |
| AT5G09840 | -1.290792329 | 4.1149E-13 | 2.1333E-12 |
| AT5G09870 | -1.264801424 | 3.1174E-43 | 4.8517E-42 |
| AT5G09930 | 6.921825241 | 8.0155E-05 | 0.00021111 |
| AT5G09978 | -2.467648825 | 0.00223826 | 0.00480054 |
| AT5G09990 | 2.063566024 | 2.038E-05 | 5.7833E-05 |
| AT5G10020 | -1.322139941 | 5.0983E-21 | 3.9035E-20 |
| AT5G10100 | 1.007157361 | 1.0807E-13 | 5.8248E-13 |
| AT5G10130 | -1.945183273 | 7.3599E-27 | 7.0704E-26 |
| AT5G10210 | -3.30569112 | 1.361E-06 | 4.3431E-06 |
| AT5G10250 | -2.769647042 | 0.00049533 | 0.00117077 |
| AT5G10278 | -2.702316681 | 0.00051133 | 0.00120647 |
| AT5G10280 | -1.332347835 | 0.00018145 | 0.00045654 |
| AT5G10300 | 2.628394115 | 1.1219E-62 | 2.6803E-61 |
| AT5G10390 | -1.639747463 | 6.224E-24 | 5.3933E-23 |
| AT5G10410 | 1.692508335 | 2.0356E-19 | 1.4693E-18 |
| AT5G10470 | -1.010432136 | 3.1018E-37 | 4.1386E-36 |
| AT5G10480 | 1.34419449 | 4.1181E-72 | 1.1444E-70 |
| AT5G10625 | 1.309568657 | 1.1762E-13 | 6.3157E-13 |
| AT5G10650 | 1.226124914 | 3.5341E-19 | 2.5214E-18 |
| AT5G10730 | 1.792143096 | 7.1735E-94 | 2.8415E-92 |
| AT5G10740 | 1.278463421 | 1.6908E-12 | 8.4287E-12 |
| AT5G10770 | -2.352513414 | 1.8356E-54 | 3.7096E-53 |
| AT5G10850 | -1.002515547 | 0.00733799 | 0.01433912 |
| AT5G10860 | 1.363648172 | 6.966E-103 | 3.08E-101 |
| AT5G10920 | -1.192578694 | 6.0374E-36 | 7.7431E-35 |
| AT5G10930 | 3.341456975 | 6.476E-194 | 6.809E-192 |
| AT5G10946 | 2.334876085 | 4.534E-21 | 3.4795E-20 |
| AT5G10980 | 1.012519414 | 7.9544E-59 | 1.7654E-57 |
| AT5G11060 | 1.407770718 | 1.0811E-74 | 3.1383E-73 |
| AT5G00365 | 3.173191304 | 3.0046E-10 | 1.3087E-09 |
| AT5G11110 | 3.467748267 | 0 | 0 |
| AT5G11160 | -2.274631485 | 9.1902E-12 | 4.3821E-11 |
| AT5G11210 | -1.83038737 | 0.00210343 | 0.0045301 |
| AT5G11240 | -1.255620942 | 1.2568E-29 | 1.3331E-28 |
| AT5G11270 | -1.33017363 | 7.4016E-25 | 6.6225E-24 |
| AT5G11420 | -2.209578419 | 5.876E-101 | 2.544E-99 |
| AT5G11440 | -4.340745374 | 0.00264248 | 0.00559129 |
| AT5G11450 | -1.354746219 | 1.6444E-57 | 3.5731E-56 |
| AT5G11520 | 1.098588099 | 3.2887E-44 | 5.2398E-43 |
| AT5G11540 | -1.028926302 | 0.00964892 | 0.01843184 |
| AT5G11590 | -1.941205523 | 1.2636E-06 | 4.0423E-06 |
| AT5G11600 | 1.19487527 | 5.7977E-12 | 2.7966E-11 |
| AT5G11610 | -1.622295414 | 2.9507E-07 | 1.0056E-06 |
| AT5G11630 | -1.045733561 | 4.559E-07 | 1.5252E-06 |
| AT5G11650 | 1.210234093 | 6.9567E-21 | 5.3014E-20 |
| AT5G11750 | -1.300831196 | 1.5035E-11 | 7.0831E-11 |
| AT5G11920 | -2.09810519 | 3.0928E-05 | 8.5847E-05 |
| AT5G11930 | -3.643758818 | 5.1098E-05 | 0.00013818 |
| AT5G11970 | 1.469179987 | 0.00026679 | 0.00065643 |
| AT5G12020 | 2.097978789 | 4.967E-18 | 3.3589E-17 |
| AT5G12030 | 2.752387071 | 5.6374E-42 | 8.5012E-41 |
| AT5G12110 | -1.555728535 | 1.2009E-13 | 6.4411E-13 |
| AT5G12140 | 1.317876623 | 5.9433E-85 | 2.0422E-83 |
| AT5G12235 | -6.489529562 | 0.00050199 | 0.00118554 |
| AT5G12270 | -2.909399878 | 9.2371E-11 | 4.1479E-10 |
| AT5G12340 | -2.061528099 | 9.9688E-08 | 3.5544E-07 |
| AT5G12840 | 2.217731813 | 2.2489E-85 | 7.7978E-84 |
| AT5G12860 | -1.098378673 | 1.6335E-81 | 5.2791E-80 |
| AT5G12900 | -1.029231126 | 1.82E-10 | 8.0196E-10 |
| AT5G12970 | -1.235958513 | 1.6411E-05 | 4.7028E-05 |
| AT5G13060 | -1.064300872 | 7.7078E-07 | 2.5191E-06 |
| AT5G13080 | 2.998733531 | 1.7259E-07 | 6.0136E-07 |
| AT5G13150 | -1.265760576 | 0.00165631 | 0.00362254 |
| AT5G13170 | 6.749074706 | 1.7221E-62 | 4.1058E-61 |
| AT5G13200 | 2.936642131 | 1.486E-154 | 1.134E-152 |
| AT5G13205 | 3.490125872 | 5.4876E-23 | 4.5712E-22 |
| AT5G13210 | 2.046645071 | 1.7755E-19 | 1.2848E-18 |
| AT5G13310 | 1.677528856 | 3.6836E-28 | 3.7287E-27 |
| AT5G13330 | 2.034328556 | 3.0037E-40 | 4.3376E-39 |
| AT5G13370 | 1.468458826 | 1.266E-74 | 3.6656E-73 |
| AT5G13550 | 1.23237828 | 3.6149E-31 | 3.9979E-30 |
| AT5G13570 | 1.113090405 | 1.8221E-18 | 1.2617E-17 |
| AT5G13580 | 1.157383402 | 1.0662E-13 | 5.7524E-13 |
| AT5G13700 | 2.494977704 | 2.7874E-22 | 2.2528E-21 |
| AT5G13740 | 1.873020659 | 5.5959E-54 | 1.12E-52 |
| AT5G13750 | 1.990033099 | 1.585E-130 | 9.49E-129 |
| AT5G13760 | 1.296174216 | 5.5672E-22 | 4.4538E-21 |
| AT5G13800 | 1.989637193 | 1.212E-139 | 7.832E-138 |
| AT5G13820 | 2.203172831 | 1.2715E-41 | 1.8961E-40 |
| AT5G13830 | -1.561349486 | 0.00043667 | 0.00104151 |
| AT5G13880 | 2.787444618 | 1.1576E-36 | 1.5136E-35 |
| AT5G13900 | 1.810554205 | 1.0787E-14 | 6.1393E-14 |
| AT5G13930 | -1.158223215 | 2.0509E-69 | 5.473E-68 |
| AT5G13970 | 1.012959562 | 6.1449E-14 | 3.3654E-13 |
| AT5G13990 | -1.91391419 | 3.4062E-06 | 1.0439E-05 |
| AT5G14050 | -1.213403993 | 2.0435E-42 | 3.125E-41 |
| AT5G14150 | -2.162978874 | 7.3187E-07 | 2.3975E-06 |
| AT5G14200 | -2.639761609 | 1.037E-279 | 1.923E-277 |
| AT5G14230 | -1.117623347 | 0.00025925 | 0.00063906 |
| AT5G14330 | -1.844264387 | 3.2232E-17 | 2.0875E-16 |
| AT5G14340 | -4.099576755 | 0.0002244 | 0.00055766 |
| AT5G14390 | 1.293638842 | 1.8046E-26 | 1.7106E-25 |
| AT5G14500 | 1.36502956 | 2.4938E-24 | 2.196E-23 |
| AT5G14580 | -1.627415879 | 7.7577E-37 | 1.0184E-35 |
| AT5G14650 | -2.532849588 | 3.191E-13 | 1.6637E-12 |
| AT5G14730 | -1.28660099 | 2.4285E-08 | 9.1343E-08 |
| AT5G14740 | -1.207719356 | 2.1E-104 | 9.378E-103 |
| AT5G14750 | -2.31403844 | 2.8308E-07 | 9.6644E-07 |
| AT5G14780 | 1.58114166 | 1.114E-138 | 7.102E-137 |
| AT5G14920 | -2.01710947 | 1.197E-118 | 6.324E-117 |
| AT5G15025 | 1.018955749 | 0.00061121 | 0.00142504 |
| AT5G15120 | 1.199986335 | 0.00683658 | 0.01344569 |
| AT5G15130 | -3.296093453 | 3.2395E-18 | 2.209E-17 |
| AT5G15150 | -1.443956515 | 0.00020447 | 0.00051098 |
| AT5G15160 | 1.468314624 | 3.1144E-08 | 1.1605E-07 |
| AT5G15190 | 3.476830224 | 6.3344E-62 | 1.4946E-60 |
| AT5G15210 | -2.086368385 | 1.0216E-23 | 8.7663E-23 |
| AT5G15240 | 2.039394742 | 1.3767E-29 | 1.4569E-28 |
| AT5G15250 | 6.436594251 | 2.2332E-08 | 8.4289E-08 |
| AT5G15260 | 1.948064215 | 1.9075E-37 | 2.5616E-36 |
| AT5G15350 | -1.60302007 | 7.1876E-63 | 1.7226E-61 |
| AT5G15430 | 2.457325766 | 0.00190885 | 0.00413288 |
| AT5G15450 | 1.091516269 | 1.2447E-45 | 2.0549E-44 |
| AT5G15500 | 5.805439383 | 0 | 0 |
| AT5G15580 | -1.250106857 | 2.2494E-15 | 1.3282E-14 |
| AT5G15660 | 4.460619119 | 2.924E-08 | 1.0922E-07 |
| AT5G15725 | -3.532991953 | 0.00097589 | 0.00220782 |
| AT5G15820 | 1.353963369 | 2.872E-08 | 1.0733E-07 |
| AT5G15830 | -1.466991642 | 0.00915462 | 0.01755668 |
| AT5G15860 | 1.881355323 | 6.382E-52 | 1.234E-50 |
| AT5G15870 | 1.117740416 | 1.1882E-18 | 8.275E-18 |
| AT5G15890 | -3.615136722 | 5.6384E-09 | 2.2261E-08 |
| AT5G15950 | 1.111822188 | 5.801E-19 | 4.1002E-18 |
| AT5G15960 | 4.17679115 | 0 | 0 |
| AT5G15970 | 1.661781759 | 6.84E-180 | 6.422E-178 |
| AT5G15980 | -1.614327015 | 3.6551E-28 | 3.7015E-27 |
| AT5G16000 | -1.293610538 | 4.4547E-19 | 3.1624E-18 |
| AT5G02515 | -2.591996612 | 0.00017545 | 0.00044282 |
| AT5G16110 | 1.534821025 | 8.6979E-71 | 2.3681E-69 |
| AT5G16120 | 1.229287423 | 1.3428E-19 | 9.7697E-19 |
| AT5G16130 | -1.134520786 | 4.5524E-73 | 1.2823E-71 |
| AT5G16170 | -2.222117002 | 0.00010569 | 0.00027521 |
| AT5G16190 | -1.308246228 | 5.4004E-05 | 0.00014576 |
| AT5G16200 | 1.442764514 | 5.91E-06 | 1.771E-05 |
| AT5G16360 | 1.71126142 | 1.6043E-16 | 1.0001E-15 |
| AT5G16380 | 1.312166088 | 3.4042E-15 | 1.9895E-14 |
| AT5G16450 | 1.71301992 | 2.56E-35 | 3.204E-34 |
| AT5G02535 | 5.277877032 | 0.00209656 | 0.00451572 |
| AT5G16530 | -1.784403999 | 2.6717E-08 | 1.0006E-07 |
| AT5G16550 | 1.098840027 | 4.5447E-21 | 3.4854E-20 |
| AT5G16590 | -1.661332137 | 2.1951E-34 | 2.6598E-33 |
| AT5G16600 | 2.761373889 | 3.085E-52 | 5.9854E-51 |
| AT5G16650 | 1.156091102 | 6.6468E-17 | 4.235E-16 |
| AT5G16720 | -1.170430378 | 1.2148E-09 | 5.067E-09 |
| AT5G16830 | 1.65864273 | 1.5848E-44 | 2.5571E-43 |
| AT5G02605 | 2.403067358 | 0.00388386 | 0.00798255 |
| AT5G16880 | 1.082472798 | 1.7632E-38 | 2.4264E-37 |
| AT5G16960 | 2.053220267 | 1.7481E-15 | 1.0381E-14 |
| AT5G17040 | -2.433501148 | 1.5387E-23 | 1.3127E-22 |
| AT5G17160 | -1.230016372 | 1.2441E-18 | 8.657E-18 |
| AT5G17165 | 1.659801838 | 0.0025438 | 0.00539851 |
| AT5G17170 | -1.712193806 | 0.00010246 | 0.00026706 |
| AT5G17190 | 1.027457202 | 1.1898E-26 | 1.1363E-25 |
| AT5G17210 | 1.101756099 | 1.7275E-09 | 7.1145E-09 |
| AT5G17220 | 1.155640723 | 7.0213E-27 | 6.7537E-26 |
| AT5G17290 | 1.28158846 | 8.6425E-15 | 4.9507E-14 |
| AT5G17300 | 2.565561718 | 1.7556E-21 | 1.3685E-20 |
| AT5G17450 | 1.603611912 | 1.3403E-17 | 8.8641E-17 |
| AT5G17460 | 4.947774988 | 0 | 0 |
| AT5G17490 | 2.285518493 | 7.518E-34 | 8.976E-33 |
| AT5G17520 | 1.152803945 | 2.5817E-25 | 2.3403E-24 |
| AT5G17630 | -1.312472574 | 2.7886E-25 | 2.5238E-24 |
| AT5G17650 | 1.060555374 | 3.8566E-08 | 1.4243E-07 |
| AT5G17700 | -1.592235026 | 8.0126E-40 | 1.1397E-38 |
| AT5G17760 | 2.675188802 | 2.302E-158 | 1.817E-156 |
| AT5G17820 | -2.041308557 | 9.5916E-52 | 1.8468E-50 |
| AT5G17850 | 2.519137906 | 9.4657E-48 | 1.6399E-46 |
| AT5G17860 | 2.546402627 | 9.479E-110 | 4.505E-108 |
| AT5G17920 | -1.191229795 | 3.02E-127 | 1.74E-125 |
| AT5G18020 | -3.833489353 | 0.00112372 | 0.00251904 |
| AT5G18030 | -3.27521414 | 0.00016584 | 0.00042038 |
| AT5G18130 | 3.23983832 | 0 | 0 |
| AT5G18270 | 1.043052855 | 1.0505E-07 | 3.7381E-07 |
| AT5G18300 | -2.058980989 | 0.00197968 | 0.00427407 |
| AT5G18430 | -1.458726853 | 5.6625E-06 | 1.7011E-05 |
| AT5G18440 | -1.015606228 | 4.1596E-09 | 1.6647E-08 |
| AT5G18570 | -1.552386834 | 9.0863E-65 | 2.2594E-63 |
| AT5G18630 | 1.633388959 | 2.6429E-36 | 3.4242E-35 |
| AT5G18633 | 2.303814116 | 0.00569528 | 0.01135968 |
| AT5G18690 | -2.249118592 | 7.0392E-16 | 4.2556E-15 |
| AT5G18780 | 1.147727232 | 1.1052E-05 | 3.2237E-05 |
| AT5G18840 | -1.20180149 | 1.6568E-06 | 5.2327E-06 |
| AT5G19040 | -2.070258234 | 4.2013E-07 | 1.4107E-06 |
| AT5G19090 | -1.348607301 | 1.8067E-44 | 2.9029E-43 |
| AT5G19120 | -1.298986109 | 7.8501E-19 | 5.5161E-18 |
| AT5G19160 | -2.367287961 | 6.3014E-08 | 2.2887E-07 |
| AT5G19190 | -2.245407493 | 1.2721E-12 | 6.3971E-12 |
| AT5G19230 | -2.794468623 | 6.9235E-36 | 8.8546E-35 |
| AT5G19240 | -2.79497721 | 6.912E-51 | 1.2969E-49 |
| AT5G19260 | -2.249852303 | 1.6838E-09 | 6.941E-09 |
| AT5G19300 | -1.691627986 | 1.4568E-31 | 1.6309E-30 |
| AT5G19430 | 1.144209221 | 1.9119E-14 | 1.0752E-13 |
| AT5G19440 | 1.022505223 | 7.9376E-40 | 1.1298E-38 |
| AT5G19470 | 4.003534393 | 3.7843E-74 | 1.0861E-72 |
| AT5G19560 | -3.03887654 | 0.00095002 | 0.00215357 |
| AT5G19600 | -3.278751743 | 5.0703E-51 | 9.5686E-50 |
| AT5G19700 | -6.895179635 | 9.371E-05 | 0.00024502 |
| AT5G19750 | -1.465667327 | 8.7513E-29 | 9.08E-28 |
| AT5G19770 | -1.296205723 | 1.4517E-86 | 5.143E-85 |
| AT5G19780 | -1.189499864 | 6.6296E-78 | 2.0168E-76 |
| AT5G19790 | -4.104607075 | 4.3211E-08 | 1.5879E-07 |
| AT5G19800 | -6.625388684 | 0.00027859 | 0.00068303 |
| AT5G19855 | 1.440103813 | 3.9184E-63 | 9.4406E-62 |
| AT5G19875 | 2.375445443 | 5.8472E-46 | 9.7166E-45 |
| AT5G19880 | -6.58732841 | 0.00032714 | 0.00079404 |
| AT5G19980 | -1.110807093 | 1.3644E-11 | 6.4476E-11 |
| AT5G20000 | 1.410000498 | 1.0141E-56 | 2.1564E-55 |
| AT5G20040 | -1.392121365 | 5.3637E-25 | 4.8181E-24 |
| AT5G20250 | -2.488033874 | 6.589E-155 | 5.062E-153 |
| AT5G20270 | 1.889111479 | 4.403E-68 | 1.156E-66 |
| AT5G20360 | 1.192127957 | 1.8017E-28 | 1.845E-27 |
| AT5G20380 | 1.552907758 | 6.3517E-68 | 1.6581E-66 |
| AT5G20520 | 1.252779188 | 5.0836E-43 | 7.8795E-42 |
| AT5G20540 | -1.039648795 | 2.1145E-09 | 8.6476E-09 |
| AT5G20630 | -1.924962171 | 8.053E-264 | 1.312E-261 |
| AT5G20640 | -4.596170858 | 5.8798E-06 | 1.7624E-05 |
| AT5G20720 | -1.162165342 | 9.5014E-97 | 3.9131E-95 |
| AT5G20740 | -2.268945187 | 3.7753E-26 | 3.5331E-25 |
| AT5G20790 | -4.320751081 | 1.414E-16 | 8.8339E-16 |
| AT5G20830 | 3.657474098 | 0 | 0 |
| AT5G20840 | 1.315832835 | 2.7382E-43 | 4.2704E-42 |
| AT5G20860 | -1.93761723 | 0.00101901 | 0.00229832 |
| AT5G20900 | 1.642499658 | 4.6018E-98 | 1.923E-96 |
| AT5G20910 | 1.720597768 | 9.2903E-35 | 1.1347E-33 |
| AT5G20980 | -1.000110448 | 1.2813E-09 | 5.3367E-09 |
| AT5G21020 | 1.543124253 | 6.5988E-96 | 2.6885E-94 |
| AT5G21080 | -3.077225499 | 0.00437538 | 0.00891486 |
| AT5G21930 | -1.099631595 | 3.3215E-25 | 3.0013E-24 |
| AT5G21940 | 1.329325537 | 1.3637E-45 | 2.2449E-44 |
| AT5G22000 | 1.133151624 | 6.3661E-47 | 1.0799E-45 |
| AT5G22110 | -1.300967304 | 1.2993E-07 | 4.5832E-07 |
| AT5G22120 | 1.590865927 | 7.8527E-38 | 1.0665E-36 |
| AT5G22140 | -1.156990284 | 6.7292E-16 | 4.0714E-15 |
| AT5G22220 | 1.632816203 | 3.3328E-17 | 2.1572E-16 |
| AT5G22270 | 1.585606107 | 7.4648E-19 | 5.2502E-18 |
| AT5G22290 | 2.131365028 | 5.2973E-65 | 1.3253E-63 |
| AT5G22410 | -6.109957225 | 5.1088E-05 | 0.00013817 |
| AT5G22460 | 4.778853489 | 4.601E-122 | 2.505E-120 |
| AT5G22470 | 10.01302148 | 1.4029E-11 | 6.6257E-11 |
| AT5G22500 | 2.069477115 | 1.5624E-70 | 4.2286E-69 |
| AT5G22545 | 8.004207026 | 5.733E-07 | 1.8968E-06 |
| AT5G22550 | -1.4585591 | 0.00614418 | 0.01218369 |
| AT5G22555 | -6.469356756 | 3.1144E-12 | 1.5288E-11 |
| AT5G22580 | -2.474375164 | 2.2024E-43 | 3.4442E-42 |
| AT5G22640 | -1.532791683 | 1.1791E-96 | 4.8384E-95 |
| AT5G22650 | -1.130565063 | 2.8692E-74 | 8.2552E-73 |
| AT5G22794 | 1.252910575 | 0.00283463 | 0.00596138 |
| AT5G22850 | 1.19506909 | 1.787E-27 | 1.7635E-26 |
| AT5G22860 | 2.48854299 | 9.958E-115 | 5.038E-113 |
| AT5G22890 | -2.401534643 | 9.3172E-17 | 5.8871E-16 |
| AT5G22930 | -3.421647842 | 2.0584E-21 | 1.6013E-20 |
| AT5G22940 | -2.520765628 | 1.4026E-21 | 1.099E-20 |
| AT5G23010 | -2.142829011 | 4.257E-205 | 4.738E-203 |
| AT5G23020 | -1.64008524 | 2.4525E-38 | 3.3628E-37 |
| AT5G23030 | -7.519224429 | 5.6515E-06 | 1.6986E-05 |
| AT5G23050 | 1.605742983 | 3.1775E-83 | 1.0522E-81 |
| AT5G23060 | -2.070874643 | 2.768E-171 | 2.467E-169 |
| AT5G23070 | -1.152424882 | 5.9304E-17 | 3.7848E-16 |
| AT5G23100 | -1.841679402 | 1.0406E-14 | 5.9268E-14 |
| AT5G23210 | -2.075523864 | 6.0502E-61 | 1.3944E-59 |
| AT5G23220 | 2.995394212 | 1.684E-104 | 7.532E-103 |
| AT5G23300 | -1.720043318 | 1.6271E-33 | 1.9245E-32 |
| AT5G23310 | -1.536944471 | 1.9757E-56 | 4.17E-55 |
| AT5G23340 | 1.111907465 | 1.5197E-15 | 9.0532E-15 |
| AT5G23380 | 2.144968753 | 4.9233E-23 | 4.1147E-22 |
| AT5G23400 | -1.391712074 | 2.8242E-09 | 1.1422E-08 |
| AT5G23410 | 1.536493749 | 1.5241E-15 | 9.0769E-15 |
| AT5G23420 | -1.128757711 | 1.9628E-15 | 1.162E-14 |
| AT5G23460 | 1.52284958 | 6.5634E-06 | 1.9575E-05 |
| AT5G23480 | -1.442000269 | 0.00011438 | 0.00029645 |
| AT5G23510 | -1.539979639 | 2.5384E-09 | 1.0316E-08 |
| AT5G23690 | -1.320865242 | 3.3561E-13 | 1.7478E-12 |
| AT5G23750 | 1.026232727 | 2.3691E-07 | 8.1503E-07 |
| AT5G23830 | -1.898429557 | 2.7475E-38 | 3.7604E-37 |
| AT5G23840 | -1.736409886 | 0.0003863 | 0.00092893 |
| AT5G23850 | 1.717078997 | 5.7593E-35 | 7.0992E-34 |
| AT5G23860 | -1.213758003 | 2.1353E-29 | 2.2462E-28 |
| AT5G23870 | -1.353264994 | 2.8947E-06 | 8.9408E-06 |
| AT5G23980 | -2.56659895 | 2.6944E-13 | 1.4126E-12 |
| AT5G23990 | -4.390077082 | 0.00277206 | 0.0058411 |
| AT5G24080 | 6.640096859 | 9.632E-169 | 8.42E-167 |
| AT5G24100 | -3.558971832 | 6.9991E-10 | 2.9771E-09 |
| AT5G24110 | 1.921221665 | 0.00166434 | 0.00363837 |
| AT5G24120 | 2.237167625 | 6.473E-118 | 3.372E-116 |
| AT5G24230 | -5.649863312 | 7.354E-16 | 4.4424E-15 |
| AT5G24290 | -1.058953583 | 4.7476E-07 | 1.5846E-06 |
| AT5G24313 | -4.447163509 | 0.00143966 | 0.00317609 |
| AT5G24330 | -1.948728493 | 1.0113E-06 | 3.2705E-06 |
| AT5G24380 | 1.243540798 | 6.8667E-46 | 1.1394E-44 |
| AT5G24490 | 1.053426456 | 1.1492E-52 | 2.243E-51 |
| AT5G24530 | 1.02356523 | 5.2646E-18 | 3.5538E-17 |
| AT5G24600 | 2.30712555 | 2.8912E-07 | 9.8575E-07 |
| AT5G24655 | 1.267456513 | 0.00073218 | 0.00168965 |
| AT5G24735 | 1.280731356 | 1.0134E-25 | 9.3498E-25 |
| AT5G24770 | 2.912712456 | 6.4225E-34 | 7.672E-33 |
| AT5G24780 | 1.053185003 | 0.00492002 | 0.00993321 |
| AT5G24800 | 2.478322032 | 1.466E-120 | 7.851E-119 |
| AT5G24860 | 3.563202841 | 2.1617E-09 | 8.8359E-09 |
| AT5G24870 | 1.208093182 | 1.3893E-10 | 6.1646E-10 |
| AT5G24880 | -2.504338233 | 4.7263E-05 | 0.00012854 |
| AT5G24890 | 1.333655674 | 1.4025E-27 | 1.3901E-26 |
| AT5G24900 | -2.740178293 | 0.00098459 | 0.0022264 |
| AT5G24930 | 1.36491983 | 2.157E-112 | 1.056E-110 |
| AT5G24940 | 2.096696373 | 1.4043E-13 | 7.4964E-13 |
| AT5G24960 | -2.468080124 | 0.00066712 | 0.00154607 |
| AT5G24990 | -1.09074001 | 1.7256E-06 | 5.4425E-06 |
| AT5G25110 | 3.439494575 | 3.0771E-84 | 1.0386E-82 |
| AT5G25220 | 1.312094679 | 5.1334E-63 | 1.2316E-61 |
| AT5G25240 | 2.037954989 | 1.1192E-13 | 6.0226E-13 |
| AT5G25250 | -1.985028056 | 4.2747E-14 | 2.3587E-13 |
| AT5G25260 | -3.318645234 | 3.344E-12 | 1.6366E-11 |
| AT5G25280 | 2.127791208 | 1.024E-217 | 1.223E-215 |
| AT5G25390 | 3.565815664 | 1.393E-05 | 4.023E-05 |
| AT5G25460 | -2.932676895 | 3.432E-267 | 5.8E-265 |
| AT5G25540 | 1.19216966 | 1.8089E-28 | 1.8516E-27 |
| AT5G25560 | 1.312903285 | 5.0862E-75 | 1.4821E-73 |
| AT5G25570 | 1.122928724 | 7.3798E-05 | 0.00019561 |
| AT5G25610 | 2.491851807 | 0 | 0 |
| AT5G04065 | 3.979723271 | 0.0087081 | 0.0167765 |
| AT5G25757 | -1.358258719 | 2.8826E-23 | 2.4332E-22 |
| AT5G25810 | -2.554284999 | 3.2359E-18 | 2.2072E-17 |
| AT5G25840 | -1.046409791 | 1.629E-09 | 6.7247E-09 |
| AT5G25880 | -1.388118508 | 0.00320535 | 0.00668434 |
| AT5G25920 | -6.551885087 | 0.00037852 | 0.00091139 |
| AT5G25930 | -1.006045505 | 8.9919E-09 | 3.4998E-08 |
| AT5G25970 | -3.360676919 | 5.5649E-13 | 2.8635E-12 |
| AT5G26040 | 1.059962404 | 1.3481E-09 | 5.6075E-09 |
| AT5G26180 | -1.212912532 | 5.9073E-17 | 3.7711E-16 |
| AT5G26270 | -1.878194269 | 1.138E-24 | 1.0123E-23 |
| AT5G26280 | -2.235867222 | 1.785E-157 | 1.399E-155 |
| AT5G26290 | -2.84723975 | 2.7134E-47 | 4.6549E-46 |
| AT5G26310 | -1.764694266 | 6.6741E-06 | 1.989E-05 |
| AT5G26330 | -1.307252077 | 3.908E-06 | 1.1914E-05 |
| AT5G26340 | 1.382586244 | 6.1307E-61 | 1.4115E-59 |
| AT5G26742 | -1.218604217 | 1.2466E-94 | 4.99E-93 |
| AT5G26670 | -1.669040479 | 5.9419E-12 | 2.8644E-11 |
| AT5G26760 | 1.048887478 | 1.112E-26 | 1.0643E-25 |
| AT5G26770 | 1.716990148 | 2.8676E-26 | 2.698E-25 |
| AT5G26920 | -1.007139723 | 2.1958E-05 | 6.2058E-05 |
| AT5G27110 | -1.042544177 | 0.00063807 | 0.00148326 |
| AT5G27120 | -1.317514487 | 1.1085E-63 | 2.6934E-62 |
| AT5G27150 | 1.512977869 | 7.3224E-84 | 2.4461E-82 |
| AT5G27280 | 1.92516216 | 2.0203E-47 | 3.471E-46 |
| AT5G27330 | -1.938334297 | 1.8672E-67 | 4.8412E-66 |
| AT5G27360 | -2.229446728 | 2.0573E-10 | 9.0477E-10 |
| AT5G27395 | -1.064783408 | 1.0962E-18 | 7.6437E-18 |
| AT5G27390 | -1.1077333 | 4.0159E-19 | 2.8579E-18 |
| AT5G27520 | 2.290854934 | 1.3586E-86 | 4.8357E-85 |
| AT5G27610 | 2.170181346 | 6.4192E-49 | 1.1451E-47 |
| AT5G27760 | 1.395157878 | 1.0381E-78 | 3.2006E-77 |
| AT5G27930 | 1.495347781 | 1.5958E-50 | 2.965E-49 |
| AT5G28010 | 1.09154089 | 0.00744581 | 0.0145299 |
| AT5G28030 | -1.530992724 | 9.1235E-06 | 2.6832E-05 |
| AT5G28500 | -1.206487681 | 2.7068E-73 | 7.6529E-72 |
| AT5G28510 | 2.087586682 | 6.1964E-24 | 5.3735E-23 |
| AT5G28520 | 6.987773689 | 8.6765E-10 | 3.6646E-09 |
| AT5G28540 | -1.139956613 | 2.5652E-49 | 4.6267E-48 |
| AT5G28630 | -4.548152694 | 4.7489E-13 | 2.4518E-12 |
| AT5G28770 | -1.4180772 | 3.615E-08 | 1.3387E-07 |
| AT5G28830 | 1.28635269 | 6.7867E-14 | 3.7018E-13 |
| AT5G29000 | 1.406846124 | 1.0185E-30 | 1.1108E-29 |
| AT5G32450 | -1.035553125 | 2.4022E-18 | 1.6533E-17 |
| AT5G33370 | -2.278393045 | 1.5577E-13 | 8.3002E-13 |
| AT5G35110 | 2.657911058 | 5.1799E-08 | 1.8911E-07 |
| AT5G35190 | -6.639460136 | 4.0301E-39 | 5.6239E-38 |
| AT5G35220 | -1.464406129 | 6.1774E-55 | 1.2595E-53 |
| AT5G35320 | 1.46138624 | 1.8754E-27 | 1.8484E-26 |
| AT5G35460 | 1.33703307 | 5.9724E-40 | 8.538E-39 |
| AT5G35480 | -2.368858294 | 0.00210546 | 0.00453405 |
| AT5G35735 | -1.007694357 | 5.415E-17 | 3.4685E-16 |
| AT5G35740 | -1.129679938 | 7.3135E-07 | 2.3961E-06 |
| AT5G35777 | -3.738090033 | 3.9523E-06 | 1.2041E-05 |
| AT5G35970 | 1.384749241 | 7.2489E-60 | 1.6343E-58 |
| AT5G36120 | -1.415680646 | 2.1263E-23 | 1.8068E-22 |
| AT5G36130 | -4.597598519 | 5.2749E-18 | 3.559E-17 |
| AT5G36140 | -5.773670468 | 1.3389E-21 | 1.0501E-20 |
| AT5G36150 | -3.997445223 | 1.9036E-13 | 1.0084E-12 |
| AT5G36180 | -3.794294137 | 7.1113E-33 | 8.295E-32 |
| AT5G36920 | 1.829755529 | 0.00117719 | 0.00263321 |
| AT5G36960 | -2.736744816 | 0.00600634 | 0.01193215 |
| AT5G37260 | 1.807163506 | 1.0235E-96 | 4.2077E-95 |
| AT5G37300 | 6.07011252 | 1.63E-162 | 1.328E-160 |
| AT5G37360 | 1.234746316 | 5.3646E-60 | 1.2107E-58 |
| AT5G37500 | 1.520667869 | 2.9524E-20 | 2.1949E-19 |
| AT5G37540 | 2.944555466 | 3.9519E-81 | 1.2646E-79 |
| AT5G37670 | 1.7883975 | 2.588E-06 | 8.026E-06 |
| AT5G37840 | -6.451583801 | 0.00057457 | 0.00134455 |
| AT5G37930 | 1.106205362 | 7.0536E-11 | 3.1849E-10 |
| AT5G38000 | -1.401872501 | 1.5831E-07 | 5.5426E-07 |
| AT5G38010 | -2.506589085 | 1.2116E-12 | 6.0997E-12 |
| AT5G38020 | -1.718327469 | 2.7269E-44 | 4.3569E-43 |
| AT5G38030 | -1.547910277 | 4.3033E-25 | 3.8716E-24 |
| AT5G38100 | -3.428847165 | 0.00050428 | 0.00119032 |
| AT5G38200 | 2.042373503 | 2.4484E-44 | 3.9174E-43 |
| AT5G38240 | 2.416571014 | 1.1906E-09 | 4.9698E-09 |
| AT5G38340 | -1.256079753 | 0.00081633 | 0.00187209 |
| AT5G38420 | -1.842918625 | 7.784E-228 | 1.003E-225 |
| AT5G38430 | -3.527926915 | 0 | 0 |
| AT5G38540 | -4.759799111 | 2.7585E-05 | 7.713E-05 |
| AT5G38550 | -1.759278203 | 2.3474E-06 | 7.3097E-06 |
| AT5G38720 | -1.42165139 | 1.8865E-28 | 1.9293E-27 |
| AT5G00550 | 3.054904045 | 0.00188543 | 0.00408412 |
| AT5G38747 | 2.123822532 | 0.00223481 | 0.00479585 |
| AT5G38890 | -1.098009523 | 7.7342E-12 | 3.7057E-11 |
| AT5G38895 | 2.008687362 | 6.0085E-22 | 4.7984E-21 |
| AT5G38900 | -2.223727122 | 1.7379E-17 | 1.1414E-16 |
| AT5G38910 | -1.526728694 | 0.00123955 | 0.00276322 |
| AT5G38940 | -1.502811027 | 6.7594E-18 | 4.536E-17 |
| AT5G38970 | -4.159879929 | 0.00016745 | 0.00042399 |
| AT5G38990 | -1.143624793 | 8.8369E-16 | 5.3143E-15 |
| AT5G39020 | -2.17775442 | 4.3898E-06 | 1.3317E-05 |
| AT5G00560 | 1.455682189 | 2.6983E-06 | 8.3522E-06 |
| AT5G39050 | 2.236132019 | 1.349E-176 | 1.236E-174 |
| AT5G39090 | 1.590590715 | 2.1356E-05 | 6.0432E-05 |
| AT5G39210 | -1.367095034 | 7.6914E-22 | 6.1018E-21 |
| AT5G39220 | 1.15924636 | 0.00081754 | 0.00187467 |
| AT5G39240 | -1.432197184 | 0.00161967 | 0.00354886 |
| AT5G00570 | -5.783505316 | 0.00541349 | 0.0108498 |
| AT5G39520 | 5.453985349 | 3.1497E-42 | 4.7845E-41 |
| AT5G39580 | -3.31779828 | 3.4579E-37 | 4.6003E-36 |
| AT5G39590 | 1.472173699 | 2.4796E-98 | 1.04E-96 |
| AT5G39610 | 3.732834699 | 6.075E-129 | 3.582E-127 |
| AT5G39660 | 2.01483471 | 2.0759E-22 | 1.6892E-21 |
| AT5G39720 | 5.403022766 | 1.047E-09 | 4.3903E-09 |
| AT5G39840 | -1.210226078 | 1.4357E-11 | 6.7748E-11 |
| AT5G39970 | -2.060535304 | 2.0513E-09 | 8.4086E-09 |
| AT5G40000 | 2.261877966 | 0.00094118 | 0.00213502 |
| AT5G40030 | -1.195166075 | 0.00477078 | 0.00966096 |
| AT5G40140 | -1.063374633 | 4.7182E-06 | 1.4289E-05 |
| AT5G40150 | -2.349229775 | 2.1348E-17 | 1.3961E-16 |
| AT5G40382 | 2.829081325 | 0.00605344 | 0.01201838 |
| AT5G40390 | 1.024937847 | 8.7118E-39 | 1.2047E-37 |
| AT5G40480 | -1.556552509 | 2.2947E-71 | 6.3079E-70 |
| AT5G40490 | -1.210015323 | 1.2207E-20 | 9.2074E-20 |
| AT5G40510 | -1.724963488 | 3.9071E-18 | 2.6539E-17 |
| AT5G40540 | 1.038602589 | 5.1307E-08 | 1.8742E-07 |
| AT5G40590 | -3.754671633 | 7.7212E-22 | 6.1233E-21 |
| AT5G40670 | 1.1574788 | 8.6281E-32 | 9.7454E-31 |
| AT5G40690 | 1.212345917 | 1.0503E-12 | 5.3241E-12 |
| AT5G40730 | 1.131318423 | 5.6029E-15 | 3.2429E-14 |
| AT5G40780 | -1.08173398 | 4.0631E-16 | 2.4894E-15 |
| AT5G40790 | 4.298760018 | 1.8617E-29 | 1.9656E-28 |
| AT5G40800 | 4.347376086 | 5.7605E-12 | 2.7799E-11 |
| AT5G40830 | -1.822053139 | 1.5613E-33 | 1.8476E-32 |
| AT5G40850 | -1.242407546 | 1.002E-57 | 2.1878E-56 |
| AT5G40880 | 1.246666927 | 2.57E-14 | 1.4368E-13 |
| AT5G40960 | 1.464054687 | 2.2413E-06 | 6.9956E-06 |
| AT5G40990 | -5.424388212 | 2.765E-09 | 1.1198E-08 |
| AT5G41040 | 1.964242031 | 1.1121E-86 | 3.9647E-85 |
| AT5G41050 | -1.044195939 | 1.3037E-06 | 4.1683E-06 |
| AT5G41090 | 3.635106125 | 0.00041777 | 0.00099882 |
| AT5G41110 | 1.1444089 | 5.7169E-09 | 2.2559E-08 |
| AT5G41140 | -1.311552646 | 7.1132E-24 | 6.1522E-23 |
| AT5G41300 | -6.84339772 | 0.00011462 | 0.00029697 |
| AT5G41340 | 1.020006965 | 1.6068E-18 | 1.1147E-17 |
| AT5G41350 | 1.269835831 | 2.1221E-18 | 1.4637E-17 |
| AT5G41460 | 1.028294931 | 7.8527E-16 | 4.7324E-15 |
| AT5G41560 | 1.170897232 | 5.9553E-11 | 2.7051E-10 |
| AT5G41670 | -1.210597716 | 2.1955E-61 | 5.122E-60 |
| AT5G41700 | 1.030183797 | 1.6793E-44 | 2.7059E-43 |
| AT5G41880 | -1.391418532 | 1.0391E-10 | 4.6505E-10 |
| AT5G41900 | 1.84953303 | 5.4329E-42 | 8.1982E-41 |
| AT5G42000 | 1.161785747 | 8.2272E-19 | 5.7704E-18 |
| AT5G42050 | 2.040339909 | 1.338E-270 | 2.313E-268 |
| AT5G42070 | -1.222210025 | 1.1249E-10 | 5.0238E-10 |
| AT5G42100 | -1.21307643 | 3.7885E-33 | 4.4488E-32 |
| AT5G42110 | -1.599578688 | 8.2516E-12 | 3.9436E-11 |
| AT5G42180 | -4.876063592 | 6.2424E-51 | 1.1732E-49 |
| AT5G42200 | 1.800687267 | 2.6454E-10 | 1.1574E-09 |
| AT5G42250 | -1.216424832 | 1.5195E-10 | 6.7267E-10 |
| AT5G42380 | 3.26024729 | 9.3421E-58 | 2.0436E-56 |
| AT5G42450 | -1.175409658 | 0.00122623 | 0.00273594 |
| AT5G42500 | -2.260840373 | 1.9392E-08 | 7.3497E-08 |
| AT5G42510 | 2.002207727 | 4.5901E-15 | 2.6676E-14 |
| AT5G42570 | 2.280120627 | 9.279E-124 | 5.151E-122 |
| AT5G42580 | -2.490454529 | 2.1685E-13 | 1.1434E-12 |
| AT5G42590 | -3.647706772 | 1.9545E-27 | 1.9229E-26 |
| AT5G42655 | -4.123243934 | 0.00726262 | 0.01420766 |
| AT5G42680 | -1.343267355 | 0.00170043 | 0.00371085 |
| AT5G42780 | -2.03433703 | 5.6084E-08 | 2.0431E-07 |
| AT5G42785 | -3.011974766 | 0.00024448 | 0.00060459 |
| AT5G42800 | 1.264250081 | 4.7473E-35 | 5.8802E-34 |
| AT5G42830 | -2.261129124 | 1.3451E-20 | 1.0115E-19 |
| AT5G42840 | -2.174056129 | 0.00029267 | 0.0007157 |
| AT5G42860 | -1.328254261 | 7.93E-12 | 3.7963E-11 |
| AT5G42900 | 1.893693525 | 1.146E-117 | 5.958E-116 |
| AT5G43020 | -1.201458492 | 4.4531E-15 | 2.5906E-14 |
| AT5G43030 | -1.820117685 | 0.00511298 | 0.01029817 |
| AT5G43040 | -1.779796475 | 6.0726E-05 | 0.00016277 |
| AT5G43150 | 2.696386839 | 2.647E-115 | 1.354E-113 |
| AT5G43180 | 1.753757153 | 8.0972E-26 | 7.5069E-25 |
| AT5G43230 | -3.590358981 | 0.00016221 | 0.00041171 |
| AT5G43250 | -2.303473106 | 1.2053E-09 | 5.0283E-09 |
| AT5G43260 | 2.186359796 | 3.336E-122 | 1.821E-120 |
| AT5G43300 | 3.255777376 | 2.74E-05 | 7.6641E-05 |
| AT5G43350 | -3.383089642 | 1.183E-141 | 7.893E-140 |
| AT5G43380 | 2.016627081 | 2.6163E-31 | 2.909E-30 |
| AT5G43403 | 1.324279684 | 2.4131E-09 | 9.823E-09 |
| AT5G43430 | 1.471526397 | 3.2736E-37 | 4.3653E-36 |
| AT5G43450 | 1.411620001 | 7.5535E-43 | 1.1668E-41 |
| AT5G43460 | 1.10983327 | 3.6657E-42 | 5.5462E-41 |
| AT5G43570 | -2.355783297 | 1.2198E-05 | 3.5417E-05 |
| AT5G43580 | -2.687439458 | 2.588E-110 | 1.243E-108 |
| AT5G43620 | 1.881744486 | 7.894E-09 | 3.0883E-08 |
| AT5G43770 | 4.052615375 | 0.00012667 | 0.00032672 |
| AT5G43840 | 6.483720508 | 1.8992E-12 | 9.4406E-12 |
| AT5G43850 | 2.331461604 | 1.301E-143 | 8.831E-142 |
| AT5G43870 | -1.181067592 | 1.0442E-11 | 4.9656E-11 |
| AT5G43930 | 1.048939012 | 1.7238E-17 | 1.1325E-16 |
| AT5G43935 | 2.77880087 | 0.00859886 | 0.01658143 |
| AT5G44005 | 1.718341409 | 6.3286E-24 | 5.4799E-23 |
| AT5G44020 | -3.763442773 | 0 | 0 |
| AT5G44040 | -1.02387004 | 1.0219E-09 | 4.2905E-09 |
| AT5G44050 | 1.03484825 | 3.3829E-06 | 1.0372E-05 |
| AT5G44130 | -2.352900623 | 3.7583E-86 | 1.3253E-84 |
| AT5G44160 | -1.045840911 | 0.0016166 | 0.00354249 |
| AT5G44190 | -1.1596135 | 1.2349E-19 | 8.9904E-19 |
| AT5G44260 | 2.998558586 | 3.3489E-27 | 3.2654E-26 |
| AT5G44310 | 4.978067173 | 1.9593E-22 | 1.5971E-21 |
| AT5G44350 | -1.663102423 | 9.7765E-06 | 2.8675E-05 |
| AT5G44370 | -1.275228553 | 2.9718E-11 | 1.3734E-10 |
| AT5G44380 | -2.07811682 | 5.7749E-71 | 1.578E-69 |
| AT5G44390 | -2.391183456 | 8.6872E-11 | 3.9071E-10 |
| AT5G44400 | -1.912558829 | 5.684E-59 | 1.2652E-57 |
| AT5G44460 | -2.626634171 | 0.00033707 | 0.00081727 |
| AT5G44480 | -2.144933247 | 1.7484E-26 | 1.6587E-25 |
| AT5G44550 | -1.793361307 | 1.6717E-19 | 1.2104E-18 |
| AT5G44572 | -1.362326171 | 0.00017242 | 0.00043556 |
| AT5G44574 | -3.347050673 | 0.00084694 | 0.00193663 |
| AT5G44578 | -1.456561284 | 0.00026691 | 0.00065667 |
| AT5G44610 | -1.189591139 | 3.4721E-08 | 1.2885E-07 |
| AT5G44635 | -1.857377284 | 1.0128E-28 | 1.048E-27 |
| AT5G44670 | 2.340657889 | 1.5672E-76 | 4.6682E-75 |
| AT5G44680 | -1.271987448 | 1.7078E-14 | 9.633E-14 |
| AT5G44700 | -2.635304314 | 0.00213955 | 0.0046044 |
| AT5G44770 | -5.727367411 | 0.00661264 | 0.01303898 |
| AT5G44780 | -1.230746855 | 0.00021776 | 0.00054199 |
| AT5G44820 | -1.601952467 | 0.0006398 | 0.00148713 |
| AT5G44900 | -5.551589425 | 8.691E-06 | 2.5623E-05 |
| AT5G44910 | -1.553054509 | 4.0639E-06 | 1.2363E-05 |
| AT5G44925 | -4.519642613 | 0.00179118 | 0.00389326 |
| AT5G45080 | -1.024595904 | 0.00445414 | 0.00905754 |
| AT5G45090 | -3.460687191 | 0.00031984 | 0.00077773 |
| AT5G45130 | 1.178870813 | 3.1652E-47 | 5.4177E-46 |
| AT5G45200 | -2.734170516 | 1.0363E-07 | 3.6903E-07 |
| AT5G45210 | -3.722280381 | 1.0514E-12 | 5.3287E-12 |
| AT5G45310 | 3.039330422 | 3.3274E-22 | 2.6826E-21 |
| AT5G45340 | 4.001469356 | 2.389E-106 | 1.092E-104 |
| AT5G45380 | -3.200604461 | 3.4141E-37 | 4.55E-36 |
| AT5G45460 | -1.038948336 | 0.00061646 | 0.00143639 |
| AT5G45570 | -6.253362302 | 0.00135689 | 0.00300397 |
| AT5G45630 | 7.893935531 | 1.694E-130 | 1.012E-128 |
| AT5G45670 | -1.395756661 | 3.0392E-25 | 2.7484E-24 |
| AT5G45690 | 5.998077051 | 4.3417E-17 | 2.7936E-16 |
| AT5G45700 | -1.516620987 | 2.4426E-11 | 1.1348E-10 |
| AT5G45810 | 1.845243103 | 1.0029E-08 | 3.8857E-08 |
| AT5G45850 | -2.863873889 | 1.665E-07 | 5.8105E-07 |
| AT5G45900 | 1.058222807 | 6.7585E-14 | 3.6881E-13 |
| AT5G45950 | 2.537282132 | 1.8522E-30 | 1.9991E-29 |
| AT5G46180 | 1.208827772 | 1.1454E-34 | 1.396E-33 |
| AT5G46280 | -1.271571847 | 1.2282E-21 | 9.6493E-21 |
| AT5G46330 | -1.403565218 | 6.051E-07 | 1.9962E-06 |
| AT5G46410 | 1.822458695 | 1.0523E-62 | 2.5166E-61 |
| AT5G46490 | -1.028206756 | 3.3553E-05 | 9.2748E-05 |
| AT5G46580 | -2.158142452 | 3.017E-156 | 2.341E-154 |
| AT5G46690 | -1.032517484 | 2.0808E-10 | 9.1457E-10 |
| AT5G46790 | -1.466014891 | 1.9226E-25 | 1.7518E-24 |
| AT5G46825 | 5.729903235 | 0.00591105 | 0.0117541 |
| AT5G47000 | -2.319390673 | 0.00719481 | 0.01408466 |
| AT5G47020 | 1.861903063 | 3.626E-125 | 2.033E-123 |
| AT5G47040 | 2.020297814 | 1.074E-218 | 1.296E-216 |
| AT5G47050 | 1.162895548 | 2.4935E-17 | 1.6264E-16 |
| AT5G47060 | 1.542333315 | 9.3588E-50 | 1.7069E-48 |
| AT5G47070 | 1.176301756 | 6.4921E-12 | 3.1204E-11 |
| AT5G47120 | 1.396557798 | 1.9128E-88 | 7.0277E-87 |
| AT5G47180 | 1.488645296 | 1.7115E-36 | 2.2263E-35 |
| AT5G47200 | 1.371816005 | 1.4312E-91 | 5.4882E-90 |
| AT5G47240 | 1.380742471 | 8.6352E-23 | 7.1333E-22 |
| AT5G47330 | 2.786595755 | 4.544E-144 | 3.113E-142 |
| AT5G47370 | 1.538948436 | 8.6979E-24 | 7.4972E-23 |
| AT5G47380 | -1.301187866 | 2.3301E-07 | 8.0224E-07 |
| AT5G47430 | 1.05994526 | 2.6676E-34 | 3.2203E-33 |
| AT5G47450 | 1.431896861 | 1.82E-08 | 6.9128E-08 |
| AT5G47530 | 1.931699665 | 6.6723E-15 | 3.8482E-14 |
| AT5G47550 | 2.77992197 | 3.244E-192 | 3.38E-190 |
| AT5G47560 | 2.890132608 | 0 | 0 |
| AT5G47590 | 1.612837368 | 3.5487E-05 | 9.7906E-05 |
| AT5G47600 | 1.520130189 | 0.00453691 | 0.00921518 |
| AT5G47610 | 1.84385448 | 8.8174E-42 | 1.3218E-40 |
| AT5G47640 | 2.619787478 | 8.685E-190 | 8.886E-188 |
| AT5G47730 | 1.221450677 | 3.4018E-28 | 3.448E-27 |
| AT5G47880 | 2.07911483 | 1.184E-212 | 1.386E-210 |
| AT5G47910 | -1.207829241 | 3.3985E-25 | 3.0673E-24 |
| AT5G47950 | -3.661035082 | 3.9847E-10 | 1.7206E-09 |
| AT5G47960 | -1.88046252 | 0.00017125 | 0.00043294 |
| AT5G47980 | -5.56483698 | 1.7345E-45 | 2.845E-44 |
| AT5G47990 | -2.965296709 | 2.7146E-27 | 2.6503E-26 |
| AT5G48000 | -2.746878627 | 1.313E-130 | 7.882E-129 |
| AT5G48010 | -2.753824247 | 9.451E-121 | 5.074E-119 |
| AT5G48110 | -3.279637681 | 1.6376E-26 | 1.5562E-25 |
| AT5G48180 | 2.825241339 | 0 | 0 |
| AT5G48220 | -1.002527798 | 9.0284E-18 | 6.0196E-17 |
| AT5G48290 | -1.504857159 | 2.7499E-08 | 1.0291E-07 |
| AT5G48320 | -1.666143965 | 0.00015778 | 0.00040105 |
| AT5G48360 | -1.276507314 | 3.468E-29 | 3.6347E-28 |
| AT5G48370 | 1.152079924 | 1.2038E-14 | 6.8359E-14 |
| AT5G48410 | -1.624283533 | 9.8561E-06 | 2.8901E-05 |
| AT5G48412 | 2.021043688 | 1.4387E-86 | 5.105E-85 |
| AT5G48430 | -5.395475 | 4.854E-108 | 2.279E-106 |
| AT5G48450 | -1.244301653 | 2.5714E-08 | 9.6414E-08 |
| AT5G48460 | -1.041439425 | 1.6752E-09 | 6.9105E-09 |
| AT5G48650 | 1.396450412 | 2.4081E-08 | 9.0605E-08 |
| AT5G48655 | 1.262678653 | 1.0058E-29 | 1.0688E-28 |
| AT5G48657 | -1.230279217 | 2.0951E-05 | 5.9358E-05 |
| AT5G48660 | -1.071532396 | 0.00011242 | 0.00029161 |
| AT5G48730 | -1.361725898 | 1.3296E-14 | 7.5333E-14 |
| AT5G48800 | -1.245975842 | 4.2732E-06 | 1.2969E-05 |
| AT5G48830 | -1.498353106 | 4.002E-30 | 4.2788E-29 |
| AT5G48840 | -1.063924441 | 1.1928E-08 | 4.5895E-08 |
| AT5G48850 | 2.64528214 | 2.698E-149 | 1.923E-147 |
| AT5G48880 | -1.639397121 | 9.2632E-80 | 2.9032E-78 |
| AT5G49080 | -4.529450044 | 7.6405E-31 | 8.3649E-30 |
| AT5G49120 | 4.188740065 | 1.0634E-25 | 9.7992E-25 |
| AT5G49170 | -1.309243184 | 0.00933319 | 0.01786761 |
| AT5G49215 | -1.117848605 | 1.3383E-07 | 4.7178E-07 |
| AT5G49270 | -5.31883338 | 7.9299E-25 | 7.0841E-24 |
| AT5G49280 | 1.189852954 | 2.3231E-15 | 1.3699E-14 |
| AT5G49290 | 1.17607462 | 0.00446521 | 0.00907762 |
| AT5G49300 | -1.343893901 | 2.1186E-05 | 5.9995E-05 |
| AT5G49330 | -1.097026812 | 1.7909E-08 | 6.8056E-08 |
| AT5G49360 | -2.156028492 | 8.4549E-25 | 7.5443E-24 |
| AT5G49440 | -1.265450962 | 3.9704E-51 | 7.5178E-50 |
| AT5G49450 | 1.424999015 | 6.9452E-15 | 4.0025E-14 |
| AT5G49520 | 1.093139811 | 7.307E-07 | 2.3943E-06 |
| AT5G49525 | 2.046694556 | 1.5191E-06 | 4.8119E-06 |
| AT5G49560 | -2.656153294 | 9.6716E-17 | 6.1076E-16 |
| AT5G49630 | -4.424312014 | 5.6806E-64 | 1.3966E-62 |
| AT5G49700 | 1.212835363 | 0.00022429 | 0.00055746 |
| AT5G49710 | 1.1328959 | 9.1911E-15 | 5.2584E-14 |
| AT5G49730 | -1.663588677 | 3.4227E-07 | 1.1591E-06 |
| AT5G49740 | -1.984780448 | 2.0222E-12 | 1.0043E-11 |
| AT5G49760 | -1.606708383 | 1.62E-53 | 3.2029E-52 |
| AT5G49770 | -2.407739516 | 2.9807E-09 | 1.202E-08 |
| AT5G49780 | -2.462055391 | 5.3455E-09 | 2.1145E-08 |
| AT5G49800 | -1.176128034 | 0.0005438 | 0.00127727 |
| AT5G49870 | -3.442829118 | 3.8649E-05 | 0.00010617 |
| AT5G49900 | 1.19210529 | 2.5877E-19 | 1.8572E-18 |
| AT5G49910 | -1.834672427 | 7.513E-141 | 4.898E-139 |
| AT5G49930 | 1.081277394 | 2.024E-29 | 2.1311E-28 |
| AT5G50010 | -1.518117731 | 2.5895E-09 | 1.0513E-08 |
| AT5G50100 | 2.147457659 | 8.7521E-83 | 2.8732E-81 |
| AT5G50170 | 1.617935927 | 1.5433E-28 | 1.584E-27 |
| AT5G50200 | -1.635259059 | 3.2306E-50 | 5.9635E-49 |
| AT5G50240 | 2.515091399 | 9.451E-55 | 1.9202E-53 |
| AT5G50300 | -1.399867499 | 0.00021789 | 0.00054226 |
| AT5G50350 | 1.021871743 | 3.0288E-15 | 1.7747E-14 |
| AT5G50360 | 8.051676545 | 0 | 0 |
| AT5G50420 | -1.218971726 | 9.8249E-15 | 5.6055E-14 |
| AT5G50720 | 2.385639469 | 7.3504E-29 | 7.6404E-28 |
| AT5G50740 | -1.808454842 | 2.2288E-35 | 2.7941E-34 |
| AT5G50760 | -1.809334401 | 4.604E-12 | 2.2345E-11 |
| AT5G50770 | 4.036268654 | 0.00033523 | 0.00081299 |
| AT5G50780 | 1.368381808 | 3.9226E-24 | 3.4212E-23 |
| AT5G50890 | -1.035326091 | 0.00035145 | 0.00084998 |
| AT5G50915 | -1.229815514 | 1.8135E-07 | 6.3016E-07 |
| AT5G50950 | 1.258227771 | 9.0906E-65 | 2.2594E-63 |
| AT5G51070 | 2.290271469 | 3.1829E-42 | 4.8286E-41 |
| AT5G51260 | 1.341777113 | 3.8197E-13 | 1.9834E-12 |
| AT5G51310 | -1.553183758 | 0.00536968 | 0.01077147 |
| AT5G51460 | -1.781217112 | 5.7423E-23 | 4.7781E-22 |
| AT5G51465 | -4.471590523 | 0.00134005 | 0.00296985 |
| AT5G51470 | -2.856409794 | 0.00326555 | 0.00679931 |
| AT5G51490 | -4.417847507 | 4.7485E-10 | 2.0427E-09 |
| AT5G51520 | -4.336313686 | 0.00224202 | 0.00480725 |
| AT5G51540 | -1.876123899 | 8.1312E-22 | 6.4417E-21 |
| AT5G51550 | -1.7905589 | 5.7414E-64 | 1.4101E-62 |
| AT5G51640 | 1.04541468 | 1.281E-07 | 4.521E-07 |
| AT5G51710 | 1.05862614 | 4.6394E-14 | 2.5556E-13 |
| AT5G51720 | -2.068068761 | 1.182E-05 | 3.4381E-05 |
| AT5G51740 | 1.484965206 | 2.4662E-22 | 1.9989E-21 |
| AT5G51760 | 10.06351095 | 1.0676E-11 | 5.0727E-11 |
| AT5G51780 | -2.763704701 | 3.2578E-18 | 2.2195E-17 |
| AT5G51795 | -2.567468754 | 2.3691E-08 | 8.9211E-08 |
| AT5G51830 | 1.060203052 | 1.6482E-36 | 2.1464E-35 |
| AT5G51850 | -2.092018161 | 0.00109697 | 0.00246343 |
| AT5G51930 | -6.360437859 | 0.00082995 | 0.00190065 |
| AT5G51990 | 8.287808685 | 4.6842E-15 | 2.7208E-14 |
| AT5G52010 | -1.55712847 | 3.0556E-32 | 3.4928E-31 |
| AT5G52050 | 2.04606372 | 2.8955E-31 | 3.2132E-30 |
| AT5G00650 | -5.83141107 | 8.3388E-07 | 2.718E-06 |
| AT5G52230 | 1.156313021 | 1.2846E-08 | 4.9303E-08 |
| AT5G52280 | 1.06287285 | 1.807E-25 | 1.6484E-24 |
| AT5G52300 | 9.843358605 | 0 | 0 |
| AT5G52310 | 4.005350638 | 0 | 0 |
| AT5G52380 | -1.441319978 | 1.3409E-17 | 8.8654E-17 |
| AT5G52390 | 1.762313 | 0.00098313 | 0.00222332 |
| AT5G52550 | 1.366496078 | 5.4032E-32 | 6.1303E-31 |
| AT5G52570 | 2.13935581 | 1.2105E-37 | 1.6352E-36 |
| AT5G52580 | 1.477194275 | 6.2131E-54 | 1.2413E-52 |
| AT5G52660 | 2.173638763 | 8.2962E-64 | 2.0266E-62 |
| AT5G52670 | -6.649323651 | 0.00028426 | 0.00069617 |
| AT5G52750 | 1.411666419 | 5.8201E-05 | 0.00015641 |
| AT5G52760 | 4.774206502 | 1.1043E-06 | 3.5507E-06 |
| AT5G52780 | -2.362099256 | 2.7145E-66 | 6.9277E-65 |
| AT5G52810 | 1.433003728 | 1.2599E-31 | 1.414E-30 |
| AT5G52830 | -1.382159509 | 1.9244E-06 | 6.0412E-06 |
| AT5G52850 | -1.041697335 | 8.9117E-05 | 0.0002335 |
| AT5G52882 | -1.064730608 | 1.9992E-25 | 1.8202E-24 |
| AT5G52910 | -1.140139421 | 3.9519E-06 | 1.2041E-05 |
| AT5G53070 | -1.315149837 | 2.1656E-19 | 1.5596E-18 |
| AT5G53120 | 2.714459687 | 1.823E-253 | 2.81E-251 |
| AT5G53130 | 1.078896555 | 3.7688E-28 | 3.8116E-27 |
| AT5G53160 | -1.085465234 | 4.4641E-26 | 4.1658E-25 |
| AT5G53220 | 1.43650777 | 4.5108E-12 | 2.1907E-11 |
| AT5G53250 | -3.043775421 | 1.5959E-11 | 7.4997E-11 |
| AT5G53320 | -1.497060217 | 3.4748E-06 | 1.064E-05 |
| AT5G53330 | 1.138538021 | 2.7707E-23 | 2.3413E-22 |
| AT5G53360 | 1.508199289 | 2.739E-08 | 1.0255E-07 |
| AT5G53390 | 1.921420394 | 1.1404E-06 | 3.6607E-06 |
| AT5G53420 | 1.115932986 | 1.4326E-43 | 2.2496E-42 |
| AT5G53450 | 2.611603169 | 8.8771E-08 | 3.1801E-07 |
| AT5G53486 | -2.843426957 | 3.938E-15 | 2.2962E-14 |
| AT5G53500 | -1.156821059 | 7.4793E-17 | 4.7481E-16 |
| AT5G53590 | 2.225618253 | 4.5592E-77 | 1.3687E-75 |
| AT5G53660 | 2.405709405 | 1.19E-13 | 6.3855E-13 |
| AT5G53710 | 7.773524112 | 5.9696E-81 | 1.8996E-79 |
| AT5G53810 | 1.652179688 | 0.00928808 | 0.01779319 |
| AT5G53820 | 2.357286381 | 9.1497E-05 | 0.00023943 |
| AT5G53830 | 1.152393234 | 9.2802E-08 | 3.3162E-07 |
| AT5G53870 | 4.129726544 | 4.7265E-47 | 8.0358E-46 |
| AT5G53880 | -1.224105192 | 1.2398E-23 | 1.0623E-22 |
| AT5G53970 | 2.57363407 | 9.59E-157 | 7.493E-155 |
| AT5G53980 | -2.410165851 | 0.00601501 | 0.01194668 |
| AT5G54040 | -2.464864395 | 6.4168E-07 | 2.1123E-06 |
| AT5G54050 | -6.749449819 | 0.00017123 | 0.00043293 |
| AT5G54080 | 2.731237128 | 2.014E-123 | 1.112E-121 |
| AT5G54140 | -1.054084053 | 0.00066078 | 0.001532 |
| AT5G54165 | 6.074997126 | 4.934E-171 | 4.363E-169 |
| AT5G54230 | 3.157219485 | 1.8105E-48 | 3.1972E-47 |
| AT5G54240 | 2.945468131 | 4.3918E-32 | 4.9977E-31 |
| AT5G54300 | 2.623954253 | 2.4787E-60 | 5.6273E-59 |
| AT5G54380 | -2.291911326 | 2.9322E-65 | 7.3519E-64 |
| AT5G54470 | 1.643465826 | 5.0835E-05 | 0.00013759 |
| AT5G54585 | 4.240285805 | 1.973E-113 | 9.724E-112 |
| AT5G54630 | -1.234057634 | 2.6226E-12 | 1.2912E-11 |
| AT5G54720 | -1.396836903 | 0.00743253 | 0.01450895 |
| AT5G54730 | 1.942402639 | 3.6826E-72 | 1.0247E-70 |
| AT5G54840 | 1.955471638 | 5.1003E-62 | 1.2059E-60 |
| AT5G54860 | 1.102464245 | 6.8146E-20 | 4.9946E-19 |
| AT5G54870 | 1.100507737 | 7.2559E-31 | 7.9553E-30 |
| AT5G54910 | -1.078930362 | 2.3545E-18 | 1.6215E-17 |
| AT5G54940 | 1.107861612 | 8.4923E-57 | 1.8075E-55 |
| AT5G54970 | -1.40055997 | 3.465E-17 | 2.2402E-16 |
| AT5G54980 | 1.021025906 | 4.7948E-07 | 1.5996E-06 |
| AT5G55050 | -1.079129298 | 3.9281E-05 | 0.00010783 |
| AT5G55110 | -2.48005672 | 8.3282E-08 | 2.9891E-07 |
| AT5G55180 | 1.434491261 | 3.5129E-59 | 7.8426E-58 |
| AT5G55250 | -1.086121012 | 0.00302439 | 0.00633186 |
| AT5G55280 | -1.03949077 | 1.2788E-40 | 1.8608E-39 |
| AT5G55400 | 1.84098069 | 3.1622E-64 | 7.8083E-63 |
| AT5G55460 | 1.8410736 | 0.00532964 | 0.01069866 |
| AT5G55470 | 2.385692067 | 1.4731E-17 | 9.7168E-17 |
| AT5G55510 | -1.194810092 | 1.6239E-14 | 9.1642E-14 |
| AT5G55580 | -1.902786468 | 8.8703E-33 | 1.032E-31 |
| AT5G55620 | -1.455367977 | 9.2836E-24 | 7.993E-23 |
| AT5G55640 | 1.01505117 | 2.6651E-07 | 9.1199E-07 |
| AT5G55700 | 1.461335232 | 2.6836E-37 | 3.5827E-36 |
| AT5G55720 | -2.213134778 | 0.00176088 | 0.00383435 |
| AT5G55730 | -1.657996758 | 4.6693E-49 | 8.3687E-48 |
| AT5G55740 | -1.190549757 | 5.6864E-14 | 3.1203E-13 |
| AT5G55830 | -1.037025904 | 0.00951514 | 0.01819914 |
| AT5G55850 | 1.164797907 | 6.9383E-55 | 1.4122E-53 |
| AT5G55900 | 1.086101623 | 5.6883E-05 | 0.00015305 |
| AT5G55920 | -1.546604226 | 5.5407E-84 | 1.8618E-82 |
| AT5G55970 | 1.031124931 | 1.6162E-08 | 6.1591E-08 |
| AT5G56040 | -1.750324006 | 8.3134E-59 | 1.8433E-57 |
| AT5G56080 | -3.1269761 | 6.7608E-90 | 2.5454E-88 |
| AT5G56150 | 1.605599838 | 1.0812E-72 | 3.0343E-71 |
| AT5G56160 | 1.986708664 | 8.8677E-18 | 5.9142E-17 |
| AT5G56180 | 1.149217374 | 2.5051E-17 | 1.6335E-16 |
| AT5G56190 | 1.53707623 | 2.6454E-42 | 4.032E-41 |
| AT5G56210 | 1.542143689 | 9.7068E-26 | 8.9701E-25 |
| AT5G56320 | -2.735448314 | 1.8098E-21 | 1.4093E-20 |
| AT5G56340 | 1.597994721 | 5.6239E-37 | 7.4128E-36 |
| AT5G56520 | 2.457962936 | 8.5668E-39 | 1.1853E-37 |
| AT5G56530 | -1.476103441 | 1.1915E-21 | 9.3681E-21 |
| AT5G56750 | 1.810544065 | 5.4751E-58 | 1.2012E-56 |
| AT5G56840 | -2.595879523 | 5.5422E-07 | 1.8363E-06 |
| AT5G56850 | -1.587356922 | 1.7385E-30 | 1.8799E-29 |
| AT5G56860 | -2.527616251 | 1.8789E-78 | 5.7776E-77 |
| AT5G56870 | -2.513151967 | 3.8444E-36 | 4.9555E-35 |
| AT5G56970 | -3.507227228 | 0.00524925 | 0.01055307 |
| AT5G57040 | 2.695762797 | 4.865E-308 | 1.057E-305 |
| AT5G57050 | 4.466199359 | 0 | 0 |
| AT5G57070 | -1.116683564 | 3.9667E-18 | 2.6912E-17 |
| AT5G57090 | -1.276540089 | 7.5559E-24 | 6.5301E-23 |
| AT5G57100 | 1.303007327 | 1.3984E-16 | 8.7437E-16 |
| AT5G57120 | -1.308776865 | 1.9729E-40 | 2.8581E-39 |
| AT5G57180 | -1.516812321 | 6.2234E-57 | 1.3308E-55 |
| AT5G57220 | -3.52403676 | 2.887E-120 | 1.539E-118 |
| AT5G57240 | 2.356613988 | 1.9232E-08 | 7.2902E-08 |
| AT5G57350 | 1.421411517 | 3.025E-114 | 1.517E-112 |
| AT5G57390 | 1.141029706 | 4.3629E-07 | 1.463E-06 |
| AT5G57490 | -1.066024951 | 2.0273E-24 | 1.7887E-23 |
| AT5G57500 | -1.305755497 | 0.00678514 | 0.01335489 |
| AT5G57530 | -6.56663432 | 4.9167E-09 | 1.9516E-08 |
| AT5G57540 | -6.421113669 | 1.5519E-08 | 5.9203E-08 |
| AT5G57560 | 1.541034613 | 3.678E-33 | 4.3212E-32 |
| AT5G57565 | 1.83134571 | 2.5603E-17 | 1.6676E-16 |
| AT5G57570 | -2.575082736 | 2.7595E-09 | 1.1177E-08 |
| AT5G57610 | 2.683540738 | 9.111E-219 | 1.106E-216 |
| AT5G57620 | -1.604833501 | 0.00016283 | 0.0004132 |
| AT5G57625 | -6.216152809 | 1.2309E-32 | 1.427E-31 |
| AT5G57660 | 1.160216588 | 1.2357E-21 | 9.7016E-21 |
| AT5G57685 | -1.402389337 | 7.9443E-12 | 3.8024E-11 |
| AT5G57770 | -3.248725857 | 5.674E-06 | 1.7041E-05 |
| AT5G57780 | -2.63850285 | 2.0907E-11 | 9.7467E-11 |
| AT5G57785 | -1.451853228 | 1.0937E-22 | 9.0088E-22 |
| AT5G57790 | 1.41467219 | 3.5353E-11 | 1.6272E-10 |
| AT5G57800 | 1.937499894 | 4.215E-107 | 1.955E-105 |
| AT5G57810 | 2.775299731 | 3.9736E-07 | 1.3372E-06 |
| AT5G57860 | 1.007245834 | 5.7622E-12 | 2.7801E-11 |
| AT5G57900 | 2.256614077 | 3.246E-111 | 1.569E-109 |
| AT5G57910 | 2.139890417 | 5.0939E-44 | 8.0879E-43 |
| AT5G58010 | -2.377953569 | 3.5715E-07 | 1.2079E-06 |
| AT5G58040 | 1.030858335 | 2.7239E-40 | 3.9385E-39 |
| AT5G58070 | 2.144665266 | 1.575E-275 | 2.853E-273 |
| AT5G58160 | 1.932113353 | 1.5013E-74 | 4.3358E-73 |
| AT5G58250 | -1.160054848 | 7.1331E-56 | 1.4904E-54 |
| AT5G58320 | 1.129799754 | 3.0097E-18 | 2.0566E-17 |
| AT5G58370 | -1.639973638 | 2.3315E-23 | 1.9775E-22 |
| AT5G58380 | 1.681033952 | 5.5346E-56 | 1.1574E-54 |
| AT5G58480 | -1.482949228 | 2.9043E-14 | 1.6178E-13 |
| AT5G58500 | -2.148390162 | 0.00024036 | 0.00059486 |
| AT5G58575 | 1.243938495 | 9.3726E-13 | 4.7723E-12 |
| AT5G58650 | 1.887199861 | 4.4226E-15 | 2.5735E-14 |
| AT5G58660 | 1.174595384 | 0.00276513 | 0.00582917 |
| AT5G58700 | 1.277495785 | 1.3083E-29 | 1.3858E-28 |
| AT5G58720 | 1.172605041 | 8.0025E-35 | 9.8006E-34 |
| AT5G58730 | 1.314713801 | 1.3883E-20 | 1.0426E-19 |
| AT5G58750 | -4.649133642 | 1.4999E-11 | 7.0681E-11 |
| AT5G58780 | 1.370856078 | 0.0001081 | 0.00028106 |
| AT5G58784 | -2.952829494 | 0.00518899 | 0.0104393 |
| AT5G59010 | -1.026304264 | 5.0641E-12 | 2.4526E-11 |
| AT5G59020 | -1.984027562 | 1.1011E-15 | 6.5906E-15 |
| AT5G59030 | 1.191832009 | 2.6137E-20 | 1.9482E-19 |
| AT5G59090 | -1.274113252 | 7.3857E-18 | 4.9446E-17 |
| AT5G59220 | 6.34492797 | 0 | 0 |
| AT5G59230 | 4.781394757 | 1.1822E-06 | 3.789E-06 |
| AT5G59260 | -3.466768162 | 0.00355967 | 0.00736198 |
| AT5G59270 | -5.66853383 | 0.00851157 | 0.01643256 |
| AT5G59310 | 8.267732735 | 3.786E-243 | 5.537E-241 |
| AT5G59320 | 6.071311688 | 0 | 0 |
| AT5G59340 | 2.744564561 | 2.4609E-08 | 9.2516E-08 |
| AT5G59480 | 2.035434873 | 5.809E-124 | 3.233E-122 |
| AT5G59490 | 3.030481338 | 2.4432E-36 | 3.169E-35 |
| AT5G59520 | -3.030075185 | 4.0149E-69 | 1.0676E-67 |
| AT5G59530 | -1.51150222 | 1.1252E-09 | 4.7097E-09 |
| AT5G59550 | 1.479226665 | 6.8093E-35 | 8.3573E-34 |
| AT5G59570 | 2.387528255 | 1.579E-100 | 6.797E-99 |
| AT5G59680 | -3.725986947 | 2.8963E-20 | 2.1553E-19 |
| AT5G59720 | 2.668478114 | 6.9457E-30 | 7.4053E-29 |
| AT5G59770 | -1.208138778 | 1.3957E-17 | 9.222E-17 |
| AT5G59780 | -1.037823907 | 3.5391E-05 | 9.7652E-05 |
| AT5G59820 | 2.188517803 | 1.948E-71 | 5.368E-70 |
| AT5G59840 | 1.285608073 | 2.2023E-25 | 2.0035E-24 |
| AT5G59845 | 2.820510237 | 3.745E-18 | 2.5445E-17 |
| AT5G59850 | -1.178360395 | 1.2702E-53 | 2.52E-52 |
| AT5G59930 | -2.013656643 | 0.00377864 | 0.007781 |
| AT5G59940 | -2.175735162 | 0.00333124 | 0.00692093 |
| AT5G59960 | 1.662345769 | 3.7802E-62 | 8.975E-61 |
| AT5G60270 | -1.157514392 | 4.2004E-06 | 1.2759E-05 |
| AT5G60360 | 2.044082164 | 0 | 0 |
| AT5G60430 | 1.461108448 | 9.5134E-08 | 3.3968E-07 |
| AT5G60490 | -2.208203886 | 2.8901E-09 | 1.1667E-08 |
| AT5G60520 | -6.09557091 | 4.2119E-13 | 2.1826E-12 |
| AT5G60530 | -2.809803848 | 4.2841E-52 | 8.2976E-51 |
| AT5G60580 | 1.13006575 | 4.3096E-18 | 2.9186E-17 |
| AT5G60590 | -1.233159848 | 4.3261E-10 | 1.8648E-09 |
| AT5G60610 | 2.081589868 | 0.00036474 | 0.00088054 |
| AT5G60660 | -1.582619882 | 2.4151E-09 | 9.8292E-09 |
| AT5G60670 | -1.607479139 | 3.9816E-64 | 9.7999E-63 |
| AT5G60680 | 2.881318341 | 4.998E-148 | 3.541E-146 |
| AT5G60730 | -1.744766619 | 1.9712E-09 | 8.0974E-09 |
| AT5G60760 | 2.576627155 | 2.6954E-48 | 4.7417E-47 |
| AT5G60790 | 1.233210104 | 3.818E-106 | 1.742E-104 |
| AT5G60850 | 1.015449908 | 5.0569E-12 | 2.4496E-11 |
| AT5G60860 | 1.135039207 | 2.2905E-05 | 6.4603E-05 |
| AT5G60890 | -1.117985347 | 9.4149E-25 | 8.3976E-24 |
| AT5G60910 | 2.185341832 | 7.0791E-06 | 2.1042E-05 |
| AT5G61000 | -1.404204019 | 3.4537E-17 | 2.2336E-16 |
| AT5G61020 | -1.012964235 | 6.4543E-48 | 1.1216E-46 |
| AT5G61030 | -1.442389082 | 1.7616E-48 | 3.1156E-47 |
| AT5G61130 | -1.619950324 | 5.9374E-50 | 1.0899E-48 |
| AT5G61160 | -3.240039433 | 3.2715E-07 | 1.1101E-06 |
| AT5G61170 | -1.19398128 | 9.7067E-76 | 2.865E-74 |
| AT5G61350 | -2.199534257 | 1.677E-05 | 4.8001E-05 |
| AT5G61390 | 1.041344487 | 4.3027E-07 | 1.4435E-06 |
| AT5G61420 | -2.572434191 | 1.765E-106 | 8.087E-105 |
| AT5G61440 | -2.387985714 | 1.1942E-22 | 9.8296E-22 |
| AT5G61450 | 1.209702366 | 1.2582E-20 | 9.4835E-20 |
| AT5G61530 | 1.57431088 | 1.6842E-64 | 4.1767E-63 |
| AT5G61610 | -4.421916643 | 0.0015826 | 0.00347365 |
| AT5G61670 | 1.031213722 | 5.1169E-23 | 4.2686E-22 |
| AT5G61770 | -1.125123138 | 8.9288E-22 | 7.0516E-21 |
| AT5G61790 | -1.840616022 | 8.8131E-31 | 9.6441E-30 |
| AT5G61810 | 1.691742257 | 2.6108E-73 | 7.3906E-72 |
| AT5G61820 | 2.373899772 | 4.244E-253 | 6.499E-251 |
| AT5G61850 | 3.066268047 | 1.0803E-05 | 3.1539E-05 |
| AT5G61865 | 1.362018733 | 9.4662E-05 | 0.00024737 |
| AT5G62020 | 3.222036681 | 8.416E-160 | 6.714E-158 |
| AT5G62040 | 4.92045128 | 2.7671E-25 | 2.5053E-24 |
| AT5G62090 | 1.576679741 | 3.2877E-61 | 7.6231E-60 |
| AT5G62100 | 2.149432539 | 1.2128E-40 | 1.7659E-39 |
| AT5G62130 | 1.562973758 | 7.4078E-19 | 5.2134E-18 |
| AT5G62210 | -1.895685036 | 1.9525E-94 | 7.7746E-93 |
| AT5G62220 | 1.182345635 | 2.118E-36 | 2.7504E-35 |
| AT5G62280 | -1.933729707 | 2.6075E-07 | 8.9328E-07 |
| AT5G62310 | -1.743017284 | 0.0004441 | 0.0010578 |
| AT5G62340 | -3.489830354 | 1.648E-17 | 1.0842E-16 |
| AT5G62350 | -1.154423126 | 1.0878E-33 | 1.2934E-32 |
| AT5G62360 | -4.065615679 | 2.741E-107 | 1.277E-105 |
| AT5G62430 | 1.969103442 | 4.5204E-22 | 3.6265E-21 |
| AT5G62460 | 1.450735978 | 7.7844E-30 | 8.284E-29 |
| AT5G62470 | 3.613134312 | 1.661E-227 | 2.129E-225 |
| AT5G62480 | 1.367085872 | 7.9702E-17 | 5.0485E-16 |
| AT5G62490 | 4.654561842 | 2.6139E-08 | 9.7945E-08 |
| AT5G62520 | 3.418031483 | 3.3024E-66 | 8.4188E-65 |
| AT5G62540 | 2.124966679 | 1.303E-111 | 6.327E-110 |
| AT5G62610 | 1.141064092 | 5.5148E-28 | 5.5528E-27 |
| AT5G62630 | -1.59453754 | 3.2077E-28 | 3.2585E-27 |
| AT5G62710 | -1.187961468 | 5.0699E-12 | 2.4549E-11 |
| AT5G62730 | -5.478156141 | 7.7709E-06 | 2.2999E-05 |
| AT5G62760 | 1.111289101 | 6.3585E-11 | 2.8796E-10 |
| AT5G62770 | 1.005797713 | 1.3095E-05 | 3.791E-05 |
| AT5G62800 | 7.679336434 | 2.4762E-06 | 7.691E-06 |
| AT5G62840 | -1.388020203 | 2.6183E-18 | 1.7972E-17 |
| AT5G63040 | -1.18335512 | 7.2897E-14 | 3.9686E-13 |
| AT5G63087 | -6.895179635 | 9.371E-05 | 0.00024502 |
| AT5G63100 | -1.340737051 | 4.1935E-08 | 1.543E-07 |
| AT5G63130 | 3.743801501 | 1.3006E-93 | 5.125E-92 |
| AT5G63135 | 1.75466704 | 5.506E-17 | 3.5238E-16 |
| AT5G63140 | -2.003493465 | 2.9796E-24 | 2.6147E-23 |
| AT5G63160 | 2.535986015 | 2.086E-160 | 1.682E-158 |
| AT5G63180 | -3.439040819 | 1.753E-140 | 1.14E-138 |
| AT5G63190 | 2.302115685 | 2.2168E-65 | 5.5765E-64 |
| AT5G63270 | -7.331346182 | 1.3996E-05 | 4.0411E-05 |
| AT5G63350 | 7.44320765 | 4.8701E-87 | 1.7526E-85 |
| AT5G63370 | 1.493972134 | 1.3022E-89 | 4.8786E-88 |
| AT5G63595 | -2.081680225 | 1.3069E-05 | 3.7841E-05 |
| AT5G63600 | -1.021573699 | 2.7442E-38 | 3.7582E-37 |
| AT5G63640 | 1.298494577 | 1.6202E-31 | 1.8129E-30 |
| AT5G63660 | -2.590409339 | 1.0195E-09 | 4.2816E-09 |
| AT5G63780 | -1.803261371 | 1.5592E-47 | 2.6849E-46 |
| AT5G63790 | 1.223959947 | 3.7274E-85 | 1.2847E-83 |
| AT5G63880 | 1.445675137 | 1.7767E-24 | 1.5706E-23 |
| AT5G63905 | 1.167125037 | 8.9178E-06 | 2.6251E-05 |
| AT5G64080 | 1.715652844 | 2.3433E-71 | 6.4338E-70 |
| AT5G64100 | -2.172255288 | 1.085E-166 | 9.201E-165 |
| AT5G64120 | -5.740278106 | 0 | 0 |
| AT5G64170 | 2.206555919 | 6.2682E-81 | 1.9918E-79 |
| AT5G64180 | 1.198129418 | 5.9724E-13 | 3.0705E-12 |
| AT5G64210 | 5.806194214 | 1.8226E-16 | 1.1331E-15 |
| AT5G64230 | 1.666549073 | 9.8619E-33 | 1.1457E-31 |
| AT5G64250 | 1.941694094 | 5.164E-166 | 4.332E-164 |
| AT5G64260 | 2.053163604 | 3.706E-194 | 3.915E-192 |
| AT5G64310 | 2.450745437 | 2.005E-233 | 2.659E-231 |
| AT5G64410 | -1.081340716 | 1.1446E-10 | 5.1096E-10 |
| AT5G64430 | 2.650205105 | 7.989E-187 | 7.891E-185 |
| AT5G64530 | 1.361273132 | 0.00084601 | 0.0019347 |
| AT5G64570 | -1.324351395 | 1.1992E-20 | 9.0508E-20 |
| AT5G64580 | -1.377162286 | 4.4045E-54 | 8.8306E-53 |
| AT5G64620 | -1.048299315 | 3.5318E-14 | 1.9559E-13 |
| AT5G64670 | -1.298886274 | 5.1341E-35 | 6.3388E-34 |
| AT5G64750 | 1.618656898 | 6.8231E-05 | 0.00018165 |
| AT5G64780 | 1.99955813 | 1.7918E-14 | 1.0094E-13 |
| AT5G64810 | 2.193170395 | 3.8501E-16 | 2.3608E-15 |
| AT5G64840 | 1.537863463 | 1.423E-87 | 5.1616E-86 |
| AT5G64900 | -1.469393315 | 0.00497508 | 0.01003635 |
| AT5G64980 | -1.26724752 | 9.837E-05 | 0.00025683 |
| AT5G65010 | -1.68914076 | 2.299E-149 | 1.644E-147 |
| AT5G65040 | 1.72072639 | 6.0707E-16 | 3.6818E-15 |
| AT5G65090 | -1.774529655 | 0.00127994 | 0.00284574 |
| AT5G65110 | 2.009538651 | 6.441E-190 | 6.62E-188 |
| AT5G65140 | 3.318021027 | 6.702E-174 | 6.02E-172 |
| AT5G65160 | -1.402234437 | 3.4031E-07 | 1.1529E-06 |
| AT5G65207 | 3.812927271 | 1.1242E-85 | 3.922E-84 |
| AT5G65280 | 1.107221695 | 2.7328E-13 | 1.4317E-12 |
| AT5G65300 | 1.793139343 | 1.0159E-16 | 6.4064E-16 |
| AT5G65310 | -1.124528702 | 2.2806E-47 | 3.9152E-46 |
| AT5G65340 | -2.183455682 | 0.0066904 | 0.01317864 |
| AT5G65410 | -2.046854624 | 1.3084E-38 | 1.8048E-37 |
| AT5G65470 | 1.823295613 | 3.9931E-98 | 1.6717E-96 |
| AT5G65530 | -1.529818316 | 1.6511E-08 | 6.2903E-08 |
| AT5G65575 | 1.064224488 | 0.00366689 | 0.00756865 |
| AT5G65600 | -1.777133745 | 0.00030474 | 0.00074345 |
| AT5G65683 | -2.31961887 | 7.8989E-55 | 1.6063E-53 |
| AT5G65690 | -3.610190179 | 1.3691E-11 | 6.4686E-11 |
| AT5G65700 | -1.392420537 | 2.7072E-57 | 5.8603E-56 |
| AT5G65730 | -4.799049562 | 4.0888E-78 | 1.2489E-76 |
| AT5G65810 | -1.087742799 | 6.5368E-16 | 3.956E-15 |
| AT5G65840 | 1.332356914 | 5.0737E-48 | 8.857E-47 |
| AT5G65860 | -1.005336791 | 1.8304E-12 | 9.1065E-12 |
| AT5G65880 | -1.137877496 | 5.5434E-07 | 1.8365E-06 |
| AT5G65910 | 1.480473642 | 3.0647E-45 | 4.991E-44 |
| AT5G65970 | -2.645881332 | 1.0782E-23 | 9.2451E-23 |
| AT5G65990 | 2.194286139 | 2.705E-148 | 1.923E-146 |
| AT5G66000 | -1.672201398 | 8.0278E-07 | 2.6196E-06 |
| AT5G66020 | 6.186638794 | 0.00131919 | 0.00292789 |
| AT5G66050 | 1.323919009 | 2.4338E-35 | 3.0477E-34 |
| AT5G66052 | 2.426101579 | 2.9507E-62 | 7.0129E-61 |
| AT5G66070 | 2.630997192 | 1.065E-99 | 4.5688E-98 |
| AT5G66080 | 1.683204349 | 9.3802E-18 | 6.2505E-17 |
| AT5G66110 | 5.511705575 | 4.0208E-19 | 2.8606E-18 |
| AT5G66180 | 1.214534969 | 1.6609E-13 | 8.8212E-13 |
| AT5G66230 | -1.384451319 | 2.2982E-19 | 1.6521E-18 |
| AT5G66250 | 1.062512132 | 3.3569E-14 | 1.8631E-13 |
| AT5G66300 | 1.119505311 | 0.000626 | 0.00145743 |
| AT5G66310 | -1.330059832 | 9.4677E-19 | 6.6242E-18 |
| AT5G66400 | 7.764469589 | 2.745E-231 | 3.579E-229 |
| AT5G66460 | 1.77060814 | 3.5519E-58 | 7.83E-57 |
| AT5G66470 | -1.12309592 | 2.2829E-32 | 2.6227E-31 |
| AT5G66480 | 1.514100208 | 1.4631E-07 | 5.139E-07 |
| AT5G66540 | -1.220610632 | 1.4798E-30 | 1.6047E-29 |
| AT5G66590 | -1.206684026 | 1.7041E-05 | 4.8717E-05 |
| AT5G66631 | -1.055788816 | 0.00759352 | 0.01480295 |
| AT5G66650 | 1.533008447 | 8.1837E-19 | 5.7417E-18 |
| AT5G66690 | -2.202206352 | 3.936E-99 | 1.6752E-97 |
| AT5G66700 | 2.206110274 | 5.2539E-07 | 1.7454E-06 |
| AT5G66760 | 1.457858884 | 2.808E-147 | 1.971E-145 |
| AT5G66770 | -1.822874076 | 2.5321E-32 | 2.9046E-31 |
| AT5G66780 | 8.490890687 | 1.997E-234 | 2.681E-232 |
| AT5G66815 | -6.618573149 | 0.00030095 | 0.00073463 |
| AT5G66816 | -6.168312611 | 0.00272365 | 0.0057513 |
| AT5G09735 | -6.148765122 | 0.0016632 | 0.00363691 |
| AT5G66920 | -1.464607242 | 2.3019E-34 | 2.7862E-33 |
| AT5G66930 | 1.298888701 | 7.0119E-20 | 5.1359E-19 |
| AT5G66940 | -1.472055247 | 0.00145934 | 0.00321704 |
| AT5G66960 | -1.094836549 | 0.00027179 | 0.0006678 |
| AT5G66985 | -1.937312811 | 9.1052E-08 | 3.2598E-07 |
| AT5G67030 | 2.190136224 | 1.27E-242 | 1.834E-240 |
| AT5G67080 | 4.479551917 | 1.4294E-17 | 9.4395E-17 |
| AT5G67090 | 2.425738521 | 4.1529E-20 | 3.0694E-19 |
| AT5G67140 | 2.276781193 | 4.1993E-15 | 2.4454E-14 |
| AT5G67150 | -1.735376974 | 4.2085E-25 | 3.7878E-24 |
| AT5G67180 | 2.142994977 | 9.7346E-26 | 8.9921E-25 |
| AT5G67200 | -1.757734311 | 4.0539E-18 | 2.7495E-17 |
| AT5G67230 | 1.044433534 | 0.00014536 | 0.00037163 |
| AT5G67245 | 1.561769243 | 2.0359E-06 | 6.3796E-06 |
| AT5G67280 | -1.78029606 | 5.4241E-40 | 7.7688E-39 |
| AT5G67300 | 1.41345908 | 4.5357E-59 | 1.0106E-57 |
| AT5G67310 | 3.432039229 | 1.3856E-06 | 4.4166E-06 |
| AT5G67340 | 1.061013423 | 1.951E-09 | 8.0176E-09 |
| AT5G67390 | -1.697262293 | 1.4505E-06 | 4.6054E-06 |
| AT5G67400 | -4.885848244 | 2.7786E-48 | 4.8841E-47 |
| AT5G67450 | -1.481334437 | 0.00290309 | 0.00609227 |
| AT5G67460 | 1.108871106 | 4.8603E-08 | 1.7774E-07 |
| AT5G67510 | -1.789743087 | 1.6012E-77 | 4.8582E-76 |
| AT5G67520 | 1.175174053 | 0.00204719 | 0.00441315 |
| AT5G67580 | 1.005468074 | 1.2095E-20 | 9.1254E-20 |
| AT5G67600 | 1.92973527 | 2.9572E-97 | 1.229E-95 |
