# Supplementary material for: MYB44-ENAP1/2 restricts HDT4 to regulate drought tolerance in Arabidopsis
Source: PLoS Genet. 2022 Nov 22;18(11):e1010473. doi: 10.1371/journal.pgen.1010473 (PMC9681084; doi:10.1371/journal.pgen.1010473)
Supplement: S4 Table — (DOCX) [file pgen.1010473.s010.docx]

Table S4. Primers:

| Primer names | Sequence (5’à3’) | Purpose |
| --- | --- | --- |
| MBY44 qF | TCTCCACCTGTTGTTACTGGGCTT | qPCR |
| MBY44 qR | TTGACTCGTG GCTACGGTTT GACT | qPCR |
| HDT4 qF1 | GGTATCGAGATTAAGCCAGGG | qPCR |
| HDT4 qR1 | CACCAATCTTCACATAAACGGC | qPCR |
| HDT4 qF2 | AAGATGTAGAGGCTGAAGCAC | qPCR |
| HDT4 qR2 | GAGGGACCACAAGGGAATG | qPCR |
| RAB18 qF | ATGACGAGTACGGAAATCCGA | qPCR |
| RAB18 qF | CTTGGCCACCTGTTCCGTAT | qPCR |
| RD29A qF | TGGACACGAATTCTCCATCA | qPCR |
| RD29AqR | TTCCAGCTCAGCTCCTGATT | qPCR |
| RD29B qF | ACAAAGCTATAGCCGCAAAGA | qPCR |
| RD29B qR | ACTTTCTGCCCGTAAGCAGTA | qPCR |
| UGT74E2 qF | GCAACACTTTCGATAAATTGGAGG | qPCR |
| UGT74E2 qR | GCATTGAAGAGGCTAAAACCG | qPCR |
| RD28 qF | TTCATCCTTGTCTACTGCACC | qPCR |
| RD28 qR | CCACAAATAGCACCCAAACAC | qPCR |
| DREB2C qF | TCCAAATTCTAACTTGCTCGG | qPCR |
| DREB2C qR | CATTGAACCTGCGATGGTCT | qPCR |
| UBQ10 qF | CACACTCCACTTGGTCTTGCGT | qPCR |
| UBQ10 qR | TGGTCTTTCCGGTGAGAGTCTTCA | qPCR |
| UGT74E2 ChIP-qF | GCATCTCAGCCTCTCAGCTAT | ChIP-qCPR |
| UGT74E2 ChIP-qR | TTTGGGGACGAATTAACAGGTA | ChIP-qCPR |
| RD28 ChIP-qF | AGCTCCGTCAATTGCATCGG | ChIP-qCPR |
| RD28 ChIP-qR | TGGAATTGAGGAGTGAGAGCA | ChIP-qCPR |
| DREB2C ChIP-qF | GCTGGATC CAGAGTCCTTTC | ChIP-qCPR |
| DREB2C ChIP-qR | TCAACGAAAATGAGTAAATGACG | ChIP-qCPR |
| ENAP2 Y2H-F | TCC CCCGGG A ATGGAGACGACGACGCCGC | Cloning |
| ENAP2 Y2H-R | GG ACTAGT CTACAATTGCTTACCTGAAGCA | Cloning |
| MYB44 Y2H-F | ACGC GTCGAC C ATGGCTGATAGGATCAAAGGT | Cloning |
| MYB44 Y2H-R | GG ACTAGT CTACTCGATT CTCCCAACTC | Cloning |
| MYB44 Y2H-F1 | ACGC GTCGAC C ATG GACCATCGGGGTTACGATG | Cloning |
| MYB44 Y2H-R1 | GG ACTAGT CTA GTAACCGCCG CATTTCCTC | Cloning |
| ANAC102 Y2H-F | ACGC GTCGAC C ATGGACTTTGCTCTCTTCTCCT | Cloning |
| ANAC102 Y2H-R | GG ACTAGT TTACCCTTGAGGAGCAAAATTCC | Cloning |
| ZAT6 Y2H-F | TCC CCCGGG A ATGGCACTTGAAACTCTTACTTCT | Cloning |
| ZAT6 Y2H-R | GG ACTAGT TTAGGGTTTCTCCGGGAAGTC | Cloning |
| DIV2 Y2H-F | ACGC GTCGAC C ATGGCGTCAAGTCAGTGGA | Cloning |
| DIV2 Y2H-R | GG ACTAGT TCACATCCGAAACCCAAAATCC | Cloning |
| GBF2 Y2H-F | ACGC GTCGAC C ATGGGTAGCAACGAAGAAGG | Cloning |
| GBF2 Y2H-R | GG ACTAGT TCAGCTAGCCGCGACAG | Cloning |
| GBF3 Y2H-F | TCC CCCGGG A ATGGGAAATAGCAGCGAGGAA | Cloning |
| GBF3 Y2H-R | GG ACTAGT TCAGCCTGCAGCTACTGC | Cloning |
| HAT22 Y2H-F | ACGC GTCGAC C ATGGGTCTTGATGATTCATGCAAC | Cloning |
| HAT22 Y2H-R | GG ACTAGT CTAACATGCTGCAGAAGGATTAGT | Cloning |
| HB5 Y2H-F | ACGC GTCGAC C ATGAAGAGATCACGTGGAAGCT | Cloning |
| HB5 Y2H-R | GG ACTAGT TTACGAATTCCACTGATCGGAG | Cloning |
| HB6 Y2H-F | ACGC GTCGAC C ATGATGAAGAGATTAAGTAGTTCAGATTCAG | Cloning |
| HB6 Y2H-R | GG ACTAGT TCAATTCCAATGATCAACGGTGG | Cloning |
| NF-YB2 Y2H-F | ACGC GTCGAC C ATGGGGGATTCCGACAG | Cloning |
| NF-YB2 Y2H-R | GG ACTAGT TTAAGTCCTTGTCCTACCGGAGG | Cloning |
| NF-YC2 Y2H-F | ACGC GTCGAC C ATGGAGCAGTCAGAAGAGG | Cloning |
| NF-YC2 Y2H-R | GG ACTAGT TTAAGACTCATCAGGGTGTTGCT | Cloning |
| RD26 Y2H-F | ACGC GTCGAC C ATGGGTGTTAGAGAGAAAGATCCG | Cloning |
| RD26 Y2H-R | GG ACTAGT TCATTGCCTAAACTCGAATGTTTGAC | Cloning |
| pUBQ10-F | CCG GAATTC TCGACGAGTCAGTAATAAACG | Cloning |
| pUBQ10-Myc-R | CGG GGTACC AAGATCCTCCTCAGAAATCAACTTTTGCTC CAT CTGTTAATCAGAAAAACTCAGAT | Cloning |
| MYB44oe-F | CGG GGTACC ATGGCTGATAGGATCAAAGGT | Cloning |
| MYB44oe-3UTR-R | ACGCGTCGAC GTACATGAGTTTTTAATTCCTATAA | Cloning |
| HDT4oe-F | GGGGTACCATGGAGTTTTGGGGTATCG | Cloning |
| HDT4oe-Flag-TAA-R | ACGCGTCGACCTACTTGTCGTCGTCGTCCTTGTAGTCCTTTTTGCAAGAGGGACCAC | Cloning |
| pENTR-ENAP2-F | CACC ATGGAGACGACGACGCCGC | Cloning |
| pENTR-ENAP2-R | CTACAATTGCTTACCTGAAGCA | Cloning |
| pENTR-MYB44-F | CACC ATGGCTGATAGGATCAAAGG | Cloning |
| pENTR-MYB44-R | CTACTCGATTCTCCCAACTCCAA | Cloning |
| GST-MYB44-F | CCGGAATTC TA ATGGCTGATAGGATCAAAGGT | Cloning |
| GST-MYB44-R | ACGCGTCGAC CTACTCGATT CTCCCAACTC | Cloning |
| His-HDT4-F | CGTCGGATCC ATGGAGTTTTGGGGTATCG | Cloning |
| His-HDT4-R | CACCGTCGAC CTTTTTGCAAGAGGGACCAC | Cloning |
| LP(SALK_039074) | TTGTCAATTTGTCATGCACTG | Genotyping |
| RP(SALK_039074) | CACAGGCTTGAAAAGCTCAAC | Genotyping |
| ENAP1 CRISPR-F | CTTATCCTAAGCCTCTTTCTCC | Genotyping |
| ENAP1 CRISPR-R | CCAATTCGATAAAGGTTTCGCC | Genotyping |
| LP(SALKseq_083981.2) | AAACGCTCCAATACGGTCAG | Genotyping |
| RP(SALKseq_083981.2) | CAAAAGCAGAGAGGCACATG | Genotyping |
| LP(SALKseq_083981.3) | TCCTCTTTGCTTCACCATGG | Genotyping |
| RP(SALKseq_083981.3) | ACACTACTCCCGACGAATGG | Genotyping |
| LP(SALKseq_127604.1) | ATTCATGGTCCATGCCTCTC | Genotyping |
| RP(SALKseq_127604.1) | CTTTTTGCAAGAGGGACCAC | Genotyping |
